# Sexually Transmitted Infections Treatment Guidelines, 2021

**DOI:** 10.15585/mmwr.rr7004a1

**Published:** 2021-07-23

**Authors:** Kimberly A. Workowski, Laura H. Bachmann, Philip A. Chan, Christine M. Johnston, Christina A. Muzny, Ina Park, Hilary Reno, Jonathan M. Zenilman, Gail A. Bolan

**Affiliations:** ^1^Division of STD Prevention, National Center for HIV/AIDS, Viral Hepatitis, STD, and TB Prevention, CDC, Atlanta, Georgia; ^2^Emory University, Atlanta, Georgia; ^3^Brown University, Providence, Rhode Island; ^4^University of Washington, Seattle, Washington; ^5^University of Alabama at Birmingham, Birmingham, Alabama; ^6^University of California San Francisco, San Francisco, California; ^7^Washington University, St. Louis, Missouri; ^8^Johns Hopkins University, Baltimore, Maryland

## Abstract

These guidelines for the treatment of persons who have or are at risk for sexually transmitted infections (STIs) were updated by CDC after consultation with professionals knowledgeable in the field of STIs who met in Atlanta, Georgia, June 11–14, 2019. The information in this report updates the 2015 guidelines. These guidelines discuss 1) updated recommendations for treatment of *Neisseria gonorrhoeae, Chlamydia trachomatis, *and* Trichomonas vaginalis*; 2) addition of metronidazole to the recommended treatment regimen for pelvic inflammatory disease; 3) alternative treatment options for bacterial vaginosis; 4) management of *Mycoplasma genitalium*; 5) human papillomavirus vaccine recommendations and counseling messages; 6) expanded risk factors for syphilis testing among pregnant women; 7) one-time testing for hepatitis C infection; 8) evaluation of men who have sex with men after sexual assault; and 9) two-step testing for serologic diagnosis of genital herpes simplex virus. Physicians and other health care providers can use these guidelines to assist in prevention and treatment of STIs.

## Introduction

The term “sexually transmitted infection” (STI) refers to a pathogen that causes infection through sexual contact, whereas the term “sexually transmitted disease” (STD) refers to a recognizable disease state that has developed from an infection. Physicians and other health care providers have a crucial role in preventing and treating STIs. These guidelines are intended to assist with that effort. Although the guidelines emphasize treatment, prevention strategies and diagnostic recommendations also are discussed.

This report updates *Sexually Transmitted Diseases Treatment Guidelines, 2015* (*1*) and should be regarded as a source of clinical guidance rather than prescriptive standards. Health care providers should always consider the clinical circumstances of each person in the context of local disease prevalence. These guidelines are applicable to any patient care setting that serves persons at risk for STIs, including family planning clinics, HIV care clinics, correctional health care settings, private physicians’ offices, Federally Qualified Health Centers, clinics for adolescent care, and other primary care facilities. These guidelines are focused on treatment and counseling and do not address other community services and interventions that are essential to STI and HIV prevention efforts.

These STI treatment guidelines complement *Recommendations for Providing Quality Sexually Transmitted Diseases Clinical Services, 2020* (*2*) regarding quality clinical services for STIs in primary care and STD specialty care settings. This guidance specifies operational determinants of quality services in various clinical settings, describes on-site treatment and partner services, and indicates when STI-related conditions should be managed through consultation with or referral to a specialist.

## Methods

These guidelines were developed by CDC staff who worked with subject matter experts with expertise in STI clinical management from other federal agencies, nongovernmental academic and research institutions, and professional medical organizations. CDC staff identified governmental and nongovernmental subject matter experts on the basis of their expertise and assisted them in developing questions to guide individual literature reviews. CDC staff informed the subject matter experts that they were being consulted to exchange information and observations and to obtain their individual input. All subject matter experts disclosed potential conflicts of interest. STI Treatment Guidelines, 2021, Work Group members are listed at the end of this report.

In 2018, CDC staff identified key questions about treatment and clinical management to guide an update of the 2015 STD treatment guidelines (*1*). To answer these questions and synthesize new information available since publication of the 2015 guidelines, subject matter experts and CDC staff collaborated to conduct systematic literature reviews by using an extensive MEDLINE database evidence-based approach for each section of the 2015 guidelines (e.g., using English-language published abstracts and peer reviewed journal articles). These systematic reviews were focused on four principal outcomes of STI therapy for each disease or infection: 1) treatment of infection on the basis of microbiologic eradication; 2) alleviation of signs and symptoms; 3) prevention of sequelae; and 4) prevention of transmission, including advantages (e.g., cost-effectiveness, single-dose formulations, and directly observed therapy) and disadvantages (e.g., adverse effects) of specific regimens. The outcome of the literature reviews guided development of background materials, including tables of evidence from peer-reviewed publications summarizing the type of study (e.g., randomized controlled trial or case series), study population and setting, treatments or other interventions, outcome measures assessed, reported findings, and weaknesses and biases in study design and analysis.

In June 2019, the subject matter experts presented their assessments of the literature reviews at an in-person meeting of governmental and nongovernmental participants. Each key question was discussed and pertinent publications were reviewed in terms of strengths, weaknesses, and relevance. Participants evaluated the quality of evidence, provided their input, and discussed findings in the context of the modified rating system used by the U.S. Preventive Services Task Force (USPSTF). The discussions were informal and not structured to reach consensus. CDC staff also reviewed the publications from other professional organizations, including the American College of Obstetricians and Gynecologists (ACOG), USPSTF, the American Cancer Society (ACS), the American Society for Colposcopy and Cervical Pathology (ASCCP), and the Advisory Committee on Immunization Practices (ACIP). The discussion culminated in a list of participants’ opinions on all the key STI topic areas for consideration by CDC. (More detailed descriptions of the key questions, search terms, systematic search, evidence tables, and review process are available at https://www.cdc.gov/std/treatment-guidelines/default.htm).

CDC staff then independently reviewed the tables of evidence prepared by the subject matter experts, individual comments from the participants and professional organizations, and existing guidelines from other organizations to determine whether revisions to the 2015 STD treatment guidelines were warranted. CDC staff ranked evidence as high, medium, and low on the basis of each study’s strengths and weaknesses according to the USPSTF ratings (https://www.uspreventiveservicestaskforce.org/uspstf/us-preventive-services-task-force-ratings). CDC staff then developed draft recommendations that were peer reviewed by public health and clinical experts as defined by the Office of Management and Budget for influential scientific information. A public webinar was held to provide an overview of the draft recommendations and invite questions and comments on the draft recommendations. The peer review comments, webinar, questions, and responses were considered by CDC staff in developing the final recommendations for the updated STI treatment guidelines. Recommendations for HIV, hepatitis C, cervical cancer screening, STI screening in pregnancy, human papillomavirus (HPV) testing, and hepatitis A virus (HAV) and hepatitis B virus (HBV) vaccination were developed after CDC staff reviewed existing published recommendations. The English-language literature was searched periodically by CDC staff to identify subsequently published articles warranting consideration.

Throughout this report, the evidence used as the basis for specific recommendations is discussed briefly. Publication of comprehensive, annotated discussions of such evidence is planned in a supplemental issue of the journal *Clinical Infectious Diseases* after publication of the treatment guidelines. When more than one therapeutic regimen is recommended and the listed regimens have similar efficacy and similar rates of intolerance or toxicity, the recommendations are listed alphabetically. If differences are specified, regimens are prioritized on the basis of these differences. Recommended regimens should be used primarily; alternative regimens can be considered in instances of notable drug allergy or other medical contraindications to the recommended regimens. Alternative regimens are considered inferior to recommended regimens on the basis of available evidence regarding the principal outcomes and disadvantages of the regimens.

## Clinical Prevention Guidance

Prevention and control of STIs are based on the following five major strategies (*3*):

1. Accurate risk assessment and education and counseling of persons at risk regarding ways to avoid STIs through changes in sexual behaviors and use of recommended prevention services
2. Pre-exposure vaccination for vaccine-preventable STIs
3. Identification of persons with an asymptomatic infection and persons with symptoms associated with an STI
4. Effective diagnosis, treatment, counseling, and follow-up of persons who are infected with an STI
5. Evaluation, treatment, and counseling of sex partners of persons who are infected with an STI

### STI and HIV Infection Risk Assessment

Primary prevention of STIs includes assessment of behavioral risk (i.e., assessing the sexual behaviors that can place persons at risk for infection) and biologic risk (i.e., testing for risk markers for STI and HIV acquisition or transmission). As part of the clinical encounter, health care providers should routinely obtain sexual histories from their patients and address risk reduction as indicated in this report. Guidance for obtaining a sexual history is available at the Division of STD Prevention resource page (https://www.cdc.gov/std/treatment/resources.htm) and in the curriculum provided by the National Network of STD Clinical Prevention Training Centers (https://www.nnptc.org). Effective interviewing and counseling skills, characterized by respect, compassion, and a nonjudgmental attitude toward all patients, are essential to obtaining a thorough sexual history and delivering effective prevention messages. Effective techniques for facilitating rapport with patients include using open-ended questions (e.g., “Tell me about any new sex partners you’ve had since your last visit” and “What has your experience with using condoms been like?”); understandable, nonjudgmental language (e.g., “What gender are your sex partners?” and “Have you ever had a sore or scab on your penis?”); and normalizing language (e.g., “Some of my patients have difficulty using a condom with every sex act. How is it for you?”). The “Five P’s” approach to obtaining a sexual history is one strategy for eliciting information about the key areas of interest (Box 1). In addition, health care professionals can consider assessing sexual history by asking patients such questions as, “Do you have any questions or concerns about your sexual health?” Additional information about gaining cultural competency when working with certain populations (e.g., gay, bisexual, or other men who have sex with men [MSM]; women who have sex with women [WSW] or with women and men [WSWM]; or transgender men and women or adolescents) is available in sections of these guidelines related to these populations.

BOX 1 **The Five P’s approach for health care providers obtaining sexual histories: partners, practices, protection from sexually transmitted infections, past history of sexually transmitted infections, and pregnancy intention**

**1. Partners**

- “Are you currently having sex of any kind?”
- “What is the gender(s) of your partner(s)?”

**2. Practices**

- “To understand any risks for sexually transmitted infections (STIs), I need to ask more specific questions about the kind of sex you have had recently.”
- “What kind of sexual contact do you have or have you had?”
  º “Do you have vaginal sex, meaning ‘penis in vagina’ sex?”
  º “Do you have anal sex, meaning ‘penis in rectum/anus’ sex?”
  º “Do you have oral sex, meaning ‘mouth on penis/vagina’?”

**3. Protection from STIs**

- “Do you and your partner(s) discuss prevention of STIs and human immunodeficiency virus (HIV)?”
- “Do you and your partner(s) discuss getting tested?”
- For condoms:
  º “What protection methods do you use? In what situations do you use condoms?”

**4. Past history of STIs**

- “Have you ever been tested for STIs and HIV?”
- “Have you ever been diagnosed with an STI in the past?”
- “Have any of your partners had an STI?”

Additional questions for identifying HIV and viral hepatitis risk:

- “Have you or any of your partner(s) ever injected drugs?”
- “Is there anything about your sexual health that you have questions about?”

**5. Pregnancy intention**

- “Do you think you would like to have (more) children in the future?”
- “How important is it to you to prevent pregnancy (until then)?”
- “Are you or your partner using contraception or practicing any form of birth control?”
- “Would you like to talk about ways to prevent pregnancy?”

In addition to obtaining a behavioral risk assessment, a comprehensive STI and HIV risk assessment should include STI screening as recommended in these guidelines because STIs are biologic markers of risk, particularly for HIV acquisition and transmission among certain MSM. In most clinical settings, STI screening is an essential and underused component of an STI and HIV risk assessment. Persons seeking treatment or evaluation for a particular STI should be screened for HIV and other STIs as indicated by community prevalence and individual risk factors (see Chlamydial Infections; Gonococcal Infections; Syphilis). Persons should be informed about all the tests for STIs they are receiving and notified about tests for common STIs (e.g., genital herpes, trichomoniasis, *Mycoplasma genitalium*, and HPV) that are available but not being performed and reasons why they are not always indicated. Persons should be informed of their test results and recommendations for future testing. Efforts should be made to ensure that all persons receive STI care regardless of personal circumstances (e.g., ability to pay, citizenship or immigration status, gender identity, language spoken, or specific sex practices).

### STI and HIV Infection Prevention Counseling

After obtaining a sexual history from their patients, all providers should encourage risk reduction by offering prevention counseling. Prevention counseling is most effective if provided in a nonjudgmental and empathetic manner appropriate to the patient’s culture, language, sex and gender identity, sexual orientation, age, and developmental level. Prevention counseling for STIs and HIV should be offered to all sexually active adolescents and to all adults who have received an STI diagnosis, have had an STI during the previous year, or have had multiple sex partners. USPSTF recommends intensive behavioral counseling for all sexually active adolescents and for adults at increased risk for STIs and HIV (*4*). Such interactive counseling, which can be resource intensive, is directed at a person’s risk, the situations in which risk occurs, and the use of personalized goal-setting strategies. One such approach, known as client-centered STI and HIV prevention counseling, involves tailoring a discussion of risk reduction to the person’s situation. Although one large study in STI clinics (Project RESPECT) demonstrated that this approach was associated with lower acquisition of curable STIs (e.g., trichomoniasis, chlamydia, gonorrhea, and syphilis) (*5*), another study conducted 10 years later in the same settings but different contexts (Project AWARE) did not replicate this result (*6*).

With the challenges that intensive behavioral counseling poses, health care professionals might find brief prevention messages and those delivered through video or in a group session to be more accessible for the client. A review of 11 studies evaluated brief prevention messages delivered by providers and health counselors and reported them to be feasible and to decrease subsequent STIs in STD clinic settings (*7*) and HIV care settings (*8*). Other approaches use motivational interviewing to move clients toward achievable risk-reduction goals. Client-centered counseling and motivational interviewing can be used effectively by clinicians and staff trained in these approaches. CDC provides additional information on these and other effective behavioral interventions at https://www.cdc.gov/std/program/interventions.htm. Training in client-centered counseling and motivational interviewing is available through the STD National Network of Prevention Training Centers (https://www.nnptc.org).

In addition to one-on-one STI and HIV prevention counseling, videos and large group presentations can provide explicit information concerning STIs and reducing disease transmission (e.g., how to use condoms consistently and correctly and the importance of routine screening). Group-based strategies have been effective in reducing the occurrence of STIs among persons at risk, including those attending STD clinics (*9*). Brief, online, electronic-learning modules for young MSM have been reported to be effective in reducing incident STIs and offer a convenient client platform for effective interventions (*10*). Because the incidence of certain STIs, most notably syphilis, is higher among persons with HIV infection, use of client-centered STI counseling for persons with HIV continues to be encouraged by public health agencies and other health organizations (https://www.cdc.gov/std/statistics/2019/default.htm). A 2014 guideline from CDC, the Health Resources and Services Administration, and the National Institutes of Health recommends that clinical and nonclinical providers assess a person’s behavioral and biologic risks for acquiring or transmitting STIs and HIV, including having sex without condoms, having recent STIs, and having partners recently treated for STIs (https://stacks.cdc.gov/view/cdc/44064). That federal guideline is for clinical and nonclinical providers to offer or make referral for regular screening for multiple STIs, on-site STI treatment when indicated, and risk-reduction interventions tailored to the person’s risks. Brief risk-reduction counseling delivered by medical providers during HIV primary care visits, coupled with routine STI screening, has been reported to reduce STI incidence among persons with HIV infection (*8*). Other specific methods have been designed for the HIV care setting (https://www.cdc.gov/hiv/effective-interventions/index.html).

### Primary Prevention Methods

#### Pre-Exposure Vaccination

Pre-exposure vaccination is one of the most effective methods for preventing transmission of HPV, HAV, and HBV, all of which can be sexually transmitted. HPV vaccination is recommended routinely for males and females aged 11 or 12 years and can be administered beginning at age 9 years. HPV vaccination is recommended through age 26 years for those not previously vaccinated (*11*). Sharing clinical decision-making about HPV vaccination is recommended for certain adults aged 27–45 years who are not adequately vaccinated in accordance with existing guidance (https://www.cdc.gov/vaccines/hcp/acip-recs/vacc-specific/hpv.html).

Hepatitis B vaccination is recommended for all unvaccinated, uninfected persons who are sexually active with more than one partner or are being evaluated or treated for an STI (*12*). In addition, hepatitis A and B vaccines are recommended for MSM, persons who inject drugs, persons with chronic liver disease, and persons with HIV or hepatitis C infections who have not had hepatitis A or hepatitis B (*12*). HAV vaccine is also recommended for persons who are homeless (*13*). Details regarding HAV and HBV vaccination, including routine childhood vaccination, are available at https://www.cdc.gov/hepatitis and at the ACIP website (https://www.cdc.gov/vaccines/hcp/acip-recs/vacc-specific/index.html).

#### Condoms

##### External Condoms

When used consistently and correctly, external latex condoms, also known as male condoms, are effective in preventing the sexual transmission of HIV infection (http://www.ashasexualhealth.org/pdfs/Male_and_Female_Condoms.pdf). In heterosexual HIV mixed-status relationships (i.e., those involving one infected and one uninfected partner) in which condoms were used consistently, HIV-negative partners were 71%–80% less likely to become infected with HIV, compared with persons in similar relationships in which condoms were not used (*14*,*15*). Two analyses of MSM mixed-status couple studies estimated the protective effect of condom use to be 70% and 91%, respectively (*16*,*17*). Moreover, studies demonstrate that consistent condom use reduces the risk for other STIs, including chlamydia, gonorrhea, hepatitis B, and trichomoniasis (*18*–*21*). By limiting lower genital tract infections, condoms also might reduce the risk for pelvic inflammatory disease (PID) among women (*22*). In addition, consistent and correct use of latex condoms reduces the risk for HPV infection and HPV-associated diseases, genital herpes, syphilis, and chancroid when the infected area or site of potential exposure is covered (*23*–*27*). Additional information is available at https://www.cdc.gov/condomeffectiveness/index.html and www.factsaboutcondoms.com/professional.php. Condoms are regulated as medical devices and are subject to random sampling and testing by the Food and Drug Administration (FDA). Each latex condom manufactured in the United States is tested electronically for holes before packaging. The rate of condom breakage during sexual intercourse and withdrawal in the United States is approximately two broken condoms per 100 condoms. Rates of breakage and slippage might be slightly higher during anal intercourse (*28*,*29*). The failure of condoms to protect against STIs or unintended pregnancy usually results from inconsistent or incorrect use rather than condom breakage (*30*). Users should check the expiration or manufacture date on the box or individual package. Latex condoms should not be used beyond their expiration date or >5 years after the manufacturing date. Condoms made of materials other than latex are available in the United States and can be classified into two general categories: 1) polyurethane, polyisoprene, or other synthetic condoms and 2) natural membrane condoms.

Polyurethane external condoms provide protection against STIs and HIV and pregnancy comparable to that of latex condoms (*20*,*31*). These can be substituted for latex condoms by persons with latex sensitivity, are typically more resistant to deterioration, and are compatible with use of both oil-based and water-based lubricants. The effectiveness of other synthetic external condoms to prevent STIs has not been extensively studied, and FDA labeling restricts their recommended use to persons who are sensitive to or allergic to latex. Natural membrane condoms (frequently called natural skin condoms or [incorrectly] lambskin condoms) are made from lamb cecum and can have pores up to 1,500 nm in diameter. Although these pores do not allow the passage of sperm, they are more than 10 times the diameter of HIV and more than 25 times that of HBV. Moreover, laboratory studies demonstrate that sexual transmission of viruses, including HBV, herpes simplex virus (HSV), and HIV, can occur with natural membrane condoms (*31*). Therefore, natural membrane condoms are not recommended for prevention of STIs and HIV.

Providers should advise that condoms must be used consistently and correctly to be effective in preventing STIs and HIV while noting that any condom use is better than no condom use. Providing instructions about the correct use of condoms can be useful. Communicating the following recommendations can help ensure that patients use external condoms correctly:

- Use a new condom with each sex act (i.e., oral, vaginal, and anal).
- Carefully handle the condom to avoid damaging it with fingernails, teeth, or other sharp objects.
- Put the condom on after the penis is erect and before any genital, oral, or anal contact with the partner.
- Use only water-based or silicone-based lubricants (e.g., K-Y Jelly, Astroglide, AquaLube, or glycerin) with latex condoms. Oil-based lubricants (e.g., petroleum jelly, shortening, mineral oil, massage oils, body lotions, or cooking oil) can weaken latex and should not be used; however, oil-based lubricants typically can be used with polyurethane or other synthetic condoms.
- Ensure adequate lubrication during vaginal and anal sex, which might require using exogenous water-based lubricants.
- Hold the condom firmly against the base of the penis during withdrawal, and withdraw while the penis is still erect to prevent the condom from slipping off.

Additional information about external condoms is available at https://www.cdc.gov/condomeffectiveness.

##### Internal Condoms

Condoms for internal vaginal use, also known as female condoms, are available worldwide (e.g., the FC2 Female Condom, Reddy condom, Cupid female condom, and Woman’s condom) (*31*,*32*). Use of internal condoms can provide protection from acquisition and transmission of STIs, although data are limited. Internal condoms are more costly compared with external condoms; however, they offer the advantage of being controlled by the receptive partner as an STI and HIV prevention method, and the newer versions might be acceptable to all persons. Although the internal condom also has been used during receptive anal intercourse, efficacy associated with this practice remains unknown (*33*). Additional information about the internal condom is available at http://www.ashasexualhealth.org/pdfs/Male_and_Female_Condoms.pdf.

#### Cervical Diaphragms

In observational studies, diaphragm use has been demonstrated to protect against cervical gonorrhea, chlamydia, and trichomoniasis (*34*). However, a trial examining the effect of a diaphragm plus lubricant on HIV acquisition among women in Africa reported no additional protective effect when compared with the use of male condoms alone. Likewise, no difference by study arm in the rate of acquisition of chlamydia, gonorrhea, or herpes occurred (*35*,*36*). Diaphragms should not be relied on as the sole source of protection against HIV and other STIs.

#### Multipurpose Prevention Technologies

Methods that combine STI and HIV prevention with pregnancy prevention are known as multipurpose prevention technologies (MPTs) (*37*) (https://www.who.int/reproductivehealth/topics/linkages/mpts/en). Internal and external condoms are both examples of MPTs because they are effective prevention measures when used correctly for STI and HIV transmission or pregnancy prevention. The multicenter Evidence for Contraception Options and HIV Outcomes (ECHO) trial observed no statistically significant differences in HIV incidence rates among women randomly assigned to one of three contraceptive methods (depot medroxyprogesterone acetate [DMPA], levonorgestrel implant, and copper-containing intrauterine device [IUD]); however, rates of HIV infection were high in all groups, indicating a need for MPTs (*38*). Development of MPTs is complex and ongoing; products under study include microbicides with contraceptive devices (e.g., tenofovir with a vaginal ring contraceptive delivery package) and other innovative methods (*39*).

#### Topical Microbicides and Spermicides

Nonspecific topical microbicides are ineffective for preventing HIV infection (*40*–*45*). Tenofovir gel has been studied for prevention of herpes simplex virus 2 (HSV-2) and HIV infections (*46*,*47*). Adherence can be low (*48*), and prevention of HIV infection, especially among women, has not been demonstrated (*47*,*49*).Vaginal rings containing dapivirine have provided some reduction in HIV infection (*50*,*51*). For men and transgender women who have anal intercourse, tenofovir gel appears safe when applied before and after anal sex (*52*). Spermicides containing nonoxynol-9 (N-9) might disrupt genital or rectal epithelium and have been associated with an increased risk for HIV infection. Condoms with N-9 are no more effective than condoms without N-9; therefore, N-9 alone or in a condom is not recommended for STI and HIV prevention (*40*). N-9 use also has been associated with an increased risk for bacterial urinary tract infections among women (*53*,*54*).

#### Nonbarrier Contraception, Female Surgical Sterilization, and Hysterectomy

Contraceptive methods that are not mechanical barriers offer no protection against HIV or other STIs. The ECHO study observed no differences in HIV incidence rates among women randomly assigned to DMPA, levonorgestrel implant, or copper-containing IUD contraceptive methods (*38*). A systematic review of epidemiologic evidence reported that the majority of studies demonstrated no association between use of oral contraceptives and HIV acquisition among women (*55*). Whether hormonal contraception alters a woman’s risk for other STIs is uncertain (*56*,*57*).

Sexually active women who use contraceptive methods other than condoms should be counseled about STI and HIV infection prevention measures. These include pre-exposure prophylaxis (PrEP) and postexposure prophylaxis (PEP), limiting the number of sex partners, and correct and consistent use of condoms.

#### Emergency Contraception

Unprotected intercourse exposes women to risks for STIs and unplanned pregnancy. Providers should offer counseling about the option of emergency contraception if pregnancy is not desired. Options for emergency contraception in the United States include copper-containing IUDs and emergency contraceptive pills (ECPs) (*58*,*59*). More information is available at https://www.acog.org/clinical/clinical-guidance/practice-bulletin/articles/2015/09/emergency-contraception?utm_source=redirect&utm_medium=web&utm_campaign=otn. ECPs are available in the following formulations: ulipristal acetate in a single dose (30 mg) available by prescription, levonorgestrel in a single dose (1.5 mg) available over the counter or by prescription, or a combined estrogen and progestin pill regimen. Insertion of a copper-containing IUD ≤5 days after unprotected sex can reduce pregnancy risk from a sex act by approximately 99% (*60*). ECPs are most efficacious when initiated as soon as possible after unprotected sex. Ulipristal acetate is effective ≤5 days after unprotected sex, and levonorgestrel is most effective ≤3 days after unprotected sex but has some efficacy at ≤5 days. ECPs are ineffective (but not harmful) if the woman is already pregnant (*61*). A 2019 Cochrane review summarized the efficacy, safety, and convenience of different emergency contraception methods (*61*).

More information about emergency contraception is available in *Contraceptive Technology, 21st Edition* (*31*), in the 2016 U.S. Selected Practice Recommendations (U.S. SPR) for Contraceptive Use (emergency contraception) available at https://www.cdc.gov/reproductivehealth/contraception/mmwr/spr/emergency.html, and in the 2016 U.S. Medical Eligibility Criteria (U.S. MEC) for Contraceptive Use (copper IUDs for emergency contraception) available at https://www.cdc.gov/reproductivehealth/contraception/mmwr/mec/appendixj.html.

Providers should educate males and females about emergency contraception, especially if other methods of contraception were used incorrectly or not at all and pregnancy is not desired (*62*). An advance supply of ECPs can be provided or prescribed so that ECPs will be available when needed (*59*).

#### Male Circumcision

Male circumcision reduces the risk for HIV infection and certain STIs among heterosexual men. Three randomized, controlled trials performed in regions of sub-Saharan Africa, where generalized HIV epidemics involving predominantly heterosexual transmission were occurring, demonstrated that male circumcision reduces the risk for HIV acquisition among men by 50%–60% (*63*–*65*). In those trials, circumcision also was protective against other STIs, including high-risk genital HPV infection and genital herpes (*66*–*68*). Follow-up studies have demonstrated sustained benefit of circumcision for HIV prevention (*69*) and that the effect is not mediated solely through a reduction in HSV-2 infection or genital ulcer disease (GUD) (*70*).

The World Health Organization (WHO) and the Joint United Nations Programme on HIV/AIDS (UNAIDS) recommend that male circumcision efforts be scaled up as an effective intervention for preventing heterosexually acquired HIV infection (*71*) in countries with hyperendemic and generalized HIV epidemics within the context of ensuring universal access to comprehensive HIV prevention, treatment, care, and support (https://www.afro.who.int/publications/voluntary-medical-male-circumcision-hiv-prevention). In the United States, the American Academy of Pediatrics (AAP) recommends that newborn male circumcision be available to families that desire it because the benefits of the procedure, including prevention of penile cancers, urinary tract infections, GUD, and HIV infection, outweigh the risks. ACOG has also endorsed AAP’s policy statement. In light of these benefits, the American Urological Association states that male circumcision should be considered an option for risk reduction, among other strategies (*72*). Additional information for providers counseling male patients and parents regarding male circumcision for preventing HIV, STIs, and other adverse health outcomes is available at https://www.cdc.gov/hiv/risk/male-circumcision.html.

No definitive data exist to determine whether male circumcision reduces HIV acquisition among MSM, although one meta-analysis of 62 observational studies reported that circumcision was protective against HIV acquisition in low- to middle-income countries but not in high-income countries (*73*). Further studies are needed to confirm any potential benefit of male circumcision for this population.

#### Pre-Exposure Prophylaxis for HIV

Daily oral antiretroviral PrEP with a fixed-dose combination of emtricitabine (FTC) and either tenofovir disoproxil fumarate (TDF) or tenofovir alafenamide (TAF) have demonstrated safety (*74*) and a substantial reduction in the rate of HIV acquisition for MSM (*75*). TDF/FTC has demonstrated safety and efficacy for mixed-status heterosexual couples (*76*) and heterosexual men and women recruited individually (*77*); however, no evidence is yet available regarding TAF/FTC among heterosexually active women. In addition, one clinical trial involving persons who inject drugs (*78*) and one involving heterosexual mixed-status couples (*76*) demonstrated substantial efficacy and safety of daily oral PrEP with TDF alone. High adherence to oral PrEP was strongly associated with protection from HIV infection. Studies conducted with MSM have demonstrated that taking PrEP at specific times before and after sexual intercourse was effective in preventing HIV; however, less experience exits with this regimen, it is not FDA cleared, and it has not been studied among other populations (*79*).

Comprehensive clinical practice guidelines are available for providers in prescribing PrEP to reduce the risk for HIV infection (*80*). Among HIV-negative sexually active men and women, bacterial STIs are key indicators of risk for HIV acquisition. Studies have documented the risk for HIV acquisition among MSM within 1 year after infection with rectal gonorrhea or chlamydia (one in 15 men), primary or secondary syphilis (one in 18), and among men with no rectal STI or syphilis infection (one in 53) (*81*–*83*). Sexually active adults and adolescents should be screened for STIs (e.g., chlamydia, gonorrhea, and syphilis) in accordance with recommendations, and persons with infection should be offered PrEP. The USPSTF recommends that persons at risk for HIV acquisition be offered PrEP (*84*). Persons at risk for HIV acquisition include HIV-negative persons whose sexual partner or partners have HIV infection (especially if viral load is detectable or unknown), persons who have had gonorrhea or syphilis during the previous 6 months, and injecting drug users who share injection equipment (*84*). Clinical practice guidelines recommend STI screening for persons taking PrEP (*80*) because increased rates of STI acquisition have been described (*85*–*87*).

#### Pre-Exposure Prophylaxis for STIs

Providing HSV treatment to persons with HIV and HSV infection has not demonstrated benefit in reducing HIV acquisition among uninfected partners. A large randomized controlled trial evaluated mixed-status heterosexual couples among the partners with HIV infection who also were seropositive for HSV-2 (*88*). Use of acyclovir had no effect on HIV transmission. These findings are consistent with a previous trial that reported no benefit of acyclovir in preventing HIV acquisition among persons seropositive for HSV-2 (*89*).

Doxycycline prophylaxis has been examined for preventing bacterial STIs. In a pilot study, 30 MSM living with HIV with previous syphilis (two or more episodes since HIV diagnosis) were randomly assigned to doxycycline 100 mg for 48 weeks versus a financial incentive–based behavioral intervention (*90*). That study demonstrated a 73% reduction in any bacterial STI at any site, without substantial differences in sexual behavior. Additional studies examining doxycycline prophylaxis are under way or in development (*91*).

#### Postexposure Prophylaxis for HIV and STIs

Guidelines for using PEP aimed at preventing HIV and other STIs as a result of sexual exposure are available at https://www.cdc.gov/hiv/pdf/programresources/cdc-hiv-npep-guidelines.pdf. Sexually active persons seeking HIV PEP should be evaluated for PrEP after completing their PEP course and testing negative for HIV. HIV PEP is also discussed elsewhere in this report (see Sexual Assault and Abuse and STIs). Genital hygiene methods (e.g., vaginal washing and douching) after sexual exposure are ineffective in protecting against HIV and STIs and might increase the risk for bacterial vaginosis (BV), certain STIs, and HIV infection (*92*).

STI PEP in the form of doxycycline 200 mg taken after unprotected anal sex has been studied among MSM and transgender women; results demonstrated reduction in incident chlamydia and syphilis by 70% and 73%, respectively, but no effect on gonorrhea (*93*). Other studies are under way or in development regarding doxycycline prophylaxis for bacterial STIs (*91*). No long-term data are available regarding the impact of STI PEP on antimicrobial resistance and the microbiome. Further studies are needed to determine whether STI PEP is an effective and beneficial strategy for STI prevention.

#### HIV Treatment as Prevention: Antiretroviral Treatment of Persons with HIV to Prevent HIV Among Partners

In 2011, the randomized controlled trial HPTN 052 demonstrated that, among HIV mixed-status heterosexual couples, HIV antiretroviral therapy (ART) for the infected partner decreased the risk for transmission to the uninfected partner by 96% (*94*). Therefore, ART not only is beneficial to the health of persons with HIV infection, it also reduces the risk for transmission. Additional studies of HIV mixed-status couples, heterosexual and MSM couples (PARTNER study), and MSM couples (Opposites Attract and PARTNERS2 studies) reported that patients with HIV taking ART who maintain an undetectable viral load demonstrate no risk for transmitting HIV to their HIV-negative sex partners (*95*–*97*). For those reasons, ART should be offered to all persons with HIV infection to obtain viral suppression. Detailed guidance regarding ART regimens is available in the U.S. Department of Health and Human Services’ HIV treatment guidelines (*98*).

#### HIV Seroadaptive Strategies

Seroadaptive strategies for HIV prevention have largely originated within communities of MSM. They are predicated on knowledge of self and partner HIV status. One specific seroadaptive practice is serosorting, which includes limiting anal sex without a condom to partners with the same HIV status as their own or choosing to selectively use condoms with HIV mixed-status partners. Another practice among mixed-status couples is seropositioning, in which the person with HIV infection is the receptive partner for anal intercourse. Observational studies have consistently reported that serosorting confers greater risk for HIV infection than consistent condom use but has lower risk compared with anal intercourse without a condom and without serosorting (*99*–*101*). Serosorting practices have been associated with increased risk for STIs, including chlamydia and gonorrhea (*102*,*103*).

Serosorting is not recommended for the following reasons: many MSM who have HIV infection do not know they have HIV because they have not been tested recently, men’s assumptions about the HIV status of their partners might be wrong, and some men with HIV infection might not disclose or might misrepresent their HIV status. All of these factors increase the risk that serosorting can lead to HIV infection. Serosorting has not been studied among heterosexually active persons.

#### Abstinence and Reduction of Number of Sex Partners

Abstinence from oral, vaginal, and anal sex and participating in a long-term, mutually monogamous relationship with a partner known to be uninfected are prevention approaches to avoid transmission of STIs. For persons who are being treated for an STI (or whose partners are undergoing treatment), counseling that encourages abstinence from sexual intercourse until completion of the entire course of medication is vital for preventing reinfection. A trial conducted among women regarding the effectiveness of counseling messages when patients have cervicitis or vaginal discharge demonstrated that women whose sex partners have used condoms might benefit from a hierarchical message that includes condoms but women without such experience might benefit more from an abstinence-only message (*104*). A more comprehensive discussion of abstinence and other sexual practices that can help persons reduce their risk for STIs is available in *Contraceptive Technology, 21st Edition* (*31*).

### Partner Services

The term “partner services” refers to a continuum of clinical evaluation, counseling, diagnostic testing, and treatment designed to increase the number of infected persons brought to treatment and to reduce transmission among sexual networks. This continuum includes efforts of health departments, medical providers, and patients themselves. The term “public health partner services” refers to efforts by public health departments to identify the sex and needle-sharing partners of infected persons to ensure their medical evaluation and treatment. Health departments are increasingly incorporating referral to additional services, as indicated, into the partner services continuum. Aside from the general benefit to patients and partners, service referrals and linkage can mitigate the circumstances that increase risk for future STI and HIV acquisition.

The types and comprehensiveness of public health partner services and the specific STIs for which they are offered vary by public health agency, their resources, and the geographic prevalence of STIs. In most areas of the United States, health departments routinely attempt to provide partner services to all persons with infectious syphilis (primary or secondary) and persons with a new diagnosis of HIV infection. Health departments should provide partner services for persons who might have cephalosporin-resistant gonorrhea. In contrast, relatively few U.S. health departments routinely provide STI partner services to persons with gonorrhea, chlamydia, trichomoniasis, or other STIs (*105*). Because STI diagnoses often can serve as risk markers for HIV acquisition (*83*), public health services might include follow-up of MSM with an STI to offer HIV PrEP. Public health services can also include HIV and STI prevention interventions including HIV and STI testing, linkage and relinkage of persons with HIV infection to HIV care clinics, and referral of partners of persons with STIs or HIV infection to HIV PrEP, as indicated (*106*–*109*). Clinicians should familiarize themselves with public health practices in their area; however, in most instances, providers should understand that responsibility for discussing the treatment of partners of persons with STIs rests with the diagnosing provider and the patient. State laws require a good faith effort by the provider to inform partners, and providers should familiarize themselves with public health laws.

Clinicians who do not notify partners of patients directly can still provide partner services by counseling infected persons and providing them with written information and medication to give to their partners (if recommended and allowable by state law), directly evaluating and treating sex partners, and cooperating with state and local health departments. Clinicians’ efforts to ensure treatment of patients’ sex partners can reduce the risk for reinfection and potentially diminish transmission of STIs (*110*). Therefore, clinicians should encourage all persons with STIs to notify their sex partners and urge them to seek medical evaluation and treatment. Exceptions to this practice include circumstances posing a risk for intimate partner violence (*111*). Available data are limited regarding the rate of intimate partner violence directly attributable to partner notification (*112*,*113*); however, because of the reported prevalence of intimate partner violence in the general population (*114*), providers should consider the potential risk before notifying partners of persons or encouraging partner notification. Time spent counseling patients about the importance of notifying partners is associated with improved notification outcomes (*115*). When possible, clinicians should advise persons to bring their primary sex partner with them when returning for treatment and should concurrently treat both persons. Although this approach can be effective for a main partner (*116*,*117*), it might not be a feasible approach for additional sex partners. Evidence indicates that providing patients with written information to share with sex partners can increase rates of partner treatment (*110*).

Certain health departments now use technology (e.g., email, texting, mobile applications, and social media outlets) to facilitate partner services for locating and notifying the sex partners of persons with STIs, including HIV (*118*,*119*). Patients now have the option to use Internet sites to send anonymous email or text messages advising partners of their exposure to an STI (*120*); anonymous notification via the Internet is considered better than no notification at all. However, because the extent to which these sites affect partner notification and treatment is uncertain, patients should be encouraged to notify their partners in person or by telephone, email, or text message; alternatively, patients can authorize a medical provider or public health professional to notify their sex partners.

#### Expedited Partner Therapy

Expedited partner therapy (EPT) is a harm-reduction strategy and the clinical practice of treating the sex partners of persons with diagnosed chlamydia or gonorrhea, who are unable or unlikely to seek timely treatment, by providing medications or prescriptions to the patient as allowable by law. Patients then provide partners with these therapies without the health care provider having examined the partner (https://www.cdc.gov/std/ept). Unless prohibited by law or other regulations, medical providers should routinely offer EPT to patients with chlamydia when the provider cannot ensure that all of a patient’s sex partners from the previous 60 days will seek timely treatment. If the patient has not had sex during the 60 days before diagnosis, providers should offer EPT for the patient’s most recent sex partner. Because EPT must be an oral regimen and current gonorrhea treatment involves an injection, EPT for gonorrhea should be offered to partners unlikely to access timely evaluation after linkage is explored. EPT is legal in the majority of states but varies by chlamydial or gonococcal infection. Providers should visit https://www.cdc.gov/std/ept to obtain updated information for their state. Providing patients with packaged oral medication is the preferred approach because the efficacy of EPT using prescriptions has not been evaluated, obstacles to EPT can exist at the pharmacy level (*121*,*122*), and many persons (especially adolescents) do not fill the prescriptions provided to them by a sex partner (*123*,*124*). Medication or prescriptions provided for EPT should be accompanied by educational materials for the partner, including treatment instructions, warnings about taking medications (e.g., if the partner is pregnant or has an allergy to the medication), general health counseling, and a statement advising that partners seek medical evaluation as soon as possible for HIV infection and any symptoms of STIs, particularly PID.

Evidence supporting EPT is based on three U.S. clinical trials involving heterosexual men and women with chlamydia or gonorrhea (*125*–*127*). All three trials reported that more partners were treated when patients were offered EPT. Two reported statistically significant decreases in the rate of reinfection, and one observed a lower risk for persistent or recurrent infection that was statistically nonsignificant. A fourth trial in the United Kingdom did not demonstrate a difference in the risk for reinfection or in the numbers of partners treated between persons offered EPT and those advised to notify their sex partners (*128*). U.S. trials and a meta-analysis of EPT revealed that the magnitude of reduction in reinfection of index patients, compared with patient referral, differed according to the STI and the sex of the index patient (*110*,*125*–*127*). However, across trials, reductions in chlamydia prevalence at follow-up were approximately 20%, and reductions in gonorrhea were approximately 50% at follow-up.

Existing data indicate that EPT also might have a role in partner management for trichomoniasis; however, no partner management intervention has been reported to be more effective than any other in reducing trichomoniasis reinfection rates (*129*,*130*). No data support use of EPT in the routine management of patients with syphilis.

Data are limited regarding use of EPT for gonococcal or chlamydial infections among MSM, compared with heterosexuals (*131*,*132*). Published studies, including recent data regarding extragenital testing, indicated that male partners of MSM with diagnosed gonorrhea or chlamydia might have other bacterial STIs (gonorrhea or syphilis) or HIV (*133*–*135*). Studies have reported that 5% of MSM have a new diagnosis of HIV when evaluated as partners of men with gonococcal or chlamydial infections (*133*,*134*); however, more recent data indicate that, in certain settings, the frequency of HIV infection is much lower (*135*). Considering limited data and potential for other bacterial STIs among MSM partners, shared clinical decision-making regarding EPT is recommended. All persons who receive bacterial STI diagnoses and their sex partners, particularly MSM, should be tested for HIV, and those at risk for HIV infection should be offered HIV PrEP (https://www.cdc.gov/hiv/pdf/risk/prep/cdc-hiv-prep-guidelines-2017.pdf).

### Reporting and Confidentiality

Accurate and timely reporting of STIs is integral to public health efforts in assessing morbidity trends, allocating limited resources, and assisting local health authorities with partner notification and treatment. STI and HIV/AIDS cases should be reported in accordance with state and local statutory requirements. Syphilis (including congenital syphilis), gonorrhea, chlamydia, chancroid, and HIV are reportable diseases in every state. Because the requirements for reporting other STIs differ by state, clinicians should be familiar with the reporting requirements applicable within their jurisdictions.

Reporting can be provider based, laboratory based, or both. Clinicians who are unsure of state and local reporting requirements should seek advice from state or local health department STI programs. STI and HIV reports are kept confidential. In most jurisdictions, such reports are protected by statute or regulation. Before conducting a follow-up of a person with a positive STI test result, public health professionals should consult the patient’s health care provider, if possible, to inform them of the purpose of the public health visit, verify the diagnosis, determine the treatments received, and ascertain the best approaches to patient follow-up.

### Retesting After Treatment to Detect Repeat Infections

Retesting 3 months after diagnosis of chlamydia, gonorrhea, or trichomoniasis can detect repeat infection and potentially can be used to enhance population-based prevention (*136*,*137*). Any person who has a positive test for chlamydia or gonorrhea, along with women who have a positive test for trichomonas, should be rescreened 3 months after treatment. Any person who receives a syphilis diagnosis should undergo follow-up serologic syphilis testing per current recommendations and follow-up testing for HIV (see Syphilis). Additional information regarding retesting is available elsewhere in this report (see Chlamydial Infections; Gonococcal Infections; Syphilis; Trichomoniasis).

## STI Detection Among Special Populations

### Pregnant Women

Intrauterine or perinatally transmitted STIs can have debilitating effects on pregnant women, their fetuses, and their partners. All pregnant women and their sex partners should be asked about STIs, counseled about the possibility of perinatal infections, and provided access to recommended screening and treatment, if needed.

Recommendations for screening pregnant women for STIs to detect asymptomatic infections are based on disease severity and sequelae, prevalence among the population, costs, medicolegal considerations (e.g., state laws), and other factors. The following screening recommendations for pregnant women summarize clinical guidelines from federal agencies and medical professional organizations.

#### Screening Recommendations

##### HIV Infection

 All pregnant women in the United States should be tested for HIV at the first prenatal visit, even if they have been previously tested (*138*). Testing pregnant women for HIV and prompt linkage to care of women with HIV infection are vital for women’s health and reducing perinatal transmission of HIV through ART and obstetrical interventions. HIV testing should be offered as part of the routine panel of prenatal tests (i.e., opt-out testing). For women who decline HIV testing, providers should address their concerns and, when appropriate, continue to encourage testing. Partners of pregnant patients should be offered HIV testing if their status is unknown (*139*).

Retesting in the third trimester (preferably before 36 weeks’ gestation) is recommended for women at high risk for acquiring HIV infection. Examples of women at high risk include those who inject drugs, have STIs during pregnancy, have multiple sex partners during pregnancy, have a new sex partner during pregnancy, or have partners with HIV infection; those who are receiving care in health care facilities in settings with HIV incidence ≥1 per 1,000 women per year; those who are incarcerated; those who live in areas with high rates of HIV infection; or those who have signs or symptoms of acute HIV infection (e.g., fever, lymphadenopathy, skin rash, myalgia, arthralgia, headache, oral ulcers, leukopenia, thrombocytopenia, or transaminase elevation) (*140*).

Rapid HIV testing should be performed for any woman in labor who has not been tested for HIV during pregnancy or whose HIV status is unknown, unless she declines. If a rapid HIV test result is positive, ART should be administered without waiting for the results of confirmatory testing (https://clinicalinfo.hiv.gov/sites/default/files/inline-files/PerinatalGL.pdf).

##### Syphilis

During 2012–2019, congenital syphilis rates in the United States increased from 8.4 to 48.5 cases per 100,000 births, a 477.4% increase (*141*). At least 45 states have a prenatal syphilis testing requirement, with high variability among those requirements (*142*). In the United States, all pregnant women should be screened for syphilis at the first prenatal visit, even if they have been tested previously (*143*). Prenatal screening for syphilis has been reported to be suboptimal in the United States (*144*,*145*). Testing in the third trimester and at delivery can prevent congenital syphilis cases (*146*,*147*). Partners of pregnant women with syphilis should be evaluated, tested, and treated.

When access to prenatal care is not optimal, a stat rapid plasma reagin (RPR) card test and treatment, if that test is reactive, should be administered at the time that a pregnancy is confirmed or when the pregnancy test is performed, if follow-up is uncertain. Pregnant women should be retested for syphilis at 28 weeks’ gestation and at delivery if the mother lives in a community with high syphilis rates or is at risk for syphilis acquisition during pregnancy (e.g., misuses drugs or has an STI during pregnancy, having multiple sex partners, having a new sex partner, or having a sex partner with an STI). Neonates should not be discharged from the hospital unless the syphilis serologic status of the mother has been determined at least once during pregnancy. Any woman who delivers a stillborn infant should be tested for syphilis.

##### Hepatitis B

All pregnant women should be routinely tested for hepatitis B surface antigen (HBsAg) at the first prenatal visit even if they have been previously vaccinated or tested (*148*). Women who are HBsAg positive should be provided with, or referred for, counseling and medical management. Women who are HBsAg negative but at risk for HBV infection should be vaccinated. Women who were not screened prenatally, those who engage in behaviors that put them at high risk for infection (e.g., having had more than one sex partner during the previous 6 months, having been evaluated or treated for an STI, having had recent or current injection drug use, or having an HBsAg-positive sex partner), and those with clinical hepatitis should be tested at the time of admission to the hospital for delivery. To avoid misinterpreting a transient positive HBsAg result during the 21 days after vaccination, HBsAg testing should be performed before vaccine administration. All laboratories that conduct HBsAg tests should test initially reactive specimens with a licensed neutralizing confirmatory test. When pregnant women are tested for HBsAg at the time of admission for delivery, shortened testing protocols can be used, and initially reactive results should prompt expedited administration of immunoprophylaxis to neonates (*148*). Pregnant women who are HBsAg positive should be reported to the local or state health department to ensure that they are entered into a case-management system and that timely and age-appropriate prophylaxis is provided to their infants. Information concerning the pregnant woman’s HBsAg status should be provided to the hospital where delivery is planned and to the health care provider who will care for the newborn. In addition, household and sexual contacts of women who are HBsAg positive should be vaccinated.

##### Chlamydia

All pregnant women aged <25 years as well as older women at increased risk for chlamydia (e.g., those aged ≥25 years who have a new sex partner, more than one sex partner, a sex partner with concurrent partners, or a sex partner who has an STI) should be routinely screened for *Chlamydia trachomatis* at the first prenatal visit (*149*). Pregnant women who remain at increased risk for chlamydial infection also should be retested during the third trimester to prevent maternal postnatal complications and chlamydial infection in the neonate. Pregnant women identified as having chlamydia should be treated immediately and have a test of cure to document chlamydial eradication by a nucleic acid amplification test (NAAT) 4 weeks after treatment. All persons diagnosed with a chlamydial infection should be rescreened 3 months after treatment.

##### Gonorrhea

All pregnant women aged <25 years as well as women aged ≥25 years at increased risk for gonorrhea (e.g., those with other STIs during pregnancy or those with a new sex partner, more than one sex partner, a sex partner with concurrent partners, or a sex partner who has an STI or is exchanging sex for money or drugs) should be screened for *Neisseria gonorrhoeae* at the first prenatal visit (*149*). Pregnant women who remain at high risk for gonococcal infection also should be retested during the third trimester to prevent maternal postnatal complications and gonococcal infection in the neonate. Clinicians should consider the communities they serve and might choose to consult local public health authorities for guidance on identifying groups that are more vulnerable to gonorrhea acquisition on the basis of local disease prevalence. Gonococcal infection, in particular, is concentrated among specific geographic locations and communities (https://www.cdc.gov/std/statistics/2019/default.htm). Pregnant women identified as having gonorrhea should be treated immediately. All persons diagnosed with gonorrhea should be rescreened 3 months after treatment.

##### Hepatitis C Virus

The rate of hepatitis C virus (HCV) infection has increased among pregnant women in recent years (*150*–*153*). HCV screening should be performed for all pregnant women during each pregnancy, except in settings where the HCV infection (HCV positivity) rate is <0.1% (*154*–*156*). The most important risk factor for HCV infection is past or current injecting drug use (*157*). Additional risk factors include having had a blood transfusion or organ transplantation before July 1992, having received clotting factor concentrates produced before 1987, having received an unregulated tattoo, having been on long-term hemodialysis, having other percutaneous exposures, or having HIV infection. All women with HCV infection should receive counseling, supportive care, and linkage to care (https://www.hcvguidelines.org). No vaccine is available for preventing HCV transmission.

##### Cervical Cancer

Pregnant women should undergo cervical cancer screening and at the same frequency as nonpregnant women; however, management differs slightly during pregnancy (*158*). Colposcopy is recommended for the same indications during pregnancy as for nonpregnant women. However, biopsies may be deferred, and endocervical sampling should not be performed. Treatment should not be performed during pregnancy unless cancer is detected.

##### Bacterial Vaginosis, Trichomoniasis, and Genital Herpes

Evidence does not support routine screening for BV among asymptomatic pregnant women at high risk for preterm delivery (*159*). Symptomatic women should be evaluated and treated (see Bacterial Vaginosis). Evidence does not support routine screening for *Trichomonas vaginalis* among asymptomatic pregnant women. Women who report symptoms should be evaluated and treated (see Trichomoniasis). In addition, evidence does not support routine HSV-2 serologic screening among asymptomatic pregnant women. However, type-specific serologic tests might be useful for identifying pregnant women at risk for HSV-2 infection and for guiding counseling regarding the risk for acquiring genital herpes during pregnancy. Routine serial cultures for HSV are not indicated for women in the third trimester who have a history of recurrent genital herpes.

For more detailed discussions of STI screening and treatment among pregnant women, refer to the following references: *Screening for HIV Infection: U.S. Preventive Services Task Force Recommendation Statement* (*138*); *Recommendations for the Use of Antiretroviral Drugs in Pregnant Women with HIV Infection* *and Interventions to Reduce Perinatal HIV Transmission in the United States* (https://clinicalinfo.hiv.gov/sites/default/files/inline-files/PerinatalGL.pdf); *Guidelines for Perinatal Care* (*160*); *Prevention of Hepatitis B Virus Infection in the United States: Recommendations of the Advisory Committee on Immunization Practices* (*12*); *Screening for Chlamydia and Gonorrhea: U.S. Preventive Services Task Force Recommendation Statement* (*149*); *Screening for Bacterial Vaginosis in Pregnant Persons to Prevent Preterm Delivery: U.S. Preventive Service*s *Task Force Recommendation Statement *(*159*); *Screening for Syphilis Infection in Pregnant Women: U.S. Preventive Services Task Force Recommendation Statement *(*161*); *Serologic Screening for Genital Herpes Infection: U.S. Preventive Services Task Force Recommendation Statement* (*162*); *Screening for HIV Infection in Pregnant Women: A Systematic Review for the U.S. Preventive Services Task Force* (*163*); *Screening for Hepatitis B in Pregnant Women: Updated Evidence Report and Systematic Review for the U.S. Preventive Services Task Force* (*164*); and *CDC Recommendations for Hepatitis C Screening Among Adults — United States, 2020 *(*156*).

### Adolescents

In the United States, prevalence rates of certain STIs are highest among adolescents and young adults (*141*). For example, reported rates of chlamydia and gonorrhea are highest among females during their adolescent and young adult years, and many persons acquire HPV infection during that time.

Persons who initiate sex early in adolescence are at higher risk for STIs, as are adolescents living in detention facilities; those receiving services at STD clinics; those who are involved in commercial sex exploitation or survival sex and are exchanging sex for drugs, money, food, or housing; young males who have sex with males (YMSM); transgender youths; and youths with disabilities, substance misuse, or mental health disorders. Factors contributing to increased vulnerability to STIs during adolescence include having multiple sex partners, having sequential sex partnerships of limited duration or concurrent partnerships, failing to use barrier protection consistently and correctly, having lower socioeconomic status, and facing multiple obstacles to accessing health care (*141*,*165*).

All 50 states and the District of Columbia explicitly allow minors to consent for their own STI services. No state requires parental consent for STI care, although the age at which a minor can provide consent for specified health care services (i.e., HPV vaccination and HIV testing and treatment) varies among states. In 2019, a total of 18 states allowed but did not require physicians to notify parents of a minor’s receipt of STI services, including states where minors can legally provide their own consent to the service (https://www.cdc.gov/hiv/policies/law/states/minors.html).

Protecting confidentiality for STI care, particularly for adolescents enrolled in private health insurance plans, presents multiple problems. After a claim has been submitted, many states mandate that health plans provide a written statement to the beneficiary indicating the service performed, the charges covered, what the insurer allows, and the amount for which the patient is responsible (i.e., explanation of benefits [EOB]) (*166*–*169*). In addition, federal laws obligate notices to beneficiaries when claims are denied, including alerting beneficiaries who need to pay for care until the allowable deductible is reached. For STI testing and treatment-related care, an EOB or medical bill that is received by a parent might disclose services provided and list STI laboratory tests performed or treatment administered. Some states have instituted mechanisms for protecting adolescents’ confidentiality and limiting EOBs. Additional risks to confidentiality breaches can inadvertently occur through electronic health records, although technology continues to evolve to assist with ensuring confidential care. AAP and the Society for Adolescent Health and Medicine (SAHM) have published guidance on strategies to address emerging risks for confidentiality breaches associated with health information technology (*169*).

AAP and the SAHM recommend that providers have time alone with their adolescent patients that includes assessment for sexual behavior. The AAP recommendations are available at https://services.aap.org/en/news-room/campaigns-and-toolkits/adolescent-health-care and the SAHM recommendations are available at https://www.adolescenthealth.org/My-SAHM/Login-or-Create-an-Account.aspx?returnurl=%2fResources%2fClinical-Care-Resources%2fConfidentiality.aspx. Discussions concerning sexual behavior should be tailored for the patient’s developmental level and be aimed at identifying risk behaviors (e.g., multiple partners; oral, anal, or vaginal sex; or drug misuse behaviors). Careful, nonjudgmental, and thorough counseling is particularly vital for adolescents who might not feel comfortable acknowledging their engagement in behaviors that make them more vulnerable to acquiring STIs.

#### Screening Recommendations

Recommendations for screening adolescents for STIs to detect asymptomatic infections are based on disease severity and sequelae, prevalence among the population, costs, medicolegal considerations (e.g., state laws), and other factors. Routine laboratory screening for common STIs is indicated for all sexually active adolescents. The following screening recommendations summarize published clinical prevention guidelines for sexually active adolescents from federal agencies and medical professional organizations.

##### Chlamydia

Routine screening for *C. trachomatis* infection on an annual basis is recommended for all sexually active females aged <25 years (*149*). Rectal chlamydial testing can be considered for females on the basis of reported sexual behaviors and exposure, through shared clinical decision-making between the patient and the provider (*170*,*171*). Evidence is insufficient to recommend routine screening for *C. trachomatis* among sexually active young males, on the basis of efficacy and cost-effectiveness. However, screening of sexually active young males should be considered in clinical settings serving populations of young men with a high prevalence of chlamydial infections (e.g., adolescent service clinics, correctional facilities, and STD clinics). Chlamydia screening, including pharyngeal or rectal testing, should be offered to all YMSM at least annually on the basis of sexual behavior and anatomic site of exposure (see Men Who Have Sex with Men).

##### Gonorrhea

Routine screening for *N. gonorrhoeae* on an annual basis is recommended for all sexually active females aged <25 years (*149*). Extragenital gonorrhea screening (pharyngeal or rectal) can be considered for females on the basis of reported sexual behaviors and exposure, through shared clinical-decision between the patient and the provider (*170*,*171*). Gonococcal infection is more prevalent among certain geographic locations and communities (*141*). Clinicians should consider the communities they serve and consult local public health authorities for guidance regarding identifying groups that are more vulnerable to gonorrhea acquisition on the basis of local disease prevalence. Evidence is insufficient to recommend routine screening, on the basis of efficacy and cost-effectiveness, for *N. gonorrhoeae* among asymptomatic sexually active young males who have sex with females only. Screening for gonorrhea, including pharyngeal or rectal testing, should be offered to YMSM at least annually (see Men Who Have Sex with Men).

Providers might consider opt-out chlamydia and gonorrhea screening (i.e., the patient is notified that testing will be performed unless the patient declines, regardless of reported sexual activity) for adolescent and young adult females during clinical encounters. Cost-effectiveness analyses indicate that opt-out chlamydia screening among adolescent and young adult females might substantially increase screening, be cost-saving (*172*), and identify infections among patients who do not disclose sexual behavior (*173*).

##### HIV Infection

HIV screening should be discussed and offered to all adolescents. Frequency of repeat screenings should be based on the patient’s sexual behaviors and the local disease prevalence (*138*). Persons with HIV infection should receive prevention counseling and linkage to care before leaving the testing site.

##### Cervical Cancer

Guidelines from USPSTF and ACOG recommend that cervical cancer screening begin at age 21 years (*174*,*175*). This recommendation is based on the low incidence of cervical cancer and limited usefulness of screening for cervical cancer among adolescents (*176*). In contrast, the 2020 ACS guidelines recommend that cervical cancer screening begin at age 25 years with HPV testing. This change is recommended because the incidence of invasive cervical cancer in women aged <25 years is decreasing because of vaccination (*177*). Adolescents with HIV infection who have initiated sexual intercourse should have cervical screening cytology in accordance with HIV/AIDS guidelines (https://clinicalinfo.hiv.gov/en/guidelines/adult-and-adolescent-opportunistic-infection/human-papillomavirus-disease?view=full).

##### Other Sexually Transmitted Infections

YMSM and pregnant females should be routinely screened for syphilis (see Pregnant Women; Men Who Have Sex with Men). Local disease prevalence can help guide decision-making regarding screening for *T. vaginalis*, especially among adolescent females in certain areas. Routine screening of adolescents and young adults who are asymptomatic for certain STIs (e.g., syphilis, trichomoniasis, BV, HSV, HAV, and HBV) is not typically recommended. 

#### Primary Prevention Recommendations

Primary prevention and anticipatory guidance for recognizing symptoms and behaviors associated with STIs are strategies that should be incorporated into all types of health care visits for adolescents and young adults. The following recommendations for primary prevention of STIs (i.e., vaccination and counseling) are based on published clinical guidelines for sexually active adolescents and young adults from federal agencies and medical professional organizations.

- HPV vaccination is recommended through age 26 years for those not vaccinated previously at the routine age of 11 or 12 years (https://www.cdc.gov/vaccines/hcp/acip-recs/vacc-specific/hpv.html).
- The HBV vaccination series is recommended for all adolescents and young adults who have not previously received the universal HBV vaccine series during childhood (*12*).
- The HAV vaccination series should be offered to adolescents and young adults as well as those who have not previously received the universal HAV vaccine series during childhood (https://www.cdc.gov/vaccines/schedules/hcp/imz/child-indications.html#note-hepa).
- Information regarding HIV transmission, prevention, testing, and implications of infection should be regarded as an essential component of the anticipatory guidance provided to all adolescents and young adults as part of routine health care.
- CDC and USPSTF recommend offering HIV PrEP to adolescents weighing ≥35 kg and adults who are HIV negative and at substantial risk for HIV infection (*80*,*178*). YMSM should be offered PrEP in youth-friendly settings with tailored adherence support (e.g., text messaging and visits per existing guidelines). Indications for PrEP, initial and follow-up prescribing guidance, and laboratory testing recommendations are the same for adolescents and adults (https://www.cdc.gov/hiv/risk/prep).
- Medical providers who care for adolescents and young adults should integrate sexuality education into clinical practice. Health care providers should counsel adolescents about the sexual behaviors that are associated with risk for acquiring STIs and should educate patients regarding evidence-based prevention strategies, which includes a discussion about abstinence and other risk-reduction behaviors (e.g., consistent and correct condom use and reduction in the number of sex partners including concurrent partners). Interactive counseling approaches (e.g., patient-centered counseling and motivational interviewing) are effective STI and HIV prevention strategies and are recommended by USPSTF. Educational materials (e.g., handouts, pamphlets, and videos) can reinforce office-based educational efforts.

### Children

Management of children who have STIs requires close cooperation among clinicians, laboratorians, and child-protection authorities. Official investigations, when indicated, should be initiated promptly. Certain diseases (e.g., gonorrhea, syphilis, HIV, chlamydia, and trichomoniasis), if acquired after the neonatal period, strongly indicate sexual contact. For other diseases (e.g., HSV, HPV and anogenital warts, and vaginitis), the association with sexual contact is not as clear (see Sexual Assault and Abuse and STIs).

### Men Who Have Sex with Men

MSM comprise a diverse group in terms of behaviors, identities, and health care needs (*179*). The term “MSM*”* often is used clinically to refer to sexual behavior alone, regardless of sexual orientation (e.g., a person might identify as heterosexual but still be classified as MSM). Sexual orientation is independent of gender identity. Classification of MSM can vary in the inclusion of transgender men and women on the basis of whether men are defined by sex at birth (i.e., transgender women included) or current gender identity (i.e., transgender men included). Therefore, sexual orientation as well as gender identity of individual persons and their sex partners should be obtained during health care visits. MSM might be at increased risk for HIV and other STIs because of their sexual network or behavioral or biologic factors, including number of concurrent partners, condomless sex, anal sex, or substance use (*180*–*182*). These factors, along with sexual network or higher community disease prevalence, can increase the risk for STIs among MSM compared with other groups (*183*,*184*).

Performing a detailed and comprehensive sexual history is the first step in identifying vulnerability and providing tailored counseling and care (*3*). Factors associated with increased vulnerability to STI acquisition among MSM include having multiple partners, anonymous partners, and concurrent partners (*185*,*186*). Repeat syphilis infections are common and might be associated with HIV infection, substance use (e.g., methamphetamines), Black race, and multiple sex partners (*187*). Similarly, gonorrhea incidence has increased among MSM and might be more likely to display antimicrobial resistance compared with other groups (*188*,*189*). Gonococcal infection among MSM has been associated with similar risk factors to syphilis, including having multiple anonymous partners and substance use, especially methamphetamines (*190*). Disparities in gonococcal infection are also more pronounced among certain racial and ethnic groups of MSM (*141*).

#### HIV Risk Among Men Who Have Sex with Men

MSM are disproportionately at risk for HIV infection. In the United States, the estimated lifetime risk for HIV infection among MSM is one in six, compared with heterosexual men at one in 524 and heterosexual women at one in 253 (*191*). These disparities are further exacerbated by race and ethnicity, with African American/Black and Hispanic/Latino MSM having a one in two and a one in four lifetime risk for HIV infection, respectively. For HIV, transmission occurs much more readily through receptive anal sex, compared with penile-vaginal sex (*192*). Similar to other STIs, multiple partners, anonymous partners, condomless sex, and substance use are all associated with HIV infection (*193*–*196*). Importantly, other STIs also might significantly increase the risk for HIV infection (*197*–*199*). An estimated 10% of new HIV infections were attributable to chlamydial or gonococcal infection (*81*). A substantial number of MSM remain unaware of their HIV diagnosis (*200*). Clinical care involving MSM, including those who have HIV infection, should involve asking about STI-related risk factors and routine STI testing. Clinicians should routinely ask MSM about their sexual behaviors and symptoms consistent with common STIs, including urethral discharge, dysuria, ulcers, rash, lymphadenopathy, and anorectal symptoms that might be consistent with proctitis (e.g., discharge, rectal bleeding, pain on defecation, or pain during anal sex). However, certain STIs are asymptomatic, especially at rectal and pharyngeal sites, and routine testing is recommended. In addition, clinicians should provide education and counseling regarding evidence-based safer-sex approaches that have demonstrated effectiveness in reducing STI incidence (see HIV Infection, Detection, Counseling, and Referral).

#### Pre-Exposure Prophylaxis for HIV Prevention

PrEP is the use of medications for preventing an infection before exposure. Studies have demonstrated that a daily oral medication TDF/FTC is effective in preventing HIV acquisition, and specifically among MSM (*74*,*75*,*201*). PrEP guidelines provide information regarding sexually active persons who are at substantial risk for acquiring HIV infection (having had anal or vaginal sex during the previous 6 months with either a partner with HIV infection, a bacterial STI in the past 6 months, or inconsistent or no condom use with a sex partner) or persons who inject drugs (injecting partner with HIV infection or sharing injection equipment) (*80*). Those guidelines provide information regarding daily PrEP use for either TDF/FTC (men or women) or tenofovir alafenamide and emtricitabine for MSM. Screening for bacterial STIs should occur at least every 6 months for all sexually active patients and every 3 months among MSM or among patients with ongoing risk behaviors. MSM taking PrEP might compensate for decreased HIV acquisition risk by using condoms less frequently or modifying their behavior in other ways (*202*,*203*), although data regarding this behavior are inconsistent. Studies have reported that MSM taking PrEP have high rates of STIs, and frequent screening is warranted (*204*–*206*).

#### Importance of Rectal and Pharyngeal Testing

Rectal and pharyngeal testing by NAAT for gonorrhea and chlamydia is recognized as an important sexual health consideration for MSM. Rectal gonorrhea and chlamydia are associated with HIV infection (*82*,*207*), and men with repeat rectal infections can be at substantially higher risk for HIV acquisition (*208*). Pharyngeal infections with gonorrhea or chlamydia might be a principal source of urethral infections (*209*–*211*). Studies have demonstrated that among MSM, prevalence of rectal gonorrhea and chlamydia ranges from 0.2% to 24% and 2.1% to 23%, respectively, and prevalence of pharyngeal gonorrhea and chlamydia ranges from 0.5% to 16.5% and 0% to 3.6%, respectively (*171*). Approximately 70% of gonococcal and chlamydial infections might be missed if urogenital-only testing is performed among MSM (*212*–*216*) because most pharyngeal and rectal infections are asymptomatic. Self-collected swabs have been reported to be an acceptable means of collection for pharyngeal and rectal specimens (*217*–*219*), which can enhance patient comfort and reduce clinical workloads.

A detailed sexual history should be taken for all MSM to identify anatomic locations exposed to infection for screening. Clinics that provide services for MSM at high risk should consider implementing routine extragenital screening for *N. gonorrhoeae* and *C. trachomatis* infections, and screening is likely to be cost-effective (*220*).

#### Screening Recommendations

STI screening among MSM has been reported to be suboptimal. In a cross-sectional sample of MSM in the United States, approximately one third reported not having had an STI test during the previous 3 years, and MSM with multiple sex partners reported less frequent screening (*221*). MSM living with HIV infection and engaged in care also experience suboptimal rates of STI testing (*222*,*223*). Limited data exist regarding the optimal frequency of screening for gonorrhea, chlamydia, and syphilis among MSM, with the majority of evidence derived from mathematical modeling. Models from Australia have demonstrated that increasing syphilis screening frequency from two times a year to four times a year resulted in a relative decrease of 84% from peak prevalence (*224*). In a compartmental model applied to different populations in Canada, quarterly syphilis screening averted more than twice the number of syphilis cases, compared with semiannual screening (*225*). Furthermore, MSM screening coverage needed for eliminating syphilis among a population is substantially reduced from 62% with annual screening to 23% with quarterly screening (*226*,*227*). In an MSM transmission model that explored the impact of HIV PrEP use on STI prevalence, quarterly chlamydia and gonorrhea screening was associated with an 83% reduction in incidence (*205*). The only empiric data available that examined the impact of screening frequency come from an observational cohort of MSM using HIV PrEP in which quarterly screening identified more bacterial STIs, and semiannual screening would have resulted in delayed treatment of 35% of total identified STI infections (*206*). In addition, quarterly screening was reported to have prevented STI exposure in a median of three sex partners per STI infection (*206*). On the basis of available evidence, quarterly screening for gonorrhea, chlamydia, and syphilis for certain sexually active MSM can improve case finding, which can reduce the duration of infection at the population level, reduce ongoing transmission and, ultimately, prevalence among this population (*228*).

Preventive screening for common STIs is indicated for all MSM. The following screening recommendations summarize published federal agency and USPSTF clinical prevention guidelines for MSM and should be performed at least annually.

##### HIV Infection

HIV serologic testing is indicated if HIV status is unknown or if HIV negative and the patient or their sex partner has had more than one sex partner since the most recent HIV test.

##### Syphilis

Syphilis serologic testing is indicated to establish whether persons with reactive tests have untreated syphilis, have partially treated syphilis, or are manifesting a slow or inadequate serologic response to recommended previous therapy.

##### Gonorrhea and Chlamydia

The following testing is recommended for MSM:

- A test for urethral infection* with *N. gonorrhoeae* and *C. trachomatis* among men who have had insertive intercourse during the preceding year (urine NAAT is preferred).
- A test for rectal infection* with *N. gonorrhoeae* and *C. trachomatis* among men who have had receptive anal intercourse during the preceding year (rectal NAAT is preferred).
- A test for pharyngeal infection* with *N. gonorrhoeae* among men who have had receptive oral intercourse during the preceding year (pharyngeal NAAT is preferred).
- Testing for *C. trachomatis* pharyngeal infection is not recommended.

Basing screening practices solely on history might be suboptimal because providers might feel uncomfortable taking a detailed sexual history (*229*), men might also feel uncomfortable sharing personal sexual information with their provider, and rectal and pharyngeal infections can be identified even in the absence of reported risk behaviors (*171*). Furthermore, the role of saliva, kissing, and rimming (i.e., oral-rectal contact) in the transmission of *N. gonorrhoeae* and *C. trachomatis* has not been well studied (*230*–*232*).

Rectal and pharyngeal testing (provider-collected or self-collected specimens) should be performed for all MSM who report exposure at these sites. Testing can be offered to MSM who do not report exposure at these sites after a detailed explanation, due to known underreporting of risk behaviors. All MSM with HIV infection entering care should be screened for gonorrhea and chlamydia at appropriate anatomic sites of exposure as well as for syphilis.

More frequent STI screening (i.e., for syphilis, gonorrhea, and chlamydia) at 3- to 6-month intervals is indicated for MSM, including those taking PrEP and those with HIV infection, if risk behaviors persist or if they or their sex partners have multiple partners. In addition, providers can consider the benefits of offering more frequent HIV screening (e.g., every 3–6 months) to MSM at increased risk for acquiring HIV infection.

##### Hepatitis B Virus

All MSM should be screened with HBsAg, HBV core antibody, and HBV surface antibody testing to detect HBV infection (*233*). Vaccination against both HAV and HBV is recommended for all MSM for whom previous infection or vaccination cannot be documented. Serologic testing can be considered before vaccinating if the patient’s vaccination history is unknown; however, vaccination should not be delayed. Vaccinating persons who have had previous infection or vaccination does not increase the risk for vaccine-related adverse events (see Hepatitis A Virus; Hepatitis B Virus).

##### Hepatitis C Virus

CDC recommends HCV screening at least once for all adults aged ≥18 years, except in settings where the prevalence of HCV infection (HCV RNA positivity) is <0.1% (*156*). The American Association for the Study of Liver Diseases/Infectious Diseases Society of America guidelines recommend all MSM with HIV infection be screened for HCV during the initial HIV evaluation and at least annually thereafter (https://www.hcvguidelines.org). More frequent screening depends on ongoing risk behaviors, high-risk sexual behavior, and concomitant ulcerative STIs or STI-related proctitis. Sexual transmission of HCV can occur and is most common among MSM with HIV infection (*234*–*237*). Screening for HCV in this setting is cost-effective (*238*,*239*). Screening should be performed by using HCV antibody assays followed by HCV RNA testing for those with a positive antibody test. Suspicion for acute HCV infection (e.g., clinical evidence of hepatitis and risk behaviors) should prompt consideration for HCV RNA testing, despite a negative antibody test.

##### Human Papillomavirus

HPV infection and associated conditions (e.g., anogenital warts and anal squamous intraepithelial lesions) are highly prevalent among MSM. The HPV vaccination is recommended for all men, including MSM and transgender persons or immunocompromised males, including those with HIV infection, through age 26 years (*11*). More information is available at https://www.cdc.gov/hpv/downloads/9vhpv-guidance.pdf.

A digital anorectal examination (DARE) should be performed to detect early anal cancer among persons with HIV and MSM without HIV but who have a history of receptive anal intercourse. Data are insufficient to recommend routine anal cancer screening with anal cytology in populations at risk for anal cancer (see Anal Cancer). Health centers that initiate a cytology-based screening program should only do so if referrals to high-resolution anoscopy (HRA) and biopsy are available.

##### Herpes Simplex Virus-2

Evaluation for HSV-2 infection with type-specific serologic tests also can be considered if infection status is unknown among persons with previously undiagnosed genital tract infection (see Genital Herpes).

#### Postexposure Prophylaxis and Pre-Exposure Prophylaxis for STI Prevention

Studies have reported that a benefit might be derived from STI PEP and PrEP for STI prevention. One study demonstrated that monthly oral administration of a 1-g dose of azithromycin reduced infection with *N. gonorrhoeae* and *C. trachomatis* but did not decrease the incidence of HIV transmission (*240*). Among MSM, doxycycline taken as PEP in a single oral dose ≤24 hours after sex decreased infection with *Treponema pallidum* and *C. trachomatis;* however, no substantial effect was observed for infection with *N. gonorrhoeae* (*93*). Doxycycline taken as STI PrEP as 100 mg orally once daily also demonstrated a substantial reduction in gonorrhea, chlamydia, and syphilis among MSM (*90*). However, these studies had limitations because of small sample size, short duration of therapy, and concerns about antibiotic resistance, specifically regarding *N. gonorrhoeae* (*241*). Further study is needed to determine the effectiveness of using antimicrobials for STI PrEP or PEP.

#### Counseling and Education Approaches

Different counseling and STI prevention strategies are needed to effectively engage different groups of MSM. Outreach efforts should be guided by local surveillance efforts and community input. Engaging MSM at risk through social media, specifically online hookup sites, is an important outreach effort to consider. Hookup sites are Internet sites and mobile telephone applications that men might use for meeting other men for sex. Internet use might facilitate sexual encounters and STI transmission among MSM, and many men report using hookup sites to meet partners (*242*–*245*). The ease and accessibility of meeting partners online might reduce stigma and barriers of meeting partners through other settings. Moreover, these sites offer an opportunity for effective STI prevention messaging (*246*), although the cost might be limiting (*247*). Different groups of MSM might use different hookup sites, and efforts should be guided by local community input. Studies have demonstrated the acceptability and feasibility of reaching MSM through these hookup sites to promote STI prevention efforts (*248*,*249*).

#### Enteric Infections Among Men Who Have Sex with Men

The importance of sexual transmission of enteric pathogens among MSM has been recognized since the 1970s, after the first report of MSM-associated shigellosis was reported in San Francisco (*250*,*251*). Global increases in the incidence of shigellosis among adult MSM have been more recently observed (*252*–*256*). Sporadic outbreaks of *Shigella sonnei* and *Shigella flexneri* have been reported among MSM (*257*–*262*). Transmission occurs through oral-anal contact or sexual contact, and transmission efficiency is enhanced by both biologic or host and behavioral factors. HIV without viral suppression can be an independent risk factor that can contribute to transmission by increasing shedding of the enteric pathogen, increasing susceptibility of the host, or both (*255*,*263*). Surveillance data in England during 2004–2015 demonstrated that 21% of nontravel-associated *Shigella* diagnoses among MSM were among persons with HIV infection (*255*).

Other enteric organisms might also cause disease among MSM through sexual activities leading to oral-anal contact, including bacteria such as *Escherichia coli* (*264*) and *Campylobacter jejuni *or* Campylobacter coli* (*265*,*266*); viruses such as HAV (*267*); and parasites such as *Giardia lamblia* or *Entamoeba histolytic*a (*268*,*269*). Behavioral characteristics associated with the sexual transmission of enteric infections are broadly similar to those associated with other STIs (e.g., gonorrhea, syphilis, and lymphogranuloma venereum [LGV]). This includes multiple sex partners and online hookup sites that increase opportunities for sexual mixing, which might create dense sexual networks that facilitate STI transmission among MSM (*270*). Specific behaviors associated with sexually transmitted enteric infections among MSM involve attendance at sex parties and recreational drug use including chem sex (i.e., using crystal methamphetamine, gamma-butyrolactone, or mephedrone before or during sex), which might facilitate condomless sex, group sex, fisting, use of sex toys, and scat play (*253*,*271*). The growing number of sexually transmitted enteric infections might be attributable in part to the emergence of antimicrobial resistance. This is well reported regarding *Shigella* species, for which rapid intercontinental dissemination of a *S. flexneri* 3a lineage with high-level resistance to azithromycin through sexual transmission among MSM (*272*) and clusters of multidrug resistant shigella cases among MSM have recently been reported (*273*). Multidrug-resistant *Campylobacter* species have also been documented (*266*,*274*). For MSM patients with diarrhea, clinicians should request laboratory examinations, including stool culture; provide counseling about the risk for infection with enteric pathogens during sexual activity (oral-anal, oral-genital, anal-genital, and digital-anal contact) that could expose them to enteric pathogens; and choose treatment, when needed, according to antimicrobial drug susceptibility.

### Women Who Have Sex with Women and Women Who Have Sex with Women and Men

WSW and WSWM comprise diverse groups with variations in sexual identity, practices, and risk behaviors. Studies indicate that certain WSW, particularly adolescents, young women, and WSWM, might be at increased risk for STIs and HIV on the basis of reported risk behaviors (*275*–*280*). Studies have highlighted the diversity of sexual practices and examined use of protective or risk-reduction strategies among WSW populations (*281*–*283*). Use of barrier protection with female partners (e.g., gloves during digital-genital sex, external condoms with sex toys, and latex or plastic barriers [also known as dental dams for oral-genital sex]) was infrequent in all studies. Although health organizations have online materials directed to patients, few comprehensive and reliable resources of sexual health information for WSW are available (*284*).

Recent studies regarding STI rates among WSW and WSWM indicate that WSWM experience higher rates of STIs than WSW, with rates comparable with women who have sex with men (WSM) in all studies reviewed (*279*,*285*,*286*). These studies indicate that WSW might experience STIs at lower rates than WSWM and WSM, although still at significant rates (*287*). One study reported higher sexual-risk behaviors among adolescent WSWM and WSW than among adolescent WSM (*280*). WSW report reduced knowledge of STI risks (*288*), and both WSW and WSWM experience barriers to care, especially Black WSW and WSWM (*289*,*290*). In addition, a continuum of sexual behaviors reported by WSW and WSWM indicates the need for providers to not assume lower risk for WSW, highlighting the importance of an open discussion about sexual health.

Few data are available regarding the risk for STIs conferred by sex between women; however, transmission risk probably varies by the specific STI and sexual practice (e.g., oral-genital sex; vaginal or anal sex using hands, fingers, or penetrative sex items; and oral-anal sex) (*291*,*292*). Practices involving digital-vaginal or digital-anal contact, particularly with shared penetrative sex items, present a possible means for transmission of infected cervicovaginal or anal secretions. This possibility is most directly supported by reports of shared trichomonas infections (*293*,*294*) and by concordant drug-resistance genotype testing and phylogenetic linkage analysis identifying HIV transmitted sexually between women (*295*,*296*). The majority of WSW (53%–97%) have had sex with men in the past and continue to do so, with 5%–28% of WSW reporting male partners during the previous year (*292*,*297*–*300*).

HPV can be transmitted through skin-to-skin contact, and sexual transmission of HPV likely occurs between WSW (*301*–*303*). HPV DNA has been detected through polymerase chain reaction (PCR)–based methods from the cervix, vagina, and vulva among 13%–30% of WSW (*301*,*302*) and can persist on fomites, including sex toys (*304*). Among WSW who report no lifetime history of sex with men, 26% had antibodies to HPV-16, and 42% had antibodies to HPV-6 (*301*). High-grade squamous intraepithelial lesions (HSIL) and low-grade squamous intraepithelial lesions (LSIL) have been detected on Papanicolaou smears (Pap tests) among WSW who reported no previous sex with men (*301*,*302*). WSWM are at risk for acquiring HPV from both their female partners and male partners and thus are at risk for cervical cancer. Therefore, routine cervical cancer screening should be offered to all women, regardless of sexual orientation or practices, and young adult WSW and WSWM should be offered HPV vaccination in accordance with recommendations (*11*) (https://www.cdc.gov/vaccines/hcp/acip-recs/vacc-specific/hpv.html).

Genital transmission of HSV-2 between female sex partners is inefficient but can occur. A U.S. population-based survey among women aged 18–59 years demonstrated an HSV-2 seroprevalence of 30% among women reporting same-sex partners during the previous year, 36% among women reporting same-sex partners in their lifetime, and 24% among women reporting no lifetime same-sex behavior (*299*). HSV-2 seroprevalence among women self-identifying as homosexual or lesbian was 8%, similar to a previous clinic-based study of WSW (*299*,*305*) but was 26% among Black WSW in one study (*287*). The relatively frequent practice of orogenital sex among WSW and WSWM might place them at higher risk for genital infection with HSV-1, a hypothesis supported by the recognized association between HSV-1 seropositivity and previous number of female partners. Thus, sexual transmission of HSV-1 and HSV-2 can occur between female sex partners. This information should be communicated to women as part of sexual health counseling.

Trichomonas is a relatively common infection among WSW and WSWM, with prevalence rates higher than for chlamydia or gonorrhea (*306*,*307*), and direct transmission of trichomonas between female partners has been demonstrated (*293*,*294*).

Limited information is available regarding transmission of bacterial STIs between female partners. Transmission of syphilis between female sex partners, probably through oral sex, has been reported. Although the rate of transmission of *C. trachomatis* or *N. gonorrhoeae* between women is unknown, infection also might be acquired from past or current male partners. Data indicate that *C. trachomatis* infection among WSW can occur (*275*,*286*,*308*,*309*). Data are limited regarding gonorrhea rates among WSW and WSWM (*170*). Reports of same-sex behavior among women should not deter providers from offering and providing screening for STIs, including chlamydia, according to guidelines.

BV is common among women, and even more so among women with female partners (*310*–*312*). Epidemiologic data strongly demonstrate that BV is sexually transmitted among women with female partners. Evidence continues to support the association of such sexual behaviors as having a new partner, having a partner with BV, having receptive oral sex, and having digital-vaginal and digital-anal sex with incident BV (*313*,*314*). A study including monogamous couples demonstrated that female sex partners frequently share identical genital *Lactobacillus* strains (*315*). Within a community-based cohort of WSW, extravaginal (i.e., oral and rectal) reservoirs of BV-associated bacteria were a risk factor for incident BV (*316*). Studies have examined the impact of specific sexual practices on the vaginal microflora (*306*,*317*–*319*) and on recurrent (*320*) or incident (*321*,*322*) BV among WSW. A BV pathogenesis study in WSW reported that *Prevotella bivia, Gardnerella vaginalis*, and *Atopobium vaginae* might have substantial roles in development of incident BV (*323*). These studies have continued to support, although have not proven, the hypothesis that sexual behaviors, specific BV-associated bacteria, and possibly exchange of vaginal or extravaginal microbiota (e.g., oral bacterial communities) between partners might be involved in the pathogenesis of BV among WSW.

Although BV is common among WSW, routine screening for asymptomatic BV is not recommended. Results of one randomized trial used a behavioral intervention to reduce persistent BV among WSW through reduced sharing of vaginal fluid on hands or sex toys. Women randomly assigned to the intervention were 50% less likely to report receptive digital-vaginal contact without gloves than control subjects, and they reported sharing sex toys infrequently. However, these women had no reduction in persistent BV at 1 month posttreatment and no reduction in incident episodes of recurrent BV (*324*). Trials have not been reported examining the benefits of treating female partners of women with BV. Recurrent BV among WSW is associated with having a same-sex partner and a lack of condom use (*325*). Increasing awareness of signs and symptoms of BV among women and encouraging healthy sexual practices (e.g., avoiding shared sex toys, cleaning shared sex toys, and using barriers) might benefit women and their partners.

Sexually active women are at risk for acquiring bacterial, viral, and protozoal STIs from current and previous partners, both male and female. WSW should not be presumed to be at low or no risk for STIs on the basis of their sexual orientation. Report of same-sex behavior among women should not deter providers from considering and performing screening for STIs and cervical cancer according to guidelines. Effective screening requires that care providers and their female patients engage in a comprehensive and open discussion of sexual and behavioral risks that extends beyond sexual identity.

### Transgender and Gender Diverse Persons

Transgender persons often experience high rates of stigma and socioeconomic and structural barriers to care that negatively affect health care usage and increase susceptibility to HIV and STIs (*326*–*332*). Persons who are transgender have a gender identity that differs from the sex that they were assigned at birth (*333*,*334*). Transgender women (also known as trans women, transfeminine persons, or women of transgender experience) are women who were assigned male sex at birth (born with male anatomy). Transgender men (also known as trans men, transmasculine persons, or men of transgender experience) are men who were assigned female sex at birth (i.e., born with female anatomy). In addition, certain persons might identify outside the gender binary of male or female or move back and forth between different gender identities and use such terms as “gender nonbinary,” “genderqueer,” or “gender fluid” to describe themselves. Persons who use terms such as “agender” or “null gender” do not identify with having any gender. The term “cisgender” is used to describe persons who identify with their assigned sex at birth. Prevalence studies of transgender persons among the overall population have been limited and often are based on small convenience samples.

Gender identity is independent of sexual orientation. Sexual orientation identities among transgender persons are diverse. Persons who are transgender or gender diverse might have sex with cisgender men, cisgender women, or other transgender or gender nonbinary persons.

#### Clinical Environment Assessment

Providers should create welcoming environments that facilitate disclosure of gender identity and sexual orientation. Clinics should document gender identity and sex assigned at birth for all patients to improve sexual health care for transgender and gender nonbinary persons. Assessment of gender identity and sex assigned at birth has been validated among diverse populations, has been reported to be acceptable (*335*,*336*), and might result in increased patients identifying as transgender (*337*).

Lack of medical provider knowledge and other barriers to care (e.g., discrimination in health care settings or denial of services) often result in transgender and gender nonbinary persons avoiding or delaying preventive care services (*338*–*340*) and incurring missed opportunities for HIV and STI prevention services. Gender-inclusive and trauma-guided health care might increase the number of transgender patients who seek sexual health services, including STI testing (*341*), because transgender persons are at high risk for sexual violence (*342*).

Primary care providers should take a comprehensive sexual history, including a discussion of STI screening, HIV PrEP and PEP, behavioral health, and social determinants of sexual health. Clinicians can improve the experience of sexual health screening and counseling for transgender persons by asking for their choice of terminology or modifying language (e.g., asking patients their gender pronouns) to be used during clinic visits and history taking and examination (*343*). Options for fertility preservation, pregnancy potential, and contraception options should also be discussed, if indicated. For transgender persons who retain a uterus and ovaries, ovulation might continue in the presence of testosterone therapy, and pregnancy potential exists (https://transcare.ucsf.edu).

#### Transgender Women

A systematic review and meta-analysis of HIV infection among transgender women estimated that HIV prevalence in the United States is 14% among transgender women, with the highest prevalence among Black (44%) and Hispanic (26%) transgender women (*344*). Data also demonstrate high rates of HIV infection among transgender women worldwide (*345*). Bacterial STI prevalence varies among transgender women and is based largely on convenience samples. Despite limited data, international and U.S. studies have indicated elevated incidence and prevalence of gonorrhea and chlamydia among transgender women similar to rates among cisgender MSM (*346*–*348*). A recent study using data from the STD Surveillance Network revealed that the proportions of transgender women with extragenital chlamydial or gonococcal infections were similar to those of cisgender MSM (*349*).

Providers caring for transgender women should have knowledge of their patients’ current anatomy and patterns of sexual behavior before counseling them about STI and HIV prevention. The majority of transgender women have not undergone genital-affirmation surgery and therefore might retain a functional penis; in these instances, they might engage in insertive oral, vaginal, or anal sex as well as receptive oral or anal sex. In the U.S. Transgender Survey, 12% of transgender women had undergone vaginoplasty surgery, and approximately 50% more were considering surgical intervention (*350*). Providers should have knowledge about the type of tissue used to construct the neovagina, which can affect future STI and HIV preventive care and screening recommendations. The majority of vaginoplasty surgeries conducted in the United States use penile and scrotal tissue to create the neovagina (*351*). Other surgical techniques use intestinal tissue (e.g., sigmoid colon graft) or split-skin grafts (*352*). Although these surgeries involve penectomy and orchiectomy, the prostate remains intact. Transgender women who have had a vaginoplasty might engage in receptive vaginal, oral, or anal sex.

Neovaginal STIs have infrequently been reported in the literature and include HSV and HPV/genital warts in penile-inversion vaginoplasty, *C. trachomatis* in procedures that involved penile skin and grafts with urethra mucosa or abdominal peritoneal lining (*353*), and *N. gonorrhoeae* in both penile-inversion and colovaginoplasty (*354*–*359*). If the vaginoplasty used an intestinal graft, a risk also exists for bowel-related disease (e.g., adenocarcinoma, inflammatory bowel disease, diversion colitis, and polyps) (*360*–*362*).

#### Transgender Men

The few studies of HIV prevalence among transgender men indicated that they have a lower prevalence of HIV infection than transgender women. A recent estimate of HIV prevalence among transgender men was 2% (*344*). However, transgender men who have sex with cisgender men might be at elevated risk for HIV infection (*332*,*363*,*364*). Data are limited regarding STI prevalence among transgender men, and the majority of studies have used clinic-based data or convenience sampling. Recent data from the STD Surveillance Network demonstrated higher prevalence of gonorrhea and chlamydia among transgender men, similar to rates reported among cisgender MSM (*365*).

The U.S. Transgender Survey indicated that the proportion of transgender men and gender diverse persons assigned female sex at birth who have undergone gender-affirmation genital surgery is low. Providers should consider the anatomic diversity among transgender men because a person can undergo a metoidioplasty (a procedure to increase the length of the clitoris), with or without urethral lengthening, and might not have a hysterectomy and oophorectomy and therefore be at risk for bacterial STIs, HPV, HSV, HIV, and cervical cancer (*366*). For transgender men using gender-affirming hormone therapy, the decrease in estradiol levels caused by exogenous testosterone can lead to vaginal atrophy (*367*,*368*) and is associated with a high prevalence of unsatisfactory sample acquisition (*369*). The impact of these hormonal changes on mucosal susceptibility to HIV and STIs is unknown.

Transgender men who have not chosen to undergo hysterectomy with removal of the cervix remain at risk for cervical cancer. These persons often avoid cervical cancer screening because of multiple factors, including discomfort with medical examinations and fear of discrimination (*338*,*370*). Providers should be aware that conducting a speculum examination can be technically difficult after metoidioplasty surgery because of narrowing of the introitus. In these situations, high-risk HPV testing using a swab can be considered; self-collected swabs for high-risk HPV testing has been reported to be an acceptable option for transgender men (*371*).

#### Screening Recommendations

The following are screening recommendations for transgender and gender diverse persons:

- Because of the diversity of transgender persons regarding surgical gender-affirming procedures, hormone use, and their patterns of sexual behavior, providers should remain aware of symptoms consistent with common STIs and screen for asymptomatic infections on the basis of the patient’s sexual practices and anatomy.
- Gender-based screening recommendations should be adapted on the basis of anatomy (e.g., routine screening for *C. trachomatis* and *N. gonorrhoeae)* as recommended for all sexually active females aged <25 years on an annual basis and should be extended to transgender men and nonbinary persons with a cervix among this age group.
- HIV screening should be discussed and offered to all transgender persons. Frequency of repeat screenings should be based on level of risk.
- For transgender persons with HIV infection who have sex with cisgender men and transgender women, STI screening should be conducted at least annually, including syphilis serology, HCV testing, and urogenital and extragenital NAAT for gonorrhea and chlamydia.
- Transgender women who have had vaginoplasty surgery should undergo routine STI screening for all exposed sites (e.g., oral, anal, or vaginal). No data are available regarding the optimal screening method (urine or vaginal swab) for bacterial STIs of the neovagina. The usual techniques for creating a neovagina do not result in a cervix; therefore, no rationale exists for cervical cancer screening (*368*).
- If transgender men have undergone metoidioplasty surgery with urethral lengthening and have not had a vaginectomy, assessment of genital bacterial STIs should include a cervical swab because a urine specimen will be inadequate for detecting cervical infections.
- Cervical cancer screening for transgender men and nonbinary persons with a cervix should follow current screening guidelines (see Human Papillomavirus Infections).

### Persons in Correctional Facilities

Multiple studies have demonstrated that persons entering correctional facilities have a high prevalence of STIs, HIV, and viral hepatitis, especially those aged ≤35 years (*141*,*372*,*373*). Risk behaviors for acquiring STIs (e.g., having condomless sex, having multiple sex partners, substance misuse, and engaging in commercial, survival, or coerced sex) are common among incarcerated populations. Before their incarceration, many persons have had limited access to medical care. Other social determinants of health (e.g., insufficient social and economic support or living in communities with high local STI prevalence) are common. Addressing STIs in correctional settings is vital for addressing the overall STI impact among affected populations.

Growing evidence demonstrates the usefulness of expanded STI screening and treatment services in correctional settings, including short-term facilities (jails), long-term institutions (prisons), and juvenile detention centers. For example, in jurisdictions with comprehensive, targeted jail screening, more chlamydial infections among females (and males if screened) are detected and subsequently treated in the correctional setting than in any other single reporting source (*141*,*374*) and might represent the majority of reported cases in certain jurisdictions (*375*). Screening in the jail setting has the potential to reach substantially more persons at risk than screening among the prison population alone.

Both males and females aged ≤35 years in juvenile and adult detention facilities have been reported to have higher rates of chlamydia and gonorrhea than nonincarcerated persons in the community (*141*,*374*,*376*). Syphilis seroprevalence rates, which can indicate previously treated or current infection, are considerably higher among incarcerated adult men and women than among adolescents, which is consistent with the overall national syphilis trends (*141*,*374*). Detection and treatment of early syphilis in correctional facilities might affect rates of transmission among adults and prevention of congenital syphilis (*377*).

In jails, approximately half of entrants are released back into the community within 48 hours. As a result, treatment completion rates for those screened for STIs and who receive STI diagnoses in short-term facilities might not be optimal. However, because of the mobility of incarcerated populations in and out of the community, the impact of screening in correctional facilities on the prevalence of infections among detainees and subsequent transmission in the community after release might be considerable (*378*). Moreover, treatment completion rates of ≥95% in short-term facilities can be achieved by offering screening at or shortly after intake, thus facilitating earlier receipt of test results and, if needed, follow-up of untreated persons can be conducted through public health outreach.

Universal, opt-out screening for chlamydia and gonorrhea among females aged ≤35 years entering juvenile and adult correctional facilities is recommended (*379*). Males aged <30 years entering juvenile and adult correctional facilities should also be screened for chlamydia and gonorrhea (*380*). Opt-out screening has the potential to substantially increase the number tested and the number of chlamydia and gonorrhea infections detected (*381*–*385*). Point-of-care (POC) NAAT might also be considered if the tests have demonstrated sufficient sensitivity and specificity. Studies have demonstrated high prevalence of trichomoniasis among incarcerated females (*386*–*392*).

#### Screening Recommendations

##### Chlamydia and Gonorrhea

Females aged ≤35 years and males aged <30 years housed in correctional facilities should be screened for chlamydia and gonorrhea. This screening should be conducted at intake and offered as opt-out screening.

##### Trichomonas

Females aged ≤35 years housed in correctional facilities should be screened for trichomonas. This screening should be conducted at intake and offered as opt-out screening.

##### Syphilis

Opt-out screening for incarcerated persons should be conducted on the basis of the local area and institutional prevalence of early (primary, secondary, or early latent) infectious syphilis. Correctional facilities should stay apprised of local syphilis prevalence. In short-term facilities, screening at entry might be indicated.

##### Viral Hepatitis

All persons housed in juvenile and adult correctional facilities should be screened at entry for viral hepatitis, including HAV, HBV, and HCV, depending on local prevalence and the person’s vaccination status. Vaccination for HAV and HBV should be offered if the person is susceptible.

##### Cervical Cancer

Women and transgender men who are housed in correctional facilities should be screened for cervical cancer as for women who are not incarcerated (*393*,*394*) (see Cervical Cancer).

##### HIV Infection

All persons being housed in juvenile and adult correctional facilities should be screened at entry for HIV infection; screening should be offered as opt-out screening. For those identified as being at risk for HIV infection (e.g., with diagnosed gonorrhea or syphilis or persons who inject drugs) and being released into the community, starting HIV PrEP (or providing linkage to a community clinic for HIV PrEP) for HIV prevention should be considered (*395*,*396*). Persons are likely to engage in high-risk activities immediately after release from incarceration (*397*). For those identified with HIV infection, treatment should be initiated. Those persons receiving PrEP or HIV treatment should have linkage to care established before release. Correctional settings should consider implementing other STI prevention approaches, both during incarceration and upon release, which might include educational and behavioral counseling interventions (*398*–*401*), vaccination (e.g., for HPV) (*402*,*403*), condom distribution (*404*,*405*), EPT (*125*), and PrEP to prevent HIV infection (see Primary Prevention Methods).

## HIV Infection

### Detection, Counseling, and Referral

Infection with HIV causes an acute but brief and nonspecific influenza-like retroviral syndrome that can include fever, malaise, lymphadenopathy, pharyngitis, arthritis, or skin rash. Most persons experience at least one symptom; however, some might be asymptomatic or have no recognition of illness (*406*–*409*). Acute infection transitions to a multiyear, chronic illness that progressively depletes CD4^+^ T lymphocytes crucial for maintenance of effective immune function. Ultimately, persons with untreated HIV infection experience symptomatic, life-threatening immunodeficiency (i.e., AIDS).

Effective ART that suppresses HIV replication to undetectable levels reduces morbidity, provides a near-normal lifespan, and prevents sexual transmission of HIV to others (*95*–*97*,*410*–*412*). Early diagnosis of HIV and rapid linkage to care are essential for achieving these goals. Guidelines from both the U.S. Department of Health and Human Services and the International AIDS Society–USA Panel recommend that all persons with HIV infection be offered effective ART as soon as possible, both to reduce morbidity and mortality and to prevent HIV transmission (*413*).

STD specialty or sexual health clinics are a vital partner in reducing HIV infections in the United States. These clinics provide safety net services to vulnerable populations in need of HIV prevention services who are not served by the health care system and HIV partner service organizations. Diagnosis of an STI is a biomarker for HIV acquisition, especially among persons with primary or secondary syphilis or, among MSM, rectal gonorrhea or chlamydia (*197*). STD clinics perform only approximately 20% of all federally funded HIV tests nationally but identify approximately 30% of all new infections (*414*). Among testing venues, STD clinics are high performing in terms of linkage to HIV care within 90 days of diagnosis; during 2013–2017, the percentage of persons with a new diagnosis in an STD clinic and linked to care within 90 days increased from 55% to >90% (*415*,*415*).

#### Screening Recommendations

The following recommendations apply to testing for HIV:

- HIV testing is recommended for all persons seeking STI evaluation who are not already known to have HIV infection. Testing should be routine at the time of the STI evaluation, regardless of whether the patient reports any specific behavioral risks for HIV. Testing for HIV should be performed at the time of STI diagnosis and treatment if not performed at the initial STI evaluation and screening (*82*,*195*,*416*).
- CDC and USPSTF recommend HIV screening at least once for all persons aged 15–65 years (*417*).
- Persons at higher risk for HIV acquisition, including sexually active gay, bisexual, and other MSM, should be screened for HIV at least annually. Providers can consider the benefits of offering more frequent screening (e.g., every 3–6 months) among MSM at increased risk for acquiring HIV (*418*,*419*).
- All pregnant women should be tested for HIV during the first prenatal visit. A second test during the third trimester, preferably at <36 weeks’ gestation, should be considered and is recommended for women who are at high risk for acquiring HIV infection, women who receive health care in jurisdictions with high rates of HIV, and women examined in clinical settings in which HIV incidence is ≥1 per 1,000 women screened per year (*138*,*140*).
- HIV screening should be voluntary and free from coercion. Patients should not be tested without their knowledge.
- Opt-out HIV screening (notifying the patient that an HIV test will be performed, unless the patient declines) is recommended in all health care settings. CDC also recommends that consent for HIV screening be incorporated into the general informed consent for medical care in the same manner as other screening or diagnostic tests.
- Requirement of specific signed consent for HIV testing is not recommended. General informed consent for medical care is considered sufficient to encompass informed consent for HIV testing.
- Providers should use a laboratory-based antigen/antibody (Ag/Ab) combination assay as the first test for HIV, unless persons are unlikely to follow up with a provider to receive their HIV test results; in those cases screening with a rapid POC test can be useful.
- Preliminary positive screening tests for HIV should be followed by supplemental testing to establish the diagnosis.
- Providing prevention counseling as part of HIV screening programs or in conjunction with HIV diagnostic testing is not required (*6*). However, persons might be more likely to think about HIV and consider their risk-related behavior when undergoing an HIV test. HIV testing gives providers an opportunity to conduct STI and HIV prevention counseling and communicate risk-reduction messages.
- Acute HIV infection can occur among persons who report recent sexual or needle-sharing behavior or who have had an STI diagnosis.
- Providers should test for HIV RNA if initial testing according to the HIV testing algorithm recommended by CDC is negative or indeterminate when concerned about acute HIV infection (https://stacks.cdc.gov/view/cdc/50872).
- Providers should not assume that a laboratory report of a negative HIV Ag/Ab or antibody test indicates that the requisite HIV RNA testing for acute HIV infection has been conducted. They should consider explicitly requesting HIV RNA testing when concerned about early acute HIV infection.
- Providers should assess eligibility of all persons seeking STI services for HIV PrEP and PEP. For persons with substantial risk whose results are HIV negative, providers should offer or provide referral for PrEP services, unless the last potential HIV exposure occurred <72 hours, in which case PEP might be indicated.

#### Diagnostic Considerations

HIV infection can be diagnosed by HIV 1/2 Ag/Ab combination immunoassays. All FDA-cleared HIV tests are highly sensitive and specific. Available serologic tests can detect all known subtypes of HIV-1. The majority also detect HIV-2 and uncommon variants of HIV-1 (e.g., group O and group N).

According to an algorithm for HIV diagnosis, CDC recommends that HIV testing begin with a laboratory-based HIV-1/HIV-2 Ag/Ab combination assay, which, if repeatedly reactive, is followed by a laboratory-based assay with a supplemental HIV-1/HIV-2 antibody differentiation assay (https://stacks.cdc.gov/view/cdc/50872). This algorithm confers an additional advantage because it can detect HIV-2 antibodies after the initial immunoassay. Although HIV-2 is uncommon in the United States, accurate identification is vital because monitoring and therapy for HIV-2 differs from that for HIV-1 (*420*). RNA testing should be performed on all specimens with reactive immunoassay but negative supplemental antibody test results to determine whether the discordance represents acute HIV infection.

Rapid POC HIV tests can enable clinicians to make a preliminary diagnosis of HIV infection in <20 minutes. The majority of rapid antibody assays become reactive later in the course of HIV infection than conventional laboratory-based assays and thus can produce negative results among persons recently infected (e.g., acutely infected persons). Furthermore, HIV home-test kits only detect HIV antibodies and therefore will not detect acute HIV infection. If early or acute infection is suspected and a rapid HIV antibody assay is negative, confirmatory testing with combined laboratory-based assays or RNA testing should be performed. CDC recommends that all persons with reactive rapid tests be assessed with a laboratory-based Ag/Ab assay. Additional details about interpretation of results by using the HIV testing algorithm recommended by CDC are available at https://stacks.cdc.gov/view/cdc/48472.

#### Acute HIV Infection

Providers serving persons at risk for STIs are in a position to diagnose HIV infection during its acute phase. Diagnosing HIV infection during the acute phase is particularly important because persons with acute HIV have highly infectious disease due to the concentration of virus in plasma and genital secretions, which is extremely elevated during that stage of infection (*421*,*422*) (https://clinicalinfo.hiv.gov/en/guidelines/adult-and-adolescent-arv/acute-and-recent-early-hiv-infection?view=full). ART during acute HIV infection is recommended because it substantially reduces infection transmission to others, improves laboratory markers of disease, might decrease severity of acute disease, lowers viral setpoint, reduces the size of the viral reservoir, decreases the rate of viral mutation by suppressing replication, and preserves immune function (https://clinicalinfo.hiv.gov/en/guidelines/adult-and-adolescent-arv/acute-and-recent-early-hiv-infection?view=full). Persons who receive an acute HIV diagnosis should be referred immediately to an HIV clinical care provider, provided prevention counseling (e.g., advised to reduce the number of partners and to use condoms correctly and consistently), and screened for STIs. Information should be provided regarding availability of PEP for sexual and injecting drug use partners not known to have HIV infection if the most recent contact was <72 hours preceding HIV diagnosis.

When providers test by using the CDC algorithm, specimens collected during acute infection might give indeterminate or negative results because insufficient anti-HIV antibodies and potentially insufficient antigen are present to be reactive on Ag/Ab combination assays and supplemental HIV-1/HIV-2 antibody differentiation assays. Whenever acute HIV infection is suspected (e.g., initial testing according to the CDC algorithm is negative or indeterminate after a possible sexual exposure to HIV within the previous few days to weeks, especially if the person has symptoms or has primary or secondary syphilis, gonorrhea, or chlamydia), additional testing for HIV RNA is recommended. If this additional testing for HIV RNA is also negative, repeat testing in a few weeks is recommended to rule out very early acute infection when HIV RNA might not be detectable. A more detailed discussion of testing in the context of acute HIV infection is available at https://clinicalinfo.hiv.gov/en/guidelines/adult-and-adolescent-arv/initiation-antiretroviral-therapy?view=full.

#### Treatment

ART should be initiated as soon as possible for all persons with HIV infection regardless of CD4^+^ T-cell count, both for individual health and to prevent HIV transmission (https://clinicalinfo.hiv.gov/sites/default/files/inline-files/AdultandAdolescentGL.pdf). Persons with HIV infection who achieve and maintain a viral load suppressed to <200 copies/mL with ART have effectively no risk for sexually transmitting HIV (*95*–*97*,*421*). Early HIV diagnosis and treatment is thus not only vital for individual health but also as a public health intervention to prevent new infections. Knowledge of the prevention benefit of treatment can help reduce stigma and increase the person’s commitment to start and remain adherent to ART (*423*). The importance of adherence should be stressed as well as the fact that ART does not protect against other STIs that can be prevented by using condoms. Interventions to assist persons to remain adherent to their prescribed HIV treatment, to otherwise reduce the possibility of transmission to others, and to protect themselves against STIs, have been developed for diverse populations at risk (*424*) (https://clinicalinfo.hiv.gov/sites/default/files/inline-files/AdultandAdolescentGL.pdf).

Comprehensive HIV treatment and care services might not be available in facilities focused primarily on STI treatment. Providers in such settings should be knowledgeable about HIV treatment and care options available in their communities and promptly link persons who have newly diagnosed HIV infection and any persons with HIV infection who are not engaged in ongoing effective care to a health care provider or facility experienced in caring for persons living with HIV (https://clinicalinfo.hiv.gov/sites/default/files/inline-files/AdultandAdolescentGL.pdf).

#### Other HIV Management Considerations

Behavioral and psychosocial services are integral to caring for persons with HIV infection. Providers should expect persons to be distressed when first informed that they have HIV. They face multiple adaptive challenges, including coping with the reactions of others to a stigmatizing illness, developing and adopting strategies to maintain physical and emotional health, initiating changes in behavior to prevent HIV transmission to others, and reducing the risk for acquiring additional STIs. Many persons will require assistance gaining access to health care and other support services and coping with changes in personal relationships.

Persons with HIV infection might have additional needs (e.g., referral for substance use or mental health disorders). Others require assistance to secure and maintain employment and housing. Persons capable of reproduction might require family planning counseling, information about reproductive health choices, and referral for reproductive health care.

The following recommendations apply to managing persons with diagnosed HIV infection:

- Link persons with HIV infection to care and start them on ART as soon as possible.
- Report cases (in accordance with local requirements) to public health and initiate partner services.
- Provide prevention counseling to persons with diagnosed HIV infection.
- Ensure all persons with HIV infection are informed that if they achieve and maintain a suppressed viral load, they have effectively no risk for transmitting HIV. Stress that a suppressed viral load is not a substitute for condoms and behavioral modifications because ART does not protect persons with HIV against other STIs.
- Provide additional counseling, either on-site or through referral, about the psychosocial and medical implications of having HIV infection.
- Assess the need for immediate medical care and psychosocial support.
- Link persons with diagnosed HIV infection to services provided by health care personnel experienced in managing HIV infection. Additional services that might be needed include substance misuse counseling and treatment, treatment for mental health disorders or emotional distress, reproductive counseling, risk-reduction counseling, and case management. Providers should follow up to ensure that patients have received services for any identified needs.
- Persons with HIV infection should be educated about the importance of ongoing medical care and what to expect from these services.

#### STI Screening of Persons with HIV Infection in HIV Care Settings

At the initial HIV care visit, providers should screen all sexually active persons for syphilis, gonorrhea, and chlamydia, and perform screening for these infections at least annually during the course of HIV care (*425*). Specific testing includes syphilis serology and NAAT for *N. gonorrhoeae* and *C. trachomatis* at the anatomic site of exposure. Women should also be screened for trichomoniasis at the initial visit and annually thereafter. Women should be screened for cervical cancer precursor lesions per existing guidelines (*98*).

More frequent screening for syphilis, gonorrhea, and chlamydia (e.g., every 3 or 6 months) should be tailored to individual risk and the local prevalence of specific STIs. Certain STIs can be asymptomatic; their diagnosis might prompt referral for partner services, might identify sexual and needle-sharing partners who can benefit from early diagnosis and treatment of HIV, and might prompt reengagement in care or HIV prevention services (e.g., PEP or PrEP) (*8*). More detailed information on screening, testing, and treatment is provided in pathogen-specific sections of this report.

### Partner Services and Reporting

Partner notification is a key component in the evaluation of persons with HIV infection. Early diagnosis and treatment of HIV among all potentially exposed sexual and injecting drug sharing partners can improve their health and reduce new infections. For those partners without HIV infection, partner services also provide an opportunity for offering HIV prevention services, including PrEP or PEP (if exposure was <72 hours previous) and STI testing and treatment.

Health care providers should inform persons with diagnosed HIV infection about any legal obligations of providers to report cases of HIV to public health; the local confidential processes for managing partner services, including that a public health department still might be in contact to follow up in their care and partner services; and the benefits and risks of partner notification and services. Health care providers should also encourage persons with a new HIV diagnosis to notify their partners and provide them with referral information for their partners about HIV testing. Partner notification for exposure to HIV should be confidential. Health care providers can assist in the partner notification process, either directly or by referral to health department partner notification programs. Health department staff are trained to use public health investigation strategies for confidentially locating persons who can benefit from HIV treatment, care, or prevention services. Guidance regarding spousal notification varies by jurisdiction. Detailed recommendations for notification, evaluation, and treatment of exposed partners are available in *Recommendations for Partner Services Programs for HIV Infection, Syphilis, Gonorrhea, and Chlamydial Infections* (*111*).

#### Special Considerations

##### Pregnancy

All pregnant women should be tested for HIV during the first prenatal visit. A second test during the third trimester, preferably at <36 weeks’ gestation, should be considered and is recommended for women who are at high risk for acquiring HIV, women who receive health care in jurisdictions with high rates of HIV infection, and women served in clinical settings in which prenatal screening identifies ≥1 pregnant woman with HIV per 1,000 women screened (*138*). Diagnostic algorithms for HIV for pregnant women do not differ from those for nonpregnant women (see STI Detection Among Special Populations). Pregnant women should be informed that HIV testing will be performed as part of the routine panel of prenatal tests (*138*); for women who decline HIV testing, providers should address concerns that pose obstacles, discuss the benefits of testing (e.g., early HIV detection, treatment, and care for improving health of the mother and reducing perinatal transmission of HIV), and encourage testing at subsequent prenatal visits. Women who decline testing because they have had a previous negative HIV test result should be informed about the importance of retesting during each pregnancy. Women with no prenatal care should be tested for HIV at the time of delivery.

Testing pregnant women is crucial because knowledge of infection status can help maintain the woman’s health, and it enables receipt of interventions (i.e., ART or specialized obstetrical care) that can substantially reduce the risk for perinatal transmission of HIV. Pregnant women with diagnosed HIV infection should be educated about the benefits of ART for their own health and for reducing the risk for HIV transmission to their infant. In the absence of ART, a mother’s risk for transmitting HIV to her neonate is approximately 30%; however, risk can be reduced to <2% through ART, obstetrical interventions (i.e., elective cesarean delivery at 38 weeks’ pregnancy), and breastfeeding avoidance (https://clinicalinfo.hiv.gov/sites/default/files/inline-files/PerinatalGL.pdf). Pregnant women with HIV infection should be linked to an HIV care provider experienced in managing HIV in pregnancy and provided antenatal and postpartum treatment and advice. Detailed and regularly updated recommendations for managing pregnant patients with HIV infection are available at https://clinicalinfo.hiv.gov/sites/default/files/inline-files/PerinatalGL.pdf.

##### HIV Infection Among Neonates, Infants, and Children

Diagnosis of HIV infection in a pregnant woman indicates the need for evaluating and managing the HIV-exposed neonate and considering whether the woman’s other children, if any, might be infected. Detailed recommendations regarding diagnosis and management of HIV infection among neonates and children of mothers with HIV are beyond the scope of these guidelines but are available at https://clinicalinfo.hiv.gov/en/guidelines. Exposed neonates and children with HIV infection should be referred to physicians with expertise in neonatal and pediatric HIV management.

## Diseases Characterized by Genital, Anal, or Perianal Ulcers

In the United States, the majority of young, sexually active patients who have genital, anal, or perianal ulcers have either genital herpes or syphilis. The frequency of each condition differs by geographic area and population; however, genital herpes is the most prevalent of these diseases. More than one etiologic agent (e.g., herpes and syphilis) can be present in any genital, anal, or perianal ulcer. Less common infectious causes of genital, anal, or perianal ulcers include chancroid, LGV, and granuloma inguinale (donovanosis). GUDs (e.g., syphilis, herpes, and LGV) might also present as oral ulcers. Genital herpes, syphilis, chlamydia, gonorrhea, and chancroid have been associated with an increased risk for HIV acquisition and transmission. Genital, anal, or perianal lesions can also be associated with infectious and noninfectious conditions that are not sexually transmitted (e.g., yeast, trauma, carcinoma, aphthae or Behcet’s disease, fixed drug eruption, or psoriasis).

A diagnosis based only on medical history and physical examination frequently can be inaccurate. Therefore, all persons who have genital, anal, or perianal ulcers should be evaluated. Specific evaluation of genital, anal, or perianal ulcers includes syphilis serology tests and darkfield examination from lesion exudate or tissue, or NAAT if available; NAAT or culture for genital herpes type 1 or 2; and serologic testing for type-specific HSV antibody. In settings where chancroid is prevalent, a NAAT or culture for *Haemophilus ducreyi* should be performed.

No FDA-cleared NAAT for diagnosing syphilis is available in the United States; however, multiple FDA-cleared NAATs are available for diagnosing HSV-1 and HSV-2 in genital specimens. Certain clinical laboratories have developed their own syphilis and HSV NAATs and have conducted Clinical Laboratory Improvement Amendment (CLIA) verification studies with genital specimens. Type-specific serology for HSV-2 might aid in identifying persons with genital herpes (see Genital Herpes). In addition, biopsy of ulcers with immunohistochemistry can help identify the cause of ulcers that are unusual or that do not respond to initial therapy. HIV testing should be performed on all persons not known to have HIV infection who present with genital, anal, or perianal ulcers (see Diagnostic Considerations in disease-specific sections). NAAT testing at extragenital sites should be considered for cases in which GUDs are suspected (e.g., oral manifestations of syphilis, herpes, or LGV). Commercially available NAATs have not been cleared by FDA for these indications; however, they can be used by laboratories that have met regulatory requirements for an off-label procedure.

Because early syphilis treatment decreases transmission possibility, public health standards require health care providers to presumptively treat any patient with a suspected case of infectious syphilis at the initial visit, even before test results are available. Presumptive treatment of a patient with a suspected first episode of genital herpes also is recommended because HSV treatment benefits depend on prompt therapy initiation. The clinician should choose the presumptive treatment on the basis of the clinical presentation (i.e., HSV lesions begin as vesicles and primary syphilis as a papule) and epidemiologic circumstances (e.g., high incidence of disease among populations and communities and travel history). For example, syphilis is so common among MSM that any male who has sex with men presenting with a genital ulcer should be presumptively treated for syphilis at the initial visit after syphilis and HSV tests are performed. After a complete diagnostic evaluation, >25% of patients who have genital ulcers might not have a laboratory-confirmed diagnosis (*426*).

### Chancroid

Chancroid prevalence has declined in the United States (*141*). When infection does occur, it is usually associated with sporadic outbreaks. Worldwide, chancroid appears to have decreased as well, although infection might still occur in certain Africa regions and the Caribbean. Chancroid is a risk factor in HIV transmission and acquisition (*197*).

#### Diagnostic Considerations

A definitive diagnosis of chancroid requires identifying *H. ducreyi* on special culture media that is not widely available from commercial sources; even when these media are used, sensitivity is <80% (*427*). No FDA-cleared NAAT for *H. ducreyi* is available in the United States; however, such testing can be performed by clinical laboratories that have developed their own NAAT and have conducted CLIA verification studies on genital specimens.

The combination of one or more deep and painful genital ulcers and tender suppurative inguinal adenopathy indicates the chancroid diagnosis; inguinal lymphadenitis typically occurs in <50% of cases (*428*). For both clinical and surveillance purposes, a probable diagnosis of chancroid can be made if all of the following four criteria are met: 1) the patient has one or more painful genital ulcers; 2) the clinical presentation, appearance of genital ulcers and, if present, regional lymphadenopathy are typical for chancroid; 3) the patient has no evidence of *T. pallidum* infection by darkfield examination or NAAT (i.e., ulcer exudate or serous fluid) or by serologic tests for syphilis performed at least 7–14 days after onset of ulcers; and 4) HSV-1 or HSV-2 NAAT or HSV culture performed on the ulcer exudate or fluid are negative.

#### Treatment

Successful antimicrobial treatment for chancroid cures the infection, resolves the clinical symptoms, and prevents transmission to others. In advanced cases, genital scarring and rectal or urogenital fistulas from suppurative buboes can result despite successful therapy.

**Recommended Regimens for Chancroid**
**Azithromycin **1 g orally in a single dose
*or*
**Ceftriaxone** 250 mg IM in a single dose
*or*
**Ciprofloxacin** 500 mg orally 2 times/day for 3 days
*or*
**Erythromycin base** 500 mg orally 3 times/day for 7 days

Azithromycin and ceftriaxone offer the advantage of single-dose therapy (*429*). Worldwide, several isolates with intermediate resistance to either ciprofloxacin or erythromycin have been reported. However, because cultures are not routinely performed, and chancroid is uncommon, data are limited regarding prevalence of *H. ducreyi* antimicrobial resistance.

#### Other Management Considerations

Men who are uncircumcised and persons with HIV infection do not respond as well to treatment as persons who are circumcised or are HIV negative (*430*). Patients should be tested for HIV at the time chancroid is diagnosed. If the initial HIV test results were negative, the provider can consider the benefits of offering more frequent testing and HIV PrEP to persons at increased risk for HIV infection.

#### Follow-Up

Patients should be reexamined 3–7 days after therapy initiation. If treatment is successful, ulcers usually improve symptomatically within 3 days and objectively within 7 days after therapy. If no clinical improvement is evident, the clinician should consider whether the diagnosis is correct, another STI is present, the patient has HIV infection, the treatment was not used as instructed, or the *H. ducreyi* strain causing the infection is resistant to the prescribed antimicrobial. The time required for complete healing depends on the size of the ulcer; large ulcers might require >2 weeks. In addition, healing can be slower for uncircumcised men who have ulcers under the foreskin. Clinical resolution of fluctuant lymphadenopathy is slower than that of ulcers and might require needle aspiration or incision and drainage, despite otherwise successful therapy. Although needle aspiration of buboes is a simpler procedure, incision and drainage might be preferred because of reduced need for subsequent drainage procedures.

#### Management of Sex Partners

Regardless of whether disease symptoms are present, sex partners of patients with chancroid should be examined and treated if they had sexual contact with the patient during the 10 days preceding the patient’s symptom onset.

#### Special Considerations

##### Pregnancy

Data indicate ciprofloxacin presents a low risk to the fetus during pregnancy but has potential for toxicity during breastfeeding (*431*). Alternative drugs should be used if the patient is pregnant or lactating. No adverse effects of chancroid on pregnancy outcome have been reported.

##### HIV Infection

Persons with HIV infection who have chancroid infection should be monitored closely because they are more likely to experience chancroid treatment failure and to have ulcers that heal slowly (*430*,*432*). Persons with HIV might require repeated or longer courses of therapy, and treatment failures can occur with any regimen. Data are limited concerning the therapeutic efficacy of the recommended single-dose azithromycin and ceftriaxone regimens among persons with HIV infection.

##### Children

Because sexual contact is the major primary transmission route among U.S. patients, diagnosis of chancroid ulcers among infants and children, especially in the genital or perineal region, is highly suspicious of sexual abuse. However, *H. ducreyi* is recognized as a major cause of nonsexually transmitted cutaneous ulcers among children in tropical regions and, specifically, countries where yaws is endemic (*433*–*435*). Acquisition of a lower-extremity ulcer attributable to *H. ducreyi* in a child without genital ulcers and reported travel to a region where yaws is endemic should not be considered evidence of sexual abuse.

### Genital Herpes 

Genital herpes is a chronic, lifelong viral infection. Two types of HSV can cause genital herpes: HSV-1 and HSV-2. Most cases of recurrent genital herpes are caused by HSV-2, and 11.9% of persons aged 14–49 years are estimated to be infected in the United States (*436*). However, an increasing proportion of anogenital herpetic infections have been attributed to HSV-1, which is especially prominent among young women and MSM (*186*,*437*,*438*).

The majority of persons infected with HSV-2 have not had the condition diagnosed, many of whom have mild or unrecognized infections but shed virus intermittently in the anogenital area. Consequently, most genital herpes infections are transmitted by persons unaware that they have the infection or who are asymptomatic when transmission occurs. Management of genital HSV should address the chronic nature of the infection rather than focusing solely on treating acute episodes of genital lesions.

#### Diagnostic Considerations

Clinical diagnosis of genital herpes can be difficult because the self-limited, recurrent, painful, and vesicular or ulcerative lesions classically associated with HSV are absent in many infected persons at the time of clinical evaluation. If genital lesions are present, clinical diagnosis of genital herpes should be confirmed by type-specific virologic testing from the lesion by NAAT or culture (*186*). Recurrences and subclinical shedding are much more frequent for HSV-2 genital herpes infection than for HSV-1 genital herpes (*439*,*440*). Therefore, prognosis and counseling depend on which HSV type is present. Type-specific serologic tests can be used to aid in the diagnosis of HSV infection in the absence of genital lesions. Both type-specific virologic and type-specific serologic tests for HSV should be available in clinical settings that provide care to persons with or at risk for STIs. HSV-2 genital herpes infection increases the risk for acquiring HIV twofold to threefold; therefore, all persons with genital herpes should be tested for HIV (*441*).

##### Virologic Tests

HSV NAAT assays are the most sensitive tests because they detect HSV from genital ulcers or other mucocutaneous lesions; these tests are increasingly available (*442*–*444*). Although multiple FDA-cleared assays exist for HSV detection, these tests vary in sensitivity from 90.9% to 100%; however, they are considered highly specific (*445*–*447*). PCR is also the test of choice for diagnosing HSV infections affecting the central nervous system (CNS) and systemic infections (e.g., meningitis, encephalitis, and neonatal herpes). HSV PCR of the blood should not be performed to diagnose genital herpes infection, except in cases in which concern exists for disseminated infection (e.g., hepatitis). In certain settings, viral culture is the only available virologic test. The sensitivity of viral culture is low, especially for recurrent lesions, and decreases rapidly as lesions begin to heal (*443*,*448*). Viral culture isolates and PCR amplicons should be typed to determine whether HSV-1 or HSV-2 is causing the infection. Failure to detect HSV by NAAT or culture, especially in the presence of older lesions or the absence of active lesions, does not indicate an absence of HSV infection because viral shedding is intermittent. Similarly, random or blind genital swabs in the absence of lesions should not be used to diagnose genital HSV infection because sensitivity is low, and a negative result does not exclude the presence of HSV infection.

Cytologic detection of cellular changes associated with HSV infection is an insensitive and nonspecific method of diagnosing genital lesions (i.e., Tzanck preparation) and therefore should not be relied on. Although a direct immunofluorescence assay using fluorescein-labeled monoclonal antibodies is also available for detecting HSV antigen from genital specimens, this assay lacks sensitivity and is not recommended (*449*).

##### Type-Specific Serologic Tests

Both type-specific and type-common antibodies to HSV develop during the first weeks after infection and persist indefinitely. The majority of available, accurate type-specific HSV serologic assays are based on the HSV-specific glycoprotein G2 (gG2) (HSV-2) and glycoprotein G1 (gG1) (HSV-1). Type-common antibody tests do not distinguish between HSV-1 and HSV-2 infection; therefore, type-specific serologic assays should be requested (*450*–*452*).

Both laboratory-based assays and POC tests that provide results for HSV-2 antibodies from capillary blood or serum during a clinic visit are available. The sensitivity of glycoprotein G type-specific tests for detecting HSV-2 antibody varies from 80% to 98%; false-negative results might be more frequent at early stages of infection (*451*,*453*,*454*). Therefore, in cases of recent suspected HSV-2 acquisition, repeat type-specific antibody testing 12 weeks after the presumed time of acquisition is indicated. The most commonly used test, HerpeSelect HSV-2 enzyme immunoassay (EIA), often is falsely positive at low index values (1.1–3.0) (*457*–*457*). One study reported an overall specificity of 57.4%, with a specificity of 39.8% for index values of 1.1–2.9 (*458*). Because of the poor specificity of commercially available type-specific EIAs, particularly with low index values (<3.0), a confirmatory test (Biokit or Western blot) with a second method should be performed before test interpretation. Use of confirmatory testing with the Biokit or the Western blot assays have been reported to improve accuracy of HSV-2 serologic testing (*459*). The HerpeSelect HSV-2 immunoblot should not be used for confirmation because it uses the same antigen as the HSV-2 EIA. If confirmatory tests are unavailable, patients should be counseled about the limitations of available testing before obtaining serologic tests, and health care providers should be aware that false-positive results occur. Immunoglobulin M (IgM) testing for HSV-1 or HSV-2 is not useful because IgM tests are not type specific and might be positive during recurrent genital or oral episodes of herpes (*460*). Therefore, HSV IgM testing is not recommended.

Because approximately all HSV-2 infections are sexually acquired, presence of type-specific HSV-2 antibody implies anogenital infection. In this instance, education and counseling for persons with genital HSV infections should be provided. The presence of HSV-1 antibody alone is more difficult to interpret. HSV-1 serologic testing does not distinguish between oral and genital infection and typically should not be performed for diagnosing genital HSV-1 infection. Persons with HSV-1 antibodies often have oral HSV infection acquired during childhood, which might be asymptomatic. Lack of symptoms in a person who is HSV-1 seropositive does not distinguish anogenital from orolabial or cutaneous infection, and, regardless of site of infection, these persons remain at risk for acquiring HSV-2. In addition, HSV-1 serologic testing has low sensitivity for detection of HSV-1 antibody (*458*). However, acquisition of HSV-1 genital herpes is increasing, and HSV-1 genital herpes also can be asymptomatic (*437*–*439*,*461*,*462*). Diagnosis of HSV-1 infection is confirmed by virologic tests from genital lesions.

Type-specific HSV-2 serologic assays for diagnosing HSV-2 are useful in the following scenarios: recurrent or atypical genital symptoms or lesions with a negative HSV PCR or culture result, clinical diagnosis of genital herpes without laboratory confirmation, and a patient’s partner has genital herpes. HSV-2 serologic screening among the general population is not recommended. Patients who are at higher risk for infection (e.g., those presenting for an STI evaluation, especially for persons with ≥10 lifetime sex partners, and persons with HIV infection) might need to be assessed for a history of genital herpes symptoms, followed by type-specific HSV serologic assays to diagnose genital herpes for those with genital symptoms.

#### Genital Herpes Management

Antiviral medication offers clinical benefits to symptomatic patients and is the mainstay of management. The goals for use of antiviral medications to treat genital herpes infection are to treat or prevent symptomatic genital herpes recurrences and improve quality of life and suppress the virus to prevent transmission to sexual partners. Counseling regarding the natural history of genital herpes, risks for sexual and perinatal transmission, and methods for reducing transmission is also integral to clinical management.

Systemic antiviral drugs can partially control the signs and symptoms of genital herpes when used to treat first clinical and recurrent episodes or when used as daily suppressive therapy. However, these drugs neither eradicate latent virus nor affect the risk, frequency, or severity of recurrences after the drug is discontinued. Randomized trials have indicated that three FDA-approved antiviral medications provide clinical benefit for genital herpes: acyclovir, valacyclovir, and famciclovir (*463*–*471*). Valacyclovir is the valine ester of acyclovir and has enhanced absorption after oral administration, allowing for less frequent dosing than acyclovir. Famciclovir also has high oral bioavailability. Topical therapy with antiviral drugs offers minimal clinical benefit and is discouraged.

##### First Clinical Episode of Genital Herpes

Newly acquired genital herpes can cause a prolonged clinical illness with severe genital ulcerations and neurologic involvement. Even persons with first-episode herpes who have mild clinical manifestations initially can experience severe or prolonged symptoms during recurrent infection. Therefore, all patients with first episodes of genital herpes should receive antiviral therapy.

Recommended Regimens for First Clinical Episode of Genital Herpes*
**Acyclovir^†^** 400 mg orally 3 times/day for 7–10 days
*or*
**Famciclovir** 250 mg orally 3 times/day for 7–10 days
*or*
**Valacyclovir** 1 g orally 2 times/day for 7–10 days
* Treatment can be extended if healing is incomplete after 10 days of therapy.
^† ^Acyclovir 200 mg orally 5 times/day is also effective but is not recommended because of the frequency of dosing.

##### Recurrent HSV-2 Genital Herpes

Almost all persons with symptomatic first-episode HSV-2 genital herpes subsequently experience recurrent episodes of genital lesions. Intermittent asymptomatic shedding occurs among persons with HSV-2 genital herpes infection, even those with longstanding clinically silent infection. Antiviral therapy for recurrent genital herpes can be administered either as suppressive therapy to reduce the frequency of recurrences or episodically to ameliorate or shorten the duration of lesions. Certain persons, including those with mild or infrequent recurrent outbreaks, benefit from antiviral therapy; therefore, options for treatment should be discussed. Many persons prefer suppressive therapy, which has the additional advantage of decreasing the risk for transmitting HSV-2 genital herpes to susceptible partners (*472*,*473*).

##### Suppressive Therapy for Recurrent HSV-2 Genital Herpes

Suppressive therapy reduces frequency of genital herpes recurrences by 70%–80% among patients who have frequent recurrences (*469*–*472*). Persons receiving such therapy often report having experienced no symptomatic outbreaks. Suppressive therapy also is effective for patients with less frequent recurrences. Long-term safety and efficacy have been documented among patients receiving daily acyclovir, valacyclovir, and famciclovir (*474*). Quality of life is improved for many patients with frequent recurrences who receive suppressive therapy rather than episodic treatment (*475*). Providers should discuss with patients on an annual basis whether they want to continue suppressive therapy because frequency of genital HSV-2 recurrence diminishes over time for many persons. However, neither treatment discontinuation nor laboratory monitoring is necessary because adverse events and development of HSV antiviral resistance related to long-term antiviral use are uncommon.

Treatment with valacyclovir 500 mg daily decreases the rate of HSV-2 transmission for discordant heterosexual couples in which a partner has a history of genital HSV-2 infection (*473*). Such couples should be encouraged to consider suppressive antiviral therapy as part of a strategy for preventing transmission, in addition to consistent condom use and avoidance of sexual activity during recurrences. Suppressive antiviral therapy for persons with a history of symptomatic genital herpes also is likely to reduce transmission when used by those who have multiple partners. HSV-2 seropositive persons without a history of symptomatic genital herpes have a 50% decreased risk for genital shedding, compared with those with symptomatic genital herpes (*476*). No data are available regarding efficacy of suppressive therapy for preventing HSV-2 transmission among discordant couples in which a partner has a history of asymptomatic HSV-2 infection identified by a positive HSV-2 serologic test. Among HSV-2 seropositive persons without HIV infection, oral TDF/FTC and intravaginal tenofovir are ineffective at reducing the risk for HSV-2 shedding or recurrences (*477*).

**Recommended Regimens for Suppression of Recurrent HSV-2 Genital Herpes**
**Acyclovir** 400 mg orally 2 times/day
*or*
**Valacyclovir** 500 mg orally once a day*
*or*
**Valacyclovir** 1 g orally once a day
*or*
**Famciclovir **250 mg orally 2 times/day
* Valacyclovir 500 mg once a day might be less effective than other valacyclovir or acyclovir dosing regimens for persons who have frequent recurrences (i.e., ≥10 episodes/year).

Famciclovir appears somewhat less effective for suppression of viral shedding (*478*). Ease of administration and cost also are key considerations for prolonged treatment.

##### Recurrent HSV-1 Genital Herpes

Recurrences are less frequent after the first episode of HSV-1 genital herpes, compared with genital HSV-2 genital herpes, and genital shedding rapidly decreases during the first year of infection (*479*). No data are available regarding the efficacy of suppressive therapy for preventing transmission among persons with HSV-1 genital herpes infection. Because of the decreased risk for recurrences and shedding, suppressive therapy for HSV-1 genital herpes should be reserved for those with frequent recurrences through shared clinical decision-making between the patient and the provider.

##### Episodic Therapy for Recurrent HSV-2 Genital Herpes

Episodic treatment of recurrent herpes is most effective if therapy is initiated within 1 day of lesion onset or during the prodrome that precedes some outbreaks. The patient should be provided with a supply of drug or a prescription for the medication with instructions to initiate treatment immediately when symptoms begin. Acyclovir, famciclovir, and valacyclovir appear equally effective for episodic treatment of genital herpes (*466*–*470*).

Recommended Regimens for Episodic Therapy for Recurrent HSV-2 Genital Herpes*
**Acyclovir** 800 mg orally 2 times/day for 5 days
*or*
**Acyclovir** 800 mg orally 3 times/day for 2 days
*or*
**Famciclovir** 1 g orally 2 times/day for 1 day
*or*
**Famciclovir** 500 mg orally once, followed by 250 mg 2 times/day for 2 days
*or*
**Famciclovir** 125 mg orally 2 times/day for 5 days
*or*
**Valacyclovir** 500 mg orally 2 times/day for 3 days
*or*
**Valacyclovir** 1 g orally once daily for 5 days
*Acyclovir 400 mg orally 3 times/day for 5 days is also effective but is not recommended because of frequency of dosing.

##### Severe Disease

Intravenous (IV) acyclovir therapy (5–10 mg/kg body weight IV every 8 hours) should be provided for patients who have severe HSV disease or complications that necessitate hospitalization (e.g., disseminated infection, pneumonitis, or hepatitis) or CNS complications (e.g., meningitis or encephalitis). HSV-2 meningitis is a rare complication of HSV-2 genital herpes infection that affects women more than men (*480*). IV therapy should be considered until clinical improvement followed by oral antiviral therapy to complete >10 days of total therapy. Longer duration is recommended for CNS complications. HSV-2 meningitis is characterized clinically by signs of headache, photophobia, fever, meningismus, and cerebrospinal fluid (CSF) lymphocytic pleocytosis, accompanied by mildly elevated protein and normal glucose (*481*). Optimal therapies for HSV-2 meningitis have not been well studied (*482*); however, acyclovir 5–10 mg/kg body weight IV every 8 hours until clinical improvement is observed, followed by high-dose oral antiviral therapy (valacyclovir 1 g 3 times/day) to complete a 10- to 14-day course of total therapy, is recommended. For patients with previous episodes of documented HSV-2 meningitis, oral valacyclovir may be used for the entire course during episodes of recurrent HSV-2 meningitis. A randomized clinical trial indicated that suppressive therapy (valacyclovir 500 mg 2 times/day) did not prevent recurrent HSV-2 meningitis episodes; however, the dose might not have been sufficient for CNS penetration (*483*). Valacyclovir 500 mg 2 times/day is not recommended for suppression of HSV-2 meningitis; higher doses have not been studied in clinical trials. HSV meningitis should be distinguished from encephalitis, which requires a longer course (14–21 days) of IV therapy. Impaired renal function warrants an adjustment in acyclovir dosage.

#### Hepatitis

Hepatitis is a rare manifestation of disseminated HSV infection, often reported among pregnant women who acquire HSV during pregnancy (*484*). Pregnant women in any trimester can present with fever and hepatitis (markedly elevated transaminases) but might not have any genital or skin lesions. HSV hepatitis is associated with fulminant liver failure and high mortality (25%). Therefore, a high index of suspicion for HSV is necessary, with a confirmatory diagnosis by HSV PCR from blood (*485*). Among pregnant women with fever and unexplained severe hepatitis, disseminated HSV infection should be considered, and empiric IV acyclovir should be initiated pending confirmation (*484*).

#### Prevention

Consistent and correct condom use has been reported in multiple studies to decrease, but not eliminate, the risk for HSV-2 transmission from men to women (*486*–*488*). Condoms are less effective for preventing transmission from women to men (*489*). Two randomized clinical trials of medical male circumcision (MMC) demonstrated a decreased risk for HSV-2 acquisition among men in Uganda and South Africa (*66*,*68*). Results from a third trial conducted in Kenya did not demonstrate a substantial difference in HSV-2 acquisition among men who received MMC (*490*). A systematic review indicated high consistency for decreased risk for HSV-2 acquisition among women with a male partner who underwent MMC (*491*). These data indicate that MMC can be associated with decreased risk for HSV-2 acquisition among adult heterosexual men and with decreased risk for HSV-2 transmission from male to female partners.

Randomized clinical trials have demonstrated that PrEP with daily oral TDF/FTC decreases the risk for HSV-2 acquisition by 30% in heterosexual partnerships (*492*). Pericoital intravaginal tenofovir 1% gel also decreases the risk for HSV-2 acquisition among heterosexual women (*493*). Among MSM and transgender women, daily oral TDF/FTC decreases the risk for severe ulcers with symptomatic genital HSV-2 infection but not for HSV-2 acquisition (*494*). Insufficient evidence exists that TDF/FTC use among those who are not at risk for HIV acquisition will prevent HSV-2 infection, and it should not be used for that sole purpose. Oral TDF does not prevent HSV-2 acquisition among persons with HIV infection who are taking TDF as part of their ART regimen (*495*). No data indicate that antivirals (acyclovir, valacyclovir, or famciclovir) can be taken as PrEP by persons without HSV-2 to prevent its acquisition.

#### Counseling

Counseling of persons with genital herpes and their sex partners is crucial for management. The goals of counseling include helping patients cope with the infection and preventing sexual and perinatal transmission. Although initial counseling can be provided at the first visit, patients often benefit from learning about the chronic aspects of the disease after the acute illness subsides. Multiple resources, including Internet sites and printed materials, are available to assist patients, their partners, and clinicians who provide counseling (*496*,*497*) (https://www.ashasexualhealth.org and https://www.cdc.gov/std/herpes).

Although the psychological effect of a serologic diagnosis of HSV-2 infection in a person with asymptomatic or unrecognized genital herpes appears minimal and transient (*498*,*499*), certain persons with HSV infection might express anxiety concerning genital herpes that does not reflect the actual clinical severity of their disease; the psychological effect of HSV infection can be substantial. Common concerns about genital herpes include the severity of initial clinical manifestations, recurrent episodes, sexual relationships and transmission to sex partners, and ability to bear healthy children.

##### Symptomatic HSV-2 Genital Herpes

When counseling persons with symptomatic HSV-2 genital herpes infection, the provider should discuss the following:

- The natural history of the disease, with emphasis on the potential for recurrent episodes, asymptomatic viral shedding, and the attendant risks for sexual transmission of HSV to occur during asymptomatic periods (asymptomatic viral shedding is most frequent during the first 12 months after acquiring HSV-2).
- The effectiveness of daily suppressive antiviral therapy for preventing symptomatic recurrent episodes of genital herpes for persons experiencing a first episode or recurrent genital herpes.
- The effectiveness of daily use of valacyclovir in reducing risk for transmission of HSV-2 among persons without HIV (*473*) and use of episodic therapy to shorten the duration of recurrent episodes.
- The importance of informing current sex partners about genital herpes and informing future partners before initiating a sexual relationship.
- The importance of abstaining from sexual activity with uninfected partners when lesions or prodromal symptoms are present.
- The effectiveness of male latex condoms, which when used consistently and correctly can reduce, but not eliminate, the risk for genital herpes transmission (*486*–*488*).
- The type-specific serologic testing of partners of persons with symptomatic HSV-2 genital herpes to determine whether such partners are already HSV seropositive or whether risk for acquiring HSV exists.
- The low risk for neonatal HSV except when genital herpes is acquired late in pregnancy or if prodrome or lesions are present at delivery.
- The increased risk for HIV acquisition among HSV-2 seropositive persons who are exposed to HIV (*76*,*471*).
- The lack of effectiveness of episodic or suppressive therapy among persons with HIV infection to reduce risk for transmission to partners who might be at risk for HSV-2 acquisition.

##### Asymptomatic HSV-2 Genital Herpes

When counseling persons with asymptomatic HSV-2 genital herpes infection, the provider should consider the following:

- Asymptomatic persons who receive a diagnosis of HSV-2 by type-specific serologic testing (with confirmatory testing, if needed) should receive education about the symptoms of genital herpes infection (see Diagnostic Considerations).
- Episodic and suppressive antiviral therapies are used predominantly to treat recurrences, prevent recurrences, and prevent transmission to sex partners of persons with symptomatic HSV-2 infection.
- For patients with serological evidence of HSV-2 (with combination testing if needed) without symptomatic recurrences, neither episodic nor suppressive therapy is indicated for prevention of recurrences (see Diagnostic Considerations).
- Among persons with asymptomatic infection, the efficacy of suppressive therapy to prevent HSV-2 transmission to sex partners has not been studied.
- Because of the decreased risk for shedding among those with asymptomatic HSV-2 genital herpes, the benefit of suppressive therapy for preventing transmission is unknown among this population.

##### HSV-1 Genital Herpes

When counseling persons with HSV-1 genital herpes infection, the provider should consider the following:

- Persons with virologic laboratory-documented symptomatic HSV-1 genital herpes infection should be educated that the risk for recurrent genital herpes and genital shedding is lower with HSV-1 infection, compared with HSV-2 infection.
- Because of the decreased risk for recurrences and shedding, suppressive therapy for HSV-1 genital herpes should be reserved for those with frequent recurrences.
- For patients with frequently recurring HSV-1 genital herpes, suppressive therapy might be considered. Suppressive therapy to prevent HSV-1 transmission to sex partners has not been studied.

For persons with symptomatic HSV-1 genital herpes or asymptomatic HSV-2 genital herpes, suppressive therapy can be considered for those who have substantial psychosocial distress caused by the diagnosis of genital herpes. For women who have genital herpes, the providers who care for them during pregnancy and those who will care for their newborn infant should be informed of their infection (see Genital Herpes During Pregnancy).

#### Management of Sex Partners

The sex partners of persons who have symptomatic genital herpes can benefit from evaluation and counseling. Symptomatic sex partners should be evaluated and treated in the same manner as patients who have symptomatic genital herpes. Asymptomatic sex partners of patients who have symptomatic genital herpes should be asked about a history of genital symptoms and offered type-specific serologic testing for HSV-2. For partners without genital herpes, no data are available on which to base a recommendation for PEP or PrEP with antiviral medications or that they would prevent acquisition, and this should not be offered to patients as a prevention strategy.

#### Special Considerations

##### Drug Allergy, Intolerance, or Adverse Reactions

Allergic and other adverse reactions to oral acyclovir, valacyclovir, and famciclovir are rare. Desensitization to acyclovir has been described (*500*).

##### HIV Infection

Immunocompromised patients can have prolonged or severe episodes of genital, perianal, or oral herpes. Lesions caused by HSV are common among persons with HIV infection and might be severe, painful, and atypical (*501*). HSV shedding is increased among persons with HIV infection (*502*). Whereas ART reduces the severity and frequency of symptomatic genital herpes, frequent subclinical shedding still occurs (*503*,*504*). Clinical manifestations of genital herpes might worsen during immune reconstitution early after initiation of ART. HSV-2 type-specific serologic testing can be considered for persons with HIV infection during their initial evaluation, particularly among those with a history of genital symptoms indicative of HSV infection.

Recommended therapy for first-episode genital herpes is the same as for persons without HIV infection, although treatment courses might need to be extended for lesion resolution. Suppressive or episodic therapy with oral antiviral agents is effective in decreasing the clinical manifestations of HSV infection among persons with HIV (*503*,*504*). The risk for GUD increases during the first 6 months after starting ART, especially among persons who have a CD4^+^ T-cell count <200 cell/mm^3^. Suppressive antiviral therapy reduces the risk for GUD among this population and can be continued for 6 months after ART initiation (*504*) when the risk for GUD returns to baseline levels. Suppressive antiviral therapy among persons with HIV and HSV infection does not reduce the risk for either HIV transmission or HSV-2 transmission to susceptible sex partners (*88*,*505*). Suppressive antiviral therapy does not delay HIV disease progression and is not associated with decreased risk for HIV-related inflammation among persons taking ART (*506*). For severe HSV disease, initiating therapy with acyclovir 5–10 mg/kg IV every 8 hours might be necessary.

**Recommended Regimens for Daily Suppression of Genital Herpes Among Persons with HIV Infection**
**Acyclovir** 400–800 mg orally 2–3 times/day
*or*
**Famciclovir** 500 mg orally 2 times/day
*or*
**Valacyclovir** 500 mg orally 2 times/day
**Recommended Regimens for Episodic Genital Herpes Infection Among Persons with HIV Infection**
**Acyclovir** 400 mg orally 3 times/day for 5–10 days
*or*
**Famciclovir** 500 mg orally 2 times/day for 5–10 days
*or*
**Valacyclovir** 1 g orally 2 times/day for 5–10 days

##### Antiviral-Resistant HSV Infection

If lesions persist or recur in a patient receiving antiviral treatment, acyclovir resistance should be suspected and a viral culture obtained for phenotypic sensitivity testing (*507*). Molecular testing for acyclovir resistance is not available. Such persons should be managed in consultation with an infectious disease specialist, and alternative therapy should be administered. All acyclovir-resistant strains are also resistant to valacyclovir, and the majority are resistant to famciclovir. Foscarnet (40–80 mg/kg body weight IV every 8 hours until clinical resolution is attained) is the treatment of choice for acyclovir-resistant genital herpes (*508*,*509*). Intravenous cidofovir 5 mg/kg body weight once weekly might also be effective. Foscarnet and cidofovir are nephrotoxic medications that require intensive laboratory monitoring and infectious disease specialist consultation. Imiquimod 5% applied to the lesion for 8 hours 3 times/week until clinical resolution is an alternative that has been reported to be effective (*510*,*511*). Topical cidofovir gel 1% can be applied to lesions 2–4 times daily; however, cidofovir must be compounded at a pharmacy (*512*).

Prevention of antiviral resistance remains challenging among persons with HIV infection. Experience with another group of immunocompromised persons (e.g., hematopoietic stem-cell recipients) demonstrated that persons receiving daily suppressive antiviral therapy were less likely to experience acyclovir-resistant HSV infection compared with those who received episodic therapy for outbreaks (*513*).

##### Genital Herpes During Pregnancy

Prevention of neonatal herpes depends both on preventing acquisition of genital herpes during late pregnancy and avoiding exposure of the neonate to herpetic lesions and viral shedding during delivery. Mothers of newborns who acquire neonatal herpes often lack histories of clinically evident genital herpes (*514*,*515*). The risk for transmission to the neonate from an infected mother is high (30%–50%) among women who acquire genital herpes near the time of delivery and low (<1%) among women with prenatal histories of recurrent herpes or who acquire genital herpes during the first half of pregnancy (*516*,*517*). Women who acquire HSV in the second half of pregnancy should be managed in consultation with maternal-fetal medicine and infectious disease specialists.

All pregnant women should be asked whether they have a history of genital herpes or genital symptoms concerning for HSV infection. At the onset of labor, all women should be questioned thoroughly about symptoms of genital herpes, including prodromal symptoms (e.g., pain or burning at site before appearance of lesion), and all women should be examined thoroughly for herpetic lesions. Women without symptoms or signs of genital herpes or its prodrome can deliver vaginally. Although cesarean delivery does not eliminate the risk for HSV transmission to the neonate (*517*), women with recurrent genital herpetic lesions at the onset of labor should have a cesarean delivery to reduce the risk for neonatal HSV infection.

Routine HSV-2 serologic screening of pregnant women is not recommended. Women without known genital herpes should be counseled to abstain from vaginal intercourse during the third trimester with partners known to have or suspected of having genital herpes. In addition, to prevent HSV-1 genital herpes, pregnant women without known orolabial herpes should be advised to abstain from receptive oral sex during the third trimester with partners known to have or suspected to have orolabial herpes. Type-specific serologic tests can be useful for identifying pregnant women at risk for HSV infection and for guiding counseling regarding the risk for acquiring genital herpes during pregnancy. For example, such testing might be offered to a woman with no history of genital herpes whose sex partner has HSV infection. Many fetuses are exposed to acyclovir each year, and the medication is believed to be safe for use during all trimesters of pregnancy. A case-control study reported an increased risk for the rare neonatal outcome of gastroschisis among women who used antiviral medications between the month before conception and the third month of pregnancy (*518*). Acyclovir is also believed to be safe during breastfeeding (*431*,*519*). Although data regarding prenatal exposure to valacyclovir and famciclovir are limited, data from animal trials indicate that these drugs also pose a low risk among pregnant women (*520*). Acyclovir can be administered orally to pregnant women with first-episode genital herpes or recurrent herpes and should be administered IV to pregnant women with severe HSV (see Genital Herpes, Hepatitis). Suppressive acyclovir treatment starting at 36 weeks’ gestation reduces the frequency of cesarean delivery among women who have recurrent genital herpes by diminishing the frequency of recurrences at term (*521*–*523*). However, such treatment might not protect against transmission to neonates in all cases (*524*). No data support use of antiviral therapy among asymptomatic HSV-seropositive women without a history of genital herpes. In addition, the effectiveness of antiviral therapy among sex partners with a history of genital herpes to decrease the risk for HSV transmission to a pregnant woman has not been studied. Additional information on the clinical management of genital herpes in pregnancy is available through existing guidelines (*525*).

Recommended Regimen for Suppression of Recurrent Genital Herpes Among Pregnant Women*
**Acyclovir** 400 mg orally 3 times/day
*or*
**Valacyclovir** 500 mg orally 2 times/day
* Treatment recommended starting at 36 weeks’ gestation.

##### Neonatal Herpes

Newborn infants exposed to HSV during birth, as documented by virologic testing of maternal lesions at delivery or presumed by observation of maternal lesions, should be followed clinically in consultation with a pediatric infectious disease specialist. Detailed guidance is available regarding management of neonates who are delivered vaginally in the presence of maternal genital herpes lesions and is beyond the scope of these guidelines; more information is available from the AAP (https://redbook.solutions.aap.org). Surveillance cultures or PCR of mucosal surfaces of the neonate to detect HSV infection might be considered before the development of clinical signs of neonatal herpes to guide treatment initiation. In addition, administration of acyclovir might be considered for neonates born to women who acquired HSV near term because the risk for neonatal herpes is high for these newborn infants. All newborn infants who have neonatal herpes should be promptly evaluated and treated with systemic acyclovir. The recommended regimen for infants treated for known or suspected neonatal herpes is acyclovir 20 mg/kg body weight IV every 8 hours for 14 days if disease is limited to the skin and mucous membranes, or for 21 days for disseminated disease and disease involving the CNS.

### Granuloma Inguinale (Donovanosis)

Granuloma inguinale (donovanosis) is a genital ulcerative disease caused by the intracellular gram-negative bacterium *Klebsiella granulomatis* (formerly known as *Calymmatobacterium granulomatis*). The disease occurs rarely in the United States; however, sporadic cases have been described in India, South Africa, and South America (*526*–*535*). Although granuloma inguinale was previously endemic in Australia, it is now extremely rare (*536*,*537*). Clinically, the disease is characterized as painless, slowly progressive ulcerative lesions on the genitals or perineum without regional lymphadenopathy; subcutaneous granulomas (pseudobuboes) also might occur. The lesions are highly vascular (i.e., beefy red appearance) and can bleed. Extragenital infection can occur with infection extension to the pelvis, or it can disseminate to intra-abdominal organs, bones, or the mouth. The lesions also can develop secondary bacterial infection and can coexist with other sexually transmitted pathogens.

#### Diagnostic Considerations

The causative organism of granuloma inguinale is difficult to culture, and diagnosis requires visualization of dark-staining Donovan bodies on tissue crush preparation or biopsy. Although no FDA-cleared molecular tests for the detection of *K. granulomatis* DNA exist, molecular assays might be useful for identifying the causative agent.

#### Treatment

Multiple antimicrobial regimens have been effective; however, only a limited number of controlled trials have been published (*538*). Treatment has been reported to halt progression of lesions, and healing typically proceeds inward from the ulcer margins. Prolonged therapy is usually required to permit granulation and reepithelialization of the ulcers. Relapse can occur 6–18 months after apparently effective therapy.

**Recommended Regimen for Granuloma Inguinale (Donovanosis)**
**Azithromycin** 1 g orally once weekly or 500 mg daily for >3 weeks and until all lesions have completely healed
**Alternative Regimens**
**Doxycycline** 100 mg orally 2 times/day for at least 3 weeks and until all lesions have completely healed
*or*
**Erythromycin base** 500 mg orally 4 times/day for >3 weeks and until all lesions have completely healed
*or*
**Trimethoprim-sulfamethoxazole** one double-strength (160 mg/800 mg) tablet orally 2 times/day for >3 weeks and until all lesions have completely healed

The addition of another antibiotic to these regimens can be considered if improvement is not evident within the first few days of therapy.

#### Other Management Considerations

Patients should be followed clinically until signs and symptoms have resolved. All persons who receive a diagnosis of granuloma inguinale should be tested for HIV.

#### Follow-Up

Patients should be followed clinically until signs and symptoms resolve.

#### Management of Sex Partners

Persons who have had sexual contact with a patient who has granuloma inguinale within the 60 days before onset of the patient’s symptoms should be examined and offered therapy. However, the value of empiric therapy in the absence of clinical signs and symptoms has not been established.

#### Special Considerations

##### Pregnancy

Use of doxycycline in pregnancy might be associated with discoloration of teeth; however, the risk is not well defined. Doxycycline is compatible with breastfeeding (*431*). Sulfonamides can be associated with neonatal kernicterus among those with glucose-6-phospate dehydrogenase deficiency and should be avoided during the third trimester and while breastfeeding (*431*). For these reasons, pregnant and lactating women with granuloma inguinale should be treated with a macrolide regimen (erythromycin or azithromycin).

##### HIV Infection

Persons with granuloma inguinale and HIV infection should receive the same regimens as those who do not have HIV.

### Lymphogranuloma Venereum

LGV is caused by *C. trachomatis* serovars L1, L2, or L3 (*539*,*540*). LGV can cause severe inflammation and invasive infection, in contrast with *C. trachomatis* serovars A—K that cause mild or asymptomatic infection. Clinical manifestations of LGV can include GUD, lymphadenopathy, or proctocolitis. Rectal exposure among MSM or women can result in proctocolitis, which is the most common presentation of LGV infection (*541*), and can mimic inflammatory bowel disease with clinical findings of mucoid or hemorrhagic rectal discharge, anal pain, constipation, fever, or tenesmus (*542*,*543*). Outbreaks of LGV proctocolitis have been reported among MSM with high rates of HIV infection (*544*–*547*). LGV proctocolitis can be an invasive, systemic infection and, if it is not treated early, can lead to chronic colorectal fistulas and strictures; reactive arthropathy has also been reported. However, reports indicate that rectal LGV can also be asymptomatic (*548*). A common clinical manifestation of LGV among heterosexuals is tender inguinal or femoral lymphadenopathy that is typically unilateral. A self-limited genital ulcer or papule sometimes occurs at the site of inoculation. However, by the time persons seek care, the lesions have often disappeared. LGV-associated lymphadenopathy can be severe, with bubo formation from fluctuant or suppurative inguinal or femoral lymphadenopathy. Oral ulceration can occur and might be associated with cervical adenopathy (*549*–*551*). Persons with genital or colorectal LGV lesions can also experience secondary bacterial infection or can be infected with other sexually and nonsexually transmitted pathogens.

#### Diagnostic Considerations

A definitive LGV diagnosis can be made only with LGV-specific molecular testing (e.g., PCR-based genotyping). These tests can differentiate LGV from non–LGV *C. trachomatis* in rectal specimens. However, these tests are not widely available, and results are not typically available in a time frame that would influence clinical management. Therefore, diagnosis is based on clinical suspicion, epidemiologic information, and a *C. trachomatis* NAAT at the symptomatic anatomic site, along with exclusion of other etiologies for proctocolitis, inguinal lymphadenopathy, or genital, oral, or rectal ulcers (*551*,*552*). Genital or oral lesions, rectal specimens, and lymph node specimens (i.e., lesion swab or bubo aspirate) can be tested for *C. trachomatis* by NAAT or culture. NAAT is the preferred approach for testing because it can detect both LGV strains and non–LGV *C. trachomatis* strains (*553*). Therefore, all persons presenting with proctocolitis should be tested for chlamydia with a NAAT performed on rectal specimens. Severe symptoms of proctocolitis (e.g., bloody discharge, tenesmus, and rectal ulcers) indicate LGV. A rectal Gram stain with >10 white blood cells (WBCs) has also been associated with rectal LGV (*545*,*554*,*555*).

Chlamydia serology (complement fixation or microimmunofluorescence) should not be used routinely as a diagnostic tool for LGV because the utility of these serologic methods has not been established, interpretation has not been standardized, and validation for clinical proctitis presentation has not been done. It might support an LGV diagnosis in cases of isolated inguinal or femoral lymphadenopathy for which diagnostic material for *C. trachomatis* NAAT cannot be obtained.

#### Treatment

At the time of the initial visit (before diagnostic NAATs for chlamydia are available), persons with a clinical syndrome consistent with LGV should be presumptively treated. Presumptive treatment for LGV is indicated among patients with symptoms or signs of proctocolitis (e.g., bloody discharge, tenesmus, or ulceration); in cases of severe inguinal lymphadenopathy with bubo formation, particularly if the patient has a recent history of a genital ulcer; or in the presence of a genital ulcer if other etiologies have been ruled out. The goal of treatment is to cure infection and prevent ongoing tissue damage, although tissue reaction to the infection can result in scarring. Buboes might require aspiration through intact skin or incision and drainage to prevent formation of inguinal or femoral ulcerations.

**Recommended Regimen for Lymphogranuloma Venereum**
**Doxycycline** 100 mg orally 2 times/day for 21 days
**Alternative Regimens**
**Azithromycin** 1 g orally once weekly for 3 weeks*
*or*
**Erythromycin** base 500 mg orally 4 times/day for 21 days
* Because this regimen has not been validated, a test of cure with *C. trachomatis* NAAT 4 weeks after completion of treatment can be considered.

The optimal treatment duration for symptomatic LGV has not been studied in clinical trials. The recommended 21-day course of doxycycline is based on long-standing clinical practice and is highly effective, with an estimated cure rate of >98.5% (*555*,*556*). Shorter courses of doxycycline might be effective on the basis of a small retrospective study of MSM with rectal LGV, 50% of whom were symptomatic, who received a 7- to 14-day course of doxycycline and had a 97% cure rate (*558*). Randomized prospective studies of shorter-course doxycycline for treating LGV are needed. Longer courses of therapy might be required in the setting of fistulas, buboes, and other forms of severe disease (*559*).

A small nonrandomized study from Spain involving patients with rectal LGV demonstrated cure rates of 97% with a regimen of azithromycin 1 g once weekly for 3 weeks (*560*). Pharmacokinetic data support this dosing strategy (*561*); however, this regimen has not been validated. Fluoroquinolone-based treatments also might be effective; however, the optimal duration of treatment has not been evaluated. The clinical significance of asymptomatic LGV is unknown, and it is effectively treated with a 7-day course of doxycycline (*562*).

#### Other Management Considerations

Patients should be followed clinically until signs and symptoms have resolved. Persons who receive an LGV diagnosis should be tested for other STIs, especially HIV, gonorrhea, and syphilis. Those whose HIV test results are negative should be offered HIV PrEP.

#### Follow-Up

All persons who have been treated for LGV should be retested for chlamydia approximately 3 months after treatment. If retesting at 3 months is not possible, providers should retest at the patient’s next visit for medical care within the 12-month period after initial treatment.

#### Management of Sex Partners

Persons who have had sexual contact with a patient who has LGV within the 60 days before onset of the patient’s symptoms should be evaluated, examined, and tested for chlamydial infection, depending on anatomic site of exposure. Asymptomatic partners should be presumptively treated with a chlamydia regimen (doxycycline 100 mg orally 2 times/day for 7 days).

#### Special Considerations

##### Pregnancy

Use of doxycycline in pregnancy might be associated with discoloration of teeth; however, the risk is not well defined (*563*). Doxycycline is compatible with breastfeeding (*431*). Azithromycin might prove useful for LGV treatment during pregnancy, at a presumptive dose of 1 g weekly for 3 weeks; no published data are available regarding an effective dose and duration of treatment. Pregnant and lactating women with LGV can be treated with erythromycin, although this regimen is associated with frequent gastrointestinal side effects. Pregnant women treated for LGV should have a test of cure performed 4 weeks after the initial *C. trachomatis* NAAT-positive test.

##### HIV Infection

Persons with LGV and HIV infection should receive the same regimens as those who do not have HIV. Prolonged therapy might be required because a delay in resolution of symptoms might occur.

## Syphilis

Syphilis is a systemic disease caused by *T. pallidum*. The disease has been divided into stages on the basis of clinical findings, which guide treatment and follow-up. Persons who have syphilis might seek treatment for signs or symptoms. Primary syphilis classically presents as a single painless ulcer or chancre at the site of infection but can also present with multiple, atypical, or painful lesions (*564*). Secondary syphilis manifestations can include skin rash, mucocutaneous lesions, and lymphadenopathy. Tertiary syphilis can present with cardiac involvement, gummatous lesions, tabes dorsalis, and general paresis.

Latent infections (i.e., those lacking clinical manifestations) are detected by serologic testing. Latent syphilis acquired within the preceding year is referred to as early latent syphilis; all other cases of latent syphilis are classified as late latent syphilis or latent syphilis of unknown duration.

*T. pallidum* can infect the CNS, which can occur at any stage of syphilis and result in neurosyphilis. Early neurologic clinical manifestations or syphilitic meningitis (e.g., cranial nerve dysfunction, meningitis, meningovascular syphilis, stroke, and acute altered mental status) are usually present within the first few months or years of infection. Late neurologic manifestations (e.g., tabes dorsalis and general paresis) occur 10 to >30 years after infection.

Infection of the visual system (ocular syphilis) or auditory system (otosyphilis) can occur at any stage of syphilis but is commonly identified during the early stages and can present with or without additional CNS involvement. Ocular syphilis often presents as panuveitis but can involve structures in both the anterior and posterior segment of the eye, including conjunctivitis, anterior uveitis, posterior interstitial keratitis, optic neuropathy, and retinal vasculitis. Ocular syphilis can result in permanent vision loss. Otosyphilis typically presents with cochleo-vestibular symptoms, including tinnitus, vertigo, and sensorineural hearing loss. Hearing loss can be unilateral or bilateral, have a sudden onset, and progress rapidly. Otosyphilis can result in permanent hearing loss.

### Diagnostic Considerations

Darkfield examinations and molecular tests for detecting *T. pallidum* directly from lesion exudate or tissue are the definitive methods for diagnosing early syphilis and congenital syphilis (*565*). Although no *T. pallidum* direct-detection molecular NAATs are commercially available, certain laboratories provide locally developed and validated PCR tests for detecting *T. pallidum* DNA. A presumptive diagnosis of syphilis requires use of two laboratory serologic tests: a nontreponemal test (i.e., Venereal Disease Research Laboratory [VDRL] or rapid plasma reagin [RPR] test) and a treponemal test (i.e., the *T. pallidum* passive particle agglutination [TP-PA] assay, various EIAs, chemiluminescence immunoassays [CIAs] and immunoblots, or rapid treponemal assays) (*566*–*568*). At least 18 treponemal-specific tests are cleared for use in the United States. Use of only one type of serologic test (nontreponemal or treponemal) is insufficient for diagnosis and can result in false-negative results among persons tested during primary syphilis and false-positive results among persons without syphilis or previously treated syphilis.

#### Nontreponemal Tests and Traditional Algorithm

False-positive nontreponemal test results can be associated with multiple medical conditions and factors unrelated to syphilis, including other infections (e.g., HIV), autoimmune conditions, vaccinations, injecting drug use, pregnancy, and older age (*566*,*569*). Therefore, persons with a reactive nontreponemal test should always receive a treponemal test to confirm the syphilis diagnosis (i.e., traditional algorithm). Nontreponemal test antibody titers might correlate with disease activity and are used for monitoring treatment response. Serum should be diluted to identify the highest titer, and results should be reported quantitatively. A fourfold change in titer, equivalent to a change of two dilutions (e.g., from 1:16 to 1:4 or from 1:8 to 1:32), is considered necessary for demonstrating a clinically significant difference between two nontreponemal test results obtained by using the same serologic test, preferably from the same manufacturer to avoid variation in results. Sequential serologic tests for a patient should be performed using the same testing method (VDRL or RPR), preferably by the same laboratory. VDRL and RPR are equally valid assays; however, quantitative results from the two tests cannot be compared directly with each other because the methods are different, and RPR titers frequently are slightly higher than VDRL titers.

Nontreponemal test titers usually decrease after treatment and might become nonreactive with time. However, for certain persons, nontreponemal antibodies might decrease less than fourfold after treatment (i.e., inadequate serologic response) or might decline appropriately but fail to serorevert and persist for a long period. Atypical nontreponemal serologic test results (e.g., unusually high, unusually low, or fluctuating titers) might occur regardless of HIV status. When serologic tests do not correspond with clinical findings indicative of primary, secondary, or latent syphilis, presumptive treatment is recommended for persons with risk factors for syphilis, and use of other tests (e.g., biopsy for histology and immunostaining and PCR of lesion) should be considered. For the majority of persons with HIV infection, serologic tests are accurate and reliable for diagnosing syphilis and evaluating response to treatment.

#### Treponemal Tests and Reverse Sequence Algorithm

The majority of patients who have reactive treponemal tests will have reactive tests for the remainder of their lives, regardless of adequate treatment or disease activity. However, 15%–25% of patients treated during the primary stage revert to being serologically nonreactive after 2–3 years (*570*). Treponemal antibody titers do not predict treatment response and therefore should not be used for this purpose.

Clinical laboratories sometimes screen syphilis serologic samples by using automated treponemal immunoassays, typically by EIA or CIA (*571*–*573*). This reverse sequence algorithm for syphilis testing can identify persons previously treated for syphilis, those with untreated or incompletely treated syphilis, and those with false-positive results that can occur with a low likelihood of infection (*574*). Persons with a positive treponemal screening test should have a standard quantitative nontreponemal test with titer performed reflexively by the laboratory to guide patient management decisions. If the nontreponemal test is negative, the laboratory should perform a treponemal test different from the one used for initial testing, preferably TP-PA or treponemal assay based on different antigens than the original test, to adjudicate the results of the initial test.

If a second treponemal test is positive (e.g., EIA reactive, RPR nonreactive, or TP-PA reactive), persons with a history of previous treatment will require no further management unless sexual history indicates a reexposure. In this instance, a repeat nontreponemal test 2–4 weeks after a confirmed medical history and physical examination is recommended to evaluate for early infection. Those without a history of treatment for syphilis should be offered treatment. Unless a medical history or results of a physical examination indicate a recent infection, previously untreated persons should be treated for syphilis of unknown duration or late latent syphilis.

If the second treponemal test is negative (e.g., EIA reactive, RPR nonreactive, TP-PA nonreactive) and the epidemiologic risk and clinical probability for syphilis are low, further evaluation or treatment is not indicated.

Multiple studies demonstrate that high quantitative index values or high signal-to-cutoff ratio from treponemal EIA or CIA tests correlate with TP-PA positivity, which might eliminate the need for additional confirmatory testing; however, the range of index values varies among different treponemal immunoassays, and the values that correspond to high levels of reactivity with confirmatory testing might differ by immunoassay (*567*,*575*–*582*).

#### Cerebrospinal Fluid Evaluation

Further testing with CSF evaluation is warranted for persons with clinical signs of neurosyphilis (e.g., cranial nerve dysfunction, meningitis, stroke, acute or chronic altered mental status, or loss of vibration sense). All patients with ocular symptoms and reactive syphilis serology need a full ocular examination, including cranial nerve evaluation. If cranial nerve dysfunction is present, a CSF evaluation is needed. Among persons with isolated ocular symptoms (i.e., no cranial nerve dysfunction or other neurologic abnormalities), confirmed ocular abnormalities on examination, and reactive syphilis serology, a CSF examination is unnecessary before treatment. CSF analysis can be helpful in evaluating persons with ocular symptoms and reactive syphilis serology who do not have ocular findings or cranial nerve dysfunction on examination. Among patients with isolated auditory abnormalities and reactive syphilis serology, CSF evaluation is likely to be normal and is unnecessary before treatment (*583*,*584*).

Laboratory testing is helpful in supporting the diagnosis of neurosyphilis; however, no single test can be used to diagnose neurosyphilis in all instances. Diagnosis of neurosyphilis depends on a combination of CSF tests (e.g., CSF cell count, protein, or reactive CSF-VDRL) in the presence of reactive serologic test (nontreponemal and treponemal) results and neurologic signs and symptoms. CSF laboratory abnormalities are common for persons with early syphilis and are of unknown medical significance in the absence of neurologic signs or symptoms (*585*). CSF-VDRL is highly specific but insensitive. For a person with neurologic signs or symptoms, a reactive CSF-VDRL (in the absence of blood contamination) is considered diagnostic of neurosyphilis.

When CSF-VDRL is negative despite clinical signs of neurosyphilis, reactive serologic tests results, lymphocytic pleocytosis, or protein, neurosyphilis should be considered. In that instance, additional evaluation by using fluorescent treponemal-antibody absorption (FTA-ABS) or TP-PA testing on CSF might be warranted. The CSF FTA-ABS test is less specific for neurosyphilis than the CSF-VDRL but is highly sensitive. Fewer data are available regarding CSF TP-PA; however, the sensitivity and specificity appear similar to the CSF FTA-ABS (*586*). Neurosyphilis is highly unlikely with a negative CSF FTA-ABS or TP-PA test, especially among persons with nonspecific neurologic signs and symptoms (*587*).

Among persons with HIV infection, CSF leukocyte count can be elevated (>5 WBCs/mm^3^); the association with CSF leukocyte count and plasma HIV viral suppression has not been well characterized. Using a higher cutoff (>20 WBCs/mm^3^) might improve the specificity of neurosyphilis diagnosis among this population (*588*).

### Treatment

Penicillin G, administered parenterally, is the preferred drug for treating patients in all stages of syphilis. The preparation used (i.e., benzathine, aqueous procaine, or aqueous crystalline), dosage, and length of treatment depend on the stage and clinical manifestations of the disease. Treatment for late latent syphilis (>1 years’ duration) and tertiary syphilis requires a longer duration of therapy because organisms theoretically might be dividing more slowly (the validity of this rationale has not been assessed). Longer treatment duration is required for persons with latent syphilis of unknown duration to ensure that those who did not acquire syphilis within the preceding year are adequately treated.

Selection of the appropriate penicillin preparation is important because *T. pallidum* can reside in sequestered sites (e.g., the CNS and aqueous humor) that are poorly accessed by certain forms of penicillin. Combinations of benzathine penicillin, procaine penicillin, and oral penicillin preparations are not considered appropriate for syphilis treatment. Reports have indicated that practitioners have inadvertently prescribed combination long- and short-acting benzathine-procaine penicillin (Bicillin C-R) instead of the standard benzathine penicillin product (Bicillin L-A) recommended in the United States for treating primary, secondary, and latent syphilis. Practitioners, pharmacists, and purchasing agents should be aware of the similar names of these two products to avoid using the incorrect combination therapy agent for treating syphilis (*589*).

Penicillin’s effectiveness for treating syphilis was well established through clinical experience even before the value of randomized controlled clinical trials was recognized. Therefore, approximately all recommendations for treating syphilis are based not only on clinical trials and observational studies, but on many decades of clinical experience.

#### Special Considerations

##### Pregnancy

Parenteral penicillin G is the only therapy with documented efficacy for syphilis during pregnancy. Pregnant women with syphilis at any stage who report penicillin allergy should be desensitized and treated with penicillin (see Management of Persons Who Have a History of Penicillin Allergy).

##### Jarisch-Herxheimer Reaction

The Jarisch-Herxheimer reaction is an acute febrile reaction frequently accompanied by headache, myalgia, and fever that can occur within the first 24 hours after the initiation of any syphilis therapy; it is a reaction to treatment and not an allergic reaction to penicillin. Patients should be informed about this possible adverse reaction and how to manage it if it occurs. The Jarisch-Herxheimer reaction occurs most frequently among persons who have early syphilis, presumably because bacterial loads are higher during these stages. Antipyretics can be used to manage symptoms; however, they have not been proven to prevent this reaction. The Jarisch-Herxheimer reaction might induce early labor or cause fetal distress in pregnant women; however, this should not prevent or delay therapy (*590*) (see Syphilis During Pregnancy).

#### Management of Sex Partners

Sexual transmission of *T. pallidum* is thought to occur only when mucocutaneous syphilitic lesions are present. Such manifestations are uncommon after the first year of infection. Persons exposed through sexual contact with a person who has primary, secondary, or early latent syphilis should be evaluated clinically and serologically and treated according to the following recommendations:

- Persons who have had sexual contact with a person who receives a diagnosis of primary, secondary, or early latent syphilis <90 days before the diagnosis should be treated presumptively for early syphilis, even if serologic test results are negative.
- Persons who have had sexual contact with a person who receives a diagnosis of primary, secondary, or early latent syphilis >90 days before the diagnosis should be treated presumptively for early syphilis if serologic test results are not immediately available and the opportunity for follow-up is uncertain. If serologic tests are negative, no treatment is needed. If serologic tests are positive, treatment should be based on clinical and serologic evaluation and syphilis stage.
- In certain areas or among populations with high syphilis infection rates, health departments recommend notification and presumptive treatment of sex partners of persons with syphilis of unknown duration who have high nontreponemal serologic test titers (i.e., >1:32) because high titers might be indicative of early syphilis. These partners should be managed as if the index patient had early syphilis.
- Long-term sex partners of persons who have late latent syphilis should be evaluated clinically and serologically for syphilis and treated on the basis of the evaluation’s findings.
- The following sex partners of persons with syphilis are considered at risk for infection and should be confidentially notified of the exposure and need for evaluation: partners who have had sexual contact within 3 months plus the duration of symptoms for persons who receive a diagnosis of primary syphilis, within 6 months plus duration of symptoms for those with secondary syphilis, and within 1 year for persons with early latent syphilis.

### Primary and Secondary Syphilis

#### Treatment

Parenteral penicillin G has been used effectively for achieving clinical resolution (i.e., the healing of lesions and prevention of sexual transmission) and for preventing late sequelae. However, no comparative trials have been conducted to guide selection of an optimal penicillin regimen. Substantially fewer data are available for nonpenicillin regimens.

Recommended Regimen for Primary and Secondary Syphilis* Among Adults
**Benzathine penicillin G** 2.4 million units IM in a single dose
* Recommendations for treating syphilis among persons with HIV infection and pregnant women are discussed elsewhere in this report (see Syphilis Among Persons with HIV Infection; Syphilis During Pregnancy).

Available data demonstrate that use of additional doses of benzathine penicillin G, amoxicillin, or other antibiotics do not enhance efficacy of this recommended regimen when used to treat primary and secondary syphilis, regardless of HIV status (*591*–*593*).

**Recommended Regimen for Syphilis Among Infants and Children**
**Benzathine penicillin G** 50,000 units/kg body weight IM, up to the adult dose of 2.4 million units in a single dose

Infants and children aged ≥1 month who receive a syphilis diagnosis should have birth and maternal medical records reviewed to assess whether they have congenital or acquired syphilis (see Congenital Syphilis). Infants and children aged ≥1 month with primary and secondary syphilis should be managed by a pediatric infectious disease specialist and evaluated for sexual abuse (e.g., through consultation with child protective services) (see Sexual Assault or Abuse of Children).

#### Other Management Considerations

All persons who have primary and secondary syphilis should be tested for HIV at the time of diagnosis and treatment. Those persons whose HIV test results are negative should be offered HIV PrEP. In geographic areas in which HIV prevalence is high, persons who have primary or secondary syphilis should be offered PrEP and retested for HIV in 3 months if the initial HIV test result was negative.

Persons who have syphilis and symptoms or signs indicating neurologic disease (e.g., cranial nerve dysfunction, meningitis, stroke, or altered mental state) should have an evaluation that includes CSF analysis. Persons with syphilis who have symptoms or signs of ocular syphilis (e.g., uveitis, iritis, neuroretinitis, or optic neuritis) should have a thorough cranial nerve examination and ocular slit-lamp and ophthalmologic examinations. CSF evaluation is not always needed for persons with ocular syphilis if no evidence of cranial nerves 2, 3, 4, 5, and 6 dysfunction or other evidence of neurologic disease exists. If symptoms and signs of otic syphilis are present then an otologic examination is needed; CSF evaluation in persons with otic syphilis does not aid in the clinical management and therefore is not recommended (see Cerebrospinal Fluid Evaluation). Treatment should be guided by the results of these evaluations. Invasion of CSF by *T. pallidum* accompanied by CSF laboratory abnormalities is common among adults who have primary or secondary syphilis but has unknown medical significance (*585*). In the absence of clinical neurologic findings, no evidence supports variation from the recommended treatment regimen for primary or secondary syphilis. Symptomatic neurosyphilis after treatment with the penicillin regimens recommended for primary and secondary syphilis is rare. Therefore, unless clinical signs or symptoms of neurologic or ophthalmic involvement are present, routine CSF analysis is not recommended for persons who have primary or secondary syphilis.

#### Follow-Up

Clinical and serologic evaluation should be performed at 6 and 12 months after treatment; more frequent evaluation might be prudent if opportunity for follow-up is uncertain or if repeat infection is a clinical concern. Serologic response (i.e., titer) should be compared with the titer at the time of treatment. However, assessing serologic response to treatment can be difficult, and definitive criteria for cure or failure by serologic criteria have not been well established. In addition, nontreponemal test titers might decrease more slowly for persons previously treated for syphilis (*594*,*595*).

Persons who have signs or symptoms that persist or recur and those with at least a fourfold increase in nontreponemal test titer persisting for >2 weeks likely were reinfected or experienced treatment failure. Among persons who have neurologic findings or persons with no neurologic findings without any reported sexual exposure during the previous 3–6 months indicating that treatment failure might be possible, a CSF examination is recommended with treatment guided by CSF findings. These persons should also be reevaluated for HIV infection.

Among persons with no neurologic findings after a thorough neurologic examination and who are sexually active, reinfection is likely and repeat treatment for early syphilis is recommended. These persons should also be reevaluated for HIV infection.

Failure of nontreponemal test titers to decrease fourfold within 12 months after therapy for primary or secondary syphilis (inadequate serologic response) might be indicative of treatment failure. However, clinical trial data have demonstrated that 10%–20% of persons with primary and secondary syphilis treated with the recommended therapy will not achieve the fourfold decrease in nontreponemal titer within 12 months after treatment (*591*,*596*,*597*). Serologic response to treatment appears to be associated with multiple factors, including the person’s syphilis stage (earlier stages are more likely to decrease fourfold and become nonreactive), initial nontreponemal antibody titers (titers <1:8 are less likely to decline fourfold than higher titers), and age (titers among older patients might be less likely to decrease fourfold than those of younger patients) (*596*–*598*). Optimal management of persons who have an inadequate serologic response after syphilis treatment is unclear. At a minimum, these persons should receive additional neurologic examinations, clinical and serologic follow-up annually, and reevaluation for HIV infection. If neurologic symptoms or signs are identified, a CSF evaluation is recommended, with findings guiding management. If additional follow-up cannot be ensured, retreatment is recommended. Because treatment failure might be the result of unrecognized CNS infection, CSF examination can be considered in situations in which follow-up is uncertain.

For retreatment, weekly injections of benzathine penicillin G 2.4 million units intramuscularly (IM) for 3 weeks is recommended, unless CSF examination indicates that neurosyphilis is present (see Neurosyphilis, Ocular Syphilis, and Otosyphilis). Serologic titers might not decrease, despite a negative CSF examination and a repeated 3-week therapy course (*599*). In these circumstances, the benefit of additional therapy or repeated CSF examinations is unclear, and it is not typically recommended. Serologic and clinical monitoring at least annually should continue to monitor for any sustained increases in nontreponemal titer.

#### Management of Sex Partners

See Syphilis, Management of Sex Partners.

#### Special Considerations

##### Penicillin Allergy

Data to support use of alternatives to penicillin in treating primary and secondary syphilis are limited. However, multiple therapies might be effective for nonpregnant persons with penicillin allergy who have primary or secondary syphilis. Doxycycline (100 mg orally 2 times/day for 14 days) (*600*,*601*) and tetracycline (500 mg orally 4 times/day for 14 days) have been used for years and can be effective. Compliance is likely to be better with doxycycline than tetracycline because tetracycline can cause more gastrointestinal side effects and requires more frequent dosing. Limited clinical studies, along with biologic and pharmacologic evidence, indicate that ceftriaxone (1 g daily either IM or IV for 10 days) is effective for treating primary and secondary syphilis; however, the optimal dose and duration of ceftriaxone therapy have not been defined (*602*,*603*). Azithromycin as a single 2-g oral dose has been effective for treating primary and secondary syphilis among certain populations (*602*,*604*,*605*). However, because of *T. pallidum* chromosomal mutations associated with azithromycin and other macrolide resistance and documented treatment failures in multiple U.S. geographic areas, azithromycin should not be used as treatment for syphilis (*606*–*608*). Thorough clinical and serologic follow-up of persons receiving any alternative therapy is essential.

Persons with a penicillin allergy whose compliance with therapy or follow-up cannot be ensured should be desensitized and treated with benzathine penicillin G. Skin testing for penicillin allergy might be useful in circumstances in which the reagents and expertise are available for performing the test adequately (see Management of Persons Who Have a History of Penicillin Allergy).

##### Pregnancy

Pregnant women with primary or secondary syphilis who are allergic to penicillin should be desensitized and treated with penicillin G. Skin testing or oral graded penicillin dose challenge might be helpful in identifying women at risk for acute allergic reactions (see Management of Persons Who Have a History of Penicillin Allergy; Syphilis During Pregnancy).

##### HIV Infection

Persons with HIV infection who have primary or secondary syphilis should be treated similarly to those without HIV (see Syphilis Among Persons with HIV Infection).

### Latent Syphilis

Latent syphilis is defined as syphilis characterized by seroreactivity without other evidence of primary, secondary, or tertiary disease. Persons who have latent syphilis and who acquired syphilis during the preceding year are classified as having early latent syphilis (early nonprimary, nonsecondary). Persons can receive a diagnosis of early latent syphilis if, during the year preceding the diagnosis, they had a documented seroconversion or a sustained (>2 weeks) fourfold or greater increase in nontreponemal test titers in a previously treated person; unequivocal symptoms of primary or secondary syphilis; or a sex partner documented to have primary, secondary, or early latent syphilis. In addition, for persons with reactive nontreponemal and treponemal tests whose only possible exposure occurred during the previous 12 months, early latent syphilis can be assumed.

In the absence of these conditions associated with latent syphilis, an asymptomatic person should be considered to have latent syphilis of unknown duration or late latent syphilis (>1 year’s duration). Nontreponemal serologic titers usually are higher early in the course of syphilis infection. However, early latent syphilis cannot be reliably diagnosed solely on the basis of nontreponemal titers. All persons with latent syphilis should have careful examination of all accessible mucosal surfaces to evaluate for mucosal lesions (primary or secondary syphilis) before making a latent syphilis diagnosis. Physical examination should include the oral cavity, perianal area, perineum, rectum, and genitals (vagina and cervix for women; scrotum, penis, and underneath the foreskin for uncircumcised men).

#### Treatment

Because latent syphilis is not transmitted sexually, the objective of treating persons in this disease stage is to prevent medical complications of syphilis. Latent syphilis can also be vertically transmitted to a fetus; therefore, the goal of treating a pregnant woman is to prevent congenital syphilis. Although clinical experience supports the effectiveness of penicillin in achieving this goal, limited evidence is available for guiding choice of specific regimens or duration. Available data demonstrate that additional doses of benzathine penicillin G, amoxicillin, or other antibiotics in early latent syphilis do not enhance efficacy, regardless of HIV status (*592*,*593*,*609*).

Recommended Regimens for Latent Syphilis* Among Adults
**Early latent syphilis: Benzathine penicillin G** 2.4 million units IM in a single dose
**Late latent syphilis: Benzathine penicillin G** 7.2 million units total, administered as 3 doses of 2.4 million units IM each at 1-week intervals
* Recommendations for treating syphilis in persons with HIV and pregnant women are discussed elsewhere in this report (see Syphilis Among Persons with HIV Infection; Syphilis During Pregnancy).

Infants and children aged ≥1 month with diagnosed latent syphilis should be managed by a pediatric infectious disease specialist and receive a CSF examination. In addition, birth and maternal medical records should be reviewed to assess whether these infants and children have congenital or acquired syphilis. For those with congenital syphilis, treatment should be undertaken as described (see Congenital Syphilis). Those with acquired syphilis should be evaluated for sexual abuse (e.g., through consultation with child protection services) (see Sexual Assault or Abuse of Children). These regimens are for children who are not allergic to penicillin who have acquired syphilis and who have normal CSF examinations.

#### Other Management Considerations

All persons who have latent syphilis should be tested for HIV at the time of diagnosis or treatment. Those persons whose HIV test results are negative should be offered HIV PrEP. In geographic areas in which the prevalence of HIV infection is high or among populations vulnerable to HIV acquisition, persons who have early latent or late latent syphilis should be offered PrEP and retested for HIV in 3 months if the first HIV test result was negative.

Persons who receive a diagnosis of latent syphilis and have neurologic or ocular signs and symptoms (e.g., cognitive dysfunction, motor or sensory deficits, ophthalmic or auditory symptoms, cranial nerve palsies, or symptoms or signs of meningitis or stroke) should be evaluated for neurosyphilis, ocular syphilis, or otosyphilis according to their clinical presentation (see Neurosyphilis, Ocular Syphilis, and Otosyphilis).

If a person receives a delayed dose of penicillin in a course of weekly therapy for late latent syphilis or syphilis of unknown duration, the course of action that should be recommended is unclear. Clinical experience indicates that an interval of 10–14 days between doses of benzathine penicillin for latent syphilis might be acceptable before restarting the sequence of injections (i.e., if dose 1 is administered on day 0, dose 2 is administered on days 10–14). Pharmacologic considerations indicate that an interval of 7–9 days between doses, if feasible, might be preferred (*610*–*612*). Delayed doses are not optimal for pregnant women receiving therapy for latent syphilis (*613*). Pregnant women who have delays in any therapy dose >9 days between doses should repeat the full course of therapy.

#### Follow-Up

Quantitative nontreponemal serologic tests should be repeated at 6, 12, and 24 months. These serologic titers should be compared with the titer at the time of treatment. Persons with at least a fourfold sustained increase in nontreponemal test titer persisting for >2 weeks or who experienced signs or symptoms attributable to primary or secondary syphilis were likely reinfected or experienced treatment failure. These persons should be retreated and reevaluated for HIV infection. Among persons who have neurologic findings after a thorough neurologic examination or among persons with no neurologic findings and no sexual exposure during the previous year, a CSF examination is recommended. Treatment should be guided by CSF findings. Among persons with no neurologic findings after neurologic examination and who are sexually active, treatment with weekly injections of benzathine penicillin G 2.4 million units IM for 3 weeks is recommended.

Optimal management of persons who have less than a fourfold decrease in titers 24 months after treatment (i.e., an inadequate serologic response) is unclear, especially if the initial titer was <1:8. At a minimum, these persons should receive additional clinical and serologic follow-up and be evaluated for HIV infection. If neurologic symptoms or signs are identified, a CSF evaluation is recommended, with the findings guiding management. If additional follow-up cannot be ensured or if an initially high titer (>1:32) does not decrease at least fourfold 24 months after treatment, retreatment with weekly injections of benzathine penicillin G 2.4 million units IM for 3 weeks is recommended. Because treatment failure might be the result of unrecognized CNS infection, CSF examination can be considered in such situations where follow-up is uncertain or initial high titers do not decrease after 24 months.

If the CSF examination is negative, repeat treatment for latent syphilis is recommended. Serologic titers might not decrease despite a negative CSF examination and a repeated course of therapy, especially if the initial nontreponemal titer is low (<1:8); in these circumstances, the need for additional therapy or repeated CSF examinations is unclear but is usually not recommended. Serologic and clinical monitoring at least annually should continue to monitor for any sustained increases in nontreponemal titer.

#### Management of Sex Partners

See Syphilis, Management of Sex Partners.

#### Special Considerations

##### Penicillin Allergy

 The effectiveness of alternatives to penicillin in treating latent syphilis has not been well documented. Nonpregnant patients allergic to penicillin who have clearly defined early latent syphilis should respond to antibiotics recommended as alternatives to penicillin for treating primary and secondary syphilis (see Primary and Secondary Syphilis). The only acceptable alternatives for treating late latent syphilis or syphilis of unknown duration are doxycycline (100 mg orally 2 times/day) or tetracycline (500 mg orally 4 times/day), each for 28 days. The efficacy of these alternative regimens among persons with HIV infection has not been well studied. These therapies should be used only in conjunction with close serologic and clinical follow-up, especially among persons with HIV infection. On the basis of biologic plausibility and pharmacologic properties, ceftriaxone might be effective for treating latent syphilis. However, the optimal dose and duration of ceftriaxone therapy have not been defined; treatment decisions should be discussed in consultation with a specialist. Persons with a penicillin allergy whose compliance with therapy or follow-up cannot be ensured should be desensitized and treated with benzathine penicillin G. Skin testing for penicillin allergy might be useful in circumstances in which the reagents and expertise are available for performing the test adequately (see Management of Persons Who Have a History of Penicillin Allergy).

##### Pregnancy

Pregnant women who are allergic to penicillin should be desensitized and treated with penicillin G. Skin testing for penicillin allergy might be useful in circumstances in which the reagents and expertise are available for performing the test adequately (see Management of Persons Who Have a History of Penicillin Allergy; Syphilis During Pregnancy).

##### HIV Infection

Persons with HIV infection who have latent syphilis should be treated similarly to persons who do not have HIV (see Syphilis Among Persons with HIV Infection).

### Tertiary Syphilis

Tertiary syphilis refers to gummas, cardiovascular syphilis, psychiatric manifestations (e.g., memory loss or personality changes), or late neurosyphilis. Guidelines for all forms of neurosyphilis (e.g., early or late neurosyphilis) are discussed elsewhere in these recommendations (see Neurosyphilis, Ocular Syphilis, and Otosyphilis). Persons with gummas and cardiovascular syphilis who are not allergic to penicillin and have no evidence of neurosyphilis by clinical and CSF examination should be treated with the following regimen.

**Recommended Regimen for Tertiary Syphilis Among Adults **
**Tertiary syphilis with normal CSF examination: Benzathine penicillin G** 7.2 million units total, administered as 3 doses of 2.4 million units IM each at 1-week intervals

#### Other Management Considerations

All persons who have tertiary syphilis should receive a CSF examination before therapy is initiated and have an HIV test. Those persons whose HIV test results are negative should be offered HIV PrEP. Persons with CSF abnormalities should be treated with a neurosyphilis regimen. Certain providers treat all persons who have cardiovascular syphilis with a neurosyphilis regimen. These persons should be managed in consultation with an infectious disease specialist. Limited information is available concerning clinical response and follow-up of persons who have tertiary syphilis.

#### Management of Sex Partners

See Syphilis, Management of Sex Partners.

#### Special Considerations

##### Penicillin Allergy

Any person allergic to penicillin should be treated in consultation with an infectious disease specialist.

##### Pregnancy

Pregnant women who are allergic to penicillin should be desensitized and treated with penicillin G. Skin testing or oral graded penicillin dose challenge might be helpful in identifying women at risk for acute allergic reactions (see Management of Persons Who Have a History of Penicillin Allergy; Syphilis During Pregnancy).

##### HIV Infection

Persons with HIV infection who have tertiary syphilis should be treated as described for persons without HIV (see Syphilis Among Persons with HIV Infection).

### Neurosyphilis, Ocular Syphilis, and Otosyphilis

#### Treatment

CNS involvement can occur during any stage of syphilis, and CSF laboratory abnormalities are common among persons with early syphilis, even in the absence of clinical neurologic findings. No evidence exists to support variation from recommended diagnosis and treatment for syphilis at any stage for persons without clinical neurologic findings, except tertiary syphilis. If clinical evidence of neurologic involvement is observed (e.g., cognitive dysfunction, motor or sensory deficits, cranial nerve palsies, or symptoms or signs of meningitis or stroke), a CSF examination should be performed before treatment.

Syphilitic uveitis or other ocular syphilis manifestations (e.g., neuroretinitis and optic neuritis) can occur at any stage of syphilis and can be isolated abnormalities or associated with neurosyphilis. All persons with ocular symptoms and reactive syphilis serology need a full ocular examination, including cranial nerve evaluation. If cranial nerve dysfunction is present, a CSF evaluation is needed. Among persons with isolated ocular symptoms (no cranial nerve dysfunction or other neurologic abnormalities), reactive syphilis serology, and confirmed ocular abnormalities on examination, CSF examination is unnecessary before treatment. CSF analysis might be helpful in evaluating persons with ocular symptoms and reactive syphilis serology who do not have ocular findings on examination. If ocular syphilis is suspected, immediate referral to and management in collaboration with an ophthalmologist is crucial. Ocular syphilis should be treated similarly to neurosyphilis, even if a CSF examination is normal.

Hearing loss and other otologic symptoms can occur at any stage of syphilis and can be isolated abnormalities or associated with neurosyphilis, especially of cranial nerve 8. However, among persons with isolated auditory symptoms, normal neurologic examination, and reactive syphilis serology, CSF examination is likely to be normal and is not recommended before treatment. Otosyphilis should be managed in collaboration with an otolaryngologist and treated by using the same regimen as for neurosyphilis.

**Recommended Regimen for Neurosyphilis, Ocular Syphilis, or Otosyphilis Among Adults **
**Aqueous crystalline penicillin G** 18–24 million units per day, administered as 3–4 million units IV every 4 hours or continuous infusion for 10–14 days

If compliance with therapy can be ensured, the following alternative regimen might be considered.

**Alternative Regimen**
**Procaine penicillin G** 2.4 million units IM once daily
*plus*
**Probenecid **500 mg orally 4 times/day, both for 10–14 days

The durations of the recommended and alternative regimens for neurosyphilis are shorter than the duration of the regimen used for latent syphilis. Therefore, benzathine penicillin, 2.4 million units IM once per week for 1–3 weeks, can be considered after completion of these neurosyphilis treatment regimens to provide a comparable total duration of therapy.

#### Other Management Considerations

The following are other considerations in the management of persons who have neurosyphilis:

- All persons who have neurosyphilis, ocular syphilis, or otosyphilis should be tested for HIV at the time of diagnosis. Those whose HIV test results are negative should be offered HIV PrEP.
- Although systemic steroids are used frequently as adjunctive therapy for otosyphilis and for ocular syphilis, such drugs have not been proven to be beneficial.

#### Follow-Up

Data from two studies indicate that, among immunocompetent persons and persons with HIV infection who are on effective ART, normalization of the serum RPR titer predicts normalization of abnormal CSF parameters after neurosyphilis treatment (*614*,*615*). Therefore, repeated CSF examinations are unnecessary for persons without HIV infection or persons with HIV infection who are on ART and who exhibit serologic and clinical responses after treatment.

#### Management of Sex Partners

See Syphilis, Management of Sex Partners.

#### Special Considerations

##### Penicillin Allergy

Limited data indicate that ceftriaxone 1–2 g daily either IM or IV for 10–14 days can be used as an alternative treatment for persons with neurosyphilis (*603*,*616*,*617*). Cross-sensitivity between ceftriaxone and penicillin can occur; however, the risk for penicillin cross-reactivity between third-generation cephalosporins is negligible (*618*–*621*) (see Management of Persons Who Have a History of Penicillin Allergy). If concern exists regarding ceftriaxone safety for a patient with neurosyphilis, skin testing should be performed to confirm penicillin allergy and, if necessary, penicillin desensitization in consultation with a specialist is recommended. Other regimens have not been adequately evaluated for treatment of neurosyphilis.

##### Pregnancy

Pregnant women who are allergic to penicillin should be desensitized and treated with penicillin G. Skin testing or oral graded penicillin dose challenge might be helpful in identifying women at risk for acute allergic reactions (see Management of Persons Who Have a History of Penicillin Allergy).

##### HIV Infection

Persons with HIV infection who have neurosyphilis should be treated as described for persons without HIV (see Syphilis Among Persons with HIV Infection).

### Syphilis Among Persons with HIV Infection

#### Diagnostic Considerations

Interpretation of treponemal and nontreponemal serologic tests for persons with HIV infection is the same as for persons without HIV. Although rare, unusual serologic responses have been observed among persons with HIV infection who have syphilis. The majority of reports have involved posttreatment serologic titers that were higher than expected (i.e., high serofast) or fluctuated, and false-negative serologic test results and delayed appearance of seroreactivity have also been reported (*622*).

When clinical findings are indicative of syphilis, but serologic tests are nonreactive or their interpretation is unclear, alternative tests (e.g., biopsy of a lesion, darkfield examination, or PCR of lesion material) might be useful for diagnosis. Neurosyphilis, ocular syphilis, and otosyphilis should be considered in the differential diagnosis of neurologic, ocular, and other signs and symptoms among persons with HIV infection.

#### Treatment

Persons with HIV infection who have early syphilis might be at increased risk for neurologic complications (*623*) and might have higher rates of inadequate serologic response with recommended regimens. The magnitude of these risks is not defined precisely but is likely small. Although long-term (>1 year) comparative data are lacking, no treatment regimens for syphilis have been demonstrated to be more effective in preventing neurosyphilis among persons with HIV infection than the syphilis regimens recommended for persons without HIV (*609*). Careful follow-up after therapy is essential. Using ART per current HIV guidelines might improve clinical outcomes among persons coinfected with HIV and syphilis; concerns regarding adequate treatment of syphilis among persons with HIV infection might not apply to those with HIV virologic suppression (*624*,*625*).

#### Primary and Secondary Syphilis Among Persons with HIV Infection

**Recommended Regimen for Primary and Secondary Syphilis Among Persons with HIV Infection**
**Benzathine penicillin G** 2.4 million units IM in a single dose

Available data demonstrate that additional doses of benzathine penicillin G, amoxicillin, or other antibiotics in primary and secondary syphilis among persons with HIV infection do not result in enhanced efficacy (*592*,*593*,*609*).

##### Other Management Considerations

The majority of persons with HIV infection respond appropriately to the recommended benzathine penicillin G treatment regimen for primary and secondary syphilis (*626*). CSF abnormalities (e.g., mononuclear pleocytosis and elevated protein levels) can be common among persons with HIV, even those without syphilis. The clinical and prognostic significance of such CSF laboratory abnormalities among persons with primary and secondary syphilis who lack neurologic symptoms is unknown. Certain studies have demonstrated that among persons with HIV infection and syphilis, CSF abnormalities are associated with a CD4^+^ T-cell count of ≤350 cells/mL or an RPR titer of ≥1:32 (*614*,*627*). However, CSF examination followed by treatment for neurosyphilis on the basis of laboratory abnormalities has not been associated with improved clinical outcomes in the absence of neurologic signs and symptoms. All persons with HIV infection and primary and secondary syphilis should have a thorough neurologic, ocular, and otic examination (*614*,*622*,*625*). CSF examination should be reserved for those with an abnormal neurologic examination.

##### Follow-Up

Persons with HIV infection and primary or secondary syphilis should be evaluated clinically and serologically for possible treatment failure at 3, 6, 9, 12, and 24 months after therapy; those who meet the criteria for treatment failure (i.e., signs or symptoms that persist or recur or a sustained [>2 weeks] fourfold or greater increase in titer) should be managed in the same manner as persons without HIV infection (i.e., depending on history of sexual activity and on findings of neurologic examination, either repeat treatment with weekly injections of benzathine penicillin G 2.4 million units IM for 3 weeks or CSF examination and repeat treatment guided by CSF findings) (see Primary and Secondary Syphilis). 

In addition, CSF examination and retreatment can be considered for persons whose nontreponemal test titers do not decrease fourfold within 24 months of therapy. If CSF examination is normal, treatment with benzathine penicillin G administered as 2.4 million units IM at weekly intervals for 3 weeks is recommended. Serologic titers might not decrease despite a negative CSF examination and a repeated 3-week course of therapy (*599*). Especially if the initial nontreponemal titer is low (<1:8) in these circumstances, the benefit of additional therapy or repeated CSF examinations is unclear but is not usually recommended. Serologic and clinical monitoring at least annually should continue to monitor for any sustained increases in nontreponemal titer.

##### Management of Sex Partners

See Syphilis, Management of Sex Partners.

##### Special Considerations

###### Penicillin Allergy

Persons with HIV infection who are allergic to penicillin and have primary or secondary syphilis should be managed according to the recommendations for persons without HIV who are allergic to penicillin (see Primary and Secondary Syphilis). Persons with penicillin allergy whose compliance with alternative therapy or follow-up cannot be ensured should be desensitized and treated with penicillin G (see Management of Persons Who Have a History of Penicillin Allergy). Using penicillin alternatives has not been well studied among persons with HIV infection; azithromycin is not recommended for persons with HIV and primary or secondary syphilis infection. Alternative therapies should be used only in conjunction with close serologic and clinical follow-up. Persons with HIV and latent syphilis should be treated similarly to persons who do not have HIV (see Latent Syphilis).

#### Latent Syphilis Among Persons with HIV Infection

**Recommended Regimen for Early Latent Syphilis Among Persons with HIV Infection**
**Benzathine penicillin G** 2.4 million units IM in a single dose
**Recommended Regimen for Late Latent Syphilis or Latent Syphilis of Unknown Duration Among Persons with HIV Infection**
**Benzathine penicillin G** 7.2 million units total, administered as 3 doses of 2.4 million units IM at 1-week intervals

##### Other Management Considerations

All persons with HIV and latent syphilis infection should undergo a thorough neurologic, ocular, and otic examination; those with neurologic symptoms or signs should undergo immediate CSF examination. In the absence of neurologic symptoms or signs, CSF examination has not been associated with improved clinical outcomes and therefore is not recommended. Those with ocular or otic symptoms or signs should be evaluated for ocular syphilis and otosyphilis according to those clinical presentations (see Neurosyphilis, Ocular Syphilis, and Otosyphilis).

##### Follow-Up

Patients with HIV and latent syphilis infection should be evaluated clinically and serologically at 6, 12, 18, and 24 months after therapy. Those persons who meet the criteria for treatment failure (i.e., signs or symptoms that persist or recur or a sustained [>2 weeks] fourfold or greater increase in titer) should be managed in the same manner as persons without HIV (i.e., depending on history of sexual activity and on findings of neurologic examination, either repeat treatment with weekly injections of benzathine penicillin G 2.4 million units IM for 3 weeks or CSF examination and repeat treatment guided by CSF findings) (see Latent Syphilis).

In addition, CSF examination and retreatment can be considered for persons whose nontreponemal test titers do not decrease fourfold within 24 months of therapy. If CSF examination is normal, treatment with benzathine penicillin G administered as 2.4 million units IM at weekly intervals for 3 weeks is recommended. Serologic titers might not decrease despite a negative CSF examination and a repeated 3-week course of therapy (*599*). Especially if the initial nontreponemal titer is low (<1:8) in these circumstances, the benefit of additional therapy or repeated CSF examinations is unclear but is not usually recommended. Serologic and clinical monitoring at least annually should continue to ensure nontreponemal titers remain stable without any sustained titer increases.

##### Management of Sex Partners

See Syphilis, Management of Sex Partners.

##### Special Considerations

###### Penicillin Allergy

The efficacy of alternative nonpenicillin regimens for latent syphilis for persons living with HIV infection has not been well studied, and these therapies should be used only in conjunction with close serologic and clinical follow-up. Patients with penicillin allergy whose compliance with alternative therapy or follow-up cannot be ensured should be desensitized and treated with penicillin G (see Management of Persons Who Have a History of Penicillin Allergy).

#### Neurosyphilis, Ocular Syphilis, and Otic Syphilis Among Persons with HIV Infection

All persons with HIV and syphilis infection should receive a careful neurologic ocular and otic examination. Persons with HIV infection and neurosyphilis should be treated according to the recommendations for persons with neurosyphilis and without HIV infection (see Neurosyphilis, Ocular Syphilis, and Otosyphilis).

##### Follow-Up

Persons with HIV and neurosyphilis infection should be managed according to the recommendations for persons without HIV infection. Serum RPR can be followed for necessary treatment success rather than following CSF parameters (see Neurosyphilis, Ocular Syphilis, and Otosyphilis). Limited data indicate that changes in CSF parameters might occur more slowly among persons with HIV infection, especially those with more advanced immunosuppression (*588*,*624*).

##### Management of Sex Partners

See Syphilis, Management of Sex Partners.

##### Special Considerations

###### Penicillin Allergy

Persons with HIV who are allergic to penicillin and have neurosyphilis infection should be managed according to the recommendations for persons without HIV infection with neurosyphilis who are allergic to penicillin (see Neurosyphilis, Ocular Syphilis, and Otosyphilis). Small observational studies conducted among persons with HIV and neurosyphilis report that ceftriaxone 1–2 g IV daily for 10–14 days might be effective as an alternative agent (*628*–*630*). The possibility of cross-sensitivity between ceftriaxone and penicillin exists; however, the risk for penicillin cross-reactivity between third-generation cephalosporins is negligible (*619*–*621*,*631*) (see Management of Persons Who Have a History of Penicillin Allergy). If concern exists regarding the safety of ceftriaxone for a person with HIV and neurosyphilis, skin testing should be performed to confirm penicillin allergy and, if necessary, penicillin desensitization in consultation with a specialist is recommended. Other regimens have not been adequately evaluated for treatment of neurosyphilis.

### Syphilis During Pregnancy

All women should be screened serologically for syphilis at the first prenatal care visit (*174*), which is mandated by the majority of states (*142*). Among populations for whom receipt of prenatal care is not optimal, serologic screening and treatment (if serologic test is reactive) should be performed at the time of pregnancy testing (*632*). Antepartum screening can be performed by manual nontreponemal antibody testing (e.g., RPR) by using the traditional syphilis screening algorithm or by treponemal antibody testing (e.g., immunoassays) using the reverse sequence algorithm.

Pregnant women with positive treponemal screening tests (e.g., EIA, CIA, or immunoblot) should have additional quantitative nontreponemal testing because titers are essential for monitoring treatment response. Serologic testing should also be performed twice during the third trimester: at 28 weeks’ gestation and at delivery for pregnant women who live in communities with high rates of syphilis and for women who have been at risk for syphilis acquisition during pregnancy.

Maternal risk factors for syphilis during pregnancy include sex with multiple partners, sex in conjunction with drug use or transactional sex, late entry to prenatal care (i.e., first visit during the second trimester or later) or no prenatal care, methamphetamine or heroin use, incarceration of the woman or her partner, and unstable housing or homelessness (*174*,*633*–*636*). Moreover, as part of the management of pregnant women who have syphilis, providers should obtain information concerning ongoing risk behaviors and treatment of sex partners to assess the risk for reinfection.

Any woman who has a fetal death after 20 weeks’ gestation should be tested for syphilis. No mother or neonate should leave the hospital without maternal serologic status having been documented at least once during pregnancy. Any woman who at the time of delivery has no prenatal care history or has been at risk for syphilis acquisition during pregnancy (e.g., misuses drugs; has had another STI during pregnancy; or has had multiple sex partners, a new partner, or a partner with an STI) should have the results of a syphilis serologic test documented before discharge.

#### Diagnostic Considerations

Pregnant women seropositive for syphilis should be considered infected unless an adequate treatment history is clearly documented in the medical records and sequential serologic antibody titers have decreased as recommended for the syphilis stage. The risk for antepartum fetal infection or congenital syphilis at delivery is related to the syphilis stage during pregnancy, with the highest risk occurring during the primary and secondary stages. Quantitative maternal nontreponemal titer, especially if >1:8, might be a marker of early infection and bacteremia. However, risk for fetal infection is still substantial among pregnant women with late latent syphilis and low titers. Pregnant women with stable, serofast low nontreponemal titers who have previously been treated for syphilis might not require additional treatment; however, increasing or high antibody titers in a pregnant woman previously treated might indicate reinfection or treatment failure, and treatment should be offered.

If an automated treponemal test (e.g., EIA or CIA) is used for antepartum syphilis screening, all positive tests should be reflexed to a quantitative nontreponemal test (e.g., RPR or VDRL). If the nontreponemal test is negative, the results are considered discrepant and a second treponemal test (TP-PA is preferred) should be performed, preferably on the same specimen.

If the second treponemal test is positive (e.g., EIA positive, RPR negative, or TP-PA positive), current or previous syphilis infection can be confirmed. For women with a history of adequately treated syphilis who do not have ongoing risk, no further treatment is necessary. Women without a history of treatment should have the syphilis stage determined and should be treated accordingly with a recommended penicillin regimen.

If the second treponemal test is negative (e.g., EIA positive, RPR negative, or TP-PA negative), the positive EIA or CIA is more likely to represent a false-positive test result for women who are living in communities with low rates of syphilis, have a partner who is uninfected, and have no history of treated syphilis (*637*,*638*). If the woman is at low risk for syphilis, lacks signs or symptoms of primary syphilis, has a partner with no clinical or serologic evidence of syphilis, and is likely to follow up with clinical care, repeat serologic testing within 4 weeks can be considered to determine whether the EIA or CIA remains positive or if the RPR, VDRL, or TP-PA result becomes positive. If both the RPR and TP-PA remain negative, no further treatment is necessary. If follow-up is not likely, women with an isolated reactive treponemal test and without a history of treated syphilis should be treated according to the syphilis stage.

#### Treatment

Penicillin G is the only known effective antimicrobial for treating fetal infection and preventing congenital syphilis (*639*). Evidence is insufficient to determine the optimal penicillin regimen during pregnancy (*640*).

**Recommended Regimen for Syphilis During Pregnancy**
Pregnant women should be treated with the recommended penicillin regimen for their stage of infection

#### Other Management Considerations

The following recommendations should be considered for pregnant women with syphilis infection:

- Certain evidence indicates that additional therapy is beneficial for pregnant women to prevent congenital syphilis. For women who have primary, secondary, or early latent syphilis, a second dose of benzathine penicillin G 2.4 million units IM can be administered 1 week after the initial dose (*641*–*643*).
- When syphilis is diagnosed during the second half of pregnancy, management should include a sonographic fetal evaluation for congenital syphilis. However, this evaluation should not delay therapy. Sonographic signs of fetal or placental syphilis (e.g., hepatomegaly, ascites, hydrops, fetal anemia, or a thickened placenta) indicate a greater risk for fetal treatment failure (*644*); cases accompanied by these signs should be managed in consultation with obstetric specialists. A second dose of benzathine penicillin G 2.4 million units IM after the initial dose might be beneficial for fetal treatment in these situations.
- Women treated for syphilis during the second half of pregnancy are at risk for premature labor or fetal distress if the treatment precipitates the Jarisch-Herxheimer reaction (*590*). These women should be advised to seek obstetric attention after treatment if they notice any fever, contractions, or decrease in fetal movements. Stillbirth is a rare complication of treatment; however, concern for this complication should not delay necessary treatment. No data are available to support that corticosteroid treatment alters the risk for treatment-related complications during pregnancy.
- Missed doses >9 days between doses are not acceptable for pregnant women receiving therapy for late latent syphilis (*613*). An optimal interval between doses is 7 days for pregnant women. If a pregnant woman does not return for the next dose on day 7, every effort should be made to contact her and link her to immediate treatment within 2 days to avoid retreatment. Pregnant women who miss a dose of therapy should repeat the full course of therapy.
- All women who have syphilis should be offered testing for HIV at the time of diagnosis.

#### Follow-Up

Coordinated prenatal care and treatment are vital because providers should document that women are adequately treated for the syphilis stage and ensure that the clinical and antibody responses are appropriate for the patient’s disease stage. If syphilis is diagnosed and treated at or before 24 weeks’ gestation, serologic titers should not be repeated before 8 weeks after treatment (e.g., at 32 weeks’ gestation) but should be repeated again at delivery. Titers should be repeated sooner if reinfection or treatment failure is suspected. For syphilis diagnosed and treated after 24 weeks’ gestation, serologic titers should be repeated at delivery.

A majority of women will not achieve a fourfold decrease in titers before delivery, although this does not indicate treatment failure (*645*). However, a fourfold increase in titer after treatment (e.g., from 1:8 to 1:32) that is sustained for >2 weeks is concerning for reinfection or treatment failure. Nontreponemal titers can increase immediately after treatment, presumably related to the treatment response. Therefore, unless symptoms and signs exist of primary or secondary syphilis, follow-up titer should not be repeated until approximately 8 weeks after treatment. Inadequate maternal treatment is likely if delivery occurs within 30 days of therapy, clinical signs of infection are present at delivery, or the maternal antibody titer at delivery is fourfold higher than the pretreatment titer.

#### Management of Sex Partners

See Syphilis, Management of Sex Partners.

#### Special Considerations

##### Penicillin Allergy

No proven alternatives to penicillin are available for treatment of syphilis during pregnancy. Pregnant women who have a history of penicillin allergy should be desensitized and treated with penicillin G. Skin testing or oral graded penicillin dose challenge might be helpful in identifying women at risk for acute allergic reactions (see Management of Persons Who Have a History of Penicillin Allergy).

Tetracycline and doxycycline are to be avoided in the second and third trimesters of pregnancy (*431*). Erythromycin and azithromycin should not be used because neither reliably cures maternal infection nor treats an infected fetus (*640*). Data are insufficient to recommend ceftriaxone or other cephalosporins for treatment of maternal infection and prevention of congenital syphilis (*646*,*647*).

##### HIV Infection

Placental inflammation from congenital syphilis infection might increase the risk for perinatal transmission of HIV. All women with HIV infection should be evaluated for syphilis and receive a penicillin regimen appropriate for the syphilis stage. Data are insufficient to recommend any alternative regimens for pregnant women with syphilis and HIV infection (see Syphilis Among Persons with HIV).

### Congenital Syphilis

The rate of reported congenital syphilis in the United States has increased dramatically since 2012. During 2019, a total of 1,870 cases of congenital syphilis were reported, including 94 stillbirths and 34 infant deaths (*141*). The 2019 national rate of 48.5 cases per 100,000 live births represents a 41% increase relative to 2018 (34.3 cases per 100,000 live births) and a 477% increase relative to 2012 (8.4 cases per 100,000 live births). During 2015–2019, the rate of congenital syphilis increased 291.1% (12.4 to 48.5 per 100,000 live births), which mirrors increases in the rate of primary and secondary syphilis among females aged 15–44 years (a 171.9% increase, from 3.2 to 8.7 per 100,000 females).

Effective prevention and detection of congenital syphilis depend on identifying syphilis among pregnant women and, therefore, on the routine serologic screening of pregnant women during the first prenatal visit and at 28 weeks’ gestation and at delivery for women who live in communities with high rates of syphilis, women with HIV infection, or those who are at increased risk for syphilis acquisition. Certain states have recommended screening three times during pregnancy for all women; clinicians should screen according to their state’s guidelines.

Maternal risk factors for syphilis during pregnancy include sex with multiple partners, sex in conjunction with drug use or transactional sex, late entry to prenatal care (i.e., first visit during the second trimester or later) or no prenatal care, methamphetamine or heroin use, incarceration of the woman or her partner, and unstable housing or homelessness (*174*,*633*–*636*). Moreover, as part of the management of pregnant women who have syphilis, providers should obtain information concerning ongoing risk behaviors and treatment of sex partners to assess the risk for reinfection.

Routine screening of neonatal sera or umbilical cord blood is not recommended because diagnosis at that time does not prevent congenital syphilis in certain newborns. No mother or newborn infant should leave the hospital without maternal serologic status having been documented at least once during pregnancy. Any woman who had no prenatal care before delivery or is considered at increased risk for syphilis acquisition during pregnancy should have the results of a syphilis serologic test documented before she or her neonate is discharged. A quantitative RPR is needed at the time of delivery to compare with the neonate’s nontreponemal test result. If a stat RPR is unavailable and a rapid treponemal test is performed at delivery, the results should be confirmed by using standard syphilis serologic laboratory tests (e.g., RPR and treponemal test) and algorithms.

#### Evaluation and Treatment of Neonates

Diagnosis of congenital syphilis can be difficult because maternal nontreponemal and treponemal immunoglobulin G (IgG) antibodies can be transferred through the placenta to the fetus, complicating the interpretation of reactive serologic tests for syphilis among neonates (infants aged <30 days). Therefore, treatment decisions frequently must be made on the basis of identification of syphilis in the mother; adequacy of maternal treatment; presence of clinical, laboratory, or radiographic evidence of syphilis in the neonate; and comparison of maternal (at delivery) and neonatal nontreponemal serologic titers (e.g., RPR or VDRL) by using the same test, preferably conducted by the same laboratory. Any neonate at risk for congenital syphilis should receive a full evaluation and testing for HIV.

All neonates born to mothers who have reactive nontreponemal and treponemal test results should be evaluated with a quantitative nontreponemal serologic test (RPR or VDRL) performed on the neonate’s serum because umbilical cord blood can become contaminated with maternal blood and yield a false-positive result, and Wharton’s jelly within the umbilical cord can yield a false-negative result. The nontreponemal test performed on the neonate should be the same type of nontreponemal test performed on the mother.

Conducting a treponemal test (e.g., TP-PA, immunoassay-EIA, CIA, or microbead immunoassay) on neonatal serum is not recommended because it is difficult to interpret, as passively transferred maternal antibodies can persist for >15 months. Commercially available IgM tests are not recommended.

All neonates born to women who have reactive nontreponemal serologic tests for syphilis at delivery should be examined thoroughly for evidence of congenital syphilis (e.g., nonimmune hydrops, conjugated or direct hyperbilirubinemia^†^ or cholestatic jaundice or cholestasis, hepatosplenomegaly, rhinitis, skin rash, or pseudoparalysis of an extremity). Pathologic examination of the placenta or umbilical cord using specific staining (e.g., silver) or a *T. pallidum* PCR test using a CLIA-validated test should be considered; direct fluorescence antibody (DFA-TP) reagents are unavailable (*565*). Darkfield microscopic examination or PCR testing of suspicious lesions or body fluids (e.g., bullous rash or nasal discharge) also should be performed. In addition to these tests, for stillborn infants, skeletal survey demonstrating typical osseous lesions might aid in the diagnosis of congenital syphilis because these abnormalities are not detected on fetal ultrasound.

The following scenarios describe the recommended congenital syphilis evaluation and treatment of neonates born to women who had reactive nontreponemal and treponemal serologic tests for syphilis during pregnancy (e.g., RPR reactive, TP-PA reactive or EIA reactive, RPR reactive) and have a reactive nontreponemal test at delivery (e.g., RPR reactive). Maternal history of infection with *T. pallidum* and treatment for syphilis should be considered when evaluating and treating the neonate for congenital syphilis in most scenarios, except when congenital syphilis is proven or highly probable.

##### Scenario 1: Confirmed Proven or Highly Probable Congenital Syphilis

Any neonate with

- an abnormal physical examination that is consistent with congenital syphilis;
- a serum quantitative nontreponemal serologic titer that is fourfold^§^ (or greater) higher than the mother’s titer at delivery (e.g., maternal titer = 1:2, neonatal titer ≥1:8 or maternal titer = 1:8, neonatal titer ≥1:32)^¶^; or
- a positive darkfield test or PCR of placenta, cord, lesions, or body fluids or a positive silver stain of the placenta or cord.

###### Recommended Evaluation

- CSF analysis for VDRL, cell count, and protein**
- Complete blood count (CBC) and differential and platelet count
- Long-bone radiographs
- Other tests as clinically indicated (e.g., chest radiograph, liver function tests, neuroimaging, ophthalmologic examination, and auditory brain stem response)
**Recommended Regimens, Confirmed or Highly Probable Congenital Syphilis**
  **Aqueous crystalline penicillin G** 100,000–150,000 units/kg body weight/day, administered as 50,000 units/kg body weight/dose IV every 12 hours during the first 7 days of life and every 8 hours thereafter for a total of 10 days
*or*
  **Procaine penicillin G** 50,000 units/kg body weight/dose IM in a single daily dose for 10 days

If >1 day of therapy is missed, the entire course should be restarted. Data are insufficient regarding use of other antimicrobial agents (e.g., ampicillin). When possible, a full 10-day course of penicillin is preferred, even if ampicillin was initially provided for possible sepsis (*648*–*650*). Using agents other than penicillin requires close serologic follow-up for assessing therapy adequacy.

##### Scenario 2: Possible Congenital Syphilis

Any neonate who has a normal physical examination and a serum quantitative nontreponemal serologic titer equal to or less than fourfold of the maternal titer at delivery (e.g., maternal titer = 1:8, neonatal titer ≤1:16) and one of the following:

- The mother was not treated, was inadequately treated, or has no documentation of having received treatment.
- The mother was treated with erythromycin or a regimen other than those recommended in these guidelines (i.e., a nonpenicillin G regimen).^††^
- The mother received the recommended regimen but treatment was initiated <30 days before delivery.

###### Recommended Evaluation

- CSF analysis for VDRL, cell count, and protein**
- CBC, differential, and platelet count
- Long-bone radiographs

This evaluation is not necessary if a 10-day course of parenteral therapy is administered, although such evaluations might be useful. For instance, a lumbar puncture might document CSF abnormalities that would prompt close follow-up. Other tests (e.g., CBC, platelet count, and long-bone radiographs) can be performed to further support a diagnosis of congenital syphilis.

**Recommended Regimens, Possible Congenital Syphilis**
**Aqueous crystalline penicillin G** 100,000–150,000 units/kg body weight/day, administered as 50,000 units/kg body weight/dose IV every 12 hours during the first 7 days of life and every 8 hours thereafter for a total of 10 days
*or*
**Procaine penicillin G** 50,000 units/kg body weight/dose IM in a single daily dose for 10 days
*or*
**Benzathine penicillin G** 50,000 units/kg body weight/dose IM in a single dose

Before using the single-dose benzathine penicillin G regimen, the recommended evaluation (i.e., CSF examination, long-bone radiographs, and CBC with platelets) should be normal, and follow-up should be certain. If any part of the neonate’s evaluation is abnormal or not performed, if the CSF analysis is uninterpretable because of contamination with blood, or if follow-up is uncertain, a 10-day course of penicillin G is required.

If the neonate’s nontreponemal test is nonreactive and the provider determines that the mother’s risk for untreated syphilis is low, treatment of the neonate with a single IM dose of benzathine penicillin G 50,000 units/kg body weight for possible incubating syphilis can be considered without an evaluation. Neonates born to mothers with untreated early syphilis at the time of delivery are at increased risk for congenital syphilis, and the 10-day course of penicillin G should be considered even if the neonate’s nontreponemal test is nonreactive, the complete evaluation is normal, and follow-up is certain.

##### Scenario 3: Congenital Syphilis Less Likely

Any neonate who has a normal physical examination and a serum quantitative nontreponemal serologic titer equal or less than fourfold of the maternal titer at delivery (e.g., maternal titer = 1:8, neonatal titer ≤1:16) and both of the following are true:

- The mother was treated during pregnancy, treatment was appropriate for the infection stage, and the treatment regimen was initiated ≥30 days before delivery.
- The mother has no evidence of reinfection or relapse.

###### Recommended Evaluation

No evaluation is recommended.

**Recommended Regimen, Congenital Syphilis Less Likely**
**Benzathine penicillin G** 50,000 units/kg body weight/dose IM in a single dose*
* Another approach involves not treating the newborn if follow-up is certain but providing close serologic follow-up every 2–3 months for 6 months for infants whose mothers’ nontreponemal titers decreased at least fourfold after therapy for early syphilis or remained stable for low-titer, latent syphilis (e.g., VDRL <1:2 or RPR <1:4).

##### Scenario 4: Congenital Syphilis Unlikely

Any neonate who has a normal physical examination and a serum quantitative nontreponemal serologic titer equal to or less than fourfold of the maternal titer at delivery^§^ and both of the following are true:

- The mother’s treatment was adequate before pregnancy.
- The mother’s nontreponemal serologic titer remained low and stable (i.e., serofast) before and during pregnancy and at delivery (e.g., VDRL ≤1:2 or RPR ≤1:4).

###### Recommended Evaluation

No evaluation is recommended.

**Recommended Regimen, Congenital Syphilis Unlikely**
No treatment is required. However, any neonate with reactive nontreponemal tests should be followed serologically to ensure the nontreponemal test returns to negative (see Follow-Up). Benzathine penicillin G 50,000 units/kg body weight as a single IM injection might be considered, particularly if follow-up is uncertain and the neonate has a reactive nontreponemal test.

The following situations describe management of neonates born to women screened during pregnancy by using the reverse sequence algorithm with reactive treponemal serologic tests and a nonreactive nontreponemal serologic test.

**Reactive maternal treponemal serologies with a nonreactive nontreponemal serology (e.g., EIA reactive, RPR nonreactive, or TP-PA reactive) during pregnancy.** Syphilis is highly unlikely for neonates born to mothers with a nonreactive nontreponemal test after adequate treatment for syphilis during pregnancy or documentation of adequate treatment before pregnancy (with no evidence of reinfection of relapse). If testing is performed again at delivery and 1) the maternal nontreponemal test remains nonreactive and 2) the neonate has a normal physical examination and nonreactive nontreponemal test (e.g., RPR nonreactive), the provider should consider managing similarly to Scenario 4 without a laboratory evaluation and with no treatment required. Benzathine penicillin G 50,000 units/kg body weight as a single IM injection might be considered if syphilis exposure is possible within 1 month of delivery and follow-up of the mother and infant is uncertain.

**Isolated reactive maternal treponemal serology (e.g., EIA reactive, RPR nonreactive, or TP-PA nonreactive) during pregnancy.** Syphilis is unlikely for neonates born to mothers screened with the reverse sequence algorithm with isolated reactive maternal treponemal serology. Among low-prevalence populations, these are likely false-positive results and might become nonreactive with repeat testing (*638*). If these neonates have a normal physical examination and the risk for syphilis is low in the mother, no evaluation and treatment are recommended for the neonate. If syphilis exposure is possible or unknown in the mother or the mother desires further evaluation to definitively rule out syphilis, repeat serology within 4 weeks is recommended to evaluate for early infection (see Syphilis During Pregnancy).

**Isolated reactive maternal treponemal serology (e.g., rapid treponemal test) at delivery.** For mothers with late or no prenatal care with a reactive rapid treponemal test at delivery, confirmatory laboratory-based testing should be performed; however, results should not delay evaluation and treatment of the neonate. These neonates should be evaluated and treated with a 10-day course of penicillin as recommended in Scenario 1, and consultation with a specialist is recommended.

#### Follow-Up

All neonates with reactive nontreponemal tests should receive thorough follow-up examinations and serologic testing (i.e., RPR or VDRL) every 2–3 months until the test becomes nonreactive.

For a neonate who was not treated because congenital syphilis was considered less likely or unlikely, nontreponemal antibody titers should decrease by age 3 months and be nonreactive by age 6 months, indicating that the reactive test result was caused by passive transfer of maternal IgG antibody. At age 6 months, if the nontreponemal test is nonreactive, no further evaluation or treatment is needed; if the nontreponemal test is still reactive, the infant is likely infected and should be treated.

Treated neonates who exhibit persistent nontreponemal test titers by age 6–12 months should be reevaluated through CSF examination and managed in consultation with an expert. Retreatment with a 10-day course of a penicillin G regimen might be indicated.

Neonates with a negative nontreponemal test at birth and whose mothers were seroreactive at delivery should be retested at age 3 months to rule out serologically negative incubating congenital syphilis at the time of birth. Treponemal tests should not be used to evaluate treatment response because the results are qualitative, and passive transfer of maternal IgG treponemal antibody might persist for >15 months.

Neonates whose initial CSF evaluations are abnormal do not need repeat lumbar puncture unless they exhibit persistent nontreponemal serologic test titers at age 6–12 months. Persistent nontreponemal titers and CSF abnormalities should be managed in consultation with an expert.

#### Special Considerations

##### Penicillin Allergy

Neonates who require treatment for congenital syphilis but who have a history of penicillin allergy or develop an allergic reaction presumed secondary to penicillin should be desensitized and then treated with penicillin G (see Management of Persons Who Have a History of Penicillin Allergy). Skin testing remains unavailable for neonates because the procedure has not been standardized for this age group. Data are insufficient regarding use of other antimicrobial agents (e.g., ceftriaxone) for congenital syphilis among neonates. If a nonpenicillin G agent is used, close clinical and serologic follow-up is required in consultation with an expert. Repeat CSF examination should be performed if the initial CSF examination was abnormal.

##### Penicillin Shortage

During periods when the availability of aqueous crystalline penicillin G is compromised, the following is recommended (https://www.cdc.gov/std/treatment/drug-notices.htm):

- For neonates with clinical evidence of congenital syphilis (see Scenario 1), check local sources for aqueous crystalline penicillin G (potassium or sodium) and notify CDC and FDA of limited supply. If IV penicillin G is limited, substitute some or all daily doses with procaine penicillin G (50,000 units/kg body weight/dose IM/day in a single daily dose for 10 days).
- If aqueous or procaine penicillin G is unavailable, ceftriaxone (50–75 mg/kg body weight/day IV every 24 hours) can be considered with thorough clinical and serologic follow-up and in consultation with an expert because evidence is insufficient to support using ceftriaxone for treating congenital syphilis. Ceftriaxone should be used with caution in neonates with jaundice.
- For neonates without any clinical evidence of congenital syphilis (see Scenario 2 and Scenario 3), use
  º procaine penicillin G 50,000 units/kg body weight/dose/day IM in a single dose for 10 days, or
  º benzathine penicillin G 50,000 units/kg body weight IM as a single dose.
- If any part of the evaluation for congenital syphilis is abnormal or was not performed, CSF examination is not interpretable, or follow-up is uncertain, procaine penicillin G is recommended. A single dose of ceftriaxone is inadequate therapy.
- For premature neonates who have no clinical evidence of congenital syphilis (see Scenario 2 and Scenario 3) and might not tolerate IM injections because of decreased muscle mass, IV ceftriaxone can be considered with thorough clinical and serologic follow-up and in consultation with an expert. Ceftriaxone dosing should be adjusted according to birthweight.

##### HIV Infection

Evidence is insufficient to determine whether neonates who have congenital syphilis and HIV infection or whose mothers have HIV require different therapy or clinical management than is recommended for all neonates. All neonates with congenital syphilis should be managed similarly, regardless of HIV status.

#### Evaluation and Treatment of Infants and Children with Congenital Syphilis

Infants and children aged ≥1 month who are identified as having reactive serologic tests for syphilis (e.g., RPR reactive, TP-PA reactive or EIA reactive, RPR reactive) should be examined thoroughly and have maternal serology and records reviewed to assess whether they have congenital or acquired syphilis (see Primary and Secondary Syphilis; Latent Syphilis; Sexual Assault or Abuse of Children). In the case of extremely early or incubating syphilis at the time of delivery, all maternal serologic tests might have been negative; thus, infection might be undetected until a diagnosis is made later in the infant or child. Any infant or child at risk for congenital syphilis should receive a full evaluation and testing for HIV infection.

International adoptee, immigrant, or refugee children from countries where treponemal infections (e.g., yaws or pinta) are endemic might have reactive nontreponemal and treponemal serologic tests, which cannot distinguish between syphilis and other subspecies of *T. pallidum* (*651*). These children might also have syphilis (*T. pallidum *subspecies *pallidum*) and should be evaluated for congenital syphilis.

##### Recommended Evaluation

The following evaluations should be performed:

- CSF analysis for VDRL, cell count, and protein
- CBC, differential, and platelet count
- Other tests as clinically indicated (e.g., long-bone radiographs, chest radiograph, liver function tests, abdominal ultrasound, ophthalmologic examination, neuroimaging, and auditory brain-stem response)
**Recommended Regimen for Congenital Syphilis Among Infants and Children**
  **Aqueous crystalline penicillin G** 200,000–300,000 units/kg body weight/day IV, administered as 50,000 units/kg body weight every 4–6 hours for 10 days

If the infant or child has no clinical manifestations of congenital syphilis and the evaluation (including the CSF examination) is normal, treatment with <3 weekly doses of benzathine penicillin G 50,000 units/kg body weight IM can be considered. A single dose of benzathine penicillin G 50,000 units/kg body weight IM up to the adult dose of 2.4 million units in a single dose can be considered after the 10-day course of IV aqueous penicillin G to provide more comparable duration for treatment in those who have no clinical manifestations and normal CSF. All of these treatment regimens should also be adequate for children who might have other treponemal infections.

##### Follow-Up

Thorough follow-up examinations and serologic testing (i.e., RPR or VDRL) of infants and children treated for congenital syphilis after the neonatal period (aged >30 days) should be performed every 3 months until the test becomes nonreactive or the titer has decreased fourfold. The serologic response after therapy might be slower for infants and children than neonates. If these titers increase at any point >2 weeks or do not decrease fourfold after 12–18 months, the infant or child should be evaluated (e.g., CSF examination), treated with a 10-day course of parenteral penicillin G, and managed in consultation with an expert. Treponemal tests (e.g., EIA, CIA, or TP-PA) should not be used to evaluate treatment response because the results are qualitative and persist after treatment, and passive transfer of maternal IgG treponemal antibody might persist for >15 months after delivery. Infants or children whose initial CSF evaluations are abnormal do not need repeat lumbar puncture unless their serologic titers do not decrease fourfold after 12–18 months. After 18 months of follow-up, abnormal CSF indices that persist and cannot be attributed to other ongoing illness indicate that retreatment is needed for possible neurosyphilis and should be managed in consultation with an expert.

##### Special Considerations

###### Penicillin Allergy

Infants and children who require treatment for congenital syphilis but who have a history of penicillin allergy or develop an allergic reaction presumed secondary to penicillin should be desensitized and treated with penicillin G (see Management of Persons Who Have a History of Penicillin Allergy). Skin testing remains unavailable for infants and children because the procedure has not been standardized for this age group. Data are insufficient regarding use of other antimicrobial agents (e.g., ceftriaxone) for congenital syphilis among infants and children. If a nonpenicillin G agent is used, close clinical, serologic, and CSF follow-up is required in consultation with an expert.

###### Penicillin Shortage

During periods when availability of penicillin G is compromised, management options are similar to options for the neonate (see Evaluation and Treatment of Neonates).

- For infants and children with clinical evidence of congenital syphilis, if IV penicillin is limited after checking local sources and notifying CDC and FDA about limited supplies, procaine penicillin G (50,000 units/kg body weight/dose IM up to the adult dose of 2.4 million units a day in a single daily dose for 10 days) is recommended.
- If procaine penicillin G is not available, ceftriaxone (in doses for age and weight) can be considered with thorough clinical and serologic follow-up. Infants and children receiving ceftriaxone should be managed in consultation with an expert because evidence is insufficient to support use of ceftriaxone for treatment of congenital syphilis among infants or children. For infants aged ≥30 days, use ceftriaxone 75 mg/kg body weight/day IV or IM in a single daily dose for 10–14 days (dose adjustment might be necessary on the basis of current weight). For children, ceftriaxone 100 mg/kg body weight/day in a single daily dose is recommended.
- For infants and children without any clinical evidence of infection (see Scenario 2 and Scenario 3), use
  º procaine penicillin G 50,000 units/kg body weight/dose IM up to the adult dose of 2.4 million units a day in a single dose for 10 days, or
  º benzathine penicillin G 50,000 units/kg body weight IM up to the adult dose of 2.4 million units as a single dose.
- If any part of the evaluation for congenital syphilis is abnormal or not performed, CSF examination is not interpretable, or follow-up is uncertain, procaine penicillin G is recommended. In these scenarios, a single dose of ceftriaxone is inadequate therapy.

###### HIV Infection

Evidence is insufficient to determine whether infants and children who have congenital syphilis and HIV infection or whose mothers have HIV require different therapy or clinical management than what is recommended for all infants and children. All infants and children with congenital syphilis should be managed similarly, regardless of HIV status.

## Management of Persons Who Have a History of Penicillin Allergy

Penicillin and other ß-lactam antibiotics have a crucial role in treating STIs. Penicillin is recommended for all clinical stages of syphilis, and no proven alternatives exist for treating neurosyphilis, congenital syphilis, or syphilis during pregnancy. Ceftriaxone, a third-generation cephalosporin, is recommended for gonorrhea treatment. For extragenital site infections, especially pharyngeal, failure rates of nonceftriaxone regimens can be substantial. In most clinical settings, patients who report a penicillin allergy are not treated with ß-lactam antimicrobials. For patients with a diagnosis of gonorrhea and a concomitant reported allergy to penicillin, ceftriaxone is often avoided, even though the cross-reactivity between penicillin allergy and third-generation cephalosporins is low (*652*–*654*).

Prevalence of reported allergy to penicillin is approximately 10% among the U.S. population and higher among hospital inpatients and residents in health care–related facilities (*655*–*658*). One large study in an STI clinic revealed that 8.3% of patients reported penicillin or another ß-lactam antibiotic allergy (*659*). Penicillin allergy is often overreported, with the majority of patients who report penicillin allergy able to tolerate the medication (*660*). The prevalence of reported penicillin allergy in low-income countries is unknown; however, limited data indicate that penicillin is one of the most frequently reported antibiotic allergies (*661*).

Patients often are incorrectly labeled as allergic to penicillin and are therefore denied the benefit of a ß-lactam therapy. The presence of a penicillin allergy label considerably reduces prescribing options for affected patients. Moreover, penicillin allergy labels lead to the use of more expensive and less effective drugs and can result in adverse consequences, including longer length of hospital stay and increased risk for infection. Multiple studies have described that persons with reported penicillin or another ß-lactam antibiotic allergy have higher rates of surgical-site infections, methicillin-resistant *Staphylococcus aureus* infections, and higher medical care usage (*653*,*662*–*664*).

The overreported prevalence of penicillin allergy is secondary to imprecise use of the term “allergy” by families and clinicians and lack of clarity to differentiate between immunoglobulin E (IgE)-mediated hypersensitivity reactions, drug intolerances, and other idiosyncratic reactions that can occur days after exposure. Approximately 80% of patients with a true IgE-mediated allergic reaction to penicillin have lost the sensitivity after 10 years (*658*). Thus, patients with recent reactions are more likely to be allergic than patients with remote reactions, and patients who had allergic reactions in the distant past might no longer be reactive.

In a Baltimore, Maryland, STI clinic study, only 7.1% of the patients who reported allergy to penicillin or to another ß-lactam antibiotic had an objective positive test for penicillin allergy (*659*). Moreover, in studies that have incorporated penicillin skin testing and graded oral challenge among persons with reported penicillin allergy, the true rates of allergy are low, ranging from 1.5% to 6.1% (*665*–*667*). Studies in preoperative surgical patients with reported penicillin allergy, evaluated for cardiovascular surgery (*668*) or orthopedics (*669*), have rates of skin test positivity <8.5%. However, when patients with high-risk penicillin allergy histories are excluded, 99% of patients could receive ß-lactams. In hospitalized patients and other populations with comorbidities, the typical rates of validated penicillin allergy among patients who report a history of penicillin allergy are 2.5%–9.0% (*670*–*673*).

### Cross-Reactivity with Cephalosporins

Penicillin and cephalosporins both contain a ß-lactam ring. This structural similarity has led to considerable confusion regarding cross-reactivity of these drugs and the risks for allergic reactions from cephalosporins among penicillin-allergic patients. In most clinical settings, patients with reported penicillin allergy are precluded from treatment with such cephalosporin antibiotics as ceftriaxone. Third-generation cephalosporins (e.g., ceftriaxone and cefixime) have lower cross-reactivity with IgE-mediated penicillin-allergic patients (<1%) compared with first- and second-generation cephalosporins (range: 1%–8%). Moreover, anaphylaxis secondary to cephalosporins is extremely rare among persons who report a penicillin allergy and is estimated to occur at a rate of one per 52,000 persons (*652*). Data from the Kaiser health care system reported that among 3,313 patients with self-reported cephalosporin allergy who received a cephalosporin (mostly first generation), no cases of anaphylaxis were reported (*652*). Use of third- and fourth-generation cephalosporins and carbapenems is safe for patients without a history of any IgE-mediated symptoms (e.g., anaphylaxis or urticaria) from penicillin during the preceding 10 years.

### Validating Penicillin or Another ß-Lactam Antibiotic Allergy

Evaluating a patient who reports a penicillin or another ß-lactam antibiotic allergy involves three steps: 1) obtaining a thorough medical history, including previous exposures to penicillin or other ß-lactam antibiotics (*658*); 2) performing a skin test evaluation by using the penicillin major and minor determinants; and 3) among those who have a negative penicillin skin test, performing an observed oral challenge with 250 mg amoxicillin before proceeding directly to treatment with the indicated ß-lactam therapy (*667*,*675*).

For persons who have a positive skin test reactive to penicillin (either to the major or minor determinants), treatment with a ß-lactam antibiotic is not usually advised, and other effective antimicrobials should be used (*656*,*658*). For persons among whom the only therapy option is a penicillin antibiotic (e.g., a patient with neurosyphilis or a pregnant woman with syphilis) and among whom a penicillin skin test is positive, induction of penicillin tolerance (also referred to as desensitization) is required (*675*). Desensitization protocols to penicillin should be performed by allergists, and they require a monitored inpatient environment.

#### Penicillin Skin Testing

Penicillin skin testing with a major determinant analog (penicilloyl-polylysine) and minor determinants (benzylpenicilloate, benzylpenilloate, or benzylpenicillin isomers of penicillin) are used for skin test evaluation for IgE-dependent penicillin allergy and can reliably identify persons at high risk for IgE-mediated reactions to penicillin (*658*,*660*,*676*). Until recently, penicillin skin testing in the United States only included the major determinant benzyl penicillin poly-L-lysine (Pre-Pen) in addition to penicillin G. This test identifies approximately 90%–99% of the IgE-mediated penicillin-allergic patients. Because the remaining 1%–10% of penicillin-allergic patients who are not captured by this penicillin skin test are due to minor determinants IgE antibodies, the standard practice is to follow skin testing with an observed oral challenge of amoxicillin 250 mg with 1 hour of observation. If the skin test and oral challenge are both negative, the risk for IgE-mediated anaphylaxis approaches zero and is equivalent to that of a person who has never reported an allergy to penicillin.

A revised version of the penicillin skin test kit, which includes the major determinant reagent Pre-Pen, minor determinants, and amoxicillin, is being evaluated by FDA. This penicillin skin test kit has been evaluated among 455 patients (*677*) with previous allergy history and has a negative predictive value of 98%. If approved, this kit might eliminate the need for oral challenge.

Penicillin skin testing has become a clinically significant element in antibiotic stewardship programs, and the procedure has been increasingly used by hospital-based pharmacists, hospitalists, and infectious disease physicians (*670*,*672*,*673*,*678*,*679*) as part of overall antibiotic stewardship interventions. When integrated into stewardship, the rates of ß-lactam antibiotic use increased substantially (*670*).

#### Recommendations

Persons with a history of severe adverse cutaneous reaction (e.g., Stevens-Johnson syndrome or toxic epidermal necrolysis) and other severe non–IgE-mediated reactions (e.g., interstitial nephritis or hemolytic anemia) are not candidates for penicillin skin testing or challenge. Penicillin and any other ß-lactam antibiotics should be avoided indefinitely among these patients, who should be referred to an allergy center for further evaluation. Similarly, patients who deny penicillin allergy, but who report previous IgE-type reactions to cephalosporins, should be referred to an allergist for specific cephalosporin testing.

In a time of increasing antimicrobial resistance, following recommended use of antibiotic treatments is crucial. STI programs and clinicians should promote increased access to penicillin allergy testing. Allergy testing is being provided by clinicians in primary care and hospital settings. If appropriate, STI programs and ambulatory settings should consider developing expanded access to penicillin or ß-lactam allergy assessment.

Persons with high-risk symptom histories (e.g., anaphylaxis within the previous 10 years) should not be administered penicillin or a ß-lactam antibiotic in an ambulatory setting. Furthermore, these persons with high-risk symptoms should not receive penicillin skin testing or amoxicillin oral challenge in an ambulatory STI setting and should be referred to an allergist for further evaluation.

High-risk symptom histories include development of the following after penicillin or ß-lactam administration: anaphylaxis within 6 hours or severe adverse cutaneous reaction (e.g., eosinophilia and systemic symptoms, Stevens-Johnson syndrome, toxic epidermal necrolysis, or acute generalized exanthematous pustulosis) and other severe non–IgE-mediated reactions (e.g., kidney or hepatic injury, hemolytic anemia, or thrombocytopenia).

#### Direct Treatment Approach for Ceftriaxone

Among persons with confirmed IgE-mediated penicillin allergy, the level of cross-reactivity with third-generation cephalosporins is low (*652*,*680*,*681*). If a patient has a low-risk history for an IgE-mediated penicillin allergy, ambulatory settings often treat with third-generation cephalosporins without further testing. Low-risk history includes one nonspecific symptom (e.g., gastrointestinal intolerance, headache, fatigue, or nonurticarial rash) (Box 2). In addition, a family history of penicillin or ß-lactam allergy alone is not a contraindication for treatment with ß-lactam antibiotics. This practice is increasingly being used in ambulatory settings and for preoperative prophylaxis (*658*,*663*,*680*,*682*–*684*).

BOX 2 **Low-risk history in patients who report penicillin allergy**

Gastrointestinal symptoms

Headache

Pruritis without rash

Localized rash

Delayed onset rash (>24 hours)

Symptoms unknown

Family history of penicillin or another drug allergy

Patient denies allergy but it is on the medical record

#### Patients at Low Risk for Oral Challenge

If the patient gives only a low-risk history of IgE-mediated penicillin allergy that includes symptoms such gastrointestinal intolerance, headache, fatigue, or nonspecific pruritus, or gives a family history only, an oral challenge can be administered to document the absence of allergy (Box 2). If the reaction occurred in the distant past (>10 years), the likelihood is reduced even further (*653*,*658*,*663*,*682*,*683*,*685*,*686*). The risk for severe amoxicillin-mediated anaphylaxis has decreased over time and is rare. In the United Kingdom during 1972–2007, one fatal case of amoxicillin-medicated anaphylaxis was reported (*684*).

###  Skin Testing for Penicillin Allergy

Skin testing for penicillin allergy should be performed if any indication exists that the symptoms were secondary to an IgE-mediated hypersensitivity. Testing is also indicated as a potential diagnostic procedure to definitively rule out penicillin allergy and document a negative allergy status in the medical record (i.e., delabeling). Because penicillin allergy testing does not test for multiple minor determinants, a person with a negative skin test should follow up with an oral challenge to confirm the negative status.

Persons with negative results of a penicillin skin test, followed by an amoxicillin oral challenge, can receive conventional penicillin therapy safely if needed. Persons with positive skin test results and for whom no other clinical options exist (e.g., neurosyphilis and syphilis in a pregnant woman) should be referred to an allergist and desensitized before initiating treatment.

#### Testing Procedures

Penicillin skin testing includes use of skin test reagents for identifying persons at risk for adverse reactions (Box 3), followed by initial pinprick screening with penicillin major determinants (Pre-Pen) and penicillin G, followed by intradermal testing if pinprick results are negative. Penicillin testing procedures are performed in accordance with the Pre-Pen test kit instructions (https://penallergytest.com/wp-content/uploads/PRE-PEN-Package-Insert.pdf). Saline negative controls and histamine positive controls are an integral part of the procedure. Penicillin skin testing should not be performed for patients who have taken antihistamines within the past 7 days.

BOX 3 **Skin test reagents for identifying persons at risk for adverse reactions to penicillin**

**Major determinant**

- Benzylpenicilloyl polylysine injection (Pre-Pen) (AllerQuest) (6 × 10^-5^M)

**Minor determinant precursors**

- Benzylpenicillin G (10^-2^M, 3.3 mg/mL, 10,000 units/mL)
- Benzylpenicilloate (10^-2^M, 3.3 mg/mL)
- Benzylpenicilloate (or penicilloyl propylamine) (10^-2^M, 3.3 mg/mL)

Aged penicillin is not an adequate source of minor determinants. Penicillin G should either be freshly prepared or come from a fresh-frozen source.

**Positive control**

- Commercial histamine for scratch testing (1.0 mg/mL)

**Negative control**

- Diluent (usually saline) or allergen diluent

**Source:** Adapted from Saxon A, Beall GN, Rohr AS, Adelman DC. Immediate hypersensitivity reactions to beta-lactam antibiotics. Ann Intern Med 1987;107:204−15.

Skin testing can be safely performed by trained nonallergists and has been implemented as an antimicrobial stewardship intervention by internal medicine physicians, pharmacists, hospitalists, and infectious disease physicians (*670*,*673*,*678*,*679*). Patients tested should also receive documentation of status, and the results should be entered in the medical record.

Penicillin skin testing during pregnancy is considered safe. For pregnant persons who report a penicillin or ß-lactam allergy, penicillin allergy is an important consideration in treating syphilis during pregnancy and the potential for group B streptococcal infection and preoperative prophylaxis if a cesarean delivery is required. However, oral challenges should not be performed unless in a setting where additional support services are available.

#### Managing Persons Being Tested

Patients who have a positive skin test should not receive ß-lactam drugs in the ambulatory setting and should be referred to an allergist or penicillin allergy expert for further evaluation. The allergy testing results should be documented in the medical record. Patients who test negative should be informed that their risk for anaphylaxis is extremely low and is equivalent to a person who does not report an allergy history. If treatment with penicillin or ceftriaxone is indicated, it can be administered safely. Documentation of testing results should be provided to the patient.

#### Desensitization

Desensitization is required for persons who have a documented penicillin allergy and for whom no therapeutic alternatives exist (e.g., syphilis during pregnancy and persons with neurosyphilis). Modified protocols might be considered on the basis of the clinical syndrome, drug of choice, and route of administration (*687*–*690*). Patients might require referral to a specialty center where desensitization can be performed.

#### Allergy Referral Resources

With increased access to skin testing kits and the need to better target therapy for gonorrhea and syphilis, programs should identify local allergy consultant resources.

## Diseases Characterized by Urethritis and Cervicitis

### Urethritis

Urethritis, as characterized by urethral inflammation, can result from either infectious or noninfectious conditions. Symptoms, if present, include dysuria, urethral pruritis, and mucoid, mucopurulent, or purulent discharge. Signs of urethral discharge on examination can also be present among persons without symptoms. Although *N. gonorrhoeae* and *C. trachomatis* are well established as clinically important infectious causes of urethritis, *M. genitalium* has been strongly associated with urethritis and, less commonly, prostatitis (*691*–*697*). If POC diagnostic tools (e.g., Gram, methylene blue [MB], or gentian violet [GV] stain microscopy) are unavailable, drug regimens effective against both gonorrhea and chlamydia should be administered. Further testing to determine the specific etiology is recommended for preventing complications, reinfection, and transmission because a specific diagnosis might improve treatment compliance, delivery of risk-reduction interventions, and partner services. Both chlamydia and gonorrhea are reportable to health departments. NAATs are preferred for detecting *C. trachomatis* and *N. gonorrhoeae*, and urine is the preferred specimen for males (*553*). NAAT-based tests for diagnosing *T. vaginalis* among men with urethritis have not been cleared by FDA; however, laboratories have performed the CLIA-compliant validation studies (*698*) needed to provide such testing.

#### Etiology

Multiple organisms can cause infectious urethritis. The presence of gram-negative intracellular diplococci (GNID) or purple intracellular diplococci (MB or GV) on urethral smear is indicative of presumed gonococcal infection, which is frequently accompanied by chlamydial infection. Nongonococcal urethritis (NGU), which is diagnosed when microscopy of urethral secretions indicate inflammation without GNID or MB or GV purple intracellular diplococci, is caused by *C. trachomatis* in 15%–40% of cases; however, prevalence varies by age group, with a lower proportion of disease occurring among older men (*699*). Documentation of chlamydial infection as NGU etiology is essential because of the need for partner referral for evaluation and treatment to prevent complications of chlamydia, especially for female partners. Complications of *C. trachomatis*–associated NGU among males include epididymitis, prostatitis, and reactive arthritis.

*M. genitalium* is associated with symptoms of urethritis and urethral inflammation and accounts for 15%–25% of NGU cases in the United States (*691*–*693*,*696*,*697*,*700*). Among men with symptoms of urethritis, *M. genitalium* was detected in 11% of those with urethritis in Australia (*701*), 12%–15% in the United Kingdom (*702*–*704*), 15% in South Africa (*696*), 19% in China (*705*), 21% in Korea, 22% in Japan (*706*), and 28.7% in the United States (range: 20.4%–38.8%) (*697*). Data are inconsistent regarding other *Mycoplasma* and *Ureaplasma* species as etiologic agents of urethritis (*707*). The majority of men with *Ureaplasma* infections do not have overt disease unless a high organism load is present.

*T. vaginalis* can cause urethritis among heterosexual men; however, the prevalence varies substantially by U.S. geographic region, age, and sexual behavior and within specific populations. Studies among men with and without overt urethritis in developed countries document relatively low rates of *T. vaginalis* in the Netherlands (0.5%) (*708*), Japan (1.3%) (*706*,*709*), the United States (2.4%) (*710*), and the United Kingdom (3.6%) (*703*). Studies in other countries have documented higher rates, such as in Croatia (8.2%) (*711*) and Zimbabwe (8.4%) (*712*), particularly among symptomatic patients.

*Neisseria meningitidis* can colonize mucosal surfaces and cause urethritis (*713*). Urogenital *N. meningitidis* rates and duration of carriage, prevalence of asymptomatic and symptomatic infection, and modes of transmission have not been systematically described; however, studies indicate that *N. meningitidis* can be transmitted through oral-penile contact (i.e., fellatio) (*714*–*716*). *N. meningitidis* has similar colony morphology appearance on culture and cannot be distinguished from *N. gonorrhoeae* on Gram stain. Identification of *N. meningitidis* as the etiologic agent with presumed gonococcal urethritis on the basis of Gram stain but negative NAAT for gonorrhea requires a confirmation by culture. Meningococcal urethritis is treated with the same antimicrobial regimens as gonococcal urethritis. Although evidence is limited regarding the risk for sexual transmission or recurrent infections with meningococcal urethritis, treatment of sex partners of patients with meningococcal urethritis with the same antimicrobial regimens as for exposure to gonococcal infection can be considered. No indication exists for treating persons with *N. meningitidis* identified in their oropharynx when not also associated with symptomatic urethritis.

In other instances, NGU can be caused by HSV, Epstein-Barr virus, and adenovirus (*699*) acquired by fellatio (i.e., oral-penile contact). In a retrospective review of 80 cases of HSV urethritis in Australia (*717*), the majority of infections were associated with HSV-1 with clinical findings of meatitis (62%), genital ulceration (37%), and dysuria (20%). Adenovirus can present with dysuria, meatal inflammation, and conjunctivitis (*718*). Enteric bacteria have been identified as an uncommon cause of NGU and might be associated with insertive anal intercourse (*699*).

Other bacterial pathogens have been implicated as potential causes of clinical urethritis, either in clustered case series or as sporadic cases such as *Haemophilus influenzae* and *Haemophilus parainfluenzae* (*719*–*723*). *Haemophilus* was identified in 12.6% of cases among 413 men (mostly MSM reporting insertive oral sex) (*724*), and high rates of azithromycin resistance (39.5%) were identified among *Haemophilus* urethritis patients (*725*). Individual case reports have linked NGU to multiple bacterial species, including *Corynebacterium propinquum* (*726*), *Kurthia gibsonii* (*727*), *Corynebacterium glucuronolyticum* (*728*,*729*), *Corynebacterium striatrium* (*730*), *Aerococcus urinae* (*731*)*,* and *Neisseria elongata* (*732*). Diagnostic testing and treatment for less-common organisms are reserved for situations in which these infections are suspected (e.g., sexual partner with trichomoniasis, urethral lesions, or severe dysuria and meatitis) or when NGU is not responsive to recommended therapy.

Even in settings that provide comprehensive diagnostic testing, etiology can remain obscure in half of cases. Idiopathic NGU was reported in 772 (59%) of 1,295 first presentations of NGU among men seeking sexual health services in Australia (*701*). In a case-control study of 211 men with NGU symptoms in Denmark, no identifiable pathogen was identified in 24% of acute cases and 33% of chronic cases (*733*). NGU’s importance if not caused by a defined pathogen is uncertain; neither complications (e.g., urethral stricture or epididymitis) nor adverse outcomes among sex partners have been identified in these cases.

Associations between NGU and insertive anal and oral exposure have been reported (*734*), as have higher rates of BV-associated *Leptotrichia* or *Sneathia *species among heterosexual men with urethritis (*735*). These studies increase concern for possible undetected infectious rectal or vaginal pathogens, or alternatively, a transient reactive dysbiosis after exposure to a new microbiome or even a noninfectious reactive etiology (*736*).

#### Diagnostic Considerations

Clinicians should attempt to obtain objective evidence of urethral inflammation. If POC diagnostic tests (e.g., Gram stain or MB or GV microscopy) are unavailable, urethritis can be documented on the basis of any of the following signs or laboratory tests:

- Mucoid, mucopurulent, or purulent discharge on examination.
- Gram stain is a POC diagnostic test for evaluating urethritis that is highly sensitive and specific for documenting both urethritis and the presence or absence of gonococcal infection; MB or GV stain of urethral secretions is an alternative POC diagnostic test with performance characteristics similar to Gram stain; thus, the cutoff number for WBCs per oil immersion field should be the same (*737*).
  º Presumed gonococcal infection is established by documenting the presence of WBCs containing GNID in Gram stain or intracellular purple diplococci in MB or GV smears; men should be tested for *C. trachomatis* and *N. gonorrhoeae* by NAATs and presumptively treated and managed accordingly for gonococcal infection (see Gonococcal Infections).
  º If no intracellular gram-negative or purple diplococci are present, men should receive NAATs for *C. trachomatis* and *N. gonorrhoeae* and can be managed for NGU as recommended (see Nongonococcal Urethritis).
  º Gram stain of urethral secretions exist that demonstrate ≥2 WBCs per oil immersion field (*738*). The microscopy diagnostic cutoff might vary, depending on background prevalence (≥2 WBCs/high power field [HPF] in high-prevalence settings [STI clinics] or ≥5 WBCs/HPF in lower-prevalence settings).^§§^
- Positive leukocyte esterase test on first-void urine or microscopic examination of sediment from a spun first-void urine demonstrating ≥10 WBCs/HPF.

Men evaluated in settings in which Gram stain or MB or GV smear is unavailable who meet at least one criterion for urethritis (i.e., urethral discharge, positive leukocyte esterase test on first void urine, or microscopic examination of first-void urine sediment with ≥10 WBCs/HPF) should be tested for *C. trachomatis* and *N. gonorrhoeae* by NAATs and treated with regimens effective against gonorrhea and chlamydia.

If symptoms are present but no evidence of urethral inflammation is present, NAATs for *C. trachomatis* and *N. gonorrhoeae* might identify infections (*739*). Persons with chlamydia or gonorrhea should receive recommended treatment, and sex partners should be referred for evaluation and treatment. If none of these clinical criteria are present, empiric treatment of men with symptoms of urethritis is recommended only for those at high risk for infection who are unlikely to return for a follow-up evaluation or test results. Such men should be treated with drug regimens effective against gonorrhea and chlamydia.

### Nongonococcal Urethritis

NGU is a nonspecific diagnosis that can have various infectious etiologies. *C. trachomatis* has been well established as an NGU etiology; however, prevalence varies across populations and accounts for <50% of overall cases (*712*,*740*–*742*). *M. genitalium* is estimated to account for 10%–25% of cases (*696*,*697*,*701*,*703*,*704*,*706*,*733*,*743*), and *T. vaginalis* for 1%–8% of cases depending on population and location (*703*,*706*,*708*,*710*,*712*). Other etiologies include different bacteria, such as *Haemophilus* species (*724*,*725*), *N. meningitidis* (*713*,*716*), HSV (*706*,*717*), and adenovirus (*744*). However, even when extensive testing is performed, no pathogens are identified in approximately half of cases (*701*,*733*).

#### Diagnostic Considerations

Clinical presentation can include urethral discharge, irritation, dysuria, or meatal pruritus (*697*,*743*,*745*). NGU is confirmed for symptomatic men when diagnostic evaluation of urethral secretions indicates inflammation, without evidence of diplococci by Gram, MB, or GV smear on microscopy (*712*,*746*,*747*). Visible discharge or secretions can be collected by a swab without inserting it into the urethra; if no visible secretions, the swab can be inserted into the urethral meatus and rotated, making contact with the urethral wall before removal. If microscopy is unavailable, urine testing for leukocyte esterase can be performed on first-void urine, and microscopic examination of sediment from a spun first-void urine demonstrating ≥10 WBCs/HPF has a high negative predictive value.

All men who have suspected or confirmed NGU should be tested for chlamydia and gonorrhea by using NAATs. A specific diagnosis can potentially reduce complications, reinfection, and transmission. *M. genitalium* testing should be performed for men who have persistent or recurrent symptoms after initial empiric treatment. Testing for *T. vaginalis* should be considered in areas or among populations with high prevalence, in cases where a partner is known to be infected, or for men who have persistent or recurrent symptoms after initial empiric treatment.

#### Treatment

Ideally, treatment should be pathogen based; however, diagnostic information might not be immediately available. Presumptive treatment should be initiated at NGU diagnosis. Doxycycline is highly effective for chlamydial urethral infections and is also effective for chlamydial infections of the rectum; it also has some activity against *M. genitalium*. In contrast, reports have increased of azithromycin treatment failures for chlamydial infection (*748*,*749*), and the incidence of macrolide resistance in *M. genitalium* also has been rapidly rising (*697*,*702*,*705*,*750*,*751*). Pharmacokinetic data indicate that changing azithromycin dosing from a single-dose strategy to a multiday strategy might protect against inducing resistance in *M. genitalium* infections (*745*,*752*) (see *Mycoplasma genitalium*).

**Recommended Regimen for Nongonococcal Urethritis**
**Doxycycline **100 mg orally 2 times/day for 7 days
**Alternative Regimens**
**Azithromycin **1 g orally in a single dose
*or*
**Azithromycin** 500 mg orally in a single dose; then 250 mg orally daily for 4 days

To maximize compliance with recommended therapies, medications should be dispensed on-site at the clinic, and, regardless of the number of doses involved in the regimen, the first dose should be directly observed. Erythromycin is no longer recommended for NGU because of its gastrointestinal side effects and dosing frequency. Levofloxacin is no longer recommended for NGU because of its inferior efficacy, especially for *M. genitalium.*

#### Management Considerations

To minimize transmission and reinfections, men treated for NGU should be instructed to abstain from sexual intercourse until they and their partners have been treated (i.e., until completion of a 7-day regimen and symptoms have resolved or for 7 days after single-dose therapy). Men with NGU should be tested for HIV and syphilis.

#### Follow-Up

Men should be provided their testing results obtained as part of the NGU evaluation. Those with a specific diagnosis of chlamydia, gonorrhea, or trichomoniasis should be offered partner services and instructed to return 3 months after treatment for repeat testing because of high rates of reinfection, regardless of whether their sex partners were treated (*136*,*137*,*753*,*754*) (see Chlamydial Infections; Gonococcal Infections; Trichomoniasis).

If symptoms persist or recur after therapy completion, men should be instructed to return for reevaluation and should be tested for *M. genitalium* and *T. vaginalis*. Symptoms alone, without documentation of signs or laboratory evidence of urethral inflammation, are insufficient basis for retreatment. Providers should be alert to the possible diagnosis of chronic prostatitis or chronic pelvic pain syndrome in men experiencing persistent perineal, penile, or pelvic pain or discomfort; voiding symptoms; pain during or after ejaculation; or new-onset premature ejaculation lasting for >3 months. Men with persistent pain should be referred to a urologist with expertise in pelvic pain disorders.

#### Management of Sex Partners

All sex partners of men with NGU within the preceding 60 days should be referred for evaluation and testing and presumptive treatment with a drug regimen effective against chlamydia. All partners should be evaluated and treated according to the management section for their respective pathogen; EPT could be an alternate approach if a partner is unable to access timely care. To avoid reinfection, sex partners should abstain from sexual intercourse until they and their partners are treated.

#### Persistent or Recurrent Nongonococcal Urethritis

The objective diagnosis of persistent or recurrent NGU should be made before considering additional antimicrobial therapy. Symptomatic recurrent or persistent urethritis might be caused by treatment failure or reinfection after successful treatment. Among men who have persistent symptoms after treatment without objective signs of urethral inflammation, the value of extending the duration of antimicrobials has not been demonstrated. Treatment failure for chlamydial urethritis has been estimated at 6%–12% (*755*). The most common cause of persistent or recurrent NGU is *M. genitalium*, especially after doxycycline therapy (*756*,*757*). Treatment failure for *M. genitalium* is harder to determine because certain men achieve clinical cure (i.e., resolution of symptoms) but can still have detectable *M. genitalium* in urethral specimens (*758*).

The initial step in recurrent urethritis is assessing compliance with treatment or potential reexposure to an untreated sex partner (*697*,*743*). If the patient did not comply with the treatment regimen or was reexposed to an untreated partner, retreatment with the initial regimen can be considered. If therapy was appropriately completed and no reexposure occurred, therapy is dependent on the initial treatment regimen. Ideally, diagnostic testing among men with recurrent or persistent symptoms, including those with gonorrhea, chlamydia, *M. genitalium*, and trichomoniasis, can be used to guide further management decisions.

*T. vaginalis* is also known to cause urethritis among men who have sex with women. In areas where *T. vaginalis* is prevalent, men who have sex with women with persistent or recurrent urethritis should be tested for *T. vaginalis* and presumptively treated with metronidazole 2 g orally in a single dose or tinidazole 2 g orally in a single dose; their partners should be referred for evaluation and treatment, if needed.

If *T. vaginalis* is unlikely (MSM with NGU or negative *T. vaginalis* NAAT), men with recurrent NGU should be tested for *M. genitalium* by using an FDA-cleared NAAT. Treatment for *M. genitalium* includes a two-stage approach, ideally using resistance-guided therapy. If *M. genitalium* resistance testing is available it should be performed, and the results should be used to guide therapy (see *Mycoplasma genitalium*). If *M. genitalium* resistance testing is not available, doxycycline 100 mg orally 2 times/day for 7 days followed by moxifloxacin 400 mg orally once daily for 7 days should be used. The rationale for this approach is that although not curative, doxycycline decreases the *M. genitalium* bacterial load, thereby increasing likelihood of moxifloxacin success (*759*). Higher doses of azithromycin have not been effective for *M. genitalium* after azithromycin treatment failures. Men with persistent or recurrent NGU after treatment for *M. genitalium *or* T. vaginalis* should be referred to an infectious disease or urology specialist.

#### Special Considerations

##### HIV Infection

NGU might facilitate HIV transmission (*760*). Persons with NGU and HIV infection should receive the same treatment regimen as those who do not have HIV.

### Cervicitis

Two major diagnostic signs characterize cervicitis: 1) a purulent or mucopurulent endocervical exudate visible in the endocervical canal or on an endocervical swab specimen (commonly referred to as mucopurulent cervicitis), and 2) sustained endocervical bleeding easily induced by gentle passage of a cotton swab through the cervical os. Either or both signs might be present. Cervicitis frequently is asymptomatic; however, certain women might report an abnormal vaginal discharge and intermenstrual vaginal bleeding (e.g., especially after sexual intercourse). The criterion of using an increased number of WBCs on endocervical Gram stain in the diagnosis of cervicitis has not been standardized; it is not sensitive, has a low positive predictive value for *C. trachomatis* and *N. gonorrhoeae* infections, and is not available in most clinical settings (*297*,*761*). Leukorrhea, defined as >10 WBCs/HPF on microscopic examination of vaginal fluid, might be a sensitive indicator of cervical inflammation with a high negative predictive value (i.e., cervicitis is unlikely in the absence of leukorrhea) (*762*,*763*). Finally, although the presence of gram-negative intracellular diplococci on Gram stain of endocervical exudate might be specific for diagnosing gonococcal cervical infection when evaluated by an experienced laboratorian, it is not a sensitive indicator of infection (*764*).

#### Etiology

*C. trachomatis* or *N. gonorrhoeae* is the most common etiology of cervicitis defined by diagnostic testing. Trichomoniasis, genital herpes (especially primary HSV-2 infection), or *M. genitalium* (*761*,*765*–*768*) also have been associated with cervicitis. However, in many cases of cervicitis, no organism is isolated, especially among women at relatively low risk for recent acquisition of these STIs (e.g., women aged >30 years) (*769*). Limited data indicate that BV and frequent douching might cause cervicitis (*770*–*772*). The majority of persistent cases of cervicitis are not caused by reinfection with *C. trachomatis* or *N. gonorrhoeae;* other factors might be involved (e.g., persistent abnormality of vaginal flora, *M. genitalium,* douching or exposure to other types of chemical irritants, dysplasia, or idiopathic inflammation in the zone of ectopy). Available data do not indicate an association between group B streptococcus colonization and cervicitis (*773*,*774*). No specific evidence exists for a role for *Ureaplasma parvum* or *Ureaplasma urealyticum* in cervicitis (*707*,*761*,*765*,*775*,*776*).

#### Diagnostic Considerations

Because cervicitis might be a sign of upper genital tract infection (e.g., endometritis), women should be assessed for signs of PID and tested for *C. trachomatis* and *N. gonorrhoeae* with NAAT on vaginal, cervical, or urine samples (*553*) (see Chlamydial Infections; Gonococcal Infections). Women with cervicitis also should be evaluated for concomitant BV and trichomoniasis. Because sensitivity of microscopy for detecting *T. vaginalis* is relatively low (approximately 50%), symptomatic women with cervicitis and negative wet-mount microscopy for trichomonads should receive further testing (i.e., NAAT, culture, or other FDA-cleared diagnostic test) (see Trichomoniasis). Testing for *M. genitalium* with the FDA-cleared NAAT can be considered. Although HSV-2 infection has been associated with cervicitis, the utility of specific testing (i.e., PCR or culture) for HSV-2 is unknown. Testing for *U. parvum, U. urealyticum, Mycoplasma hominis*, or genital culture for group B streptococcus is not recommended.

#### Treatment

Multiple factors should affect the decision to provide presumptive therapy for cervicitis. Presumptive treatment with antimicrobials for *C. trachomatis* and *N. gonorrhoeae* should be provided for women at increased risk (e.g., those aged <25 years and women with a new sex partner, a sex partner with concurrent partners, or a sex partner who has an STI), if follow-up cannot be ensured, or if testing with NAAT is not possible. Trichomoniasis and BV should be treated if detected (see Bacterial Vaginosis; Trichomoniasis). For women at lower risk for STIs, deferring treatment until results of diagnostic tests are available is an option. If treatment is deferred and *C. trachomatis* and *N. gonorrhoeae* NAATs are negative, a follow-up visit to determine whether the cervicitis has resolved can be considered.

Recommended Regimen for Cervicitis*
**Doxycycline **100 mg orally 2 times/day for 7 days
* Consider concurrent treatment for gonococcal infection if the patient is at risk for gonorrhea or lives in a community where the prevalence of gonorrhea is high (see Gonococcal Infections).
**Alternative Regimen**
**Azithromycin** 1 g orally in a single dose

#### Other Management Considerations

To minimize transmission and reinfection, women treated for cervicitis should be instructed to abstain from sexual intercourse until they and their partners have been treated (i.e., until completion of a 7-day regimen or for 7 days after single-dose therapy) and symptoms have resolved. Women who receive a cervicitis diagnosis should be tested for syphilis and HIV in addition to other recommended diagnostic tests.

#### Follow-Up

Women receiving treatment should return to their provider for a follow-up visit to determine whether cervicitis has resolved. For women who are untreated, a follow-up visit gives providers an opportunity to communicate test results obtained as part of the cervicitis evaluation. Providers should treat on the basis of any positive test results and determine whether cervicitis has resolved. Women with a specific diagnosis of chlamydia, gonorrhea, or trichomoniasis should be offered partner services and instructed to return in 3 months after treatment for repeat testing because of high rates of reinfection, regardless of whether their sex partners were treated (*753*). If symptoms persist or recur, women should be instructed to return for reevaluation.

#### Management of Sex Partners

Management of sex partners of women treated for cervicitis should be tailored for the specific infection identified or suspected. All sex partners during the previous 60 days should be referred for evaluation, testing, and presumptive treatment if chlamydia, gonorrhea, or trichomoniasis was identified. EPT and other effective partner referral strategies are alternative approaches for treating male partners of women who have chlamydial or gonococcal infection (*125*–*127*) (see Partner Services). To avoid reinfection, sex partners should abstain from sexual intercourse until they and their partners are treated.

#### Persistent or Recurrent Cervicitis

Women with persistent or recurrent cervicitis despite antimicrobial therapy should be reevaluated for possible reexposure or treatment failure. If relapse or reinfection with a specific infection has been excluded, BV is not present, and sex partners have been evaluated and treated, management options for persistent cervicitis are undefined. In addition, the usefulness of repeated or prolonged administration of antimicrobial therapy for persistent symptomatic cervicitis remains unknown. The etiology of persistent cervicitis, including the potential role of *M. genitalium* (*777*)*,* is unclear. *M. genitalium* might be considered for cases of cervicitis that persist after azithromycin or doxycycline therapy in which reexposure to an infected partner or medical nonadherence is unlikely. Among women with persistent cervicitis who were previously treated with doxycycline or azithromycin, testing for *M. genitalium* can be considered and treatment initiated on the basis of results of diagnostic testing (*318*) (see *Mycoplasma genitalium*). For women with persistent symptoms that are clearly attributable to cervicitis, referral to a gynecologic specialist can be considered for evaluation of noninfectious causes (e.g., cervical dysplasia or polyps) (*778*).

#### Special Considerations

##### HIV Infection

Women with cervicitis and HIV infection should receive the same treatment regimen as those who do not have HIV. Cervicitis can increase cervical HIV shedding, and treatment reduces HIV shedding from the cervix and thereby might reduce HIV transmission to susceptible sex partners (*779*–*783*).

##### Pregnancy

Diagnosis and treatment of cervicitis for pregnant women does not differ from that for women who are not pregnant (see Diagnostic Considerations; Treatment).

##### Contraceptive Management

According to *U.S. Medical Eligibility Criteria for Contraceptive Use, 2016*, leaving an IUD in place during treatment for cervicitis is advisable (*58*). However, current recommendations specify that an IUD should not be placed if active cervicitis is diagnosed (*59*).

## Chlamydial Infections

### Chlamydial Infection Among Adolescents and Adults

Chlamydial infection is the most frequently reported bacterial infectious disease in the United States, and prevalence is highest among persons aged ≤24 years (*141*,*784*). Multiple sequelae can result from *C. trachomatis* infection among women, the most serious of which include PID, ectopic pregnancy, and infertility. Certain women who receive a diagnosis of uncomplicated cervical infection already have subclinical upper genital tract infection.

Asymptomatic infection is common among both men and women. To detect chlamydial infection, health care providers frequently rely on screening tests. Annual screening of all sexually active women aged <25 years is recommended, as is screening of older women at increased risk for infection (e.g., women aged ≥25 years who have a new sex partner, more than one sex partner, a sex partner with concurrent partners, or a sex partner who has an STI) (*149*). In a community-based cohort of female college students, incident chlamydial infection was also associated with BV and high-risk HPV infection (*785*). Although chlamydia incidence might be higher among certain women aged ≥25 years in certain communities, overall, the largest proportion of infection is among women aged <25 years (*141*).

Chlamydia screening programs have been demonstrated to reduce PID rates among women (*786*,*787*). Although evidence is insufficient to recommend routine screening for *C. trachomatis* among sexually active young men because of certain factors (i.e., feasibility, efficacy, and cost-effectiveness), screening of sexually active young men should be considered in clinical settings with a high prevalence of chlamydia (e.g., adolescent clinics, correctional facilities, or STD specialty clinics) or for populations with a high burden of infection (e.g., MSM) (*149*,*788*). Among women, the primary focus of chlamydia screening should be to detect and treat chlamydia, prevent complications, and test and treat their partners, whereas targeted chlamydia screening for men should be considered only when resources permit, prevalence is high, and such screening does not hinder chlamydia screening efforts for women (*789*–*791*). More frequent screening than annual for certain women (e.g., adolescents) or certain men (e.g., MSM) might be indicated on the basis of risk behaviors.

#### Diagnostic Considerations

For women,* C. trachomatis* urogenital infection can be diagnosed by vaginal or cervical swabs or first-void urine. For men, *C. trachomatis* urethral infection can be diagnosed by testing first-void urine or a urethral swab. NAATs are the most sensitive tests for these specimens and are the recommended test for detecting *C. trachomatis* infection (*553*). NAATs that are FDA cleared for use with vaginal swab specimens can be collected by a clinician or patient in a clinical setting. Patient-collected vaginal swab specimens are equivalent in sensitivity and specificity to those collected by a clinician using NAATs (*792*,*793*), and this screening strategy is highly acceptable among women (*794*,*795*). Optimal urogenital specimen types for chlamydia screening by using NAAT include first-catch urine (for men) and vaginal swabs (for women) (*553*). Recent studies have demonstrated that among men, NAAT performance on self-collected meatal swabs is comparable to patient-collected urine or provider-collected urethral swabs (*796*–*798*). Patient collection of a meatal swab for *C. trachomatis* testing might be a reasonable approach for men who are either unable to provide urine or prefer to collect their own meatal swab over providing urine. Previous evidence indicates that the liquid-based cytology specimens collected for Pap smears might be acceptable specimens for NAAT, although test sensitivity using these specimens might be lower than that associated with use of cervical or vaginal swab specimens (*799*); regardless, certain NAATs have been cleared by FDA for use on liquid-based cytology specimens.

Rectal and oropharyngeal *C. trachomatis* infection among persons engaging in receptive anal or oral intercourse can be diagnosed by testing at the anatomic exposure site. NAATs have been demonstrated to have improved sensitivity and specificity, compared with culture, for detecting *C. trachomatis* at rectal and oropharyngeal sites (*553*,*800*–*804*), and certain NAAT platforms have been cleared by FDA for these anatomic sites (*805*). Data indicate that NAAT performance on self-collected rectal swabs is comparable to clinician-collected rectal swabs, and this specimen collection strategy for rectal *C. trachomatis* screening is highly acceptable among men (*217*,*806*). Self-collected rectal swabs are a reasonable alternative to clinician-collected rectal swabs for *C. trachomatis* screening by NAAT, especially when clinicians are not available or when self-collection is preferred over clinician collection. Annual screening for rectal *C. trachomatis* infection should be performed among men who report sexual activity at the rectal site. Extragenital chlamydial testing at the rectal site can be considered for females on the basis of reported sexual behaviors and exposure through shared clinical decision-making by the patient and the provider. The majority of persons with *C. trachomatis* detected at oropharyngeal sites do not have oropharyngeal symptoms. The clinical significance of oropharyngeal *C. trachomatis* infection is unclear, and prevalence is low, even among populations at high risk. However, when gonorrhea testing is performed at the oropharyngeal site, chlamydia test results might be reported because certain NAATs detect both bacteria from a single specimen.

POC tests for *C. trachomatis* among asymptomatic persons can expedite treatment of infected persons and their sex partners. Among symptomatic patients, POC tests for *C. trachomatis* can optimize treatment by limiting unnecessary presumptive treatment at the time of clinical decision-making and improve antimicrobial stewardship. Thus, using a POC test will likely be a cost-effective diagnostic strategy for *C. trachomatis* infection (*807*). Newer NAAT-based POC tests have promising performance and are becoming commercially available (*807*–*809*).

#### Treatment

Treating persons with *C. trachomatis* prevents adverse reproductive health complications and continued sexual transmission. Furthermore, treating their sex partners can prevent reinfection and infection of other partners. Treating pregnant women usually prevents transmission of *C. trachomatis* to neonates during birth. Treatment should be provided promptly for all persons with chlamydial infection; treatment delays have been associated with complications (e.g., PID) in a limited proportion of women (*810*).

**Recommended Regimen for Chlamydial Infection Among Adolescents and Adults**
**Doxycycline** 100 mg orally 2 times/day for 7 days
**Alternative Regimens**
**Azithromycin** 1 g orally in a single dose
*or*
**Levofloxacin** 500 mg orally once daily for 7 days

A meta-analysis and a Cochrane systematic review evaluated data from randomized clinical trials of azithromycin versus doxycycline for treating urogenital chlamydial infection determined that microbiologic treatment failure among men was higher for azithromycin than for doxycycline (*748*,*749*). Observational studies have also demonstrated that doxycycline is more efficacious for rectal *C. trachomatis* infection for men and women than azithromycin (*748*,*811*). A randomized trial for the treatment of rectal chlamydia infection among MSM reported microbiologic cure was 100% with doxycycline and 74% with azithromycin (*812*). A published review reported that *C. trachomatis* was detected at the anorectal site among 33%–83% of women who had urogenital *C. trachomatis* infection, and its detection was not associated with report of receptive anorectal sexual activity (*813*).

Although the clinical significance of oropharyngeal *C. trachomatis* infection is unclear and routine oropharyngeal screening is not recommended, oropharyngeal *C. trachomatis* can be sexually transmitted to genital sites (*211*,*814*); therefore, if *C. trachomatis* is identified from an oropharyngeal specimen while screening for pharyngeal gonorrhea, it should be treated. Evidence is limited regarding the efficacy of antimicrobial regimens for oropharyngeal chlamydia; however, a recently published observational study indicates doxycycline might be more efficacious than azithromycin for oropharyngeal chlamydia (*815*).

Available evidence supports that doxycycline is efficacious for *C. trachomatis* infections of urogenital, rectal, and oropharyngeal sites. Although azithromycin maintains high efficacy for urogenital *C. trachomatis* infection among women, concern exists regarding effectiveness of azithromycin for concomitant rectal *C. trachomatis* infection, which can occur commonly among women and cannot be predicted by reported sexual activity. Inadequately treated rectal *C. trachomatis* infection among women who have urogenital chlamydia can increase the risk for transmission and place women at risk for repeat urogenital *C. trachomatis* infection through autoinoculation from the anorectal site (*816*). Doxycycline is also available in a delayed-release 200-mg tablet formulation, which requires once-daily dosing for 7 days and is as effective as doxycycline 100 mg twice daily for 7 days for treating urogenital *C. trachomatis* infection in men and women. It is more costly but also has lower frequency of gastrointestinal side effects (*817*). Levofloxacin is an effective treatment alternative but is more expensive. Erythromycin is no longer recommended because of the frequency of gastrointestinal side effects, which can result in nonadherence. When nonadherence to doxycycline regimen is a substantial concern, azithromycin 1 g regimen is an alternative treatment option but might require posttreatment evaluation and testing because it has demonstrated lower treatment efficacy among persons with rectal infection.

Among persons receiving multidose regimens, medication should be dispensed with all doses involved, on-site and in the clinic, and the first dose should be directly observed. To maximize adherence with recommended therapies, on-site, directly observed single-dose therapy with azithromycin should always be available for persons for whom adherence with multiday dosing is a considerable concern.

#### Other Management Considerations

To minimize disease transmission to sex partners, persons treated for chlamydia should be instructed to abstain from sexual intercourse for 7 days after single-dose therapy or until completion of a 7-day regimen and resolution of symptoms if present. To minimize risk for reinfection, patients also should be instructed to abstain from sexual intercourse until all of their sex partners have been treated. Persons who receive a diagnosis of chlamydia should be tested for HIV, gonorrhea, and syphilis. MSM who are HIV negative with a rectal chlamydia diagnosis should be offered HIV PrEP.

#### Follow-Up

Test of cure to detect therapeutic failure (i.e., repeat testing 4 weeks after completing therapy) is not advised for nonpregnant persons treated with the recommended or alternative regimens, unless therapeutic adherence is in question, symptoms persist, or reinfection is suspected. Moreover, using chlamydial NAATs at <4 weeks after completion of therapy is not recommended because the continued presence of nonviable organisms (*553*,*818*,*819*) can lead to false-positive results.

A high prevalence of *C. trachomatis* infection has been observed among women and men who were treated for chlamydial infection during the preceding months (*753*,*755*,*820*–*822*). The majority of posttreatment infections do not result from treatment failure but rather from reinfection caused by failure of sex partners to receive treatment or initiation of sexual activity with a new infected partner (*823*), indicating a need for improved education and treatment of sex partners. Repeat infections confer an elevated risk for PID and other complications among women. Men and women who have been treated for chlamydia should be retested approximately 3 months after treatment, regardless of whether they believe their sex partners were treated; scheduling the follow-up visit at the time of treatment is encouraged (*753*). If retesting at 3 months is not possible, clinicians should retest whenever persons next seek medical care <12 months after initial treatment.

#### Management of Sex Partners

Sex partners should be referred for evaluation, testing, and presumptive treatment if they had sexual contact with the partner during the 60 days preceding the patient’s onset of symptoms or chlamydia diagnosis. Although the exposure intervals defining identification of sex partners at risk are based on limited data, the most recent sex partner should be evaluated and treated, even if the time of the last sexual contact was >60 days before symptom onset or diagnosis.

If health department partner management strategies (e.g., disease intervention specialists) are impractical or unavailable for persons with chlamydia, and if a provider is concerned that sex partners are unable to promptly access evaluation and treatment services, EPT should be considered as permitted by law (see Partner Services). Compared with standard patient referral of partners, this approach to therapy, which involves delivering the medication itself or a prescription by the patient or collaborating pharmacy, has been associated with decreased rates of persistent or recurrent chlamydia among women (*125*–*127*). Providers should provide patients with written educational materials to give to their partners about chlamydia, which should include notification that partners have been exposed and information about the importance of treatment. These materials also should inform partners about potential therapy-related allergies and adverse effects, along with symptoms indicative of complications (e.g., testicular pain among men and pelvic or abdominal pain among women). Educational materials for female partners should include information about the importance of seeking medical evaluation, especially if PID symptoms are present; undertreatment of PID among female partners and missed opportunities for diagnosing other STIs among women are concerning. MSM with chlamydia have a high risk for coexisting infections, especially undiagnosed HIV, among their partners and might have partners without HIV who could benefit from HIV PrEP. Data are also limited regarding effectiveness of EPT in reducing persistent or recurrent chlamydia among MSM (*123*,*133*,*134*); thus, shared clinical decision-making regarding EPT for MSM is recommended. Having partners accompany patients when they return for treatment is another strategy that has been used successfully for ensuring partner treatment (see Partner Services). To avoid reinfection, sex partners should be instructed to abstain from condomless sexual intercourse until they and their sex partners have been treated (i.e., after completion of a 7-day regimen) and any symptoms have resolved.

#### Special Considerations

##### Pregnancy

Clinical experience and published studies indicate that azithromycin is safe and effective during pregnancy (*824*–*826*). Doxycycline is contraindicated during the second and third trimesters of pregnancy because of risk for tooth discoloration. Human data reveal that levofloxacin presents a low risk to the fetus during pregnancy but has potential for toxicity during breastfeeding; however, data from animal studies increase concerns regarding cartilage damage to neonates (*431*).

Test of cure (i.e., repeat testing after completion of therapy) to document chlamydial eradication, preferably by NAAT, at approximately 4 weeks after therapy completion during pregnancy is recommended because severe sequelae can occur among mothers and neonates if the infection persists. In addition, all pregnant women who have chlamydial infection diagnosed should be retested 3 months after treatment. Detection of *C. trachomatis* infection during the third semester is not uncommon among adolescent and young adult women, including those without *C. trachomatis* detected at the time of initial prenatal screening (*827*). Women aged <25 years and those at increased risk for chlamydia (i.e., those who have a new sex partner, more than one sex partner, a sex partner with concurrent partners, or a sex partner who has an STI) should be screened at the first prenatal visit and rescreened during the third trimester to prevent maternal postnatal complications and chlamydial infection in the infant (*149*).

**Recommended Regimen for Chlamydial Infection During Pregnancy**
**Azithromycin** 1 g orally in a single dose
**Alternative Regimen**
**Amoxicillin** 500 mg orally 3 times/day for 7 days

Because of concerns regarding chlamydia persistence after exposure to penicillin-class antibiotics that has been demonstrated in animal and in vitro studies, amoxicillin is listed as an alternative therapy for *C. trachomatis* for pregnant women (*828*,*829*). Erythromycin is no longer recommended because of the frequency of gastrointestinal side effects that can result in therapy nonadherence. In addition, systematic reviews and meta-analyses have noted an association with macrolide antimicrobials, especially erythromycin, during pregnancy and adverse child outcomes, indicating cautious use in pregnancy (*830*–*831*).

##### HIV Infection

Persons who have chlamydia and HIV infection should receive the same treatment regimen as those who do not have HIV.

### Chlamydial Infection Among Neonates

Prenatal screening and treatment of pregnant women is the best method for preventing chlamydial infection among neonates. *C. trachomatis* infection of neonates results from perinatal exposure to the mother’s infected cervix. Initial *C. trachomatis* neonatal infection involves the mucous membranes of the eye, oropharynx, urogenital tract, and rectum, although infection might be asymptomatic in these locations. Instead, *C. trachomatis* infection among neonates is most frequently recognized by conjunctivitis that develops 5–12 days after birth. *C. trachomatis* also can cause a subacute, afebrile pneumonia with onset at ages 1–3 months. Although *C. trachomatis* has been the most frequent identifiable infectious cause of ophthalmia neonatorum, neonatal chlamydial infections, including ophthalmia and pneumonia, have occurred less frequently since institution of widespread prenatal screening and treatment of pregnant women. Neonates born to mothers at high risk for chlamydial infection, with untreated chlamydia, or with no or unconfirmed prenatal care, are at high risk for infection. However, presumptive treatment of the neonate is not indicated because the efficacy of such treatment is unknown. Infants should be monitored to ensure prompt and age-appropriate treatment if symptoms develop. Processes should be in place to ensure communication between physicians and others caring for the mother and the newborn to ensure thorough monitoring of the newborn after birth.

#### Ophthalmia Neonatorum Caused by *C. trachomatis*

A chlamydial etiology should be considered for all infants aged ≤30 days who experience conjunctivitis, especially if the mother has a history of chlamydial infection. These infants should receive evaluation and age-appropriate care and treatment.

##### Preventing Ophthalmia Neonatorum Caused by *C. trachomatis*

Neonatal ocular prophylaxis with erythromycin, the only agent available in the United States for this purpose, is ineffective against chlamydial ophthalmia neonatorum (or pneumonia) (*833*). As an alternative, prevention efforts should focus on prenatal screening for *C. trachomatis,* including

- screening pregnant women at risk for *C. trachomatis* infection at the first prenatal visit (e.g., women aged <25 years and those aged ≥25 years who have a new sex partner, more than one sex partner, a sex partner with concurrent partners, or a sex partner who has an STI);
- treating all pregnant women with *C. trachomatis* during pregnancy and performing a test of cure 4 weeks after treatment to verify chlamydial eradication; these women should also be retested 3 months after treatment and again in the third trimester or at time of delivery, and their partners should also be tested and treated;
- retesting pregnant women during the third trimester who initially tested negative but remained at increased risk for acquiring infection (e.g., women aged <25 years and those aged ≥25 years who have a new sex partner, more than one sex partner, a sex partner with concurrent partners, or a sex partner who has an STI); and
- screening at delivery those pregnant women who were not screened for *C. trachomatis* during pregnancy if at risk or who had no prenatal care; physicians and others caring for the mother and the newborn should communicate to ensure follow-up on the results of laboratory tests performed at delivery, and if positive, prompt and age-appropriate treatment for the newborn and the mother.

Neonates born to mothers for whom prenatal chlamydia screening has been confirmed and the results are negative are not at high risk for infection.

##### Diagnostic Considerations

Sensitive and specific methods for diagnosing chlamydial ophthalmia in the neonate include both tissue culture and nonculture tests (e.g., DFA tests and NAATs). DFA is the only nonculture FDA-cleared test for detecting chlamydia from conjunctival swabs. NAATs are not cleared by FDA for detecting chlamydia from conjunctival swabs, and clinical laboratories should verify the procedure according to CLIA regulations. Specimens for culture isolation and nonculture tests should be obtained from the everted eyelid by using a Dacron (DuPont)-tipped swab or the swab specified by the manufacturer’s test kit; for culture and DFA, specimens must contain conjunctival cells, not exudate alone. Ocular specimens from neonates being evaluated for chlamydial conjunctivitis also should be tested for *N. gonorrhoeae* (see Ophthalmia Neonatorum Caused by *N. gonorrhoeae*).

##### Treatment

**Recommended Regimen for Chlamydial Infection Among Neonates**
**Erythromycin base or ethyl succinate** 50 mg/kg body weight/day orally, divided into 4 doses daily for 14 days*
* An association between oral erythromycin and azithromycin and infantile hypertrophic pyloric stenosis (IHPS) has been reported among infants aged <6 weeks. Infants treated with either of these antimicrobials should be followed for IHPS signs and symptoms.

Although data regarding use of azithromycin for treating neonatal chlamydial infection are limited, available data demonstrate that a short therapy course might be effective (*834*). Topical antibiotic therapy alone is inadequate for treating ophthalmia neonatorum caused by chlamydia and is unnecessary when systemic treatment is administered.

##### Follow-Up

Because the efficacy of erythromycin treatment for ophthalmia neonatorum is approximately 80%, a second course of therapy might be required (*834*,*835*). Data regarding the efficacy of azithromycin for ophthalmia neonatorum are limited. Therefore, follow-up of infants is recommended to determine whether the initial treatment was effective. The possibility of concomitant chlamydial pneumonia should be considered (see Infant Pneumonia Caused by *C. trachomatis*).

##### Management of Mothers and Their Sex Partners

Mothers of infants who have ophthalmia caused by chlamydia and the sex partners of these women should be evaluated and presumptively treated for chlamydia (see Chlamydial Infection Among Adolescents and Adults).

#### Infant Pneumonia Caused by *C. trachomatis*

Chlamydial pneumonia among infants typically occurs at age 1–3 months and is a subacute pneumonia. Characteristic signs of chlamydial pneumonia among infants include a repetitive staccato cough with tachypnea and hyperinflation and bilateral diffuse infiltrates on a chest radiograph. In addition, peripheral eosinophilia (≥400 cells/mm^3^) occurs frequently. Because clinical presentations differ, all infants aged 1–3 months suspected of having pneumonia, especially those whose mothers have a history of, are at risk for (e.g., aged <25 years and those aged ≥25 years who have a new sex partner, more than one sex partner, a sex partner with concurrent partners, or a sex partner who has an STI), or suspected of having a chlamydial infection should be tested for *C. trachomatis* and treated if infected.

##### Diagnostic Considerations

Specimens for chlamydial testing should be collected from the nasopharynx. Tissue culture is the definitive standard diagnostic test for chlamydial pneumonia. Nonculture tests (e.g., DFA and NAAT) can be used. DFA is the only nonculture FDA-cleared test for detecting *C. trachomatis* from nasopharyngeal specimens; however, DFA of nasopharyngeal specimens has a lower sensitivity and specificity than culture. NAATs are not cleared by FDA for detecting chlamydia from nasopharyngeal specimens, and clinical laboratories should verify the procedure according to CLIA regulations (*553*). Tracheal aspirates and lung biopsy specimens, if collected, should be tested for *C. trachomatis*.

##### Treatment

Because test results for chlamydia often are unavailable at the time initial treatment decisions are being made, treatment for *C. trachomatis* pneumonia frequently is based on clinical and radiologic findings, age of the infant (i.e., 1–3 months), and risk for chlamydia in the mother (i.e., aged <25 years, history of chlamydial infection, multiple sex partners, a sex partner with a concurrent partner, or a sex partner with a history of an STI). In the absence of laboratory results in a situation with a high degree of suspicion of chlamydial infection and the mother is unlikely to return with the infant for follow-up, exposed infants can be presumptively treated with the shorter-course regimen of azithromycin 20 mg/kg body weight/day orally, 1 dose daily for 3 days.

**Recommended Regimen for Chlamydial Pneumonia Among Infants**
**Erythromycin base or ethyl succinate** 50 mg/kg body weight/day orally divided into 4 doses daily for 14 days
**Alternative Regimen**
**Azithromycin suspension** 20 mg/kg body weight/day orally, 1 dose daily for 3 days

##### Follow-Up

Because erythromycin effectiveness in treating pneumonia caused by *C. trachomatis* is approximately 80%, a second course of therapy might be required (*836*). Data regarding effectiveness of azithromycin in treating chlamydial pneumonia are limited. Follow-up of infants is recommended to determine whether the pneumonia has resolved, although certain infants with chlamydial pneumonia continue to have abnormal pulmonary function tests later during childhood.

##### Management of Mothers and Their Sex Partners

Mothers of infants who have chlamydial pneumonia and the sex partners of these women should be evaluated, tested, and presumptively treated for chlamydia (see Chlamydial Infection Among Adolescents and Adults).

### Chlamydial Infection Among Infants and Children

Sexual abuse should be considered a cause of chlamydial infection among infants and children. However, perinatally transmitted *C. trachomatis* infection of the nasopharynx, urogenital tract, and rectum can persist for 2–3 years (see Sexual Assault or Abuse of Children).

#### Diagnostic Considerations

NAATs can be used to test vaginal and urine specimens from girls and urine in boys (see Sexual Assault or Abuse of Children). Data are lacking regarding use of NAATs for specimens from extragenital sites (rectum and pharynx) among boys and girls (*553*); other nonculture tests (e.g., DFA) are not recommended because of specificity concerns. Although data regarding NAATs for specimens from extragenital sites for children are more limited and performance is test dependent (*553*), no evidence supports that NAAT performance for detecting *C. trachomatis* for extragenital sites among children would differ from that among adults. Because of the implications of a diagnosis of *C. trachomatis* infection in a child, only CLIA-validated, FDA-cleared NAAT should be used for extragenital site specimens (*837*).

**Recommended Regimens for Chlamydial Infection Among Infants and Children**
**For infants and children weighing <45 kg: Erythromycin base or ethyl succinate** 50 mg/kg body weight/day orally divided into 4 doses daily for 14 days
Data are limited regarding the effectiveness and optimal dose of azithromycin for treating chlamydial infection among infants and children weighing <45 kg.
**For children weighing ≥45 kg but aged <8 years: Azithromycin **1 g orally in a single dose
**For children aged ≥8 years: Azithromycin **1 g orally in a single dose
***or***
**Doxycycline** 100 mg orally 2 times/day for 7 days

#### Other Management Considerations

See Sexual Assault or Abuse of Children.

#### Follow-Up

A test of cure to detect therapeutic failure ensures treatment effectiveness and should be obtained at a follow-up visit approximately 4 weeks after treatment is completed.

## Gonococcal Infections

### Gonococcal Infection Among Adolescents and Adults

In the United States, an estimated 1,568,000 new *N. gonorrhoeae* infections occur each year (*141*,*838*), and gonorrhea is the second most commonly reported bacterial communicable disease. Urethral infections caused by *N. gonorrhoeae* can produce symptoms among men that cause them to seek curative treatment soon enough to prevent sequelae, but often not soon enough to prevent transmission to others. Among women, gonococcal infections are commonly asymptomatic or might not produce recognizable symptoms until complications (e.g., PID) have occurred. PID can result in tubal scarring that can lead to infertility or ectopic pregnancy.

Annual screening for *N. gonorrhoeae* infection is recommended for all sexually active women aged <25 years and for older women at increased risk for infection (e.g., those aged ≥25 years who have a new sex partner, more than one sex partner, a sex partner with concurrent partners, or a sex partner who has an STI) (*149*). Additional risk factors for gonorrhea include inconsistent condom use among persons who are not in mutually monogamous relationships, previous or coexisting STIs, and exchanging sex for money or drugs. Clinicians should consider the communities they serve and consult local public health authorities for guidance regarding identifying groups at increased risk. Gonococcal infection, in particular, is concentrated in specific geographic locations and communities. MSM at high risk for gonococcal infection (e.g., those with multiple anonymous partners or substance abuse) or those at risk for HIV acquisition should be screened at all anatomic sites of exposure every 3–6 months (see Men Who Have Sex with Men). At least annual screening is recommended for all MSM. Screening for gonorrhea among heterosexual men and women aged >25 years who are at low risk for infection is not recommended (*149*). A recent travel history with sexual contacts outside the United States should be part of any gonorrhea evaluation.

#### Diagnostic Considerations

Specific microbiologic diagnosis of *N. gonorrhoeae* infection should be performed for all persons at risk for or suspected of having gonorrhea; a specific diagnosis can potentially reduce complications, reinfections, and transmission. Culture, NAAT, and POC NAAT, such as GeneXpert (Cepheid), are available for detecting genitourinary infection with *N. gonorrhoeae* (*149*); culture requires endocervical (women) or urethral (men) swab specimens. Culture is also available for detecting rectal, oropharyngeal, and conjunctival gonococcal infection. NAATs and POC NAATs allow for the widest variety of FDA-cleared specimen types, including endocervical and vaginal swabs and urine for women, urethral swabs and urine for men, and rectal swabs and pharyngeal swabs for men and women (www.accessdata.fda.gov/cdrh_docs/reviews/K121710.pdf). However, product inserts for each NAAT manufacturer should be consulted carefully because collection methods and specimen types vary. Certain NAATs that have been demonstrated to detect commensal *Neisseria* species might have comparable low specificity when testing oropharyngeal specimens for *N. gonorrhoeae* (*553*). NAAT sensitivity for detecting *N. gonorrhoeae* from urogenital and nongenital anatomic sites is superior to culture but varies by NAAT type (*553*,*800*–*803*). For urogenital infections, optimal specimen types for gonorrhea screening using NAATs include first-void urine for men and vaginal swab specimens for women (*553*). Patient-collected samples can be used in place of provider-collected samples in clinical settings when testing by NAAT for urine (men and women), vaginal swabs, rectal swabs, and oropharyngeal swabs after patient instructions have been provided (*209*,*806*,*839*–*842*). Patient-collected specimens are reasonable alternatives to provider-collected swabs for gonorrhea screening by NAAT.

In cases of suspected or documented treatment failure, clinicians should perform both culture and antimicrobial susceptibility testing because NAATs cannot provide antimicrobial susceptibility results. Because *N. gonorrhoeae* has demanding nutritional and environmental growth requirements, optimal recovery rates are achieved when specimens are inoculated directly and when the growth medium is promptly incubated in an increased carbon dioxide (CO_2_) environment (*553*). Nonnutritive swab transport systems are available that might maintain gonococcal viability for <48 hours in ambient temperatures (*843*–*845*).

Because of its high specificity (>99%) and sensitivity (>95%), a Gram stain of urethral discharge or secretions that demonstrate polymorphonuclear leukocytes with intracellular gram-negative diplococci can be considered diagnostic for infection with *N. gonorrhoeae* among symptomatic men. However, because of lower sensitivity, a negative Gram stain should not be considered sufficient for ruling out infection among asymptomatic men. Infection detection by using Gram stain of endocervical, pharyngeal, and rectal specimens also is insensitive and is not recommended. MB or GV stain of urethral secretions is an alternative POC diagnostic test with performance characteristics similar to Gram stain. Gonococcal infection is diagnosed among symptomatic men by documenting the presence of a WBC-containing intracellular purple diplococci in MB or GV smears.

#### Antimicrobial-Resistant *N. gonorrhoeae*

Gonorrhea treatment is complicated by the ability of *N. gonorrhoeae* to develop resistance to antimicrobials (*846*–*848*). In 1986, the Gonococcal Isolate Surveillance Project (GISP), a national sentinel surveillance system, was established to monitor trends in antimicrobial susceptibilities of urethral *N. gonorrhoeae* strains in the United States (*849*). The epidemiology of antimicrobial resistance guides decisions about gonococcal treatment recommendations and has evolved because of shifts in antimicrobial resistance patterns. During 2007, emergence of fluoroquinolone-resistant *N. gonorrhoeae* in the United States prompted CDC to cease recommending fluoroquinolones for gonorrhea treatment, leaving cephalosporins as the only remaining class of antimicrobials available for gonorrhea treatment in the United States (*850*). Reflecting concern about emerging gonococcal resistance, CDC’s 2010 STD treatment guidelines recommended dual therapy for gonorrhea with a cephalosporin plus either azithromycin or doxycycline, even if NAAT for *C. trachomatis* was negative at the time of treatment (*851*). However, during 2006–2011, the minimum concentrations of cefixime needed to inhibit in vitro growth of the *N. gonorrhoeae* strains circulating in the United States and other countries increased, demonstrating that cefixime effectiveness might be waning (*851*). In addition, treatment failures with cefixime or other oral cephalosporins were reported in Asia (*852*–*855*), Europe (*856*–*860*), South Africa (*861*), and Canada (*862*,*863*). During that time, case reports of ceftriaxone treatment failures for pharyngeal infections reported in Australia (*864*,*865*), Japan (*866*), and Europe were concerning (*856*,*867*). Consequently, CDC no longer recommends cefixime as a first-line regimen for gonorrhea treatment in the United States (*868*). Since 2013, the proportion of GISP isolates that demonstrate reduced susceptibility (minimal inhibitory concentration [MIC] ≥2.0 *µ*g/mL) to azithromycin has increased almost tenfold, to 5.1% in 2019 (*141*). Unlike the appearance of ciprofloxacin resistance in the early 2000s, and cefixime reduced-susceptibility isolates during 2010–2011, emergence of azithromycin resistance is not concentrated among certain populations (e.g., MSM in the western United States). Azithromycin has unique pharmacokinetic properties that might predispose to resistance due to its prolonged half-life (*869*,*870*). With the exception of a small cluster of gonorrhea strains with azithromycin resistance and reduced susceptibility to cefixime and ceftriaxone among seven patients during 2016, all gonorrhea strains identified by GISP are susceptible to either or both azithromycin and ceftriaxone or cefixime. In addition, since 2013, antimicrobial stewardship has become an urgent public health concern in the United States as described in *Antimicrobial Resistant Threats in the United States* (*871*). Emergence of azithromycin resistance is not isolated to *N. gonorrhoeae*; it has also been demonstrated in *M. genitalium *and such enteric pathogens as *Shigella* and *Campylobacter* (see *Mycoplasma genitalium*; Proctitis, Proctocolitis, and Enteritis). Finally, concern exists regarding azithromycin treatment efficacy for chlamydia (see Chlamydial Infections).

Dual therapy for gonococcal infection with ceftriaxone and azithromycin recommended in previous guidance might have mitigated emergence of reduced susceptibility to ceftriaxone in *N. gonorrhoeae*; however, concerns regarding potential harm to the microbiome and the effect on other pathogens diminishes the benefits of maintaining dual therapy. Consequently, only ceftriaxone is recommended for treating gonorrhea in the United States (*872*). Clinicians remaining vigilant for treatment failures is paramount, and CDC plans to continue to monitor for changing ceftriaxone MICs until additional antimicrobials or a vaccine is available. In cases in which chlamydial infection has not been excluded, patients should also receive antichlamydial therapy. CDC and state health departments participate in CDC-supported gonorrhea surveillance activities (https://www.cdc.gov/std/gisp) and can provide the most current information regarding gonococcal susceptibility.

Criteria for resistance to cefixime and ceftriaxone have not been defined by the Clinical and Laboratory Standards Institute (CLSI). However, isolates with cefixime or ceftriaxone MICs ≥0.5 *µ*g/mL are considered to have decreased susceptibility (*873*). In the United States, the proportion of isolates in GISP demonstrating decreased susceptibility to ceftriaxone or cefixime has remained low; during 2019, <0.1% of isolates with decreased susceptibility (MIC ≥0.5 *µ*g/mL) to ceftriaxone or cefixime were identified (*141*). Because increasing MICs might predict resistance emergence, GISP established lower cephalosporin MIC threshold values that are lower than the susceptibility breakpoints set by CLSI to provide greater sensitivity in detecting decreasing gonococcal susceptibility for surveillance purposes. The percentage of isolates with cefixime MICs ≥0.25 *µ*g/mL increased from 0.1% during 2006 to 1.4% during 2011 (*851*,*874*) and declined to 0.3% during 2019 (*141*). The percentage of isolates with ceftriaxone MICs ≥0.125 *µ*g/mL increased from <0.1% in 2006 to 0.4% in 2011 and decreased to 0.1% in 2019 (*141*). Isolates with high-level cefixime and ceftriaxone MICs (MICs = 1.5–8.0 *µ*g/mL and MICs = 1.5–4.0 *µ*g/mL, respectively) have been identified in Japan (*866*), France (*867*,*875*), Spain (*876*,*877*), the United Kingdom, and Australia (*878*,*879*). Decreased susceptibility of *N. gonorrhoeae* to cephalosporins and other antimicrobials is expected to continue; state and local surveillance for antimicrobial resistance is crucial for guiding local therapy recommendations (*846*,*847*). Although approximately 3% of all U.S. men who have gonococcal infections are sampled through GISP, surveillance by clinicians also is crucial. Clinicians who diagnose *N. gonorrhoeae* infection in a person with suspected cephalosporin treatment failure should perform culture and AST of relevant clinical specimens, consult an infectious disease specialist or an STD clinical expert (https://www.stdccn.org/render/Public) for guidance in clinical management, and report the case to CDC through state and local public health authorities within 24 hours. Isolates should be saved and sent to CDC through local and state public health laboratory mechanisms. Health departments should prioritize notification and culture evaluation for sexual partners of persons with *N. gonorrhoeae* infection thought to be associated with cephalosporin treatment failure or persons whose isolates demonstrate decreased susceptibility to cephalosporin. Agar dilution is the reference standard and preferred method of antimicrobial susceptibility testing with *N. gonorrhoeae*. Antibiotic gradient strips, such as Etest (bioMérieux), can be used and are considered an acceptable alternative for quantitative antimicrobial susceptibility testing with *N. gonorrhoeae* when manufacturer instructions are followed. Disc diffusion only provides qualitative susceptibility results.

#### Uncomplicated Gonococcal Infection of the Cervix, Urethra, or Rectum

**Recommended Regimen for Uncomplicated Gonococcal Infection of the Cervix, Urethra, or Rectum Among Adults and Adolescents**
**Ceftriaxone** 500 mg* IM in a single dose for persons weighing <150 kg
If chlamydial infection has not been excluded, treat for chlamydia with doxycycline 100 mg orally 2 times/day for 7 days.
* For persons weighing ≥150 kg, 1 g ceftriaxone should be administered.

Although clinical data confirm that a single injection of ceftriaxone 250 mg is >99% (95% confidence interval [CI]: 97.6%–99.7%) effective in curing anogenital gonorrhea of circulating isolates (MIC = 0.03 *µ*g/mL), a higher dose is likely necessary for isolates with elevated MICs (*880*,*881*). Effective treatment of uncomplicated urogenital gonorrhea with ceftriaxone requires concentrations higher than the strain MIC for approximately 24 hours; although individual variability exists in the pharmacokinetics of ceftriaxone, a 500-mg dose of ceftriaxone is expected to achieve in approximately 50 hours MIC >0.03 *µ*g/mL (*880*,*881*). The pharmacokinetics of ceftriaxone might be different in the pharynx with longer times higher than the strain MIC likely needed to prevent selection of mutant strains in the pharynx (*882*).

Single-dose injectable cephalosporin regimens, other than ceftriaxone, that are safe and have been effective against uncomplicated urogenital and anorectal gonococcal infections in the past include ceftizoxime (500 mg IM), cefoxitin (2 g IM with probenecid 1 g orally), and cefotaxime (500 mg IM). None of these injectable cephalosporins offer any advantage over ceftriaxone 250 mg for urogenital infection, and efficacy for pharyngeal infection is less certain (*883*,*884*). Because the ceftriaxone dose has been increased and the pharmacokinetics of other cephalosporins have not been evaluated, these dosing regimens might be at a disadvantage over ceftriaxone 500 mg.

**Alternative Regimens if Ceftriaxone Is Not Available**
**Gentamicin **240 mg IM in a single dose
*plus*
**Azithromycin** 2 g orally in a single dose
*or*
Cefixime* 800 mg orally in a single dose
* If chlamydial infection has not been excluded, providers should treat for chlamydia with doxycycline 100 mg orally 2 times/day for 7 days.

In one clinical trial, dual treatment with single doses of IM gentamicin 240 mg plus oral azithromycin 2 g cured 100% of cases (lower one-sided 95% CI bound: 98.5%) and can be considered an alternative to ceftriaxone for persons with cephalosporin allergy (*885*). This trial was not powered enough to provide reliable estimates of the efficacy of these regimens for treatment of rectal or pharyngeal infection; however, this regimen cured the few extragenital infections among study participants. Notably, gastrointestinal adverse events, primarily vomiting <1 hour after dosing, occurred among 3%–4% of persons treated with gentamicin plus azithromycin, necessitating retreatment with ceftriaxone and azithromycin. A similar trial that studied gentamicin 240 mg plus azithromycin 1 g determined lower cure rates at extragenital sites; 80% (95% CI: 72%–88%) of pharyngeal and 90% (95% CI: 84%–95%) of rectal infections were cured with this regimen (*886*). Gemifloxacin plus azithromycin has been studied and is no longer recommended as an alternative regimen because of limited availability, cost, and antimicrobial stewardship concerns (*885*).

An 800-mg oral dose of cefixime should be considered only as an alternative cephalosporin regimen because it does not provide as high, nor as sustained, bactericidal blood levels as a 500-mg IM dose of ceftriaxone. Furthermore, it demonstrates limited efficacy for treatment of pharyngeal gonorrhea (92.3% cure; 95% CI: 74.9%–99.1%); in older clinical studies, cefixime cured 97.5% of uncomplicated urogenital and anorectal gonococcal infections (95% CI: 95.4%–99.8%) (*883*,*884*). The increase in the prevalence of isolates obtained through GISP with elevated cefixime MICs might indicate early stages of development of clinically significant gonococcal resistance to cephalosporins. Changes in cefixime MICs can result in decreasing effectiveness of cefixime for treating urogenital gonorrhea. Furthermore, as cefixime becomes less effective, continued used of cefixime might hasten the development of resistance to ceftriaxone, a safe, well-tolerated, injectable cephalosporin and the last antimicrobial known to be highly effective in a single dose for treatment of gonorrhea at all anatomic infection sites. Other oral cephalosporins (e.g., cefpodoxime and cefuroxime) are not recommended because of inferior efficacy and less favorable pharmacodynamics (*883*).

Monotherapy with azithromycin 2 g orally as a single dose has been demonstrated to be 99.2% effective against uncomplicated urogenital gonorrhea (95% CI: 97.3%–99.9%) (*883*). However, monotherapy is not recommended because of concerns about the ease with which *N. gonorrhoeae* can develop resistance to macrolides, the high proportion of isolates with azithromycin decreased susceptibility, and documented azithromycin treatment failures (*859*). Strains of *N. gonorrhoeae* circulating in the United States are not adequately susceptible to penicillin, tetracycline, and older macrolides (e.g., erythromycin), and thus use of these antimicrobials cannot be recommended.

Spectinomycin is effective (98.2% in curing uncomplicated urogenital and anorectal gonococcal infections) but has poor efficacy for pharyngeal infections (*883*,*887*). It is unavailable in the United States, and the gentamicin alternative regimen has replaced the need for spectinomycin, if a cephalosporin allergy exists, in the United States.

#### Uncomplicated Gonococcal Infection of the Pharynx

The majority of gonococcal infections of the pharynx are asymptomatic and can be relatively common among certain populations (*800*,*801*,*888*–*890*). Although these infections rarely cause complications, they have been reported to be a major source of community transmission and might be a driver of antimicrobial resistance (*891*,*892*). Gonococcal infections of the pharynx are more difficult to eradicate than infections at urogenital and anorectal sites (*862*). Few antimicrobial regimens reliably cure >90% of gonococcal pharyngeal infections (*883*,*884*). Providers should ask their patients with urogenital or rectal gonorrhea about oral sexual exposure; if reported, pharyngeal testing should be performed.

**Recommended Regimen for Uncomplicated Gonococcal Infection of the Pharynx Among Adolescents and Adults**
**Ceftriaxone **500 mg* IM in a single dose for persons weighing <150 kg
* For persons weighing ≥150 kg, 1 g ceftriaxone should be administered.

If chlamydial infection is identified when pharyngeal gonorrhea testing is performed, treat for chlamydia with doxycycline 100 mg orally 2 times/day for 7 days. No reliable alternative treatments are available for pharyngeal gonorrhea. For persons with an anaphylactic or other severe reaction (e.g., Stevens Johnson syndrome) to ceftriaxone, consult an infectious disease specialist for an alternative treatment recommendation.

##### Other Management Considerations

To maximize adherence with recommended therapies and reduce complications and transmission, medication for gonococcal infection should be provided on-site and directly observed. If medications are unavailable when treatment is indicated, linkage to an STI treatment facility should be provided for same-day treatment. To minimize disease transmission, persons treated for gonorrhea should be instructed to abstain from sexual activity for 7 days after treatment and until all sex partners are treated (7 days after receiving treatment and resolution of symptoms, if present). All persons who receive a diagnosis of gonorrhea should be tested for other STIs, including chlamydia, syphilis, and HIV. Those persons whose HIV test results are negative should be offered HIV PrEP.

##### Follow-Up

A test of cure (i.e., repeat testing after completion of therapy) is unnecessary for persons who receive a diagnosis of uncomplicated urogenital or rectal gonorrhea who are treated with any of the recommended or alternative regimens. Any person with pharyngeal gonorrhea should return 7–14 days after initial treatment for a test of cure by using either culture or NAAT; however, testing at 7 days might result in an increased likelihood of false-positive tests. If the NAAT is positive, effort should be made to perform a confirmatory culture before retreatment, especially if a culture was not already collected. All positive cultures for test of cure should undergo antimicrobial susceptibility testing. Symptoms that persist after treatment should be evaluated by culture for *N. gonorrhoeae* (with or without simultaneous NAAT) and antimicrobial susceptibility. Persistent urethritis, cervicitis, or proctitis also might be caused by other organisms (see Urethritis; Cervicitis; Proctitis).

A high prevalence of *N. gonorrhoeae* infection has been observed among men and women previously treated for gonorrhea (*137*,*753*,*754*,*893*). The majority of these infections result from reinfection caused by failure of sex partners to receive treatment or the initiation of sexual activity with a new infected partner, indicating a need for improved patient education and treatment of sex partners. Men or women who have been treated for gonorrhea should be retested 3 months after treatment regardless of whether they believe their sex partners were treated; scheduling the follow-up visit at the time of treatment is encouraged. If retesting at 3 months is not possible, clinicians should retest whenever persons next seek medical care <12 months after initial treatment.

##### Management of Sex Partners

Recent sex partners (i.e., persons having sexual contact with the infected patient <60 days preceding onset of symptoms or gonorrhea diagnosis) should be referred for evaluation, testing, and presumptive treatment. If the patient’s last potential sexual exposure was >60 days before onset of symptoms or diagnosis, the most recent sex partner should be treated.

If health department partner-management strategies (e.g., disease intervention specialists) are impractical or unavailable for persons with gonorrhea and partners’ access to prompt clinical evaluation and treatment is limited, EPT can be delivered to the partner by the patient or a collaborating pharmacy as permitted by law (see Partner Services). Treatment of the sexual partner with cefixime 800 mg as a single dose is recommended, provided that concurrent chlamydial infection has been excluded. If a chlamydia test result has not been documented, the partner may be treated with a single dose of oral cefixime 800 mg plus oral doxycycline 100 mg 2 times/day for 7 days. If adherence with multiday dosing is a considerable concern, azithromycin 1 g can be considered but has lower treatment efficacy among persons with rectal chlamydia (see Chlamydial Infections). Provision of medication by EPT should be accompanied by written materials (*125*,*127*) for educating partners about gonorrhea, their exposure to gonorrhea, and the importance of therapy. These materials should also educate partners about seeking clinical evaluation for adverse reactions or complications and general follow-up when able. Educational materials for female partners should include information about the importance of seeking medical evaluation for PID, especially if symptomatic; undertreatment of PID among female partners and missed opportunities for diagnosing other STIs among women are of concern. MSM with gonorrhea have a high risk for coexisting infections (especially undiagnosed HIV) among their partners, and they might have partners without HIV who could benefit from PrEP. Data are also limited regarding the effectiveness of EPT in reducing persistent or recurrent gonorrhea among MSM (*133*,*135*); thus, shared clinical decision-making regarding EPT for MSM is recommended (see Partner Services). To avoid reinfection, sex partners should be instructed to abstain from condomless sexual intercourse for 7 days after they and their sex partners have completed treatment and after resolution of symptoms, if present.

#### Suspected Cephalosporin Treatment Failure

Cephalosporin treatment failure is the persistence of *N. gonorrhoeae* infection despite recommended cephalosporin treatment; such failure is indicative of infection with cephalosporin-resistant gonorrhea among persons whose partners were treated and whose risk for reinfection is low. Suspected treatment failure has been reported among persons receiving oral and injectable cephalosporins (*852*–*855*,*857*,*859*,*861*,*863*,*864*,*867*,*875*,*894*). Treatment failure should be considered for persons whose symptoms do not resolve within 3–5 days after recommended treatment and report no sexual contact during the posttreatment follow-up period and persons with a positive test of cure (i.e., positive culture >72 hours or positive NAAT >7 days after receiving recommended treatment) when no sexual contact is reported during the posttreatment follow-up period (*874*). Treatment failure should also be considered for persons who have a positive culture on test of cure, if obtained, if evidence exists of decreased susceptibility to cephalosporins on antimicrobial susceptibility testing, regardless of whether sexual contact is reported during the posttreatment follow-up period.

The majority of suspected treatment failures in the United States are likely to be reinfections rather than actual treatment failures (*137*,*753*,*754*,*894*). However, in cases in which reinfection is unlikely and treatment failure is suspected, before retreatment, relevant clinical specimens should be obtained for culture (preferably with simultaneous NAAT) and antimicrobial susceptibility testing if *N. gonorrhoeae* is isolated. Phenotypic antimicrobial susceptibility testing should be performed by using Etest or agar dilution. All isolates of suspected treatment failures should be sent to CDC for antimicrobial susceptibility testing by agar dilution; local laboratories should store isolates for possible further testing if needed. Testing or storage of specimens or isolates should be facilitated by the state or local health department according to local public health protocol. Instructions for shipping isolates to CDC are available at https://www.cdc.gov/std/gonorrhea/arg/specimen_shipping_instructions1-29-08.pdf.

For persons with suspected cephalosporin treatment failure, the treating clinician should consult an infectious disease specialist, the National Network of STD Clinical Prevention Training Center clinical consultation line (https://www.stdccn.org/render/Public), the local or state health department STI program, or CDC (telephone: 800-232-4636) for advice about obtaining cultures, antimicrobial susceptibility testing, and treatment. Suspected treatment failure should be reported to CDC through the local or state health department <24 hours after diagnosis.

Patients with suspected treatment failures should first be retreated routinely with the initial regimen used (ceftriaxone 500 mg IM), with the addition of doxycycline if chlamydia infection exists, because reinfections are more likely than actual treatment failures. However, in situations with a higher likelihood of treatment failure than reinfection, relevant clinical specimens should be obtained for culture (preferably with simultaneous NAAT) and antimicrobial susceptibility testing before retreatment. Dual treatment with single doses of IM gentamicin 240 mg plus oral azithromycin 2 g can be considered, particularly when isolates are identified as having elevated cephalosporin MICs (*885*,*886*,*895*). Persons with suspected treatment failure after treatment with the alternative regimen (cefixime or gentamicin) should be treated with ceftriaxone 500 mg as a single IM dose or as a single dose with or without an antichlamydial agent on the basis of chlamydia infection status. A test of cure at relevant clinical sites should be obtained 7–14 days after retreatment; culture is the recommended test, preferably with simultaneous NAAT, and antimicrobial susceptibility testing of *N. gonorrhoeae* if isolated. Clinicians should ensure that the patients’ sex partners from the preceding 60 days are evaluated promptly with culture and presumptively treated by using the same regimen used for the patients.

#### Special Considerations

##### Drug Allergy, Intolerance, and Adverse Reactions

The risk for penicillin cross-reactivity is highest with first-generation cephalosporins but is rare (<1%) with third-generation cephalosporins (e.g., ceftriaxone and cefixime) (*631*,*680*,*896*). Clinicians should first thoroughly assess a patient’s allergy history, including type of reaction, associated medications, and previous prescription records. If IgE-mediated penicillin allergy is strongly suspected, dual treatment with single doses of IM gentamicin 240 mg plus oral azithromycin 2 g can be administered (*885*,*886*). If a patient is asymptomatic and the treating facility is able to perform gyrase A (*gyrA*) testing to identify ciprofloxacin susceptibility (wild type), oral ciprofloxacin 500 mg in a single dose can be administered. Providers treating persons with IgE-mediated cephalosporin or penicillin allergy should refer to the section of these guidelines regarding evaluation (see Management of Persons Who Have a History of Penicillin Allergy).

##### Pregnancy

Pregnant women infected with *N. gonorrhoeae* should be treated with ceftriaxone 500 mg in a single IM dose plus treatment for chlamydia if infection has not been excluded. When cephalosporin allergy or other considerations preclude treatment with this regimen, consultation with an infectious disease specialist or an STD clinical expert is recommended (https://www.stdccn.org/render/Public). Gentamicin use is cautioned during pregnancy because of risk for neonatal birth defects, nephrotoxicity, or ototoxicity (*897*).

##### HIV Infection

Persons who have gonorrhea and HIV infection should receive the same treatment regimen as those who do not have HIV.

#### Gonococcal Conjunctivitis

In the only published study of the treatment regarding gonococcal conjunctivitis among adults, all 12 study participants responded to a single 1-g IM injection of ceftriaxone (*898*). Because gonococcal conjunctivitis is uncommon and data regarding treatment of gonococcal conjunctivitis among adults are limited, consultation with an infectious disease specialist should be considered.

**Recommended Regimen for Gonococcal Conjunctivitis Among Adolescents and Adults**
**Ceftriaxone** 1 g IM in a single dose
Providers should consider one-time lavage of the infected eye with saline solution.

##### Management of Sex Partners

Patients should be instructed to refer their sex partners for evaluation and treatment (see Gonococcal Infections, Management of Sex Partners).

#### Disseminated Gonococcal Infection

Infrequently, *N. gonorrhoeae* can cause disseminated infection. Disseminated gonococcal infection (DGI) frequently results in petechial or pustular acral skin lesions, asymmetric polyarthralgia, tenosynovitis, or oligoarticular septic arthritis (*899*–*901*). Rarely, DGI is complicated by perihepatitis associated with gonococcal PID, endocarditis, or meningitis. Certain strains of *N. gonorrhoeae* that cause DGI can cause minimal genital inflammation, and urogenital or anorectal infections are often asymptomatic among DGI patients. If DGI is suspected, NAATs or culture specimens from all exposed urogenital and extragenital sites should be collected and processed, in addition to disseminated sites of infection (e.g., skin, synovial fluid, blood, or CSF). All *N. gonorrhoeae* isolates should be tested for antimicrobial susceptibility. Risk factors for dissemination have included female sex, menstruation, pregnancy, and terminal complement deficiency (*899*); however, reports are increasing among men (*900*,*901*). Persons receiving eculizumab, a monoclonal antibody that inhibits terminal complement activation, also might be at higher risk for DGI (*902*).

Hospitalization and consultation with an infectious disease specialist are recommended for initial therapy, especially for persons who might not comply with treatment, have an uncertain diagnosis, or have purulent synovial effusions or other complications. Examination for clinical evidence of endocarditis and meningitis should be performed.

##### Treatment of Arthritis and Arthritis-Dermatitis Syndrome

**Recommended Regimen for Gonococcal-Related Arthritis and Arthritis-Dermatitis Syndrome**
**Ceftriaxone** 1 g IM or IV every 24 hours
If chlamydial infection has not been excluded, providers should treat for chlamydia with doxycycline 100 mg orally 2 times/day for 7 days.
**Alternative Regimens**
**Cefotaxime** 1 g IV every 8 hours
*or*
**Ceftizoxime **1 g every 8 hours
If chlamydial infection has not been excluded, providers should treat for chlamydia with doxycycline 100 mg orally 2 times/day for 7 days.

When treating for the arthritis-dermatitis syndrome, the provider can switch to an oral agent guided by antimicrobial susceptibility testing 24–48 hours after substantial clinical improvement, for a total treatment course of >7 days.

##### Treatment of Gonococcal Meningitis and Endocarditis

**Recommended Regimen for Gonococcal Meningitis and Endocarditis**
**Ceftriaxone** 1–2 g IV every 24 hours
If chlamydial infection has not been excluded, providers should treat for chlamydia with doxycycline 100 mg orally 2 times/day for 7 days.

No recent studies have been published regarding treatment of DGI involving the CNS or cardiovascular system. The duration of treatment for DGI in these situations has not been systematically studied and should be determined in consultation with an infectious disease specialist. Treatment for DGI should be guided by the results of antimicrobial susceptibility testing. Length of treatment should be determined based on clinical presentation. Therapy for meningitis should be continued with recommended parenteral therapy for 10–14 days. Parenteral antimicrobial therapy for endocarditis should be administered for >4 weeks. Treatment of gonococcal perihepatitis should be managed in accordance with the recommendations for PID in these guidelines.

##### Management of Sex Partners

Gonococcal infection frequently is asymptomatic among sex partners of persons who have DGI. Providers should instruct patients to refer partners with whom they have had sexual contact during the previous 60 days for evaluation, testing, and presumptive treatment (see Gonococcal Infections, Management of Sex Partners).

### Gonococcal Infection Among Neonates

Prenatal screening and treatment of pregnant women for gonorrhea is the best method for preventing *N. gonorrhoeae* infection among neonates. Gonococcal infection among neonates results from perinatal exposure to the mother’s infected cervix. It is usually an acute illness that manifests 2–5 days after birth. Prevalence of infection among neonates depends on the prevalence of infection among pregnant women and whether pregnant women are screened and treated for gonorrhea during pregnancy. The most severe manifestations of *N. gonorrhoeae* infection among neonates are ophthalmia neonatorum and sepsis, which can include arthritis and meningitis. Less severe manifestations include rhinitis, vaginitis, urethritis, and scalp infection at sites of previous fetal monitoring.

#### Preventing Ophthalmia Neonatorum Caused by *N. gonorrhoeae*

Ocular prophylaxis and preventive gonorrhea screening and treatment of infected pregnant women are especially important because ophthalmia neonatorum can result in perforation of the globe of the eye and blindness (*903*). Ocular prophylaxis for gonococcal ophthalmia neonatorum has a long history of preventing sight-threatening gonococcal ocular infections. Cases in the United States are uncommon, which is likely attributable to gonorrhea screening programs for women, including pregnant women, that have contributed substantially to reduction in ophthalmia neonatorum (*904*). Neonatal ocular prophylaxis with erythromycin, the only agent available in the United States, is required by law in most states and is recommended because of safety, low cost, and ease of administration. It can contribute to preventing gonococcal blindness because not all pregnant women are screened for gonorrhea. The USPSTF recommends ocular prophylaxis with erythromycin ointment for all newborns <24 hours after birth (*903*). In addition to continuing routine ocular prophylaxis, prevention should focus on prenatal screening for *N. gonorrhoeae,* including

- screening pregnant women at risk (e.g., women aged <25 years and those aged ≥25 years who have a new sex partner, more than one sex partner, a sex partner with concurrent partners, a sex partner who has an STI, or live in a community with high rates of gonorrhea) for *N. gonorrhoeae* infection at the first prenatal visit;
- treating all pregnant women with *N. gonorrhoeae* infection during pregnancy and retesting in 3 months, in the third trimester or at time of delivery (sex partners should be tested and treated);
- retesting pregnant women in the third trimester who initially tested negative but remained at increased risk for acquiring infection (e.g., women aged <25 years and those aged ≥25 years who have a new sex partner, more than one sex partner, a sex partner with concurrent partners, a sex partner who has an STI, or live in a community with high rates of gonorrhea); and
- screening for gonorrhea at delivery for women not tested during pregnancy and at risk for infection (e.g., women aged <25 years and those aged ≥25 years who have a new sex partner, more than one sex partner, a sex partner with concurrent partners, a sex partner who has an STI, or live in a community with high rates of gonorrhea) or received no prenatal care; providers caring for the mother and the newborn should communicate to ensure follow-up on the results of laboratory tests performed at delivery, and if positive, prompt appropriate treatment of the newborn and mother.

Erythromycin is the only ophthalmic ointment recommended for use among neonates. Silver nitrate and tetracycline ophthalmic ointments are no longer manufactured in the United States, bacitracin is ineffective, and povidone iodine has not been studied adequately (*905*,*906*). Gentamicin ophthalmic ointment has been associated with severe ocular reactions (*907*,*908*). If erythromycin ointment is unavailable, infants at risk for exposure to *N. gonorrhoeae,* especially those born to a mother at risk for gonococcal infection or with no prenatal care, can be administered ceftriaxone 25–50 mg/kg body weight IV or IM, not to exceed 250 mg in a single dose.

**Recommended Regimen to Prevent Ophthalmia Neonatorum Caused by *N. gonorrhoeae***
**Erythromycin** **0.5% ophthalmic ointment** in each eye in a single application at birth

Erythromycin ophthalmic ointment should be instilled into both eyes of neonates as soon as possible after delivery, regardless of whether they are delivered vaginally or by cesarean delivery. Ideally, ointment should be applied by using single-use tubes or ampules rather than multiple-use tubes. If prophylaxis is delayed (i.e., not administered in the delivery room), a monitoring system should be established to ensure that all newborns receive prophylaxis <24 hours after delivery.

##### Diagnostic Considerations

Newborns at increased risk for gonococcal ophthalmia include those who did not receive ophthalmic prophylaxis and whose mothers had no prenatal care, have a history of STIs during pregnancy, or have a history of substance misuse. Gonococcal ophthalmia is strongly suspected when intracellular gram-negative diplococci are identified on Gram stain of conjunctival exudate, justifying presumptive treatment for gonorrhea after appropriate cultures and antimicrobial susceptibility testing for *N. gonorrhoeae* are performed. Presumptive treatment for *N. gonorrhoeae* might be indicated for newborns at increased risk for gonococcal ophthalmia who have increased WBCs (no GNID) in a Gram-stained smear of conjunctival exudate. Nongonococcal causes of neonatal ophthalmia include *Moraxella catarrhalis* and other *Neisseria* species, which are organisms that are indistinguishable from *N. gonorrhoeae* on Gram-stained smear but can be differentiated in the microbiology laboratory.

##### Treatment of Gonococcal Ophthalmia Neonatorum

**Recommended Regimen for Gonococcal Ophthalmia Neonatorum**
**Ceftriaxone** 25–50 mg/kg body weight IV or IM in a single dose, not to exceed 250 mg

One dose of ceftriaxone is adequate therapy for gonococcal ophthalmia. Ceftriaxone should be administered cautiously to neonates with hyperbilirubinemia, especially those born prematurely. Cefotaxime 100 mg/kg body weight IV or IM as a single dose can be administered for those neonates unable to receive ceftriaxone because of simultaneous administration of IV calcium. Topical antibiotic therapy alone is inadequate and unnecessary if systemic treatment is administered.

##### Other Management Considerations

Chlamydial testing should be performed simultaneously from the inverted eyelid specimen (see Ophthalmia Neonatorum Caused by *C. trachomatis*). Newborns who have gonococcal ophthalmia should be evaluated for signs of disseminated infection (e.g., sepsis, arthritis, and meningitis). Newborns who have gonococcal ophthalmia should be managed in consultation with an infectious disease specialist.

##### Management of Mothers and Their Sex Partners

Mothers of newborns with ophthalmia neonatorum caused by *N. gonorrhoeae* should be evaluated, tested, and presumptively treated for gonorrhea, along with their sex partners (see Gonococcal Infection Among Adolescents and Adults).

#### Disseminated Gonococcal Infection and Gonococcal Scalp Abscesses Among Neonates

DGI might present as sepsis, arthritis, or meningitis and is a rare complication of neonatal gonococcal infection. Localized gonococcal infection of the scalp can result from fetal monitoring through scalp electrodes. Detecting gonococcal infection among neonates who have sepsis, arthritis, meningitis, or scalp abscesses requires cultures of blood, CSF, or joint aspirate. Specimens obtained from the conjunctiva, vagina, oropharynx, and rectum are useful for identifying the primary site or sites of infection. Antimicrobial susceptibility testing of all isolates should be performed. Positive Gram-stained smears of abscess exudate, CSF, or joint aspirate provide a presumptive basis for initiating treatment for *N. gonorrhoeae*.

##### Treatment

**Recommended Regimens for Disseminated Gonococcal Infection Among Neonates**
**Ceftriaxone **25–50 mg/kg body weight/day IV or IM in a single daily dose for 7 days, with a duration of 10–14 days if meningitis is documented
*or*
**Cefotaxime **25 mg/kg body weight/day IV or IM every 12 hours for 7 days, with a duration of 10–14 days if meningitis is documented

Ceftriaxone should be administered cautiously to neonates with hyperbilirubinemia, especially those born prematurely. Cefotaxime 100 mg/kg body weight IV or IM as a single dose can be administered for those neonates unable to receive ceftriaxone because of simultaneous administration of IV calcium.

##### Other Management Considerations

Chlamydial testing should be performed simultaneously among neonates with gonococcal infection (see Chlamydial Infection Among Neonates). Neonates who have DGI should be managed in consultation with an infectious disease specialist.

##### Management of Mothers and Their Sex Partners

Mothers of newborns who have DGI or scalp abscesses caused by *N. gonorrhoeae* should be evaluated, tested, and presumptively treated for gonorrhea, along with their sex partners (see Gonococcal Infection Among Adolescents and Adults).

#### Neonates Born to Mothers Who Have Gonococcal Infection

Neonates born to mothers who have untreated gonorrhea are at high risk for infection. Neonates should be tested for gonorrhea at exposed sites (e.g., conjunctiva, vagina, rectum, and oropharynx) and treated presumptively for gonorrhea.

##### Treatment in the Absence of Signs of Gonococcal Infection

**Recommended Regimen for Neonates Without Signs of Gonococcal Infection**
**Ceftriaxone** 20–50 mg/kg body weight IV or IM in a single dose, not to exceed 250 mg

##### Other Management Considerations

Ceftriaxone should be administered cautiously to neonates with hyperbilirubinemia, especially those born prematurely. Cefotaxime 100 mg/kg body weight IV or IM as a single dose can be administered for those neonates unable to receive ceftriaxone because of simultaneous administration of IV calcium. Age-appropriate chlamydial testing should be performed simultaneously among neonates with gonococcal infection (see Chlamydial Infection Among Neonates). Follow-up examination is not required.

##### Management of Mothers and Their Sex Partners

Mothers who have gonorrhea and their sex partners should be evaluated, tested, and presumptively treated for gonorrhea (see Gonococcal Infection Among Adolescents and Adults).

### Gonococcal Infection Among Infants and Children

Sexual abuse is the most frequent cause of gonococcal infection among infants and children (see Sexual Assault or Abuse of Children). For preadolescent girls, vaginitis is the most common manifestation of this infection; gonococcal-associated PID after vaginal infection can be less common among preadolescents than adults. Among sexually abused children, anorectal and pharyngeal infections with *N. gonorrhoeae* are frequently asymptomatic.

#### Diagnostic Considerations

Culture can be used to test urogenital and extragenital sites for girls and boys. NAAT can be used to test for *N. gonorrhoeae* from vaginal and urine specimens from girls and urine for boys (see Sexual Assault or Abuse of Children). Although data regarding NAAT from extragenital sites (rectum and pharynx) among children are more limited, and performance is test dependent, no evidence supports that performance of NAAT for detection of *N. gonorrhoeae* among children differs from that among adults (*553*). Because of the implications of a *N. gonorrhoeae* diagnosis in a child, only validated FDA-cleared NAAT assays should be used with extragenital specimens. Consultation with an expert is necessary before using NAAT to minimize the possibility of cross-reaction with nongonococcal *Neisseria* species and other commensals (e.g., *N. meningitidis*, *Neisseria sicca*, *Neisseria lactamica*, *Neisseria cinerea*, or *M. catarrhalis*) and to ensure correct interpretation of results.

Gram stains are inadequate for evaluating prepubertal children for gonorrhea and should not be used to diagnose or exclude gonorrhea. If evidence of DGI exists, gonorrhea culture and antimicrobial susceptibility testing should be obtained from relevant clinical sites (see Disseminated Gonococcal Infection).

**Recommended Regimen for Uncomplicated Gonococcal Vulvovaginitis, Cervicitis, Urethritis, Pharyngitis, or Proctitis Among Infants and Children Weighing ≤45 kg**
**Ceftriaxone** 25–50 mg/kg body weight IV or IM in a single dose, not to exceed 250 mg IM
**Recommended Regimen for Uncomplicated Gonococcal Vulvovaginitis, Cervicitis, Urethritis, Pharyngitis, or Proctitis Among Children Weighing >45 kg**
**Treat with the regimen recommended for adults** (see Gonococcal Infections)
**Recommended Regimen for Bacteremia or Arthritis Among Children Weighing ≤45 kg **
**Ceftriaxone **50 mg/kg body weight (maximum dose: 2 g) IM or IV in a single dose daily every 24 hours for 7 days
**Recommended Regimen for Bacteremia or Arthritis Among Children Weighing >45** kg
**Ceftriaxone** 1 g IM or IV in a single dose daily every 24 hours for 7 days

#### Other Management Considerations

Follow-up cultures are unnecessary. Only parenteral cephalosporins (i.e., ceftriaxone) are recommended for use among children. All children identified as having gonococcal infections should be tested for *C. trachomatis*, syphilis, and HIV (see Sexual Assault or Abuse of Children).

## Mycoplasma genitalium

*M. genitalium* causes symptomatic and asymptomatic urethritis among men and is the etiology of approximately 15%–20% of NGU, 20%–25% of nonchlamydial NGU, and 40% of persistent or recurrent urethritis (*697*,*909*,*910*). Infection with *C. trachomatis* is common in selected geographic areas (*911*–*913*), although *M. genitalium* is often the sole pathogen. Data are insufficient to implicate *M. genitalium* infection with chronic complications among men (e.g., epididymitis, prostatitis, or infertility). The consequences of asymptomatic infection with *M. genitalium* among men are unknown.

Among women, *M. genitalium* has been associated with cervicitis, PID, preterm delivery, spontaneous abortion, and infertility, with an approximately twofold increase in the risk for these outcomes among women infected with *M. genitalium* (*766*)*. M. genitalium* infections among women are also frequently asymptomatic, and the consequences associated with asymptomatic *M. genitalium* infection are unknown.

*M. genitalium* can be detected among 10%–30% of women with clinical cervicitis (*767*,*770*,*772*,*914*–*916*). The existing evidence between *M. genitalium* and cervicitis is mostly supportive of a causal association. Elevated proinflammatory cytokines have been demonstrated among women with *M. genitalium*, with return to baseline levels after clearance of the pathogen (*917*)*.*

*M. genitalium* is identified in the cervix or endometrium of women with PID more often than in women without PID (*918*–*924*). Prevalence of *M. genitalium* among women with PID ranges from 4% to 22% (*925*,*926*) and was reported as 60% in one study of women with postabortal PID (*918*). The association with PID is supported by early studies among nonhuman primates that determined that endosalpingitis develops after inoculation with *M. genitalium* (*927*). Recent studies evaluating the lower and upper genital tract using highly sensitive *M. genitalium* NAAT assays or the role of *M. genitalium* in histologically defined endometritis have reported significantly elevated risk for PID (*928*). However, most studies of *M. genitalium* and PID, even those that controlled extensively for other infections and behavioral and biologic risk, are cross-sectional. The few prospective studies that have evaluated the role of *M. genitalium* in establishing subsequent PID demonstrated increased PID risk; however, these were not statistically significant associations, often because of a lack of statistical power. No clinical trial data are available that demonstrate that treating *M. genitalium* cervical infection prevents development of PID or endometritis. Although data regarding the benefits of testing women with PID for *M. genitalium* and the importance of directing treatment against this organism are limited, the associations of *M. genitalium* with cervicitis and PID in cross-sectional studies using NAAT testing are consistent (*928*).

Data from case-control serologic studies (*929*–*931*) and a meta-analysis of clinical studies (*766*) indicate a potential role in causing infertility. However, seroassays are suboptimal and inconclusive. Similarly, evidence for a role for *M. genitalium* infection during pregnancy as a cause of perinatal complications, including preterm delivery, spontaneous abortion, or low birthweight, are conflicting because evidence is insufficient to attribute cause (*766*,*932*–*934*). Data are limited regarding ectopic pregnancy and neonatal *M. genitalium* infection (*935*,*936*).

Rectal infection with *M. genitalium* has been reported among 1%–26% of MSM (*937*–*940*) and among 3% of women (*941*). Rectal infections often are asymptomatic, although higher prevalence of *M. genitalium* has been reported among men with rectal symptoms. Similarly, although asymptomatic *M. genitalium* has been detected in the pharynx, no evidence exists of it causing oropharyngeal symptoms or systemic disease.

Urogenital *M. genitalium* infection is associated with HIV among both men and women (*942*–*944*); however, the data are from case-control and cross-sectional studies. Risk for HIV infection is increased among women with *M. genitalium*, and evidence indicates that HIV shedding occurs more often among persons with *M. genitalium* and HIV infection who are not taking ART than among persons without *M. genitalium* (*942*,*944*).

### Antimicrobial Resistance

Resistance to azithromycin has been rapidly increasing and has been confirmed in multiple studies. Prevalence of molecular markers for macrolide resistance, which highly correlates with treatment failure, ranges from 44% to 90% in the United States, Canada, Western Europe, and Australia (*697*,*702*,*945*–*953*). Treatment with azithromycin alone has been reported to select for resistance (*705*,*954*,*955*), with treatment of macrolide-susceptible infections with a 1-g dose of azithromycin resulting in selection of resistant-strain populations in 10%–12% of cases. The prevalence of quinolone resistance markers is much lower (*697*,*956*–*959*). The first clinical treatment failures after moxifloxacin were associated specifically with the S83I mutation in the *parC* gene (*954*,*960*). Prevalence of the S83I mutation in the United States ranges from 0% to 15% (*947*); however, correlation with fluoroquinolone treatment failure is less consistent than that with mutations associated with macrolide resistance (*953*,*961*,*962*). Clinically relevant quinolone resistance often is associated with coexistent macrolide resistance (*954*).

### Diagnostic Considerations

*M. genitalium* is an extremely slow-growing organism. Culture can take up to 6 months, and technical laboratory capacity is limited to research settings. NAAT for *M. genitalium* is FDA cleared for use with urine and urethral, penile meatal, endocervical, and vaginal swab samples (https://www.hologic.com/package-inserts/diagnostic-products/aptima-mycoplasma-genitalium-assay). Molecular tests for macrolide (i.e., azithromycin) or quinolone (i.e., moxifloxacin) resistance markers are not commercially available in the United States. However, molecular assays that incorporate detection of mutations associated with macrolide resistance are under evaluation.

Men with recurrent NGU should be tested for *M. genitalium* using an FDA-cleared NAAT. If resistance testing is available, it should be performed and the results used to guide therapy. Women with recurrent cervicitis should be tested for *M. genitalium*, and testing should be considered among women with PID. Testing should be accompanied with resistance testing, if available. Screening of asymptomatic *M. genitalium* infection among women and men or extragenital testing for *M. genitalium* is not recommended. In clinical practice, if testing is unavailable, *M. genitalium* should be suspected in cases of persistent or recurrent urethritis or cervicitis and considered for PID.

### Treatment

*M. genitalium* lacks a cell wall, and thus antibiotics targeting cell-wall biosynthesis (e.g., ß-lactams including penicillins and cephalosporins) are ineffective against this organism. Because of the high rates of macrolide resistance with treatment failures (*707*) and efficient selection of additional resistance, a 1-g dose of azithromycin should not be used.

Two-stage therapy approaches, ideally using resistance-guided therapy, are recommended for treatment. Resistance-guided therapy has demonstrated cure rates of >90% and should be used whenever possible (*759*,*963*); however, it requires access to macrolide-resistance testing. As part of this approach, doxycycline is provided as initial empiric therapy, which reduces the organism load and facilitates organism clearance, followed by macrolide-sensitive *M.* *genitalium* infections treated with high-dose azithromycin; macrolide-resistant infections are treated with moxifloxacin (*964*,*965*).

**Recommended Regimens if *M. genitalium* Resistance Testing Is Available**
**If macrolide sensitive: Doxycycline **100 mg orally 2 times/day for 7 days, followed by **azithromycin** 1 g orally initial dose, followed by 500 mg orally once daily for 3 additional days (2.5 g total)
**If macrolide resistant: Doxycycline **100 mg orally 2 times/day for 7 days followed by **moxifloxacin** 400 mg orally once daily for 7 days
**Recommended Regimen if *M. genitalium* Resistance Testing Is Not Available**
**If *M. genitalium *is detected by an FDA-cleared NAAT: Doxycycline** 100 mg orally 2 times/day for 7 days, followed by **moxifloxacin** 400 mg orally once daily for 7 days

Although the majority of *M. genitalium* strains are sensitive to moxifloxacin, resistance has been reported, and adverse side effects and cost should be considered with this regimen. In settings without access to resistance testing and when moxifloxacin cannot be used, an alternative regimen can be considered, based on limited data: doxycycline 100 mg orally 2 times/day for 7 days, followed by azithromycin (1 g orally on day 1 followed by 500 mg once daily for 3 days) and a test of cure 21 days after completion of therapy (*963*). Because of the high prevalence of macrolide resistance and high likelihood of treatment failure, this regimen should be used only when a test of cure is possible, and no other alternatives exist. If symptomatic treatment failure or a positive test of cure occurs after this regimen, expert consultation is recommended. Data are limited regarding use of minocycline in instances of treatment failure (*966*).

Recommended PID treatment regimens are not effective against *M. genitalium*. Initial empiric therapy for PID, which includes doxycycline 100 mg orally 2 times/day for 14 days, should be provided at the time of presentation for care. If *M. genitalium* is detected, a regimen of moxifloxacin 400 mg orally once daily for 14 days has been effective in eradicating the organism. Nevertheless, no data have been published that assess the benefits of testing women with PID for *M. genitalium,* and the importance of directing treatment against this organism is unknown.

### Follow-Up

Test of cure is not recommended for asymptomatic persons who received treatment with a recommended regimen. In settings in which *M. genitalium* testing is available, persons with persistent urethritis, cervicitis, or PID accompanied by detection of *M. genitalium* should be treated with moxifloxacin.

### Management of Sex Partners

Recent studies report a high concordance of *M. genitalium* among partners of males, females, and MSM; however, no studies have determined whether reinfection is reduced with partner treatment (*940*,*967*,*968*). Sex partners of patients with symptomatic *M. genitalium* infection can be tested, and those with a positive test can be treated to possibly reduce the risk for reinfection. If testing the partner is not possible, the antimicrobial regimen that was provided to the patient can be provided.

### Special Considerations

#### HIV Infection

Persons who have *M. genitalium* and HIV infection should receive the same treatment regimen as those persons without HIV.

## Diseases Characterized by Vulvovaginal Itching, Burning, Irritation, Odor, or Discharge

The majority of women will have a vaginal infection, characterized by discharge, itching, burning, or odor, during their lifetime. With the availability of complementary and alternative therapies and over-the-counter medications for candidiasis, symptomatic women often seek these products before or in addition to an evaluation by a medical provider.

Obtaining a medical history alone has been reported to be insufficient for accurate diagnosis of vaginitis and can lead to inappropriate administration of medication (*969*). Therefore, a careful history, examination, and laboratory testing to determine the etiology of any vaginal symptoms are warranted. Information regarding sexual behaviors and practices, sex of sex partners, menses, vaginal hygiene practices (e.g., douching), and self-treatment with oral and intravaginal medications or other products should be elicited. The infections most frequently associated with vaginal symptoms are BV (i.e., replacement of the vaginal flora by an overgrowth of anaerobic bacteria including *G. vaginalis*, *Prevotella* *bivia*, *A. vaginae*, *Megasphaera* type 1, and numerous other fastidious or uncultivated anaerobes), trichomoniasis, and vulvovaginal candidiasis (VVC). Cervicitis can also cause an abnormal vaginal discharge. Although VVC is usually not sexually transmitted, it is included in this section because it is frequently diagnosed among women who have vaginal symptoms or are being evaluated for an STI.

Multiple diagnostic methods are available for identifying the etiology of vaginal symptoms. Clinical laboratory testing can identify the vaginitis cause in the majority of women and is discussed in detail in the sections of this report dedicated to each condition. In the clinician’s office, the cause of vaginal symptoms can often be determined by pH, a potassium hydroxide (KOH) test, and microscopic examination of a wet mount of fresh samples of vaginal discharge. The pH of the vaginal secretions can be measured by pH paper; an elevated pH (i.e., >4.5) is common with BV or trichomoniasis (although trichomoniasis can also be present with a normal vaginal pH). Because pH testing is not highly specific, vaginal discharge should be further examined microscopically by first diluting one sample in 1 or 2 drops of 0.9% normal saline solution on one slide and a second sample in 10% KOH solution (samples that emit an amine odor immediately upon application of KOH suggest BV or trichomoniasis). Coverslips are then placed on the slides, and they are examined under a microscope at low and high power. The saline-solution specimen might display motile trichomonads or clue cells (i.e., epithelial cells with borders obscured by small anaerobic bacteria), which are characteristic of BV. The KOH specimen typically is used to identify hyphae or blastospores observed with candidiasis. However, absence of trichomonads in saline or fungal elements in KOH samples does not rule out these infections because the sensitivity of microscopy is approximately 50% compared with NAAT (trichomoniasis) or culture (yeast) (*670*). Presence of WBCs without evidence of trichomonads or yeast might also indicate cervicitis (see Cervicitis).

In settings where pH paper, KOH, and microscopy are unavailable, a broad range of clinical laboratory tests, described in the diagnosis section for each disease, can be used. Presence of objective signs of vulvovaginal inflammation in the absence of vaginal pathogens after laboratory testing indicates the possibility of mechanical, chemical, allergic, or other noninfectious causes of vulvovaginal signs or symptoms. For women with persistent symptoms and no clear etiology, referral to a specialist should be considered.

### Bacterial Vaginosis

BV is a vaginal dysbiosis resulting from replacement of normal hydrogen peroxide and lactic-acid–producing *Lactobacillus* species in the vagina with high concentrations of anaerobic bacteria, including *G. vaginalis*, *Prevotella* species, *Mobiluncus* species, *A. vaginae*, and other BV-associated bacteria. A notable feature is the appearance of a polymicrobial biofilm on vaginal epithelial cells (*970*). Certain women experience transient vaginal microbial changes, whereas others experience them for longer intervals (*971*). BV is a highly prevalent condition and the most common cause of vaginal discharge worldwide (*972*). However, in a nationally representative survey, the majority of women with BV were asymptomatic (*310*).

BV is associated with having multiple male sex partners, female partners, sexual relationships with more than one person (*973*), a new sex partner, lack of condom use (*974*), douching (*975*,*976*), and HSV-2 seropositivity (*977*). Male circumcision reduces the risk for BV among women (*978*). In addition, BV prevalence increases during menses (*979*,*980*). Women who have never been sexually active are rarely affected (*981*). The cause of the microbial alteration that precipitates BV is not fully understood, and whether BV results from acquisition of a single sexually transmitted pathogen is unknown. BV prevalence has been reported to increase among women with copper-containing IUDs (*972*,*982*). Hormonal contraception does not increase risk for BV (*983*) and might protect against BV development (*983*,*984*). Vitamin D deficiency has not been reported to be a risk factor for BV (*985*).

Women with BV are at increased risk for STI acquisition, such as HIV, *N. gonorrhoeae*, *C. trachomatis*, *T. vaginalis* (*977*), *M. genitalium* (*986*), HPV (*987*), and HSV-2 (*988*); complications after gynecologic surgery; complications of pregnancy; and recurrence of BV (*971*,*989*–*991*). BV also increases HIV infection acquisition (*992*) because specific BV-associated bacteria can increase susceptibility to HIV (*993*,*994*) and the risk for HIV transmission to male sex partners (*187*). Evaluation of short-term valacyclovir suppression among women with HSV-2 did not decrease the risk for BV, despite effective suppression of HSV-2 (*995*).

Although BV-associated bacteria can be identified on male genitalia (*996*,*997*), treatment of male sex partners has not been beneficial in preventing the recurrence of BV (*998*). Among WSW, a high level of BV concordance occurs between sex partners (*292*); however, no studies have evaluated treatment of female sex partners of WSW to prevent BV recurrence.

#### Diagnostic Considerations

BV can be diagnosed by using clinical criteria (i.e., Amsel’s diagnostic criteria) (*999*) or by determining the Nugent score from a vaginal Gram stain (*1000*). Vaginal Gram stain, considered the reference standard laboratory method for diagnosing BV, is used to determine the relative concentration of lactobacilli (i.e., long gram-positive rods), small gram-negative and gram-variable rods (i.e., *G. vaginalis* or *Bacteroides*), and curved gram-negative rods (i.e., *Mobiluncus*) characteristic of BV. A Nugent score of 0–3 is consistent with a *Lactobacillus*-predominant vaginal microbiota, 4–6 with intermediate microbiota (emergence of *G. vaginalis*), and 7–10 with BV. Clinical diagnosis of BV by Amsel criteria requires at least three of the following four symptoms or signs:

- Homogeneous, thin discharge (milklike consistency) that smoothly coats the vaginal walls
- Clue cells (e.g., vaginal epithelial cells studded with adherent bacteria) on microscopic examination
- pH of vaginal fluid >4.5
- A fishy odor of vaginal discharge before or after addition of 10% KOH (i.e., the whiff test)

Detection of at least three Amsel criteria has been correlated with results by Gram stain (*1001*). The sensitivity and specificity of the Amsel criteria are 37%–70% and 94%–99%, respectively, compared with the Nugent score (*1002*).

In addition to the Amsel criteria, multiple POC tests are available for BV diagnosis. The Osom BV Blue test (Sekisui Diagnostics) detects vaginal sialidase activity (*1003*,*1004*). The Affirm VP III (Becton Dickinson) is an oligonucleotide probe test that detects high concentrations of *G. vaginalis* nucleic acids (>5 x 10^5^ CFU of *G. vaginalis/*mL of vaginal fluid) for diagnosing BV, *Candida* species, and *T. vaginalis.* This test has been reported to be most useful for symptomatic women in conjunction with vaginal pH measurement and presence of amine odor (sensitivity of 97%); specificity is 81% compared with Nugent. Finally, the FemExam Test Card (Cooper Surgical) measures vaginal pH, presence of trimethylamine (a metabolic by-product of *G. vaginalis*), and proline aminopeptidase (*1005*). Sensitivity is 91% and specificity is 61%, compared with Nugent. This test has primarily been studied in resource-poor settings (*1005*), and although it has been reported to be beneficial compared with syndromic management, it is not a preferred diagnostic method for BV diagnosis.

Multiple BV NAATs are available for BV diagnosis among symptomatic women (*1002*). These tests are based on detection of specific bacterial nucleic acids and have high sensitivity and specificity for BV (i.e., *G. vaginalis*, *A. vaginae*, BVAB2, or *Megasphaera* type 1) (*1006*) and certain lactobacilli (i.e., *Lactobacillus crispatus*, *Lactobacillus jensenii*, and *Lactobacillus gasseri*). They can be performed on either clinician- or self-collected vaginal specimens with results available in <24 hours, depending on the availability of the molecular diagnostic platform (*1002*). Five quantitative multiplex PCR assays are available: Max Vaginal Panel (Becton Dickinson) (*1007*), Aptima BV (Hologic), NuSwab VG (LabCorp) (*1008*), OneSwab BV Panel PCR with Lactobacillus Profiling by qPCR (Medical Diagnostic Laboratories) (*1009*), and SureSwab BV (Quest Diagnostics). Two of these assays are FDA cleared (BD Max Vaginal Panel and Aptima BV), and the other three are laboratory-developed tests.

The Max Vaginal Panel provides results by an algorithmic analysis of molecular DNA detection of *Lactobacillus* species (*L. crispatus* and *L. jensenii*) in addition to *G. vaginalis*, *A. vaginae*, BVAB2, and *Megasphaera* type 1. This test has 90.5% sensitivity and 85.8% specificity for BV diagnosis, compared with Amsel criteria and Nugent score. It also provides results for *Candida* species and *T. vaginalis*. The Aptima BV detects *G. vaginalis*, *A. vaginae*, and certain *Lactobacillus* species including *L. crispatus*, *L. jensenii*, and *L. gasseri*, with sensitivity and specificity ranging from 95.0% to 97.3% and 85.8% to 89.6%, respectively (using either clinician- or patient-collected vaginal swabs). The three laboratory-developed tests (NuSwab VG, OneSwab BV Panel PCR with Lactobacillus Profiling by qPCR, and SureSwab BV) have to be internally validated before use for patient care yet have good sensitivity and specificity, similar to FDA-cleared assays. BV NAATs should be used among symptomatic women only (e.g., women with vaginal discharge, odor, or itch) because their accuracy is not well defined for asymptomatic women. Despite the availability of BV NAATs, traditional methods of BV diagnosis, including the Amsel criteria, Nugent score, and the Affirm VP III assay, remain useful for diagnosing symptomatic BV because of their lower cost and ability to provide a rapid diagnosis. Culture of *G. vaginalis* is not recommended as a diagnostic tool because it is not specific. Cervical Pap tests have no clinical utility for diagnosing BV because of their low sensitivity and specificity.

#### Treatment

Treatment for BV is recommended for women with symptoms. Established benefits of therapy among nonpregnant women are to relieve vaginal symptoms and signs of infection. Other potential benefits of treatment include reduction in the risk for acquiring *C. trachomatis, N. gonorrhoeae*, *T. vaginalis*, *M. genitalium*, HIV, HPV, and HSV-2 (*971*,*986*–*988*,*990*,*1010*). No data are available that directly compare the efficacy of oral and topical medications for treating BV.

**Recommended Regimens for Bacterial Vaginosis**
**Metronidazole **500 mg orally 2 times/day for 7 days
*or*
**Metronidazole gel 0.75%** one full applicator (5 g) intravaginally, once daily for 5 days
*or*
**Clindamycin cream** **2%** one full applicator (5 g) intravaginally at bedtime for 7 days

A review regarding alcohol consumption during metronidazole treatment reported no in vitro studies, animal models, reports of adverse effects, or clinical studies providing convincing evidence of a disulfiram-like interaction between alcohol and metronidazole (*1011*). The previous warning against simultaneous use of alcohol and metronidazole was based on laboratory experiments and individual case histories in which the reported reactions were equally likely to have been caused by alcohol alone or by adverse effects of metronidazole.

Metronidazole does not inhibit acetaldehyde dehydrogenase, as occurs with disulfiram. Ethanol alone or ethanol-independent side effects of metronidazole might explain the suspicion of disulfiram-like effects. Thus, refraining from alcohol use while taking metronidazole (or tinidazole) is unnecessary. Clindamycin cream is oil based and might weaken latex condoms and diaphragms for 5 days after use (refer to clindamycin product labeling for additional information).

Women should be advised to refrain from sexual activity or to use condoms consistently and correctly during the BV treatment regimen. Douching might increase the risk for relapse, and no data support use of douching for treatment or symptom relief.

**Alternative Regimens**
**Clindamycin** 300 mg orally 2 times/day for 7 days
*or*
**Clindamycin ovules** 100 mg intravaginally once at bedtime for 3 days*
*or*
**Secnidazole** 2 g oral granules in a single dose^†^
*or*
**Tinidazole** 2 g orally once daily for 2 days
*or*
**Tinidazole **1 g orally once daily for 5 days
* Clindamycin ovules use an oleaginous base that might weaken latex or rubber products (e.g., condoms and diaphragms). Use of such products within 72 hours after treatment with clindamycin ovules is not recommended.
^† ^Oral granules should be sprinkled onto unsweetened applesauce, yogurt, or pudding before ingestion. A glass of water can be taken after administration to aid in swallowing.

Alternative regimens include secnidazole oral granules (*1012*–*1014*), multiple oral tinidazole regimens (*1015*), or clindamycin (oral or intravaginal) (*1016*). In a phase 3 clinical trial of secnidazole 2 g oral granules versus placebo, BV clinical cure rates at days 21–30 were 53% in the secnidazole arm compared with 19% in the placebo arm (p<0.001) (*1013*). Secnidazole is listed as an alternative regimen, due to its higher cost and lack of long-term outcomes compared with recommended BV treatments. A patient savings card for secnidazole is available at https://www.solosec.com/savings-card.

Additional BV treatment regimens include metronidazole 1.3% vaginal gel in a single dose (*1017*,*1018*) and clindamycin phosphate (Clindesse) 2% vaginal cream in a single dose (*1019*). In a phase 3 clinical trial of metronidazole 1.3% vaginal gel versus placebo, BV clinical cure rates at day 21 were 37.2% in the metronidazole 1.3% vaginal gel arm, compared with 26.6% in the placebo arm (p = 0.01) (*1018*). A patient savings card for metronidazole 1.3% vaginal gel is available at https://nuvessa.com/nuvessa_files/19_Nuvessa_WEB_Card_032819.pdf. In a multicenter, randomized, single-blind, parallel-group study of Clindesse 2% vaginal cream single dose versus clindamycin 2% vaginal cream at bedtime for 7 days among 540 women with BV, no statistically significant difference existed between groups in clinical cure at days 21–30 (64.3% versus 63.2%; p = 0.95) (*1019*); however, this study had methodologic problems. A patient savings card for Clindesse 2% vaginal cream is available at https://www.clindesse.com/pdf/CLINDESSE_SavingsCard.pdf.

BV biofilm disrupting agents (i.e., TOL-463) (*1020*) are being investigated to determine their role in enhancing the likelihood of BV cure relative to approved therapies. Studies have evaluated the clinical and microbiologic efficacy of intravaginal *Lactobacillus* and other probiotic formulations to treat BV and restore normal vaginal microbiota (*1021*–*1025*); overall, no studies support these products as an adjunctive or replacement therapy for women with BV.

#### Other Management Considerations

All women with BV should be tested for HIV and other STIs.

#### Follow-Up

Follow-up visits are unnecessary if symptoms resolve. Because persistent or recurrent BV is common, women should be advised to return for evaluation if symptoms recur. Limited data are available regarding optimal management strategies for women with persistent or recurrent BV. Using a different recommended treatment regimen can be considered for women who have a recurrence; however, retreatment with the same recommended regimen is an acceptable approach for treating persistent or recurrent BV after the first occurrence (*1026*). For women with multiple recurrences after completion of a recommended regimen, either 0.75% metronidazole gel or 750 mg metronidazole vaginal suppository twice weekly for >3 months has been reported to reduce recurrences, although this benefit does not persist when suppressive therapy is discontinued (*1027*,*1028*). Limited data indicate that for women with multiple recurrences, an oral nitroimidazole (metronidazole or tinidazole 500 mg 2 times/day for 7 days), followed by intravaginal boric acid 600 mg daily for 21 days and suppressive 0.75% metronidazole gel twice weekly for 4–6 months, might be an option for women with recurrent BV (*1029*). Monthly oral metronidazole 2 g administered with fluconazole 150 mg has also been evaluated as suppressive therapy; this regimen reduced the BV incidence and promoted colonization with normal vaginal microbiota (*1030*). A randomized controlled trial of a dendrimer-based microbicide 1% vaginal gel (Astodrimer) also reported favorable results in prolonging the time to BV recurrence, compared with placebo (*1031*). In addition, a clinical trial of *L. crispatus* CTV-05 (Lactin-V), administered vaginally in 4 consecutive daily doses for 4 days in week 1 followed by twice weekly doses for 10 weeks (after initial treatment with 5 days of 0.75% vaginal metronidazole gel), reported a substantially lower incidence of BV recurrence at 12 weeks in the Lactin-V arm, compared with placebo (*1032*); however this medication is not yet FDA cleared or commercially available. High-dose Vitamin D supplementation has not been determined to decrease BV recurrence in randomized controlled trials (*1033*) and is not recommended.

#### Management of Sex Partners

Data from earlier clinical trials indicate that a woman’s response to therapy and the likelihood of relapse or recurrence are not affected by treatment of her sex partner (*998*). Therefore, routine treatment of sex partners is not recommended. However, a pilot study reported that male partner treatment (i.e., metronidazole 400 mg orally 2 times/day in conjunction with 2% clindamycin cream applied topically to the penile skin 2 times/day for 7 days) of women with recurrent BV had an immediate and sustained effect on the composition of the vaginal microbiota, with an overall decrease in bacterial diversity at day 28 (*1034*). Male partner treatment also had an immediate effect on the composition of the penile microbiota; however, this was not as pronounced at day 28, compared with that among women. A phase 3 multicenter randomized double-blinded trial evaluating the efficacy of a 7-day oral metronidazole regimen versus placebo for treatment of male sex partners of women with recurrent BV did not find that male partner treatment reduced BV recurrence in female partners, although women whose male partners adhered to multidose metronidazole were less likely to experience treatment failure (*1035*).

#### Special Considerations

##### Drug Allergy, Intolerance, or Adverse Reactions

Intravaginal clindamycin cream is preferred in case of allergy or intolerance to metronidazole or tinidazole. Intravaginal metronidazole gel can be considered for women who are not allergic to metronidazole but do not tolerate oral metronidazole.

##### Pregnancy

BV treatment is recommended for all symptomatic pregnant women because symptomatic BV has been associated with adverse pregnancy outcomes, including premature rupture of membranes, preterm birth, intra-amniotic infection, and postpartum endometritis (*989*,*991*,*1036*). Studies have been undertaken to determine the efficacy of BV treatment among this population, including two trials demonstrating that oral metronidazole was efficacious during pregnancy by using the 250 mg 3 times/day regimen (*1037*,*1038*); however, oral metronidazole administered as a 500 mg 2 times/day regimen can also be used. One trial involving a limited number of participants revealed treatment with oral metronidazole 500 mg 2 times/day for 7 days to be equally effective as metronidazole gel 0.75% for 5 days, with cure rates of 70% by using Amsel criteria to define cure (*1039*). Another trial demonstrated a cure rate of 85% by using Gram-stain criteria after treatment with oral clindamycin 300 mg 2 times/day for 7 days (*1040*–*1043*).

Although older studies indicated a possible link between using vaginal clindamycin during pregnancy and adverse outcomes for the newborn, newer data demonstrate that this treatment approach is safe for pregnant women (*1044*). Although metronidazole crosses the placenta, no evidence of teratogenicity or mutagenic effects among infants has been reported in multiple cross-sectional, case-control, and cohort studies of pregnant women (*1041*–*1043*). These data indicate that metronidazole therapy poses low risk during pregnancy. Data from human studies are limited regarding the use of tinidazole in pregnancy; however, animal data demonstrate that such therapy poses moderate risk. Thus, tinidazole should be avoided during pregnancy (*431*). Data are insufficient regarding efficacy and adverse effects of secnidazole, Clindesse 2% vaginal cream, metronidazole 1.3% vaginal gel, and 750-mg vaginal metronidazole tablets during pregnancy; thus, their use should be avoided.

Oral therapy has not been reported to be superior to topical therapy for treating symptomatic BV in effecting cure or preventing adverse outcomes of pregnancy. Pregnant women can be treated with any of the recommended regimens for nonpregnant women, in addition to the alternative regimens of oral clindamycin and clindamycin ovules.

Treatment of asymptomatic BV among pregnant women at high risk for preterm delivery (i.e., those with a previous preterm birth or late miscarriage) has been evaluated by multiple studies, which have yielded mixed results. Seven trials have evaluated treatment of pregnant women with asymptomatic BV at high risk for preterm delivery: one revealed harm (*1045*), two reported no benefit (*1046*,*1047*), and four demonstrated benefit (*1037*,*1038*,*1048*,*1049*).

Treatment of asymptomatic BV among pregnant women at low risk for preterm delivery has not been reported to reduce adverse outcomes of pregnancy in a large multicenter randomized controlled trial (*1050*). Therefore, routine screening for BV among asymptomatic pregnant women at high or low risk for preterm delivery for preventing preterm birth is not recommended.

Metronidazole is secreted in breast milk. With maternal oral therapy, breastfed infants receive metronidazole in doses that are less than those used to treat infections among infants, although the active metabolite adds to the total infant exposure. Plasma levels of the drug and metabolite are measurable but remain less than maternal plasma levels (https://www.ncbi.nlm.nih.gov/books/NBK501922/?report=classic). Although multiple reported case series identified no evidence of metronidazole-associated adverse effects for breastfed infants, certain clinicians recommend deferring breastfeeding for 12–24 hours after maternal treatment with a single 2-g dose of metronidazole (*1051*). Lower doses produce a lower concentration in breast milk and are considered compatible with breastfeeding (*1052*,*1053*).

##### HIV Infection

BV appears to recur with higher frequency among women who have HIV infection (*1054*). Women with HIV infection and BV should receive the same treatment regimen as those who do not have HIV.

### Trichomoniasis

Trichomoniasis is estimated to be the most prevalent nonviral STI worldwide, affecting approximately 3.7 million persons in the United States (*838**,**1055*). Because trichomoniasis is not a reportable disease (*1056*), and no recommendations are available for general screening for *T. vaginalis*, the epidemiology of trichomoniasis has largely come from population-based and clinic-based surveillance studies. The U.S. population-based *T. vaginalis* prevalence is 2.1% among females and 0.5% among males, with the highest rates among Black females (9.6%) and Black males (3.6%), compared with non-Hispanic White women (0.8%) and Hispanic women (1.4%) (*1057*,*1058*). Unlike chlamydia and gonorrhea, *T. vaginalis* prevalence rates are as high among women aged >24 years as they are for women aged <24 years (*1057*). Among persons attending nine geographically diverse STD clinics, the trichomonas prevalence was 14.6% among women (*1059*), and a study of STD clinic attendees in Birmingham, Alabama, identified a prevalence of 27% among women and 9.8% among men (*1060*). Symptomatic women have a four times higher rate of infection than asymptomatic women (26% versus 6.5%) (*1061*). Rates are also high among incarcerated persons of both sexes at 9%–32% of incarcerated women (*386*,*387*,*391*,*392*,*1062*) and 3.2%–8% of incarcerated men (*388*). Women with a history of incarceration are two to five times more likely to have *T. vaginalis* (*387*,*388*,*1063*,*1064*). Other risk factors for *T. vaginalis* include having two or more sex partners during the previous year, having less than a high school education, and living below the national poverty level (*1065*). Women with BV are at higher risk for *T. vaginalis* (*1066*). Male partners of women with trichomoniasis are likely to have infection (*1067*), although the prevalence of trichomoniasis among MSM is low (*179*,*1068*).

The majority of persons who have trichomoniasis (70%–85%) either have minimal or no genital symptoms, and untreated infections might last from months to years (*137*,*1069*,*1070*). Men with trichomoniasis sometimes have symptoms of urethritis, epididymitis, or prostatitis, and women with trichomoniasis sometimes have vaginal discharge, which can be diffuse, malodorous, or yellow-green with or without vulvar irritation, and might have a strawberry-appearing cervix, which is observed more often on colposcopy than on physical examination (*1071*). Although many persons might be unaware of their infection, it is readily passed between sex partners during penile-vaginal sex (*1072*) or through transmission of infected vaginal fluids or fomites among women who have sex with women (*275*,*294*).

Among persons who are sexually active, the best way to prevent genital trichomoniasis is through consistent and correct use of condoms (external or internal) (*18*). Partners of men who have been circumcised might have a somewhat reduced risk for *T. vaginalis* infection (*1072*,*1073*). Douching is not recommended because it might increase the risk for vaginal infections, including trichomoniasis (*1074*).

*T. vaginalis* causes reproductive morbidity and has been reported to be associated with a 1.4-times greater likelihood of preterm birth, premature rupture of membranes, and infants who are small for gestational age (*1075*). *T. vaginalis* was also determined to be associated with a 2.1-fold increased risk for cervical cancer in a meta-analysis (*1076*). Another meta-analysis of six studies reported a slightly elevated but not statistically significant association between *T. vaginalis* and prostate cancer (*1077*).

*T. vaginalis* infection is associated with a 1.5-fold increased risk for HIV acquisition and is associated with an increase in HIV vaginal shedding, which is reduced with *T. vaginalis* treatment among women without viral suppression (*1078*,*1079*). Among women with HIV infection, *T. vaginalis* infection is associated with increased risk for PID (*1080*–*1082*).

Diagnostic testing for *T. vaginalis* should be performed for women seeking care for vaginal discharge. Annual screening might be considered for persons receiving care in high-prevalence settings (e.g., STD clinics and correctional facilities) and for asymptomatic women at high risk for infection (e.g., multiple sex partners, transactional sex, drug misuse, or a history of STIs or incarceration). However, data are lacking regarding whether screening and treatment for asymptomatic trichomoniasis in high-prevalence settings for women at high risk can reduce any adverse health events and health disparities or reduce community infection burden. Decisions about screening can be guided by local epidemiology of *T. vaginalis* infection. Routine annual screening for *T. vaginalis* among asymptomatic women with HIV infection is recommended because of these adverse events associated with trichomoniasis and HIV infection.

Extragenital *T. vaginalis* is possible but highly uncommon compared with genital infections. A study of 500 men in San Francisco, California, reported a 0.6% rate of rectal *T. vaginalis* (*1083*); however, this might reflect deposition of *T. vaginalis* DNA and not necessarily active infection. Few studies of extragenital *T. vaginalis* among women have been published. The efficacy, benefit, and cost-effectiveness of extragenital screening are unknown, and no tests are FDA cleared for extragenital testing; therefore, rectal and oral testing for *T. vaginalis* is not recommended.

#### Diagnostic Considerations

Wet-mount microscopy traditionally has been used as the preferred diagnostic test for *T. vaginalis* among women because it is inexpensive and can be performed at the POC; however, it has low sensitivity (44%–68%) compared with culture (*1084*–*1086*). To improve detection, clinicians using wet mounts should attempt to evaluate slides immediately after specimen collection because sensitivity decreases quickly to 20% within 1 hour after collection (*1087*). More highly sensitive and specific molecular diagnostic options are available, which should be used in conjunction with a negative wet mount when possible.

NAATs are highly sensitive, detecting more *T. vaginalis* infections than wet-mount microscopy among women (*1060*). The Aptima *T. vaginalis* assay (Beckton Dickinson) is FDA cleared for detection of *T. vaginalis* from symptomatic or asymptomatic women. Reliable samples include clinician-collected endocervical swabs, clinician-collected vaginal swabs, female urine specimens, and liquid Pap smear specimens collected in PreservCyt Solution (Hologic) (*698*,*1088*). This assay detects RNA by transcription-mediated amplification with a sensitivity of 95.3%–100% and specificity of 95.2%–100%, compared with wet mount and culture (*1088*,*1089*). Among women, vaginal swabs and urine specimens have <100% concordance (*1084*). This assay has not been FDA cleared for use among men and should be internally validated in accordance with CLIA regulations before use with urine or urethral swabs from men. The Probe Tec TV Q^x^ Amplified DNA Assay (Becton Dickinson) is FDA cleared for detection of *T. vaginalis* from vaginal (patient-collected or clinician-collected) swabs, endocervical swabs, or urine specimens from women and has sensitivity of 98.3% and specificity of 99.6%, compared with wet mount and culture (*1090*). Similar to the Aptima *T. vaginalis* assay, this test is only FDA cleared for use among women and should be internally validated for use with men. The Max CTGCTV2 assay (Becton Dickinson) is also FDA cleared for detection of *T. vaginalis* in patient-collected or clinician-collected vaginal swab specimens and male and female urine specimens, with sensitivity and specificity of 96.2%–100% and 99.1%–100%, respectively, depending on the specimen type, compared with wet mount and culture (*1091*). GeneXpert TV (Cepheid) is a moderately complex rapid test that can be performed in ≤1 hour and can be used at the POC (*1092*). It has been FDA cleared for use with female urine specimens, endocervical swabs, patient-collected or clinician-collected vaginal specimens, and male urine specimens, with sensitivity and specificity of 99.5%–100% and 99.4%–99.9% (*1007*), respectively, compared with wet mount and culture.

Multiple FDA-cleared rapid tests are available for detecting *T. vaginalis* with improved sensitivities and specificities, compared with wet mount. The Osom trichomonas rapid test (Sekisui Diagnostics) is an antigen-detection test that uses immunochromatographic capillary flow dipstick technology that can be performed at the POC by using clinician-obtained vaginal specimens. Results are available in approximately 10–15 minutes, with sensitivities of 82%–95% and specificity of 97%–100%, compared with wet mount, culture, and transcription-mediated amplification (*1089*,*1093*,*1094*). A study of 209 women aged 14–22 years reported that >99% could correctly perform and interpret a vaginal self-test by using the Osom assay, with a high correlation with clinician interpretation (96% agreement; κ = 0.87) (*1094*). The Osom test should not be used with men because of low sensitivity (38% compared with Aptima) (*1095*). The Solana trichomonas assay (Quidel) is another rapid test for the qualitative detection of *T. vaginalis* DNA and can yield results <40 minutes after specimen collection. This assay is FDA cleared for diagnosing *T. vaginalis* from female vaginal and urine specimens from asymptomatic and symptomatic women with sensitivity >98%, compared with NAAT for vaginal specimens, and >92% for urine specimens (*1096*). The Amplivue trichomonas assay (Quidel) is another rapid test providing qualitative detection of *T.* *vaginalis* that has been FDA cleared for vaginal specimens from symptomatic and asymptomatic women, with sensitivity of 90.7% and specificity of 98.9%, compared with NAAT (*1097*). Neither the Osom assay nor the Affirm VP III test is FDA cleared for use with specimens from men.

Culture, such as the InPouch system (BioMed Diagnostics), was considered the most sensitive method for diagnosing *T. vaginalis* infection before molecular detection methods became available. Culture has sensitivity of 44%–75% and specificity of <100% (*698*,*1086*,*1098*). For women, vaginal secretions are the preferred specimen type for culture because urine culture is less sensitive (*698*,*1099*,*1100*). For men, culture specimens require a urethral swab, urine sediment, or semen. To improve diagnostic yield, multiple specimens from men can be used to inoculate a single culture. Cultures require an incubator and are necessary for *T. vaginalis* drug susceptibility testing. The InPouch specimen should be examined daily for 5 days over a 7-day period to reduce the possibility of false negatives (*1101*).

Although *T. vaginalis* might be an incidental finding on a Pap test, neither conventional nor liquid-based Pap smears are considered diagnostic tests for trichomoniasis; however, women with *T. vaginalis* identified on a Pap smear should be retested with sensitive diagnostic tests and treated if infection is confirmed (*1102*,*1103*).

#### Treatment

Treatment reduces symptoms and signs of *T. vaginalis* infection and might reduce transmission. Treatment recommendations for women are based on a meta-analysis (*1104*) and a multicenter, randomized trial of mostly symptomatic women without HIV infection (*1105*). The study demonstrated that multidose metronidazole (500 mg orally 2 times/day for 7 days) reduced the proportion of women retesting positive at a 1-month test of cure visit by half, compared with women who received the 2-g single dose. No published randomized trials are available that compare these doses among men.

**Recommended Regimen for Trichomoniasis Among Women **
**Metronidazole** 500 mg orally 2 times/day for 7 days
**Recommended Regimen for Trichomoniasis Among Men **
**Metronidazole** 2 g orally in a single dose
**Alternative Regimen for Women and Men**
**Tinidazole** 2 g orally in a single dose

The nitroimidazoles are the only class of medications with clinically demonstrated efficacy against *T. vaginalis* infections. Tinidazole is usually more expensive, reaches higher levels in serum and the genitourinary tract, has a longer half-life than metronidazole (12.5 hours versus 7.3 hours), and has fewer gastrointestinal side effects (*1106*,*1107*). In randomized clinical trials, recommended metronidazole regimens have resulted in cure rates of approximately 84%–98% (*1108*), and the recommended tinidazole regimen has resulted in cure rates of approximately 92%–100% (*1108*–*1112*). Randomized controlled trials comparing single 2-g doses of metronidazole and tinidazole indicated that tinidazole is equivalent or superior to metronidazole in achieving parasitologic cure and symptom resolution (*1110*,*1113*,*1114*).

Metronidazole gel does not reach therapeutic levels in the urethra and perivaginal glands. Because it is less efficacious than oral metronidazole, it is not recommended.

#### Other Management Considerations

Providers should advise persons with *T. vaginalis* infections to abstain from sex until they and their sex partners are treated (i.e., when therapy has been completed and any symptoms have resolved). Testing for other STIs, including HIV, syphilis, gonorrhea, and chlamydia, should be performed for persons with *T. vaginalis*.

#### Follow-Up

Because of the high rate of reinfection among women treated for trichomoniasis, retesting for *T. vaginalis* is recommended for all sexually active women <3 months after initial treatment regardless of whether they believe their sex partners were treated (*137*,*1115*). If retesting at 3 months is not possible, clinicians should retest whenever persons next seek medical care <12 months after initial treatment. Data are insufficient to support retesting men after treatment.

#### Management of Sex Partners

Concurrent treatment of all sex partners is vital for preventing reinfections. Current partners should be referred for presumptive therapy. Partners also should be advised to abstain from intercourse until they and their sex partners have been treated and any symptoms have resolved. EPT might have a role in partner management for trichomoniasis (*129*,*1116*) and can be used in states where permissible by law (https://www.cdc.gov/std/ept/legal/default.htm); however, no partner management intervention has been demonstrated to be superior in reducing reinfection rates (*129*,*130*). Although no definitive data exist to guide treatment for partners of persons with persistent or recurrent trichomoniasis among whom nonadherence and reinfection are unlikely, partners might benefit from being evaluated and receiving treatment (see Recurrent Trichomoniasis).

#### Recurrent Trichomoniasis

A recurrent infection can result from treatment failure (antimicrobial-resistant *T. vaginalis* or host-related problems), lack of adherence, or reinfection from an untreated sex partner. In the case of a recurrent infection, the origin of the repeat infection should be assessed because most recurrent infections likely result from reinfection. Retesting can be considered in cases of persistent or recurrent trichomoniasis with culture, the preferred test. If NAAT is used, it should not be conducted before 3 weeks after treatment completion because of possible detection of residual nucleic acid that is not clinically relevant (*1117*).

The nitroimidazoles are the only class of antimicrobials known to be effective against trichomonas infection. Metronidazole resistance occurs in 4%–10% of cases of vaginal trichomoniasis (*1116*,*1118*). Tinidazole resistance is less well studied but was present in 1% of infections in one study (*1116*). Overall, more *T. vaginalis* isolates have reported susceptibility to tinidazole than metronidazole (*1119*). Multidose oral metronidazole is more effective than single-dose treatment, particularly for women who are symptomatic or have a history of *T. vaginalis* (*1120*)*.*

Nitroimidazole-resistant trichomoniasis is concerning because few alternatives to standard therapy exist. If treatment failure occurs in a woman after completing a regimen of metronidazole 500 mg 2 times/day for 7 days and she has been reexposed to an untreated partner, a repeat course of the same regimen is recommended. If no reexposure has occurred, she should be treated with metronidazole or tinidazole 2 g once daily for 7 days. If a man has persistent *T. vaginalis* after a single 2-g dose of metronidazole and has been reexposed to an untreated partner, he should be retreated with a single 2-g dose of metronidazole. If he has not been reexposed, he should be administered a course of metronidazole 500 mg 2 times/day for 7 days.

For persons who are experiencing persistent infection not attributable to reexposure, clinicians should request a kit from CDC to perform drug-resistance testing (https://www.cdc.gov/laboratory/specimen-submission/detail.html?CDCTestCode=CDC-10239). CDC is experienced with susceptibility testing for nitroimidazole-resistant *T. vaginalis* and can provide guidance regarding treatment in cases of drug resistance. On the basis of drug resistance testing, an alternative treatment regimen might be recommended. Treatments for infections demonstrating in vitro resistance can include metronidazole or tinidazole 2 g daily for 7 days. If a patient has treatment failure after the 7-day regimen of high-dose oral metronidazole or tinidazole, two additional treatment options have been determined to have successful results for women. The first is high-dose oral tinidazole 2 g daily plus intravaginal tinidazole 500 mg 2 times/day for 14 days (*1121*). If this regimen fails, high-dose oral tinidazole (1 g 3 times/day) plus intravaginal paromomycin (4 g of 6.25% intravaginal paromomycin cream nightly) for 14 days should be considered (*1122*).

Alternative regimens might be effective but have not been systemically evaluated; therefore, consultation with an infectious disease specialist is recommended. Clinical improvement has been reported with intravaginal boric acid (*1123**,**1124*) but not with nitazoxanide (*1123*–*1125*). The following topically applied agents have minimal success (<50%) and are not recommended: intravaginal betadine (povidone-iodine), clotrimazole, acetic acid, furazolidone, GV, nonoxynol-9, and potassium permanganate (*1126*). No other topical microbicide has been reported to be effective against trichomoniasis.

#### Special Considerations

##### Drug Allergy, Intolerance, and Adverse Reactions

Metronidazole and tinidazole are both nitroimidazoles. Patients with an IgE-mediated-type hypersensitivity reaction to 5-nitroimidazole antimicrobials should be managed by metronidazole desensitization according to published regimens (*1127*,*1128*) and in consultation with an allergy specialist. The optimal treatment for patients with *T. vaginalis* who are unable to be desensitized has not been systematically investigated and is based on case reports, some of which report using paromomycin or boric acid for treating *T. vaginalis* (*1123*,*1129*).

##### Pregnancy

*T. vaginalis* infection among pregnant women is associated with adverse pregnancy outcomes, particularly premature rupture of membranes, preterm delivery, and delivery of infants who are small for gestational age (*1075*). One randomized trial of pregnant women with asymptomatic trichomoniasis reported no substantial difference in preterm birth after treatment with 2 g of metronidazole 48 hours apart during 16–23 and 24–29 weeks’ gestation, compared with placebo (*1130*). However, that trial had multiple limitations, including use of an atypical metronidazole regimen. Another multicenter observational study of asymptomatic pregnant women in sub-Sahara African, the majority with HIV infection, reported neither trichomoniasis nor its treatment appeared to influence the risk for preterm birth or a low-birthweight infant (*1131*).

Although metronidazole crosses the placenta, data indicate that it poses a low risk to the developing fetus (*1040*,*1042*,*1132*). No evidence of teratogenicity or mutagenic effects among infants has been found in multiple cross-sectional and cohort studies among pregnant women examining single-dose (2 g) and multidose metronidazole regimens (*1040*,*1131*–*1135*).

Symptomatic pregnant women, regardless of pregnancy stage, should be tested and treated. Treatment of *T. vaginalis* infection can relieve symptoms of vaginal discharge for pregnant women and reduce sexual transmission to partners. Although perinatal transmission of trichomoniasis is uncommon, treatment might also prevent respiratory or genital infection in the newborn (*1136*,*1137*). Clinicians should counsel symptomatic pregnant women with trichomoniasis about the potential risks and benefits of treatment and about the importance of partner treatment and condom use in the prevention of sexual transmission. The benefit of routine screening for *T. vaginalis* in asymptomatic pregnant women has not been established.

Metronidazole is secreted in breast milk. With maternal oral therapy, breastfed infants receive metronidazole in doses that are lower than those used to treat infections among infants, although the active metabolite adds to the total infant exposure. Plasma levels of the drug and metabolite are measurable but remain less than maternal plasma levels (https://www.ncbi.nlm.nih.gov/books/NBK501922). Although multiple reported case series studies demonstrated no evidence of adverse effects among infants exposed to metronidazole in breast milk, clinicians sometimes advise deferring breastfeeding for 12–24 hours after maternal treatment with metronidazole (*1051*). In one study, maternal treatment with metronidazole (400 mg 3 times/day for 7 days) produced a lower concentration in breast milk and was considered compatible with breastfeeding over longer periods (*1052*).

Data from studies involving human subjects are limited regarding tinidazole use during pregnancy; however, animal data indicate this drug poses moderate risk. Thus, tinidazole should be avoided for pregnant women, and breastfeeding should be deferred for 72 hours after a single 2-g oral dose of tinidazole (https://www.ncbi.nlm.nih.gov/books/NBK501922).

##### HIV Infection

Up to 53% of women with HIV have *T. vaginalis* infection (*1115*,*1138*). *T. vaginalis* infection among these women is substantially associated with pelvic inflammatory disease (*1082*). Among women who are not virally suppressed, treatment of trichomoniasis is associated with decreases in genital tract HIV viral load and viral shedding (*1079*,*1139*); however, no difference might occur among women who are virally suppressed (*1140*). Because of the high prevalence of *T. vaginalis* among women with HIV and the potential for adverse reproductive health, poor birth outcomes, and possibly amplified HIV transmission, routine screening and prompt treatment are recommended for all women with HIV infection; screening should occur at entry to care and then at least annually thereafter.

A randomized clinical trial involving women with HIV and *T. vaginalis* infection demonstrated that a single dose of metronidazole 2 g orally was less effective than 500 mg 2 times/day for 7 days (*1105*). Factors that might interfere with standard single-dose treatment for trichomoniasis among women with HIV include high rates of asymptomatic BV infection, ART use, changes in vaginal ecology, and impaired immunity (*1141*). Thus, to improve cure rates, women with HIV who receive a diagnosis of *T. vaginalis* infection should be treated with metronidazole 500 mg orally 2 times/day for 7 days. For pregnant women with HIV, screening at the first prenatal visit and prompt treatment, as needed, are recommended because *T. vaginalis* infection is a risk factor for vertical transmission of HIV (*1142*).

#### Treatment

Treatment reduces symptoms and signs of *T. vaginalis* infection, cures infection, and might reduce transmission. Likelihood of adverse outcomes among women with HIV infection is also reduced with *T. vaginalis* therapy.

**Recommended Regimen for Trichomonas and HIV Infection Among Women **
**Metronidazole** 500 mg orally 2 times/day for 7 days

If a woman with HIV infection experiences treatment failure, the protocol outlined is recommended (see Recurrent Trichomonas). Other management considerations, follow-up, and management of sex partners should be performed as for women without HIV infection. Treatment of men with HIV infection should follow the same guidelines as for men without HIV.

For women with HIV who receive a diagnosis of *T. vaginalis* infection, retesting is recommended 3 months after treatment; NAAT is encouraged because of higher sensitivity of these tests. Data are insufficient to support retesting of men with trichomonas and HIV infection.

### Vulvovaginal Candidiasis

VVC usually is caused by *Candida albicans* but can occasionally be caused by other *Candida* species or yeasts. Typical symptoms of VVC include pruritus, vaginal soreness, dyspareunia, external dysuria, and abnormal vaginal discharge. None of these symptoms is specific for VVC. An estimated 75% of women will have at least one episode of VVC, and 40%–45% will have two or more episodes. On the basis of clinical presentation, microbiology, host factors, and response to therapy, VVC can be classified as either uncomplicated or complicated (Box 4). Approximately 10%–20% of women will have complicated VVC, requiring special diagnostic and therapeutic considerations.

BOX 4 **Classification of vulvovaginal candidiasis**

**Uncomplicated vulvovaginal candidiasis (VVC)**

- Sporadic or infrequent VVC ***and***
- Mild-to-moderate VVC ***and***
- Likely to be *Candida albicans ****and***
- Nonimmunocompromised women

**Complicated VVC**

- Recurrent VVC (three or more episodes of symptomatic VVC in <1 year) ***or***
- Severe VVC ***or***
- Non–*albicans* candidiasis ***or***
- Women with diabetes, immunocompromising conditions (e.g., HIV infection), underlying immunodeficiency, or immunosuppressive therapy (e.g., corticosteroids)

**Source:** Sobel JD, Faro S, Force RW, et al. Vulvovaginal candidiasis: epidemiologic, diagnostic, and therapeutic considerations. Am J Obstet Gynecol 1998;178:203–11.

#### Uncomplicated Vulvovaginal Candidiasis

##### Diagnostic Considerations

A diagnosis of *Candida* vaginitis is clinically indicated by the presence of external dysuria and vulvar pruritus, pain, swelling, and redness. Signs include vulvar edema, fissures, excoriations, and thick curdy vaginal discharge. Most healthy women with uncomplicated VVC have no identifiable precipitating factors. The diagnosis can be made in a woman who has signs and symptoms of vaginitis when either a wet preparation (saline, 10% KOH) of vaginal discharge demonstrates budding yeasts, hyphae, or pseudohyphae, or a culture or other test yields a positive result for a yeast species. *Candida* vaginitis is associated with normal vaginal pH (<4.5). Use of 10% KOH in wet preparations improves the visualization of yeast and mycelia by disrupting cellular material that might obscure the yeast or pseudohyphae. Examination of a wet mount with KOH preparation should be performed for all women with symptoms or signs of VVC, and women with a positive result should be treated. For those with negative wet mounts but existing signs or symptoms, vaginal cultures for *Candida* should be considered. If *Candida* cultures cannot be performed for these women, empiric treatment can be considered. Identifying *Candida* by culture in the absence of symptoms or signs is not an indication for treatment because approximately 10%–20% of women harbor *Candida* species and other yeasts in the vagina. The majority of PCR tests for yeast are not FDA cleared, and providers who use these tests should be familiar with the performance characteristics of the specific test used. Yeast culture, which can identify a broad group of pathogenic yeasts, remains the reference standard for diagnosis.

##### Treatment

Short-course topical formulations (i.e., single dose and regimens of 1–3 days) effectively treat uncomplicated VVC. Treatment with azoles results in relief of symptoms and negative cultures in 80%–90% of patients who complete therapy.

**Recommended Regimens for Vulvovaginal Candidiasis**
**Over-the-Counter Intravaginal Agents**
**Clotrimazole** **1% cream **5 g intravaginally daily for 7–14 days
*or*
**Clotrimazole 2% cream** 5 g intravaginally daily for 3 days
*or*
**Miconazole** **2% cream** 5 g intravaginally daily for 7 days
*or*
**Miconazole 4% cream** 5 g intravaginally daily for 3 days
*or*
**Miconazole 100 mg vaginal suppository** one suppository daily for 7 days
*or*
**Miconazole 200 mg vaginal suppository** one suppository for 3 days
*or*
**Miconazole 1,200 mg vaginal suppository** one suppository for 1 day
*or*
**Tioconazole 6.5% ointment** 5 g intravaginally in a single application
**Prescription Intravaginal Agents**
**Butoconazole 2% cream** (single-dose bioadhesive product) 5 g intravaginally in a single application
*or*
**Terconazole 0.4% cream** 5 g intravaginally daily for 7 days
*or*
**Terconazole 0.8% cream** 5 g intravaginally daily for 3 days
*or*
**Terconazole 80 mg vaginal suppository** one suppository daily for 3 days
**Oral Agent**
**Fluconazole** 150 mg orally in a single dose

The creams and suppositories in these regimens are oil based and might weaken latex condoms and diaphragms. Patients should refer to condom product labeling for further information. Even women who have previously received a diagnosis of VVC by a clinician are not necessarily more likely to be able to diagnose themselves; therefore, any woman whose symptoms persist after using an over-the-counter preparation or who has a recurrence of symptoms <2 months after treatment for VVC should be evaluated clinically and tested. Unnecessary or unapproved use of over-the-counter preparations is common and can lead to a delay in treatment of other vulvovaginitis etiologies, which can result in adverse outcomes. No substantial evidence exists to support using probiotics or homeopathic medications for treating VVC.

##### Follow-Up

Follow-up typically is not required. However, women with persistent or recurrent symptoms after treatment should be instructed to return for follow-up visits.

##### Management of Sex Partners

Uncomplicated VVC is not usually acquired through sexual intercourse, and data do not support treatment of sex partners. A minority of male sex partners have balanitis, characterized by erythematous areas on the glans of the penis in conjunction with pruritus or irritation. These men benefit from treatment with topical antifungal agents to relieve symptoms.

##### Special Considerations

###### Drug Allergy, Intolerance, and Adverse Reactions

Topical agents usually cause no systemic side effects, although local burning or irritation might occur. Oral azoles occasionally cause nausea, abdominal pain, and headache. Therapy with the oral azoles has rarely been associated with abnormal elevations of liver enzymes. Clinically important interactions can occur when oral azoles are administered with other drugs (*1141*).

#### Complicated Vulvovaginal Candidiasis

##### Diagnostic Considerations

Vaginal culture or PCR should be obtained from women with complicated VVC to confirm clinical diagnosis and identify non–*albicans Candida*. *Candida glabrata* does not form pseudohyphae or hyphae and is not easily recognized on microscopy. *C. albicans* azole resistance is becoming more common in vaginal isolates (*1144*,*1145*), and non–*albicans Candida* is intrinsically resistant to azoles; therefore, culture and susceptibility testing should be considered for patients who remain symptomatic.

##### Recurrent Vulvovaginal Candidiasis

Recurrent VVC, usually defined as three or more episodes of symptomatic VVC in <1 year, affects <5% of women but carries a substantial economic burden (*1146*). Recurrent VVC can be either idiopathic or secondary (related to frequent antibiotic use, diabetes, or other underlying host factors). The pathogenesis of recurrent VVC is poorly understood, and the majority of women with recurrent VVC have no apparent predisposing or underlying conditions. *C. glabrata* and other non–*albicans Candida* species are observed in 10%–20% of women with recurrent VVC. Conventional antimycotic therapies are not as effective against these non–*albicans* yeasts as against *C. albicans*.

##### Treatment

Most episodes of recurrent VVC caused by *C. albicans* respond well to short-duration oral or topical azole therapy. However, to maintain clinical and mycologic control, a longer duration of initial therapy (e.g., 7–14 days of topical therapy or a 100-mg, 150-mg, or 200-mg oral dose of fluconazole every third day for a total of 3 doses [days 1, 4, and 7]) is recommended, to attempt mycologic remission, before initiating a maintenance antifungal regimen.

Oral fluconazole (i.e., a 100-mg, 150-mg, or 200-mg dose) weekly for 6 months is the indicated maintenance regimen. If this regimen is not feasible, topical treatments used intermittently can also be considered. Suppressive maintenance therapies are effective at controlling recurrent VVC but are rarely curative long-term (*1147*). Because *C. albicans* azole resistance is becoming more common, susceptibility tests, if available, should be obtained among symptomatic patients who remain culture positive despite maintenance therapy. These women should be managed in consultation with a specialist.

##### Severe Vulvovaginal Candidiasis

Severe VVC (i.e., extensive vulvar erythema, edema, excoriation, and fissure formation) is associated with lower clinical response rates among patients treated with short courses of topical or oral therapy. Either 7–14 days of topical azole or 150 mg of fluconazole in two sequential oral doses (second dose 72 hours after initial dose) is recommended.

##### Non–*albicans* Vulvovaginal Candidiasis

Because approximately 50% of women with a positive culture for non–*albicans Candida* might be minimally symptomatic or have no symptoms, and because successful treatment is often difficult, clinicians should make every effort to exclude other causes of vaginal symptoms for women with non–*albicans* yeast (*1148*). The optimal treatment of non–*albicans* VVC remains unknown; however, a longer duration of therapy (7–14 days) with a nonfluconazole azole regimen (oral or topical) is recommended. If recurrence occurs, 600 mg of boric acid in a gelatin capsule administered vaginally once daily for 3 weeks is indicated. This regimen has clinical and mycologic eradication rates of approximately 70% (*1149*). If symptoms recur, referral to a specialist is advised.

##### Management of Sex Partners

No data exist to support treating sex partners of patients with complicated VVC. Therefore, no recommendation can be made.

#### Special Considerations

##### Compromised Host

Women with underlying immunodeficiency, those with poorly controlled diabetes or other immunocompromising conditions (e.g., HIV), and those receiving immunosuppression therapy (e.g., corticosteroid treatment) might not respond as well to short-term therapies. Efforts to correct modifiable conditions should be made, and more prolonged (i.e., 7–14 days) conventional treatment is necessary.

##### Pregnancy

VVC occurs frequently during pregnancy. Only topical azole therapies, applied for 7 days, are recommended for use among pregnant women. Epidemiologic studies indicate a single 150-mg dose of fluconazole might be associated with spontaneous abortion (*1150*) and congenital anomalies; therefore, it should not be used (*1151*).

##### HIV Infection

Vaginal *Candida* colonization rates among women with HIV infection are higher than among women without HIV with similar demographic and risk behavior characteristics, and the colonization rates correlate with increasing severity of immunosuppression (*1152*). Symptomatic VVC is also more frequent among women with HIV infection and similarly correlates with severity of immunodeficiency (*1153*). In addition, among women with HIV, systemic azole exposure is associated with isolation of non–*albicans Candida* species from the vagina.

Treatment for uncomplicated and complicated VVC among women with HIV infection should not differ from that for women who do not have HIV. Although long-term prophylactic therapy with fluconazole 200 mg weekly has been effective in reducing *C. albicans* colonization and symptomatic VVC (*1154*), this regimen is not recommended for women with HIV infection in the absence of complicated VVC (*98*). Although VVC is associated with increased HIV seroconversion among HIV-negative women and increased HIV cervicovaginal levels among women with HIV infection, the effect of treatment for VVC on HIV acquisition and transmission remains unknown.

## Pelvic Inflammatory Disease

PID comprises a spectrum of inflammatory disorders of the upper female genital tract, including any combination of endometritis, salpingitis, tubo-ovarian abscess, and pelvic peritonitis (*1155*–*1157*). Sexually transmitted organisms, especially *N. gonorrhoeae* and *C. trachomatis*, often are implicated. Recent studies report that the proportion of PID cases attributable to *N. gonorrhoeae* or *C. trachomatis* is decreasing; of women who received a diagnosis of acute PID, approximately 50% have a positive test for either of those organisms (*1158*–*1160*). Micro-organisms that comprise the vaginal flora, such as strict and facultative anaerobes (*1160*) and *G. vaginalis*, *H. influenzae*, enteric gram-negative rods, and *Streptococcus agalactiae, *have been associated with PID (*1161*). In addition, cytomegalovirus (CMV), *T. vaginalis*, *M. hominis*, and *U. urealyticum* might be associated with certain PID cases (*1072*). Data also indicate that *M. genitalium* might have a role in PID pathogenesis (*765*,*928*) and might be associated with milder symptoms (*919*,*923*,*928*), although one study failed to demonstrate a substantial increase in PID after detection of *M. genitalium* in the lower genital tract (*925*).

Screening and treating sexually active women for chlamydia and gonorrhea reduces their risk for PID (*1162*,*1163*). Although BV is associated with PID, whether PID incidence can be reduced by identifying and treating women with BV is unclear (*1161*). Whether screening young women for *M. genitalium* is associated with a reduction in PID is unknown.

### Diagnostic Considerations

Acute PID is difficult to diagnose because of the considerable variation in symptoms and signs associated with this condition. Women with PID often have subtle or nonspecific symptoms or are asymptomatic. Delay in diagnosis and treatment probably contributes to inflammatory sequelae in the upper genital tract. Laparoscopy can be used to obtain a more accurate diagnosis of salpingitis and a more complete bacteriologic diagnosis. However, this diagnostic tool frequently is not readily available, and its use is not easily justifiable when symptoms are mild or vague. Moreover, laparoscopy will not detect endometritis and might not detect subtle inflammation of the fallopian tubes. Consequently, a PID diagnosis usually is based on imprecise clinical findings (*1164*–*1166*).

Data indicate that a clinical diagnosis of symptomatic PID has a positive predictive value for salpingitis of 65%–90%, compared with laparoscopy (*1167*–*1170*). The positive predictive value of a clinical diagnosis of acute PID depends on the epidemiologic characteristics of the population, with higher positive predictive values among sexually active young women (particularly adolescents), women attending STD clinics, and those who live in communities with high rates of gonorrhea or chlamydia. Regardless of positive predictive value, no single historical, physical, or laboratory finding is both sensitive and specific for the diagnosis of acute PID. Combinations of diagnostic findings that improve either sensitivity (i.e., detect more women who have PID) or specificity (i.e., exclude more women who do not have PID) do so only at the expense of the other. For example, requiring two or more findings excludes more women who do not have PID and reduces the number of women with PID who are identified.

Episodes of PID often go unrecognized. Although certain cases are asymptomatic, others are not diagnosed because the patient or the health care provider do not recognize the implications of mild or nonspecific symptoms or signs (e.g., abnormal bleeding, dyspareunia, and vaginal discharge). Even women with mild or asymptomatic PID might be at risk for infertility (*1157*). Because of the difficulty of diagnosis and the potential for damage to the reproductive health of women, health care providers should maintain a low threshold for the clinical diagnosis of PID (*1158*). The recommendations for diagnosing PID are intended to assist health care providers to recognize when PID should be suspected and when additional information should be obtained to increase diagnostic certainty. Diagnosis and management of other causes of lower abdominal pain (e.g., ectopic pregnancy, acute appendicitis, ovarian cyst, ovarian torsion, or functional pain) are unlikely to be impaired by initiating antimicrobial therapy for PID. Presumptive treatment for PID should be initiated for sexually active young women and other women at risk for STIs if they are experiencing pelvic or lower abdominal pain, if no cause for the illness other than PID can be identified, or if one or more of the following three minimum clinical criteria are present on pelvic examination: cervical motion tenderness, uterine tenderness, or adnexal tenderness.

More specific criteria for diagnosing PID include endometrial biopsy with histopathologic evidence of endometritis; transvaginal sonography or magnetic resonance imaging techniques demonstrating thickened, fluid-filled tubes with or without free pelvic fluid or tubo-ovarian complex, or Doppler studies indicating pelvic infection (e.g., tubal hyperemia); and laparoscopic findings consistent with PID. A diagnostic evaluation that includes some of these more extensive procedures might be warranted in certain cases. Endometrial biopsy is warranted for women undergoing laparoscopy who do not have visual evidence of salpingitis because endometritis is the only sign of PID for certain women.

Requiring that all three minimum criteria be present before the initiation of empiric treatment can result in insufficient sensitivity for a PID diagnosis. After deciding whether to initiate empiric treatment, clinicians should also consider the risk profile for STIs. More elaborate diagnostic evaluation frequently is needed because incorrect diagnosis and management of PID might cause unnecessary morbidity. For example, the presence of signs of lower genital tract inflammation (predominance of leukocytes in vaginal secretions, cervical discharge, or cervical friability), in addition to one of the three minimum criteria, increases the specificity of the diagnosis. One or more of the following additional criteria can be used to enhance the specificity of the minimum clinical criteria and support a PID diagnosis:

- Oral temperature >38.3°C (>101°F)
- Abnormal cervical mucopurulent discharge or cervical friability
- Presence of abundant numbers of WBCs on saline microscopy of vaginal fluid
- Elevated erythrocyte sedimentation rate
- Elevated C-reactive protein
- Laboratory documentation of cervical infection with *N. gonorrhoeae* or *C. trachomatis*

The majority of women with PID have either mucopurulent cervical discharge or evidence of WBCs on a microscopic evaluation of a saline preparation of vaginal fluid (i.e., wet prep). If the cervical discharge appears normal and no WBCs are observed on the wet prep of vaginal fluid, a PID diagnosis is unlikely, and alternative causes of pain should be considered. A wet prep of vaginal fluid also can detect the presence of concomitant infections (e.g., BV or trichomoniasis).

### Treatment

PID treatment regimens should provide empiric, broad-spectrum coverage of likely pathogens. Multiple parenteral and oral antimicrobial regimens have been effective in achieving clinical and microbiologic cure in randomized clinical trials with short-term follow-up (*1171*–*1173*). However, only a limited number of studies have assessed and compared these regimens with regard to infection elimination in the endometrium and fallopian tubes or determined the incidence of long-term complications (e.g., tubal infertility and ectopic pregnancy) after antimicrobial regimens (*1159*,*1164*,*1174*). The optimal treatment regimen and long-term outcome of early treatment of women with subclinical PID are unknown. All regimens used to treat PID should also be effective against *N. gonorrhoeae* and *C. trachomatis* because negative endocervical screening for these organisms does not rule out upper genital tract infection. Anaerobic bacteria have been isolated from the upper genital tract of women who have PID, and data from in vitro studies have revealed that some anaerobes (e.g., *Bacteroides fragilis*) can cause tubal and epithelial destruction. BV is often present among women who have PID (*22*,*1160*,*1161*,*1175*). Addition of metronidazole to IM or oral PID regimens more effectively eradicates anaerobic organisms from the upper genital tract (*1160*). Until treatment regimens that do not cover anaerobic microbes have been demonstrated to prevent long-term sequelae (e.g., infertility and ectopic pregnancy) as successfully as the regimens that are effective against these microbes, using regimens with anaerobic activity should be considered. Treatment should be initiated as soon as the presumptive diagnosis has been made because prevention of long-term sequelae is dependent on early administration of recommended antimicrobials. For women with PID of mild or moderate clinical severity, parenteral and oral regimens appear to have similar efficacy. The decision of whether hospitalization is necessary should be based on provider judgment and whether the woman meets any of the following criteria:

- Surgical emergencies (e.g., appendicitis) cannot be excluded
- Tubo-ovarian abscess
- Pregnancy
- Severe illness, nausea and vomiting, or oral temperature >38.5°C (101°F)
- Unable to follow or tolerate an outpatient oral regimen
- No clinical response to oral antimicrobial therapy

No evidence is available to indicate that adolescents have improved outcomes from hospitalization for treatment of PID, and the clinical response to outpatient treatment is similar among younger and older women. The decision to hospitalize adolescents with acute PID should be based on the same criteria used for older women.

#### Parenteral Treatment

Randomized trials have demonstrated the efficacy of parenteral regimens (*1160*,*1171*,*1172*,*1176*). Clinical experience should guide decisions regarding transition to oral therapy, which usually can be initiated within 24–48 hours of clinical improvement. For women with tubo-ovarian abscesses, >24 hours of inpatient observation is recommended.

**Recommended Parenteral Regimens for Pelvic Inflammatory Disease**
**Ceftriaxone** 1 g IV every 24 hours
*plus*
**Doxycycline** 100 mg orally or IV every 12 hours
*plus*
**Metronidazole **500 mg orally or IV every 12 hours
*or*
**Cefotetan **2 g IV every 12 hours
*plus*
**Doxycycline** 100 mg orally or IV every 12 hours
*or*
**Cefoxitin** 2 g IV every 6 hours
*plus*
**Doxycycline** 100 mg orally or IV every 12 hours

Because of the pain associated with IV infusion, doxycycline should be administered orally when possible. Oral and IV administration of doxycycline and metronidazole provide similar bioavailability. Oral metronidazole is well absorbed and can be considered instead of IV for women without severe illness or tubo-ovarian abscess when possible. After clinical improvement with parenteral therapy, transition to oral therapy with doxycycline 100 mg 2 times/day and metronidazole 500 mg 2 times/day is recommended to complete 14 days of antimicrobial therapy.

#### Alternative Parenteral Regimens

Only limited data are available to support using other parenteral second- or third- generation cephalosporins (e.g., ceftizoxime or cefotaxime). Because these cephalosporins are less active than cefotetan or cefoxitin against anaerobic bacteria, the addition of metronidazole should be considered.

Ampicillin-sulbactam plus doxycycline has been investigated in at least one clinical trial and has broad-spectrum coverage (*1177*). Ampicillin-sulbactam plus doxycycline is effective against *C. trachomatis*, *N. gonorrhoeae*, and anaerobes for women with tubo-ovarian abscess. Another trial demonstrated short-term clinical cure rates with azithromycin monotherapy or combined with metronidazole (*1178*).

When using the clindamycin and gentamicin alternative parenteral regimen, women with clinical improvement after 24–28 hours can be transitioned to clindamycin (450 mg orally 4 times/day) or doxycycline (100 mg orally 2 times/day) to complete the 14-day therapy. However, when tubo-ovarian abscess is present, clindamycin (450 mg orally 4 times/day) or metronidazole (500 mg orally 2 times/day) should be used to complete 14 days of therapy with oral doxycycline to provide more effective anaerobic coverage.

**Alternative Parenteral Regimens**
**Ampicillin-sulbactam** 3 g IV every 6 hours
*plus*
**Doxycycline** 100 mg orally or IV every 12 hours
*or*
**Clindamycin **900 mg IV every 8 hours
*plus*
**Gentamicin** loading dose IV or IM (2 mg/kg body weight), followed by a maintenance dose (1.5 mg/kg body weight) every 8 hours; single daily dosing (3–5 mg/kg body weight) can be substituted

#### Intramuscular or Oral Treatment

IM or oral therapy can be considered for women with mild-to-moderate acute PID because the clinical outcomes among women treated with these regimens are similar to those treated with IV therapy (*1158*). Women who do not respond to IM or oral therapy within 72 hours should be reevaluated to confirm the diagnosis and be administered therapy IV.

**Recommended Intramuscular or Oral Regimens for Pelvic Inflammatory Disease**
**Ceftriaxone** 500 mg* IM in a single dose
*plus*
**Doxycycline **100 mg orally 2 times/day for 14 days with **metronidazole** 500 mg orally 2 times/day for 14 days
*or*
**Cefoxitin** 2 g IM in a single dose and **probenecid** 1 g orally administered concurrently in a single dose
*plus*
**Doxycycline** 100 mg orally 2 times/day for 14 days with **metronidazole** 500 mg orally 2 times/day for 14 days
*or*
**Other parenteral third-generation cephalosporin **(e.g., ceftizoxime or cefotaxime)
*plus*
**Doxycycline** 100 mg orally 2 times/day for 14 days with **metronidazole** 500 mg orally 2 times/day for 14 days
* For persons weighing ≥150 kg, 1 g of ceftriaxone should be administered.

These regimens provide coverage against frequent etiologic agents of PID; however, the optimal choice of a cephalosporin is unclear. Cefoxitin, a second-generation cephalosporin, has better anaerobic coverage than ceftriaxone, and, in combination with probenecid and doxycycline, has been effective in short-term clinical response among women with PID. Ceftriaxone has better coverage against *N. gonorrhoeae*. The addition of metronidazole to these regimens provides extended coverage against anaerobic organisms and will also effectively treat BV, which is frequently associated with PID.

#### Alternative Intramuscular or Oral Regimens

No data have been published regarding use of oral cephalosporins for treating PID. As a result of the emergence of quinolone-resistant *N. gonorrhoeae*, regimens that include a quinolone agent are not recommended for PID treatment. However, if the patient has cephalosporin allergy, the community prevalence and individual risk for gonorrhea are low, and follow-up is likely, alternative therapy can be considered. Use of either levofloxacin 500 mg orally once daily or moxifloxacin 400 mg orally once daily with metronidazole 500 mg orally 2 times/day for 14 days (*1179*–*1181*) or azithromycin 500 mg IV daily for 1–2 doses, followed by 250 mg orally daily in combination with metronidazole 500 mg 2 times/day for 12–14 days (*1178*), can be considered. Moxifloxacin is the preferred quinolone antimicrobial for *M. genitalium* infections; however, the importance of providing coverage for *M. genitalium* is unknown. Diagnostic tests for gonorrhea should be obtained before starting therapy, and persons should be managed as follows:

- If a culture for gonorrhea is positive, treatment should be based on results of antimicrobial susceptibility testing.
- If the isolate is determined to be quinolone-resistant *N. gonorrhoeae* or if antimicrobial susceptibility cannot be assessed (e.g., if only NAAT testing is available), consultation with an infectious disease specialist is recommended.

### Other Management Considerations

To minimize disease transmission, women should be instructed to abstain from sexual intercourse until therapy is complete, symptoms have resolved, and sex partners have been treated (see Chlamydial Infections; Gonococcal Infections). All women who receive a diagnosis of PID should be tested for gonorrhea, chlamydia, HIV, and syphilis. The value of testing women with PID for *M. genitalium* is unknown (see *Mycoplasma genitalium)*. All contraceptive methods can be continued during treatment. 

### Follow-Up

Women should demonstrate clinical improvement (e.g., defervescence; reduction in direct or rebound abdominal tenderness; and reduction in uterine, adnexal, and cervical motion tenderness) <3 days after therapy initiation. If no clinical improvement has occurred <72 hours after outpatient IM or oral therapy, then hospitalization, assessment of the antimicrobial regimen, and additional diagnostics, including consideration of diagnostic laparoscopy for alternative diagnoses, are recommended. All women who have received a diagnosis of chlamydial or gonococcal PID should be retested 3 months after treatment, regardless of whether their sex partners have been treated (*753*). If retesting at 3 months is not possible, these women should be retested whenever they next seek medical care <12 months after treatment.

### Management of Sex Partners

Persons who have had sexual contact with a partner with PID during the 60 days preceding symptom onset should be evaluated, tested, and presumptively treated for chlamydia and gonorrhea, regardless of the PID etiology or pathogens isolated. If the last sexual intercourse was >60 days before symptom onset or diagnosis, the most recent sex partner should be treated. Sex partners of persons who have PID caused by *C. trachomatis* or *N. gonorrhoeae* frequently are asymptomatic. Arrangements should be made to link sex partners to care. If linkage is delayed or unlikely, EPT is an alternative approach to treating sex partners who have chlamydial or gonococcal infection (*125*,*126*) (see Partner Services). Partners should be instructed to abstain from sexual intercourse until they and their sex partners have been treated (i.e., until therapy is completed and symptoms have resolved, if originally present).

### Special Considerations

#### Drug Allergy, Intolerance, and Adverse Reactions

The risk for penicillin cross-reactivity is highest with first-generation cephalosporins but is negligible between the majority of second-generation (e.g., cefoxitin) and all third-generation (e.g., ceftriaxone) cephalosporins (*619*,*631*,*653*,*656*) (see Management of Persons Who Have a History of Penicillin Allergy).

#### Pregnancy

Pregnant women suspected of having PID are at high risk for maternal morbidity and preterm delivery. These women should be hospitalized and treated with IV antimicrobials in consultation with an infectious disease specialist.

#### HIV Infection

Differences in PID clinical manifestations among women with HIV infection and those without have not been well delineated (*1182*). In early observational studies, women with HIV infection and PID were more likely to require surgical intervention. More comprehensive observational and controlled studies have demonstrated that women with HIV infection and PID have similar symptoms, compared with women without HIV (*1183*–*1185*), except they are more likely to have a tubo-ovarian abscess. Women with HIV responded equally well to recommended parenteral and IM or oral antibiotic regimens as women without HIV. The microbiologic findings for women with HIV and women without HIV were similar, except women with HIV had higher rates of concomitant *M. hominis* and streptococcal infections. These data are insufficient for determining whether women with HIV infection and PID require more aggressive management (e.g., hospitalization or IV antimicrobial regimens).

#### Intrauterine Devices

IUDs are one of the most effective contraceptive methods. Copper-containing and levonorgestrel-releasing IUDs are available in the United States. The risk for PID associated with IUD use is primarily confined to the first 3 weeks after insertion (*1186*–*1188*). If an IUD user receives a diagnosis of PID, the IUD does not need to be removed (*59*,*1189*). However, the woman should receive treatment according to these recommendations and should have close clinical follow-up. If no clinical improvement occurs within 48–72 hours of initiating treatment, providers should consider removing the IUD. A systematic review of evidence demonstrated that treatment outcomes did not differ between women with PID who retained the IUD and those who had the IUD removed (*1190*). These studies primarily included women using copper-containing or other nonhormonal IUDs. No studies are available regarding treatment outcomes among women using levonorgestrel-releasing IUDs.

## Epididymitis

Acute epididymitis is a clinical syndrome causing pain, swelling, and inflammation of the epididymis and lasting <6 weeks (*1191*). Sometimes a testicle is also involved, a condition referred to as epididymo-orchitis. A high index of suspicion for spermatic cord (testicular) torsion should be maintained among men who have a sudden onset of symptoms associated with epididymitis because this condition is a surgical emergency.

Acute epididymitis can be caused by STIs (e.g., *C*. *trachomatis, N. gonorrhoeae, *or* M. genitalium*) or enteric organisms (i.e., *Escherichia coli*) (*1192*). Acute epididymitis caused by an STI is usually accompanied by urethritis, which is frequently asymptomatic. Acute epididymitis caused by sexually transmitted enteric organisms might also occur among men who are the insertive partner during anal sex. Nonsexually transmitted acute epididymitis caused by genitourinary pathogens typically occurs with bacteriuria secondary to bladder outlet obstruction (e.g., benign prostatic hyperplasia) (*1193*). Among older men, nonsexually transmitted acute epididymitis is also associated with prostate biopsy, urinary tract instrumentation or surgery, systemic disease, or immunosuppression. Uncommon infectious causes of nonsexually transmitted acute epididymitis (e.g., Fournier’s gangrene) should be managed in consultation with a urologist.

Chronic epididymitis is characterized by a ≥6-week history of symptoms of discomfort or pain in the scrotum, testicle, or epididymis. Chronic infectious epididymitis is most frequently observed with conditions associated with a granulomatous reaction. *Mycobacterium tuberculosis* (TB) is the most common granulomatous disease affecting the epididymis and should be suspected, especially among men with a known history of or recent exposure to TB. The differential diagnosis of chronic noninfectious epididymitis, sometimes termed orchialgia or epididymalgia, is broad (e.g., trauma, cancer, autoimmune conditions, or idiopathic conditions). Men with this diagnosis should be referred to a urologist for clinical management (*1191*,*1192*).

### Diagnostic Considerations

Men who have acute epididymitis typically have unilateral testicular pain and tenderness, hydrocele, and palpable swelling of the epididymis. Although inflammation and swelling usually begin in the tail of the epididymis, it can spread to the rest of the epididymis and testicle. The spermatic cord is usually tender and swollen. Spermatic cord (testicular) torsion, a surgical emergency, should be considered in all cases; however, it occurs more frequently among adolescents and men without evidence of inflammation or infection. For men with severe unilateral pain with sudden onset, those whose test results do not support a diagnosis of urethritis or urinary tract infection, or for whom diagnosis of acute epididymitis is questionable, immediate referral to a urologist for evaluation for testicular torsion is vital because testicular viability might be compromised.

Bilateral symptoms should increase suspicion of other causes of testicular pain. Radionuclide scanning of the scrotum is the most accurate method for diagnosing epididymitis but it is not routinely available. Ultrasound should be used primarily for ruling out torsion of the spermatic cord in cases of acute, unilateral, painful scrotal swelling. However, because partial spermatic cord torsion can mimic epididymitis on scrotal ultrasound, differentiation between spermatic cord torsion and epididymitis when torsion is not ruled out by ultrasound should be made on the basis of clinical evaluation. Although ultrasound can demonstrate epididymal hyperemia and swelling associated with epididymitis, it provides minimal diagnostic usefulness for men with a clinical presentation consistent with epididymitis. A negative ultrasound does not rule out epididymitis and thus does not alter clinical management. Ultrasound should be reserved for men if torsion of the spermatic cord is suspected or for those with scrotal pain who cannot receive an accurate diagnosis by history, physical examination, and objective laboratory findings.

All suspected cases of acute epididymitis should be evaluated for objective evidence of inflammation by one of the following POC tests:

- Gram, MB, or GV stain of urethral secretions demonstrating ≥2 WBCs per oil immersion field (*737*) (see Urethritis). These stains are preferred POC diagnostic tests for evaluating urethritis because they are highly sensitive and specific for documenting both urethral inflammation and presence or absence of gonococcal infection. Gonococcal infection is established by documenting the presence of WBC-containing intracellular gram-negative or purple diplococci on urethral Gram, MB, or GV stain, respectively.
- Positive leukocyte esterase test on first-void urine.
- Microscopic examination of sediment from a spun first-void urine demonstrating ≥10 WBCs/HPF.

All suspected cases of acute epididymitis should be tested for *C. trachomatis* and *N. gonorrhoeae* by NAAT. Urine is the preferred specimen for NAAT for men (*553*). Urine cultures for chlamydial and gonococcal epididymitis are insensitive and are not recommended. Urine bacterial cultures should also be performed for all men to evaluate for the presence of genitourinary organisms and to determine antibiotic susceptibility.

### Treatment

To prevent complications and transmission of STIs, presumptive therapy for all sexually active men is indicated at the time of the visit before all laboratory test results are available. Selection of presumptive therapy is based on risk for chlamydial and gonococcal infections or enteric organisms. Treatment goals for acute epididymitis are 1) microbiologic infection cure, 2) improvement of signs and symptoms, 3) prevention of transmission of chlamydia and gonorrhea to others, and 4) decreased potential for chlamydial or gonococcal epididymitis complications (e.g., infertility or chronic pain). Although the majority of men with acute epididymitis can be treated on an outpatient basis, referral to a specialist and hospitalization should be considered when severe pain or fever indicates other diagnoses (e.g., torsion, testicular infarction, abscess, or necrotizing fasciitis) or when men are unable to comply with an antimicrobial regimen. Age, history of diabetes, fever, and elevated C-reactive protein can indicate more severe disease requiring hospitalization (*1193*).

**Recommended Regimens for Epididymitis**
**For acute epididymitis most likely caused by chlamydia or gonorrhea: Ceftriaxone** 500 mg* IM in a single dose
*plus*
**Doxycycline** 100 mg orally 2 times/day for 10 days
**For acute epididymitis most likely caused by chlamydia, gonorrhea, or enteric organisms (men who practice insertive anal sex): Ceftriaxone** 500 mg* IM in a single dose
*plus*
**Levofloxacin **500 mg orally once daily for 10 days
**For acute epididymitis most likely caused by enteric organisms only: Levofloxacin** 500 mg orally once daily for 10 days
* For persons weighing ≥150 kg, 1 g of ceftriaxone should be administered.

Levofloxacin monotherapy should be considered if the infection is most likely caused by enteric organisms only, and gonorrhea has been ruled out by Gram, MB, or GV stain. This includes men who have undergone prostate biopsy, vasectomy, and other urinary tract instrumentation procedures. Treatment should be guided by bacterial cultures and antimicrobial susceptibilities. As an adjunct to therapy, bed rest, scrotal elevation, and nonsteroidal anti-inflammatory drugs are recommended until fever and local inflammation have subsided. Complete resolution of discomfort might not occur for a few weeks after completion of the antibiotic regimen.

### Other Management Considerations

Men who have acute epididymitis confirmed or suspected to be caused by *N. gonorrhoeae* or *C. trachomatis* should be advised to abstain from sexual intercourse until they and their partners have been treated and symptoms have resolved. All men with acute epididymitis should be tested for HIV and syphilis.

### Follow-Up

Men should be instructed to return to their health care providers if their symptoms do not improve <72 hours after treatment. Signs and symptoms of epididymitis that do not subside in <3 days require reevaluation of the diagnosis and therapy. Men who experience swelling and tenderness that persist after completion of antimicrobial therapy should be evaluated for alternative diagnoses, including tumor, abscess, infarction, testicular cancer, TB, and fungal epididymitis.

### Management of Sex Partners

Men who have acute sexually transmitted epididymitis confirmed or suspected to be caused by *N. gonorrhoeae* or *C. trachomatis* should be instructed to refer all sex partners during the previous 60 days before symptom onset for evaluation, testing, and presumptive treatment (see Chlamydial Infections; Gonococcal Infections). If the last sexual intercourse was >60 days before onset of symptoms or diagnosis, the most recent sex partner should be evaluated and treated. Arrangements should be made to link sex partners to care. EPT is an effective strategy for treating sex partners of men who have or are suspected of having chlamydia or gonorrhea for whom linkage to care is anticipated to be delayed (*125*,*126*) (see Partner Services). Partners should be instructed to abstain from sexual intercourse until they and their sex partners are treated and symptoms have resolved.

### Special Considerations

#### Drug Allergy, Intolerance, and Adverse Reactions

The risk for penicillin cross-reactivity is negligible between all third-generation cephalosporins (e.g., ceftriaxone) (*658*,*681*) (see Management of Persons Who Have a History of Penicillin Allergy). Alternative regimens have not been studied; therefore, clinicians should consult an infectious disease specialist if such regimens are required.

#### HIV Infection

Men with HIV infection who have uncomplicated acute epididymitis should receive the same treatment regimen as those who do not have HIV. Other etiologic agents have been implicated in acute epididymitis among men with HIV, including CMV, salmonella, toxoplasmosis, *U. urealyticum*, *Corynebacterium* species, *Mycoplasma* species, and *Mima polymorpha* (*1192*)*.*

## Human Papillomavirus Infections

Approximately 150 types of HPV have been identified, at least 40 of which infect the genital area (*1194*). The majority of HPV infections are self-limited and are asymptomatic or unrecognized. Sexually active persons are usually exposed to HPV during their lifetime (*838*,*1195*,*1196*). Oncogenic, high-risk HPV infection (e.g., HPV types 16 and 18) causes the majority of cervical, penile, vulvar, vaginal, anal, and oropharyngeal cancers and precancers (*1197*), whereas other HPV infection (e.g., HPV types 6 and 11) causes genital warts and recurrent respiratory papillomatosis. Persistent oncogenic HPV infection is the strongest risk factor for development of HPV-attributable precancers and cancers. A substantial proportion of cancers and anogenital warts are attributable to HPV in the United States. An estimated 34,800 new HPV-attributable cancers occurred every year during 2012–2016 (*1198*). Before HPV vaccines were introduced, approximately 355,000 new cases of anogenital warts occurred every year (*1199*).

### Prevention

#### HPV Vaccines

Three HPV vaccines are licensed in the United States: Ceravrix, a 2-valent vaccine (2vHPV) that targets HPV types 16 and 18; Gardasil, a 4-valent vaccine (4vHPV) that targets HPV types 6, 11, 16, and 18; and Gardasil 9, a 9-valent vaccine (9vHPV) that targets HPV types 6, 11, 16, 18, 31, 33, 45, 52, and 58. Types 16 and 18 account for 66% of all cervical cancers, whereas the five additional types targeted by the 9-valent vaccine account for 15%. Types 6 and 11 cause >90% of genital warts. Only 9vHPV vaccine is available in the United States.

ACIP recommendations for HPV vaccination (https://www.cdc.gov/vaccines/hcp/acip-recs/vacc-specific/hpv.html) include the following:

- Routine HPV vaccination for all adolescents at age 11 or 12 years.
- Administering vaccine starting at age 9 years.
- Catch-up vaccination through age 26 years for those not vaccinated previously.
- Not using HPV vaccination for all adults aged >26 years. Instead, shared clinical decision-making between a patient and a provider regarding HPV vaccination is recommended for certain adults aged 27–45 years not vaccinated previously.
- A 2-dose vaccine schedule (at 0- and 6–12-month intervals) is recommended for persons who initiate vaccination before their 15th birthday.
- A 3-dose vaccine schedule (at 0-, 1–2-, and 6-month intervals) for immunocompromised persons regardless of age of initiation.

HPV vaccines are not recommended for use in pregnant women. HPV vaccines can be administered regardless of history of anogenital warts, abnormal Pap test or HPV test, or anogenital precancer. Women who have received HPV vaccine should continue routine cervical cancer screening (see Cervical Cancer). HPV vaccine is available for eligible children and adolescents aged <19 years through the Vaccines for Children (VFC) program (additional information is available at https://www.cdc.gov/vaccines/programs/vfc/index.html or by calling CDC INFO 800-232-4636). For uninsured persons aged <19 years, patient assistance programs are available from the vaccine manufacturers. Prelicensure and postlicensure safety evaluations have determined that the vaccine is well tolerated. With >120 million doses of HPV vaccines distributed in the United States, robust data demonstrate that HPV vaccines are safe (https://www.cdc.gov/vaccinesafety). Impact-monitoring studies in the United States have demonstrated reductions of genital warts as well as the HPV types contained within the quadrivalent vaccine (*1200*–*1203*). Settings that provide STI services should either administer the vaccine to eligible clients within the routine and catch-up age groups through age 26 years who have not started or completed the vaccine series, or link these persons to another facility equipped to provide the vaccine. Clinicians providing services to children, adolescents, and young adults should be knowledgeable about HPV and the vaccine (https://www.cdc.gov/vaccines/who/teens/for-hcp/hpv-resources.html). HPV vaccination has not been associated with initiation of sexual activity or sexual risk behaviors (*1204*,*1205*).

Abstaining from sexual activity is the most reliable method for preventing genital HPV infection. Persons can decrease their chances of infection by practicing consistent and correct condom use and limiting their number of sex partners. Although these interventions might not fully protect against HPV, they can decrease the chances of HPV acquisition and transmission.

### Diagnostic Considerations

HPV tests are available for detecting oncogenic types of HPV infection and are used in the context of cervical cancer screening and management or follow-up of abnormal cervical cytology or histology (see Cervical Cancer). These tests should not be used for male partners of women with HPV or women aged <25 years, for diagnosis of genital warts, or as a general STI test.

Application of 3%–5% acetic acid, which might cause affected areas to turn white, has been used by certain providers to detect genital mucosa infected with HPV. The routine use of this procedure to detect mucosal changes attributed to HPV infection is not recommended because the results do not influence clinical management.

### Treatment

Treatment is directed to the macroscopic (e.g., genital warts) or pathologic precancerous lesions caused by HPV. Subclinical genital HPV infection typically clears spontaneously; therefore, specific antiviral therapy is not recommended to eradicate HPV infection. Precancerous lesions are detected through cervical cancer screening; HPV-related precancer should be managed on the basis of existing guidance (see Cervical Cancer).

### Counseling

#### Key Messages for Persons with Human Papillomavirus Infection

When counseling persons with anogenital HPV infection, the provider should discuss the following:

- Anogenital HPV infection is common. It usually infects the anogenital area but can infect other areas, including the mouth and throat. The majority of sexually active persons get HPV at some time during their lifetime, although most never know it.
- Partners tend to share HPV, and it is not possible to determine which partner transmitted the original infection. Having HPV does not mean that a person or his or her partner is having sex outside the relationship.
- Persons who acquire HPV usually clear the infection spontaneously, meaning that HPV becomes undetectable with no associated health problems.
- If HPV infection persists, genital warts, precancers, and cancers of the cervix, anus, penis, vulva, vagina, head, or neck might develop.
- Discussion of tobacco use, and provision of cessation counseling, is important because of its contribution to the progression of precancer and cancer.
- The types of HPV that cause genital warts are different from the types that can cause cancer.
- Many types of HPV are sexually transmitted through anogenital contact, mainly during vaginal and anal sex. HPV also might be transmitted during oral sex and genital-to-genital contact without penetration. In rare cases, a pregnant woman can transmit HPV to an infant during delivery.
- Treatments are available for the conditions caused by HPV but not for the virus itself.
- Having HPV does not make it harder for a woman to get pregnant or carry a pregnancy to term. However, certain precancers or cancers that HPV can cause, and the surgical procedures needed to treat them, can affect a woman’s ability to get pregnant or carry a pregnancy to term.
- No HPV test can determine which HPV infection will become undetectable and which will persist or progress to disease. However, in certain circumstances, HPV tests can determine whether a woman is at increased risk for cervical cancer. These tests are not for detecting other HPV-related problems, nor are they useful for women aged <25 years or men of any age.

##### Prevention

- Three HPV vaccines can prevent diseases and cancers caused by HPV. The 2vHPV, 4vHPV, and 9vHPV vaccines protect against the majority of cervical cancer cases, although the 4vHPV and 9vHPV vaccines also protect against the majority of genital warts. Only 9vHPV vaccine is available in the United States. HPV vaccines are safe and effective and are recommended routinely for adolescents aged 11–12 years. Catch-up vaccination is also recommended for older adolescents and young adults through age 26 years (https://www.cdc.gov/hpv/hcp/index.html). Shared clinical decision-making is recommended regarding HPV vaccination for certain adults aged 27–45 years who are not adequately vaccinated per guidance (https://www.cdc.gov/mmwr/volumes/68/wr/pdfs/mm6832a3-H.pdf).
- Condoms used consistently and correctly can lower the chances of acquiring and transmitting HPV and developing HPV-related diseases (e.g., genital warts or cervical cancer). However, because HPV can infect areas not covered by a condom, condoms might not fully protect against HPV.
- Limiting the number of sex partners can reduce the risk for HPV. However, even persons with only one lifetime sex partner can get HPV.
- Abstaining from sexual activity is the most reliable method for preventing genital HPV infection.

### Anogenital Warts

Anogenital warts are a common disease, and 90% are caused by nononcogenic HPV types 6 or 11. These types can be commonly identified before or at the same time anogenital warts are detected (*1206*). HPV types 16, 18, 31, 33, and 35 also are occasionally identified in anogenital warts (usually as infections with HPV 6 or 11) and can be associated with foci of high-grade squamous intraepithelial lesion (HSIL), particularly among persons who have HIV infection. In addition to anogenital warts, HPV types 6 and 11 have been associated with conjunctival, nasal, oral, and laryngeal warts.

Anogenital warts are usually asymptomatic; however, depending on the size and anatomic location, they can be painful or pruritic. They are usually flat, papular, or pedunculated growths on the genital mucosa. Anogenital warts occur commonly at certain anatomic sites, including around the vaginal introitus, under the foreskin of the uncircumcised penis, and on the shaft of the circumcised penis. Warts can also occur at multiple sites in the anogenital epithelium or within the anogenital tract (e.g., cervix, vagina, urethra, perineum, perianal skin, anus, or scrotum). Intra-anal warts are observed predominantly in persons who have had receptive anal intercourse; however, they also can occur among men and women who have not had a history of anal sexual contact.

#### Prevention

Anogenital warts have decreased among adolescents, young women, and heterosexual men with use of HPV vaccination in multiple countries, including the United States (*1203*,*1207*–*1216*).

#### Diagnostic Considerations

Diagnosis of anogenital warts is usually made by visual inspection but can be confirmed by biopsy, which is indicated if lesions are atypical (e.g., pigmented, indurated, affixed to underlying tissue, bleeding, or ulcerated lesions). Biopsy might also be indicated in the following circumstances, particularly if the patient is immunocompromised (including those with HIV infection): the diagnosis is uncertain, the lesions do not respond to standard therapy, or the disease worsens during therapy. HPV testing is not recommended for anogenital wart diagnosis because test results are not confirmatory and do not guide genital wart management. Some anogenital lesions can resemble anogenital warts (condyloma accuminata), but do not respond to anogenital wart treatment. Condyloma lata, a manifestation of secondary syphilis, can be diagnosed by serologic tests or through direct detection from serous fluid from the lesions (see Syphilis, Diagnostic Considerations).

#### Treatment

The aim of treatment is removal of the warts and amelioration of symptoms, if present. The appearance of warts also can result in considerable psychosocial distress, and removal can relieve cosmetic concerns. For most patients, treatment results in resolution of the warts. If left untreated, anogenital warts can resolve spontaneously, remain unchanged, or increase in size or number. Because warts might spontaneously resolve in <1 year, an acceptable alternative for certain persons is to forego treatment and wait for spontaneous resolution. Available therapies for anogenital warts might reduce, but probably do not eradicate, HPV infectivity. Whether reduction in HPV viral DNA resulting from treatment reduces future transmission remains unknown.

Treatment of anogenital warts should be guided by wart size, number, and anatomic site; patient preference; cost of treatment; convenience; adverse effects; and provider experience. No definitive evidence indicates that any one recommended treatment is superior to another, and no single treatment is ideal for all patients or all warts. Shared clinical decision-making between a patient and a provider regarding treatment algorithms has been associated with improved clinical outcomes and should be encouraged. Because all available treatments have shortcomings, clinicians sometimes use combination therapy (e.g., provider-administered cryotherapy with patient-applied topical therapy between visits to the provider). However, limited data exist regarding the efficacy or risk for complications associated with combination therapy. Treatment regimens are classified as either patient-applied or provider-administered modalities. Patient-applied modalities are preferred by certain persons because they can be administered in the privacy of their home. To ensure that patient-applied modalities are effective, instructions should be provided to patients while in the clinic, and all anogenital warts should be accessible and identified during the clinic visit. Follow-up visits after weeks of therapy enable providers to answer any questions about use of the medication, address any side effects experienced, and facilitate assessment of the response to treatment.

Recommended Regimens for External Anogenital Warts (i.e., Penis, Groin, Scrotum, Vulva, Perineum, External Anus, or Perianus)*
**Patient-applied:** **Imiquimod 3.75% or 5% cream^†^**
*or*
**Podofilox 0.5% solution or gel**
*or*
**Sinecatechins 15% ointment^†^**
**Provider-administered: Cryotherapy** with liquid nitrogen or cryoprobe
*or*
**Surgical removal** by tangential scissor excision, tangential shave excision, curettage, laser, or electrosurgery
*or*
**Trichloroacetic acid (TCA) or bichloroacetic acid (BCA) 80%–90% solution**
* Persons with external anal or perianal warts might also have intra-anal warts. Thus, persons with external anal warts might benefit from an inspection of the anal canal by digital examination, standard anoscopy, or high-resolution anoscopy.
^†^ Might weaken condoms and vaginal diaphragms.

Imiquimod is a patient-applied, topically active immune enhancer that stimulates production of interferon and other cytokines. Imiquimod 5% cream should be applied once at bedtime, 3 times/week for <16 weeks (*1217*). Similarly, imiquimod 3.75% cream should be applied once at bedtime every night for <8 weeks (*1218*). With either formulation, the treatment area should be washed with soap and water 6–10 hours after the application. Local inflammatory reactions, including redness, irritation, induration, ulceration or erosion, and vesicles might occur with using imiquimod, and hypopigmentation has also been described (*1219*). Limited case reports demonstrate an association between treatment with imiquimod cream and worsened inflammatory or autoimmune skin diseases (e.g., psoriasis, vitiligo, or lichenoid dermatoses) (*1220*–*1222*). Data from studies of human participants are limited regarding use of imiquimod during pregnancy; however, animal data indicate that this therapy poses low risk (*431*).

Podofilox (podophyllotoxin) is a patient-applied antimitotic drug that causes wart necrosis. Podofilox solution (using a cotton swab) or podofilox gel (using a finger) should be applied to anogenital warts 2 times/day for 3 days, followed by 4 days of no therapy. This cycle can be repeated, as necessary, for up to four cycles. The total wart area treated should not exceed 10 cm^2^, and the total volume of podofilox should be limited to 0.5 mL/day. If possible, the health care provider should apply the initial treatment to demonstrate proper application technique and identify which warts should be treated. Mild to moderate pain or local irritation might develop after treatment. After each treatment, the gel or solution should be allowed to dry. Patients should wash their hands before and after each application. Podofilox is contraindicated during pregnancy (*431*).

Sinecatechins is a patient-applied, green-tea extract with an active product (catechins). Sinecatechins 15% ointment should be applied 3 times/day (0.5-cm strand of ointment to each wart) by using a finger to ensure coverage with a thin layer of ointment until complete clearance of warts is achieved. This product should not be continued for >16 weeks (*1223*–*1225*). The medication should not be washed off after use. Genital, anal, and oral sexual contact should be avoided while the ointment is on the skin. The most common side effects of sinecatechins are erythema, pruritus or burning, pain, ulceration, edema, induration, and vesicular rash. This medication is not recommended for persons with HIV infection, other immunocompromised conditions, or genital herpes because the safety and efficacy of therapy has not been evaluated. The safety of sinecatechins during pregnancy is unknown.

Cryotherapy is a provider-administered therapy that destroys warts by thermal-induced cytolysis. Health care providers should be trained on the correct use of this therapy because overtreatment or undertreatment can result in complications or low efficacy. Pain during and after application of the liquid nitrogen, followed by necrosis and sometimes blistering, is common. Local anesthesia (topical or injected) might facilitate therapy if warts are present in many areas or if the area of warts is large. Surgical therapy has the advantage of eliminating the majority of warts at a single visit, although recurrence can occur. Surgical removal requires substantial clinical training, additional equipment, and sometimes a longer office visit. After local anesthesia is applied, anogenital warts can be physically destroyed by electrocautery, in which case no additional hemostasis is required. Care should be taken to control the depth of electrocautery to prevent scarring. Alternatively, the warts can be removed either by tangential excision with a pair of fine scissors or a scalpel, by CO_2_ laser, or by curettage. Because most warts are exophytic, this procedure can be accomplished with a resulting wound that only extends into the upper dermis. Hemostasis can be achieved with an electrocautery unit or, in cases of minor bleeding, a chemical styptic (e.g., an aluminum chloride solution). Suturing is neither required nor indicated in the majority of cases. For patients with large or extensive warts, surgical therapy, including CO_2_ laser, might be most beneficial; such therapy might also be useful for intraurethral warts, particularly for those persons whose warts have not responded to other treatments. Treatment of anogenital and oral warts should be performed in a ventilated room by using standard precautions (https://www.cdc.gov/infectioncontrol/guidelines/isolation/index.html/Isolation2007.pdf#page) and local exhaust ventilation (e.g., a smoke evacuator) (*1226*).

Trichloroacetic acid (TCA) and bichloroacetic acid (BCA) are provider-administered caustic agents that destroy warts by chemical coagulation of proteins. Although these preparations are widely used, they have not been investigated thoroughly. TCA solution has a low viscosity, comparable with that of water, and can spread rapidly and damage adjacent tissues if applied excessively. A small amount should be applied only to the warts and allowed to dry (i.e., develop white frost on tissue) before the patient sits or stands. If pain is intense or an excess amount of acid is applied, the area can be covered with sodium bicarbonate (i.e., baking soda), washed with liquid soap preparations, or be powdered with talc to neutralize the acid or remove unreacted acid. TCA or BCA treatment can be repeated weekly if necessary.

##### Alternative Regimens for External Genital Warts

Fewer data are available regarding the efficacy of alternative regimens for treating anogenital warts, which include podophyllin resin, intralesional interferon, photodynamic therapy, and topical cidofovir. Shared clinical decision-making between the patient and provider regarding benefits and risks of these regimens should be provided. In addition, alternative regimens might be associated with more side effects. Podophyllin resin is no longer a recommended regimen because of the number of safer regimens available, and severe systemic toxicity has been reported when podophyllin resin was applied to large areas of friable tissue and was not washed off within 4 hours (*1227*–*1229*). Podophyllin resin 10%–25% in a compound tincture of benzoin might be considered for provider-administered treatment under conditions of strict adherence to recommendations. Podophyllin should be applied to each wart and then allowed to air dry before the treated area comes into contact with clothing. Overapplication or failure to air dry can result in local irritation caused by spread of the compound to adjacent areas and possible systemic toxicity. The treatment can be repeated weekly, if necessary. To avoid the possibility of complications associated with systemic absorption and toxicity, application should be limited to <0.5 mL of podophyllin or an area of <10 cm^2^ of warts per session; the area to which treatment is administered should not contain any open lesions, wounds, or friable tissue; and the preparation should be thoroughly washed off 1–4 hours after application. Podophyllin resin preparations differ in the concentration of active components and contaminants. Shelf life and stability of podophyllin preparations are unknown. The safety of podophyllin during pregnancy has not been established.

**Recommended Regimens for Urethral Meatus Warts**
**Cryotherapy** with liquid nitrogen
*or*
**Surgical removal**
**Recommended Regimens for Vaginal Warts**
**Cryotherapy** with liquid nitrogen
The use of a cryoprobe in the vagina is not recommended because of the risk for vaginal perforation and fistula formation.
*or*
**Surgical removal**
*or*
**Trichloracetic acid (TCA) or bichloroacetic acid (BCA) 80%–90% solution**
**Recommended Regimens for Cervical Warts**
**Cryotherapy** with liquid nitrogen
*or*
***Surgical removal***
*or*
**Trichloracetic acid (TCA) or bichloroacetic acid (BCA) 80%–90% solution**
Management of cervical warts should include consultation with a specialist. For women who have exophytic cervical warts, a biopsy evaluation to exclude HSIL should be performed before treatment is initiated.
**Recommended Regimens for Intra-Anal Warts**
**Cryotherapy** with liquid nitrogen
***or***
**Surgical removal**
***or***
**Trichloracetic acid (TCA) or bichloroacetic acid (BCA) 80%–90% solution**
Management of intra-anal warts should include consultation with a colorectal specialist.

#### Follow-Up

Anogenital warts typically respond within 3 months of therapy. Factors that might affect response to therapy include immunosuppression and treatment compliance. Warts located on moist surfaces or in intertriginous areas respond best to topical treatment. A new treatment modality should be selected when no substantial improvement is observed after a complete course of treatment or in the event of severe side effects; treatment response and therapy-associated side effects should be evaluated throughout the therapy course. Complications occur rarely when treatment is administered correctly. Persistent hypopigmentation or hyperpigmentation can occur with ablative modalities (e.g., cryotherapy and electrocautery) and have been described with immune modulating therapies (e.g., imiquimod cream). Depressed or hypertrophic scars are uncommon but can occur, especially if patients have insufficient time to heal between treatments. Rarely, treatment can result in chronic pain syndromes (e.g., vulvodynia and hyperesthesia of the treatment site) or, in the case of anal warts, painful defecation or fistulas.

#### Counseling

When counseling persons with anogenital warts, the provider should discuss the following:

- If left untreated, genital warts might resolve, stay the same, or increase in size or number. The types of HPV that cause genital warts are different from the types that can cause cancer.
- Women with genital warts do not need Pap tests more often than other women.
- Time of HPV acquisition cannot be definitively determined. Genital warts can develop months or years after acquiring HPV.
- HPV types that cause genital warts can be passed on to another person, even without visible signs of warts. Sex partners tend to share HPV, even though signs of HPV (e.g., warts) might occur in only one or neither partner.
- Although genital warts are common and benign, certain persons might experience considerable psychosocial impact after receiving this diagnosis.
- Although genital warts can be treated, such treatment does not cure the virus itself. For this reason, genital warts often recur after treatment, especially during the first 3 months.
- Because genital warts can be sexually transmitted, persons with genital warts benefit from testing for other STIs. HPV might remain present and can still be transmitted to partners even after the warts are gone.
- Condoms might lower the chances of transmitting genital warts if used consistently and correctly; however, HPV can infect areas that are not covered by a condom and might not fully protect against HPV.
- A vaccine is available for males and females to prevent genital warts (Gardasil 9) but it will not treat existing HPV or genital warts. This vaccine can prevent the majority of cases of genital warts among persons who have not yet been exposed to wart-causing types of HPV.

#### Management of Sex Partners

Persons should inform current partners about having genital warts because the types of HPV that cause warts can be passed on to partners. Partners should be counseled that they might already have HPV despite no visible signs of warts; therefore, HPV testing of sex partners of persons with genital warts is not recommended. Partners might benefit from a physical examination to detect genital warts and tests for other STIs. No recommendations can be made regarding informing future sex partners about a diagnosis of genital warts because the duration of viral persistence after warts have resolved is unknown.

#### Special Considerations

##### Pregnancy

Podofilox, podophyllin, and sinecatechins should not be used during pregnancy. Imiquimod appears to pose low risk but should be avoided until more data are available. Anogenital warts can proliferate and become friable during pregnancy. Although removal of warts during pregnancy can be considered, resolution might be incomplete or poor until pregnancy is complete. Rarely, HPV types 6 and 11 can cause respiratory papillomatosis among infants and children, although the route of transmission (i.e., transplacental, perinatal, or postnatal) is not completely understood. Whether cesarean delivery prevents respiratory papillomatosis among infants and children also is unclear (*1230*); therefore, cesarean delivery should not be performed solely to prevent transmission of HPV infection to the newborn. Cesarean delivery is indicated for women with anogenital warts if the pelvic outlet is obstructed or if vaginal delivery would result in excessive bleeding. Pregnant women with anogenital warts should be counseled about the low risk for warts on the larynx of their infants or children (recurrent respiratory papillomatosis).

##### HIV and Other Causes of Immunosuppression

Persons with HIV infection or who are otherwise immunosuppressed are more likely to develop anogenital warts than those who do not have HIV (*1231*). Moreover, such persons can have larger or more numerous lesions, might not respond to therapy as well as those who are immunocompetent, and might have more frequent recurrences after treatment (*1231*,*1232*–*1234*). Despite these factors, data do not support altered approaches to treatment for persons with HIV infection. Squamous cell carcinomas arising in or resembling anogenital warts might occur more frequently among immunosuppressed persons, therefore requiring biopsy for confirmation of diagnosis for suspicious cases (*1235*–*1237*).

##### High-Grade Squamous Intraepithelial Lesions

Biopsy of an atypical wart might reveal HSIL or cancer of the anogenital tract. In this instance, referral to a specialist for treatment is recommended.

##### Cancers and Precancers Associated with Human Papillomavirus

Persistent infection with high-risk (oncogenic) types of HPV has a causal role in approximately all cervical cancers and in certain vulvar, vaginal, penile, anal, and oropharyngeal cancers (*1238*). However, cervical cancer is the only HPV-associated cancer for which routine screening is recommended.

### Cervical Cancer

#### Screening Recommendations

Recommendations for cervical cancer screening in the United States are based on systematic evidence reviews by major medical and advocacy organizations, including USPSTF (*174*), ACS (*177*), and ACOG (*175*). Over time, general alignment across these organizations has emerged as to when to start and end cervical cancer screening as well as the periodicity of screening. Although no single guideline universally guides screening practices in the United States, the Patient Protection and Affordable Care Act required Medicaid and new private health insurance plans to provide coverage for preventive services graded A or B by USPSTF, which includes cervical cancer screening. In addition, the National Center for Quality Assurance provides a set of measures (the Healthcare Effectiveness Data and Information Set [HEDIS]) for up-to-date cervical cancer screening that aligns with USPSTF recommendations (https://www.ncqa.org/hedis/measures/cervical-cancer-screening). The Center for Medicaid and Medicare Services uses the same measure as HEDIS to measure cervical cancer screening performance.

USPSTF screening recommendations apply to persons with a cervix at average risk, defined as those with no previous cervical cancer or high-grade precancer, not currently under close follow-up for a recent abnormal result, not immunocompromised (e.g., persons with HIV), and who had no exposure to diethylstilbestrol in utero. Among these persons, screening should be performed starting at age 21 years and continue through age 65 years. Testing can be performed using either conventional or liquid-based cytologic tests (i.e., Pap tests). For persons aged ≥30 years, screening can include FDA-cleared tests for high-risk, oncogenic types of HPV. For cytopathologic testing, clinics should use CLIA-certified laboratories using acceptable terminology (Bethesda 2001 or LAST terminology) (*1239*).

Annual cervical cancer screening is not recommended for persons at average risk. Instead, cytology testing is recommended every 3 years for persons aged 21–29 years. For persons aged 30–65 years, a cytology test every 3 years, an HPV test alone every 5 years, or a cytology test plus an HPV test (cotest) every 5 years is recommended. Cotesting can be done by either collecting one sample for the cytology test and another for the HPV test or by using the remaining liquid cytology material for the HPV test. Cervical screening programs should screen those who have received HPV vaccination in the same manner as those that are unvaccinated. Screening is not recommended before age 21 years among those at average risk. For those aged 30–65 years, cytology alone or primary HPV testing is preferred by USPSTF; however, cotesting can be used as an alternative approach. ACOG (*1240*), ACS (*177*), and USPSTF (*174*) each have screening recommendations (*1241*) (Table 1).

**TABLE 1:** Cervical cancer screening and surveillance recommendations

Population	Screening specifics	Guideline group, yr of recommendation			
		USPSTF, 2018	ACOG, 2016		ACS, 2020
Persons at average risk	Age to start screening	21 yrs	21 yrs		25 yrs
	Age to end screening	65 yrs	65 yrs		65 yrs
		If three consecutive negative cytology tests or two negative cytology plus HPV tests or two negative HPV tests (ACS) with the most recent within the previous 5 yrs and no abnormal tests within the previous 10 yrs (ACS) and no CIN 2 or CIN 3 within the previous 25 yrs			
	Screening test options and intervals	Aged 21–65 yrs: Cytology alone every 3 yrs ***or ***Aged 21–29 yrs: Cytology alone every 3 yrs Aged 30–65 yrs: Cytology plus HPV testing every 5 yrs ***or ***Aged 21–29 yrs: Cytology alone every 3 yrs Aged 30–65: HPV testing alone every 5 yrs*			HPV testing alone every 5 yrs ***or ***Cytology plus HPV testing every 5 yrs ***or*** Cytology alone every 3 yrs
	Preferred strategies	Cytology alone every 3 yrs and HPV testing alone every 5 yrs (equally preferred)	Cytology plus HPV testing every 5 yrs		HPV testing alone every 5 yrs
	Previous hysterectomy with removal of cervix	Screening not recommended after hysterectomy for benign indications Surveillance testing recommended for previous diagnosis of high-grade precancer, AIS, or cancer			
Persons with an immunocompromising medical condition^†^ (e.g., HIV infection or solid organ transplantation)	Age to start screening	No specific recommendation	Within 1 yr of onset of sexual activity or, if already sexually active, within the first year after HIV or other immunocompromising medical condition diagnosis but no later than age 21 yrs		
	Age to end screening		None; lifelong screening recommended		
	Screening test options and intervals		Aged 21–65 yrs: Cytology every year; after three consecutive annual normal cytology test results, screening can be every 3 yrs ***or ***Aged 21–29 yrs: Cytology every year Aged 30–65 yrs: Cytology plus HPV testing every 3 yrs		
	Previous hysterectomy with removal of cervix		Not specified		
Persons with in utero exposure to diethylstilbestrol^§^	Age to start screening	No specific recommendation	Not specified	No specific recommendation	
	Age to end screening		Not specified		
	Screening test options and intervals		Cytology alone annually		
	Previous hysterectomy with removal of cervix		Not specified		
Persons who have received HPV vaccination	No changes to the screening approaches above				
**Population**	**Screening specifics**	**ASCCP, 2019, and ACOG, 2020**			
Persons with a diagnosis of CIN 2 or CIN 3 (histologic HSIL^¶^) within the previous 25 yrs	Age to start screening	Not applicable			
	Age to end screening	May end at age 65 yrs if CIN diagnosis ≥25 yrs ago and criteria for ending screening met, otherwise continue screening past age 65 yrs Continued screening for ≥25 yrs after diagnosis is acceptable if patient is in good health			
	Screening test options and intervals	**Initial surveillance:** HPV testing alone or cytology plus HPV testing at 6, 18, and 30 mos ***or*** Cytology at 6, 12, 18, 24, and 30 mos **Long-term surveillance:** HPV testing alone or cytology plus HPV testing every 3 yrs ***or*** Cytology alone annually Continue for ≥25 yrs from the initial CIN diagnosis, even if extends past age 65 yrs Routine screening can resume after the posttreatment surveillance period			
	Previous hysterectomy with removal of cervix	HPV testing alone or cytology plus HPV testing every 3 yrs ***or*** Cytology alone annually Continue for ≥25 yrs from the initial CIN diagnosis, even if extends past age 65 yrs			

**Source:** Perkins R, Guido R, Saraiya M, et al. Summary of current guidelines for cervical cancer screening and management of abnormal test results: 2016–2020. J Womens Health (Larchmt) 2021;30:5–13.

**Abbreviations:** ACS = American Cancer Society; ACOG = American College of Obstetricians and Gynecologists; AIS = adenocarcinoma in situ; ASCCP = American Society for Colposcopy and Cervical Pathology; CIN = cervical intraepithelial neoplasia; HPV = human papillomavirus; HSIL = high-grade squamous intraepithelial lesion; USPSTF = U.S. Preventive Services Task Force.

* Considered an alternative screening strategy by ACOG.

^† ^Panel for Opportunistic Infections, ACOG, 2016.

^§ ^ACOG, 2016.

^¶^ Either by cytology or by histology; includes a persistent cytologic diagnosis of atypical squamous cells, cannot rule out HSIL.

Clinics should weigh the benefits of each screening strategy as well as their resources, such as time and cost, in deciding on which of the three possible screening strategies to implement. Decision analytic models (*1242*) estimating the benefits, harms, and costs (*1243*) of several different strategies might be useful in making this determination (*174*,*1244*,*1245*). Adopting recommended screening and follow-up procedures, including screening methods, results provision, and follow-up, can lead to success in implementing cervical cancer screening in clinics (*1246*).

Patients should be provided a copy of their test results; those with normal results should be provided information on follow-up visits and the importance of continued cervical cancer screening, if applicable. Those with abnormal screening tests should be managed per published guidelines. National consensus guidelines are available for the management of abnormal cervical cancer screening tests (*1247*). HPV testing or cotesting is preferred to cytology alone for surveillance after an abnormal screening test result. These guidelines base management recommendations on case-by-case assessment of risk considering past screening history and current results (see Follow-Up). Patients with abnormal cervical cancer screening test results should be counseled about those results (see Counseling Messages).

The following additional management considerations are associated with performing Pap tests and HPV tests:

- Cytology (Pap tests) and HPV tests should not be considered screening tests for STIs.
- All persons with a cervix should receive cervical cancer screening, regardless of sexual orientation or gender identity (i.e., those who identify as lesbian, bisexual, heterosexual, or transgender).
- A conventional cytology test (in which the sample is smeared onto a dry slide) should ideally be scheduled for 10–20 days after the first day of menses. Liquid-based cytology can be performed at any time during the menstrual cycle.
- If specific infections other than HPV (e.g., chlamydia or gonorrhea) are identified at the visit, a repeat cytology test after appropriate treatment for those infections might be indicated. However, in most instances (even in the presence of certain severe cervical infections), cytology tests will be reported as satisfactory for evaluation, and reliable final reports can be produced without the need to repeat the cytology test after treatment.
- The presence of a mucopurulent discharge should not postpone cytology testing. The test can be performed after removal of the discharge with a saline-soaked cotton swab.
- HPV testing can be performed either as a separate test or by using material from the liquid-based cytology specimen.
- In the absence of other indications, the presence of external genital warts does not warrant more frequent cervical cancer screening.
- The sequence of cytology testing in relation to collection of other endocervical specimens does not influence Pap test results or their interpretation (*600*). Typically, vaginal specimens are preferred for chlamydia and gonorrhea screening; however, during a pelvic examination, endocervical specimens for STI testing can be collected first.
- Persons who have had a total hysterectomy with removal of the cervix do not require screening unless cervical intraepithelial neoplasia (CIN) 2, CIN 3, or adenocarcinoma in situ was diagnosed within the previous 20 years (*175*,*1247*). If the cervix remains intact after a supracervical hysterectomy, regularly scheduled Pap tests should be performed as indicated (*1248*–*1250*).
- Health care facilities that train providers on cytology test collection and use simple quality assurance measures are more likely to obtain satisfactory test results (as determined by the laboratory).
- The use of instruments designed to sample the cervical transformation zone (e.g., cytobrushes) improves the accuracy of cytology tests (*1251*).
- Both liquid-based and conventional cytology are acceptable because they have similar test-performance characteristics.
- At an initial visit, providers should ask patients about their recent cytology test and HPV results and any history of evaluation and treatment (e.g., loop electrosurgical excision procedure and colposcopy) to assist with management; effort should be made to obtain copies of recent results. The importance and frequency of screening should be reinforced.

#### Counseling

Persons might believe the cytology (Pap test) or HPV test screens for conditions other than cervical cancer, or they might be confused by abnormal results (*1252*–*1254*). Health care providers, as trusted sources of information about HPV infections and abnormal cytology test results, have an important role in educating persons about HPV and can moderate the psychosocial impact of abnormal results (*1255*,*1256*). Persons should be counseled on the risks, uncertainties, and benefits of screening (*174*,*1257*).

An abnormal cytology test or a positive HPV test can cause short-term anxiety, stress, fear, and confusion, possibly decreasing the patient’s ability to absorb and retain information and acting as a barrier to follow-up care (*1258*–*1261*). A positive HPV test might elicit concerns about partners, worries about disclosure, and feelings of guilt, anger, and stigmatization (*1260*). Providers should frame HPV positivity in a neutral, nonstigmatizing context and emphasize its common, asymptomatic, and transient nature. Providers also should emphasize that HPV infections often are shared between partners but it is often not possible to know the origin of an HPV infection; HPV tests might become positive many years after initial exposure due to reactivation of latent infections in both male and female partners. Having an HPV infection should not raise concerns about a male partner’s health (*1262*). Providers should communicate the meaning of both the cytology and HPV test results to patients at screening.

Providers also should screen for tobacco use and perform cessation counseling (www.acog.org/clinical/clinical-guidance/committee-opinion/articles/2011/09/tobacco-use-and-womens-health). Smoking contributes to the progression of CIN, with both active and passive smoking associated with squamous cell carcinoma of the cervix in women with HPV 16 or 18 infection (*1263*–*1266*).

#### Promoting Cervical Cancer Screening

Clinics can use the evidence-based interventions in the *Community Preventive Services Task Force* guidelines to promote cervical cancer screening in their communities (https://www.thecommunityguide.org/findings/cancer-screening-multicomponent-interventions-cervical-cancer). Implementing interventions that increase community demand for screening (*1266*) (e.g., client reminders, client incentives, media, group education, or one-on-one education) together with those that increase community access to screening (e.g., reducing structural barriers and reducing client out-of-pocket costs) is effective in increasing cervical cancer screening coverage. These interventions are more effective if they are implemented with interventions to increase provider delivery of screening services (e.g., provider assessment and feedback, provider incentives, and provider reminders). Print materials and online resources are available at https://www.cdc.gov/cancer/cervical/basic_info/screening.htm and https://www.cdc.gov/std/hpv/facts-brochures.htm. Patient navigators can be effective in improving both screening and follow-up after abnormal results (*1267*).

#### Key Messages About Cervical Cancer Screening

When counseling persons about cervical cancer screening, the provider should discuss the following:

- Cervical cancer can be prevented with regular screening tests, like the Pap test (cytology) and HPV tests. Those at average risk should start getting cytology tests at age 21 years.
- The cytology test can find abnormal cervical cells, which could lead to cervical cancer over time, and an HPV test detects HPV infection of the cervix. The HPV test can be used alone for cervical cancer screening or at the same time as the cytology test (known as cotesting) for those aged ≥30 years to 65 years. The HPV test is also used after a cytology test result of atypical squamous cells of undetermined significance (ASC-US) among persons aged *>*25 years (known as reflex HPV testing).
- Positive cytology and HPV tests are markers of cervical precancerous lesions, which often do not cause symptoms until they become invasive. Appropriate follow-up is essential to ensure that cervical cancer does not develop.
- HPV is a common infection and is often controlled by the body without any medical interventions. A positive HPV test does not mean that a person has cancer.
- Providers should emphasize that HPV infections often are shared between partners, and it is often not possible to know the origin of an HPV infection; HPV tests might become positive many years after initial exposure due to reactivation of latent infections in both male and female partners.

#### Management of Sex Partners

The benefit of disclosing a positive HPV test to current and future sex partners is unclear. The following counseling messages can be communicated to sex partners:

- Sex partners do not need to be tested for HPV.
- Sex partners tend to share HPV. Sex partners of persons with HPV infection also are likely have an HPV infection.
- Female sex partners of men who disclose they had a previous female partner with HPV should be screened at the same intervals as women with average risk. No data are available to suggest that more frequent screening is of benefit.
- When used correctly and consistently, condoms might lower the risk for HPV infection and might decrease the time to clear in those with HPV infection. However, HPV can infect areas not covered by the condom, and condoms might not fully protect against HPV (*24*,*25*).

Additional messages for partners include the messages for persons with HPV (see Cervical Cancer Screening; Counseling Messages).

#### Screening Recommendations in Special Populations

##### Pregnancy

Persons who are pregnant should be screened at the same intervals as those who are not. A swab, Ayre’s spatula, or cytobrush can be used for obtaining cytology test samples during pregnancy (*1268*–*1270*).

##### HIV Infection

Several studies have documented an increased risk for cervical precancers and cancers in individuals with HIV infection (*1271*–*1273*). Adolescents with HIV should be screened 1 year after onset of sexual activity but no later than age 21 years. Sexually active persons should be screened at the time of the initial HIV diagnosis. Conventional or liquid-based cytology (Pap test) should be used as primary HPV testing and is not recommended in individuals with HIV. Cotesting (cytology and HPV test) can be done in individuals aged ≥30 years with HIV. Annual screening is recommended for persons with HIV infection; after 3 years of consecutive normal cytology results or normal cotest (normal cytology and negative HPV test), the screening interval can be increased to every 3 years. Lifelong screening is recommended among persons with HIV infection.

Providers should defer to existing *Guidelines for the Prevention and Treatment of Opportunistic Infections in Adults and Adolescents with HIV* for guidance on cervical cancer screening and management of results in persons with HIV (*98*).

##### Adolescents

Prevalence of HPV infection is high among those aged <21 years (*174*); however, HPV infections and squamous intraepithelial lesions caused by HPV in adolescents are more likely to regress than those in older persons. For these reasons, cervical cancer screening and HPV testing are not recommended in immunocompetent adolescents. However, for adolescents with HIV infection, providers should screen 1 year after onset of sexual activity, regardless of age or mode of HIV acquisition (e.g., perinatally acquired or sexually acquired) (*98*); such screening is warranted because of the reported high rate of progression of abnormal cytology in adolescents with HIV.

#### Human Papillomavirus Tests for Cervical Cancer Screening

Clinical tests for HPV are used for the following: cervical cancer screening as a primary test, cervical cancer screening with a cytology test, triage of some abnormal cervical cytology results, follow-up after abnormal screening test results, follow-up after a colposcopy in which no CIN 2 or CIN 3 is found, and follow-up after treatment of cervical precancers. These tests are only FDA cleared for use with cervical specimens, not oral or anal specimens. Testing for nononcogenic HPV types (e.g., types 6 and 11) is not recommended (https://www.asccp.org/guidelines).

FDA-cleared HPV tests detect viral DNA or messenger RNA. Several FDA-cleared tests for HPV are available for use in the United States. The Cobas 4800 HPV test (Roche Molecular Diagnostics) and the Onclarity HPV test (Becton Dickinson) can detect the presence of 14 oncogenic HPV types (types 16, 18, 31, 33, 35, 39, 45, 51, 52, 56, 58, 59, 66, and 68), as well as individual types 16 and 18, and are cleared for primary cervical cancer screening.

Other HPV tests are cleared for use in conjunction with a cytology test or to triage some abnormal cervical cytology results; they should not be used for primary HPV testing because they are not cleared for this purpose. These tests include the Hybrid Capture 2 High-Risk HPV DNA test (Qiagen), the Cervista HPV High-Risk DNA and HPV 16/18 DNA tests (Hologics), and the APTIMA HR HPV (Gen Probe) test. All HPV assays should be FDA cleared and used only for the appropriate indications (https://www.fda.gov/media/122799/download) (*158*).

HPV testing should not be performed in the following situations:

- Deciding whether to vaccinate against HPV
- Conducting HPV tests for low-risk (nononcogenic) HPV types (e.g., types 6 and 11)
- Providing care to persons with genital warts or their partners
- Testing persons aged <25 years as part of routine cervical cancer screening
- Testing oral or anal specimens

Unlike cytology, samples for HPV testing have the potential to be collected by the patient and mailed to health programs for analysis, thus self-collection might be one strategy for increasing screening rates among populations where screening rates are low. Self-collection for HPV testing is not cleared by FDA or recommended by U.S. medical organizations (*174*).

#### Follow-Up of Abnormal Cytology and Human Papillomavirus Test Results

If the result of the cytology (Pap test) is abnormal, follow-up care should be provided according to the *2019* *ASCCP Risk-Based Management Consensus Guidelines for Abnormal Cervical Cancer Screening Tests and Cancer Precursors* (*158*)*.* Clinics that serve clients who might have difficulty adhering to follow-up recommendations and for whom linkage to care is unlikely should consider offering in-house colposcopy and biopsy services.

Consensus guidelines for management of abnormal cervical cancer screening tests combine patient-level risk data with clinical action thresholds to generate personalized management recommendations (Table 2). This framework allows management on the basis of risk for CIN 3, not specific test results. The guidelines were designed to identify persons at high risk who require colposcopy or expedited treatment and persons at low risk who might be able to safely defer invasive diagnostic procedures. The risk-based framework was designed to easily incorporate future revisions, such as the inclusion of new technologies for screening and management. Use of the guidelines can be facilitated by electronic technology that is continually updated, such as a smartphone application or the website (https://www.asccp.org/Default.aspx). 

**TABLE 2:** Comparison of 2012 and 2019 consensus recommendations for management of common abnormalities — American Society for Colposcopy and Cervical Pathology

Current HPV result	Current Pap test result	Previous result	Management by 2012 guidelines	Management by 2019 guidelines
Negative	ASC-US	Unknown or HPV negative*	Repeat Pap plus HPV testing in 3 yrs	Repeat HPV test with or without concurrent Pap test in 3 yrs
Negative	LSIL	Unknown or HPV negative*	Repeat Pap plus HPV testing in 1 yr preferred, colposcopy acceptable	Repeat HPV test with or without concurrent Pap test in 1 yr
Negative	ASC-H	Noncontributory	Colposcopy	Colposcopy
Noncontributory	AGC	Noncontributory	Colposcopy	Colposcopy
Positive	NILM	Unknown or HPV negative*	Repeat Pap plus HPV testing in 1 yr	Repeat HPV test with or without concurrent Pap test in 1 yr
Positive	NILM	HPV positive^†^	Colposcopy	Colposcopy
Positive for genotype HPV 16, HPV 18, or both	NILM	Noncontributory	Colposcopy	Colposcopy
Positive for genotype HPV 16, HPV 18, or both	ASC-US or LSIL	Noncontributory	Not applicable, genotyping not recommended for ASC-US or LSIL in 2012	Colposcopy
Positive	ASC-US or LSIL	Unknown or HPV positive	Colposcopy	Colposcopy
Positive	ASC-US or LSIL	Negative screening results with HPV testing or HPV plus Pap testing within the previous 5 yrs	Colposcopy	Repeat HPV test with or without concurrent Pap test in 1 yr^§^
Positive	ASC-US or LSIL	Colposcopy confirming the absence of high-grade lesion within the past yr	Colposcopy	Repeat HPV test with or without concurrent Pap test in 1 yr^§^
Positive	ASC-H	Noncontributory	Colposcopy	Colposcopy or expedited treatment
Positive untyped, positive for genotype other than HPV 16, or negative	HSIL	Noncontributory	Colposcopy or expedited treatment	Colposcopy or expedited treatment
Positive for genotype HPV 16	HSIL	Noncontributory	Colposcopy or expedited treatment	Expedited treatment^¶^

**Sources: **Massad LS, Einstein MH, Huh WK, et al.; 2012 ASCCP Consensus Guidelines Conference. 2012 updated consensus guidelines for the management of abnormal cervical cancer screening tests and cancer precursors. Obstet Gynecol 2013;121:829–46; Perkins RB, Guido RS, Castle PE, et al; 2019 ASCCP Risk-Based Management Consensus Guidelines Committee. 2019 ASCCP risk-based management consensus guidelines for abnormal cervical cancer screening tests and cancer precursors. J Low Genit Tract Dis 2020;24:102–31; Perkins R, Guido R, Saraiya M, et al. Summary of current guidelines for cervical cancer screening and management of abnormal test results: 2016–2020. J Womens Health (Larchmt) 2021;30:5–13.

**Abbreviations:** AGC = atypical glandular cells; AIS = adenocarcinoma in situ; ASC-H = atypical squamous cells cannot exclude high-grade squamous intraepithelial lesion; ASC-US = atypical squamous cells of undetermined significance; CIN = cervical intraepithelial neoplasia; HPV = human papillomavirus; HSIL = high-grade squamous intraepithelial lesion; LSIL = low-grade squamous intraepithelial lesion; NILM = negative for intraepithelial lesion or malignancy; Pap = Papanicolaou.

* Colposcopy may be warranted for patients with a history of high-grade lesions (CIN 2 or CIN 3, histologic or cytologic HSIL, ASC-H, AGC, or AIS).

^†^ Previous Pap test results do not modify the recommendation; colposcopy is always recommended for two consecutive HPV-positive tests

^§ ^Negative HPV test or cotest (HPV plus Pap test) results only reduce risk sufficiently to defer colposcopy if performed for screening purposes within the last 5 years. Colposcopy is still warranted if negative HPV test or cotest results occurred in the context of surveillance for a previous abnormal result.

^¶ ^Expedited treatment is preferred for nonpregnant patients aged ≥25 years. Colposcopy with biopsy is an acceptable option if desired by patient after shared decision-making.

The following are highlights of the new management guidelines:

- Colposcopy can be deferred for patients at low risk.
  º If a patient has a minimally abnormal test result (i.e., negative for intraepithelial lesion or malignancy HPV positive, ASC-US HPV positive, LSIL, or HPV positive) that was preceded by a negative screening HPV test or cotest within the past 5 years, follow-up in 1 year instead of colposcopy is recommended (a negative HPV test or cotest performed during follow-up of abnormal results would not similarly reduce risk).
  º Referral to colposcopy is recommended if cytology test results are abnormal or the HPV test is positive at the 1-year follow-up visit.
- Treatment can be expedited for high-risk patients.
  º If a patient has a high-grade cytology (Pap test) result (i.e., HSIL) and an HPV test that is positive for HPV type 16, then treatment with a loop electrosurgical excision procedure (LEEP) is preferred. A colposcopy with biopsy is not necessary to confirm the diagnosis first.
  º If a patient who has not been screened in more than 5 years (i.e., rarely screened) has an HSIL cytology result and a positive HPV test (regardless of type), then treatment with LEEP is preferred. A colposcopy with biopsy is not necessary to confirm the diagnosis first.
  º When considering treatment without confirmatory biopsy, shared decision-making with the patient is important. Considerations include age, concern about cancer, ability to follow up, financial concerns, and concerns about the potential effect of treatment on a future pregnancy.
- When primary HPV testing is used for screening, cytology testing should be performed for all positive HPV test results to help determine the next steps in management.
  º Ideally, cytology testing should be performed by the laboratory as a reflex test from the same specimen so the patient does not need to return to the clinic. Colposcopy is recommended if HPV genotyping is positive for types 16 or 18, and it can be considered if it is infeasible for the patient to return for cytology alone (*1274*).
  º HPV 16 is the highest-risk HPV type. Expedited treatment should be considered for HSIL cytology results, and colposcopy is recommended in all other cases, even if the cytology test is normal.
  º HPV 18 has a relatively high association with cancer, and colposcopy is recommended in all cases, even if the cytology test is normal. Because of the association of HPV 18 with adenocarcinoma, endocervical sampling is acceptable at the time of colposcopy.
  º If the HPV type is not HPV 16 or 18, and the cytology test is normal, return in 1 year is recommended in most cases.
- HPV testing or cotesting is preferred to cytology testing alone for follow-up after an abnormal test result.
  º Negative HPV testing or cotesting is less likely to miss disease than normal cytology testing alone. Therefore, cytology testing is recommended more often than HPV testing or cotesting for follow-up of abnormal results. Specifically, cytology testing is recommended annually when HPV testing or cotesting is recommended at 3-year intervals, and cytology testing is recommended at 6-month intervals when HPV testing or cotesting is recommended annually.
- After treatment for a high-grade precancer (moderate or severe dysplasia), surveillance should continue for at least 25 years.
  º Initial testing includes an HPV test or cotest at 6, 18, and 30 months. If cytology alone is used, testing should occur at 6, 12, 18, 24, and 30 months.
  º After completing initial testing, long-term surveillance includes testing at 3-year intervals if using HPV testing or cotesting, or annual testing if using cytology testing alone.
  º Surveillance should continue for at least 25 years after the initial treatment, even if this extends beyond age 65 years. If a woman undergoes hysterectomy during the surveillance period, vaginal screening should continue.

### Anal Cancer

Anal cancer is rare in the general population (1–2 cases per 100,000 person-years); however, incidence is substantially higher among specific populations, including MSM with HIV infection (80–131 cases per 100,000 person-years), men with HIV infection (40–60 cases per 100,000 person-years), women with HIV infection (20–30 cases per 100,000 person-years), and MSM without HIV infection (14 cases per 100,000 person-years) (*1275*–*1279*). Incidence is variable among women with previous HPV-related gynecologic dysplasia and cancer (6–63 cases per 100,000 person-years) (*1280*,*1281*). Persistent HPV infection might be a risk factor for preventable HPV-associated second primary cancers among survivors of HPV-associated cancers (*1282*).

Data are insufficient to recommend routine anal cancer screening with anal cytology in persons with HIV infection, MSM without HIV infection, and the general population. An annual digital anorectal examination (DARE) might be useful to detect masses on palpation in persons with HIV infection and possibly in MSM without HIV with a history of receptive anal intercourse (*98*). More evidence is needed concerning the natural history of anal intraepithelial neoplasia, the best screening methods and target populations, the safety and response to treatments, and other programmatic considerations before screening can be routinely recommended.

#### Populations at High Risk and Digital Anorectal Examination

Providers should discuss anal cancer risk with their patients among specific populations to guide management. According to the HIV Opportunistic Infection guidelines and the International Anal Neoplasia Society, a DARE should be performed to detect early anal cancer in persons with HIV infection and MSM without HIV with a history of receptive anal intercourse (*98*,*1283*). DARE is acceptable to patients and has a low risk for adverse outcomes (*1284*,*1285*).

Data are insufficient to guide initiation of DARE at a defined age or optimal intervals for examination. Whereas anal HSIL is observed among young adults, cancer incidence begins to increase after the early 30s and continues to increase as a function of age.

#### Populations at High Risk and Anal Cytology

Data are insufficient to recommend routine anal cancer screening with anal cytology among populations at risk for anal cancer. Certain clinical centers perform anal cytology to screen for anal cancer among populations at high risk (e.g., persons with HIV infection, MSM, and those having receptive anal intercourse), followed by high-resolution anoscopy (HRA) for those with abnormal cytologic results (e.g., ACS-US, LSIL, or HSIL). Sensitivity and specificity of anal cytology to detect HSIL are limited (sensitivity 55%–89% and specificity 40%–67%) (*1286*–*1291*). Health centers that initiate a cytology-based screening program should only do so if referrals to HRA and biopsy are available.

HRA can be used for diagnosis of HSIL, to monitor response to therapy, or to conduct surveillance of HSIL for evidence of progression. HRA is the primary method used for diagnosis of superficially invasive squamous carcinoma, a very early form of anal cancer that is not palpable on DARE. However, data are insufficient to conclude whether use of HRA leads to reductions in anal cancer incidence or improves anal cancer morbidity and mortality. An ongoing clinical trial is investigating whether treatment of HSIL is effective in reducing the incidence of anal cancer among persons with HIV infection (NCT02135419).

#### Human Papillomavirus Testing

HPV tests (using high-risk HPV types) are not clinically useful for anal cancer screening because of a high prevalence of anal HPV infection among populations at high risk, particularly MSM (*1278*,*1289*,*1290*). No standard HPV-based algorithms exist for anal cancer screening, due to the high prevalence of high-risk HPV infection among groups at risk.

#### Treatment of Anal High-Grade Squamous Intraepithelial Lesion

Multiple office-based treatments exist for anal HSIL, including ablative methods (e.g., laser, electrocautery, or infrared coagulation) and topical patient-applied therapies (e.g., imiquimod). Recurrence rates with both provider-applied and patient-applied treatments are high, ranging from approximately 50% at 1 year to 77% after 3 years (*1289*,*1292*,*1293*). In addition, evidence exists that HSIL might spontaneously regress without treatment (*1294*,*1295*). Shared decision-making about treatment for anal HSIL is recommended because of limited data on the natural history of anal HSIL, including factors related to progression or regression of lesions.

## Viral Hepatitis

### Hepatitis A Virus Infection

HAV infection has an incubation period of approximately 28 days (range: 15–50 days) (*1296*). HAV replicates in the liver and is shed in high concentrations in feces from 2–3 weeks before to 1 week after the onset of clinical illness. HAV infection produces a self-limited disease that does not result in chronic infection or chronic liver disease. However, approximately 10% of patients experience a relapse of symptoms during the 6 months after acute illness. Acute liver failure from hepatitis A is rare (overall case-fatality rate: 0.5%). The risk for symptomatic infection is directly related to age, with approximately 70% of adults having symptoms compatible with acute viral hepatitis and the majority of children having either asymptomatic or unrecognized infection. Antibody produced in response to HAV infection persists for life and confers protection against reinfection (*1297*).

HAV infection is primarily transmitted by the fecal-oral route, by either person-to-person contact or through consumption of contaminated food or water (*1298*). Transmission of HAV during sexual activity probably results from fecal-oral contact. Although viremia occurs early during infection and can persist for weeks after symptom onset, bloodborne transmission of HAV is uncommon (*1299*). Transmission by saliva has not been demonstrated.

In the United States, of the hepatitis A cases accompanied by risk information, a particular risk was identified among only 23.8% (*13*,*372*). Among cases with a risk factor identified, a recognized foodborne or waterborne outbreak was the most commonly identified risk (49.6%). Other infection sources identified in the United States include MSM; persons who use injecting drugs; sexual and household contacts; those experiencing homelessness; international travelers; those with children attending a nursery, childcare, or preschool; and persons working in such settings (*13*,*372*).

#### Diagnostic Considerations

Diagnosis of HAV infection cannot be made on a clinical basis alone but requires serologic testing. Presence of IgM antibody to HAV is diagnostic of acute HAV infection. A positive test for total anti-HAV indicates immunity to HAV infection but does not differentiate current from previous HAV infection. Although usually not sensitive enough to detect the low level of protective antibody after vaccination, anti-HAV tests also might be positive after hepatitis A vaccination.

#### Treatment

Patients with acute HAV infection usually require only supportive care, with no restrictions in diet or activity. Hospitalization might be necessary for patients who become dehydrated because of nausea and vomiting and is crucial for patients with signs or symptoms of acute liver failure. Medications that might cause liver damage or are metabolized by the liver should be used with caution among persons with HAV infection.

#### Prevention

Vaccination is the most effective means of preventing HAV transmission among persons at risk for infection (e.g., MSM, injecting drug users, and persons with chronic liver disease) who did not receive hepatitis A vaccination during childhood. Hepatitis A vaccines are prepared from formalin-inactivated, cell-culture–derived HAV. Two monovalent vaccines (Havrix and Vaqta are approved by FDA for persons aged ≥12 months (Table 3). These vaccines are available for eligible children and adolescents aged <19 years through the VFC program (https://www.cdc.gov/vaccines/programs/vfc/index.html). Administered IM in a 2-dose series at 0 and 6–12 months, hepatitis A vaccines induce protective antibody levels among virtually all adults. By 1 month after the first dose, 94%–100% of adults have protective antibody levels, and after a second dose, 100% achieve protective levels (*1297*,*1300*,*1301*). Kinetic models of antibody decrease among adults indicate that protective levels persist for >40 years (*1302*–*1304*). A study of Alaska Natives demonstrated that seropositivity for hepatitis A persists for >20 years after completing 2-dose vaccination at age 12–21 months (*1302*). Anti-HAV persistence of >20 years was demonstrated among immunocompetent adults vaccinated with a 2-dose hepatitis A schedule as adults (*1303*,*1305*). A combined hepatitis A and hepatitis B vaccine (Twinrix) has been developed and licensed for use as a 3-dose series for adults aged ≥18 years at risk for HAV or HBV infections. When administered IM on a 0-, 1-, and 6-month schedule, the vaccine has equivalent immunogenicity to that of the monovalent hepatitis A vaccines.

**TABLE 3:** Vaccines for preventing hepatitis A infection

Vaccine	Trade name (manufacturer)	Age group (yrs)	Dose	Route	Schedule	Booster
Hep A inactivated (2 doses)	Havrix (GlaxoSmithKline)	1–18	0.5 mL (720 ELISA units inactivated HAV)	IM	0, 6–12 mos	None
		≥19	1 mL (1,440 ELISA units inactivated HAV	IM	0, 6–12 mos	None
Hep A inactivated (2 doses)	Vaqta (Merck)	1–18	0.5 mL (25 units HAV antigen)	IM	0, 6–18 mos	None
		≥19	1 mL (50 units HAV antigen)	IM	0, 6–18 mos	None
Combined Hep A and Hep B* (3 doses)	Twinrix (GlaxoSmithKline)	≥18 (primary)	1 mL (720 ELISA units inactivated plus 20 *µ*g HBsAg	IM	0, 1, 6 mos	None
		≥18 (accelerated)	1 mL (720 ELISA units inactivated plus 20 *µ*g HBsAg	IM	0, 7, 21–30 days	12 mos

**Source: **Nelson NP, Weng MK, Hofmeister MG, et al. Prevention of hepatitis A virus infection in the United States: recommendations of the Advisory Committee on Immunization Practices, 2020. MMWR Recomm Rep 2020;69(No. RR-5).

**Abbreviations:** ELISA = enzyme-linked immunosorbent assay; HAV = hepatitis A virus; HBsAg = hepatitis B surface antigen; Hep A = hepatitis A; Hep B = hepatitis B; IM= intramuscular.

* Combined Hep A and Hep B vaccine (Twinrix) should not be used as postexposure prophylaxis.

##### Pre-Exposure Vaccination

Persons at risk for HAV infection (Box 5) (*1297*) should be offered vaccine (Table 3). If persons are at risk for both HAV and HBV, the combined vaccine can be considered.

BOX 5 **Populations recommended for hepatitis A vaccination — Advisory Committee on Immunization Practices, 2020**

**Children**

- All children aged 12–23 months
- Unvaccinated children and adolescents aged 2–18 years

**Persons at increased risk for hepatitis A virus (HAV) infection**

- International travelers
- Men who have sex with men
- Persons who use injecting or noninjecting drugs (i.e., all those who use illegal drugs)
- Persons with occupational risk for exposure
- Persons who anticipate close personal contact with an international adoptee
- Persons experiencing homelessness

**Persons at increased risk for severe disease from HAV infection**

- Persons with chronic liver disease
- Persons with HIV infection

**Other persons recommended for vaccination**

- Pregnant women at risk for HAV infection or severe outcome from HAV infection
- Any persons who requests a vaccine

**Vaccination during outbreaks**

- Unvaccinated persons in outbreak settings who are at risk for HAV infection or at risk for severe disease from HAV

**Implementation strategies for settings providing services to adults**

- Persons in settings that provide services to adults where a high proportion of those persons have risk factors for HAV infection

**Hepatitis A vaccination is no longer recommended by the Advisory Committee on Immunization Practices**

- Persons who receive blood products for clotting disorders (e.g., hemophilia)

**Source**: Nelson NP, Weng MK, Hofmeister MG, et al. Prevention of hepatitis A virus infection in the United States: recommendations of the Advisory Committee on Immunization Practices, 2020. MMWR Recomm Rep 2020;69(No. RR-5).

##### Prevaccination Serologic Testing

Among U.S.-born adults aged >20 years, HAV susceptibility prevalence (i.e., total antibody to HAV was negative) was 74.1% (95% CI: 72.9%–75.3%) during 2007–2016 (*1306*).Prevaccination serologic testing for HAV immunity before vaccination is not routinely recommended; however, it can be considered in specific settings to reduce costs by not vaccinating persons who are already immune. Prevaccination serologic testing should not be a barrier to vaccination of susceptible persons, especially for populations that are difficult to access. If prevaccination testing is performed, commercially available tests for total anti-HAV or IgG anti-HAV should be used (*1297*).

Persons for whom prevaccination testing will likely be most cost-effective include adults who were either born in or lived for extensive periods in geographic areas where HAV endemicity is high or intermediate (*1297*). Prevaccination serologic testing of children is not indicated because of the low prevalence of infection among that age group.

For populations who are expected to have high rates of previous HAV infection, vaccination history should be obtained when feasible before testing or vaccination. Vaccination should not be postponed if vaccination history cannot be obtained, records are unavailable, or prevaccination testing is infeasible. Vaccinating persons immune from natural infection carries no known risk, nor does giving extra doses of hepatitis A vaccine (*1307*). Vaccination of a person who is already immune is not harmful. Persons who have a documented history of ≥2 doses of hepatitis A vaccine do not need further vaccination or serologic testing.

##### Postvaccination Serologic Testing

Serologic testing for immunity is unnecessary after routine vaccination of infants, children, or adults (*1297*). Testing for anti-HAV antibody after vaccination is recommended for persons whose subsequent clinical management depends on knowledge of their immune status and persons for whom revaccination might be indicated (e.g., persons with HIV infection and other immunocompromising conditions).

##### Postexposure Prophylaxis

Persons who recently have been exposed to HAV and who previously have not received hepatitis A vaccine should be administered a single dose of monovalent hepatitis A vaccine or immunoglobulin (IG) (0.1 mL/kg body weight) as soon as possible, ideally <2 weeks after exposure because the efficacy of vaccine or IG when administered >2 weeks after exposure has not been established (*1297*). In most cases, monovalent hepatitis A vaccine at the age-appropriate dose is preferred over IG for PEP. Advantages of hepatitis A vaccine for PEP include induction of active immunity, longer-term protection, ease of administration, and better acceptability and availability. Decisions to use vaccine versus IG should be guided by patient characteristics associated with more severe manifestations of HAV infection (e.g., older age, immunocompromising conditions, and chronic liver disease) and the magnitude of the risk for HAV transmission resulting from the exposure (*1297*).

IG should be used for children aged <6 months, immunocompromised persons, persons with chronic liver disease, and persons for whom vaccine is contraindicated. IG can be administered to persons aged >40 years, in addition to hepatitis A vaccine (*1297*).

IG administered IM can provide PEP against HAV (Table 4). IG is a sterile solution of concentrated immunoglobulins prepared from pooled human plasma processed by cold ethanol fractionation. In the United States, IG is produced only from plasma that has tested negative for HBsAg, antibodies to HIV and HCV, and HIV and HCV RNA. In addition, the process used to manufacture IG inactivates viruses (e.g., HBV, HCV, and HIV). When administered IM <2 weeks after exposure to HAV, IG is >85% effective in preventing HAV infection (*1308*).

**TABLE 4:** Recommendations for hepatitis A postexposure prophylaxis and pre-exposure protection, by age group and risk category — Advisory Committee on Immunization Practices, 2020

Indication and age group	Risk category and health status	Hepatitis A vaccine	IG*
**Postexposure prophylaxis**			
0–11 mos	Healthy	No	0.1 mL/kg body weight
12 mos to 40 yrs	Healthy	1 dose^†^	None
>40 yrs	Healthy	1 dose^†^	0.1 mL/kg body weight^§^
≥12 mos	Immunocompromised or chronic liver disease	1 dose^†^	0.1 mL/kg body weight^¶^
≥12 mos	Vaccine contraindicated**	No	0.1 mL/kg body weight
**Pre-exposure protection (e.g., travel)^††^**			
<6 mos	Healthy	No	0.1–0.2 mL/kg body weight^§§^
6–11 mos	Healthy	1 dose^¶¶^	None
12 mos to 40 yrs	Healthy	1 dose***	None
>40 yrs	Healthy	1 dose***	0.1–0.2 mL/kg body weight^§§,†††^
>6 mos	Immunocompromised or chronic liver disease	1 dose***	0.1–0.2 mL/kg body weight^§§,†††^
>6 mos	Persons who elect not to receive vaccine or for whom vaccine is contraindicated**	No	0.1–0.2 mL/kg body weight^§§^

**Source:** Nelson NP, Weng MK, Hofmeister MG, et al. Prevention of hepatitis A virus infection in the United States: recommendations of the Advisory Committee on Immunization Practices, 2020. MMWR Recomm Rep 2020;69(No. RR-5).

**Abbreviations:** HAV = hepatitis A virus; IG = immune globulin.

* Measles, mumps, and rubella vaccine should not be administered for ≥2 weeks before and 6 months after administration of IG.

^† ^A second dose of hepatitis A vaccine is not required for postexposure prophylaxis; however, for long-term immunity, the vaccination series should be completed with a second dose ≥6 months after the first dose.

^§ ^The provider’s risk assessment should determine the need for IG administration. If the provider’s risk assessment determines that both vaccine and IG are warranted, hepatitis A vaccine and IG should be administered simultaneously at different anatomic sites (e.g., separate limbs).

^¶ ^Vaccine and IG should be administered simultaneously at different anatomic sites (e.g., separate limbs).

** Life-threatening allergic reaction to a previous dose of hepatitis A vaccine or allergy to any vaccine component.

^†† ^IG should be considered before travel for persons with special risk factors for either HAV infection or severe disease from HAV infection.

^§§ ^0.1 mL/kg body weight for travel ≤1 month; 0.2 mL/kg body weight for travel ≤2 months; 0.2 mL/kg every 2 months for travel of ≥2 months’ duration.

^¶¶ ^This dose should not be counted toward the routine 2-dose series, which should be initiated at age 12 months.

*** For persons not previously vaccinated with hepatitis A vaccine, administer dose as soon as travel is considered and complete the series according to routine schedule if the next dose is needed before travel.

^††† ^Can be administered on the basis of the provider’s risk assessment.

If IG is administered to persons for whom hepatitis A vaccine also is recommended, a dose of vaccine should be provided simultaneously with IG in different anatomic sites (e.g., different limbs) as soon as possible, and the second vaccine dose should be administered according to the licensed schedule to complete the series. The combined vaccine can be considered for persons among whom both hepatitis A and hepatitis B vaccine is recommended (*13*,*1297*,*1302*–*1304*).

#### Special Considerations

For persons with HIV infection, antibody response can be directly related to CD4^+^ T-cell levels. Although persons with HIV who have lower CD4^+^ T-cell counts or percentages might have a weaker response to the vaccine, vaccination should not be delayed for the CD4^+^ T-cell count to exceed a certain threshold because of the prolonged risk for HAV exposure created by missed opportunities to vaccinate.

### Hepatitis B Virus Infection

The incubation period for HBV infection from time of exposure to symptom onset ranges from 6 weeks to 6 months. The highest concentrations of HBV are located in blood, with lower concentrations in other body fluids including wound exudates, semen, vaginal secretions, and saliva (*1309*,*1310*). HBV is more infectious and more stable in the environment than other bloodborne pathogens (e.g., HCV or HIV).

HBV infection can be either self-limited or chronic. Among adults, approximately half of newly acquired HBV infections are symptomatic, and approximately 1% of reported cases result in acute liver failure and death (*1311*). Risk for chronic infection is inversely related to age at acquisition; approximately 90% of infected infants and 30% of infected children aged <5 years become chronically infected, compared with 2%–6% of persons who become infected as adults (*1312*). Among persons with chronic HBV infection, the risk for premature death from cirrhosis or hepatocellular carcinoma is 15%–25% (*1313*).

HBV is efficiently transmitted by percutaneous or mucous membrane exposure to HBV-infected blood or body fluids that contain HBV. The primary risk factors associated with infection among adolescents and adults are unprotected sex with an infected partner, having multiple partners, men having sex with men, having history of other STIs, and injecting drug use (*233*). In addition, studies have demonstrated other modes of HBV transmission, including premastication and lapses in health care infection control procedures, as less common sources of transmission (*1314*–*1317*).

CDC’s national strategy for eliminating transmission of HBV infection includes prevention of perinatal infection through routine screening of all pregnant women for HBsAg and immunoprophylaxis of infants born to mothers with HBsAg or mothers whose HBsAg status is unknown, routine infant vaccination, vaccination of previously unvaccinated children and adolescents through age 18 years, and vaccination of previously unvaccinated adults at increased risk for infection (*12*). High vaccination coverage rates with subsequent decreases in acute HBV infection incidence have been achieved among infants and adolescents (*1318*). The vaccination of persons as children and adolescents likely has led to improved vaccination coverage among adults aged <30 years (*1319*) and corresponding lower rates of acute HBV infection among this group. In contrast, vaccination coverage among the majority of adult populations at high risk aged ≥30 years (e.g., persons with multiple sex partners, MSM, and injecting drug users) has remained low (*1320*,*1321*); these groups account for the highest rates of preventable acute infections (*12*,*1319*,*1322*). STD clinics and other health care settings providing STI services to adults at high risk for infection should administer hepatitis B vaccine to those who are unvaccinated.

#### Diagnosis

Diagnosis of acute or chronic HBV infection requires serologic testing (Table 5). Because HBsAg is present in both acute and chronic infection, presence of IgM antibody to hepatitis B core antigen (IgM anti-HBc) is diagnostic of acute or recently acquired HBV infection. Antibody to HBsAg (anti-HBs) is produced after a resolved infection and is the only HBV antibody marker present after vaccination. The presence of HBsAg and anti-HBc, with a negative test for IgM anti-HBc, indicates chronic HBV infection. The presence of total anti-HBc alone might indicate acute, resolved, or chronic infection or a false-positive result.

**TABLE 5:** Interpretation of serologic test results* for hepatitis B virus infection

Serologic marker				Interpretation
HBsAg	Total anti-HBc	IgM anti-HBc	Anti-HBs	
-	-	-	-	Never infected
+^†^	-	-	-	Early acute infection; transient (≤18 days) after vaccination
+	+	+	-	Acute infection
-	+	+	-	Acute resolving infection
-	+	-	+	Recovered from past infection and immune
+	+	-	-	Chronic infection
-	+	-	-	Past infection; low-level chronic infection^§^; passive transfer to infant born to HBsAg-positive mother; false positive (no infection)
-	-	-	+	Immune if concentration is >10 mIU/mL after vaccination, passive transfer after HBIG administration

**Source: **Adapted from Schillie S, Vellozzi C, Reingold A, et al. Prevention of hepatitis B virus infection in the United States: recommendations of the Advisory Committee on Immunization Practices. MMWR Recomm Rep 2018;67(No. RR-1).

**Abbreviations:** anti-HBc = antibody to hepatitis B core antigen; anti-HBs = antibody to hepatitis B surface antigen; HBIG = hepatitis B immune globulin; HBsAg = hepatitis B surface antigen; IgM = immunoglobulin M.

* - = negative test result; + = positive test result.

^† ^To ensure that an HBsAg-positive test result is not false positive, samples with repeatedly reactive HBsAg results should be tested with a neutralizing confirmatory test cleared by the Food and Drug Administration.

^§ ^Persons positive for only anti-HBc are unlikely to be infectious, except under unusual circumstances involving direct percutaneous exposure to large quantities of blood (e.g., blood transfusion or organ transplantation) or mutant HBsAg-related infection.

#### Treatment

No specific therapy is available for persons with acute HBV infection; treatment is supportive. Persons with chronic HBV infection should be referred for evaluation to a provider experienced in managing such infections. Therapeutic agents approved by FDA for treatment of chronic HBV infection can achieve sustained suppression of HBV replication and remission of liver disease (*1323*).

#### Prevention

Two products have been approved for HBV prevention: hepatitis B immune globulin (HBIG) for PEP and hepatitis B vaccine (*12*). HBIG provides temporary (i.e., 3–6 months) protection from HBV infection and is typically used as PEP as an adjunct to hepatitis B vaccination for previously unvaccinated persons or for persons who have not responded to vaccination. HBIG is prepared from plasma known to contain high concentrations of anti-HBs. The recommended dose of HBIG is 0.06 mL/kg body weight.

Hepatitis B vaccine contains HBsAg produced in yeast by recombinant DNA technology and provides protection from HBV infection when used for both pre-exposure vaccination and PEP. The three available monovalent hepatitis B vaccines for use in the United States are Recombivax HB, Engerix-B, and Heplisav-B. A combination hepatitis A and hepatitis B vaccine for use among persons aged ≥18 years, Twinrix, also is available.

When selecting a hepatitis B vaccination schedule, health care providers should consider the need to achieve completion of the vaccine series. The recommended HBV dose and schedule varies by product and age of recipient (Table 6). Three different 3-dose schedules for adolescents and adults have been approved for both monovalent hepatitis B vaccines (i.e., Engerix-B and Recombivax HB); these vaccines can be administered at 0, 1, and 6 months; 0, 1, and 4 months; or 0, 2, and 4 months. A 4-dose schedule of Engerix-B at 0, 1, 2, and 12 months is licensed for all age groups. A 2-dose schedule of Recombivax HB adult formulation (10 *µ*g) is licensed for adolescents aged 11–15 years, with a 4-month minimal interval between doses. When scheduled to receive the second dose, adolescents aged 16–19 years should be switched to a 3-dose series, with doses 2 and 3 consisting of the pediatric formulation (5 *µ*g) administered on a recommended schedule. Heplisav-B is a new single-antigen recombinant hepatitis B vaccine with a novel cytosine-phosphate-guanine 1018 oligodeoxynucleotide adjuvant for prevention of HBV infection among persons aged ≥18 years, administered as a 2-dose series at 0 and 1 month (>4 weeks apart) (*156*). Twinrix is a 3-dose schedule administered at 0, 1, and 6 months to persons aged ≥18 years at risk for both HAV and HBV infections.

**TABLE 6:** Recommended doses of licensed formulations of hepatitis B vaccines

Age group (yrs)	Single-antigen vaccine						Combination vaccine	
	Recombivax HB		Engerix-B		Heplisav-B*		Twinrix^†^	
	Dose (*μ*g)^§^	Volume (mL)	Dose (*μ*g)^§^	Volume (mL)	Dose (*μ*g)^§^	Volume (mL)	Dose (*μ*g)^§^	Volume (mL)
Infants (<1)	5	0.5	10	0.5	—^¶^	—^¶^	NA	NA
Children (1–10)	5	0.5	10	0.5	—^¶^	—^¶^	NA	NA
Adolescents (11–15)	10**	1.0	NA	NA	—^¶^	—^¶^	NA	NA
Adolescents (11–19)	5	0.5	10	0.5	—^¶^	—^¶^	NA	NA
Adults (≥18)	—^††^	—^††^	—^††^	—^††^	20*	0.5	20^†^	1
Adults (≥20)	10	1	20	1	20^†^	0.5	20^†^	1
Hemodialysis patients and other immunocompromised persons (<20^§§^)	5	0.5	10	0.5	20	0.5	NA	NA
Hemodialysis patients and other immunocompromised persons (≥20)	40^¶¶^	1	40***	2.0	20	0.5	NA	NA

**Source: **Adapted from Schillie S, Vellozzi C, Reingold A, et al. Prevention of hepatitis B virus infection in the United States: recommendations of the Advisory Committee on Immunization Practices. MMWR Recomm Rep 2018;67(No. RR-1).

**Abbreviation:** NA = not applicable.

* Administered on a 2-dose schedule.

^† ^Combined hepatitis A and B vaccines. This vaccine is recommended for persons aged ≥18 years who are at increased risk for both hepatitis B and hepatitis A virus infections.

^§ ^Recombinant hepatitis B surface antigen protein dose.

^¶^ Heplisav-B should not be used for vaccination of infants, children, or adolescents because the safety and effectiveness of Heplisav-B has not been established in persons aged <8 years and is not approved for use in these populations.

** Adult formulation administered on a 2-dose schedule.

^††^ Engerix-B and Recombivax HB are approved for use in persons of all ages.

^§§ ^Higher doses might be more immunogenic; however, no specific recommendations have been made.

^¶¶ ^Dialysis formulation administered on a 3-dose schedule at 0, 1, and 6 months.

*** Two 1.0-mL doses administered at one site, on a 4-dose schedule at 0, 1, 2, and 6 months.

Hepatitis B vaccine should be administered IM in the deltoid muscle and can be administered simultaneously with other vaccines. If the vaccine series is interrupted after the first or second dose of vaccine, the missed dose should be administered as soon as possible. The series does not need to be restarted after a missed dose. HBV vaccination is available for eligible children and adolescents aged <19 years through the VFC program (https://www.cdc.gov/vaccines/programs/vfc/contacts-state.html). When feasible, the same manufacturer’s vaccines should be used to complete the series; however, vaccination should not be deferred when the manufacturer of the previously administered vaccine is unknown or when the vaccine from the same manufacturer is unavailable (*1324*).

Among adolescents and healthy adults aged <40 years, approximately 30%–55% achieve a protective antibody response (i.e., anti-HBs ≥10 mIU/mL) after the first single-antigen vaccine dose, 75% after the second, and >90% after the third. Recent clinical trials reported a protective antibody response achieved among approximately 90% of participants receiving Heplisav-B, compared with 70.5%–90.2% of participants receiving Engerix-B (*12*). Vaccine-induced immune memory has been demonstrated to persist for >30 years (*1325*–*1327*). Periodic testing to determine antibody levels after routine vaccination among immunocompetent persons is unnecessary, and booster doses of vaccine are not recommended.

Hepatitis B vaccination is usually well tolerated by the majority of recipients. Pain at the injection site and low-grade fever are reported by a minority of recipients. For children and adolescents, a causal association exists between receipt of hepatitis B vaccination and anaphylaxis. For each 1.1 million doses of vaccine administered, approximately one recipient will experience this type of reaction (*1328*); however, no deaths have been reported among these patients (*1318*,*1328*). Vaccine is contraindicated for persons with a history of anaphylaxis after a previous dose of hepatitis B vaccine and persons with a known anaphylactic reaction to any vaccine component (*1329*). No other adverse events after administration of hepatitis B vaccine have been demonstrated.

##### Pre-Exposure Vaccination

Hepatitis B vaccination is recommended for all unvaccinated children and adolescents; all unvaccinated adults at risk for HBV infection, especially injecting drug users; MSM; adults with multiple sex partners; sex partners, needle-sharing contacts, or household contacts of persons with chronic hepatitis B; and persons with diabetes and all adults seeking protection from HBV infection (*1318*). For adults, acknowledgment of a specific risk factor is not a requirement for vaccination.

Hepatitis B vaccine should be routinely offered to all unvaccinated persons attending STD clinics and to all unvaccinated persons seeking evaluation or treatment for STIs in other settings, especially correctional facilities, facilities providing substance misuse treatment and prevention services, Federally Qualified Health Centers, and settings serving MSM (e.g., HIV infection care and prevention settings). If hepatitis B vaccine is unavailable at a particular facility, persons should be linked to a setting where they can receive vaccine. Persons with a reliable vaccination history (i.e., a written, dated record of each dose of a complete series) or reliable history of hepatitis B infection (i.e., a written record of infection and serologic results providing evidence of previous infection) do not require vaccination. In all settings, vaccination should be initiated at the initial visit, even if concerns about completion of the vaccine series exist.

##### Prevaccination Serologic Testing

Conducting prevaccination serologic testing for susceptibility just before the initial vaccine dose is administered can be considered for identifying persons with chronic HBV infection and, potentially, reducing the cost of completing the vaccination series for adult populations that have an expected high prevalence (20%–30%) of HBV infection (e.g., injecting drug users and MSM, especially those among older age groups, or persons born where HBV endemicity is moderate to high). In addition, prevaccination testing for susceptibility is recommended for unvaccinated household, sexual, and needle-sharing contacts of HBsAg-positive persons (*1318*). Serologic testing should not be a barrier to vaccination. The first vaccine dose should be administered immediately after collection of the blood sample for serologic testing. Vaccination of persons who are immune to HBV infection because of current or previous infection or vaccination is not harmful and does not increase the risk for adverse events.

Prevaccination testing should be performed with HBsAg, anti-HBs, and total anti-HBc to define patients’ HBV clinical status and deliver recommended care (*1330*). Persons who test HBsAg positive should receive prevention counseling and evaluation for antiviral treatment (see Management of Persons Who Are HBsAg Positive). Persons who test total anti-HBc positive and anti-HBs positive should be counseled that they have had previous HBV infection and are immune. Those persons with isolated anti-HBc (i.e., negative HBsAg and anti-HBs) need further assessment to rule out occult HBV infection, and they are at higher risk for reactivation if exposed to immunosuppressants. Persons who test negative to all three HBV seromarkers should receive the complete vaccination series, with the first vaccine dose administered immediately.

##### Postvaccination Serologic Testing for Response

Postvaccination serologic testing for immunity is unnecessary after routine vaccination of adolescents or adults. However, such testing is recommended for persons whose subsequent clinical management depends on knowledge of their immune status. Persons recommended to receive postvaccination serologic testing include health care personnel and public safety workers, persons with HIV infection, sex and needle-sharing partners of HBsAg-positive persons, hemodialysis patients and others who might require outpatient hemodialysis (e.g., predialysis, peritoneal dialysis, or home dialysis), and other immunocompromised persons (e.g., hematopoietic stem-cell transplant recipients or persons receiving chemotherapy) (*1318*).

If indicated, anti-HBs testing should be performed 1–2 months after administration of the last dose of the vaccine series. Persons determined to have anti-HBs levels of <10 mIU/mL after the primary vaccine series should be revaccinated with a 3-dose series and tested again for anti-HBs 1–2 months after the third dose. Persons who do not respond to revaccination should be tested for HBsAg and HBc. If HBsAg positive, persons should receive recommended management (see Management of Persons Who Are HBsAg Positive). If HBsAg negative, persons should be considered susceptible to HBV infection and counseled about precautions for preventing HBV infection and the need for HBIG PEP for any known exposure. If isolated anti-HBc positive (i.e., negative HBsAg and anti-HBs), persons will need further assessment to rule out occult HBV infection and are at higher risk for reactivation if exposed to immunosuppressants.

##### Postexposure Prophylaxis

Both passive and active PEP (simultaneous administration of HBIG [i.e., 0.06 mL/kg body weight] and hepatitis B vaccine at separate anatomic sites) and active PEP (administration of hepatitis B vaccination alone) have been demonstrated to be highly effective in preventing transmission after exposure to HBV (*12*). HBIG alone also has been demonstrated to be effective in preventing HBV transmission; however, with the availability of hepatitis B vaccine, HBIG typically is used as an adjunct to vaccination.

##### Exposure to a Source Who Is HBsAg Positive

Unvaccinated persons or persons known not to have responded to a complete hepatitis B vaccine series should receive both HBIG and hepatitis vaccine as soon as possible (preferably ≤24 hours) after a discrete, identifiable exposure to blood or body fluids that contain blood from a person with HBsAg (Table 7). Hepatitis B vaccine should be administered simultaneously with HBIG at a separate anatomic site, and the vaccine series should be completed by using the age-appropriate vaccine dose and schedule (Table 6). Exposed persons who are not fully vaccinated because they have not completed the vaccine series should receive HBIG (i.e., 0.06 mL/kg body weight) and complete the vaccine series. Persons who have written documentation of a complete hepatitis B vaccine series who did not receive postvaccination testing should receive a single vaccine booster dose. Exposed persons who are known to have responded to vaccination by postvaccination testing are considered protected; therefore, they need no additional doses of vaccine or HBIG. All persons with an occupational exposure to blood or body fluids that contain HBV should be managed according to guidelines (*12*).

**TABLE 7:** Guidelines for postexposure prophylaxis* of persons with nonoccupational exposure^† ^to blood or body fluids that contain blood, by exposure type and hepatitis B vaccination status

Source of exposure	Unvaccinated person^§^	Previously vaccinated person^¶^
**HBsAg-positive source **Percutaneous (e.g., bite or needlestick) or mucosal exposure to HBsAg-positive blood or body fluids ***or ***Sex or needle-sharing contact with an HBsAg-positive person ***or ***Victim of sexual assault or abuse by an assailant who is HBsAg positive	Administer hepatitis B vaccine series and HBIG	Complete hepatitis B vaccine series and HBIG, if vaccine series not completed ***or ***Administer hepatitis B vaccine booster dose, if previous vaccination without testing**
**Source with unknown HBsAg status **Percutaneous (e.g., bite or needlestick) or mucosal exposure to potentially infectious blood or body fluids from a source with unknown HBsAg status ***or ***Sex or needle-sharing contact with person with unknown HBsAg status ***or ***Victim of sexual assault or abuse by a perpetrator with unknown HBsAg status	Administer hepatitis B vaccine series	Complete hepatitis B vaccine series

**Sources:** CDC. CDC guidance for evaluating health-care personnel for hepatitis B virus protection and for administering postexposure management. MMWR Recomm Rep 2013;62(No. RR-10); CDC. Postexposure prophylaxis to prevent hepatitis B virus infection. MMWR Recomm Rep 2006;55(No. RR-16).

**Abbreviations:** HBIG = hepatitis B immune globulin; HBsAg = hepatitis B surface antigen.

* When indicated, immunoprophylaxis should be initiated as soon as possible, preferably within 24 hours. Studies are limited regarding the maximum interval after exposure during which postexposure prophylaxis is effective, but the interval is unlikely to exceed 7 days for percutaneous exposures or 14 days for sexual exposures. The hepatitis B vaccine series should be completed. These guidelines apply to nonoccupational exposures.

^† ^These guidelines apply to nonoccupational exposures.

^§ ^A person who is in the process of being vaccinated but who has not completed the vaccine series should complete the series and receive treatment for hepatitis B as indicated.

^¶ ^A person who has written documentation of a complete hepatitis B vaccine series and who did not receive postvaccination testing.

** No booster dose is needed for persons who have written documentation of hepatitis B vaccine series with serologic response.

##### Exposure to a Source with Unknown HBsAg Status

Unvaccinated persons and persons with previous nonresponse to hepatitis B vaccination who have a discrete, identifiable exposure to blood or body fluids containing blood from a person with unknown HBsAg status should receive the hepatitis B vaccine series, with the first dose initiated as soon as possible after exposure (preferably <24 hours) and the series completed according to the age-appropriate dose and schedule. Exposed persons who are not fully vaccinated but started the series should complete the vaccine series. Exposed persons with written documentation of a complete hepatitis B vaccine series who did not receive postvaccination testing require no further treatment.

##### Other Management Considerations

All persons with HBV infection should be tested for HIV, syphilis, gonorrhea, and chlamydia.

#### Management of Persons Who Are HBsAg Positive

Recommendations for management of all persons with HBsAg include the following:

- All persons with HBsAg documented on laboratory results should be reported to the state or local health department.
- To verify the presence of chronic HBV infection, persons with HBsAg should be retested. The absence of IgM anti-HBc or the persistence of HBsAg for ≥6 months indicates chronic HBV infection.
- Persons with chronic HBV infection should be referred for evaluation to a specialist experienced in managing chronic hepatitis B infection.
- Household, sexual, and needle-sharing contacts of persons with chronic infection should be evaluated. Unvaccinated sex partners and household and needle-sharing contacts should be tested for susceptibility to HBV infection and receive the first dose of hepatitis B vaccine immediately after collection of the blood sample for serologic testing (see Prevaccination Serologic Testing). Susceptible persons should complete the vaccine series by using an age-appropriate vaccine dose and schedule.
- Sex partners of persons with HBsAg should be counseled to use latex condoms (*1331*) to protect themselves from sexual exposure to infectious body fluids (e.g., semen and vaginal secretions), unless they have been demonstrated to be immune after vaccination (anti-HBs ≥10 mIU/mL) or previously infected (anti-HBc positive).
- To prevent or reduce the risk for transmission to others in addition to vaccination, persons with HBsAg also should be advised to
  º use methods (e.g., condoms) to protect nonimmune sex partners from acquiring HBV infection from sexual activity until the partner can be vaccinated and immunity documented;
  º cover cuts and skin lesions to prevent spread by infectious secretions or blood;
  º refrain from donating blood, plasma, body organs, other tissue, or semen; and
  º refrain from sharing household articles (e.g., toothbrushes, razors, or personal injecting equipment) that could become contaminated with blood, and refrain from premastication of food.
- To protect the liver from further harm, persons with HBsAg should be advised to
  º avoid or limit alcohol consumption because of the effects of alcohol on the liver;
  º refrain from starting any new medicines, including over-the-counter and herbal medicines, without checking with their health care provider; and
  º obtain vaccination against hepatitis A.

When seeking medical or dental care, persons who are HBsAg positive should be advised to inform their health care providers of their HBsAg status so that they can be evaluated and managed. The following are key counseling messages for persons with HBsAg:

- HBV is not usually spread by hugging, coughing, food or water, sharing eating utensils or drinking glasses, or casual contact.
- Persons should not be excluded from work, school, play, childcare, or other settings because they are infected with HBV.
- Involvement with a support group might help patients cope with chronic HBV infection.
- HBV infection is a chronic condition that can be treated, and patients should receive prevention counseling and be evaluated for antiviral treatment.

#### Special Considerations

##### Pregnancy

Regardless of whether they have been previously tested or vaccinated, all pregnant women should be tested for HBsAg at the first prenatal visit and again at delivery if at high risk for HBV infection (see STI Detection Among Special Populations). Pregnant women at risk for HBV infection and without documentation of a complete hepatitis B vaccine series should receive hepatitis B vaccination. All pregnant women with HBsAg should be reported to state and local perinatal hepatitis B prevention programs and referred to a specialist. Information about management of pregnant women with HBsAg and their infants is available at https://www.cdc.gov/hepatitis/hbv/perinatalxmtn.htm.

##### HIV Infection

HIV infection can impair the response to hepatitis B vaccination. Persons with HIV should be tested for anti-HBs 1–2 months after the third vaccine dose (see Postvaccination Serologic Testing). Modified dosing regimens, including a doubling of the standard antigen dose and administration of additional doses, might increase the response rate and should be managed in consultation with an infectious disease specialist. Additional recommendations for management of persons with HBsAg and HIV infection are available (*98*).

### Hepatitis C Virus Infection

HCV infection is the most common chronic bloodborne infection in the United States, with an estimated 2.4 million persons living with chronic infection (*1332*). HCV is not efficiently transmitted through sex (*1333*–*1335*). Studies of HCV transmission between heterosexual couples and MSM have yielded mixed results; however, studies have reported either no or minimally increased rates of HCV infection among partners of persons with HCV infection compared with partners of those without HCV (*1334*,*1336*–*1338*). However, data indicate that sexual transmission of HCV can occur, especially among persons with HIV infection. Increasing incidence of acute HCV infection among MSM with HIV infection has been reported in multiple U.S. (*96*,*236*,*239*,*1339*) and European cities (*237*,*1340*–*1342*). A recent systematic review reported an HCV incidence of 6.35 per 1,000 person years among MSM with HIV infection (*1343*). An association exists with high-risk and traumatic sexual practices (e.g., condomless receptive anal intercourse or receptive fisting) and concurrent genital ulcerative disease or STI-related proctitis (*237*,*1342*). HCV transmission among MSM with HIV infection has also been associated with group sex and chemsex (i.e., using recreational drugs in a sexual context) (*1344*–*1348*). Shedding of HCV in the semen and in the rectum of men with HIV infection has been documented (*1349*,*1350*). Certain studies have revealed that risk increases commensurate with increasing numbers of sex partners among heterosexual persons (*1337*,*1338*,*1351*–*1353*) and MSM with HIV infection (*1349*,*1354*–*1357*), especially if their partners are also coinfected with HIV (*237*,*1340*,*1354*–*1356*,*1358*). More recently, acute HCV infections have been reported among MSM on PrEP, increasing concerns that certain MSM might be at increased risk for incident HCV infection through condomless sexual intercourse with MSM with HCV infection (*1359*,*1360*).

Persons newly infected with HCV typically are either asymptomatic or have a mild clinical illness. HCV RNA can be detected in blood within 1–3 weeks after exposure. The average time from exposure to antibody to HCV (anti-HCV) seroconversion is 4−10 weeks, and anti-HCV can be detected among approximately 97% of persons by 6 months after exposure (*1361*–*1364*) (https://www.cdc.gov/hepatitis/hcv/hcvfaq.htm#section3).

Chronic HCV infection develops among 75%–85% of persons with HCV infection (*1365*,*1366*), and 10%–20% of persons with chronic infection develop cirrhosis in 20–30 years of active liver disease (*1367*). The majority of infected persons remain unaware of their infection because they are not clinically ill. However, infected persons are a source of transmission to others and are at risk for cirrhosis and hepatocellular carcinoma decades after infection.

HCV is primarily transmitted parenterally, usually through shared drug-injecting needles and paraphernalia. HCV also can be transmitted through exposures in health care settings as a consequence of inadequate infection control practices (*1314*). Transmission after receipt of blood from donors and from transplantation of tissues and organs with HCV infection has occurred only rarely since 1992, when routine screening of these donated products was mandated in the United States (*1367*,*1369*). Tattoos applied in regulated settings have not been associated with HCV transmission, although those obtained in certain settings have been linked to such transmission (*1336*). Occupational and perinatal exposures also can result in transmission of HCV; however, such transmission is uncommon.

Acute HCV infection is a reportable condition in 49 states. Matching viral hepatitis and HIV surveillance registries, and molecular epidemiologic assessments, can facilitate early detection of social networks of HCV transmission among MSM with HIV infection.

CDC recommends hepatitis C screening at least once in a lifetime for all adults aged ≥18 years and for all women during each pregnancy, except in settings where the prevalence of HCV infection is <0.1% (*156*). One-time hepatitis C testing is also recommended regardless of age, setting, or recognized conditions or exposures (e.g., HIV infection, history of injecting drug use, or children born to women with HCV infection). Routine periodic HCV testing is recommended for persons with ongoing risk factors (e.g., injecting drug use or hemodialysis).

#### Diagnosis

Testing for HCV infection should include use of an FDA-cleared test for antibody to HCV (i.e., immunoassay, EIA, or enhanced CIA and, if recommended, a supplemental antibody test) followed by NAAT to detect HCV RNA for those with a positive antibody result (*1370*). Persons with HIV infection with low CD4^+ ^T-cell count might require further testing by NAAT because of the potential for a false-negative antibody assay.

Persons determined to have HCV infection (i.e., positive for HCV RNA) should be evaluated for treatment. Antibody to HCV remains positive after spontaneously resolving or successful treatment; therefore, subsequent testing for HCV reinfection among persons with ongoing risk factors should be limited to HCV RNA. Persons who have spontaneous resolution or who have undergone successful treatment are not immune to reinfection.

#### Treatment

HCV infection is curable, and persons with diagnosed HCV infection should be linked to care and treatment. Providers should consult existing guidelines to learn about the latest advances in treating HCV infection (https://www.hcvguidelines.org) and with hepatitis specialists, as needed. Persons at high risk for transmitting HCV to others should be treated both for individual benefit and to prevent HCV transmission.

#### Management of Sex Partners

Because incident HCV has not been demonstrated to occur among heterosexual couples followed over time (*1334*,*1371*–*1373*), condom use might not be necessary in such circumstances. Persons with HCV infection with one long-term, steady sex partner do not need to change their sexual practices. However, they should discuss the risk for transmission with their partner and discuss the need for testing (*234*) (https://www.cdc.gov/hepatitis/hcv/index.htm). Heterosexual persons and MSM with HCV infection and more than one partner, especially those with concurrent HIV infection, should protect their partners against HCV and HIV acquisition by using external latex condoms (*237*,*1358*,*1374*) and HIV PrEP. Partners of persons with HCV and HIV should be tested for both infections.

#### Other Management Considerations

All persons with HCV infection for whom HIV and HBV infection status is unknown should be tested for these infections. Those who have HIV or HBV infection should be referred for or provided with recommended care and treatment. Persons without previous exposure to HAV or HBV should be vaccinated.

#### Prevention

Reducing the burden of HCV infection and disease in the United States requires implementing both primary and secondary prevention activities. Primary prevention reduces or eliminates HCV transmission, whereas secondary prevention identifies persons through screening and then provides treatment to reduce chronic liver disease and other chronic diseases and HCV transmission. No vaccine for hepatitis C is available, and prophylaxis with IG is not effective in preventing HCV infection after exposure. PEP using direct-acting antivirals is not recommended.

Persons with HCV infection should be provided information about how to protect their liver from further harm (i.e., hepatotoxic agents); for instance, persons with HCV infection should be advised to avoid drinking alcohol and taking any new medicines, including over-the-counter or herbal medications, without checking with their clinician. In addition, a need for hepatitis A and B vaccination should be determined; persons who are not immune should be vaccinated.

To reduce the risk for transmission to others, persons with HCV infection should be advised not to donate blood, body organs, other tissue, or semen; not to share any personal items that might have blood on them (e.g., toothbrushes or razors); and to cover cuts and sores on the skin to keep the virus from spreading by blood or secretions. Women with HCV infection do not need to avoid pregnancy or breastfeeding, although children born to women with HCV also should be tested for HCV.

Persons who use or inject drugs should be counseled about the importance of prevention and provided access to substance misuse treatment, including medication-assisted treatment, if indicated. Persons who inject drugs should be encouraged to take the following additional steps to reduce personal and public health risks:

- Never reuse or share syringes, water, or drug preparation equipment.
- Only use syringes obtained from a reliable source (e.g., a syringe services program or a pharmacy).
- Use a new, sterile syringe to prepare and inject drugs each time.
- If possible, use sterile water to prepare drugs; otherwise, use clean water from a reliable source (e.g., fresh tap water).
- Use a new or disinfected container (i.e., cooker) and a new filter (i.e., cotton) to prepare drugs.
- Clean the injection site with a new alcohol swab before injection.
- Safely dispose of syringes after one use.

#### Postexposure Follow-Up

No PEP has been demonstrated to be effective against HCV infection. Testing for HCV is recommended for health care workers after percutaneous or perimucosal exposures to HCV-positive blood. Prompt identification of acute infection is vital because outcomes are improved when treatment is initiated early during the illness course.

#### Special Considerations

##### Pregnancy

All pregnant women should be screened with each pregnancy for HCV antibodies at the first prenatal visit in settings where the HCV prevalence is >0.1% (https://www.cdc.gov/hepatitis/hcv/index.htm) (*154*,*155*). Although the rate of transmission is highly variable, more than six of every 100 infants born to women with HCV infection become infected; this infection occurs predominantly during or near delivery, and no treatment or delivery method (e.g., cesarean delivery) has been demonstrated to decrease this risk (*1375*). However, the risk is increased by the presence of maternal HCV viremia at delivery and is twofold to threefold greater if the woman has HIV infection. Although no recommendations are available for HCV treatment during pregnancy, discussion about the individual risks and benefits of postpartum treatment can be considered in accordance with existing guidance (https://www.hcvguidelines.org/unique-populations/pregnancy).

HCV has not been reported to be transmitted through breast milk, although mothers with HCV infection should consider abstaining from breastfeeding if their nipples are cracked or bleeding. Infants born to mothers with HCV infection should be tested for HCV infection; children should be tested for anti-HCV no sooner than age 18 months because anti-HCV from the mother might last until that age. If diagnosis is desired before the child reaches age 18 months, testing for HCV RNA can be performed at or after the infant’s first well-child visit at age 1–2 months. HCV RNA testing can be repeated at a subsequent visit, independent of the initial HCV RNA test result (*1376*) (https://www.cdc.gov/hepatitis/hcv/hcvfaq.htm#section3).

##### HIV Infection

All persons with HIV infection should undergo serologic screening for HCV at initial evaluation (*98*) (https://www.hcvguidelines.org). Providers should be aware of the likelihood that MSM with HIV infection can acquire HCV after initial screening. Because acute HCV infection acquisition among persons with HIV infection can occur, especially among MSM, and regular screening of those with HIV is cost-effective (*238*,*239*,*1377*), periodic HCV screening should be conducted (*1378*–*1380*). For persons with HIV infection, hepatitis C screening with HCV antibody assays (followed by HCV RNA if antibody positive) can be considered at least yearly, for those at high risk for infection, and more frequently depending on specific circumstances (e.g., community HCV infection prevalence and incidence, high-risk sexual behavior, and concomitant ulcerative STIs and proctitis). Antibody to HCV remains positive after spontaneously resolved infection or successful treatment; therefore, subsequent testing for potential HCV reinfection among persons with ongoing risk should be limited to HCV RNA testing only. Indirect testing (e.g., alanine aminotransferase [ALT]) is not recommended for detecting incident HCV infections because such testing, especially if performed once a year, can miss persons who have reverted after acute HCV infection to a normal ALT level at the time of testing (*239*) (https://www.hcvguidelines.org). Conversely, ALT can be elevated by antiretroviral and other medications, alcohol, and toxins. If ALT levels are being monitored, persons with HIV infection who experience new or unexplained increases in ALT should be tested for acute HCV infection and evaluated for possible medication toxicity or excessive alcohol use.

Continued unprotected sexual contact between partners with HIV can facilitate spread of HCV infection because the virus can be recovered from the semen of men with HIV infection (*1349*,*1381*). Specific prevention practices (e.g., barrier precautions that limit contact with body fluids during sexual contact with other MSM) should be discussed.

Because a minimal percentage of persons with HIV infection do not develop HCV antibodies, HCV RNA testing should be performed for persons with HIV infection and unexplained liver disease who are anti-HCV negative. The course of liver disease is more rapid among persons with HIV and HCV, and the risk for cirrhosis is higher than that for persons with HCV infection alone.

## Proctitis, Proctocolitis, and Enteritis

Sexually transmitted gastrointestinal syndromes include proctitis, proctocolitis, and enteritis. Evaluation for these syndromes should include recommended diagnostic procedures, including anoscopy or sigmoidoscopy, stool examination for WBCs, and microbiologic workup (e.g., gonorrhea, chlamydia [LGV PCR if available], herpes simplex NAAT, and syphilis serology). For those with enteritis, stool culture or LGV PCR also is recommended.

Proctitis is inflammation of the rectum (i.e., the distal 10–12 cm) that can be associated with anorectal pain, tenesmus, or rectal discharge. Fecal leukocytes are common. Proctitis occurs predominantly among persons who have receptive anal exposures (oral-anal, digital-anal, or genital-anal). *N. gonorrhoeae*, *C. trachomatis* (including LGV serovars), HSV, and *T. pallidum* are the most common STI pathogens. Genital HSV and LGV proctitis are more prevalent among persons with HIV infection (*545*,*556*,*1382*). *M. genitalium* has been detected in certain cases of proctitis and might be more common among persons with HIV infection (*937*,*1382*). *N. meningitidis* has been identified as an etiology of proctitis among MSM with HIV infection (*1383*).

Proctocolitis is associated with symptoms of proctitis, diarrhea or abdominal cramps, and inflammation of the colonic mucosa extending to 12 cm above the anus. Fecal leukocytes might be detected on stool examination, depending on the pathogen. Proctocolitis can be acquired through receptive anal intercourse or by oral-anal contact, depending on the pathogen.

Pathogenic organisms include *Campylobacter* species, *Shigella* species, *E. histolytica*, LGV serovars of *C. trachomatis,* and *T. pallidum*. Among immunosuppressed persons with HIV infection, CMV or other opportunistic agents should be considered. The clinical presentation can be mistaken for inflammatory bowel disease or malignancy, resulting in a delayed diagnosis (*1384*,*1385*).

Enteritis usually results in diarrhea and abdominal cramping without signs of proctitis or proctocolitis. Fecal leukocytes might be detected on stool examination, depending on the pathogen. When outbreaks of gastrointestinal illness occur among social or sexual networks of MSM, clinicians should consider sexual transmission as a mode of spread and provide counseling accordingly. Sexual practices that can facilitate transmission of enteric pathogens include oral-anal contact or, in certain instances, direct genital-anal contact. *G. lamblia* is the most frequently implicated parasite, and bacterial pathogens include *Shigella* species, *Salmonella*, *E. coli, Campylobacter* species, and *Cryptosporidium*. Outbreaks of *Shigella* species, *Campylobacter*, *Cryptosporidium*, and microsporidiosis have been reported among MSM (*259*,*274*,*1386*,*1387*). Multiple enteric pathogens and concurrent STIs have also been reported. Among immunosuppressed persons with HIV infection, CMV or other opportunistic pathogens should be considered.

### Diagnostic and Treatment Considerations for Acute Proctitis

#### Diagnosis

Persons with symptoms of acute proctitis should be examined by anoscopy. A Gram-stained smear of any anorectal exudate from anoscopic or anal examination should be examined for polymorphonuclear leukocytes. All persons should be evaluated for herpes simplex (preferably by NAAT of rectal lesions), *N. gonorrhoeae* (NAAT or culture), *C. trachomatis* (NAAT), and *T. pallidum* (darkfield of lesion if available and serologic testing). If the *C. trachomatis* NAAT test is positive on a rectal swab and severe symptoms associated with LGV are present (including rectal ulcers, anal discharge, bleeding, ≥10 WBCs on Gram stain, and tenesmus), patients should be treated empirically for LGV. Molecular testing for LGV is not widely available or not FDA cleared, and results are not typically available in time for clinical decision-making. However, if available, molecular PCR testing for *C. trachomatis* serovars L1, L2, or L3 can be considered for confirming LGV (*553*).

The pathogenic role of *M. genitalium* in proctitis is unclear. For persons with persistent symptoms after standard treatment, providers should consider testing for *M. genitalium* with NAAT and treat if positive (see *Mycoplasma genitalium*).

#### Treatment 

Acute proctitis among persons who have anal exposure through oral, genital, or digital contact is usually sexually acquired (*1382*,*1388*). Presumptive therapy should be initiated while awaiting results of laboratory tests for persons with anorectal exudate detected on examination or polymorphonuclear leukocytes detected on a Gram-stained smear of anorectal exudate or secretions. Such therapy also should be initiated when anoscopy or Gram stain is not available and the clinical presentation is consistent with acute proctitis for persons reporting receptive anal exposures.

**Recommended Regimen for Acute Proctitis**
**Ceftriaxone** 500 mg* IM in a single dose
*plus*
**Doxycycline** 100 mg orally 2 times/day for 7 days^†^
* For persons weighing ≥150 kg, 1 g of ceftriaxone should be administered.
^†^ Doxycycline course should be extended to 100 mg orally 2 times/day for 21 days in the presence of bloody discharge, perianal or mucosal ulcers, or tenesmus and a positive rectal chlamydia test.

Bloody discharge, perianal ulcers, or mucosal ulcers among persons with acute proctitis and rectal chlamydia (NAAT) should receive presumptive treatment for LGV with an extended course of doxycycline 100 mg orally 2 times/day for 3 weeks (*1389*,*1390*) (see Lymphogranuloma Venereum). If painful perianal ulcers are present or mucosal ulcers are detected on anoscopy, presumptive therapy should also include a regimen for genital herpes (see Genital Herpes).

### Diagnostic and Treatment Considerations for Proctocolitis or Enteritis

Treatment for proctocolitis or enteritis should be directed to the specific enteric pathogen identified. Multiple stool examinations might be necessary for detecting *Giardia*, and special stool preparations are required for diagnosing cryptosporidiosis and microsporidiosis. Diagnostic and treatment recommendations for all enteric infections are beyond the scope of these guidelines. Providers should be aware of the potential for antimicrobial-resistant pathogens, particularly during outbreaks of *Shigella* and *Campylobacter* among sexual networks of MSM where increased resistance to azithromycin, fluoroquinolones, and isolates resistant to multiple antibiotics have been described (*266*,*272*,*273*,*1391*,*1392*).

### Other Management Considerations

To minimize transmission and reinfection, patients treated for acute proctitis should be instructed to abstain from sexual intercourse until they and their partners have been treated (i.e., until completion of a 7-day regimen and symptoms have resolved). Studies have reported that behaviors that facilitate enteric pathogen transmission might be associated with acquisition of other STIs, including HIV infection. All persons with acute proctitis and concern for sexually transmitted proctocolitis or enteritis should be tested for HIV, syphilis, gonorrhea, and chlamydia (at other exposed sites). PEP should be considered for exposures that present a risk for HIV acquisition. For ongoing risk for HIV acquisition, PrEP should be considered.

Evidence-based interventions for preventing acquisition of sexually transmitted enteric pathogens are not available. However, extrapolating from general infection control practices for communicable diseases and established STI prevention practices, recommendations include avoiding contact with feces during sex, using barriers, and washing hands after handing materials that have been in contact with the anal area (i.e., barriers and sex toys) and after touching the anus or rectal area.

### Follow-Up

Follow-up should be based on specific etiology and severity of clinical symptoms. For proctitis associated with gonorrhea or chlamydia, retesting for the respective pathogen should be performed 3 months after treatment.

### Management of Sex Partners

Partners who have had sexual contact with persons treated for gonorrhea or chlamydia <60 days before the onset of the persons symptoms should be evaluated, tested, and presumptively treated for the respective infection. Partners of persons with proctitis should be evaluated for any diseases diagnosed in the index partner. Sex partners should abstain from sexual contact until they and their partners are treated. No specific recommendations are available for screening or treating sex partners of persons with diagnosed sexually transmitted enteric pathogens; however, partners should seek care if symptomatic.

### Special Considerations

#### Drug Allergy, Intolerance, and Adverse Reactions

Allergic reactions with third-generation cephalosporins (e.g., ceftriaxone) are uncommon among persons with a history of penicillin allergy (*620*,*631*,*658*,*896*).

#### HIV Infection 

Persons with HIV infection and acute proctitis might present with bloody discharge, painful perianal ulcers, or mucosal ulcers and LGV and herpes proctitis are more prevalent among this population. Presumptive treatment in such cases should include a regimen for genital herpes and LGV.

## Ectoparasitic Infections

### Pediculosis Pubis

Persons who have pediculosis pubis (i.e., pubic lice) usually seek medical attention because of pruritus or because they notice lice or nits on their pubic hair. Pediculosis pubis is caused by the parasite *Phthirus pubis* and is usually transmitted by sexual contact (*1393*).

#### Diagnosis

The clinical diagnosis is based on typical symptoms of itching in the pubic region. Lice and nits can be observed on pubic hair.

#### Treatment

**Recommended Regimens for Pediculosis Pubis**
**Permethrin 1% cream rinse** applied to affected areas and washed off after 10 minutes
*or*
**Pyrethrin with piperonyl butoxide** applied to the affected area and washed off after 10 minutes
**Alternative Regimens**
**Malathion 0.5% lotion** applied to affected areas and washed off after 8–12 hours
*or*
**Ivermectin** 250 *µ*g/kg body weight orally, repeated in 7–14 days

Reported resistance to pediculicides (permethrin and pyrethrin) has been increasing and is widespread (*1394*,*1395*). Malathion can be used when treatment failure is believed to have occurred as a result of resistance. The odor and longer duration of application associated with malathion therapy make it a less attractive alternative compared with the recommended pediculicides. Ivermectin has limited ovicidal activity (*1396*). Ivermectin might not prevent recurrences from eggs at the time of treatment, and therefore treatment should be repeated in 7–14 days (*1397*,*1398*). Ivermectin should be taken with food because bioavailability is increased, thus increasing penetration of the drug into the epidermis. Adjustment of ivermectin dosage is not required for persons with renal impairment; however, the safety of multiple doses among persons with severe liver disease is unknown. Lindane is not recommended for treatment of pediculosis because of toxicity, contraindications for certain populations (pregnant and breastfeeding women, children aged <10 years, and those with extensive dermatitis), and complexity of administration.

#### Other Management Considerations

The recommended regimens should not be applied to the eyes. Pediculosis of the eyelashes should be treated by applying occlusive ophthalmic ointment or petroleum jelly to the eyelid margins 2 times/day for 10 days. Bedding and clothing should be decontaminated (i.e., machine washed and dried by using the heat cycle or dry cleaned) or removed from body contact for at least 72 hours. Fumigation of living areas is unnecessary. Pubic hair removal has been associated with atypical patterns of pubic lice infestation and decreasing incidence of infection (*537*,*1399*). Persons with pediculosis pubis should be evaluated for HIV, syphilis, chlamydia, and gonorrhea.

#### Follow-Up

Evaluation should be performed after 1 week if symptoms persist. Retreatment might be necessary if lice are found or if eggs are observed at the hair-skin junction. If no clinical response is achieved to one of the recommended regimens, retreatment with an alternative regimen is recommended.

#### Management of Sex Partners

Sex partners within the previous month should be treated. Sexual contact should be avoided until patients and partners have been treated, bedding and clothing decontaminated, and reevaluation performed to rule out persistent infection.

#### Special Considerations

##### Pregnancy

Existing data from human participants demonstrate that pregnant and lactating women should be treated with either permethrin or pyrethrin with piperonyl butoxide. Because no teratogenicity or toxicity attributable to ivermectin has been observed during human pregnancy experience, ivermectin is classified as “human data suggest low risk” during pregnancy and probably compatible with breastfeeding (*431*).

##### HIV Infection

Persons who have pediculosis pubis and HIV infection should receive the same treatment regimen as those who do not have HIV.

### Scabies

Scabies is a skin infestation caused by the mite *Sarcoptes scabiei*, which causes pruritus. Sensitization to *S. scabiei* occurs before pruritus begins. The first time a person is infested with *S. scabiei*, sensitization takes weeks to develop. However, pruritus might occur <24 hours after a subsequent reinfestation. Scabies among adults frequently is sexually acquired, although scabies among children usually is not (*1400*–*1402*).

#### Diagnosis

Scabies diagnosis is made by identifying burrows, mites, eggs, or the mites’ feces from affected areas. Skin scrapings can be examined under the microscope to identify organisms, although this method has low sensitivity and is time consuming (*1403*). Alternatively, noninvasive examination of the affected skin by using videodermatoscopy, videomicroscopy, or dermoscopy can be used, each of which has high sensitivity and specificity, particularly when performed by experienced operators (*1404*). Low-technology strategies include the burrow ink test and the adhesive tape test.

#### Treatment

**Recommended Regimens for Scabies**
**Permethrin 5% cream** applied to all areas of the body from the neck down and washed off after 8–14 hours
*or*
**Ivermectin **200 ug/kg body weight orally, repeated in 14 days*
*or*
**Ivermectin 1% lotion** applied to all areas of the body from the neck down and washed off after 8–14 hours; repeat treatment in 1 week if symptoms persist
* Oral ivermectin has limited ovicidal activity; a second dose is required for eradication.
**Alternative Regimen**
**Lindane 1%** 1 oz of lotion or 30 g of cream applied in a thin layer to all areas of the body from the neck down and thoroughly washed off after 8 hours*
* Infants and children aged <10 years should not be treated with lindane.

Topical permethrin and oral and topical ivermectin have similar efficacy for cure of scabies (*1405*–*1410*). Choice of treatment might be based on patient preference for topical versus oral therapy, drug interactions with ivermectin (e.g., azithromycin, trimethoprim/sulfamethoxazole [Bactrim], or cetirizine [Zytrec]), and cost. Permethrin is safe and effective with a single application (*1411*). Ivermectin has limited ovicidal activity and might not prevent recurrences of eggs at the time of treatment; therefore, a second dose of ivermectin should be administered 14 days after the first dose (*1412*). Ivermectin should be taken with food because bioavailability is increased, thereby increasing penetration of the drug into the epidermis. Adjustments to ivermectin dosing are not required for patients with renal impairment; however, the safety of multiple doses among patients with severe liver disease is unknown.

Lindane is an alternative regimen because it can cause toxicity (*1413*); it should be used only if the patient cannot tolerate the recommended therapies or if these therapies have failed (*1414*–*1416*). Lindane is not recommended for pregnant and breastfeeding women, children aged <10 years, and persons with extensive dermatitis. Seizures have occurred when lindane was applied after a bath or used by patients who had extensive dermatitis. Aplastic anemia after lindane use also has been reported (*1413*). Lindane resistance has been reported in some areas of the world, including parts of the United States (*1413*).

#### Other Management Considerations

Bedding and clothing should be decontaminated (i.e., either machine washed and dried by using the heat cycle or dry cleaned) or removed from body contact for >72 hours. Fumigation of living areas is unnecessary. Persons with scabies should be advised to keep fingernails closely trimmed to reduce injury from excessive scratching (*1417*).

#### Crusted Scabies

Crusted scabies is an aggressive infestation that usually occurs among immunodeficient, debilitated, or malnourished persons, including persons receiving systemic or potent topical glucocorticoids, organ transplant recipients, persons with HIV infection or human T-lymphotropic virus-1 infection, and persons with hematologic malignancies. Crusted scabies is transmitted more easily than scabies (*1418*). No controlled therapeutic studies for crusted scabies have been conducted, and a recommended treatment remains unclear. Substantial treatment failure might occur with a single-dose topical scabicide or with oral ivermectin treatment. Combination treatment is recommended with a topical scabicide, either 5% topical permethrin cream (full-body application to be repeated daily for 7 days then 2 times/week until cure) or 25% topical benzyl benzoate, and oral ivermectin 200 *u*g/kg body weight on days 1, 2, 8, 9, and 15. Additional ivermectin treatment on days 22 and 29 might be required for severe cases (*1419*). Lindane should be avoided because of the risks for neurotoxicity with heavy applications on denuded skin.

#### Follow-Up

The rash and pruritus of scabies might persist for <2 weeks after treatment. Symptoms or signs persisting for >2 weeks can be attributed to multiple factors. Treatment failure can occur as a result of resistance to medication or faulty application of topical scabicides. These medications do not easily penetrate into thick, scaly skin of persons with crusted scabies, perpetuating the harboring of mites in these difficult-to-penetrate layers. In the absence of recommended contact treatment and decontamination of bedding and clothing, persisting symptoms can be attributed to reinfection by family members or fomites. Finally, other household mites can cause symptoms to persist as a result of cross-reactivity between antigens. Even when treatment is successful, reinfection is avoided, and cross-reactivity does not occur, symptoms can persist or worsen as a result of allergic dermatitis.

Retreatment 2 weeks after the initial treatment regimen can be considered for those persons who are still symptomatic or when live mites are observed. Use of an alternative regimen is recommended for those persons who do not respond initially to the recommended treatment.

#### Management of Sex Partners and Household Contacts

Persons who have had sexual, close personal, or household contact with the patient within the month preceding scabies infestation should be examined. Those identified as being infested should be provided treatment.

#### Management of Outbreaks in Communities, Nursing Homes, and Other Institutional Settings

Scabies epidemics frequently occur in nursing homes, hospitals, residential facilities, and other communities (*1420*,*1421*). Control of an epidemic can only be achieved by treating the entire population at risk. Ivermectin can be considered in these settings, especially if treatment with topical scabicides fails. Mass treatment with oral ivermectin is highly effective in decreasing prevalence in settings where scabies is endemic (*1422*). Epidemics should be managed in consultation with a specialist.

#### Special Considerations

##### Infants, Young Children, and Pregnant or Lactating Women

Infants and young children should be treated with permethrin; the safety of ivermectin for children weighing <15 kg has not been determined. Infants and children aged<10 years should not be treated with lindane. Ivermectin likely poses a low risk to pregnant women and is likely compatible with breastfeeding; however, because of limited data regarding ivermectin use for pregnant and lactating women, permethrin is the preferred treatment (*431*) (see Pediculosis Pubis).

##### HIV Infection

Persons with HIV infection who have uncomplicated scabies should receive the same treatment regimens as those who do not have HIV. Persons with HIV infection and others who are immunosuppressed are at increased risk for crusted scabies and should be managed in consultation with a specialist.

## Sexual Assault and Abuse and STIs

### Adolescents and Adults

These guidelines are primarily limited to the identification, prophylaxis, and treatment of STIs and conditions among adolescent and adult female sexual assault survivors. However, some of the following guidelines might still apply to male sexual assault survivors. Documentation of findings, collection of nonmicrobiologic specimens for forensic purposes, and management of potential pregnancy or physical and psychological trauma are beyond the scope of these guidelines. Examinations of survivors of sexual assault should be conducted by an experienced clinician in a way that minimizes further trauma to the person. The decision to obtain genital or other specimens for STI diagnosis should be made on an individual basis. Care systems for survivors should be designed to ensure continuity, including timely review of test results, support adherence, and monitoring for adverse reactions to any prescribed therapeutic or prophylactic regimens. Laws in all 50 states limit the evidentiary use of a survivor’s previous sexual history, including evidence of previously acquired STIs, as part of an effort to undermine the credibility of the survivor’s testimony. Evidentiary privilege against revealing any aspect of the examination or treatment also is enforced in most states. Although it rarely occurs, STI diagnoses might later be accessed, and the survivor and clinician might opt to defer testing for this reason. Although collection of specimens at initial examination for laboratory STI diagnosis gives the survivor and clinician the option of deferring empiric prophylactic antimicrobial treatment, compliance with follow-up visits is typically poor (*1423*–*1425*). Among sexually active adults, identification of an STI might represent an infection acquired before the assault, and therefore might be more important for the medical management of the patient than for legal purposes.

Trichomoniasis, BV, gonorrhea, and chlamydia are the most frequently diagnosed infections among women who have been sexually assaulted. Such conditions are prevalent among the population, and detection of these infections after an assault does not necessarily imply acquisition during the assault. However, a postassault examination presents an important opportunity for identifying or preventing an STI. Chlamydial and gonococcal infections among women are of particular concern because of the possibility of ascending infection. In addition, HBV infection can be prevented through postexposure vaccination (see Hepatitis B Virus Infection). Because persons who have been sexually assaulted also are at risk for acquiring HPV infection, and the efficacy of the HPV vaccine is high (*1426*,*1427*), HPV vaccination is also recommended for females and males through age 26 years (https://www.cdc.gov/vaccines/hcp/acip-recs/vacc-specific/hpv.html) (*11*). Reproductive-aged female survivors should be evaluated for pregnancy and offered emergency contraception.

#### Evaluating Adolescents and Adults for STIs

##### Initial Examination

Decisions to perform the following tests should be made on an individual basis. An initial examination after a sexual assault might include the following:

- NAATs for *C. trachomatis* and *N. gonorrhoeae* at the sites of penetration or attempted penetration should be performed (*553*). These tests are preferred for diagnostic evaluation of adolescent or adult sexual assault survivors.
- Females should be offered NAAT testing for *T. vaginalis* from a urine or vaginal specimen. POC or wet mount with measurement of vaginal pH and KOH application for the whiff test from vaginal secretions should be performed for evidence of BV and candidiasis, especially if vaginal discharge, malodor, or itching is present.
- MSM should be offered screening for *C. trachomatis* and *N. gonorrhoeae* if they report receptive oral or anal sex during the preceding year, regardless of whether sexual contact occurred at these anatomic sites during the assault. Anoscopy should be considered in instances of reported anal penetration.
- A serum sample should be performed for HIV, HBV, and syphilis infection.

#### Treatment

Compliance with follow-up visits is poor among survivors of sexual assault (*1423*–*1425*). Consequently, the following routine presumptive treatments after a sexual assault are recommended:

- An empiric antimicrobial regimen for chlamydia, gonorrhea, and trichomonas for women and chlamydia and gonorrhea for men.
- Emergency contraception should be considered when the assault could result in pregnancy (see Emergency Contraception).
- Postexposure hepatitis B vaccination (without HBIG) if the hepatitis status of the assailant is unknown and the survivor has not been previously vaccinated. If the assailant is known to be HBsAg positive, unvaccinated survivors should receive both hepatitis B vaccine and HBIG. The vaccine and HBIG, if indicated, should be administered to sexual assault survivors at the time of the initial examination, and follow-up doses of vaccine should be administered 1–2 and 4–6 months after the first dose. Survivors who were previously vaccinated but did not receive postvaccination testing should receive a single vaccine booster dose (see Hepatitis B Virus Infection).
- HPV vaccination for female and male survivors aged 9–26 years who have not been vaccinated or are incompletely vaccinated (*11*) (https://www.cdc.gov/vaccines/hcp/acip-recs/vacc-specific/hpv.html). The vaccine should be administered to sexual assault survivors at the time of the initial examination, and follow-up doses should be administered at 1–2 months and 6 months after the first dose. A 2-dose schedule (0 and 6–12 months) is recommended for persons initiating vaccination before age 15 years.
- Recommendations for HIV PEP are made on a case-by-case basis according to risk (see Risk for Acquiring HIV Infection; Recommendations for Postexposure HIV Risk Assessment of Adolescents and Adults <72 Hours After Sexual Assault).
**Recommended Regimen for Adolescent and Adult Female Sexual Assault Survivors**
  **Ceftriaxone **500 mg* IM in a single dose
*plus*
  **Doxycycline** 100 mg 2 times/day orally for 7 days
*plus*
  **Metronidazole **500 mg 2 times/day orally for 7 days
  * For persons weighing ≥150 kg, 1 g of ceftriaxone should be administered.
**Recommended Regimen for Adolescent and Adult Male Sexual Assault Survivors**
  **Ceftriaxone** 500 mg* IM in a single dose
*plus*
  **Doxycycline **100 mg 2 times/day orally for 7 days
  * For persons weighing ≥150 kg, 1 g of ceftriaxone should be administered.

Clinicians should counsel persons regarding the possible benefits and toxicities associated with these treatment regimens; gastrointestinal side effects can occur with this combination. The efficacy of these regimens in preventing infections after sexual assault has not been evaluated. For those requiring alternative treatments, refer to the specific sections in this report relevant to the specific organisms.

#### Other Management Considerations

At the initial examination and, if indicated, at follow-up examinations, patients should be counseled regarding symptoms of STIs and the need for immediate examination if symptoms occur. Further, they should be instructed to abstain from sexual intercourse until STI prophylactic treatment is completed.

#### Follow-Up

After the initial postassault examination, follow-up examinations provide an opportunity to detect new infections acquired during or after the assault, complete hepatitis B and HPV vaccinations, if indicated, complete counseling and treatment for other STIs, and monitor side effects and adherence to PEP, if prescribed. If initial testing was performed, follow-up evaluation should be conducted in <1 week to ensure that results of positive tests can be discussed promptly with the survivor, treatment is provided if not administered at the initial visit, and any follow-up for infections can be arranged. If initial tests are negative and treatment was not provided, examination for STIs can be repeated 1–2 weeks after the assault; repeat testing detects infectious organisms that might not have reached sufficient concentrations to produce positive test results at the time of initial examination. For survivors who are treated during the initial visit, regardless of whether testing was performed, posttreatment testing should be conducted only if the person reports having symptoms. If initial test results were negative and infection in the assailant cannot be ruled out, serologic tests for syphilis can be repeated at 4–6 weeks and 3 months; HIV testing can be repeated at 6 weeks and at 3 months by using methods to identify acute HIV infection.

#### Risk for Acquiring HIV Infection

HIV seroconversion has occurred among persons whose only known risk factor was sexual assault or sexual abuse; however, the frequency of this occurrence likely is low (*1428*,*1429*). In consensual sex, the per-act risk for HIV transmission from vaginal intercourse is 0.08%, and for receptive anal intercourse, 1.38% (*192*). The per-act risk for HIV transmission from oral sex is substantially lower. Specific circumstances of an assault (e.g., bleeding, which often accompanies trauma) might increase risk for HIV transmission in cases involving vaginal, anal, or oral penetration. Site of exposure to ejaculate, viral load in ejaculate, and the presence of an STI or genital lesions in the assailant or survivor also might increase risk for HIV acquisition.

PEP with a 28-day course of zidovudine was associated with an 81% reduction in risk for acquiring HIV in a study of health care workers who had percutaneous exposures to HIV-infected blood (*1430*). On the basis of these results and results from animal studies, PEP has been recommended for health care workers who have occupational exposures to HIV (*1431*). These findings have been extrapolated to nonoccupational injecting drug and sexual HIV exposures, including sexual assault. The possibility of HIV exposure from the assault should be assessed at the initial examination; survivors determined to be at risk for acquiring HIV should be informed about the possible benefit of PEP in preventing HIV infection. Initiation of PEP as soon as possible after the exposure increases the likelihood of prophylactic benefit.

Multiple factors affect the medical recommendation for PEP and affect the assault survivor’s acceptance of that recommendation. These factors include the likelihood of the assailant having HIV, any exposure characteristics that might increase the risk for HIV transmission, the time elapsed after the event, and the potential benefits and risks associated with PEP (*1431*). Determination of the assailant’s HIV status at the time of the postassault examination is usually not possible. Therefore, health care providers should assess any available information concerning the characteristics and HIV risk behaviors of the assailant (e.g., being an MSM or using injecting drugs), local epidemiology of HIV/AIDS, and exposure characteristics of the assault. When an assailant’s HIV status is unknown, determinations about risk for HIV transmission to the survivor should be based on whether vaginal or anal penetration occurred; whether ejaculation occurred on mucous membranes; whether multiple assailants were involved; whether mucosal lesions were present in the assailant or survivor; and any other characteristics of the assault, survivor, or assailant that might increase risk for HIV transmission.

If PEP is offered, the following information should be discussed with the survivor: the necessity of early initiation of PEP to optimize potential benefits (i.e., as soon as possible after and <72 hours after the assault), the importance of close follow-up, the benefit of adherence to recommended dosing, and potential adverse effects of antiretroviral medications. Providers should emphasize that severe adverse effects are rare from PEP (*1431*–*1435*). Clinical management of the survivor should be implemented according to the HIV PEP guidelines and in collaboration with specialists (*1436*). Health care providers should provide an initial course of 3–7 days of medication (i.e., a starter pack) with a prescription for the remainder of the course, or, if starter packs are unavailable, they should provide a prescription for an entire 28-day course. Provision of the entire 28-day PEP medication supply at the initial visit has been reported to increase likelihood of adherence, especially when patients have difficulty returning for multiple follow-up visits (*1437*). Routinely providing starter packs or the entire 28-day course requires that health care providers stock PEP drugs in their practice setting or have an established agreement with a pharmacy to stock, package, and urgently dispense PEP drugs with required administration instructions. Uninsured patients or those with high copayments can be enrolled in a patient-assistance program to ensure access to PEP medications. An early follow-up visit should be scheduled at which health care providers can discuss the results of HIV and STI testing, provide additional counseling and support, provide indicated vaccines not administered at the initial evaluation, assess medication side effects and adherence, or provide an altered PEP medication regimen if indicated by side effects or laboratory test results.

#### Recommendations for Postexposure HIV Risk Assessment of Adolescents and Adults <72 Hours After Sexual Assault

Health care providers should do the following:

- Assess risk for HIV infection in the assailant, and test that person for HIV whenever possible.
- Use the algorithm to evaluate the survivor for the need for HIV PEP (Figure) (*1436*).

**FIGURE:**
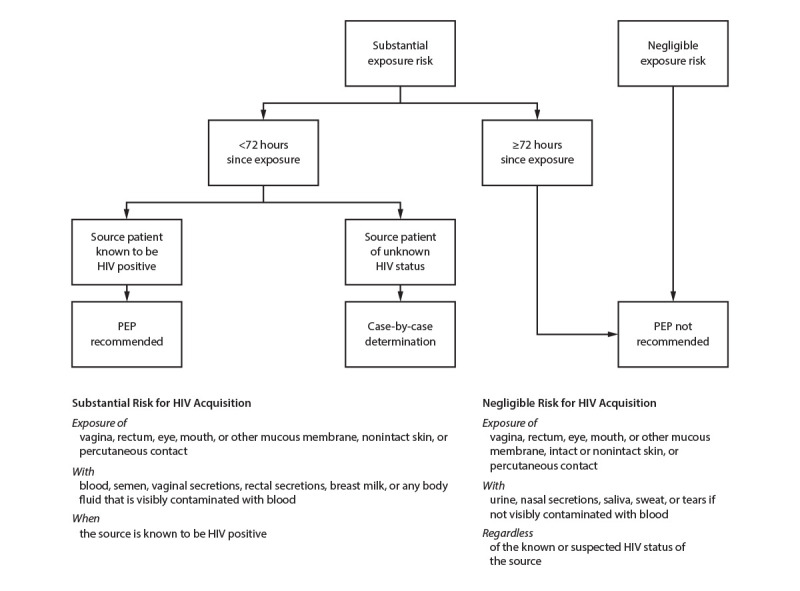
Algorithm to evaluate the need for nonoccupational HIV postexposure prophylaxis among adult and adolescent survivors of sexual assault **Abbreviation: **PEP = postexposure prophylaxis.

**Source: **Adapted from Announcement: updated guidelines for antiretroviral postexposure prophylaxis after sexual, injection-drug use, or other nonoccupational exposure to HIV—United States, 2016. MMWR Morb Mortal Wkly Rep 2016;65:458.
- Consult with a specialist in HIV treatment if PEP is being considered.
- If the survivor appears to be at risk for acquiring HIV from the assault, discuss PEP, including benefits and risks.
- If the survivor chooses to start PEP, provide an initial course of 3–7 days of medication (i.e., a starter pack) with a prescription for the remainder of the course or provide a prescription for an entire 28-day course. Schedule an early follow-up visit to discuss test results and provide additional counseling (*1438*).
- If PEP is started, obtain serum creatinine, AST, and alanine aminotransferase at baseline.
- Perform an HIV antibody test at original assessment; repeat at 6 weeks and 3 months.
- Counsel the survivor regarding ongoing risk for HIV acquisition and about HIV PrEP, and provide referrals to a PrEP provider.

Assistance with PEP-related decisions can be obtained by calling the National Clinician’s Post Exposure Prophylaxis Hotline (PEP Line) (telephone: 888-448-4911).

### Sexual Assault or Abuse of Children

These guidelines are limited to the identification and treatment of STIs in prepubertal children. Management of the psychosocial or legal aspects of the sexual assault or abuse of children is beyond the scope of these guidelines.

Identification of STIs in children past the neonatal period strongly indicates sexual abuse (*1438*). The importance of identifying a sexually transmitted organism for such children as evidence of possible child sexual abuse varies by pathogen. Postnatally acquired gonorrhea, syphilis, chlamydia, and *T. vaginalis* infection and nontransfusion, nonperinatally acquired HIV infection are indicative of sexual abuse. Sexual abuse should be suspected when anogenital herpes or anogenital warts are diagnosed. Investigation of sexual abuse among children who have an infection that might have been transmitted sexually should be conducted in compliance with recommendations by clinicians who have experience and training in all elements of the evaluation of child abuse, neglect, and assault. The social significance of an infection that might have been acquired sexually varies by the specific organism, as does the threshold for reporting suspected child sexual abuse (Table 8). When any STI has been diagnosed in a child, efforts should be made in consultation with a specialist to evaluate the possibility of sexual abuse, including conducting a history and physical examination for evidence of abuse and diagnostic testing for other commonly occurring STIs (*1439*–*1441*).

**TABLE 8:** Implications of commonly encountered sexually transmitted or sexually associated infections for diagnosis and reporting of sexual abuse among infants and prepubertal children

Infection		Evidence for sexual abuse	Recommended action
Gonorrhea*		Diagnostic	Report^†^
Syphilis*		Diagnostic	Report^†^
HIV^§^		Diagnostic	Report^†^
Chlamydia trachomatis*		Diagnostic	Report^†^
Trichomonas vaginalis*		Diagnostic	Report^†^
Anogenital herpes		Suspicious	Consider report^†,¶^
Condylomata acuminata (anogenital warts)*		Suspicious	Consider report^†,¶,^**
Anogenital molluscum contagiosum	Inconclusive		Medical follow-up
Bacterial vaginosis	Inconclusive		Medical follow-up

**Sources:** Adapted from Kellogg N; American Academy of Pediatrics Committee on Child Abuse and Neglect. The evaluation of child abuse in children. Pediatrics 2005;16:506–12; Adams JA, Farst KJ, Kellogg ND. Interpretation of medical findings in suspected child abuse: an update for 2018. J Pediatr Adolesc Gynecol 2018;31:225–31.

* If unlikely to have been perinatally acquired and vertical transmission, which is rare, is excluded.

^† ^Reports should be made to the local or state agency mandated to receive reports of suspected child abuse or neglect.

^§^ If unlikely to have been acquired perinatally or through transfusion.

^¶^ Unless a clear history of autoinoculation exists.

** Report if evidence exists to suspect abuse, including history, physical examination, or other identified infections. Lesions appearing for the first time in a child aged >5 years are more likely to have been caused by sexual transmission.

The general rule that STIs beyond the neonatal period are evidence of sexual abuse has exceptions. For example, genital infection with *T. vaginalis* (*1442*) or rectal or genital infection with *C. trachomatis* among young children might be the result of perinatally acquired infection and has, in certain cases of chlamydial infection, persisted for as long as 2–3 years (*1443*–*1445*), although perinatal chlamydial infection is now uncommon because of prenatal screening and treatment of pregnant women. Genital warts have been diagnosed among children who have been sexually abused (*1426*) but also among children who have no other evidence of sexual abuse (*1446*,*1447*); lesions appearing for the first time in a child aged >5 years are more likely to have been caused by sexual transmission (*1448*). BV has been diagnosed among children who have been abused but its presence alone does not prove sexual abuse. The majority of HBV infections among children result from household exposure to persons who have chronic HBV infection rather than sexual abuse.

#### Reporting

All U.S. states and territories have laws that require reporting of child abuse. Although the exact requirements differ by state or territory, if a health care provider has reasonable cause to suspect child abuse, a report must be made (*1448*). Health care providers should contact their state or local child protection service agency regarding child abuse reporting requirements.

#### Evaluating Children for STIs

Evaluating children for sexual assault or abuse should be conducted in a manner designed to minimize pain and trauma to the child. Examinations and collection of vaginal specimens in prepubertal girls can be extremely uncomfortable and should be performed by an experienced clinician to avoid psychological and physical trauma to the child. The decision to obtain genital or other specimens from a child to evaluate for STIs should be made on an individual basis. However, children who received a diagnosis of one STI should be screened for other STIs. History and reported type of sexual contact might not be a reliable indicator, and urogenital, pharyngeal, and rectal testing should be considered for preverbal children and children who cannot verbalize details of the assault (*1438*,*1449*). Factors that should lead the physician to consider testing for STIs include the following (*1449*):

- The child has experienced penetration or has evidence of recent or healed penetrative injury to the genitals, anus, or oropharynx.
- The child has been abused by a stranger.
- The child has been abused by an assailant known to be infected with an STI or at high risk for STIs (e.g., injecting drug user, MSM, person with multiple sex partners, or person with a history of STIs).
- The child has a sibling, other relative, or another person in the household with an STI.
- The child lives in an area with a high rate of STIs in the community.
- The child has signs or symptoms of STIs (e.g., vaginal discharge or pain, genital itching or odor, urinary symptoms, or genital lesions or ulcers).
- The child or parent requests STI testing.
- The child is unable to verbalize details of the assault.

If a child has symptoms, signs, or evidence of an infection that might be sexually transmitted, the child should be tested for common STIs before initiation of any treatment that might interfere with diagnosing other STIs. Because of the legal and psychosocial consequences of a false-positive diagnosis, only tests with high specificities should be used. The potential benefit to the child of a reliable STI diagnosis justifies deferring presumptive treatment until specimens for highly specific tests are obtained by providers with experience in evaluating sexually abused and assaulted children.

Evaluations should be performed on a case-by-case basis, according to history of assault or abuse and in a manner that minimizes the possibility for psychological trauma and social stigma. If the initial exposure was recent, the infectious organisms acquired through the exposure might not have produced sufficient concentrations to result in positive test results or examination findings (*1450*). Alternatively, positive test results after a recent exposure might represent the assailant’s secretions (but would nonetheless be an indication for treatment of the child). A second visit approximately 2–6 weeks after the most recent sexual exposure should be scheduled to include a repeat physical examination and collection of additional specimens to identify any infection that might not have been detected at the time of initial evaluation. A single evaluation might be sufficient if the child was abused for an extended period and if a substantial amount of time elapsed between the last suspected episode of abuse and the medical evaluation. Compliance with follow-up appointments might be improved when law enforcement personnel or child protective services are involved.

##### Initial Examination

Visual inspection of the genital, perianal, and oral areas for genital discharge, odor, bleeding, irritation, warts, and ulcerative lesions should be performed during initial examination. The clinical manifestations of certain STIs are different for children than for adults. For example, typical vesicular lesions might be absent even in the presence of HSV infection. The following should be performed during the initial examination, if STI testing is indicated:

- Testing for *N. gonorrhoeae* and *C. trachomatis* can be performed from specimens collected from the pharynx and rectum, as well as the vagina for girls and urine for boys. Cervical specimens are not recommended for prepubertal girls. For boys with a urethral discharge, a meatal specimen discharge is an adequate substitute for an intraurethral swab specimen. Culture or NAAT can be used to test for *N. gonorrhoeae *and *C. trachomatis.* Although data regarding NAAT for children are more limited and performance is test dependent (*553*), no evidence demonstrates that performance of NAAT for detection of *N. gonorrhoeae* or *C. trachomatis* among children differs from that among adults. Only FDA-cleared NAAT assays should be used. Consultation with an expert is necessary before using NAAT in this context, both to minimize the possibility of cross-reaction with nongonococcal *Neisseria* species and other commensals (e.g., *N. meningitidis*, *N. sicca*, *N. lactamica*, *N. cinerea*, or *M. catarrhalis*) and to ensure correct interpretation of results. Because of the implications of a diagnosis of *N. gonorrhoeae* or *C. trachomatis* infection in a child, only CLIA-validated, FDA-cleared NAATs should be used (*837*). If culture for the isolation of *N. gonorrhoeae* or *C. trachomatis* is performed, only standard culture procedures should be followed. Specimens from the vagina, urethra, pharynx, or rectum should be streaked onto selective media for isolation of *N. gonorrhoeae*, and all presumptive isolates of *N. gonorrhoeae* should be identified definitively by at least two tests that involve different approaches (e.g., biochemical, enzyme substrate, or molecular probes). Gram stains are inadequate for evaluating prepubertal children for gonorrhea and should not be used to diagnose or exclude gonorrhea. Specimens (either NAAT or culture, including any isolates) obtained before treatment should be preserved for further validation if needed. When a specimen is positive, the result should be confirmed either by retesting the original specimen or obtaining another. Because of the overall low prevalence of *N. gonorrhoeae* and *C. trachomatis* among children, false-positive results can occur, and all specimens that are initially positive should be confirmed.
- Testing for *T. vaginalis* should not be limited to girls with vaginal discharge if other indications for vaginal testing exist because evidence indicates that asymptomatic sexually abused children might be infected with *T. vaginalis* and might benefit from treatment (*1451*,*1452*). NAAT can be used as an alternative or in addition to culture and wet mount, especially in settings where culture and wet mount of vaginal swab specimens are not obtainable. Data regarding use of NAATs for detection of *T. vaginalis* among children are limited; however, no evidence indicates that performance of NAAT for detection of *T. vaginalis* for children would differ from that for adults. Consultation with an expert is necessary before using NAAT in this context to ensure correct interpretation of results. Because of the implications of a diagnosis of *T. vaginalis* infection in a child, only CLIA-validated, FDA-cleared NAATs should be used (*837*). POC tests for *T. vaginalis* have not been validated for prepubertal children and should not be used. In the case of a positive specimen, the result should be confirmed either by retesting the original specimen or obtaining another. Because of the overall low prevalence of *T. vaginalis* among children, false-positive results can occur, and all specimens that are initially positive should be confirmed.
- HSV can be indicative of sexual abuse; therefore, specimens should be obtained from all vesicular or ulcerative genital or perianal lesions and sent for NAAT or viral culture.
- Wet mount can be used for a vaginal swab specimen for BV if discharge is present.
- Collection of serum samples should be evaluated, preserved for subsequent analysis, and used as a baseline for comparison with follow-up serologic tests. Sera can be tested for antibodies to *T. pallidum*, HIV, and HBV. Decisions regarding the infectious agents for which to perform serologic tests should be made on a case-by-case basis.

#### Treatment

The risk for a child acquiring an STI as a result of sexual abuse or assault has not been well studied. Presumptive treatment for children who have been sexually assaulted or abused is not recommended because the incidence of most STIs among children is low after abuse or assault, prepubertal girls appear to be at lower risk for ascending infection than adolescent or adult women, and regular follow-up of children usually can be ensured. However, certain children or their parent or guardian might be concerned about the possibility of infection with an STI, even if the health care provider has perceived the risk to be low. Such concerns might be an indication for presumptive treatment in certain settings and might be considered after all relevant specimens for diagnostic tests have been collected.

#### Other Management Considerations

Children who are survivors of sexual assault or abuse are at increased risk for future unsafe sexual practices that have been linked to higher risk for HPV acquisition (*1426*,*1453*) and are more likely to engage in these behaviors at an earlier age; therefore, ACIP recommends vaccination of these children at age ≥9 years if they have not initiated or completed HPV vaccination (see Human Papillomavirus Infections, Prevention) (https://www.cdc.gov/vaccines/hcp/acip-recs/vacc-specific/hpv.html). Although HPV vaccine will not protect against progression of infection already acquired or promote clearance of the infection, the vaccine protects against HPV types not yet acquired.

#### Follow-Up

If no infections were identified at the initial examination after the last suspected sexual exposure, and if this exposure was recent, a follow-up evaluation approximately 2 weeks after the last exposure can be considered. Likewise, if no physical examination or diagnostic testing was performed at the initial visit, a complete examination can be scheduled approximately 2 weeks after the last exposure to identify any evidence of STIs. In circumstances in which transmission of syphilis, HIV, HBV, or HPV is a concern but baseline tests for syphilis, HIV, and HBV are negative and examinations for genital warts are negative, follow-up serologic testing and examination approximately 6 weeks and <3 months after the last suspected sexual exposure is recommended to allow time for antibodies to develop and signs of infection to appear. In addition, results of HBsAg testing should be interpreted carefully because HBV can be transmitted nonsexually. Decisions regarding which tests should be performed should be made on a case-by-case basis.

#### Risk for Acquiring HIV Infection

HIV has been reported among children for whom sexual abuse was the only known risk factor. Serologic testing for HIV should be considered for sexually abused children. The decision to test for HIV should involve the family, if possible, and be made on a case-by-case basis depending on the likelihood of infection in the assailant (*1448*,*1454*). Although data are insufficient concerning the efficacy of PEP among children, treatment is well tolerated by infants and children with and without HIV, and children have a minimal risk for serious adverse reactions because of the short period recommended for prophylaxis (*1455*).

#### Recommendations for Postexposure HIV Risk Assessment of Children <72 Hours After Sexual Assault

Providers should do the following:

- Review local HIV epidemiology, assess risk for HIV in the assailant, and test for HIV.
- Evaluate the circumstances of the assault or abuse that might affect risk for HIV transmission.
- Perform HIV antigen or antibody testing (or antibody testing, if antigen or antibody testing is unavailable) during the original assessment and again at follow-up visits, in accordance with CDC guidelines (https://stacks.cdc.gov/view/cdc/38856). In considering whether to offer PEP, health care providers should consider whether the child can be treated soon after the sexual exposure (i.e., <72 hours), the likelihood that the assailant has HIV infection, and the likelihood of high compliance with the prophylactic regimen (*1436*). Potential benefit of treating a sexually abused child should be weighed against the risk for adverse reactions.
- Consult with a provider specializing in evaluating or treating children with HIV infection to determine age-appropriate dosing and regimens and baseline laboratory testing, if PEP is being considered.
- Discuss PEP with the caregivers, including its toxicity, unknown efficacy, and possible benefits, for children determined to be at risk for HIV transmission from the assault or abuse.
- Provided adequate doses of medication, if PEP is begun, to last until the follow-up visit 3–7 days after the initial assessment, at which time the child should be reevaluated and tolerance of medication assessed (*139*).

## Footnotes

* Regardless of condom use during exposure.

† Direct hyperbilirubinemia is direct bilirubin level >2 mg/dL (34 umol/L) or 20% of the total bilirubin level.

§ One dilution is within the test performance of nontreponemal tests and is not a significant change.

¶ The absence of a fourfold or greater titer for a neonate does not exclude congenital syphilis.

** Interpretation of CSF test results requires a nontraumatic lumbar puncture (i.e., a CSF sample that is not contaminated with blood). CSF test results obtained during the neonatal period can be difficult to interpret; normal values differ by gestational age and are higher among preterm infants. Studies indicate that 95% of healthy neonates have values of ≤16–19 WBCs/mm^3^ or protein levels of ≤115–118 mg/dL on CSF examination. During the second month of life, 95% of healthy infants have ≤9–11 WBCs/mm^3^ or protein levels of ≤89–91 mg/dL. Lower values (i.e., 5 WBCs/mm^3^ and protein level of 40 mg/dL) might be considered the upper limits of normal for older infants. Other causes of elevated values should be considered when an infant is being evaluated for congenital syphilis (**Sources:** Kestenbaum LA, Ebberson J, Zorc JJ, Hodinka RL, Shah SS. Defining cerebrospinal fluid white blood cell count reference values in neonates and young infants. Pediatrics 2010;125:257–64; Shah SS, Ebberson J, Kestenbaum LA, Hodinka RL, Zorc JJ. Age-specific reference values for cerebrospinal fluid protein concentration in neonates and young infants. J Hosp Med 2011;6:22–7; Thomson J, Sucharew H, Cruz AT, et al.; Pediatric Emergency Medicine Collaborative Research Committee [PEM CRC] HSV Study Group. Cerebrospinal fluid reference values for young infants undergoing lumbar puncture. Pediatrics 2018;141:e20173405.)

†† A women treated with a regimen other than recommended in these guidelines should be considered untreated.

§§ For urethral microscopy, the cutoff for diagnosing urethritis is ≥2 WBCs/HPF (**Sources:** Rietmeijer CA, Mettenbrink CJ. Recalibrating the Gram stain diagnosis of male urethritis in the era of nucleic acid amplification testing. Sex Transm Dis 2012;39:18–20; Rietmeijer CA, Mettenbrink CJ. The diagnosis of nongonococcal urethritis in men: can there be a universal standard? Sex Transm Dis 2017;44:195–6). An additional evaluation supported this cutoff by demonstrating NGU sensitivity of 92.6% for cutoff of ≥2 versus 55.6% sensitivity for cutoff of ≥5 (**Source:** Sarier M, Sepin N, Duman I, et al. Microscopy of Gram-stained urethral smear in the diagnosis of urethritis: which threshold value should be selected? Andrologia 2018;50:e13143). Diagnostic cutoffs for 369 symptomatic and asymptomatic heterosexual men seeking STI care in Seattle revealed a maximal sensitivity and specificity achieved with a cutoff of ≥5 WBCs/HPF. Using a lower cutoff of ≥2 WBCs/HPF would miss 13% of *C. trachomatis* and *M. genitalium* and overtreat 45% of persons who have negative tests (**Source:** Leipertz G, Chambers L, Lowens S, et al. P796 Reassessing the Gram stain smear [GSS] polymorphonuclear leukocyte [PMN] cutoff for diagnosing non-gonococcal urethritis [NGU]. Sex Transm Infect 2019;95[Suppl 1]:A339). Another study discussed that the WBC/HPF cutoff value should discriminate on the basis of the prevalence of chlamydia, mycoplasma, and gonorrhea among a clinic population (**Source:** Moi H, Hartgill U, Skullerud KH, Reponen EJ, Syvertsen L, Moghaddam A. Microscopy of stained urethral smear in male urethritis: which cutoff should be used? Sex Transm Dis 2017;44:189–94).

## References

[1] Workowski KA, Bolan GA; CDC. Sexually transmitted diseases treatment guidelines, 2015. MMWR Recomm Rep 2015;64(No. RR-3). 26042815 PMC5885289

[2] Barrow RY, Ahmed F, Bolan GA, Workowski KA. Recommendations for providing quality sexually transmitted diseases clinical services, 2020. MMWR Recomm Rep 2020;68(No. RR-5). 10.15585/mmwr.rr6805a1 31899459 PMC6950496

[3] CDC. A guide to taking a sexual history. Atlanta, GA: US Department of Health and Human Services, CDC. https://www.cdc.gov/std/treatment/sexualhistory.pdf

[4] Henderson JT, Senger CA, Henninger M, Bean SI, Redmond N, O’Connor EA. Behavioral counseling interventions to prevent sexually transmitted infections: updated evidence report and systematic review for the US Preventive Services Task Force. JAMA 2020;324:682–99. 10.1001/jama.2020.1037132809007

[5] Kamb ML, Fishbein M, Douglas JM Jr, ; Project RESPECT Study Group. Efficacy of risk-reduction counseling to prevent human immunodeficiency virus and sexually transmitted diseases: a randomized controlled trial. JAMA 1998;280:1161–7. 10.1001/jama.280.13.1161 9777816

[6] Metsch LR, Feaster DJ, Gooden L, Effect of risk-reduction counseling with rapid HIV testing on risk of acquiring sexually transmitted infections: the AWARE randomized clinical trial. JAMA 2013;310:1701–10. 10.1001/jama.2013.280034 24150466 PMC4110051

[7] Brookmeyer KA, Hogben M, Kinsey J. The role of behavioral counseling in sexually transmitted disease prevention program settings. Sex Transm Dis 2016;43(Suppl 1):S102–12. 10.1097/OLQ.0000000000000327 26779681 PMC4717909

[8] Patel P, Bush T, Mayer K, ; SUN Study Investigators. Routine brief risk-reduction counseling with biannual STD testing reduces STD incidence among HIV-infected men who have sex with men in care. Sex Transm Dis 2012;39:470–4. 10.1097/OLQ.0b013e31824b3110 22592834 PMC6195212

[9] Warner L, Klausner JD, Rietmeijer CA, ; Safe in the City Study Group. Effect of a brief video intervention on incident infection among patients attending sexually transmitted disease clinics. PLoS Med 2008;5:e135. 10.1371/journal.pmed.0050135 18578564 PMC2504047

[10] Mustanski B, Parsons JT, Sullivan PS, Madkins K, Rosenberg E, Swann G. Biomedical and behavioral outcomes of Keep It Up!: an ehealth HIV prevention program RCT. Am J Prev Med 2018;55:151–8. 10.1016/j.amepre.2018.04.026 29937115 PMC6314292

[11] Meites E, Szilagyi PG, Chesson HW, Unger ER, Romero JR, Markowitz LE. Human papillomavirus vaccination for adults: updated recommendations of the Advisory Committee on Immunization Practices. MMWR Morb Mortal Wkly Rep 2019;68:698–702. 10.15585/mmwr.mm6832a3 31415491 PMC6818701

[12] Schillie S, Vellozzi C, Reingold A, Prevention of hepatitis B virus infection in the United States: recommendations of the Advisory Committee on Immunization Practices. MMWR Recomm Rep 2018;67(No. RR-1). 10.15585/mmwr.rr6701a1 29939980 PMC5837403

[13] Doshani M, Weng M, Moore KL, Romero JR, Nelson NP. Recommendations of the Advisory Committee on Immunization Practices for use of hepatitis A vaccine for persons experiencing homelessness. MMWR Morb Mortal Wkly Rep 2019;68:153–6. 10.15585/mmwr.mm6806a6 30763295 PMC6375653

[14] Weller S, Davis K. Condom effectiveness in reducing heterosexual HIV transmission. Cochrane Database Syst Rev 2002;(1):CD003255. 10.1002/14651858.CD00325511869658

[15] Giannou FK, Tsiara CG, Nikolopoulos GK, Condom effectiveness in reducing heterosexual HIV transmission: a systematic review and meta-analysis of studies on HIV serodiscordant couples. Expert Rev Pharmacoecon Outcomes Res 2016;16:489–99. 10.1586/14737167.2016.1102635 26488070

[16] Smith DK, Herbst JH, Zhang X, Rose CE. Condom effectiveness for HIV prevention by consistency of use among men who have sex with men in the United States. J Acquir Immune Defic Syndr 2015;68:337–44. 10.1097/QAI.0000000000000461 25469526

[17] Johnson WD, O’Leary A, Flores SA. Per-partner condom effectiveness against HIV for men who have sex with men. AIDS 2018;32:1499–505. 10.1097/QAD.0000000000001832 29794493

[18] Crosby RA, Charnigo RA, Weathers C, Caliendo AM, Shrier LA. Condom effectiveness against non-viral sexually transmitted infections: a prospective study using electronic daily diaries. Sex Transm Infect 2012;88:484–9. 10.1136/sextrans-2012-050618 23002192 PMC3502658

[19] Holmes KK, Levine R, Weaver M. Effectiveness of condoms in preventing sexually transmitted infections. Bull World Health Organ 2004;82:454–61. 15356939 PMC2622864

[20] Warner L, Stone KM, Macaluso M, Buehler JW, Austin HD. Condom use and risk of gonorrhea and chlamydia: a systematic review of design and measurement factors assessed in epidemiologic studies. Sex Transm Dis 2006;33:36–51. 10.1097/01.olq.0000187908.42622.fd 16385221

[21] Bernabe-Ortiz A, Carcamo CP, Scott JD, Hughes JP, Garcia PJ, Holmes KK. HBV infection in relation to consistent condom use: a population-based study in Peru. PLoS One 2011;6:e24721. 10.1371/journal.pone.0024721 21931828 PMC3172281

[22] Ness RB, Hillier SL, Kip KE, Bacterial vaginosis and risk of pelvic inflammatory disease. Obstet Gynecol 2004;104:761–9. 10.1097/01.AOG.0000139512.37582.17 15458899

[23] Martin IE, Gu W, Yang Y, Tsang RS. Macrolide resistance and molecular types of *Treponema pallidum* causing primary syphilis in Shanghai, China. Clin Infect Dis 2009;49:515–21. 10.1086/600878 19583516

[24] Winer RL, Hughes JP, Feng Q, Condom use and the risk of genital human papillomavirus infection in young women. N Engl J Med 2006;354:2645–54. 10.1056/NEJMoa053284 16790697

[25] Bleeker MC, Hogewoning CJ, Voorhorst FJ, Condom use promotes regression of human papillomavirus-associated penile lesions in male sexual partners of women with cervical intraepithelial neoplasia. Int J Cancer 2003;107:804–10. 10.1002/ijc.11473 14566831

[26] Hogewoning CJ, Bleeker MC, van den Brule AJ, Condom use promotes regression of cervical intraepithelial neoplasia and clearance of human papillomavirus: a randomized clinical trial. Int J Cancer 2003;107:811–6. 10.1002/ijc.11474 14566832

[27] Koss CA, Dunne EF, Warner L. A systematic review of epidemiologic studies assessing condom use and risk of syphilis. Sex Transm Dis 2009;36:401–5. 10.1097/OLQ.0b013e3181a396eb 19455075

[28] Hernández-Romieu AC, Siegler AJ, Sullivan PS, Crosby R, Rosenberg ES. How often do condoms fail? A cross-sectional study exploring incomplete use of condoms, condom failures and other condom problems among black and white MSM in southern U.S.A. Sex Transm Infect 2014;90:602–7. 10.1136/sextrans-2014-051581 25080511 PMC4408017

[29] D’Anna LH, Margolis AD, Warner L, ; Safe City Study Group. Condom use problems during anal sex among men who have sex with men (MSM): findings from the Safe in the City study. AIDS Care 2012;24:1028–38. 10.1080/09540121.2012.668285 22519680 PMC3389178

[30] Steiner MJ, Cates W Jr, Warner L. The real problem with male condoms is nonuse. Sex Transm Dis 1999;26:459–62. 10.1097/00007435-199909000-00007 10494937

[31] Kowal D, Hatcher RA, Nelson AL, et al., eds. Contraceptive technology. 21st ed. Atlanta, GA: Managing Contraception; 2017.

[32] Gallo MF, Kilbourne-Brook M, Coffey PS. A review of the effectiveness and acceptability of the female condom for dual protection. Sex Health 2012;9:18–26. 10.1071/SH11037 22348629

[33] Mantell JE, Kelvin EA, Exner TM, Hoffman S, Needham S, Stein ZA. Anal use of the female condom: does uncertainty justify provider inaction? AIDS Care 2009;21:1185–94. 10.1080/09540120902730005 20024779 PMC2797077

[34] Rosenberg MJ, Davidson AJ, Chen JH, Judson FN, Douglas JM. Barrier contraceptives and sexually transmitted diseases in women: a comparison of female-dependent methods and condoms. Am J Public Health 1992;82:669–74. 10.2105/AJPH.82.5.669 1566944 PMC1694156

[35] de Bruyn G, Shiboski S, van der Straten A, ; MIRA Team. The effect of the vaginal diaphragm and lubricant gel on acquisition of HSV-2. Sex Transm Infect 2011;87:301–5. 10.1136/sti.2010.047142 21447515

[36] Ramjee G, van der Straten A, Chipato T, ; MIRA team. The diaphragm and lubricant gel for prevention of cervical sexually transmitted infections: results of a randomized controlled trial. PLoS One 2008;3:e3488. 10.1371/journal.pone.0003488 18941533 PMC2567030

[37] Lusti-Narasimhan M, Merialdi M, Holt B. Multipurpose prevention technologies: maximising positive synergies. BJOG 2014;121:251. 10.1111/1471-0528.12606 24393212

[38] Ahmed K, Baeten JM, Beksinska M, ; Evidence for Contraceptive Options and HIV Outcomes (ECHO) Trial Consortium. HIV incidence among women using intramuscular depot medroxyprogesterone acetate, a copper intrauterine device, or a levonorgestrel implant for contraception: a randomised, multicentre, open-label trial. Lancet 2019;394:303–13. 10.1016/S0140-6736(19)31288-7 31204114 PMC6675739

[39] Young Holt B, Dellplain L, Creinin MD, Peine KJ, Romano J, Hemmerling A. A strategic action framework for multipurpose prevention technologies combining contraceptive hormones and antiretroviral drugs to prevent pregnancy and HIV. Eur J Contracept Reprod Health Care 2018;23:326–34. 10.1080/13625187.2018.1508650 30247084

[40] Wilkinson D, Tholandi M, Ramjee G, Rutherford GW. Nonoxynol-9 spermicide for prevention of vaginally acquired HIV and other sexually transmitted infections: systematic review and meta-analysis of randomised controlled trials including more than 5000 women. Lancet Infect Dis 2002;2:613–7. 10.1016/S1473-3099(02)00396-1 12383611

[41] McCormack S, Ramjee G, Kamali A, PRO2000 vaginal gel for prevention of HIV-1 infection (Microbicides Development Programme 301): a phase 3, randomised, double-blind, parallel-group trial. Lancet 2010;376:1329–37. 10.1016/S0140-6736(10)61086-0 20851460 PMC2956883

[42] Skoler-Karpoff S, Ramjee G, Ahmed K, Efficacy of Carraguard for prevention of HIV infection in women in South Africa: a randomised, double-blind, placebo-controlled trial. Lancet 2008;372:1977–87. 10.1016/S0140-6736(08)61842-5 19059048

[43] Van Damme L, Govinden R, Mirembe FM, ; CS Study Group. Lack of effectiveness of cellulose sulfate gel for the prevention of vaginal HIV transmission. N Engl J Med 2008;359:463–72. 10.1056/NEJMoa0707957 18669425

[44] Feldblum PJ, Adeiga A, Bakare R, SAVVY vaginal gel (C31G) for prevention of HIV infection: a randomized controlled trial in Nigeria. PLoS One 2008;3:e1474. 10.1371/journal.pone.0001474 18213382 PMC2190795

[45] Cottrell ML, Kashuba AD. Topical microbicides and HIV prevention in the female genital tract. J Clin Pharmacol 2014;54:603–15. 10.1002/jcph.292 24664786 PMC4144014

[46] Abdool Karim SS, Abdool Karim Q, Kharsany ABM, ; CAPRISA 004 Trial Group. Tenofovir gel for the prevention of herpes simplex virus Type 2 infection. N Engl J Med 2015;373:530–9. 10.1056/NEJMoa1410649 26244306 PMC4562018

[47] Abdool Karim Q, Abdool Karim SS, Frohlich JA, ; CAPRISA 004 Trial Group. Effectiveness and safety of tenofovir gel, an antiretroviral microbicide, for the prevention of HIV infection in women. Science 2010;329:1168–74. Erratum in: Science 2011;333:524. 10.1126/science.1193748 20643915 PMC3001187

[48] Marrazzo JM, Ramjee G, Richardson BA, ; VOICE Study Team. Tenofovir-based preexposure prophylaxis for HIV infection among African women. N Engl J Med 2015;372:509–18. 10.1056/NEJMoa1402269 25651245 PMC4341965

[49] Delany-Moretlwe S, Lombard C, Baron D, Tenofovir 1% vaginal gel for prevention of HIV-1 infection in women in South Africa (FACTS-001): a phase 3, randomised, double-blind, placebo-controlled trial. Lancet Infect Dis 2018;18:1241–50. 10.1016/S1473-3099(18)30428-6 30507409

[50] Baeten JM, Palanee-Phillips T, Brown ER, ; MTN-020–ASPIRE Study Team. Use of a vaginal ring containing dapivirine for HIV-1 prevention in women. N Engl J Med 2016;375:2121–32. 10.1056/NEJMoa1506110 26900902 PMC4993693

[51] Nel A, van Niekerk N, Kapiga S, ; Ring Study Team. Safety and efficacy of a dapivirine vaginal ring for HIV prevention in women. N Engl J Med 2016;375:2133–43. 10.1056/NEJMoa1602046 27959766

[52] Cranston RD, Lama JR, Richardson BA, ; MTN-017 Protocol Team. MTN-017: a rectal phase 2 extended safety and acceptability study of tenofovir reduced-glycerin 1% gel. Clin Infect Dis 2017;64:614–20. 27986684 10.1093/cid/ciw832 PMC5850518

[53] Hooton TM, Roberts PL, Stamm WE. Effects of recent sexual activity and use of a diaphragm on the vaginal microflora. Clin Infect Dis 1994;19:274–8. 10.1093/clinids/19.2.274 7986899

[54] Fihn SD, Boyko EJ, Normand EH, Association between use of spermicide-coated condoms and *Escherichia coli* urinary tract infection in young women. Am J Epidemiol 1996;144:512–20. 10.1093/oxfordjournals.aje.a008958 8781467

[55] Polis CB, Curtis KM, Hannaford PC, An updated systematic review of epidemiological evidence on hormonal contraceptive methods and HIV acquisition in women. AIDS 2016;30:2665–83. 10.1097/QAD.0000000000001228 27500670 PMC5106090

[56] Kiweewa FM, Brown E, Mishra A, ; MTN-020/ASPIRE Study Team. Acquisition of sexually transmitted infections among women using a variety of contraceptive options: a prospective study among high-risk African women. J Int AIDS Soc 2019;22:e25257. 10.1002/jia2.25257 30816632 PMC6393855

[57] McCarthy KJ, Gollub EL, Ralph L, van de Wijgert J, Jones HE. Hormonal contraceptives and the acquisition of sexually transmitted infections: an updated systematic review. Sex Transm Dis 2019;46:290–6. 10.1097/OLQ.0000000000000975 30628946

[58] Curtis KM, Tepper NK, Jatlaoui TC, U.S. medical eligibility criteria for contraceptive use, 2016. MMWR Recomm Rep 2016;65(No. RR-3). 10.15585/mmwr.rr6503a1 27467196

[59] Curtis KM, Jatlaoui TC, Tepper NK, U.S. selected practice recommendations for contraceptive use, 2016. MMWR Recomm Rep 2016;65(No. RR-4). 10.15585/mmwr.rr6504a1 27467319

[60] Cleland K, Zhu H, Goldstuck N, Cheng L, Trussell J. The efficacy of intrauterine devices for emergency contraception: a systematic review of 35 years of experience. Hum Reprod 2012;27:1994–2000. 10.1093/humrep/des140 22570193 PMC3619968

[61] Shen J, Che Y, Showell E, Chen K, Cheng L. Interventions for emergency contraception. Cochrane Database Syst Rev 2019;1:CD001324. 30661244 10.1002/14651858.CD001324.pub6 PMC7055045

[62] Marcell AV, Waks AB, Rutkow L, McKenna R, Rompalo A, Hogan MT. What do we know about males and emergency contraception? A synthesis of the literature. Perspect Sex Reprod Health 2012;44:184–93. 10.1363/4418412 22958663

[63] Gray RH, Kigozi G, Serwadda D, Male circumcision for HIV prevention in men in Rakai, Uganda: a randomised trial. Lancet 2007;369:657–66. 10.1016/S0140-6736(07)60313-4 17321311

[64] Bailey RC, Moses S, Parker CB, Male circumcision for HIV prevention in young men in Kisumu, Kenya: a randomised controlled trial. Lancet 2007;369:643–56. 10.1016/S0140-6736(07)60312-2 17321310

[65] Auvert B, Taljaard D, Lagarde E, Sobngwi-Tambekou J, Sitta R, Puren A. Randomized, controlled intervention trial of male circumcision for reduction of HIV infection risk: the ANRS 1265 Trial. PLoS Med 2005;2:e298. Erratum in: PLoS Med 2006;3:298. 10.1371/journal.pmed.0020298 16231970 PMC1262556

[66] Tobian AA, Serwadda D, Quinn TC, Male circumcision for the prevention of HSV-2 and HPV infections and syphilis. N Engl J Med 2009;360:1298–309. 10.1056/NEJMoa0802556 19321868 PMC2676895

[67] Auvert B, Sobngwi-Tambekou J, Cutler E, Effect of male circumcision on the prevalence of high-risk human papillomavirus in young men: results of a randomized controlled trial conducted in Orange Farm, South Africa. J Infect Dis 2009;199:14–9. 10.1086/595566 19086814 PMC2821597

[68] Sobngwi-Tambekou J, Taljaard D, Lissouba P, Effect of HSV-2 serostatus on acquisition of HIV by young men: results of a longitudinal study in Orange Farm, South Africa. J Infect Dis 2009;199:958–64. 10.1086/597208 19220143 PMC2868899

[69] Gray R, Kigozi G, Kong X, The effectiveness of male circumcision for HIV prevention and effects on risk behaviors in a posttrial follow-up study. AIDS 2012;26:609–15. 10.1097/QAD.0b013e3283504a3f 22210632 PMC4296667

[70] Mehta SD, Moses S, Parker CB, Agot K, Maclean I, Bailey RC. Circumcision status and incident herpes simplex virus type 2 infection, genital ulcer disease, and HIV infection. AIDS 2012;26:1141–9. 10.1097/QAD.0b013e328352d116 22382150 PMC3668787

[71] World Health Organization/UNAIDS. New data on male circumcision and HIV prevention: policy and programme implications [Internet]. Geneva, Switzerland: WHO/UNAIDS Technical Consultation on Male Circumcision and HIV Prevention: Research Implications for Policy and Programming; 2007. https://www.who.int/hiv/pub/malecircumcision/research_implications/en/

[72] American Urological Association. Circumcision policy statement [Internet]. Linthicum, MD: American Urological Association; 2017. https://www.auanet.org/guidelines/guidelines/circumcision

[73] Yuan T, Fitzpatrick T, Ko NY, Circumcision to prevent HIV and other sexually transmitted infections in men who have sex with men: a systematic review and meta-analysis of global data. Lancet Glob Health 2019;7:e436–47. 10.1016/S2214-109X(18)30567-9 30879508 PMC7779827

[74] Grohskopf LA, Chillag KL, Gvetadze R, Randomized trial of clinical safety of daily oral tenofovir disoproxil fumarate among HIV-uninfected men who have sex with men in the United States. J Acquir Immune Defic Syndr 2013;64:79–86. 10.1097/QAI.0b013e31828ece33 23466649

[75] Grant RM, Lama JR, Anderson PL, ; iPrEx Study Team. Preexposure chemoprophylaxis for HIV prevention in men who have sex with men. N Engl J Med 2010;363:2587–99. 10.1056/NEJMoa1011205 21091279 PMC3079639

[76] Baeten JM, Donnell D, Ndase P, ; Partners PrEP Study Team. Antiretroviral prophylaxis for HIV prevention in heterosexual men and women. N Engl J Med 2012;367:399–410. 10.1056/NEJMoa1108524 22784037 PMC3770474

[77] Thigpen MC, Kebaabetswe PM, Paxton LA, ; TDF2 Study Group. Antiretroviral preexposure prophylaxis for heterosexual HIV transmission in Botswana. N Engl J Med 2012;367:423–34. 10.1056/NEJMoa1110711 22784038

[78] Choopanya K, Martin M, Suntharasamai P, ; Bangkok Tenofovir Study Group. Antiretroviral prophylaxis for HIV infection in injecting drug users in Bangkok, Thailand (the Bangkok Tenofovir Study): a randomised, double-blind, placebo-controlled phase 3 trial. Lancet 2013;381:2083–90. 10.1016/S0140-6736(13)61127-7 23769234

[79] Molina JM, Charreau I, Spire B, ; ANRS IPERGAY Study Group. Efficacy, safety, and effect on sexual behaviour of on-demand pre-exposure prophylaxis for HIV in men who have sex with men: an observational cohort study. Lancet HIV 2017;4:e402–10. 10.1016/S2352-3018(17)30089-9 28747274

[80] CDC. Preexposure prophylaxis for the prevention of HIV infection in the United States—2017 update: a clinical practice guideline. Atlanta, GA: US Department of Health and Human Services, CDC; 2018. https://www.cdc.gov/hiv/pdf/risk/prep/cdc-hiv-prep-guidelines-2017.pdf

[81] Jones J, Weiss K, Mermin J, Proportion of incident human immunodeficiency virus cases among men who have sex with men attributable to gonorrhea and chlamydia: a modeling analysis. Sex Transm Dis 2019;46:357–63. 10.1097/OLQ.0000000000000980 31095100 PMC6530490

[82] Pathela P, Braunstein SL, Blank S, Schillinger JA. HIV incidence among men with and those without sexually transmitted rectal infections: estimates from matching against an HIV case registry. Clin Infect Dis 2013;57:1203–9. 10.1093/cid/cit437 23800942

[83] Pathela P, Braunstein SL, Blank S, Shepard C, Schillinger JA. The high risk of an HIV diagnosis following a diagnosis of syphilis: a population-level analysis of New York City men. Clin Infect Dis 2015;61:281–7. 10.1093/cid/civ289 25870333

[84] Chou R, Evans C, Hoverman A, Preexposure prophylaxis for the prevention of HIV infection: evidence report and systematic review for the US Preventive Services Task Force. JAMA 2019;321:2214–30. 10.1001/jama.2019.2591 31184746

[85] Liu AY, Cohen SE, Vittinghoff E, Preexposure prophylaxis for HIV infection integrated with municipal- and community-based sexual health services. JAMA Intern Med 2016;176:75–84. 10.1001/jamainternmed.2015.4683 26571482 PMC5042323

[86] McCormack S, Dunn DT, Desai M, Pre-exposure prophylaxis to prevent the acquisition of HIV-1 infection (PROUD): effectiveness results from the pilot phase of a pragmatic open-label randomised trial. Lancet 2016;387:53–60. 10.1016/S0140-6736(15)00056-2 26364263 PMC4700047

[87] Volk JE, Marcus JL, Phengrasamy T, No new HIV infections with increasing use of HIV preexposure prophylaxis in a clinical practice setting. Clin Infect Dis 2015;61:1601–3. 10.1093/cid/civ778 26334052 PMC4809999

[88] Celum C, Wald A, Lingappa JR, ; Partners in Prevention HSV/HIV Transmission Study Team. Acyclovir and transmission of HIV-1 from persons infected with HIV-1 and HSV-2. N Engl J Med 2010;362:427–39. 10.1056/NEJMoa0904849 20089951 PMC2838503

[89] Celum C, Wald A, Hughes J, ; HPTN 039 Protocol Team. Effect of aciclovir on HIV-1 acquisition in herpes simplex virus 2 seropositive women and men who have sex with men: a randomised, double-blind, placebo-controlled trial. Lancet 2008;371:2109–19. 10.1016/S0140-6736(08)60920-4 18572080 PMC2650104

[90] Bolan RK, Beymer MR, Weiss RE, Flynn RP, Leibowitz AA, Klausner JD. Doxycycline prophylaxis to reduce incident syphilis among HIV-infected men who have sex with men who continue to engage in high-risk sex: a randomized, controlled pilot study. Sex Transm Dis 2015;42:98–103. 10.1097/OLQ.0000000000000216 25585069 PMC4295649

[91] Grant JS, Stafylis C, Celum C, Doxycycline prophylaxis for bacterial sexually transmitted infections. Clin Infect Dis 2020;70:1247–53. 10.1093/cid/ciz866 31504345 PMC7319058

[92] Myer L, Kuhn L, Stein ZA, Wright TC Jr, Denny L. Intravaginal practices, bacterial vaginosis, and women’s susceptibility to HIV infection: epidemiological evidence and biological mechanisms. Lancet Infect Dis 2005;5:786–94. 10.1016/S1473-3099(05)70298-X 16310150

[93] Molina JM, Charreau I, Chidiac C, ; ANRS IPERGAY Study Group. Post-exposure prophylaxis with doxycycline to prevent sexually transmitted infections in men who have sex with men: an open-label randomised substudy of the ANRS IPERGAY trial. Lancet Infect Dis 2018;18:308–17. 10.1016/S1473-3099(17)30725-9 29229440

[94] Cohen MS, Chen YQ, McCauley M, ; HPTN 052 Study Team. Prevention of HIV-1 infection with early antiretroviral therapy. N Engl J Med 2011;365:493–505. 10.1056/NEJMoa1105243 21767103 PMC3200068

[95] Rodger AJ, Cambiano V, Bruun T, ; PARTNER Study Group. Sexual activity without condoms and risk of HIV transmission in serodifferent couples when the HIV-positive partner is using suppressive antiretroviral therapy. JAMA 2016;316:171–81. 10.1001/jama.2016.5148 27404185

[96] Bavinton BR, Pinto AN, Phanuphak N, ; Opposites Attract Study Group. Viral suppression and HIV transmission in serodiscordant male couples: an international, prospective, observational, cohort study. Lancet HIV 2018;5:e438–47. 10.1016/S2352-3018(18)30132-2 30025681

[97] Rodger AJ, Cambiano V, Bruun T, ; PARTNER Study Group. Risk of HIV transmission through condomless sex in serodifferent gay couples with the HIV-positive partner taking suppressive antiretroviral therapy (PARTNER): final results of a multicentre, prospective, observational study. Lancet 2019;393:2428–38. 10.1016/S0140-6736(19)30418-0 31056293 PMC6584382

[98] Panel on Antiretroviral Guidelines for Adults and Adolescents. Guidelines for the use of antiretroviral agents in adults and adolescents with HIV. Bethesda, MD: US Department of Health and Human Services, National Institutes of Health, AIDSinfo. https://clinicalinfo.hiv.gov/sites/default/files/inline-files/AdultandAdolescentGL.pdf

[99] Golden MR, Kerani RP, Stenger M, Effect of expedited partner therapy (EPT) on chlamydial prevalence: the Washington State Community-Level Trial. Presented at the STD Prevention Conference, Minneapolis, MN; March 12–15, 2012.

[100] Philip SS, Yu X, Donnell D, Vittinghoff E, Buchbinder S. Serosorting is associated with a decreased risk of HIV seroconversion in the EXPLORE Study Cohort. PLoS One 2010;5:e12662. 10.1371/journal.pone.0012662 20844744 PMC2936578

[101] Vallabhaneni S, Li X, Vittinghoff E, Donnell D, Pilcher CD, Buchbinder SP. Seroadaptive practices: association with HIV acquisition among HIV-negative men who have sex with men. PLoS One 2012;7:e45718. 10.1371/journal.pone.0045718 23056215 PMC3463589

[102] Jin F, Prestage GP, Templeton DJ, The impact of HIV seroadaptive behaviors on sexually transmissible infections in HIV-negative homosexual men in Sydney, Australia. Sex Transm Dis 2012;39:191–4. 10.1097/OLQ.0b013e3182401a2f 22337105 PMC3282017

[103] Hotton AL, Gratzer B, Mehta SD. Association between serosorting and bacterial sexually transmitted infection among HIV-negative men who have sex with men at an urban lesbian, gay, bisexual, and transgender health center. Sex Transm Dis 2012;39:959–64. 10.1097/OLQ.0b013e31826e870d 23191950

[104] Anderson C, Gallo MF, Hylton-Kong T, Randomized controlled trial on the effectiveness of counseling messages for avoiding unprotected sexual intercourse during sexually transmitted infection and reproductive tract infection treatment among female sexually transmitted infection clinic patients. Sex Transm Dis 2013;40:105–10. 10.1097/OLQ.0b013e31827938a1 23321990 PMC3811001

[105] Golden MR, Hogben M, Handsfield HH, St Lawrence JS, Potterat JJ, Holmes KK. Partner notification for HIV and STD in the United States: low coverage for gonorrhea, chlamydial infection, and HIV. Sex Transm Dis 2003;30:490–6. 10.1097/00007435-200306000-00004 12782949

[106] Katz DA, Dombrowski JC, Kerani RP, Integrating HIV testing as an outcome of STD partner services for men who have sex with men. AIDS Patient Care STDS 2016;30:208–14. 10.1089/apc.2016.0027 27158848 PMC4870606

[107] Katz DA, Dombrowski JC, Barry M, Spellman D, Bell TR, Golden MR. STD partner services to monitor and promote HIV pre-exposure prophylaxis use among men who have sex with men. J Acquir Immune Defic Syndr 2019;80:533–41. 10.1097/QAI.0000000000001952 30649032 PMC7027991

[108] Bocour A, Renaud TC, Udeagu CC, Shepard CW. HIV partner services are associated with timely linkage to HIV medical care. AIDS 2013;27:2961–3. 10.1097/QAD.0000000000000031 24189585

[109] Tesoriero JM, Johnson BL, Hart-Malloy R, Improving retention in HIV care through New York’s expanded partner services Data-to-Care pilot. J Public Health Manag Pract 2017;23:255–63. 10.1097/PHH.0000000000000483 27902561 PMC5381495

[110] Trelle S, Shang A, Nartey L, Cassell JA, Low N. Improved effectiveness of partner notification for patients with sexually transmitted infections: systematic review. BMJ 2007;334:354. 10.1136/bmj.39079.460741.7C 17237298 PMC1801006

[111] CDC. Recommendations for partner services programs for HIV infection, syphilis, gonorrhea, and chlamydial infection. MMWR Recomm Rep 2008;57(No. RR-9). 18987617

[112] Thurman AR, Shain RN, Holden AE, Champion JD, Perdue ST, Piper JM. Partner notification of sexually transmitted infections: a large cohort of Mexican American and African American women. Sex Transm Dis 2008;35:136–40. 10.1097/OLQ.0b013e318151498f 17898679

[113] Kissinger PJ, Niccolai LM, Magnus M, Partner notification for HIV and syphilis: effects on sexual behaviors and relationship stability. Sex Transm Dis 2003;30:75–82. 10.1097/00007435-200301000-00015 12514447

[114] Smith SG, Zhang X, Basile KC, The National Intimate Partner and Sexual Violence Survey: 2015 data brief—updated release. Atlanta GA: US Department of Health and Human Services, CDC, National Center for Injury Prevention and Control; 2018. https://www.cdc.gov/violenceprevention/pdf/2015data-brief508.pdf

[115] Wilson TE, Hogben M, Malka ES, A randomized controlled trial for reducing risks for sexually transmitted infections through enhanced patient-based partner notification. Am J Public Health 2009;99(Suppl 1):S104–10. 10.2105/AJPH.2007.112128 18556619 PMC2724934

[116] Yu YY, Frasure-Williams JA, Dunne EF, Bolan G, Markowitz L, Bauer HM. Chlamydia partner services for females in California family planning clinics. Sex Transm Dis 2011;38:913–8. 10.1097/OLQ.0b013e3182240366 21934563

[117] Mickiewicz T, Al-Tayyib A, Thrun M, Rietmeijer C. Implementation and effectiveness of an expedited partner therapy program in an urban clinic. Sex Transm Dis 2012;39:923–9. 10.1097/OLQ.0b013e3182756f20 23169171

[118] Kachur R, Strona FV, Kinsey J, Collins D. Introducing technology into partner services: a toolkit for programs. Atlanta, GA: US Department of Health and Human Services, CDC; 2015. https://www.cdc.gov/std/program/ips/ips-toolkit-12-28-2015.pdf

[119] Kachur R, Hall W, Coor A, Kinsey J, Collins D, Strona FV. The use of technology for sexually transmitted disease partner services in the United States: a structured review. Sex Transm Dis 2018;45:707–12. 10.1097/OLQ.0000000000000864 29771868 PMC6546166

[120] Pellowski J, Mathews C, Kalichman MO, Dewing S, Lurie MN, Kalichman SC. Advancing partner notification through electronic communication technology: a review of acceptability and utilization research. J Health Commun 2016;21:629–37. 10.1080/10810730.2015.1128020 27144318 PMC4948177

[121] Borchardt LN, Pickett ML, Tan KT, Visotcky AM, Drendel AL. Expedited partner therapy: pharmacist refusal of legal prescriptions. Sex Transm Dis 2018;45:350–3. 10.1097/OLQ.0000000000000751 29465689 PMC5895524

[122] Qin JZ, Diniz CP, Coleman JS. Pharmacy-level barriers to implementing expedited partner therapy in Baltimore, Maryland. Am J Obstet Gynecol 2018;218:504.e1–6. 10.1016/j.ajog.2018.01.036 29410060 PMC5916341

[123] Schillinger J, Slutsker J, Tsang L, Do prescriptions for expedited partner therapy get filled? Findings from a multi-jurisdictional evaluation, US, 2017–2018. Sex Transm Infect 2019;95(Suppl 1):A107. 10.1097/OLQ.0000000000001163 32149956

[124] Slutsker JS, Tsang LB, Schillinger JA. Do prescriptions for expedited partner therapy for chlamydia get filled? Findings from a multi-jurisdictional evaluation, United States, 2017–2019. Sex Transm Dis 2020;47:376–82. 10.1097/OLQ.0000000000001163 32149956

[125] Golden MR, Whittington WL, Handsfield HH, Effect of expedited treatment of sex partners on recurrent or persistent gonorrhea or chlamydial infection. N Engl J Med 2005;352:676–85. 10.1056/NEJMoa041681 15716561

[126] Schillinger JA, Kissinger P, Calvet H, Patient-delivered partner treatment with azithromycin to prevent repeated *Chlamydia trachomatis* infection among women: a randomized, controlled trial. Sex Transm Dis 2003;30:49–56. 10.1097/00007435-200301000-00011 12514443

[127] Kissinger P, Mohammed H, Richardson-Alston G, Patient-delivered partner treatment for male urethritis: a randomized, controlled trial. Clin Infect Dis 2005;41:623–9. 10.1086/432476 16080084

[128] Cameron ST, Glasier A, Scott G, Novel interventions to reduce re-infection in women with chlamydia: a randomized controlled trial. Hum Reprod 2009;24:888–95. 10.1093/humrep/den475 19136481

[129] Kissinger P, Schmidt N, Mohammed H, Patient-delivered partner treatment for *Trichomonas vaginalis* infection: a randomized controlled trial. Sex Transm Dis 2006;33:445–50. 10.1097/01.olq.0000204511.84485.4c 16531939

[130] Schwebke JR, Desmond RA. A randomized controlled trial of partner notification methods for prevention of trichomoniasis in women. Sex Transm Dis 2010;37:392–6. 10.1097/OLQ.0b013e3181dd1691 20453720

[131] Stephens SC, Bernstein KT, Katz MH, Philip SS, Klausner JD. The effectiveness of patient-delivered partner therapy and chlamydial and gonococcal reinfection in San Francisco. Sex Transm Dis 2010;37:525–9. 10.1097/OLQ.0b013e3181d8920f 20502392

[132] Kerani RP, Fleming M, DeYoung B, Golden MR. A randomized, controlled trial of inSPOT and patient-delivered partner therapy for gonorrhea and chlamydial infection among men who have sex with men. Sex Transm Dis 2011;38:941–6. 10.1097/OLQ.0b013e318223fcbc 21934569

[133] Stekler J, Bachmann L, Brotman RM, Concurrent sexually transmitted infections (STIs) in sex partners of patients with selected STIs: implications for patient-delivered partner therapy. Clin Infect Dis 2005;40:787–93. 10.1086/428043 15736009

[134] McNulty A, Teh MF, Freedman E. Patient delivered partner therapy for chlamydial infection—what would be missed? Sex Transm Dis 2008;35:834–6. 10.1097/OLQ.0b013e3181761993 18580822

[135] Schillinger J, Jamison K, Slutsker J, STI and HIV infections among MSM reporting exposure to gonorrhea or chlamydia: implications for expedited partner therapy. Sex Transm Infect 2019;95(Suppl 1):A107. 10.1136/sextrans-2019-sti.272

[136] Turner AN, Feldblum PJ, Hoke TH. Baseline infection with a sexually transmitted disease is highly predictive of reinfection during follow-up in Malagasy sex workers. Sex Transm Dis 2010;37:559–62. 10.1097/OLQ.0b013e3181d70a03 20716996

[137] Peterman TA, Tian LH, Metcalf CA, ; RESPECT-2 Study Group. High incidence of new sexually transmitted infections in the year following a sexually transmitted infection: a case for rescreening. Ann Intern Med 2006;145:564–72. 10.7326/0003-4819-145-8-200610170-00005 17043338

[138] Owens DK, Davidson KW, Krist AH, ; US Preventive Services Task Force. Screening for HIV infection: US Preventive Services Task Force recommendation statement. JAMA 2019;321:2326–36. 10.1001/jama.2019.6587 31184701

[139] Health and Human Services Panel on Treatment of HIV-Infected Pregnant Women and Prevention of Perinatal Transmission. Recommendations for use of antiretroviral drugs in pregnant HIV-1-infected women for maternal health and interventions to reduce perinatal HIV transmission in the United States. Bethesda, MD: US Department of Health and Human Services, National Institutes of Health, AIDSinfo; 2014. https://npin.cdc.gov/publication/recommendations-use-antiretroviral-drugs-pregnant-hiv-1-infected-women-maternal-health

[140] Committee on Obstetric Practice HIV Expert Work Group. ACOG Committee opinion no. 752: prenatal and perinatal human immunodeficiency virus testing. Obstet Gynecol 2018;132:e138–42. 10.1097/AOG.0000000000002825 30134428

[141] CDC. Sexually transmitted disease surveillance 2019 [Internet]. Atlanta GA: US Department of Health and Human Services, CDC; 2021. https://www.cdc.gov/std/statistics/2019/default.htm

[142] Warren HP, Cramer R, Kidd S, Leichliter JS. State requirements for prenatal syphilis screening in the United States, 2016. Matern Child Health J 2018;22:1227–32. 10.1007/s10995-018-2592-0 30019155 PMC6747684

[143] Lin JS, Eder M, Bean S. Screening for syphilis infection in pregnant women: a reaffirmation evidence update for the U.S. Preventive Services Task Force. Evidence Synthesis No. 167. AHRQ Publication No. 18-05238-EF-1. Rockville, MD: Agency for Healthcare Research and Quality; 2018. 30234936

[144] Neblett Fanfair R, Tao G, Owusu-Edusei K, Gift TL, Bernstein KT. Suboptimal prenatal syphilis testing among commercially insured women in the United States, 2013. Sex Transm Dis 2017;44:219–21. 10.1097/OLQ.0000000000000569 28282647

[145] Patel CG, Huppert JS, Tao G. Provider adherence to syphilis testing recommendations for women delivering a stillbirth. Sex Transm Dis 2017;44:685–90. 10.1097/OLQ.0000000000000656 28876321 PMC6784821

[146] Matthias JM, Rahman MM, Newman DR, Peterman TA. Effectiveness of prenatal screening and treatment to prevent congenital syphilis, Louisiana and Florida, 2013–2014. Sex Transm Dis 2017;44:498–502. 10.1097/OLQ.0000000000000638 28703731 PMC6190676

[147] Albright CM, Emerson JB, Werner EF, Hughes BL. Third-trimester prenatal syphilis screening: a cost-effectiveness analysis. Obstet Gynecol 2015;126:479–85. 10.1097/AOG.0000000000000997 26244531

[148] Owens DK, Davidson KW, Krist AH, ; US Preventive Services Task Force. Screening for hepatitis B virus infection in pregnant women: US Preventive Services Task Force reaffirmation recommendation statement. JAMA 2019;322:349–54. 10.1001/jama.2019.9365 31334800

[149] LeFevre ML; US Preventive Services Task Force. Screening for chlamydia and gonorrhea: U.S. Preventive Services Task Force recommendation statement. Ann Intern Med 2014;161:902–10. 10.7326/M14-1981 25243785

[150] Watts T, Stockman L, Martin J, Guilfoyle S, Vergeront JM. Increased risk for mother-to-infant transmission of hepatitis C virus among Medicaid recipients—Wisconsin, 2011–2015. MMWR Morb Mortal Wkly Rep 2017;66:1136–9. 10.15585/mmwr.mm6642a3 29072864 PMC5689103

[151] Patrick SW, Bauer AM, Warren MD, Jones TF, Wester C. Hepatitis C virus infection among women giving birth—Tennessee and United States, 2009–2014. MMWR Morb Mortal Wkly Rep 2017;66:470–3. 10.15585/mmwr.mm6618a3 28493860 PMC5657980

[152] Chappell CA, Hillier SL, Crowe D, Meyn LA, Bogen DL, Krans EE. Hepatitis C virus screening among children exposed during pregnancy. Pediatrics 2018;141:e20173273. 10.1542/peds.2017-3273 29720535 PMC5984711

[153] Gowda C, Kennedy S, Glover C, Prasad MR, Wang L, Honegger JR. Enhanced identification of maternal hepatitis C virus infection using existing public health surveillance systems. Paediatr Perinat Epidemiol 2018;32:401–10. 10.1111/ppe.12481 29972246 PMC6512319

[154] Waruingi W, Mhanna MJ, Kumar D, Abughali N. Hepatitis C virus universal screening versus risk based selective screening during pregnancy. J Neonatal Perinatal Med 2015;8:371–8. 10.3233/NPM-15915024 26836823

[155] Boudova S, Mark K, El-Kamary SS. Risk-based hepatitis C screening in pregnancy is less reliable than universal screening: a retrospective chart review. Open Forum Infect Dis 2018;5:ofy043. 10.1093/ofid/ofy043 29564364 PMC5846293

[156] Schillie S, Wester C, Osborne M, Wesolowski L, Ryerson AB. CDC recommendations for hepatitis C screening among adults—United States, 2020. MMWR Recomm Rep 2020;69(No. RR-2). 10.15585/mmwr.rr6902a1 32271723 PMC7147910

[157] Moyer VA; US Preventive Services Task Force. Screening for hepatitis C virus infection in adults: U.S. Preventive Services Task Force recommendation statement. Ann Intern Med 2013;159:349–57. 10.7326/0003-4819-159-5-201309030-00672 23798026

[158] Perkins RB, Guido RS, Castle PE, ; 2019 ASCCP Risk-Based Management Consensus Guidelines Committee. 2019 ASCCP risk-based management consensus guidelines for abnormal cervical cancer screening tests and cancer precursors. J Low Genit Tract Dis 2020;24:102–31. 10.1097/LGT.0000000000000525 32243307 PMC7147428

[159] Owens DK, Davidson KW, Krist AH, ; US Preventive Services Task Force. Screening for bacterial vaginosis in pregnant persons to prevent preterm delivery: US Preventive Services Task Force recommendation statement. JAMA 2020;323:1286–92. 10.1001/jama.2020.2684 32259236

[160] American Academy of Pediatrics Committee on Fetus and Newborn; American College of Obstetrics and Gynecology Committee on Obstetric Practice. Guidelines for perinatal care. Kilpatrick SJ, Papile LA, eds. 8th ed. Itasca, IL: American Academy of Pediatrics and Washington, DC: American College of Obstetrics and Gynecology; 2017.

[161] Curry SJ, Krist AH, Owens DK, ; US Preventive Services Task Force. Screening for syphilis infection in pregnant women: US Preventive Services Task Force reaffirmation recommendation statement. JAMA 2018;320:911–7. 10.1001/jama.2018.11785 30193283

[162] Bibbins-Domingo K, Grossman DC, Curry SJ, ; US Preventive Services Task Force. Serologic screening for genital herpes infection: US Preventive Services Task Force recommendation statement. JAMA 2016;316:2525–30. 10.1001/jama.2016.16776 27997659

[163] Selph SS, Bougatsos C, Dana T, Grusing S, Chou R. Screening for HIV infection in pregnant women: updated evidence report and systematic review for the US Preventive Services Task Force. JAMA 2019;321:2349–60. 10.1001/jama.2019.2593 31184704

[164] Henderson JT, Webber EM, Bean SI. Screening for Hepatitis B infection in pregnant women: updated evidence report and systematic review for the US Preventive Services Task Force. JAMA 2019;322:360–2. 10.1001/jama.2019.1655 31334780 PMC6652153

[165] Leichliter JS, Dittus PJ, Copen CE, Aral SO. Trends in factors indicating increased risk for STI among key subpopulations in the United States, 2002–2015. Sex Transm Infect 2020;96:121–3. 10.1136/sextrans-2019-054045 31350378 PMC6982539

[166] Committee on Adolescence; Council on Clinical and Information Technology; Blythe MJ, Del Beccaro MA. Standards for health information technology to ensure adolescent privacy. Pediatrics 2012;130:987–90. 10.1542/peds.2012-2580 23109684

[167] ACOG Committee Opinion no. 599: Committee on Adolescent Health Care: adolescent confidentiality and electronic health records. Obstet Gynecol 2014;123:1148–50. 10.1097/01.AOG.0000446825.08715.98 24785881

[168] Thompson LA, Martinko T, Budd P, Mercado R, Schentrup AM. Meaningful use of a confidential adolescent patient portal. J Adolesc Health 2016;58:134–40. 10.1016/j.jadohealth.2015.10.015 26802988

[169] Society for Adolescent Health and Medicine; American Academy of Pediatrics. Confidentiality protections for adolescents and young adults in the health care billing and insurance claims process. J Adolesc Health 2016;58:374–7. 10.1016/j.jadohealth.2015.12.009 26903437

[170] Bamberger DM, Graham G, Dennis L, Gerkovich MM. Extragenital gonorrhea and chlamydia among men and women according to type of sexual exposure. Sex Transm Dis 2019;46:329–34. 10.1097/OLQ.0000000000000967 30676485

[171] Chan PA, Robinette A, Montgomery M, Extragenital infections caused by *Chlamydia trachomatis* and *Neisseria gonorrhoeae*: a review of the literature. Infect Dis Obstet Gynecol 2016;2016:5758387. 10.1155/2016/5758387 27366021 PMC4913006

[172] Owusu-Edusei K Jr, Hoover KW, Gift TL. Cost-effectiveness of opt-out chlamydia testing for high-risk young women in the U.S. Am J Prev Med 2016;51:216–24. 10.1016/j.amepre.2016.01.007 26952078 PMC6785744

[173] DiClemente RJ, Sales JM, Danner F, Crosby RA. Association between sexually transmitted diseases and young adults’ self-reported abstinence. Pediatrics 2011;127:208–13. 10.1542/peds.2009-0892 21199852 PMC3387859

[174] Curry SJ, Krist AH, Owens DK, ; US Preventive Services Task Force. Screening for cervical cancer: US Preventive Services Task Force recommendation statement. JAMA 2018;320:674–86. 10.1001/jama.2018.10897 30140884

[175] Committee on Practice Bulletins—Gynecology. Practice bulletin no. 157: cervical cancer screening and prevention. Obstet Gynecol 2016;127:e1–20. 10.1097/AOG.0000000000001263 26695583

[176] Benard VB, Watson M, Castle PE, Saraiya M. Cervical carcinoma rates among young females in the United States. Obstet Gynecol 2012;120:1117–23. 10.1097/AOG.0b013e31826e4609 23090530 PMC4540330

[177] Fontham ETH, Wolf AMD, Church TR, Cervical cancer screening for individuals at average risk: 2020 guideline update from the American Cancer Society. CA Cancer J Clin 2020;70:321–46. 10.3322/caac.21628 32729638

[178] Owens DK, Davidson KW, Krist AH, ; US Preventive Services Task Force. Preexposure prophylaxis for the prevention of HIV infection: US Preventive Services Task Force recommendation statement. JAMA 2019;321:2203–13. 10.1001/jama.2019.6390 31184747

[179] Mayer KH, Bekker LG, Stall R, Grulich AE, Colfax G, Lama JR. Comprehensive clinical care for men who have sex with men: an integrated approach. Lancet 2012;380:378–87. 10.1016/S0140-6736(12)60835-6 22819653 PMC5603076

[180] Buchbinder SP, Vittinghoff E, Heagerty PJ, Sexual risk, nitrite inhalant use, and lack of circumcision associated with HIV seroconversion in men who have sex with men in the United States. J Acquir Immune Defic Syndr 2005;39:82–9. 10.1097/01.qai.0000134740.41585.f4 15851918

[181] Paz-Bailey G, Mendoza MC, Finlayson T, ; NHBS Study Group. Trends in condom use among MSM in the United States: the role of antiretroviral therapy and seroadaptive strategies. AIDS 2016;30:1985–90. 10.1097/QAD.0000000000001139 27149088 PMC5838316

[182] Spicknall IH, Gift TL, Bernstein KT, Aral SO. Sexual networks and infection transmission networks among men who have sex with men as causes of disparity and targets of prevention. Sex Transm Infect 2017;93:307–8. 10.1136/sextrans-2016-052676 28389442 PMC12243888

[183] Glick SN, Morris M, Foxman B, A comparison of sexual behavior patterns among men who have sex with men and heterosexual men and women. J Acquir Immune Defic Syndr 2012;60:83–90. 10.1097/QAI.0b013e318247925e 22522237 PMC3334840

[184] Goodreau SM, Golden MR. Biological and demographic causes of high HIV and sexually transmitted disease prevalence in men who have sex with men. Sex Transm Infect 2007;83:458–62. 10.1136/sti.2007.025627 17855487 PMC2598698

[185] Chew Ng RA, Samuel MC, Lo T, Sex, drugs (methamphetamines), and the Internet: increasing syphilis among men who have sex with men in California, 2004–2008. Am J Public Health 2013;103:1450–6. 10.2105/AJPH.2012.300808 23153138 PMC4007854

[186] Bernstein KT, Stephens SC, Strona FV, Kohn RP, Philip SS. Epidemiologic characteristics of an ongoing syphilis epidemic among men who have sex with men, San Francisco. Sex Transm Dis 2013;40:11–7. 10.1097/OLQ.0b013e31827763ea 23254114 PMC6830067

[187] Cohen SE, Chew Ng RA, Katz KA, Repeat syphilis among men who have sex with men in California, 2002–2006: implications for syphilis elimination efforts. Am J Public Health 2012;102:e1–8. 10.2105/AJPH.2011.300383 22095364 PMC3490561

[188] Kirkcaldy RD, Harvey A, Papp JR, *Neisseria gonorrhoeae* antimicrobial susceptibility surveillance—The Gonococcal Isolate Surveillance Project, 27 sites, United States, 2014. MMWR Surveill Summ 2016;65(No. SS-7). 10.15585/mmwr.ss6507a1 27414503

[189] Kirkcaldy RD, Zaidi A, Hook EW 3rd, *Neisseria gonorrhoeae* antimicrobial resistance among men who have sex with men and men who have sex exclusively with women: the Gonococcal Isolate Surveillance Project, 2005–2010. Ann Intern Med 2013;158:321–8. 10.7326/0003-4819-158-5-201303050-00004 23460055 PMC6697257

[190] Newman LM, Dowell D, Bernstein K, A tale of two gonorrhea epidemics: results from the STD Surveillance Network. Public Health Rep 2012;127:282–92. 10.1177/003335491212700308 22547859 PMC3314072

[191] Hess KL, Hu X, Lansky A, Mermin J, Hall HI. Lifetime risk of a diagnosis of HIV infection in the United States. Ann Epidemiol 2017;27:238–43. 10.1016/j.annepidem.2017.02.003 28325538 PMC5524204

[192] Patel P, Borkowf CB, Brooks JT, Lasry A, Lansky A, Mermin J. Estimating per-act HIV transmission risk: a systematic review. AIDS 2014;28:1509–19. 10.1097/QAD.0000000000000298 24809629 PMC6195215

[193] Koblin BA, Husnik MJ, Colfax G, Risk factors for HIV infection among men who have sex with men. AIDS 2006;20:731–9. 10.1097/01.aids.0000216374.61442.55 16514304

[194] Ackers ML, Greenberg AE, Lin CY, High and persistent HIV seroincidence in men who have sex with men across 47 U.S. cities. PLoS One 2012;7:e34972. 10.1371/journal.pone.0034972 22529964 PMC3329535

[195] Zetola NM, Bernstein KT, Wong E, Louie B, Klausner JD. Exploring the relationship between sexually transmitted diseases and HIV acquisition by using different study designs. J Acquir Immune Defic Syndr 2009;50:546–51. 10.1097/QAI.0b013e318195bd2b 19367993 PMC2680242

[196] Solomon MM, Mayer KH, Glidden DV, ; iPrEx Study Team. Syphilis predicts HIV incidence among men and transgender women who have sex with men in a preexposure prophylaxis trial. Clin Infect Dis 2014;59:1020–6. 10.1093/cid/ciu450 24928295 PMC4166980

[197] Fleming DT, Wasserheit JN. From epidemiological synergy to public health policy and practice: the contribution of other sexually transmitted diseases to sexual transmission of HIV infection. Sex Transm Infect 1999;75:3–17. 10.1136/sti.75.1.3 10448335 PMC1758168

[198] Freeman EE, Weiss HA, Glynn JR, Cross PL, Whitworth JA, Hayes RJ. Herpes simplex virus 2 infection increases HIV acquisition in men and women: systematic review and meta-analysis of longitudinal studies. AIDS 2006;20:73–83. 10.1097/01.aids.0000198081.09337.a7 16327322

[199] Reynolds SJ, Risbud AR, Shepherd ME, High rates of syphilis among STI patients are contributing to the spread of HIV-1 in India. Sex Transm Infect 2006;82:121–6. 10.1136/sti.2005.015040 16581736 PMC2564682

[200] Hoots BE, Wejnert C, Martin A, ; NHBS Study Group. Undisclosed HIV infection among MSM in a behavioral surveillance study. AIDS 2019;33:913–8. 10.1097/QAD.0000000000002147 30649053

[201] Dolling DI, Desai M, McOwan A, ; PROUD Study Group. An analysis of baseline data from the PROUD study: an open-label randomised trial of pre-exposure prophylaxis. Trials 2016;17:163. 10.1186/s13063-016-1286-4 27013513 PMC4806447

[202] Oldenburg CE, Nunn AS, Montgomery M, Behavioral changes following uptake of HIV pre-exposure prophylaxis among men who have sex with men in a clinical setting. AIDS Behav 2018;22:1075–9. 10.1007/s10461-017-1701-1 28150120 PMC5538946

[203] Montaño MA, Dombrowski JC, Dasgupta S, Changes in sexual behavior and STI diagnoses among MSM initiating PrEP in a clinic setting. AIDS Behav 2019;23:548–55. 10.1007/s10461-018-2252-9 30117076 PMC6368873

[204] Traeger MW, Schroeder SE, Wright EJ, Effects of pre-exposure prophylaxis for the prevention of human immunodeficiency virus infection on sexual risk behavior in men who have sex with men: a systematic review and meta-analysis. Clin Infect Dis 2018;67:676–86. 10.1093/cid/ciy182 29509889

[205] Jenness SM, Weiss KM, Goodreau SM, Incidence of gonorrhea and chlamydia following human immunodeficiency virus preexposure prophylaxis among men who have sex with men: a modeling study. Clin Infect Dis 2017;65:712–8. 10.1093/cid/cix439 28505240 PMC5848234

[206] Tang EC, Vittinghoff E, Philip SS, Quarterly screening optimizes detection of sexually transmitted infections when prescribing HIV preexposure prophylaxis. AIDS 2020;34:1181–6. 10.1097/QAD.0000000000002522 32205724

[207] Barbee LA, Khosropour CM, Dombrowksi JC, Golden MR. New human immunodeficiency virus diagnosis independently associated with rectal gonorrhea and chlamydia in men who have sex with men. Sex Transm Dis 2017;44:385–9. 10.1097/OLQ.0000000000000614 28608786 PMC5481158

[208] Bernstein KT, Marcus JL, Nieri G, Philip SS, Klausner JD. Rectal gonorrhea and chlamydia reinfection is associated with increased risk of HIV seroconversion. J Acquir Immune Defic Syndr 2010;53:537–43. 10.1097/QAI.0b013e3181c3ef29 19935075

[209] Barbee LA, Khosropour CM, Dombrowski JC, Manhart LE, Golden MR. An estimate of the proportion of symptomatic gonococcal, chlamydial and non-gonococcal non-chlamydial urethritis attributable to oral sex among men who have sex with men: a case-control study. Sex Transm Infect 2016;92:155–60. 10.1136/sextrans-2015-052214 26297719 PMC4861816

[210] Lafferty WE, Hughes JP, Handsfield HH. Sexually transmitted diseases in men who have sex with men. Acquisition of gonorrhea and nongonococcal urethritis by fellatio and implications for STD/HIV prevention. Sex Transm Dis 1997;24:272–8. 10.1097/00007435-199705000-00007 9153736

[211] Bernstein KT, Stephens SC, Barry PM, *Chlamydia trachomatis* and *Neisseria gonorrhoeae* transmission from the oropharynx to the urethra among men who have sex with men. Clin Infect Dis 2009;49:1793–7. 10.1086/648427 19911970

[212] Patton ME, Kidd S, Llata E, Extragenital gonorrhea and chlamydia testing and infection among men who have sex with men—STD Surveillance Network, United States, 2010–2012. Clin Infect Dis 2014;58:1564–70. 10.1093/cid/ciu184 24647015 PMC4666527

[213] Kent CK, Chaw JK, Wong W, Prevalence of rectal, urethral, and pharyngeal chlamydia and gonorrhea detected in 2 clinical settings among men who have sex with men: San Francisco, California, 2003. Clin Infect Dis 2005;41:67–74. 10.1086/430704 15937765

[214] Koedijk FD, van Bergen JE, Dukers-Muijrers NH, van Leeuwen AP, Hoebe CJ, van der Sande MA; Dutch STI centres. The value of testing multiple anatomic sites for gonorrhoea and chlamydia in sexually transmitted infection centres in the Netherlands, 2006–2010. Int J STD AIDS 2012;23:626–31. 10.1258/ijsa.2012.011378 23033514

[215] Barbee LA, Dombrowski JC, Kerani R, Golden MR. Effect of nucleic acid amplification testing on detection of extragenital gonorrhea and chlamydial infections in men who have sex with men sexually transmitted disease clinic patients. Sex Transm Dis 2014;41:168–72. 10.1097/OLQ.0000000000000093 24521722

[216] Danby CS, Cosentino LA, Rabe LK, Patterns of extragenital chlamydia and gonorrhea in women and men who have sex with men reporting a history of receptive anal intercourse. Sex Transm Dis 2016;43:105–9. 10.1097/OLQ.0000000000000384 26766527 PMC4955797

[217] van der Helm JJ, Hoebe CJ, van Rooijen MS, High performance and acceptability of self-collected rectal swabs for diagnosis of *Chlamydia trachomatis* and *Neisseria gonorrhoeae* in men who have sex with men and women. Sex Transm Dis 2009;36:493–7. 10.1097/OLQ.0b013e3181a44b8c 19617869

[218] Alexander S, Ison C, Parry J, ; Brighton Home Sampling Kits Steering Group. Self-taken pharyngeal and rectal swabs are appropriate for the detection of *Chlamydia trachomatis* and *Neisseria gonorrhoeae* in asymptomatic men who have sex with men. Sex Transm Infect 2008;84:488–92. 10.1136/sti.2008.031443 19028953

[219] Freeman AH, Bernstein KT, Kohn RP, Philip S, Rauch LM, Klausner JD. Evaluation of self-collected versus clinician-collected swabs for the detection of *Chlamydia trachomatis* and *Neisseria gonorrhoeae* pharyngeal infection among men who have sex with men. Sex Transm Dis 2011;38:1036–9. 10.1097/OLQ.0b013e318227713e 21992980

[220] Chesson HW, Bernstein KT, Gift TL, Marcus JL, Pipkin S, Kent CK. The cost-effectiveness of screening men who have sex with men for rectal chlamydial and gonococcal infection to prevent HIV Infection. Sex Transm Dis 2013;40:366–71. 10.1097/OLQ.0b013e318284e544 23588125 PMC6745689

[221] Jenness SM, Weiss KM, Prasad P, Zlotorzynska M, Sanchez T. Bacterial sexually transmitted infection screening rates by symptomatic status among men who have sex with men in the United States: a hierarchical Bayesian analysis. Sex Transm Dis 2019;46:25–30. 10.1097/OLQ.0000000000000896 30044334 PMC6292678

[222] Hoover KW, Butler M, Workowski K, ; Evaluation Group for Adherence to STD and Hepatitis Screening. STD screening of HIV-infected MSM in HIV clinics. Sex Transm Dis 2010;37:771–6. 10.1097/OLQ.0b013e3181e50058 20585275

[223] de Voux A, Bernstein KT, Bradley H, Kirkcaldy RD, Tie Y, Shouse RL; Medical Monitoring Project. Syphilis testing among sexually active men who have sex with men and who are receiving medical care for human immunodeficiency virus in the United States: Medical Monitoring Project, 2013–2014. Clin Infect Dis 2019;68:934–9. 10.1093/cid/ciy571 29985985 PMC6563935

[224] Gray RT, Hoare A, Prestage GP, Donovan B, Kaldor JM, Wilson DP. Frequent testing of highly sexually active gay men is required to control syphilis. Sex Transm Dis 2010;37:298–305. 10.1097/OLQ.0b013e3181ca3c0a 20393383

[225] Tuite AR, Fisman DN, Mishra S. Screen more or screen more often? Using mathematical models to inform syphilis control strategies. BMC Public Health 2013;13:606. 10.1186/1471-2458-13-606 23800206 PMC3699384

[226] Tuite A, Fisman D. Go big or go home: impact of screening coverage on syphilis infection dynamics. Sex Transm Infect 2016;92:49–54. 10.1136/sextrans-2014-052001 25954016

[227] Tuite AR, Shaw S, Reimer JN, Ross CP, Fisman DN, Mishra S. Can enhanced screening of men with a history of prior syphilis infection stem the epidemic in men who have sex with men? A mathematical modelling study. Sex Transm Infect 2018;94:105–10. 10.1136/sextrans-2017-053201 28705938

[228] Raifman JR, Gebo KA, Mathews WC, ; HIV Research Network. Gonorrhea and chlamydia case detection increased when testing increased in a multisite US HIV cohort, 2004–2014. J Acquir Immune Defic Syndr 2017;76:409–16. 10.1097/QAI.0000000000001514 28777262 PMC5680900

[229] Barbee LA, Dhanireddy S, Tat SA, Marrazzo JM. Barriers to bacterial sexually transmitted infection testing of HIV-infected men who have sex with men engaged in HIV primary care. Sex Transm Dis 2015;42:590–4. 10.1097/OLQ.0000000000000320 26372931 PMC4576720

[230] McMillan A, Young H, Moyes A. Rectal gonorrhoea in homosexual men: source of infection. Int J STD AIDS 2000;11:284–7. 10.1177/095646240001100502 10824935

[231] Chow EP, Cornelisse VJ, Read TR, Chen MY, Bradshaw CS, Fairley CK. Saliva use in sex: associations with use of smartphone dating applications in men who have sex with men. Int J STD AIDS 2018;29:362–6. 10.1177/0956462417727669 28835197

[232] Cornelisse VJ, Priest D, Fairley CK, The frequency of kissing as part of sexual activity differs depending on how men meet their male casual sexual partners. Int J STD AIDS 2018;29:598–602. 10.1177/0956462417748717 29256822

[233] Krist AH, Davidson KW, Mangione CM, ; US Preventive Services Task Force. Screening for hepatitis B virus infection in adolescents and adults: US Preventive Services Task Force recommendation statement. JAMA 2020;324:2415–22. 10.1001/jama.2020.22980 33320230

[234] Tohme RA, Holmberg SD. Is sexual contact a major mode of hepatitis C virus transmission? Hepatology 2010;52:1497–505. 10.1002/hep.23808 20635398

[235] Wandeler G, Gsponer T, Bregenzer A, ; Swiss HIV Cohort Study. Hepatitis C virus infections in the Swiss HIV Cohort Study: a rapidly evolving epidemic. Clin Infect Dis 2012;55:1408–16. 10.1093/cid/cis694 22893583

[236] Garg S, Taylor LE, Grasso C, Mayer KH. Prevalent and incident hepatitis C virus infection among HIV-infected men who have sex with men engaged in primary care in a Boston community health center. Clin Infect Dis 2013;56:1480–7. 10.1093/cid/cit054 23386630 PMC3634307

[237] Urbanus AT, van de Laar TJ, Stolte IG, Hepatitis C virus infections among HIV-infected men who have sex with men: an expanding epidemic. AIDS 2009;23:F1–7. 10.1097/QAD.0b013e32832e5631 19542864

[238] Linas BP, Wong AY, Schackman BR, Kim AY, Freedberg KA. Cost-effective screening for acute hepatitis C virus infection in HIV-infected men who have sex with men. Clin Infect Dis 2012;55:279–90. 10.1093/cid/cis382 22491339 PMC3403839

[239] Taylor LE, DeLong AK, Maynard MA, Acute hepatitis C virus in an HIV clinic: a screening strategy, risk factors, and perception of risk. AIDS Patient Care STDS 2011;25:571–7. 10.1089/apc.2011.0106 21859307 PMC3183653

[240] Kaul R, Kimani J, Nagelkerke NJ, ; Kibera HIV Study Group. Monthly antibiotic chemoprophylaxis and incidence of sexually transmitted infections and HIV-1 infection in Kenyan sex workers: a randomized controlled trial. JAMA 2004;291:2555–62. 10.1001/jama.291.21.2555 15173146

[241] Ong JJ, Baggaley RC, Wi TE, Global epidemiologic characteristics of sexually transmitted infections among individuals using preexposure prophylaxis for the prevention of HIV infection: a systematic review and meta-analysis. JAMA Netw Open 2019;2:e1917134. 10.1001/jamanetworkopen.2019.17134 31825501 PMC6991203

[242] Paz-Bailey G, Hoots BE, Xia M, Finlayson T, Prejean J, Purcell DW; NHBS Study Group. Trends in Internet use among men who have sex with men in the United States. J Acquir Immune Defic Syndr 2017;75(Suppl 3):S288–95. 10.1097/QAI.0000000000001404 28604430 PMC5871925

[243] Badal HJ, Stryker JE, DeLuca N, Purcell DW. Swipe right: dating website and app use among men who have sex with men. AIDS Behav 2018;22:1265–72. 10.1007/s10461-017-1882-7 28884248

[244] Chan PA, Crowley C, Rose JS, A network analysis of sexually transmitted diseases and online hookup sites among men who have sex with men. Sex Transm Dis 2018;45:462–8. 10.1097/OLQ.0000000000000784 29465663 PMC5995630

[245] Beymer MR, Weiss RE, Bolan RK, Sex on demand: geosocial networking phone apps and risk of sexually transmitted infections among a cross-sectional sample of men who have sex with men in Los Angeles County. Sex Transm Infect 2014;90:567–72. 10.1136/sextrans-2013-051494 24926041 PMC4198579

[246] Medina MM, Crowley C, Montgomery MC, Disclosure of HIV serostatus and pre-exposure prophylaxis use on internet hookup sites among men who have sex with men. AIDS Behav 2019;23:1681–8. 10.1007/s10461-018-2286-z 30267365 PMC6438768

[247] Chan PA, Towey C, Poceta J, Online hookup sites for meeting sexual partners among men who have sex with men in Rhode Island, 2013: a call for public health action. Public Health Rep 2016;131:264–71. 10.1177/003335491613100210 26957661 PMC4765975

[248] Lampkin D, Crawley A, Lopez TP, Mejia CM, Yuen W, Levy V. Reaching suburban men who have sex with men for STD and HIV services through online social networking outreach: a public health approach. J Acquir Immune Defic Syndr 2016;72:73–8. 10.1097/QAI.0000000000000930 27097365

[249] Sun CJ, Stowers J, Miller C, Bachmann LH, Rhodes SD. Acceptability and feasibility of using established geosocial and sexual networking mobile applications to promote HIV and STD testing among men who have sex with men. AIDS Behav 2015;19:543–52. 10.1007/s10461-014-0942-5 25381563 PMC4359067

[250] Dritz SK, Back AF. Letter: *Shigella* enteritis venereally transmitted. N Engl J Med 1974;291:1194. 10.1056/NEJM197411282912223 4608062

[251] Aragón TJ, Vugia DJ, Shallow S, Case-control study of shigellosis in San Francisco: the role of sexual transmission and HIV infection. Clin Infect Dis 2007;44:327–34. 10.1086/510593 17205436

[252] Simms I, Field N, Jenkins C, Intensified shigellosis epidemic associated with sexual transmission in men who have sex with men—*Shigella flexneri* and *S. sonnei* in England, 2004 to end of February 2015. Euro Surveill 2015;20:21097. 10.2807/1560-7917.ES2015.20.15.21097 25953129

[253] Gilbart VL, Simms I, Jenkins C, Sex, drugs and smart phone applications: findings from semistructured interviews with men who have sex with men diagnosed with *Shigella flexneri* 3a in England and Wales. Sex Transm Infect 2015;91:598–602. 10.1136/sextrans-2015-052014 25921020

[254] Narayan S, Galanis E; BC STEI Group. Are enteric infections sexually transmitted in British Columbia? Can Commun Dis Rep 2016;42:24–9. 10.14745/ccdr.v42i02a01 29770000 PMC5864255

[255] Mohan K, Hibbert M, Rooney G, What is the overlap between HIV and shigellosis epidemics in England: further evidence of MSM transmission? Sex Transm Infect 2018;94:67–71. 10.1136/sextrans-2016-052962 28490580

[256] Hughes G, Silalang P, Were J, Prevalence and characteristics of gastrointestinal infections in men who have sex with men diagnosed with rectal chlamydia infection in the UK: an ‘unlinked anonymous’ cross-sectional study. Sex Transm Infect 2018;94:518–21. 10.1136/sextrans-2016-053057 28360379

[257] O’Sullivan B, Delpech V, Pontivivo G, Shigellosis linked to sex venues, Australia. Emerg Infect Dis 2002;8:862–4. 10.3201/eid0808.010534 12141976 PMC2732516

[258] Marcus U, Zucs P, Bremer V, Shigellosis—a re-emerging sexually transmitted infection: outbreak in men having sex with men in Berlin. Int J STD AIDS 2004;15:533–7. 10.1258/0956462041558221 15307964

[259] Danila RN, Eikmeier DL, Robinson TJ, La Pointe A, DeVries AS. Two concurrent enteric disease outbreaks among men who have sex with men, Minneapolis-St Paul area. Clin Infect Dis 2014;59:987–9. 10.1093/cid/ciu478 24944234

[260] Okame M, Adachi E, Sato H, *Shigella sonnei* outbreak among men who have sex with men in Tokyo. Jpn J Infect Dis 2012;65:277–8. 10.7883/yoken.65.277 22627317

[261] Wilmer A, Romney MG, Gustafson R, *Shigella flexneri* serotype 1 infections in men who have sex with men in Vancouver, Canada. HIV Med 2015;16:168–75. 10.1111/hiv.12191 25656740

[262] CDC. *Shigella sonnei* outbreak among men who have sex with men—San Francisco, California, 2000–2001. MMWR Morb Mortal Wkly Rep 2001;50:922–6. 11699845

[263] Baer JT, Vugia DJ, Reingold AL, Aragon T, Angulo FJ, Bradford WZ. HIV infection as a risk factor for shigellosis. Emerg Infect Dis 1999;5:820–3. 10.3201/eid0506.990614 10603219 PMC2640795

[264] Simms I, Gilbart VL, Byrne L, Identification of verocytotoxin-producing *Escherichia coli* O117:H7 in men who have sex with men, England, November 2013 to August 2014. Euro Surveill 2014;19:20946. 10.2807/1560-7917.ES2014.19.43.20946 25375900

[265] Quinn TC, Goodell SE, Fennell C, Infections with *Campylobacter jejuni* and *Campylobacter*-like organisms in homosexual men. Ann Intern Med 1984;101:187–92. 10.7326/0003-4819-101-2-187 6547580

[266] Gaudreau C, Pilon PA, Sylvestre J-L, Boucher F, Bekal S. Multidrug-resistant *Campylobacter coli* in men who have sex with men, Quebec, Canada, 2015. Emerg Infect Dis 2016;22:1661–3. 10.3201/eid2209.151695 27533504 PMC4994334

[267] Chen GJ, Lin KY, Hung CC, Chang SC. Hepatitis A outbreak among men who have sex with men in a country of low endemicity of hepatitis A infection. J Infect Dis 2017;215:1339–40. 10.1093/infdis/jix123 28329351

[268] Lo YC, Ji DD, Hung CC. Prevalent and incident HIV diagnoses among *Entamoeba histolytica*-infected adult males: a changing epidemiology associated with sexual transmission—Taiwan, 2006–2013. PLoS Negl Trop Dis 2014;8:e3222. 10.1371/journal.pntd.0003222 25299178 PMC4191956

[269] Stark D, van Hal SJ, Matthews G, Harkness J, Marriott D. Invasive amebiasis in men who have sex with men, Australia. Emerg Infect Dis 2008;14:1141–3. 10.3201/eid1407.080017 18598643 PMC2600324

[270] Mitchell H, Hughes G. Recent epidemiology of sexually transmissible enteric infections in men who have sex with men. Curr Opin Infect Dis 2018;31:50–6. 10.1097/QCO.0000000000000423 29251673

[271] Weatherburn P, Hickson F, Reid D, Torres-Rueda S, Bourne A. Motivations and values associated with combining sex and illicit drugs (‘chemsex’) among gay men in South London: findings from a qualitative study. Sex Transm Infect 2017;93:203–6. 10.1136/sextrans-2016-052695 27519259

[272] Baker KS, Dallman TJ, Ashton PM, Intercontinental dissemination of azithromycin-resistant shigellosis through sexual transmission: a cross-sectional study. Lancet Infect Dis 2015;15:913–21. 10.1016/S1473-3099(15)00002-X 25936611

[273] Bowen A, Grass J, Bicknese A, Campbell D, Hurd J, Kirkcaldy RD. Elevated risk for antimicrobial drug-resistant *Shigella* infection among men who have sex with men, United States, 2011–2015. Emerg Infect Dis 2016;22:1613–6. 10.3201/eid2209.160624 27533624 PMC4994375

[274] Gaudreau C, Rodrigues-Coutlée S, Pilon PA, Coutlée F, Bekal S. Long-lasting outbreak of erythromycin- and ciprofloxacin-resistant *Campylobacter jejuni* subspecies *jejuni* from 2003 to 2013 in men who have sex with men, Quebec, Canada. Clin Infect Dis 2015;61:1549–52. 10.1093/cid/civ570 26187024

[275] Muzny CA, Sunesara IR, Martin DH, Mena LA. Sexually transmitted infections and risk behaviors among African American women who have sex with women: does sex with men make a difference? Sex Transm Dis 2011;38:1118–25. 10.1097/OLQ.0b013e31822e6179 22082722

[276] Eisenberg M. Differences in sexual risk behaviors between college students with same-sex and opposite-sex experience: results from a national survey. Arch Sex Behav 2001;30:575–89. 10.1023/A:1011958816438 11725456

[277] Koh AS, Gómez CA, Shade S, Rowley E. Sexual risk factors among self-identified lesbians, bisexual women, and heterosexual women accessing primary care settings. Sex Transm Dis 2005;32:563–9. 10.1097/01.olq.0000175417.17078.21 16118605

[278] Goodenow C, Szalacha LA, Robin LE, Westheimer K. Dimensions of sexual orientation and HIV-related risk among adolescent females: evidence from a statewide survey. Am J Public Health 2008;98:1051–8. 10.2105/AJPH.2005.080531 18445809 PMC2377290

[279] Muzny CA, Austin EL, Harbison HS, Hook EW 3rd. Sexual partnership characteristics of African American women who have sex with women; impact on sexually transmitted infection risk. Sex Transm Dis 2014;41:611–7. 10.1097/OLQ.0000000000000194 25211257

[280] Riskind RG, Tornello SL, Younger BC, Patterson CJ. Sexual identity, partner gender, and sexual health among adolescent girls in the United States. Am J Public Health 2014;104:1957–63. 10.2105/AJPH.2014.302037 25121821 PMC4167074

[281] Schick V, Rosenberger JG, Herbenick D, Reece M. Sexual behaviour and risk reduction strategies among a multinational sample of women who have sex with women. Sex Transm Infect 2012;88:407–12. 10.1136/sextrans-2011-050404 22563015

[282] Richters J, Prestage G, Schneider K, Clayton S. Do women use dental dams? Safer sex practices of lesbians and other women who have sex with women. Sex Health 2010;7:165–9. 10.1071/SH09072 20465981

[283] Rowen TS, Breyer BN, Lin TC, Li CS, Robertson PA, Shindel AW. Use of barrier protection for sexual activity among women who have sex with women. Int J Gynaecol Obstet 2013;120:42–5. 10.1016/j.ijgo.2012.08.011 23106842 PMC3557847

[284] Lindley LL, Friedman DB, Struble C. Becoming visible: assessing the availability of online sexual health information for lesbians. Health Promot Pract 2012;13:472–80. 10.1177/1524839910390314 21677116

[285] Chetcuti N, Beltzer N, Methy N, Laborde C, Velter A, Bajos N; CSF Group. Preventive care’s forgotten women: life course, sexuality, and sexual health among homosexually and bisexually active women in France. J Sex Res 2013;50:587–97. 10.1080/00224499.2012.657264 22497621

[286] Logie CH, Navia D, Loutfy MR. Correlates of a lifetime history of sexually transmitted infections among women who have sex with women in Toronto, Canada: results from a cross-sectional internet-based survey. Sex Transm Infect 2015;91:278–83. 10.1136/sextrans-2014-051745 25477474

[R287] 287. Muzny CA, Kapil R, Austin EL, Hook EW, Geisler WM. Lower sexually transmissible infection prevalence among lifetime exclusive women who have sex with women compared with women who have sex with women and men. Sex Health 2014;11:592–3. 10.1071/SH14181 25435197

[288] Muzny CA, Harbison HS, Pembleton ES, Austin EL. Sexual behaviors, perception of sexually transmitted infection risk, and practice of safe sex among southern African American women who have sex with women. Sex Transm Dis 2013;40:395–400. 10.1097/OLQ.0b013e31828caf34 23588129

[289] Przedworski JM, McAlpine DD, Karaca-Mandic P, VanKim NA. Health and health risks among sexual minority women: an examination of 3 subgroups. Am J Public Health 2014;104:1045–7. 10.2105/AJPH.2013.301733 24825204 PMC4048874

[290] Brenick A, Romano K, Kegler C, Eaton LA. Understanding the influence of stigma and medical mistrust on engagement in routine healthcare among Black women who have sex with women. LGBT Health 2017;4:4–10. 10.1089/lgbt.2016.0083 28113005 PMC5278794

[291] Fethers K, Marks C, Mindel A, Estcourt CS. Sexually transmitted infections and risk behaviours in women who have sex with women. Sex Transm Infect 2000;76:345–9. 10.1136/sti.76.5.345 11141849 PMC1744205

[292] Marrazzo JM, Koutsky LA, Eschenbach DA, Agnew K, Stine K, Hillier SL. Characterization of vaginal flora and bacterial vaginosis in women who have sex with women. J Infect Dis 2002;185:1307–13. 10.1086/339884 12001048

[293] Kellock D, O’Mahony CP. Sexually acquired metronidazole-resistant trichomoniasis in a lesbian couple. Genitourin Med 1996;72:60–1. 10.1136/sti.72.1.60 8655171 PMC1195594

[294] Muzny CA, Rivers CA, Mena LA, Schwebke JR. Genotypic characterization of *Trichomonas vaginalis* isolates among women who have sex with women in sexual partnerships. Sex Transm Dis 2012;39:556–8. 10.1097/OLQ.0b013e31824f1c49 22706219

[295] Chan SK, Thornton LR, Chronister KJ, ; CDC. Likely female-to-female sexual transmission of HIV—Texas, 2012. MMWR Morb Mortal Wkly Rep 2014;63:209–12. 24622284 PMC5779339

[296] Kwakwa HA, Ghobrial MW. Female-to-female transmission of human immunodeficiency virus. Clin Infect Dis 2003;36:e40–1. 10.1086/345462 12539088

[297] Marrazzo JM, Handsfield HH, Whittington WL. Predicting chlamydial and gonococcal cervical infection: implications for management of cervicitis. Obstet Gynecol 2002;100:579–84. 10.1097/00006250-200209000-00029 12220782

[298] Diamant AL, Schuster MA, McGuigan K, Lever J. Lesbians’ sexual history with men: implications for taking a sexual history. Arch Intern Med 1999;159:2730–6. 10.1001/archinte.159.22.2730 10597764

[299] Xu F, Sternberg MR, Markowitz LE. Women who have sex with women in the United States: prevalence, sexual behavior and prevalence of herpes simplex virus type 2 infection—results from national health and nutrition examination survey 2001–2006. Sex Transm Dis 2010;37:407–13. 10.1097/OLQ.0b013e3181db2e18 20531032

[300] Everett BG, Higgins JA, Haider S, Carpenter E. Do sexual minorities receive appropriate sexual and reproductive health care and counseling? J Womens Health (Larchmt) 2019;28:53–62. 10.1089/jwh.2017.6866 30372369 PMC6343198

[301] Marrazzo JM, Koutsky LA, Stine KL, Genital human papillomavirus infection in women who have sex with women. J Infect Dis 1998;178:1604–9. 10.1086/314494 9815211

[302] Marrazzo JM, Koutsky LA, Kiviat NB, Kuypers JM, Stine K. Papanicolaou test screening and prevalence of genital human papillomavirus among women who have sex with women. Am J Public Health 2001;91:947–52. 10.2105/AJPH.91.6.947 11392939 PMC1446473

[303] Bailey JV, Kavanagh J, Owen C, McLean KA, Skinner CJ. Lesbians and cervical screening. Br J Gen Pract 2000;50:481–2. 10962789 PMC1313729

[304] Anderson TA, Schick V, Herbenick D, Dodge B, Fortenberry JD. A study of human papillomavirus on vaginally inserted sex toys, before and after cleaning, among women who have sex with women and men. Sex Transm Infect 2014;90:529–31. 10.1136/sextrans-2014-051558 24739872

[305] Marrazzo JM, Stine K, Wald A. Prevalence and risk factors for infection with herpes simplex virus type-1 and -2 among lesbians. Sex Transm Dis 2003;30:890–5. 10.1097/01.OLQ.0000091151.52656.E5 14646636

[306] Muzny CA, Schwebke JR. The clinical spectrum of *Trichomonas vaginalis* infection and challenges to management. Sex Transm Infect 2013;89:423–5. 10.1136/sextrans-2012-050893 23543252

[307] Muzny CA, Blackburn RJ, Sinsky RJ, Austin EL, Schwebke JR. Added benefit of nucleic acid amplification testing for the diagnosis of *Trichomonas vaginalis* among men and women attending a sexually transmitted diseases clinic. Clin Infect Dis 2014;59:834–41. 10.1093/cid/ciu446 24928292

[308] Singh D, Fine DN, Marrazzo JM. *Chlamydia trachomatis* infection among women reporting sexual activity with women screened in family planning clinics in the Pacific Northwest, 1997 to 2005. Am J Public Health 2011;101:1284–90. 10.2105/AJPH.2009.169631 20724697 PMC3110221

[309] Muzny CA, Kapil R, Austin EL, Brown L, Hook EW 3rd, Geisler WM. *Chlamydia trachomatis* infection in African American women who exclusively have sex with women. Int J STD AIDS 2016;27:978–83. 10.1177/0956462415604092 26384942 PMC7265900

[310] Koumans EH, Sternberg M, Bruce C, The prevalence of bacterial vaginosis in the United States, 2001–2004; associations with symptoms, sexual behaviors, and reproductive health. Sex Transm Dis 2007;34:864–9. 10.1097/OLQ.0b013e318074e565 17621244

[311] Evans AL, Scally AJ, Wellard SJ, Wilson JD. Prevalence of bacterial vaginosis in lesbians and heterosexual women in a community setting. Sex Transm Infect 2007;83:470–5. 10.1136/sti.2006.022277 17611235 PMC2598706

[312] Olson KM, Boohaker LJ, Schwebke JR, Aslibekyan S, Muzny CA. Comparisons of vaginal flora patterns among sexual behaviour groups of women: implications for the pathogenesis of bacterial vaginosis. Sex Health 2018;15:61–7. 10.1071/SH17087 29212588 PMC6890514

[313] Muzny CA, Lensing SY, Aaron KJ, Schwebke JR. Incubation period and risk factors support sexual transmission of bacterial vaginosis in women who have sex with women. Sex Transm Infect 2019;95:511–5. 10.1136/sextrans-2018-053824 30872415 PMC7265897

[314] Bradshaw CS, Walker SM, Vodstrcil LA, The influence of behaviors and relationships on the vaginal microbiota of women and their female partners: the WOW Health Study. J Infect Dis 2014;209:1562–72. 10.1093/infdis/jit664 24285846

[315] Marrazzo JM, Antonio M, Agnew K, Hillier SL. Distribution of genital *Lactobacillus* strains shared by female sex partners. J Infect Dis 2009;199:680–3. 10.1086/596632 19199538 PMC3291173

[316] Marrazzo JM, Fiedler TL, Srinivasan S, Extravaginal reservoirs of vaginal bacteria as risk factors for incident bacterial vaginosis. J Infect Dis 2012;205:1580–8. 10.1093/infdis/jis242 22448002 PMC3415820

[317] Mitchell C, Manhart LE, Thomas K, Fiedler T, Fredricks DN, Marrazzo J. Behavioral predictors of colonization with *Lactobacillus crispatus* or *Lactobacillus jensenii* after treatment for bacterial vaginosis: a cohort study. Infect Dis Obstet Gynecol 2012;2012:706540. Epub May 30, 2012. 10.1155/2012/706540 22693410 PMC3369434

[318] Mitchell C, Manhart LE, Thomas KK, Agnew K, Marrazzo JM. Effect of sexual activity on vaginal colonization with hydrogen peroxide-producing *Lactobacilli* and *Gardnerella vaginalis.* Sex Transm Dis 2011;38:1137–44. 10.1097/OLQ.0b013e31822e6121 22082725 PMC3217189

[319] Fethers K, Twin J, Fairley CK, Bacterial vaginosis (BV) candidate bacteria: associations with BV and behavioural practices in sexually-experienced and inexperienced women. PLoS One 2012;7:e30633. 10.1371/journal.pone.0030633 22363457 PMC3281856

[320] Bradshaw CS, Vodstrcil LA, Hocking JS, Recurrence of bacterial vaginosis is significantly associated with posttreatment sexual activities and hormonal contraceptive use. Clin Infect Dis 2013;56:777–86. 10.1093/cid/cis1030 23243173

[321] Marrazzo JM, Thomas KK, Fiedler TL, Ringwood K, Fredricks DN. Risks for acquisition of bacterial vaginosis among women who report sex with women: a cohort study. PLoS One 2010;5:e11139. 10.1371/journal.pone.0011139 20559445 PMC2886123

[322] Vodstrcil LA, Walker SM, Hocking JS, Incident bacterial vaginosis (BV) in women who have sex with women is associated with behaviors that suggest sexual transmission of BV. Clin Infect Dis 2015;60:1042–53. 10.1093/cid/ciu1130 25516188

[323] Muzny CA, Blanchard E, Taylor CM, Identification of key bacteria involved in the induction of incident bacterial vaginosis: a prospective study. J Infect Dis 2018;218:966–78. 10.1093/infdis/jiy243 29718358 PMC6093354

[324] Marrazzo JM, Thomas KK, Ringwood K. A behavioural intervention to reduce persistence of bacterial vaginosis among women who report sex with women: results of a randomised trial. Sex Transm Infect 2011;87:399–405. 10.1136/sti.2011.049213 21653935 PMC3291171

[325] Bradshaw CS, Walker J, Fairley CK, Prevalent and incident bacterial vaginosis are associated with sexual and contraceptive behaviours in young Australian women. PLoS One 2013;8:e57688. 10.1371/journal.pone.0057688 23472099 PMC3589386

[326] Poteat T, Reisner SL, Radix A. HIV epidemics among transgender women. Curr Opin HIV AIDS 2014;9:168–73. 10.1097/COH.0000000000000030 24322537 PMC5947322

[327] White Hughto JM, Reisner SL, Pachankis JE. Transgender stigma and health: a critical review of stigma determinants, mechanisms, and interventions. Soc Sci Med 2015;147:222–31. 10.1016/j.socscimed.2015.11.010 26599625 PMC4689648

[328] Radix AE, Lelutiu-Weinberger C, Gamarel KE. Satisfaction and healthcare utilization of transgender and gender non-conforming individuals in NYC: a community-based participatory study. LGBT Health 2014;1:302–8. 10.1089/lgbt.2013.0042 26789858

[329] Rapues J, Wilson EC, Packer T, Colfax GN, Raymond HF. Correlates of HIV infection among transfemales, San Francisco, 2010: results from a respondent-driven sampling study. Am J Public Health 2013;103:1485–92. 10.2105/AJPH.2012.301109 23763398 PMC4007863

[330] Sevelius JM, Patouhas E, Keatley JG, Johnson MO. Barriers and facilitators to engagement and retention in care among transgender women living with human immunodeficiency virus. Ann Behav Med 2014;47:5–16. 10.1007/s12160-013-9565-8 24317955 PMC3925767

[331] Sevelius JM. Gender affirmation: a framework for conceptualizing risk behavior among transgender women of color. Sex Roles 2013;68:675–89. 10.1007/s11199-012-0216-5 23729971 PMC3667985

[332] Reisner SL, White Hughto JM, Pardee D, Sevelius J. Syndemics and gender affirmation: HIV sexual risk in female-to-male trans masculine adults reporting sexual contact with cisgender males. Int J STD AIDS 2016;27:955–66. 10.1177/0956462415602418 26384946 PMC4798921

[333] Coleman E, Bockting W, Botzer M, Standards of care for the health of transsexual, transgender, and gender-nonconforming people, version 7. Int J Transgenderism 2012;13:165–232. 10.1080/15532739.2011.700873

[334] Winter S, Diamond M, Green J, Transgender people: health at the margins of society. Lancet 2016;388:390–400. 10.1016/S0140-6736(16)00683-8 27323925

[335] Cahill S, Singal R, Grasso C, Do ask, do tell: high levels of acceptability by patients of routine collection of sexual orientation and gender identity data in four diverse American community health centers. PLoS One 2014;9:e107104. 10.1371/journal.pone.0107104 25198577 PMC4157837

[336] Cahill SR, Baker K, Deutsch MB, Keatley J, Makadon HJ. Inclusion of sexual orientation and gender identity in Stage 3 Meaningful Use Guidelines: a huge step forward for LGBT health. LGBT Health 2016;3:100–2. 10.1089/lgbt.2015.0136 26698386

[337] Tordoff DM, Morgan J, Dombrowski JC, Golden MR, Barbee LA. Increased ascertainment of transgender and non-binary patients using a 2-step versus 1-step gender identity intake question in an STD clinic setting. Sex Transm Dis 2019;46:254–9. 10.1097/OLQ.0000000000000952 30516726

[338] Grant JM, Mottet LA, Tanis J. National transgender discrimination survey report on health and health care. Washington, DC: National Center for Transgender Equality and the National Gay and Lesbian Task Force; 2010. https://cancer-network.org/wp-content/uploads/2017/02/National_Transgender_Discrimination_Survey_Report_on_health_and_health_care.pdf

[339] Jaffee KD, Shires DA, Stroumsa D. Discrimination and delayed health care among transgender women and men: implications for improving medical education and health care delivery. Med Care 2016;54:1010–6. 10.1097/MLR.0000000000000583 27314263

[340] Glick JL, Theall KP, Andrinopoulos KM, Kendall C. The role of discrimination in care postponement among trans-feminine individuals in the U.S. National Transgender Discrimination Survey. LGBT Health 2018;5:171–9. 10.1089/lgbt.2017.0093 29589995 PMC6425922

[341] Callander D, Cook T, Cornelisse V, Trans and gender diverse people’s experiences of sexual health care are associated with sexual health screening uptake. Sex Transm Infect 2019;95(Suppl 1):A64. https://sti.bmj.com/content/sextrans/95/Suppl_1.toc.pdf

[342] Casey LS, Reisner SL, Findling MG, Discrimination in the United States: experiences of lesbian, gay, bisexual, transgender, and queer Americans. Health Serv Res 2019;54(Suppl 2):1454–66. 10.1111/1475-6773.13229 31659745 PMC6864400

[343] Potter J, Peitzmeier SM, Bernstein I, Cervical cancer screening for patients on the female-to-male spectrum: a narrative review and guide for clinicians. J Gen Intern Med 2015;30:1857–64. 10.1007/s11606-015-3462-8 26160483 PMC4636588

[344] Becasen JS, Denard CL, Mullins MM, Higa DH, Sipe TA. Estimating the prevalence of HIV and sexual behaviors among the US transgender population: a systematic review and meta-analysis, 2006–2017. Am J Public Health 2019;109:e1–8. 10.2105/AJPH.2018.304727 30496000 PMC6301428

[345] Baral SD, Poteat T, Strömdahl S, Wirtz AL, Guadamuz TE, Beyrer C. Worldwide burden of HIV in transgender women: a systematic review and meta-analysis. Lancet Infect Dis 2013;13:214–22. 10.1016/S1473-3099(12)70315-8 23260128

[346] Allan-Blitz LT, Konda KA, Calvo GM, High incidence of extra-genital gonorrheal and chlamydial infections among high-risk men who have sex with men and transgender women in Peru. Int J STD AIDS 2018;29:568–76. 10.1177/0956462417744098 29183269

[347] Hiransuthikul A, Janamnuaysook R, Sungsing T, High burden of chlamydia and gonorrhoea in pharyngeal, rectal and urethral sites among Thai transgender women: implications for anatomical site selection for the screening of STI. Sex Transm Infect 2019;95:534–9. 10.1136/sextrans-2018-053835 30982000

[348] Kojima N, Park H, Konda KA, The PICASSO Cohort: baseline characteristics of a cohort of men who have sex with men and male-to-female transgender women at high risk for syphilis infection in Lima, Peru. BMC Infect Dis 2017;17:255. 10.1186/s12879-017-2332-x 28399798 PMC5387233

[349] Pitasi MA, Kerani RP, Kohn R, Chlamydia, gonorrhea, and human immunodeficiency virus infection among transgender women and transgender men attending clinics that provide sexually transmitted disease services in six US cities: results from the Sexually Transmitted Disease Surveillance Network. Sex Transm Dis 2019;46:112–7. 10.1097/OLQ.0000000000000917 30278030 PMC11155260

[350] James S, Herman JL, Rankin S, Keisling M, Mottet L, Anafi M. The report of the 2015 US transgender survey. Washington, DC: National Center for Transgender Equality; 2016. https://transequality.org/sites/default/files/docs/usts/USTS-Full-Report-Dec17.pdf

[351] Hadj-Moussa M, Ohl DA, Kuzon WM Jr. Feminizing genital gender-confirmation surgery. Sex Med Rev 2018;6:457–468.e2. 10.1016/j.sxmr.2017.11.005 29454634

[352] Salgado CJ, Nugent A, Kuhn J, Janette M, Bahna H. Primary sigmoid vaginoplasty in transwomen: technique and outcomes. BioMed Res Int 2018;2018:4907208. 10.1155/2018/4907208 29862275 PMC5971241

[353] Radix AE, Harris AB, Belkind U, Ting J, Goldstein ZG. *Chlamydia trachomatis* infection of the neovagina in transgender women. Open Forum Infect Dis 2019;6:ofz470. 10.1093/ofid/ofz470 32395566 PMC7200138

[354] Elfering L, van der Sluis WB, Mermans JF, Buncamper ME. *Herpes neolabialis*: herpes simplex virus type 1 infection of the neolabia in a transgender woman. Int J STD AIDS 2017;28:841–3. 10.1177/0956462416685658 28632111

[355] van der Sluis WB, Buncamper ME, Bouman MB, Prevalence of neovaginal high-risk human papillomavirus among transgender women in the Netherlands. Sex Transm Dis 2016;43:503–5. 10.1097/OLQ.0000000000000476 27414682

[356] Yang C, Liu S, Xu K, Xiang Q, Yang S, Zhang X. *Condylomata gigantea* in a male transsexual. Int J STD AIDS 2009;20:211–2. 10.1258/ijsa.2008.008213 19255276

[357] Matsuki S, Kusatake K, Hein KZ, Anraku K, Morita E. *Condylomata acuminata* in the neovagina after male-to-female reassignment treated with CO_2_ laser and imiquimod. Int J STD AIDS 2015;26:509–11. 10.1177/0956462414542476 24970474

[358] Bodsworth NJ, Price R, Davies SC. Gonococcal infection of the neovagina in a male-to-female transsexual. Sex Transm Dis 1994;21:211–2. 10.1097/00007435-199407000-00005 7974071

[359] Haustein UF. Pruritus of the artificial vagina of a transsexual patient caused by gonococcal infection [German]. Hautarzt 1995;46:858–9. 10.1007/s001050050354 8567271

[360] Yamada K, Shida D, Kato T, Yoshida H, Yoshinaga S, Kanemitsu Y. Adenocarcinoma arising in sigmoid colon neovagina 53 years after construction. World J Surg Oncol 2018;16:88. 10.1186/s12957-018-1372-z 29703260 PMC5924482

[361] Hiroi H, Yasugi T, Matsumoto K, Mucinous adenocarcinoma arising in a neovagina using the sigmoid colon thirty years after operation: a case report. J Surg Oncol 2001;77:61–4. 10.1002/jso.1067 11344485

[362] Heller DS. Lesions of the neovagina—a review. J Low Genit Tract Dis 2015;19:267–70. 10.1097/LGT.0000000000000110 26111041

[363] Scheim AI, Bauer GR, Travers R. HIV-related sexual risk among transgender men who are gay, bisexual, or have sex with men. J Acquir Immune Defic Syndr 2017;74:e89–96. 10.1097/QAI.0000000000001222 27798432

[364] Sevelius J. “There’s no pamphlet for the kind of sex I have”: HIV-related risk factors and protective behaviors among transgender men who have sex with nontransgender men. J Assoc Nurses AIDS Care 2009;20:398–410. 10.1016/j.jana.2009.06.001 19732698 PMC2785444

[365] Pitasi MA, Oraka E, Clark H, Town M, DiNenno EA. HIV testing among transgender women and men—27 states and Guam, 2014–2015. MMWR Morb Mortal Wkly Rep 2017;66:883–7. 10.15585/mmwr.mm6633a3 28837547 PMC5687817

[366] Reisner SL, Perkovich B, Mimiaga MJ. A mixed methods study of the sexual health needs of New England transmen who have sex with nontransgender men. AIDS Patient Care STDS 2010;24:501–13. 10.1089/apc.2010.0059 20666586 PMC2958438

[367] Hembree WC, Cohen-Kettenis PT, Gooren L, Endocrine treatment of gender-dysphoric/gender-incongruent persons: an Endocrine Society Clinical Practice Guideline. J Clin Endocrinol Metab 2017;102:3869–903. 10.1210/jc.2017-01658 28945902

[368] Deutsch M, ed. Guidelines for the primary and gender-affirming care of transgender and gender nonbinary people. San Francisco, CA: University of California San Francisco, Department of Family and Community Medicine, Center of Excellence for Transgender Care; 2016. https://transcare.ucsf.edu/sites/transcare.ucsf.edu/files/Transgender-PGACG-6-17-16.pdf

[369] Peitzmeier SM, Reisner SL, Harigopal P, Potter J. Female-to-male patients have high prevalence of unsatisfactory Paps compared to non-transgender females: implications for cervical cancer screening. J Gen Intern Med 2014;29:778–84. 10.1007/s11606-013-2753-1 24424775 PMC4000345

[370] Peitzmeier SM, Khullar K, Reisner SL, Potter J. Pap test use is lower among female-to-male patients than non-transgender women. Am J Prev Med 2014;47:808–12. 10.1016/j.amepre.2014.07.031 25455121

[371] Reisner SL, Deutsch MB, Peitzmeier SM, Test performance and acceptability of self- versus provider-collected swabs for high-risk HPV DNA testing in female-to-male trans masculine patients. PLoS One 2018;13:e0190172. 10.1371/journal.pone.0190172 29538411 PMC5851532

[372] CDC. Surveillance for viral hepatitis—United States, 2017. Atlanta, GA: US Department of Health and Human Services, CDC; 2019. https://www.cdc.gov/hepatitis/statistics/2017surveillance/pdfs/2017HepSurveillanceRpt.pdf

[373] Kouyoumdjian FG, Leto D, John S, Henein H, Bondy S. A systematic review and meta-analysis of the prevalence of chlamydia, gonorrhoea and syphilis in incarcerated persons. Int J STD AIDS 2012;23:248–54. 10.1258/ijsa.2011.011194 22581947

[374] CDC. Evaluation of large jail STD screening programs, 2008–2009. Atlanta, GA: US Department of Health and Human Services, CDC; 2011. https://www.cdc.gov/std/publications/JailScreening2011.pdf

[375] Pathela P, Hennessy RR, Blank S, Parvez F, Franklin W, Schillinger JA. The contribution of a urine-based jail screening program to citywide male chlamydia and gonorrhea case rates in New York City. Sex Transm Dis 2009;36(Suppl):S58–61. 10.1097/OLQ.0b013e31815615bb 17989586

[376] Joesoef MR, Weinstock HS, Kent CK, ; Corrections STD Prevalence Monitoring Group. Sex and age correlates of chlamydia prevalence in adolescents and adults entering correctional facilities, 2005: implications for screening policy. Sex Transm Dis 2009;36(Suppl):S67–71. 10.1097/OLQ.0b013e31815d6de8 19125147

[377] Blank S, McDonnell DD, Rubin SR, New approaches to syphilis control. Finding opportunities for syphilis treatment and congenital syphilis prevention in a women’s correctional setting. Sex Transm Dis 1997;24:218–26. 10.1097/00007435-199704000-00006 9101633

[378] Owusu-Edusei K Jr, Gift TL, Chesson HW, Kent CK. Investigating the potential public health benefit of jail-based screening and treatment programs for chlamydia. Am J Epidemiol 2013;177:463–73. 10.1093/aje/kws240 23403986

[379] Spaulding AC, Miller J, Trigg BG, Screening for sexually transmitted diseases in short-term correctional institutions: summary of evidence reviewed for the 2010 Centers for Disease Control and Prevention Sexually Transmitted Diseases Treatment Guidelines. Sex Transm Dis 2013;40:679–84. 10.1097/01.olq.0000431353.88464.ab 23945422

[380] CDC. Male chlamydia screening consultation, March 28–29, 2006, meeting report. Atlanta, GA: US Department of Health and Human Services, CDC; 2007. https://www.cdc.gov/std/chlamydia/chlamydiascreening-males.pdf

[381] Cole J, Hotton A, Zawitz C, Kessler H. Opt-out screening for *Chlamydia trachomatis* and *Neisseria gonorrhoeae* in female detainees at Cook County jail in Chicago, IL. Sex Transm Dis 2014;41:161–5. 10.1097/OLQ.0000000000000106 24521720

[382] Shaikh RA, Simonsen KA, O’Keefe A, Comparison of opt-in versus opt-out testing for sexually transmitted infections among inmates in a county jail. J Correct Health Care 2015;21:408–16. 10.1177/1078345815600447 26285597

[383] Spaulding AC, Kim MJ, Corpening KT, Carpenter T, Watlington P, Bowden CJ. Establishing an HIV screening program led by staff nurses in a county jail. J Public Health Manag Pract 2015;21:538–45. 10.1097/PHH.0000000000000183 25427254 PMC4492874

[384] Rosen DL, Wohl DA, Golin CE, Comparing HIV case detection in prison during opt-in vs. opt-out testing policies. J Acquir Immune Defic Syndr 2016;71:e85–8. 10.1097/QAI.0000000000000889 26536318 PMC4752376

[385] Gratrix J, Smyczek P, Bertholet L, A cross-sectional evaluation of opt-in testing for sexually transmitted and blood-borne infections in three Canadian provincial correctional facilities: a missed opportunity for public health? Int J Prison Health 2019;15:273–81. 10.1108/IJPH-07-2018-0043 31329036

[386] Sutcliffe S, Newman SB, Hardick A, Gaydos CA. Prevalence and correlates of *Trichomonas vaginalis* infection among female US federal prison inmates. Sex Transm Dis 2010;37:585–90. 10.1097/OLQ.0b013e3181de4113 20803782 PMC4800006

[387] Freeman AH, Katz KA, Pandori MW, Prevalence and correlates of *Trichomonas vaginalis* among incarcerated persons assessed using a highly sensitive molecular assay. Sex Transm Dis 2010;37:165–8. 10.1097/OLQ.0b013e3181bcd3fc 20023598

[388] Sosman J, Macgowan R, Margolis A, ; Project START Biologics Study Group. Sexually transmitted infections and hepatitis in men with a history of incarceration. Sex Transm Dis 2011;38:634–9. 10.1097/OLQ.0b013e31820bc86c 21844713

[389] Javanbakht M, Stirland A, Stahlman S, Prevalence and factors associated with *Trichomonas vaginalis* infection among high-risk women in Los Angeles. Sex Transm Dis 2013;40:804–7. 10.1097/OLQ.0000000000000026 24275733 PMC4188531

[390] Nijhawan AE, Chapin KC, Salloway R, Prevalence and predictors of *Trichomonas* infection in newly incarcerated women. Sex Transm Dis 2012;39:973–8. 10.1097/OLQ.0b013e31826e8847 23191953 PMC3878291

[391] Nijhawan AE, DeLong AK, Celentano DD, The association between *Trichomonas* infection and incarceration in HIV-seropositive and at-risk HIV-seronegative women. Sex Transm Dis 2011;38:1094–100. 10.1097/OLQ.0b013e31822ea147 22082718 PMC3767476

[392] Willers DM, Peipert JF, Allsworth JE, Stein MD, Rose JS, Clarke JG. Prevalence and predictors of sexually transmitted infection among newly incarcerated females. Sex Transm Dis 2008;35:68–72. 10.1097/OLQ.0b013e318154bdb2 18090178

[393] Nijhawan AE, Salloway R, Nunn AS, Poshkus M, Clarke JG. Preventive healthcare for underserved women: results of a prison survey. J Womens Health (Larchmt) 2010;19:17–22. 10.1089/jwh.2009.1469 20088654 PMC2828235

[394] Binswanger IA, White MC, Pérez-Stable EJ, Goldenson J, Tulsky JP. Cancer screening among jail inmates: frequency, knowledge, and willingness. Am J Public Health 2005;95:1781–7. 10.2105/AJPH.2004.052498 16186455 PMC1449436

[395] Brinkley-Rubinstein L, Peterson M, Arnold T, Knowledge, interest, and anticipated barriers of pre-exposure prophylaxis uptake and adherence among gay, bisexual, and men who have sex with men who are incarcerated. PLoS One 2018;13:e0205593. 10.1371/journal.pone.0205593 30532275 PMC6286000

[396] Brinkley-Rubinstein L, Dauria E, Tolou-Shams M, The path to implementation of HIV pre-exposure prophylaxis for people involved in criminal justice systems. Curr HIV/AIDS Rep 2018;15:93–5. 10.1007/s11904-018-0389-9 29516265 PMC5884709

[397] Morrow KM; Project START Study Group. HIV, STD, and hepatitis risk behaviors of young men before and after incarceration. AIDS Care 2009;21:235–43. 10.1080/09540120802017586 19229694

[398] Bryan AD, Magnan RE, Gillman AS, Effect of including alcohol and cannabis content in a sexual risk-reduction intervention on the incidence of sexually transmitted infections in adolescents: a cluster randomized clinical trial. JAMA Pediatr 2018;172:e175621. 10.1001/jamapediatrics.2017.5621 29435591 PMC5875326

[399] DiClemente RJ, Davis TL, Swartzendruber A, Efficacy of an HIV/STI sexual risk-reduction intervention for African American adolescent girls in juvenile detention centers: a randomized controlled trial. Women Health 2014;54:726–49. 10.1080/03630242.2014.932893 25190056 PMC4224621

[400] Fogel CI, Crandell JL, Neevel AM, Efficacy of an adapted HIV and sexually transmitted infection prevention intervention for incarcerated women: a randomized controlled trial. Am J Public Health 2015;105:802–9. 10.2105/AJPH.2014.302105 25211714 PMC4358199

[401] Son J, Miller WM, Tossone K, Butcher F, Kuo K. The effect of interprofessional student-led reproductive health education on youths in juvenile detention. J Pediatr Adolesc Gynecol 2017;30:370–5. 10.1016/j.jpag.2016.11.002 27871918

[402] Costumbrado J, Stirland A, Cox G, Implementation of a hepatitis A/B vaccination program using an accelerated schedule among high-risk inmates, Los Angeles County Jail, 2007–2010. Vaccine 2012;30:6878–82. 10.1016/j.vaccine.2012.09.006 22989688

[403] Allison M, Musser B, Satterwhite C, Ault K, Kelly P, Ramaswamy M. Human papillomavirus vaccine knowledge and intention among adult inmates in Kansas, 2016–2017. Am J Public Health 2018;108:1000–2. 10.2105/AJPH.2018.304499 29927651 PMC6050866

[404] Lucas KD, Miller JL, Eckert V, Horne RL, Samuel MC, Mohle-Boetani JC. Risk, feasibility, and cost evaluation of a prisoner condom access pilot program in one California state prison. J Correct Health Care 2014;20:184–94. 10.1177/1078345814530869 24934836

[405] Scott N, McBryde E, Kirwan A, Stoové M. Modelling the impact of condom distribution on the incidence and prevalence of sexually transmitted infections in an adult male prison system. PLoS One 2015;10:e0144869. 10.1371/journal.pone.0144869 26658518 PMC4691199

[406] Schacker T, Collier AC, Hughes J, Shea T, Corey L. Clinical and epidemiologic features of primary HIV infection. Ann Intern Med 1996;125:257–64. 10.7326/0003-4819-125-4-199608150-00001 8678387

[407] Henn A, Flateau C, Gallien S. Primary HIV infection: clinical presentation, testing, and treatment. Curr Infect Dis Rep 2017;19:37. 10.1007/s11908-017-0588-3 28884279

[408] Robb ML, Eller LA, Kibuuka H, ; RV 217 Study Team. Prospective study of acute HIV-1 infection in adults in East Africa and Thailand. N Engl J Med 2016;374:2120–30. 10.1056/NEJMoa1508952 27192360 PMC5111628

[409] Hoenigl M, Green N, Camacho M, Signs or symptoms of acute HIV infection in a cohort undergoing community-based screening. Emerg Infect Dis 2016;22:532–4. 10.3201/eid2203.151607 26890854 PMC4766914

[410] Legarth RA, Ahlström MG, Kronborg G, Long-term mortality in HIV-infected individuals 50 years or older: a nationwide, population-based cohort study. J Acquir Immune Defic Syndr 2016;71:213–8. 10.1097/QAI.0000000000000825 26334734

[411] Marcus JL, Chao CR, Leyden WA, Narrowing the gap in life expectancy between HIV-infected and HIV-uninfected individuals with access to care. J Acquir Immune Defic Syndr 2016;73:39–46. 10.1097/QAI.0000000000001014 27028501 PMC5427712

[412] Cohen MS, Chen YQ, McCauley M, ; HPTN 052 Study Team. Antiretroviral therapy for the prevention of HIV-1 transmission. N Engl J Med 2016;375:830–9. 10.1056/NEJMoa1600693 27424812 PMC5049503

[413] Saag MS, Benson CA, Gandhi RT, Antiretroviral drugs for treatment and prevention of HIV infection in adults: 2018 recommendations of the International Antiviral Society-USA Panel. JAMA 2018;320:379–96. 10.1001/jama.2018.8431 30043070 PMC6415748

[414] Seth P, Wang G, Sizemore E, Hogben M. HIV testing and HIV service delivery to populations at high risk attending sexually transmitted disease clinics in the United States, 2011–2013. Am J Public Health 2015;105:2374–81. 10.2105/AJPH.2015.302778 26378854 PMC4605158

[415] Benton S, Smith J, Wang F, Heitgerd J, Belcher L, Patel H. HIV testing, diagnosis, and linkage to care among persons tested in select CDC-funded health care and non-health care settings, 2012–2017. Presented at the National HIV Prevention Conference, Atlanta, GA: March 18–21, 2019.

[416] Pathela P, Braunstein SL, Schillinger JA, Shepard C, Sweeney M, Blank S. Men who have sex with men have a 140-fold higher risk for newly diagnosed HIV and syphilis compared with heterosexual men in New York City. J Acquir Immune Defic Syndr 2011;58:408–16. 10.1097/QAI.0b013e318230e1ca 21857351

[417] Chou R, Selph S, Dana T, Screening for HIV: systematic review to update the 2005 U.S. Preventive Services Task Force recommendation. Ann Intern Med 2012;157:706–18. 10.7326/0003-4819-157-10-201211200-00007 23165662

[418] Branson BM, Handsfield HH, Lampe MA, ; CDC. Revised recommendations for HIV testing of adults, adolescents, and pregnant women in health-care settings. MMWR Recomm Rep 2006;55(No. RR-14). 16988643

[419] DiNenno EA, Prejean J, Irwin K, Recommendations for HIV screening of gay, bisexual, and other men who have sex with men—United States, 2017. MMWR Morb Mortal Wkly Rep 2017;66:830–2. 10.15585/mmwr.mm6631a3 28796758 PMC5687782

[420] CDC. HIV-2 infection surveillance—United States, 1987–2009. MMWR Morb Mortal Wkly Rep 2011;60:985–8. 21796096

[421] Wawer MJ, Gray RH, Sewankambo NK, Rates of HIV-1 transmission per coital act, by stage of HIV-1 infection, in Rakai, Uganda. J Infect Dis 2005;191:1403–9. 10.1086/429411 15809897

[422] Pilcher CD, Eron JJ Jr, Vemazza PL, Sexual transmission during the incubation period of primary HIV infection. JAMA 2001;286:1713–4. 10.1001/jama.286.14.1713 11594895

[423] Calabrese SK, Mayer KH. Providers should discuss U=U with all patients living with HIV. Lancet HIV 2019;6:e211–3. 10.1016/S2352-3018(19)30030-X 30772420

[424] Gilbert P, Ciccarone D, Gansky SA, Interactive “Video Doctor” counseling reduces drug and sexual risk behaviors among HIV-positive patients in diverse outpatient settings. PLoS One 2008;3:e1988. 10.1371/journal.pone.0001988 18431475 PMC2292251

[425] Aberg JA, Gallant JE, Ghanem KG, Emmanuel P, Zingman BS, Horberg MA; Infectious Diseases Society of America. Primary care guidelines for the management of persons infected with HIV: 2013 update by the HIV medicine association of the Infectious Diseases Society of America. Clin Infect Dis 2014;58:e1–34. 10.1093/cid/cit665 24235263

[426] DiCarlo RP, Martin DH. The clinical diagnosis of genital ulcer disease in men. Clin Infect Dis 1997;25:292–8. 10.1086/514548 9332527

[427] Lockett AE, Dance DA, Mabey DC, Drasar BS. Serum-free media for isolation of *Haemophilus ducreyi*. Lancet 1991;338:326. 10.1016/0140-6736(91)90473-3 1677152

[428] Lewis DA, Mitjà O. *Haemophilus ducreyi*: from sexually transmitted infection to skin ulcer pathogen. Curr Opin Infect Dis 2016;29:52–7. 10.1097/QCO.0000000000000226 26658654

[429] Romero L, Huerfano C, Grillo-Ardila CF. Macrolides for treatment of *Haemophilus ducreyi* infection in sexually active adults. Cochrane Database Syst Rev 2017;12:CD012492. 10.1002/14651858.CD012492.pub2 29226307 PMC6486275

[430] Jessamine PG, Plummer FA, Ndinya Achola JO, Human immunodeficiency virus, genital ulcers and the male foreskin: synergism in HIV-1 transmission. Scand J Infect Dis Suppl 1990;69:181–6. 2263893

[431] Briggs GC. Drugs in pregnancy and lactation: a reference guide to fetal and neonatal risk. 11th ed. Philadelphia, PA: Wolters Kluwer; 2017.

[432] Lewis DA. Epidemiology, clinical features, diagnosis and treatment of *Haemophilus ducreyi*—a disappearing pathogen? Expert Rev Anti Infect Ther 2014;12:687–96. 10.1586/14787210.2014.892414 24597521

[433] Mitjà O, Lukehart SA, Pokowas G, *Haemophilus ducreyi* as a cause of skin ulcers in children from a yaws-endemic area of Papua New Guinea: a prospective cohort study. Lancet Glob Health 2014;2:e235–41. 10.1016/S2214-109X(14)70019-1 25103064

[434] Marks M, Chi KH, Vahi V, *Haemophilus ducreyi* associated with skin ulcers among children, Solomon Islands. Emerg Infect Dis 2014;20:1705–7. 10.3201/eid2010.140573 25271477 PMC4193279

[435] Ghinai R, El-Duah P, Chi KH, A cross-sectional study of ‘yaws’ in districts of Ghana which have previously undertaken azithromycin mass drug administration for trachoma control. PLoS Negl Trop Dis 2015;9:e0003496. 10.1371/journal.pntd.0003496 25632942 PMC4310597

[436] McQuillan G, Kruszon-Moran D, Flagg EW, Paulose-Ram R. Prevalence of herpes simplex virus type 1 and type 2 in persons aged 14–49: United States, 2015–2016. NCHS Data Brief 2018;304:1–8. 29442994

[437] Ryder N, Jin F, McNulty AM, Grulich AE, Donovan B. Increasing role of herpes simplex virus type 1 in first-episode anogenital herpes in heterosexual women and younger men who have sex with men, 1992–2006. Sex Transm Infect 2009;85:416–9. 10.1136/sti.2008.033902 19273479

[438] Roberts CM, Pfister JR, Spear SJ. Increasing proportion of herpes simplex virus type 1 as a cause of genital herpes infection in college students. Sex Transm Dis 2003;30:797–800. 10.1097/01.OLQ.0000092387.58746.C7 14520181

[439] Benedetti J, Corey L, Ashley R. Recurrence rates in genital herpes after symptomatic first-episode infection. Ann Intern Med 1994;121:847–54. 10.7326/0003-4819-121-11-199412010-00004 7978697

[440] Engelberg R, Carrell D, Krantz E, Corey L, Wald A. Natural history of genital herpes simplex virus type 1 infection. Sex Transm Dis 2003;30:174–7. 10.1097/00007435-200302000-00015 12567178

[441] Masese L, Baeten JM, Richardson BA, Changes in the contribution of genital tract infections to HIV acquisition among Kenyan high-risk women from 1993 to 2012. AIDS 2015;29:1077–85. 10.1097/QAD.0000000000000646 26125141 PMC4576156

[442] Sam SS, Caliendo AM, Ingersoll J, Abdul-Ali D, Kraft CS. Performance evaluation of the Aptima HSV-1 and 2 assay for the detection of HSV in cutaneous and mucocutaneous lesion specimens. J Clin Virol 2018;99-100:1–4. 10.1016/j.jcv.2017.12.006 29253834

[443] Wald A, Huang ML, Carrell D, Selke S, Corey L. Polymerase chain reaction for detection of herpes simplex virus (HSV) DNA on mucosal surfaces: comparison with HSV isolation in cell culture. J Infect Dis 2003;188:1345–51. 10.1086/379043 14593592

[444] Van Der Pol B, Warren T, Taylor SN, Type-specific identification of anogenital herpes simplex virus infections by use of a commercially available nucleic acid amplification test. J Clin Microbiol 2012;50:3466–71. 10.1128/JCM.01685-12 22875892 PMC3486267

[445] Binnicker MJ, Espy MJ, Duresko B, Irish C, Mandrekar J. Automated processing, extraction and detection of herpes simplex virus types 1 and 2: a comparative evaluation of three commercial platforms using clinical specimens. J Clin Virol 2017;89:30–3. 10.1016/j.jcv.2017.02.006 28226272

[446] Teo JW, Chiang D, Jureen R, Lin RT. Clinical evaluation of a helicase-dependant amplification (HDA)-based commercial assay for the simultaneous detection of HSV-1 and HSV-2. Diagn Microbiol Infect Dis 2015;83:261–2. 10.1016/j.diagmicrobio.2015.07.018 26302856

[447] Gitman MR, Ferguson D, Landry ML. Comparison of Simplexa HSV 1 & 2 PCR with culture, immunofluorescence, and laboratory-developed TaqMan PCR for detection of herpes simplex virus in swab specimens. J Clin Microbiol 2013;51:3765–9. 10.1128/JCM.01413-13 24006008 PMC3889765

[448] Corey L, Holmes KK. Genital herpes simplex virus infections: current concepts in diagnosis, therapy, and prevention. Ann Intern Med 1983;98:973–83. 10.7326/0003-4819-98-6-958 6344713

[449] Caviness AC, Oelze LL, Saz UE, Greer JM, Demmler-Harrison GJ. Direct immunofluorescence assay compared to cell culture for the diagnosis of mucocutaneous herpes simplex virus infections in children. J Clin Virol 2010;49:58–60. 10.1016/j.jcv.2010.06.006 20620099

[450] Song B, Dwyer DE, Mindel A. HSV type specific serology in sexual health clinics: use, benefits, and who gets tested. Sex Transm Infect 2004;80:113–7. 10.1136/sti.2003.006783 15054171 PMC1744793

[451] Whittington WL, Celum CL, Cent A, Ashley RL. Use of a glycoprotein G-based type-specific assay to detect antibodies to herpes simplex virus type 2 among persons attending sexually transmitted disease clinics. Sex Transm Dis 2001;28:99–104. 10.1097/00007435-200102000-00007 11234793

[452] Zimet GD, Rosenthal SL, Fortenberry JD, Factors predicting the acceptance of herpes simplex virus type 2 antibody testing among adolescents and young adults. Sex Transm Dis 2004;31:665–9. 10.1097/01.olq.0000143089.77493.c2 15502674

[453] Turner KR, Wong EH, Kent CK, Klausner JD. Serologic herpes testing in the real world: validation of new type-specific serologic herpes simplex virus tests in a public health laboratory. Sex Transm Dis 2002;29:422–5. 10.1097/00007435-200207000-00011 12170133

[454] Eing BR, Lippelt L, Lorentzen EU, Evaluation of confirmatory strategies for detection of type-specific antibodies against herpes simplex virus type 2. J Clin Microbiol 2002;40:407–13. 10.1128/JCM.40.2.407-413.2002 11825950 PMC153348

[455] Golden MR, Ashley-Morrow R, Swenson P, Hogrefe WR, Handsfield HH, Wald A. Herpes simplex virus type 2 (HSV-2) Western blot confirmatory testing among men testing positive for HSV-2 using the focus enzyme-linked immunosorbent assay in a sexually transmitted disease clinic. Sex Transm Dis 2005;32:771–7. 10.1097/01.olq.0000175377.88358.f3 16314775

[456] Morrow RA, Friedrich D, Meier A, Corey L. Use of “biokit HSV-2 Rapid Assay” to improve the positive predictive value of Focus HerpeSelect HSV-2 ELISA. BMC Infect Dis 2005;5:84. 10.1186/1471-2334-5-84 16225691 PMC1276011

[457] Ngo TD, Laeyendecker O, La H, Hogrefe W, Morrow RA, Quinn TC. Use of commercial enzyme immunoassays to detect antibodies to the herpes simplex virus type 2 glycoprotein G in a low-risk population in Hanoi, Vietnam. Clin Vaccine Immunol 2008;15:382–4. 10.1128/CVI.00437-06 18077617 PMC2238064

[458] Agyemang E, Le QA, Warren T, Performance of commercial enzyme-linked immunoassays for diagnosis of herpes simplex virus-1 and herpes simplex virus-2 infection in a clinical setting. Sex Transm Dis 2017;44:763–7. 10.1097/OLQ.0000000000000689 28876290

[459] Morrow R, Friedrich D. Performance of a novel test for IgM and IgG antibodies in subjects with culture-documented genital herpes simplex virus-1 or -2 infection. Clin Microbiol Infect 2006;12:463–9. 10.1111/j.1469-0691.2006.01370.x 16643524

[460] Ameli N, Bacchetti P, Morrow RA, Herpes simplex virus infection in women in the WIHS: epidemiology and effect of antiretroviral therapy on clinical manifestations. AIDS 2006;20:1051–8. 10.1097/01.aids.0000222078.75867.77 16603858

[461] Bradley H, Markowitz LE, Gibson T, McQuillan GM. Seroprevalence of herpes simplex virus types 1 and 2—United States, 1999–2010. J Infect Dis 2014;209:325–33. 10.1093/infdis/jit458 24136792

[462] Bernstein DI, Bellamy AR, Hook EW 3rd, Epidemiology, clinical presentation, and antibody response to primary infection with herpes simplex virus type 1 and type 2 in young women. Clin Infect Dis 2013;56:344–51. 10.1093/cid/cis891 23087395 PMC3540038

[463] Leone PA, Trottier S, Miller JM. Valacyclovir for episodic treatment of genital herpes: a shorter 3-day treatment course compared with 5-day treatment. Clin Infect Dis 2002;34:958–62. 10.1086/339326 11880962

[464] Wald A, Carrell D, Remington M, Kexel E, Zeh J, Corey L. Two-day regimen of acyclovir for treatment of recurrent genital herpes simplex virus type 2 infection. Clin Infect Dis 2002;34:944–8. 10.1086/339325 11880960

[465] Aoki FY, Tyring S, Diaz-Mitoma F, Gross G, Gao J, Hamed K. Single-day, patient-initiated famciclovir therapy for recurrent genital herpes: a randomized, double-blind, placebo-controlled trial. Clin Infect Dis 2006;42:8–13. 10.1086/498521 16323085

[466] Chosidow O, Drouault Y, Leconte-Veyriac F, Famciclovir vs. aciclovir in immunocompetent patients with recurrent genital herpes infections: a parallel-groups, randomized, double-blind clinical trial. Br J Dermatol 2001;144:818–24. 10.1046/j.1365-2133.2001.04139.x 11298543

[467] Bodsworth NJ, Crooks RJ, Borelli S, ; International Valaciclovir HSV Study Group. Valaciclovir versus aciclovir in patient initiated treatment of recurrent genital herpes: a randomised, double blind clinical trial. Genitourin Med 1997;73:110–6. 9215092 10.1136/sti.73.2.110 PMC1195783

[468] Fife KH, Barbarash RA, Rudolph T, Degregorio B, Roth R; The Valaciclovir International Herpes Simplex Virus Study Group. Valaciclovir versus acyclovir in the treatment of first-episode genital herpes infection. Results of an international, multicenter, double-blind, randomized clinical trial. Sex Transm Dis 1997;24:481–6. 10.1097/00007435-199709000-00007 9293612

[469] Diaz-Mitoma F, Sibbald RG, Shafran SD, Boon R, Saltzman RL; Collaborative Famciclovir Genital Herpes Research Group. Oral famciclovir for the suppression of recurrent genital herpes: a randomized controlled trial. JAMA 1998;280:887–92. 10.1001/jama.280.10.887 9739972

[470] Mertz GJ, Loveless MO, Levin MJ, Collaborative Famciclovir Genital Herpes Research Group. Oral famciclovir for suppression of recurrent genital herpes simplex virus infection in women. A multicenter, double-blind, placebo-controlled trial. Arch Intern Med 1997;157:343–9. 10.1001/archinte.1997.00440240109016 9040303

[471] Reitano M, Tyring S, Lang W, ; International Valaciclovir HSV Study Group. Valaciclovir for the suppression of recurrent genital herpes simplex virus infection: a large-scale dose range-finding study. J Infect Dis 1998;178:603–10. 10.1086/515385 9728526

[472] Romanowski B, Marina RB, Roberts JN; Valtrex HS230017 Study Group. Patients’ preference of valacyclovir once-daily suppressive therapy versus twice-daily episodic therapy for recurrent genital herpes: a randomized study. Sex Transm Dis 2003;30:226–31. 10.1097/00007435-200303000-00010 12616141

[473] Corey L, Wald A, Patel R, ; Valacyclovir HSV Transmission Study Group. Once-daily valacyclovir to reduce the risk of transmission of genital herpes. N Engl J Med 2004;350:11–20. 10.1056/NEJMoa035144 14702423

[474] Tyring SK, Baker D, Snowden W. Valacyclovir for herpes simplex virus infection: long-term safety and sustained efficacy after 20 years’ experience with acyclovir. J Infect Dis 2002;186(Suppl 1):S40–6. 10.1086/342966 12353186

[475] Bartlett BL, Tyring SK, Fife K, Famciclovir treatment options for patients with frequent outbreaks of recurrent genital herpes: the RELIEF trial. J Clin Virol 2008;43:190–5. 10.1016/j.jcv.2008.06.004 18621575

[476] Tronstein E, Johnston C, Huang ML, Genital shedding of herpes simplex virus among symptomatic and asymptomatic persons with HSV-2 infection. JAMA 2011;305:1441–9. 10.1001/jama.2011.420 21486977 PMC3144252

[477] Bender Ignacio RA, Perti T, Magaret AS, Oral and vaginal tenofovir for genital herpes simplex virus type 2 shedding in immunocompetent women: a double-blind, randomized, cross-over trial. J Infect Dis 2015;212:1949–56. 10.1093/infdis/jiv317 26044291 PMC4655855

[478] Wald A, Selke S, Warren T, Comparative efficacy of famciclovir and valacyclovir for suppression of recurrent genital herpes and viral shedding. Sex Transm Dis 2006;33:529–33. 10.1097/01.olq.0000204723.15765.91 16540883

[479] Johnston C, Magaret A, Stern M, Natural history of genital and oral herpes simplex virus-1 (HSV-1) shedding after first episode genital HSV-1 infection. Sex Transm Infect 2019;95:A42.

[480] Tang YW, Cleavinger PJ, Li H, Mitchell PS, Smith TF, Persing DH. Analysis of candidate-host immunogenetic determinants in herpes simplex virus-associated Mollaret’s meningitis. Clin Infect Dis 2000;30:176–8. 10.1086/313616 10619748

[481] Shalabi M, Whitley RJ. Recurrent benign lymphocytic meningitis. Clin Infect Dis 2006;43:1194–7. 10.1086/508281 17029141

[482] Landry ML, Greenwold J, Vikram HR. Herpes simplex type-2 meningitis: presentation and lack of standardized therapy. Am J Med 2009;122:688–91. 10.1016/j.amjmed.2009.02.017 19559173

[483] Aurelius E, Franzen-Röhl E, Glimåker M, ; HSV-2 Meningitis Study Group. Long-term valacyclovir suppressive treatment after herpes simplex virus type 2 meningitis: a double-blind, randomized controlled trial. Clin Infect Dis 2012;54:1304–13. 10.1093/cid/cis031 22460966

[484] Magawa S, Tanaka H, Furuhashi F, A literature review of herpes simplex virus hepatitis in pregnancy. J Matern Fetal Neonatal Med 2020;33:1774–9. 10.1080/14767058.2018.1527311 30235956

[485] Masadeh M, Shen H, Lee Y, A fatal case of herpes simplex virus hepatitis in a pregnant patient. Intractable Rare Dis Res 2017;6:124–7. 10.5582/irdr.2017.01013 28580213 PMC5451744

[486] Martin ET, Krantz E, Gottlieb SL, A pooled analysis of the effect of condoms in preventing HSV-2 acquisition. Arch Intern Med 2009;169:1233–40. 10.1001/archinternmed.2009.177 19597073 PMC2860381

[487] Wald A, Langenberg AG, Krantz E, The relationship between condom use and herpes simplex virus acquisition. Ann Intern Med 2005;143:707–13. 10.7326/0003-4819-143-10-200511150-00007 16287791

[488] Wald A, Langenberg AG, Link K, Effect of condoms on reducing the transmission of herpes simplex virus type 2 from men to women. JAMA 2001;285:3100–6. 10.1001/jama.285.24.3100 11427138

[489] Magaret AS, Mujugira A, Hughes JP, ; Partners in Prevention HSV/HIV Transmission Study Team. Effect of condom use on per-act HSV-2 transmission risk in HIV-1, HSV-2-discordant couples. Clin Infect Dis 2016;62:456–61. 26578538 10.1093/cid/civ908 PMC4725379

[490] Mehta SD, Moses S, Agot K, Medical male circumcision and herpes simplex virus 2 acquisition: posttrial surveillance in Kisumu, Kenya. J Infect Dis 2013;208:1869–76. 10.1093/infdis/jit371 23901089 PMC3814838

[491] Grund JM, Bryant TS, Jackson I, Association between male circumcision and women’s biomedical health outcomes: a systematic review. Lancet Glob Health 2017;5:e1113–22. 10.1016/S2214-109X(17)30369-8 29025633 PMC5728090

[492] Celum C, Morrow RA, Donnell D, ; Partners PrEP Study Team. Daily oral tenofovir and emtricitabine-tenofovir preexposure prophylaxis reduces herpes simplex virus type 2 acquisition among heterosexual HIV-1-uninfected men and women: a subgroup analysis of a randomized trial. Ann Intern Med 2014;161:11–9. 10.7326/M13-2471 24979446

[493] Abdool Karim SS, Abdool Karim Q, Gengiah TN. Tenofovir gel to prevent HSV-2 infection. N Engl J Med 2015;373:1980–1. 10.1056/NEJMoa1410649 26559584

[494] Marcus JL, Glidden DV, McMahan V, Daily oral emtricitabine/tenofovir preexposure prophylaxis and herpes simplex virus type 2 among men who have sex with men. PLoS One 2014;9:e91513. 10.1371/journal.pone.0091513 24637511 PMC3956614

[495] Celum C, Hong T, Cent A, ; ACTG PEARLS/A5175 Team. Herpes simplex virus type 2 acquisition among HIV-1-infected adults treated with tenofovir disoproxyl fumarate as part of combination antiretroviral therapy: results from the ACTG A5175 PEARLS Study. J Infect Dis 2017;215:907–10. 10.1093/infdis/jix029 28453835 PMC5406847

[496] Gilbert LK, Wyand F. Genital herpes education and counselling: testing a one-page ‘FAQ’ intervention. Herpes 2009;15:51–6. 19306603

[497] Rosenthal SL, Zimet GD, Leichliter JS, The psychosocial impact of serological diagnosis of asymptomatic herpes simplex virus type 2 infection. Sex Transm Infect 2006;82:154–7, discussion 157–8. 10.1136/sti.2005.016311 16581745 PMC2564691

[498] Miyai T, Turner KR, Kent CK, Klausner J. The psychosocial impact of testing individuals with no history of genital herpes for herpes simplex virus type 2. Sex Transm Dis 2004;31:517–21. 10.1097/01.olq.0000137901.71284.6b 15480111

[499] Ross K, Johnston C, Wald A. Herpes simplex virus type 2 serological testing and psychosocial harm: a systematic review. Sex Transm Infect 2011;87:594–600. 10.1136/sextrans-2011-050099 21903980

[500] Henry RE, Wegmann JA, Hartle JE, Christopher GW. Successful oral acyclovir desensitization. Ann Allergy 1993;70:386–8. 8498729

[501] Leeyaphan C, Surawan TM, Chirachanakul P, Clinical characteristics of hypertrophic herpes simplex genitalis and treatment outcomes of imiquimod: a retrospective observational study. Int J Infect Dis 2015;33:165–70. 10.1016/j.ijid.2015.02.002 25660091

[502] Keller MJ, Huber A, Espinoza L, Impact of herpes simplex virus type 2 and human immunodeficiency virus dual infection on female genital tract mucosal immunity and the vaginal microbiome. J Infect Dis 2019;220:852–61. 10.1093/infdis/jiz203 31111902 PMC6667798

[503] Posavad CM, Wald A, Kuntz S, Frequent reactivation of herpes simplex virus among HIV-1-infected patients treated with highly active antiretroviral therapy. J Infect Dis 2004;190:693–6. 10.1086/422755 15272395

[504] Tobian AA, Grabowski MK, Serwadda D, ; Rakai Health Sciences Program. Reactivation of herpes simplex virus type 2 after initiation of antiretroviral therapy. J Infect Dis 2013;208:839–46. 10.1093/infdis/jit252 23812240 PMC3733512

[505] Mujugira A, Magaret AS, Celum C, ; Partners in Prevention HSV/HIV Transmission Study Team. Daily acyclovir to decrease herpes simplex virus type 2 (HSV-2) transmission from HSV-2/HIV-1 coinfected persons: a randomized controlled trial. J Infect Dis 2013;208:1366–74. 10.1093/infdis/jit333 23901094 PMC3789565

[506] Van Wagoner N, Geisler WM, Bachmann LH, Hook EW. The effect of valacyclovir on HIV and HSV-2 in HIV-infected persons on antiretroviral therapy with previously unrecognised HSV-2. Int J STD AIDS 2015;26:574–81. 10.1177/0956462414546504 25147236

[507] Reyes M, Shaik NS, Graber JM, ; Task Force on Herpes Simplex Virus Resistance. Acyclovir-resistant genital herpes among persons attending sexually transmitted disease and human immunodeficiency virus clinics. Arch Intern Med 2003;163:76–80. 10.1001/archinte.163.1.76 12523920

[508] Safrin S, Crumpacker C, Chatis P, ; The AIDS Clinical Trials Group. A controlled trial comparing foscarnet with vidarabine for acyclovir-resistant mucocutaneous herpes simplex in the acquired immunodeficiency syndrome. N Engl J Med 1991;325:551–5. 10.1056/NEJM199108223250805 1649971

[509] Levin MJ, Bacon TH, Leary JJ. Resistance of herpes simplex virus infections to nucleoside analogues in HIV-infected patients. Clin Infect Dis 2004;39(Suppl 5):S248–57. 10.1086/422364 15494896

[510] Tandon S, Singh J, Sinha S, Sharma DP. Recalcitrant hypertrophic herpes genitalis in HIV-infected patient successfully treated with topical imiquimod. Dermatol Ther (Heidelb) 2017;30:e12479. 10.1111/dth.12479 28261899

[511] Perkins N, Nisbet M, Thomas M. Topical imiquimod treatment of aciclovir-resistant herpes simplex disease: case series and literature review. Sex Transm Infect 2011;87:292–5. 10.1136/sti.2010.047431 21406577

[512] McElhiney LF. Topical cidofovir for treatment of resistant viral infections. Int J Pharm Compd 2006;10:324–8. 23974309

[513] Erard V, Wald A, Corey L, Leisenring WM, Boeckh M. Use of long-term suppressive acyclovir after hematopoietic stem-cell transplantation: impact on herpes simplex virus (HSV) disease and drug-resistant HSV disease. J Infect Dis 2007;196:266–70. 10.1086/518938 17570114

[514] Brown ZA, Selke S, Zeh J, The acquisition of herpes simplex virus during pregnancy. N Engl J Med 1997;337:509–15. 10.1056/NEJM199708213370801 9262493

[515] Pinninti SG, Kimberlin DW. Maternal and neonatal herpes simplex virus infections. Am J Perinatol 2013;30:113–9. 10.1055/s-0032-1332802 23303485

[516] Brown ZA, Benedetti J, Ashley R, Neonatal herpes simplex virus infection in relation to asymptomatic maternal infection at the time of labor. N Engl J Med 1991;324:1247–52. 10.1056/NEJM199105023241804 1849612

[517] Brown ZA, Wald A, Morrow RA, Selke S, Zeh J, Corey L. Effect of serologic status and cesarean delivery on transmission rates of herpes simplex virus from mother to infant. JAMA 2003;289:203–9. 10.1001/jama.289.2.203 12517231

[518] Ahrens KA, Anderka MT, Feldkamp ML, Canfield MA, Mitchell AA, Werler MM; National Birth Defects Prevention Study. Antiherpetic medication use and the risk of gastroschisis: findings from the National Birth Defects Prevention Study, 1997–2007. Paediatr Perinat Epidemiol 2013;27:340–5. 10.1111/ppe.12064 23772935 PMC3690801

[519] Stone KM, Reiff-Eldridge R, White AD, Pregnancy outcomes following systemic prenatal acyclovir exposure: conclusions from the international acyclovir pregnancy registry, 1984–1999. Birth Defects Res A Clin Mol Teratol 2004;70:201–7. 10.1002/bdra.20013 15108247

[520] Pasternak B, Hviid A. Use of acyclovir, valacyclovir, and famciclovir in the first trimester of pregnancy and the risk of birth defects. JAMA 2010;304:859–66. 10.1001/jama.2010.1206 20736469

[521] Sheffield JS, Sánchez PJ, Wendel GD Jr, Placental histopathology of congenital syphilis. Obstet Gynecol 2002;100:126–33. 12100814 10.1016/s0029-7844(02)02010-0

[522] Watts DH, Brown ZA, Money D, A double-blind, randomized, placebo-controlled trial of acyclovir in late pregnancy for the reduction of herpes simplex virus shedding and cesarean delivery. Am J Obstet Gynecol 2003;188:836–43. 10.1067/mob.2003.185 12634667

[523] Scott LL, Hollier LM, McIntire D, Sanchez PJ, Jackson GL, Wendel GD Jr. Acyclovir suppression to prevent recurrent genital herpes at delivery. Infect Dis Obstet Gynecol 2002;10:71–7. 10.1155/S1064744902000054 12530483 PMC1784606

[524] Pinninti SG, Angara R, Feja KN, Neonatal herpes disease following maternal antenatal antiviral suppressive therapy: a multicenter case series. J Pediatr 2012;161:134–8.e1-3. 10.1016/j.jpeds.2011.12.053 22336576

[525] ACOG Committee on Practice Bulletins. ACOG Practice Bulletin. Clinical management guidelines for obstetrician-gynecologists. No. 82 June 2007. Management of herpes in pregnancy. Obstet Gynecol 2007;109:1489–98. 10.1097/01.AOG.0000263902.31953.3e 17569194

[526] Winer RL, Hughes JP, Feng Q, Early natural history of incident, type-specific human papillomavirus infections in newly sexually active young women. Cancer Epidemiol Biomarkers Prev 2011;20:699–707. 10.1158/1055-9965.EPI-10-1108 21173170 PMC3078690

[527] Ahmed N, Pillay A, Lawler M, Bobat R, Archary M. Donovanosis causing lymphadenitis, mastoiditis, and meningitis in a child. Lancet 2015;385:2644. 10.1016/S0140-6736(15)60992-8 26122163

[528] Arora AK, Kumaran MS, Narang T, Saikia UN, Handa S. Donovanosis and squamous cell carcinoma: the relationship conundrum! Int J STD AIDS 2017;28:411–4. 10.1177/0956462416665996 27535727

[529] Liverani CA, Lattuada D, Mangano S, Hypertrophic donavanosis in a young pregnant woman. J Pediatr Adolesc Gynecol 2012;25:e81–3. 10.1016/j.jpag.2011.10.002 22840941

[530] Magalhães BM, Veasey JV, Mayor SAS, Lellis RF. Donovanosis in a child victim of sexual abuse: response to doxycycline treatment. An Bras Dermatol 2018;93:592–4. 10.1590/abd1806-4841.20187948 30066775 PMC6063095

[531] Marfatia YS, Menon DS, Jose S, Patel BK. Nonhealing genital ulcer in AIDS: a diagnostic dilemma! Indian J Sex Transm Dis AIDS 2016;37:197–200. 10.4103/0253-7184.192130 27890958 PMC5111309

[532] Narang T, Kanwar AJ. Genital elephantiasis due to donovanosis: forgotten but not gone yet. Int J STD AIDS 2012;23:835–6. 10.1258/ijsa.2012.012096 23155109

[533] Pilani A, Vora R, Anjaneyan G. Granuloma inguinale mimicking as squamous cell carcinoma of penis. Indian J Sex Transm Dis AIDS 2014;35:56–8. 10.4103/0253-7184.132433 24958990 PMC4066601

[534] Ramdial PK, Sing Y, Ramburan A, Infantile donovanosis presenting as external auditory canal polyps: a diagnostic trap. Am J Dermatopathol 2012;34:818–21. 10.1097/DAD.0b013e3182540ccb 23169417

[535] Wahal SP, Tuli D. Donovanosis: an incidental finding on Pap test. J Cytol 2013;30:217–8. 10.4103/0970-9371.117638 24130421 PMC3793366

[536] Bowden FJ; National Donovanosis Eradication Advisory Committee. Donovanosis in Australia: going, going. Sex Transm Infect 2005;81:365–6. 10.1136/sti.2004.013227 16199732 PMC1745036

[537] Bright A. National Notifiable Diseases Surveillance System surveillance report: sexually transmissible infections in Aboriginal and Torres Strait Islander people. Commun Dis Intell Q Rep 2015;39:E584–9. 26779731 10.33321/cdi.2015.39.52

[538] O’Farrell N. Donovanosis. Sex Transm Infect 2002;78:452–7. 10.1136/sti.78.6.452 12473810 PMC1758360

[539] Mabey D, Peeling RW. Lymphogranuloma venereum. Sex Transm Infect 2002;78:90–2. 10.1136/sti.78.2.90 12081191 PMC1744436

[540] White JA. Manifestations and management of lymphogranuloma venereum. Curr Opin Infect Dis 2009;22:57–66. 10.1097/QCO.0b013e328320a8ae 19532081

[541] de Vries HJ, Zingoni A, White JA, Ross JD, Kreuter A. 2013 European Guideline on the management of proctitis, proctocolitis and enteritis caused by sexually transmissible pathogens. Int J STD AIDS 2014;25:465–74. 10.1177/0956462413516100 24352129

[542] Ward H, Martin I, Macdonald N, Lymphogranuloma venereum in the United kingdom. Clin Infect Dis 2007;44:26–32. 10.1086/509922 17143811

[543] Martin-Iguacel R, Llibre JM, Nielsen H, Lymphogranuloma venereum proctocolitis: a silent endemic disease in men who have sex with men in industrialised countries. Eur J Clin Microbiol Infect Dis 2010;29:917–25. 10.1007/s10096-010-0959-2 20509036

[544] de Voux A, Kent JB, Macomber K, Notes from the field: cluster of lymphogranuloma venereum cases among men who have sex with men—Michigan, August 2015–April 2016. MMWR Morb Mortal Wkly Rep 2016;65:920–1. 10.15585/mmwr.mm6534a6 27583686

[545] Pallawela SN, Sullivan AK, Macdonald N, Clinical predictors of rectal lymphogranuloma venereum infection: results from a multicentre case-control study in the U.K. Sex Transm Infect 2014;90:269–74. 10.1136/sextrans-2013-051401 24687130 PMC4033117

[546] de Vrieze NH, de Vries HJ. Lymphogranuloma venereum among men who have sex with men. An epidemiological and clinical review. Expert Rev Anti Infect Ther 2014;12:697–704. 10.1586/14787210.2014.901169 24655220

[547] Koper NE, van der Sande MA, Gotz HM, Koedijk FD; Dutch STI Clinics. Lymphogranuloma venereum among men who have sex with men in the Netherlands: regional differences in testing rates lead to underestimation of the incidence, 2006–2012. Euro Surveill 2013;18:20561. 10.2807/1560-7917.ES2013.18.34.20561 23987831

[548] Haar K, Dudareva-Vizule S, Wisplinghoff H, Lymphogranuloma venereum in men screened for pharyngeal and rectal infection, Germany. Emerg Infect Dis 2013;19:488–92. 10.3201/eid1903.121028 23621949 PMC3647660

[549] Riera-Monroig J, Fuertes de Vega I. Lymphogranuloma venereum presenting as an ulcer on the tongue. Sex Transm Infect 2019;95:169–70. 10.1136/sextrans-2018-053787 30554142

[550] Andrada MT, Dhar JK, Wilde H. Oral lymphogranuloma venereum and cervical lymphadenopathy. Case report. Mil Med 1974;139:99–101. 10.1093/milmed/139.2.99 4204816

[551] Ilyas S, Richmond D, Burns G, Orolabial lymphogranuloma venereum, Michigan, USA. Emerg Infect Dis 2019;25:2112–4. 10.3201/eid2511.190819 31625852 PMC6810219

[552] Kersh EN, Pillay A, de Voux A, Chen C. Laboratory processes for confirmation of lymphogranuloma venereum infection during a 2015 investigation of a cluster of cases in the United States. Sex Transm Dis 2017;44:691–4. 10.1097/OLQ.0000000000000667 28876314 PMC6684094

[553] CDC. Recommendations for the laboratory-based detection of *Chlamydia trachomatis* and *Neisseria gonorrhoeae*—2014. MMWR Recomm Rep 2014;63(No. RR-2). 24622331 PMC4047970

[554] Pathela P, Jamison K, Kornblum J, Quinlan T, Halse TA, Schillinger JA. Lymphogranuloma venereum: an increasingly common anorectal infection among men who have sex with men attending New York City sexual health clinics. Sex Transm Dis 2019;46:e14–7. 10.1097/OLQ.0000000000000921 30278027

[555] Cohen S, Brosnan H, Kohn R, P494 Diagnosis and management of lymphogranuloma venereum (LGV) in a municipal STD clinic, San Francisco, 2016–18. Sex Transm Infect 2019;95(Suppl 1):A229.

[556] Leeyaphan C, Ong JJ, Chow EP, Systematic review and meta-analysis of doxycycline efficacy for rectal lymphogranuloma venereum in men who have sex with men. Emerg Infect Dis 2016;22:1778–84. 10.3201/eid2210.160986 27513890 PMC5038401

[557] Cabello Úbeda A, Fernández Roblas R, García Delgado R, Anorectal lymphogranuloma venereum in Madrid: a persistent emerging problem in men who have sex with men. Sex Transm Dis 2016;43:414–9. 10.1097/OLQ.0000000000000459 27322040

[558] Simons R, Candfield S, French P, White JA. Observed treatment responses to short-course doxycycline therapy for rectal lymphogranuloma venereum in men who have sex with men. Sex Transm Dis 2018;45:406–8. 10.1097/OLQ.0000000000000772 29465660

[559] Vall-Mayans M, Isaksson J, Caballero E, Sallés B, Herrmann B. Bubonic lymphogranuloma venereum with multidrug treatment failure. Int J STD AIDS 2014;25:306–8. 10.1177/0956462413501158 24216037

[560] Blanco JL, Fuertes I, Bosch J, Effective treatment of lymphogranuloma venereum (LGV) with 1g azithormycin administered weekly for 3 weeks in HIV-infected population. Presented at the Conference on Retroviruses and Opportunist Infections, Seattle, WA; February 23–26, 2015.

[561] Kong FY, Rupasinghe TW, Simpson JA, Pharmacokinetics of a single 1g dose of azithromycin in rectal tissue in men. PLoS One 2017;12:e0174372. 10.1371/journal.pone.0174372 28350806 PMC5370104

[562] Elgalib A, Alexander S, Tong CY, White JA. Seven days of doxycycline is an effective treatment for asymptomatic rectal *Chlamydia trachomatis* infection. Int J STD AIDS 2011;22:474–7. 10.1258/ijsa.2011.011134 21764781

[563] Wormser GP, Wormser RP, Strle F, Myers R, Cunha BA. How safe is doxycycline for young children or for pregnant or breastfeeding women? Diagn Microbiol Infect Dis 2019;93:238–42. 10.1016/j.diagmicrobio.2018.09.015 30442509

[564] Towns JM, Leslie DE, Denham I, Azzato F, Fairley CK, Chen M. Painful and multiple anogenital lesions are common in men with *Treponema pallidum* PCR-positive primary syphilis without herpes simplex virus coinfection: a cross-sectional clinic-based study. Sex Transm Infect 2016;92:110–5. 10.1136/sextrans-2015-052219 26378262

[565] Theel ES, Katz SS, Pillay A. Molecular and direct detection tests for *Treponema pallidum* subspecies *pallidum*: a review of the literature, 1964–2017. Clin Infect Dis 2020;71(Suppl 1):S4–12. 10.1093/cid/ciaa176 32578865 PMC7312206

[566] Tuddenham S, Katz SS, Ghanem KG. Syphilis laboratory guidelines: performance characteristics of nontreponemal antibody tests. Clin Infect Dis 2020;71(Suppl 1):S21–42. 10.1093/cid/ciaa306 32578862 PMC7312285

[567] Park IU, Tran A, Pereira L, Fakile Y. Sensitivity and specificity of treponemal-specific tests for the diagnosis of syphilis. Clin Infect Dis 2020;71(Suppl 1):S13–20. 10.1093/cid/ciaa349 32578866 PMC7312216

[568] Bristow CC, Klausner JD, Tran A. Clinical test performance of a rapid point-of-care syphilis treponemal antibody test: a systematic review and meta-analysis. Clin Infect Dis 2020;71(Suppl 1):S52–7. 10.1093/cid/ciaa350 32578863 PMC7312211

[569] Nandwani R, Evans DT. Are you sure it’s syphilis? A review of false positive serology. Int J STD AIDS 1995;6:241–8. 10.1177/095646249500600404 7548285

[570] Romanowski B, Sutherland R, Fick GH, Mooney D, Love EJ. Serologic response to treatment of infectious syphilis. Ann Intern Med 1991;114:1005–9. 10.7326/0003-4819-114-12-1005 2029095

[571] CDC. Syphilis testing algorithms using treponemal tests for initial screening—four laboratories, New York City, 2005–2006. MMWR Morb Mortal Wkly Rep 2008;57:872–5. 18701877

[572] CDC. Discordant results from reverse sequence syphilis screening—five laboratories, United States, 2006–2010. MMWR Morb Mortal Wkly Rep 2011;60:133–7. 21307823

[573] Ortiz-Lopez N, Diez M, Diaz O, Simon F, Diaz A. Epidemiological surveillance of congenital syphilis in Spain, 2000–2010. Pediatr Infect Dis J 2012;31:988–90. 10.1097/INF.0b013e31825d3152 22572752

[574] Ortiz DA, Shukla MR, Loeffelholz MJ. The traditional or reverse algorithm for diagnosis of syphilis: pros and cons. Clin Infect Dis 2020;71(Suppl 1):S43–51. 10.1093/cid/ciaa307 32578864 PMC7312234

[575] Berry GJ, Loeffelholz MJ. Use of treponemal screening assay strength of signal to avoid unnecessary confirmatory testing. Sex Transm Dis 2016;43:737–40. 10.1097/OLQ.0000000000000524 27835625

[576] Park IU, Chow JM, Bolan G, Stanley M, Shieh J, Schapiro JM. Screening for syphilis with the treponemal immunoassay: analysis of discordant serology results and implications for clinical management. J Infect Dis 2011;204:1297–304. 10.1093/infdis/jir524 21930610

[577] Loeffelholz MJ, Wen T, Patel JA. Analysis of bioplex syphilis IgG quantitative results in different patient populations. Clin Vaccine Immunol 2011;18:2005–6. 10.1128/CVI.05335-11 21880852 PMC3209042

[578] Fakile YF, Jost H, Hoover KW, Correlation of treponemal immunoassay signal strength values with reactivity of confirmatory treponemal testing. J Clin Microbiol 2017;56:e01165-17. 10.1128/JCM.01165-17 29046410 PMC5744207

[579] Wong EH, Klausner JD, Caguin-Grygiel G, Evaluation of an IgM/IgG sensitive enzyme immunoassay and the utility of index values for the screening of syphilis infection in a high-risk population. Sex Transm Dis 2011;38:528–32. 10.1097/OLQ.0b013e318205491a 21233789

[580] Dai S, Chi P, Lin Y, Improved reverse screening algorithm for *Treponema pallidum* antibody using signal-to-cutoff ratios from chemiluminescence microparticle immunoassay. Sex Transm Dis 2014;41:29–34. 10.1097/OLQ.0000000000000066 24326578

[581] Li Z, Feng Z, Liu P, Yan C. Screening for antibodies against *Treponema pallidum* with chemiluminescent microparticle immunoassay: analysis of discordant serology results and clinical characterization. Ann Clin Biochem 2016;53:588–92. 10.1177/0004563215623806 26680646

[582] Yen-Lieberman B, Daniel J, Means C, Waletzky J, Daly TM. Identification of false-positive syphilis antibody results using a semiquantitative algorithm. Clin Vaccine Immunol 2011;18:1038–40. 10.1128/CVI.05066-11 21508162 PMC3122602

[583] Yimtae K, Srirompotong S, Lertsukprasert K. Otosyphilis: a review of 85 cases. Otolaryngol Head Neck Surg 2007;136:67–71. 10.1016/j.otohns.2006.08.026 17210336

[584] Gleich LL, Linstrom CJ, Kimmelman CP. Otosyphilis: a diagnostic and therapeutic dilemma. Laryngoscope 1992;102:1255–9. 10.1288/00005537-199211000-00010 1307698

[585] Lukehart SA, Hook EW 3rd, Baker-Zander SA, Collier AC, Critchlow CW, Handsfield HH. Invasion of the central nervous system by *Treponema pallidum*: implications for diagnosis and treatment. Ann Intern Med 1988;109:855–62. 10.7326/0003-4819-109-11-855 3056164

[586] Harding AS, Ghanem KG. The performance of cerebrospinal fluid treponemal-specific antibody tests in neurosyphilis: a systematic review. Sex Transm Dis 2012;39:291–7. 10.1097/OLQ.0b013e31824c0e62 22421696

[587] Jaffe HW, Larsen SA, Peters M, Jove DF, Lopez B, Schroeter AL. Tests for treponemal antibody in CSF. Arch Intern Med 1978;138:252–5. 10.1001/archinte.1978.03630260050016 343742

[588] Marra CM, Maxwell CL, Smith SL, Cerebrospinal fluid abnormalities in patients with syphilis: association with clinical and laboratory features. J Infect Dis 2004;189:369–76. 10.1086/381227 14745693

[589] CDC. Inadvertent use of Bicillin C-R to treat syphilis infection—Los Angeles, California, 1999–2004. MMWR Morb Mortal Wkly Rep 2005;54:217–9. 15758893 PMC6820132

[590] Butler T. The Jarisch-Herxheimer reaction after antibiotic treatment of spirochetal infections: a review of recent cases and our understanding of pathogenesis. Am J Trop Med Hyg 2017;96:46–52. 10.4269/ajtmh.16-0434 28077740 PMC5239707

[591] Rolfs RT, Joesoef MR, Hendershot EF, ; The Syphilis and HIV Study Group. A randomized trial of enhanced therapy for early syphilis in patients with and without human immunodeficiency virus infection. N Engl J Med 1997;337:307–14. 10.1056/NEJM199707313370504 9235493

[592] Yang CJ, Lee NY, Chen TC, One dose versus three weekly doses of benzathine penicillin G for patients co-infected with HIV and early syphilis: a multicenter, prospective observational study. PLoS One 2014;9:e109667. 10.1371/journal.pone.0109667 25286091 PMC4186862

[593] Ganesan A, Mesner O, Okulicz JF, ; Infectious Disease Clinical Research Program HIV/STI Working Group. A single dose of benzathine penicillin G is as effective as multiple doses of benzathine penicillin G for the treatment of HIV-infected persons with early syphilis. Clin Infect Dis 2015;60:653–60. 10.1093/cid/ciu888 25389249 PMC6477935

[594] Ghanem KG, Erbelding EJ, Wiener ZS, Rompalo AM. Serological response to syphilis treatment in HIV-positive and HIV-negative patients attending sexually transmitted diseases clinics. Sex Transm Infect 2007;83:97–101. 10.1136/sti.2006.021402 16943224 PMC2598600

[595] Seña AC, Wolff M, Martin DH, Predictors of serological cure and serofast state after treatment in HIV-negative persons with early syphilis. Clin Infect Dis 2011;53:1092–9. 10.1093/cid/cir671 21998287 PMC3205200

[596] Zhang RL, Wang QQ, Zhang JP, Yang LJ. Molecular subtyping of *Treponema pallidum* and associated factors of serofast status in early syphilis patients: identified novel genotype and cytokine marker. PLoS One 2017;12:e0175477. 10.1371/journal.pone.0175477 28410389 PMC5391950

[597] Seña AC, Zhang XH, Li T, A systematic review of syphilis serological treatment outcomes in HIV-infected and HIV-uninfected persons: rethinking the significance of serological non-responsiveness and the serofast state after therapy. BMC Infect Dis 2015;15:479. 10.1186/s12879-015-1209-0 26511465 PMC4625448

[598] Tong ML, Lin LR, Liu GL, Factors associated with serological cure and the serofast state of HIV-negative patients with primary, secondary, latent, and tertiary syphilis. PLoS One 2013;8:e70102. 10.1371/journal.pone.0070102 23894598 PMC3720935

[599] Seña AC, Wolff M, Behets F, Response to therapy following retreatment of serofast early syphilis patients with benzathine penicillin. Clin Infect Dis 2013;56:420–2. 10.1093/cid/cis918 23118269 PMC3590030

[600] Ghanem KG, Erbelding EJ, Cheng WW, Rompalo AM. Doxycycline compared with benzathine penicillin for the treatment of early syphilis. Clin Infect Dis 2006;42:e45–9. 10.1086/500406 16477545

[601] Wong T, Singh AE, De P. Primary syphilis: serological treatment response to doxycycline/tetracycline versus benzathine penicillin. Am J Med 2008;121:903–8. 10.1016/j.amjmed.2008.04.042 18823862

[602] Hook EW 3rd, Martin DH, Stephens J, Smith BS, Smith K. A randomized, comparative pilot study of azithromycin versus benzathine penicillin G for treatment of early syphilis. Sex Transm Dis 2002;29:486–90. 10.1097/00007435-200208000-00010 12172535

[603] Cao Y, Su X, Wang Q, A multicenter study evaluating ceftriaxone and benzathine penicillin G as treatment agents for early syphilis in Jiangsu, China. Clin Infect Dis 2017;65:1683–8. 10.1093/cid/cix611 29020150

[604] Riedner G, Rusizoka M, Todd J, Single-dose azithromycin versus penicillin G benzathine for the treatment of early syphilis. N Engl J Med 2005;353:1236–44. 10.1056/NEJMoa044284 16177249

[605] Hook EW 3rd, Behets F, Van Damme K, A phase III equivalence trial of azithromycin versus benzathine penicillin for treatment of early syphilis. J Infect Dis 2010;201:1729–35. 10.1086/652239 20402591

[606] Lukehart SA, Godornes C, Molini BJ, Macrolide resistance in *Treponema pallidum* in the United States and Ireland. N Engl J Med 2004;351:154–8. 10.1056/NEJMoa040216 15247355

[607] Mitchell SJ, Engelman J, Kent CK, Lukehart SA, Godornes C, Klausner JD. Azithromycin-resistant syphilis infection: San Francisco, California, 2000–2004. Clin Infect Dis 2006;42:337–45. 10.1086/498899 16392078

[608] A2058G Prevalence Workgroup. Prevalence of the 23S rRNA A2058G point mutation and molecular subtypes in *Treponema pallidum* in the United States, 2007 to 2009. Sex Transm Dis 2012;39:794–8. 23001267 10.1097/OLQ.0b013e31826f36de

[609] Rolfs RT, Joesoef MR, Hendershot EF, ; The Syphilis and HIV Study Group. A randomized trial of enhanced therapy for early syphilis in patients with and without human immunodeficiency virus infection. N Engl J Med 1997;337:307–14. 10.1056/NEJM199707313370504 9235493

[610] Collart P, Poitevin M, Milovanovic A, Herlin A, Durel J. Kinetic study of serum penicillin concentrations after single doses of benzathine and benethamine penicillins in young and old people. Br J Vener Dis 1980;56:355–62. 10.1136/sti.56.6.355 7448577 PMC1045831

[611] Hagdrup HK, Lange Wantzin G, Secher L, Rosdahl VT. Penicillin concentrations in serum following weekly injections of benzathine penicillin G. Chemotherapy 1986;32:99–101. 10.1159/000238397 3698728

[612] Frentz G, Nielsen PB, Espersen F, Czartoryski A, Aastrup H. Penicillin concentrations in blood and spinal fluid after a single intramuscular injection of penicillin G benzathine. Eur J Clin Microbiol 1984;3:147–9. 10.1007/BF02014334 6723638

[613] Nathan L, Bawdon RE, Sidawi JE, Stettler RW, McIntire DM, Wendel GD Jr. Penicillin levels following the administration of benzathine penicillin G in pregnancy. Obstet Gynecol 1993;82:338–42. 8355931

[614] Marra CM, Maxwell CL, Tantalo LC, Sahi SK, Lukehart SA. Normalization of serum rapid plasma reagin titer predicts normalization of cerebrospinal fluid and clinical abnormalities after treatment of neurosyphilis. Clin Infect Dis 2008;47:893–9. 10.1086/591534 18715154 PMC2729357

[615] Xiao Y, Tong ML, Lin LR, Serological response predicts normalization of cerebrospinal fluid abnormalities at six months after treatment in HIV-negative neurosyphilis patients. Sci Rep 2017;7:9911. 10.1038/s41598-017-10387-x 28855625 PMC5577126

[616] Hook EW 3rd, Baker-Zander SA, Moskovitz BL, Lukehart SA, Handsfield HH. Ceftriaxone therapy for asymptomatic neurosyphilis. Case report and Western blot analysis of serum and cerebrospinal fluid IgG response to therapy. Sex Transm Dis 1986;13(Suppl):185–8. 10.1097/00007435-198607000-00018 3764632

[617] Shann S, Wilson J. Treatment of neurosyphilis with ceftriaxone. Sex Transm Infect 2003;79:415–6. 10.1136/sti.79.5.415 14573840 PMC1744761

[618] Ahmed KA, Fox SJ, Frigas E, Park MA. Clinical outcome in the use of cephalosporins in pediatric patients with a history of penicillin allergy. Int Arch Allergy Immunol 2012;158:405–10. 10.1159/000333553 22487723

[619] Park MA, Koch CA, Klemawesch P, Joshi A, Li JT. Increased adverse drug reactions to cephalosporins in penicillin allergy patients with positive penicillin skin test. Int Arch Allergy Immunol 2010;153:268–73. 10.1159/000314367 20484925

[620] Novalbos A, Sastre J, Cuesta J, Lack of allergic cross-reactivity to cephalosporins among patients allergic to penicillins. Clin Exp Allergy 2001;31:438–43. 10.1046/j.1365-2222.2001.00992.x 11260156

[621] Pichichero ME, Casey JR. Safe use of selected cephalosporins in penicillin-allergic patients: a meta-analysis. Otolaryngol Head Neck Surg 2007;136:340–7. 10.1016/j.otohns.2006.10.007 17321857

[622] Kingston AA, Vujevich J, Shapiro M, Seronegative secondary syphilis in 2 patients coinfected with human immunodeficiency virus. Arch Dermatol 2005;141:431–3. 10.1001/archderm.141.4.431 15837859

[623] CDC. Symptomatic early neurosyphilis among HIV-positive men who have sex with men—four cities, United States, January 2002–June 2004. MMWR Morb Mortal Wkly Rep 2007;56:625–8. 17597693 PMC6818090

[624] Ghanem KG, Moore RD, Rompalo AM, Erbelding EJ, Zenilman JM, Gebo KA. Neurosyphilis in a clinical cohort of HIV-1-infected patients. AIDS 2008;22:1145–51. 10.1097/QAD.0b013e32830184df 18525260 PMC2553365

[625] Ghanem KG, Moore RD, Rompalo AM, Erbelding EJ, Zenilman JM, Gebo KA. Antiretroviral therapy is associated with reduced serologic failure rates for syphilis among HIV-infected patients. Clin Infect Dis 2008;47:258–65. 10.1086/589295 18532887 PMC2562285

[626] Tomkins A, Ahmad S, Cousins DE, Thng CM, Vilar FJ, Higgins SP. Screening for asymptomatic neurosyphilis in HIV patients after treatment of early syphilis: an observational study. Sex Transm Infect 2018;94:337–9. 10.1136/sextrans-2016-052938 28196838

[627] Yang CJ, Chang SY, Hung CC. Sensitivity and specificity of lumbar puncture in HIV-infected patients with syphilis and no neurologic symptoms. Clin Infect Dis 2009;49:162–3, author reply 162–3. 10.1086/599616 19500029

[628] Marra CM, Boutin P, McArthur JC, A pilot study evaluating ceftriaxone and penicillin G as treatment agents for neurosyphilis in human immunodeficiency virus-infected individuals. Clin Infect Dis 2000;30:540–4. 10.1086/313725 10722441

[629] Dowell ME, Ross PG, Musher DM, Cate TR, Baughn RE. Response of latent syphilis or neurosyphilis to ceftriaxone therapy in persons infected with human immunodeficiency virus. Am J Med 1992;93:481–8. 10.1016/0002-9343(92)90574-U 1442850

[630] Smith NH, Musher DM, Huang DB, Response of HIV-infected patients with asymptomatic syphilis to intensive intramuscular therapy with ceftriaxone or procaine penicillin. Int J STD AIDS 2004;15:328–32. 10.1177/095646240401500511 15117503

[631] Ahmed KA, Fox SJ, Frigas E, Park MA. Clinical outcome in the use of cephalosporins in pediatric patients with a history of penicillin allergy. Int Arch Allergy Immunol 2012;158:405–10. 10.1159/000333553 22487723

[632] Trivedi S, Williams C, Torrone E, Kidd S. National trends and reported risk factors among pregnant women with syphilis in the United States, 2012–2016. Obstet Gynecol 2019;133:27–32. 10.1097/AOG.0000000000003000 30531570 PMC6743074

[633] Biswas HH, Chew Ng RA, Murray EL, Characteristics associated with delivery of an infant with congenital syphilis and missed opportunities for prevention—California, 2012 to 2014. Sex Transm Dis 2018;45:435–41. 10.1097/OLQ.0000000000000782 29465666

[634] Slutsker JS, Hennessy RR, Schillinger JA. Factors contributing to congenital syphilis cases—New York City, 2010–2016. MMWR Morb Mortal Wkly Rep 2018;67:1088–93. 10.15585/mmwr.mm6739a3 30286056 PMC6171893

[635] DiOrio D, Kroeger K, Ross A. Social vulnerability in congenital syphilis case mothers: qualitative assessment of cases in Indiana, 2014 to 2016. Sex Transm Dis 2018;45:447–51. 10.1097/OLQ.0000000000000783 29465662 PMC5995623

[636] Kimball A, Torrone E, Miele K, Missed opportunities for prevention of congenital syphilis—United States, 2018. MMWR Morb Mortal Wkly Rep 2020;69:661–5. 10.15585/mmwr.mm6922a1 32497029 PMC7272112

[637] Park IU, Chow JM, Bolan G, Stanley M, Shieh J, Schapiro JM. Screening for syphilis with the treponemal immunoassay: analysis of discordant serology results and implications for clinical management. J Infect Dis 2011;204:1297–304. 10.1093/infdis/jir524 21930610

[638] Mmeje O, Chow JM, Davidson L, Shieh J, Schapiro JM, Park IU. Discordant syphilis immunoassays in pregnancy: perinatal outcomes and implications for clinical management. Clin Infect Dis 2015;61:1049–53. 10.1093/cid/civ445 26063719 PMC4560902

[639] Alexander JM, Sheffield JS, Sanchez PJ, Mayfield J, Wendel GD Jr. Efficacy of treatment for syphilis in pregnancy. Obstet Gynecol 1999;93:5–8. 9916946 10.1016/s0029-7844(98)00338-x

[640] Walker GJ. Antibiotics for syphilis diagnosed during pregnancy. Cochrane Database Syst Rev 2001;(3):CD001143. 11686978 10.1002/14651858.CD001143 PMC8407021

[641] Wendel GD Jr, Sheffield JS, Hollier LM, Hill JB, Ramsey PS, Sánchez PJ. Treatment of syphilis in pregnancy and prevention of congenital syphilis. Clin Infect Dis 2002;35(Suppl 2):S200–9. 10.1086/342108 12353207

[642] Zhu L, Qin M, Du L, Xie RH, Wong T, Wen SW. Maternal and congenital syphilis in Shanghai, China, 2002 to 2006. Int J Infect Dis 2010;14(Suppl 3):e45–8. 10.1016/j.ijid.2009.09.009 20137991

[643] Hawkes S, Matin N, Broutet N, Low N. Effectiveness of interventions to improve screening for syphilis in pregnancy: a systematic review and meta-analysis. Lancet Infect Dis 2011;11:684–91. 10.1016/S1473-3099(11)70104-9 21683653

[644] Hollier LM, Harstad TW, Sanchez PJ, Twickler DM, Wendel GD Jr. Fetal syphilis: clinical and laboratory characteristics. Obstet Gynecol 2001;97:947–53. 11384701 10.1016/s0029-7844(01)01367-9

[645] Rac MW, Bryant SN, McIntire DD, Progression of ultrasound findings of fetal syphilis after maternal treatment. Am J Obstet Gynecol 2014;211:426.e1–6. 10.1016/j.ajog.2014.05.049 24907700

[646] Zhou P, Gu Z, Xu J, Wang X, Liao K. A study evaluating ceftriaxone as a treatment agent for primary and secondary syphilis in pregnancy. Sex Transm Dis 2005;32:495–8. 10.1097/01.olq.0000170443.70739.cd 16041252

[647] Katanami Y, Hashimoto T, Takaya S, Amoxicillin and ceftriaxone as treatment alternatives to penicillin for maternal syphilis. Emerg Infect Dis 2017;23:827–9. 10.3201/eid2305.161936 28418316 PMC5403051

[648] Kestenbaum LA, Ebberson J, Zorc JJ, Hodinka RL, Shah SS. Defining cerebrospinal fluid white blood cell count reference values in neonates and young infants. Pediatrics 2010;125:257–64. 10.1542/peds.2009-1181 20064869 PMC3033868

[649] Shah SS, Ebberson J, Kestenbaum LA, Hodinka RL, Zorc JJ. Age-specific reference values for cerebrospinal fluid protein concentration in neonates and young infants. J Hosp Med 2011;6:22–7. 10.1002/jhm.711 20629018 PMC2978786

[650] Thomson J, Sucharew H, Cruz AT, ; Pediatric Emergency Medicine Collaborative Research Committee (PEM CRC) HSV Study Group. Cerebrospinal fluid reference values for young infants undergoing lumbar puncture. Pediatrics 2018;141:e20173405. 10.1542/peds.2017-3405 29437883

[651] Kimberlin DW, Brady MT, Jackson MA, Long SS, eds. Red book: 2018 report of the Committee on Infectious Diseases. 31st ed. Itasca, IL: American Academy of Pediatrics; 2018.

[652] Macy E, Contreras R. Adverse reactions associated with oral and parenteral use of cephalosporins: a retrospective population-based analysis. J Allergy Clin Immunol 2015;135:745–52.e5. 10.1016/j.jaci.2014.07.062 25262461

[653] Macy E, Vyles D. Who needs penicillin allergy testing? Ann Allergy Asthma Immunol 2018;121:523–9. 10.1016/j.anai.2018.07.041 30092265

[654] Annè S, Reisman RE. Risk of administering cephalosporin antibiotics to patients with histories of penicillin allergy. Ann Allergy Asthma Immunol 1995;74:167–70. 7697478

[655] Albin S, Agarwal S. Prevalence and characteristics of reported penicillin allergy in an urban outpatient adult population. Allergy Asthma Proc 2014;35:489–94. 10.2500/aap.2014.35.3791 25584917 PMC4210656

[656] Blumenthal KG, Peter JG, Trubiano JA, Phillips EJ. Antibiotic allergy. Lancet 2019;393:183–98. 10.1016/S0140-6736(18)32218-9 30558872 PMC6563335

[657] Macy E, Poon K-Y T. Self-reported antibiotic allergy incidence and prevalence: age and sex effects. Am J Med 2009;122:778.e1–7. 10.1016/j.amjmed.2009.01.034 19635279

[658] Shenoy ES, Macy E, Rowe T, Blumenthal KG. Evaluation and management of penicillin allergy: a review. JAMA 2019;321:188–99. 10.1001/jama.2018.19283 30644987

[659] Gadde J, Spence M, Wheeler B, Adkinson NF Jr. Clinical experience with penicillin skin testing in a large inner-city STD clinic. JAMA 1993;270:2456–63. 10.1001/jama.1993.03510200062033 8230623

[660] Macy E, Ngor EW. Safely diagnosing clinically significant penicillin allergy using only penicilloyl-poly-lysine, penicillin, and oral amoxicillin. J Allergy Clin Immunol Pract 2013;1:258–63. 10.1016/j.jaip.2013.02.002 24565482

[661] Jares EJ, Sánchez-Borges M, Cardona-Villa R, ; Latin America Drug Allergy Interest Group. Multinational experience with hypersensitivity drug reactions in Latin America. Ann Allergy Asthma Immunol 2014;113:282–9. 10.1016/j.anai.2014.06.019 25065979

[662] Macy E, Contreras R. Health care use and serious infection prevalence associated with penicillin “allergy” in hospitalized patients: A cohort study. J Allergy Clin Immunol 2014;133:790–6. 10.1016/j.jaci.2013.09.021 24188976

[663] Blumenthal KG, Lu N, Zhang Y, Li Y, Walensky RP, Choi HK. Risk of meticillin resistant *Staphylococcus aureus* and *Clostridium difficile* in patients with a documented penicillin allergy: population based matched cohort study. BMJ 2018;361:k2400. 10.1136/bmj.k2400 29950489 PMC6019853

[664] Blumenthal KG, Ryan EE, Li Y, Lee H, Kuhlen JL, Shenoy ES. The impact of a reported penicillin allergy on surgical site infection risk. Clin Infect Dis 2018;66:329–36. 10.1093/cid/cix794 29361015 PMC5850334

[665] Tucker MH, Lomas CM, Ramchandar N, Waldram JD. Amoxicillin challenge without penicillin skin testing in evaluation of penicillin allergy in a cohort of Marine recruits. J Allergy Clin Immunol Pract 2017;5:813–5. 10.1016/j.jaip.2017.01.023 28341170

[666] Goldberg A, Confino-Cohen R. Skin testing and oral penicillin challenge in patients with a history of remote penicillin allergy. Ann Allergy Asthma Immunol 2008;100:37–43. 10.1016/S1081-1206(10)60402-4 18254480

[667] Iammatteo M, Alvarez Arango S, Ferastraoaru D, Safety and outcomes of oral graded challenges to amoxicillin without prior skin testing. J Allergy Clin Immunol Pract 2019;7:236–43. 10.1016/j.jaip.2018.05.008 29802906

[668] Cook DJ, Barbara DW, Singh KE, Dearani JA. Penicillin skin testing in cardiac surgery. J Thorac Cardiovasc Surg 2014;147:1931–5. 10.1016/j.jtcvs.2014.01.019 24530197

[669] McDanel DL, Azar AE, Dowden AM, Screening for beta-lactam allergy in joint arthroplasty patients to improve surgical prophylaxis practice. J Arthroplasty 2017;32(9s):S101–8. 10.1016/j.arth.2017.01.012 28236547

[670] Trubiano JA, Thursky KA, Stewardson AJ, Impact of an integrated antibiotic allergy testing program on antimicrobial stewardship: a multicenter evaluation. Clin Infect Dis 2017;65:166–74. 10.1093/cid/cix244 28520865 PMC5849110

[671] Siew LQC, Li PH, Watts TJ, Identifying low-risk beta-lactam allergy patients in a UK tertiary centre. J Allergy Clin Immunol Pract 2019;7:2173–2181.e1. 10.1016/j.jaip.2019.03.015 30922992

[672] Chen JR, Tarver SA, Alvarez KS, Tran T, Khan DA. A proactive approach to penicillin allergy testing in hospitalized patients. J Allergy Clin Immunol Pract 2017;5:686–93. 10.1016/j.jaip.2016.09.045 27888034

[673] Leis JA, Palmay L, Ho G, Point-of-care β-lactam allergy skin testing by antimicrobial stewardship programs: a pragmatic multicenter prospective evaluation. Clin Infect Dis 2017;65:1059–65. 10.1093/cid/cix512 28575226

[674] Banks TA, Tucker M, Macy E. Evaluating penicillin allergies without skin testing. Curr Allergy Asthma Rep 2019;19:27. 10.1007/s11882-019-0854-6 30903298

[675] Pham MN, Ho HE, Desai M. Penicillin desensitization: treatment of syphilis in pregnancy in penicillin-allergic patients. Ann Allergy Asthma Immunol 2017;118:537–41. 10.1016/j.anai.2017.03.013 28477786

[676] Sogn DD, Evans R 3rd, Shepherd GM, Results of the National Institute of Allergy and Infectious Diseases Collaborative Clinical Trial to test the predictive value of skin testing with major and minor penicillin derivatives in hospitalized adults. Arch Intern Med 1992;152:1025–32. 10.1001/archinte.1992.00400170105020 1580706

[677] Solensky R, Jacobs J, Lester M, Penicillin allergy evaluation: a prospective, multicenter, open-label evaluation of a comprehensive penicillin skin test kit. J Allergy Clin Immunol Pract 2019;7:1876–85.e3. 10.1016/j.jaip.2019.02.040 30878711

[678] Heil EL, Bork JT, Schmalzle SA, Implementation of an infectious disease fellow-managed penicillin allergy skin testing service. Open Forum Infect Dis 2016;3:ofw155. 10.1093/ofid/ofw155 27704011 PMC5047432

[679] du Plessis T, Walls G, Jordan A, Holland DJ. Implementation of a pharmacist-led penicillin allergy de-labelling service in a public hospital. J Antimicrob Chemother 2019;74:1438–46. 10.1093/jac/dky575 30753497

[680] Macy E, Blumenthal KG. Are cephalosporins safe for use in penicillin allergy without prior allergy evaluation? J Allergy Clin Immunol Pract 2018;6:82–9. 10.1016/j.jaip.2017.07.033 28958745

[681] Zagursky RJ, Pichichero ME. Cross-reactivity in β-lactam allergy. J Allergy Clin Immunol Pract 2018;6:72–81.e1. 10.1016/j.jaip.2017.08.027 29017833

[682] Blumenthal KG, Shenoy ES, Varughese CA, Hurwitz S, Hooper DC, Banerji A. Impact of a clinical guideline for prescribing antibiotics to inpatients reporting penicillin or cephalosporin allergy. Ann Allergy Asthma Immunol 2015;115:294–300.e2. 10.1016/j.anai.2015.05.011 26070805 PMC4593731

[683] Kuruvilla M, Wolf F, Sexton M, Wiley Z, Thomas J. Perioperative use of cefazolin without preliminary skin testing in patients with reported penicillin allergy. Surgery 2019;165:486–96. 10.1016/j.surg.2018.05.054 30001827

[684] Lee P, Shanson D. Results of a UK survey of fatal anaphylaxis after oral amoxicillin. J Antimicrob Chemother 2007;60:1172–3. 10.1093/jac/dkm315 17761735

[685] Blumenthal KG, Shenoy ES, Wolfson AR, Addressing inpatient beta-lactam allergies: a multihospital implementation. J Allergy Clin Immunol Pract 2017;5:616–25.e7. 10.1016/j.jaip.2017.02.019 28483315 PMC5484001

[686] Mustafa SS, Conn K, Ramsey A. Comparing direct challenge to penicillin skin testing for the outpatient evaluation of penicillin allergy: a randomized controlled trial. J Allergy Clin Immunol Pract 2019;7:2163–70. 10.1016/j.jaip.2019.05.037 31170542

[687] Chastain DB, Hutzley VJ, Parekh J, Alegro JVG. Antimicrobial desensitization: a review of published protocols. Pharmacy (Basel) 2019;7:112. 10.3390/pharmacy7030112 31405062 PMC6789802

[688] Wendel GD Jr, Stark BJ, Jamison RB, Molina RD, Sullivan TJ. Penicillin allergy and desensitization in serious infections during pregnancy. N Engl J Med 1985;312:1229–32. 10.1056/NEJM198505093121905 3921835

[689] Borish L, Tamir R, Rosenwasser LJ. Intravenous desensitization to beta-lactam antibiotics. J Allergy Clin Immunol 1987;80:314–9. 10.1016/0091-6749(87)90037-6 3040836

[690] Legere HJ 3rd, Palis RI, Rodriguez Bouza T, Uluer AZ, Castells MC. A safe protocol for rapid desensitization in patients with cystic fibrosis and antibiotic hypersensitivity. J Cyst Fibros 2009;8:418–24. 10.1016/j.jcf.2009.08.002 19740711 PMC2787787

[691] Manhart LE, Holmes KK, Hughes JP, Houston LS, Totten PA. *Mycoplasma genitalium* among young adults in the United States: an emerging sexually transmitted infection. Am J Public Health 2007;97:1118–25. 10.2105/AJPH.2005.074062 17463380 PMC1874220

[692] Ross JDC, Jensen JS. *Mycoplasma genitalium* as a sexually transmitted infection: implications for screening, testing, and treatment. Sex Transm Infect 2006;82:269–71. 10.1136/sti.2005.017368 16877571 PMC2564705

[693] Taylor-Robinson D, Gilroy CB, Thomas BJ, Hay PE. *Mycoplasma genitalium* in chronic non-gonococcal urethritis. Int J STD AIDS 2004;15:21–5. 10.1258/095646204322637209 14769166

[694] Dupin N, Bijaoui G, Schwarzinger M, Detection and quantification of *Mycoplasma genitalium* in male patients with urethritis. Clin Infect Dis 2003;37:602–5. 10.1086/376990 12905147

[695] Krieger JN, Riley DE, Roberts MC, Berger RE. Prokaryotic DNA sequences in patients with chronic idiopathic prostatitis. J Clin Microbiol 1996;34:3120–8. 10.1128/JCM.34.12.3120-3128.1996 8940458 PMC229469

[696] le Roux MC, Hoosen AA. Quantitative real-time polymerase chain reaction for the diagnosis of *Mycoplasma genitalium* infection in South African men with and without symptoms of urethritis. Sex Transm Dis 2017;44:17–20. 10.1097/OLQ.0000000000000540 27898565

[697] Bachmann LH, Kirkcaldy RD, Geisler WM, ; the MAGNUM Laboratory Working Group. Prevalence of *Mycoplasma genitalium* infection, antimicrobial resistance mutations and symptom resolution following treatment of urethritis. Clin Infect Dis 2020;71:e624–32. 10.1093/cid/ciaa293 32185385 PMC7744987

[698] Nye MB, Schwebke JR, Body BA. Comparison of APTIMA *Trichomonas vaginalis* transcription-mediated amplification to wet mount microscopy, culture, and polymerase chain reaction for diagnosis of trichomoniasis in men and women. Am J Obstet Gynecol 2009;200:188.e1–7. 10.1016/j.ajog.2008.10.005 19185101

[699] Bradshaw CS, Tabrizi SN, Read TR, Etiologies of nongonococcal urethritis: bacteria, viruses, and the association with orogenital exposure. J Infect Dis 2006;193:336–45. 10.1086/499434 16388480

[700] Dombrowski JC, Harrington RD, Golden MR. Evidence for the long-term stability of HIV transmission-associated sexual behavior after HIV diagnosis. Sex Transm Dis 2013;40:41–5. 10.1097/OLQ.0b013e3182753327 23254116 PMC4150917

[701] Rane VS, Fairley CK, Weerakoon A, Characteristics of acute nongonococcal urethritis in men differ by sexual preference. J Clin Microbiol 2014;52:2971–6. 10.1128/JCM.00899-14 24899041 PMC4136159

[702] Pond MJ, Nori AV, Witney AA, Lopeman RC, Butcher PD, Sadiq ST. High prevalence of antibiotic-resistant *Mycoplasma genitalium* in nongonococcal urethritis: the need for routine testing and the inadequacy of current treatment options. Clin Infect Dis 2014;58:631–7. 10.1093/cid/cit752 24280088 PMC3922211

[703] Khatib N, Bradbury C, Chalker V, Prevalence of *Trichomonas vaginalis, Mycoplasma genitalium* and *Ureaplasma urealyticum* in men with urethritis attending an urban sexual health clinic. Int J STD AIDS 2015;26:388–92. 10.1177/0956462414539464 24925897

[704] Cox C, McKenna JP, Watt AP, Coyle PV. *Ureaplasma parvum* and *Mycoplasma genitalium* are found to be significantly associated with microscopy-confirmed urethritis in a routine genitourinary medicine setting. Int J STD AIDS 2016;27:861–7. 10.1177/0956462415597620 26378187

[705] Li Y, Su X, Le W, *Mycoplasma genitalium* in symptomatic male urethritis: macrolide use is associated with increased resistance. Clin Infect Dis 2020;70:805–10. 10.1093/cid/ciz294 30972419 PMC7390511

[706] Ito S, Hanaoka N, Shimuta K, Male non-gonococcal urethritis: from microbiological etiologies to demographic and clinical features. Int J Urol 2016;23:325–31. 10.1111/iju.13044 26845624

[707] Horner P, Donders G, Cusini M, Gomberg M, Jensen JS, Unemo M. Should we be testing for urogenital *Mycoplasma hominis, Ureaplasma parvum* and *Ureaplasma urealyticum* in men and women?—A position statement from the European STI Guidelines Editorial Board. J Eur Acad Dermatol Venereol 2018;32:1845–51. 10.1111/jdv.15146 29924422

[708] van der Veer C, van Rooijen MS, Himschoot M, de Vries HJ, Bruisten SM. *Trichomonas vaginalis* and *Mycoplasma genitalium*: age-specific prevalence and disease burden in men attending a sexually transmitted infections clinic in Amsterdam, the Netherlands. Sex Transm Infect 2016;92:83–5. 10.1136/sextrans-2015-052118 26283740

[709] Seike K, Maeda S, Kubota Y, Tamaki M, Yasuda M, Deguchi T. Prevalence and morbidity of urethral *Trichomonas vaginalis* in Japanese men with or without urethritis. Sex Transm Infect 2013;89:528–30. 10.1136/sextrans-2012-050702 23349337

[710] Napierala M, Munson E, Wenten D, Detection of *Mycoplasma genitalium* from male primary urine specimens: an epidemiologic dichotomy with *Trichomonas vaginalis.* Diagn Microbiol Infect Dis 2015;82:194–8. 10.1016/j.diagmicrobio.2015.03.016 25934156

[711] Sviben M, Missoni EM, Meštrović T, Vojnović G, Galinović GM. Epidemiology and laboratory characteristics of *Trichomonas vaginalis* infection in Croatian men with and without urethritis syndrome: a case-control study. Sex Transm Infect 2015;91:360–4. 10.1136/sextrans-2014-051771 25568091

[712] Rietmeijer CA, Mungati M, Machiha A, The etiology of male urethral discharge in Zimbabwe: results from the Zimbabwe STI Etiology Study. Sex Transm Dis 2018;45:56–60. 10.1097/OLQ.0000000000000696 29240635 PMC10719555

[713] Bazan JA, Peterson AS, Kirkcaldy RD, Notes from the field: increase in *Neisseria meningitidis*-associated urethritis among men at two sentinel clinics—Columbus, Ohio, and Oakland County, Michigan, 2015. MMWR Morb Mortal Wkly Rep 2016;65:550–2. 10.15585/mmwr.mm6521a5 27254649 PMC5390329

[714] Jannic A, Mammeri H, Larcher L, Orogenital transmission of *Neisseria meningitidis* causing acute urethritis in men who have sex with men. Emerg Infect Dis 2019;25:175–6. 10.3201/eid2501.171102 30561300 PMC6302579

[715] Hayakawa K, Itoda I, Shimuta K, Takahashi H, Ohnishi M. Urethritis caused by novel *Neisseria meningitidis* serogroup W in man who has sex with men, Japan. Emerg Infect Dis 2014;20:1585–7. 10.3201/eid2009.140349 25154021 PMC4178410

[716] Bazan JA, Tzeng YL, Stephens DS, Repeat episodes of symptomatic urethritis due to a uropathogenic meningococcal clade. Sex Transm Dis 2020;47:e1–4. 10.1097/OLQ.0000000000001079 31651709 PMC6923539

[717] Ong JJ, Morton AN, Henzell HR, Clinical characteristics of herpes simplex virus urethritis compared with chlamydial urethritis among men. Sex Transm Dis 2017;44:121–5. 10.1097/OLQ.0000000000000547 28079748

[718] Avolio M, De Rosa R, Modolo ML, Stano P, Camporese A. When should adenoviral non-gonococcal urethritis be suspected? Two case reports. New Microbiol 2014;37:109–12. 24531179

[719] You C, Hamasuna R, Ogawa M, The first report: an analysis of bacterial flora of the first voided urine specimens of patients with male urethritis using the 16S ribosomal RNA gene-based clone library method. Microb Pathog 2016;95:95–100. 10.1016/j.micpath.2016.02.022 27013259

[720] Deguchi T, Ito S, Hatazaki K, Antimicrobial susceptibility of *Haemophilus influenzae* strains isolated from the urethra of men with acute urethritis and/or epididymitis. J Infect Chemother 2017;23:804–7. 10.1016/j.jiac.2017.05.009 28619239

[721] Ito S, Hatazaki K, Shimuta K, *Haemophilus influenzae* isolated from men with acute urethritis: its pathogenic roles, responses to antimicrobial chemotherapies, and antimicrobial susceptibilities. Sex Transm Dis 2017;44:205–10. 10.1097/OLQ.0000000000000573 28282645

[722] Horie K, Ito S, Hatazaki K, ‘*Haemophilus quentini*’ in the urethra of men complaining of urethritis symptoms. J Infect Chemother 2018;24:71–4. 10.1016/j.jiac.2017.08.007 28889986

[723] Frølund M, Falk L, Ahrens P, Jensen JS. Detection of ureaplasmas and bacterial vaginosis associated bacteria and their association with non-gonococcal urethritis in men. PLoS One 2019;14:e0214425. 10.1371/journal.pone.0214425 30946763 PMC6448876

[724] Deza G, Martin-Ezquerra G, Gómez J, Villar-García J, Supervia A, Pujol RM. Isolation of *Haemophilus influenzae* and *Haemophilus parainfluenzae* in urethral exudates from men with acute urethritis: a descriptive study of 52 cases. Sex Transm Infect 2016;92:29–31. 10.1136/sextrans-2015-052135 26139207

[725] Magdaleno-Tapial J, Valenzuela-Oñate C, Giacaman-von der Weth MM, *Haemophilus* species isolated in urethral exudates as a possible causative agent in acute urethritis: a study of 38 cases. Actas Dermosifiliogr 2019;110:38–42. 10.1016/j.adengl.2018.11.011 30390917

[726] Abdolrasouli A, Roushan A. *Corynebacterium propinquum* associated with acute, nongonococcal urethritis. Sex Transm Dis 2013;40:829–31. 10.1097/OLQ.0000000000000027 24275738

[727] Ongrádi J, Stercz B, Kövesdi V, Nagy K, Chatlynne L. Isolation of *Kurthia gibsonii* from non-gonorrheal urethritis: implications for the pathomechanism upon surveying the literature. Acta Microbiol Immunol Hung 2014;61:79–87. 10.1556/AMicr.61.2014.1.8 24631755

[728] Gherardi G, Di Bonaventura G, Pompilio A, Savini V. *Corynebacterium glucuronolyticum* causing genitourinary tract infection: case report and review of the literature. IDCases 2015;2:56–8. 10.1016/j.idcr.2015.03.001 26793456 PMC4672622

[729] Meštrović T. A microbial game of whack-a-mole: clinical case series of the urethral uncloaking phenomenon caused by *Corynebacterium glucuronolyticum* in men treated for *Chlamydia trachomatis* urethritis. Infection 2019;47:121–4. 10.1007/s15010-018-1211-8 30168068

[730] Frikh M, El Yaagoubi I, Lemnouer A, Elouennass M. Urethritis due to *corynebacterium striatum*: an emerging germ. Tunis Med 2015;93:43–4. 25955369

[731] Babaeer AA, Nader C, Iacoviello V, Tomera K. Necrotizing urethritis due to *Aerococcus urinae.* Case Rep Urol 2015;2015:136147. 10.1155/2015/136147 26171271 PMC4480802

[732] Grandolfo M, Vestita M, Bonamonte D, Filoni A. Acute urethritis and balonoposthitis associated to *Neisseria elongata.* Sex Transm Dis 2016;43:778–9. 10.1097/OLQ.0000000000000532 27832027

[733] Frølund M, Lidbrink P, Wikström A, Cowan S, Ahrens P, Jensen JS. Urethritis-associated pathogens in urine from men with non-gonococcal urethritis: a case-control study. Acta Derm Venereol 2016;96:689–94. 10.2340/00015555-2314 26658669

[734] Chambers LC, Morgan JL, Lowens MS, Cross-sectional study of urethral exposures at last sexual episode associated with non-gonococcal urethritis among STD clinic patients. Sex Transm Infect 2019;95:212–8. 10.1136/sextrans-2018-053634 30181326 PMC7016488

[735] Manhart LE, Khosropour CM, Liu C, Bacterial vaginosis-associated bacteria in men: association of *Leptotrichia/Sneathia* spp. with nongonococcal urethritis. Sex Transm Dis 2013;40:944–9. 10.1097/OLQ.0000000000000054 24220356 PMC4188452

[736] Ashraf J, Radford AR, Turner A, Subramaniam R. Preliminary experience with instillation of triamcinolone acetonide into the urethra for idiopathic urethritis: a prospective pilot study. J Laparoendosc Adv Surg Tech A 2017;27:1217–21. 10.1089/lap.2017.0064 29023188

[737] Taylor SN, DiCarlo RP, Martin DH. Comparison of methylene blue/gentian violet stain to Gram’s stain for the rapid diagnosis of gonococcal urethritis in men. Sex Transm Dis 2011;38:995–6. 10.1097/OLQ.0b013e318225f7c2 21992973

[738] Rietmeijer CA, Mettenbrink CJ. Recalibrating the Gram stain diagnosis of male urethritis in the era of nucleic acid amplification testing. Sex Transm Dis 2012;39:18–20. 10.1097/OLQ.0b013e3182354da3 22183839

[739] Geisler WM, Yu S, Hook EW 3rd. Chlamydial and gonococcal infection in men without polymorphonuclear leukocytes on Gram stain: implications for diagnostic approach and management. Sex Transm Dis 2005;32:630–4. 10.1097/01.olq.0000175390.45315.a1 16205305

[740] Gottesman T, Yossepowitch O, Samra Z, Rosenberg S, Dan M. Prevalence of *Mycoplasma genitalium* in men with urethritis and in high risk asymptomatic males in Tel Aviv: a prospective study. Int J STD AIDS 2017;28:127–32. 10.1177/0956462416630675 26826161

[741] Kim HJ, Park JK, Park SC, The prevalence of causative organisms of community-acquired urethritis in an age group at high risk for sexually transmitted infections in Korean soldiers. J R Army Med Corps 2017;163:20–2. 10.1136/jramc-2015-000488 26607860

[742] Libois A, Hallin M, Crucitti T, Delforge M, De Wit S. Prevalence of *Mycoplasma genitalium* in men with urethritis in a large public hospital in Brussels, Belgium: an observational, cross-sectional study. PLoS One 2018;13:e0196217. 10.1371/journal.pone.0196217 29698421 PMC5919460

[743] Bachmann LH, Manhart LE, Martin DH, Advances in the understanding and treatment of male urethritis. Clin Infect Dis 2015;61(Suppl 8):S763–9. 10.1093/cid/civ755 26602615

[744] Samaraweera GR, Garcia K, Druce J, Characteristics of adenovirus urethritis among heterosexual men and men who have sex with men: a review of clinical cases. Sex Transm Infect 2016;92:172–4. 10.1136/sextrans-2015-052243 26574571

[745] Horner P, Blee K, O’Mahony C, Muir P, Evans C, Radcliffe K; Clinical Effectiveness Group of the British Association for Sexual Health and HIV. 2015 UK National Guideline on the management of non-gonococcal urethritis. Int J STD AIDS 2016;27:85–96. 10.1177/0956462415586675 26002319

[746] Sarier M, Sepin N, Duman I, Microscopy of Gram-stained urethral smear in the diagnosis of urethritis: which threshold value should be selected? Andrologia 2018;50:e13143. 10.1111/and.13143 30238498

[747] Sarier M, Kukul E. Classification of non-gonococcal urethritis: a review. Int Urol Nephrol 2019;51:901–7. 10.1007/s11255-019-02140-2 30953260

[748] Kong FY, Tabrizi SN, Law M, Azithromycin versus doxycycline for the treatment of genital chlamydia infection: a meta-analysis of randomized controlled trials. Clin Infect Dis 2014;59:193–205. 10.1093/cid/ciu220 24729507

[749] Páez-Canro C, Alzate JP, González LM, Rubio-Romero JA, Lethaby A, Gaitán HG. Antibiotics for treating urogenital *Chlamydia trachomatis* infection in men and non-pregnant women. Cochrane Database Syst Rev 2019;1:CD010871. 10.1002/14651858.CD010871.pub2 30682211 PMC6353232

[750] McIver R, Jalocon D, McNulty A, Men who have sex with men with *Mycoplasma genitalium*-positive nongonococcal urethritis are more likely to have macrolide resistant strains than men with only female partners: a prospective study. Sex Transm Dis 2019;46:513–7. 10.1097/OLQ.0000000000001009 31295218

[751] Lau A, Bradshaw CS, Lewis D, The efficacy of azithromycin for the treatment of genital *Mycoplasma genitalium*: a systematic review and meta-analysis. Clin Infect Dis 2015;61:1389–99. 10.1093/cid/civ644 26240201

[752] Horner P. *Mycoplasma genitalium* nongonococcal urethritis is likely to increase in men who have sex with men who practice unsafe sex: what should we do? Sex Transm Dis 2019;46:518–20. 10.1097/OLQ.0000000000001030 31295219

[753] Hosenfeld CB, Workowski KA, Berman S, Repeat infection with Chlamydia and gonorrhea among females: a systematic review of the literature. Sex Transm Dis 2009;36:478–89. 10.1097/OLQ.0b013e3181a2a933 19617871

[754] Fung M, Scott KC, Kent CK, Klausner JD. Chlamydial and gonococcal reinfection among men: a systematic review of data to evaluate the need for retesting. Sex Transm Infect 2007;83:304–9. 10.1136/sti.2006.024059 17166889 PMC2598678

[755] Kissinger PJ, White S, Manhart LE, Azithromycin treatment failure for *Chlamydia trachomatis* among heterosexual men with nongonococcal urethritis. Sex Transm Dis 2016;43:599–602. 10.1097/OLQ.0000000000000489 27631353 PMC5033507

[756] Schwebke JR, Rompalo A, Taylor S, Re-evaluating the treatment of nongonococcal urethritis: emphasizing emerging pathogens—a randomized clinical trial. Clin Infect Dis 2011;52:163–70. 10.1093/cid/ciq074 21288838 PMC3106252

[757] Manhart LE, Khosropour CM, Gillespie CW, Lowens MS, Golden MR, Totten PA. O02.3 Treatment outcomes for persistent *Mycoplasma genitalium*-associated NGU: evidence of moxifloxacin treatment failures. Sex Transm Infect 2013;89(Suppl 1):A29. 10.1136/sextrans-2013-051184.0091

[758] Romano SS, Jensen JS, Lowens MS, Long duration of asymptomatic *Mycoplasma genitalium* infection after syndromic treatment for nongonococcal urethritis. Clin Infect Dis 2019;69:113–20. 10.1093/cid/ciy843 30281079 PMC6579957

[759] Read TRH, Fairley CK, Murray GL, Outcomes of resistance-guided sequential treatment of *Mycoplasma genitalium* infections: a prospective evaluation. Clin Infect Dis 2019;68:554–60. 10.1093/cid/ciy477 29873691 PMC6355821

[760] Dowe G, Smikle M, King SD, Baum M, Chout R, Williams Y. Symptomatic and asymptomatic chlamydial non-gonococcal urethritis in Jamaica: the potential for HIV transmission. Int J STD AIDS 2000;11:187–90. 10.1258/0956462001915507 10726944

[761] Lusk MJ, Garden FL, Rawlinson WD, Naing ZW, Cumming RG, Konecny P. Cervicitis aetiology and case definition: a study in Australian women attending sexually transmitted infection clinics. Sex Transm Infect 2016;92:175–81. 10.1136/sextrans-2015-052332 26586777

[762] Lusk MJ, Konecny P. Cervicitis: a review. Curr Opin Infect Dis 2008;21:49–55. 18192786 10.1097/QCO.0b013e3282f3d988

[763] Marrazzo JM, Martin DH. Management of women with cervicitis. Clin Infect Dis 2007;44(Suppl 3):S102–10. 10.1086/511423 17342663

[764] Manavi K, Young H, Clutterbuck D. Sensitivity of microscopy for the rapid diagnosis of gonorrhoea in men and women and the role of gonorrhoea serovars. Int J STD AIDS 2003;14:390–4. 10.1258/095646203765371277 12816666

[765] Lillis RA, Martin DH, Nsuami MJ. *Mycoplasma genitalium* infections in women attending a sexually transmitted disease clinic in New Orleans. Clin Infect Dis 2019;69:459–65. 10.1093/cid/ciy922 30351348

[766] Lis R, Rowhani-Rahbar A, Manhart LE. *Mycoplasma genitalium* infection and female reproductive tract disease: a meta-analysis. Clin Infect Dis 2015;61:418–26. 10.1093/cid/civ312 25900174

[767] Oliphant J, Azariah S. Cervicitis: limited clinical utility for the detection of *Mycoplasma genitalium* in a cross-sectional study of women attending a New Zealand sexual health clinic. Sex Health 2013;10:263–7. 10.1071/SH12168 23702105

[768] Sethi S, Rajkumari N, Dhaliwal L, Roy A. P3.294 Association of *Mycoplasma genitalium* with cervicitis in North Indian women attending gynecologic clinics. Sex Transm Infect 2013;89(Suppl 1):A240–1. 10.1136/sextrans-2013-051184.0749

[769] Taylor SN, Lensing S, Schwebke J, Prevalence and treatment outcome of cervicitis of unknown etiology. Sex Transm Dis 2013;40:379–85. 10.1097/OLQ.0b013e31828bfcb1 23588127 PMC3868214

[770] Mobley VL, Hobbs MM, Lau K, Weinbaum BS, Getman DK, Seña AC. *Mycoplasma genitalium* infection in women attending a sexually transmitted infection clinic: diagnostic specimen type, coinfections, and predictors. Sex Transm Dis 2012;39:706–9. 10.1097/OLQ.0b013e318255de03 22902666 PMC3428747

[771] Marrazzo JM, Wiesenfeld HC, Murray PJ, Risk factors for cervicitis among women with bacterial vaginosis. J Infect Dis 2006;193:617–24. 10.1086/500149 16453256

[772] Gaydos C, Maldeis NE, Hardick A, Hardick J, Quinn TC. *Mycoplasma genitalium* as a contributor to the multiple etiologies of cervicitis in women attending sexually transmitted disease clinics. Sex Transm Dis 2009;36:598–606. 10.1097/OLQ.0b013e3181b01948 19704398 PMC2924808

[773] Clark LR, Atendido M. Group B streptococcal vaginitis in postpubertal adolescent girls. J Adolesc Health 2005;36:437–40. 10.1016/j.jadohealth.2004.03.009 15837348

[774] Hester EE, Middleman AB. A clinical conundrum: chronic cervicitis. J Pediatr Adolesc Gynecol 2019;32:342–4. 10.1016/j.jpag.2018.12.004 30582974

[775] Liu L, Cao G, Zhao Z, Zhao F, Huang Y. High bacterial loads of *Ureaplasma* may be associated with non-specific cervicitis. Scand J Infect Dis 2014;46:637–41. 10.3109/00365548.2014.922696 25017795

[776] Leli C, Mencacci A, Latino MA, Prevalence of cervical colonization by *Ureaplasma parvum, Ureaplasma urealyticum, Mycoplasma hominis* and *Mycoplasma genitalium* in childbearing age women by a commercially available multiplex real-time PCR: an Italian observational multicentre study. J Microbiol Immunol Infect 2018;51:220–5. 10.1016/j.jmii.2017.05.004 28711440

[777] Manhart LE. Has the time come to systematically test for *Mycoplasma genitalium?* Sex Transm Dis 2009;36:607–8. 10.1097/OLQ.0b013e3181b9d825 19734818

[778] Liu HL, Chen CM, Pai LW, Hwu YJ, Lee HM, Chung YC. Comorbidity profiles among women with postcoital bleeding: a nationwide health insurance database. Arch Gynecol Obstet 2017;295:935–41. 10.1007/s00404-017-4327-7 28246983

[779] Coleman JS, Hitti J, Bukusi EA, Infectious correlates of HIV-1 shedding in the female upper and lower genital tracts. AIDS 2007;21:755–9. 10.1097/QAD.0b013e328012b838 17413697

[780] Johnson LF, Lewis DA. The effect of genital tract infections on HIV-1 shedding in the genital tract: a systematic review and meta-analysis. Sex Transm Dis 2008;35:946–59. 10.1097/OLQ.0b013e3181812d15 18685546

[781] McClelland RS, Wang CC, Mandaliya K, Treatment of cervicitis is associated with decreased cervical shedding of HIV-1. AIDS 2001;15:105–10. 10.1097/00002030-200101050-00015 11192850

[782] Gatski M, Martin DH, Theall K, *Mycoplasma genitalium* infection among HIV-positive women: prevalence, risk factors and association with vaginal shedding. Int J STD AIDS 2011;22:155–9. 10.1258/ijsa.2010.010320 21464453 PMC3778661

[783] Gitau RW, Graham SM, Masese LN, Effect of acquisition and treatment of cervical infections on HIV-1 shedding in women on antiretroviral therapy. AIDS 2010;24:2733–7. 10.1097/QAD.0b013e32833f9f43 20871388 PMC2978313

[784] Kreisel KM, Weston EJ, St Cyr SB, Spicknall IH. Estimates of the prevalence and incidence of chlamydia and gonorrhea among US men and women, 2018. Sex Transm Dis 2021;48:222–31. 10.1097/OLQ.0000000000001382 33492094 PMC10153658

[785] Aghaizu A, Reid F, Kerry S, Frequency and risk factors for incident and redetected *Chlamydia trachomatis* infection in sexually active, young, multi-ethnic women: a community based cohort study. Sex Transm Infect 2014;90:524–8. 10.1136/sextrans-2014-051607 25100744 PMC4215355

[786] Scholes D, Satterwhite CL, Yu O, Fine D, Weinstock H, Berman S. Long-term trends in *Chlamydia trachomatis* infections and related outcomes in a U.S. managed care population. Sex Transm Dis 2012;39:81–8. 10.1097/OLQ.0b013e31823e3009 22249294

[787] Kamwendo F, Forslin L, Bodin L, Danielsson D. Decreasing incidences of gonorrhea- and chlamydia-associated acute pelvic inflammatory disease. A 25-year study from an urban area of central Sweden. Sex Transm Dis 1996;23:384–91. 10.1097/00007435-199609000-00007 8885069

[788] Rietmeijer CA, Hopkins E, Geisler WM, Orr DP, Kent CK. *Chlamydia trachomatis* positivity rates among men tested in selected venues in the United States: a review of the recent literature. Sex Transm Dis 2008;35(Suppl):S8–18. 10.1097/OLQ.0b013e31816938ba 18449072

[789] Gift TL, Blake DR, Gaydos CA, Marrazzo JM. The cost-effectiveness of screening men for *Chlamydia trachomatis*: a review of the literature. Sex Transm Dis 2008;35(Suppl):S51–60. 10.1097/OLQ.0b013e3181723dba 18520977

[790] Gift TL, Gaydos CA, Kent CK, The program cost and cost-effectiveness of screening men for *Chlamydia* to prevent pelvic inflammatory disease in women. Sex Transm Dis 2008;35(Suppl):S66–75. 10.1097/OLQ.0b013e31818b64ac 18830137

[791] Gopalappa C, Huang YL, Gift TL, Owusu-Edusei K, Taylor M, Gales V. Cost-effectiveness of screening men in Maricopa County jails for chlamydia and gonorrhea to avert infections in women. Sex Transm Dis 2013;40:776–83. 10.1097/OLQ.0000000000000023 24275727 PMC4591034

[792] Masek BJ, Arora N, Quinn N, Performance of three nucleic acid amplification tests for detection of *Chlamydia trachomatis* and *Neisseria gonorrhoeae* by use of self-collected vaginal swabs obtained via an Internet-based screening program. J Clin Microbiol 2009;47:1663–7. 10.1128/JCM.02387-08 19386838 PMC2691063

[793] Knox J, Tabrizi SN, Miller P, Evaluation of self-collected samples in contrast to practitioner-collected samples for detection of *Chlamydia trachomatis, Neisseria gonorrhoeae*, and *Trichomonas vaginalis* by polymerase chain reaction among women living in remote areas. Sex Transm Dis 2002;29:647–54. 10.1097/00007435-200211000-00006 12438900

[794] Schachter J, Chernesky MA, Willis DE, Vaginal swabs are the specimens of choice when screening for *Chlamydia trachomatis* and *Neisseria gonorrhoeae*: results from a multicenter evaluation of the APTIMA assays for both infections. Sex Transm Dis 2005;32:725–8. 10.1097/01.olq.0000190092.59482.96 16314767

[795] Doshi JS, Power J, Allen E. Acceptability of chlamydia screening using self-taken vaginal swabs. Int J STD AIDS 2008;19:507–9. 10.1258/ijsa.2008.008056 18663033

[796] Chernesky MA, Jang D, Portillo E, Self-collected swabs of the urinary meatus diagnose more *Chlamydia trachomatis* and *Neisseria gonorrhoeae* infections than first catch urine from men. Sex Transm Infect 2013;89:102–4. 10.1136/sextrans-2012-050573 23024224

[797] Dize L, Barnes P Jr, Barnes M, Performance of self-collected penile-meatal swabs compared to clinician-collected urethral swabs for the detection of *Chlamydia trachomatis, Neisseria gonorrhoeae, Trichomonas vaginali*s, and *Mycoplasma genitalium* by nucleic acid amplification assays. Diagn Microbiol Infect Dis 2016;86:131–5. 10.1016/j.diagmicrobio.2016.07.018 27497595 PMC5028267

[798] Berry L, Stanley B. Comparison of self-collected meatal swabs with urine specimens for the diagnosis of *Chlamydia trachomatis* and *Neisseria gonorrhoeae* in men. J Med Microbiol 2017;66:134–6. 10.1099/jmm.0.000428 28068218

[799] Chernesky M, Freund GG, Hook E 3rd, Leone P, D’Ascoli P, Martens M. Detection of *Chlamydia trachomatis* and *Neisseria gonorrhoeae* infections in North American women by testing SurePath liquid-based Pap specimens in APTIMA assays. J Clin Microbiol 2007;45:2434–8. 10.1128/JCM.00013-07 17581931 PMC1951209

[800] Schachter J, Moncada J, Liska S, Shayevich C, Klausner JD. Nucleic acid amplification tests in the diagnosis of chlamydial and gonococcal infections of the oropharynx and rectum in men who have sex with men. Sex Transm Dis 2008;35:637–42. 10.1097/OLQ.0b013e31817bdd7e 18520976

[801] Mimiaga MJ, Mayer KH, Reisner SL, Asymptomatic gonorrhea and chlamydial infections detected by nucleic acid amplification tests among Boston area men who have sex with men. Sex Transm Dis 2008;35:495–8. 10.1097/OLQ.0b013e31816471ae 18354345

[802] Bachmann LH, Johnson RE, Cheng H, Markowitz LE, Papp JR, Hook EW 3rd. Nucleic acid amplification tests for diagnosis of *Neisseria gonorrhoeae* oropharyngeal infections. J Clin Microbiol 2009;47:902–7. 10.1128/JCM.01581-08 19193848 PMC2668347

[803] Bachmann LH, Johnson RE, Cheng H, Nucleic acid amplification tests for diagnosis of *Neisseria gonorrhoeae* and *Chlamydia trachomatis* rectal infections. J Clin Microbiol 2010;48:1827–32. 10.1128/JCM.02398-09 20335410 PMC2863861

[804] Cosentino LA, Danby CS, Rabe LK, Use of nucleic acid amplification testing for diagnosis of extragenital sexually transmitted infections. J Clin Microbiol 2017;55:2801–7. 10.1128/JCM.00616-17 28679521 PMC5648715

[805] Food and Drug Administration. Microbiology Devices Panel of the Medical Devices Advisory Committee meeting annoucement [Internet]. Silver Spring, MD: US Department of Agriculture, Food and Drug Administration; 2019. https://www.fda.gov/advisory-committees/advisory-committee-calendar/march-8-2019-microbiology-devices-panel-medical-devices-advisory-committee-meeting-announcement#event-materials

[806] Sexton ME, Baker JJ, Nakagawa K, How reliable is self-testing for gonorrhea and chlamydia among men who have sex with men? J Fam Pract 2013;62:70–8. 23405376 PMC12080727

[807] Herbst de Cortina S, Bristow CC, Joseph Davey D, Klausner JD. A systematic review of point of care testing for *Chlamydia trachomatis, Neisseria gonorrhoeae*, and *Trichomonas vaginalis.* Infect Dis Obstet Gynecol 2016;2016:4386127. Epub May 26, 2016. 10.1155/2016/4386127 27313440 PMC4899593

[808] Rivard KR, Dumkow LE, Draper HM, Brandt KL, Whalen DW, Egwuatu NE. Impact of rapid diagnostic testing for chlamydia and gonorrhea on appropriate antimicrobial utilization in the emergency department. Diagn Microbiol Infect Dis 2017;87:175–9. 10.1016/j.diagmicrobio.2016.10.019 27836225

[809] Wingrove I, McOwan A, Nwokolo N, Whitlock G. Diagnostics within the clinic to test for gonorrhoea and chlamydia reduces the time to treatment: a service evaluation. Sex Transm Infect 2014;90:474. 10.1136/sextrans-2014-051580 25118322

[810] Geisler WM, Wang C, Morrison SG, Black CM, Bandea CI, Hook EW 3rd. The natural history of untreated *Chlamydia trachomatis* infection in the interval between screening and returning for treatment. Sex Transm Dis 2008;35:119–23. 10.1097/OLQ.0b013e318151497d 17898680

[811] Dukers-Muijrers NHTM, Wolffs PFG, De Vries H, Treatment effectiveness of azithromycin and doxycycline in uncomplicated rectal and vaginal *Chlamydia trachomatis* infections in women: a multicentre observational study (FemCure). Clin Infect Dis 2019;69:1946–54. 10.1093/cid/ciz050 30689759 PMC6853690

[812] Dombrowski JC, Wierzbicki MR, Newman LM, Doxycycline versus azithromycin for the treatment of rectal chlamydia in men who have sex with men: a randomized controlled trial. Clin Infect Dis 2021;ciab153. 10.1093/cid/ciab153 33606009 PMC8571563

[813] Dukers-Muijrers NH, Schachter J, van Liere GA, Wolffs PF, Hoebe CJ. What is needed to guide testing for anorectal and pharyngeal *Chlamydia trachomatis* and *Neisseria gonorrhoeae* in women and men? Evidence and opinion. BMC Infect Dis 2015;15:533. 10.1186/s12879-015-1280-6 26576538 PMC4650297

[814] Marcus JL, Kohn RP, Barry PM, Philip SS, Bernstein KT. *Chlamydia trachomatis* and *Neisseria gonorrhoeae* transmission from the female oropharynx to the male urethra. Sex Transm Dis 2011;38:372–3. 10.1097/OLQ.0b013e3182029008 21183864

[815] Manavi K, Hettiarachchi N, Hodson J. Comparison of doxycycline with azithromycin in treatment of pharyngeal chlamydia infection. Int J STD AIDS 2016;27:1303–8. 10.1177/0956462415614723 26511655

[816] Rank RG, Yeruva L. An alternative scenario to explain rectal positivity in *Chlamydia*-infected individuals. Clin Infect Dis 2015;60:1585–6. 10.1093/cid/civ079 25648236

[817] Geisler WM, Koltun WD, Abdelsayed N, Safety and efficacy of WC2031 versus vibramycin for the treatment of uncomplicated urogenital *Chlamydia trachomatis* infection: a randomized, double-blind, double-dummy, active-controlled, multicenter trial. Clin Infect Dis 2012;55:82–8. 10.1093/cid/cis291 22431798

[818] Renault CA, Israelski DM, Levy V, Fujikawa BK, Kellogg TA, Klausner JD. Time to clearance of *Chlamydia trachomatis* ribosomal RNA in women treated for chlamydial infection. Sex Health 2011;8:69–73. 10.1071/SH10030 21371385

[819] Lazenby GB, Korte JE, Tillman S, Brown FK, Soper DE. A recommendation for timing of repeat *Chlamydia trachomatis* test following infection and treatment in pregnant and nonpregnant women. Int J STD AIDS 2017;28:902–9. 10.1177/0956462416680438 27864473 PMC5798859

[820] Dunne EF, Chapin JB, Rietmeijer CA, Rate and predictors of repeat *Chlamydia trachomatis* infection among men. Sex Transm Dis 2008;35(Suppl):S40–4. 10.1097/OLQ.0b013e31817247b2 18520978

[821] Kjaer HO, Dimcevski G, Hoff G, Olesen F, Ostergaard L. Recurrence of urogenital *Chlamydia trachomatis* infection evaluated by mailed samples obtained at home: 24 weeks’ prospective follow up study. Sex Transm Infect 2000;76:169–72. 10.1136/sti.76.3.169 10961191 PMC1744142

[822] Whittington WL, Kent C, Kissinger P, Determinants of persistent and recurrent *Chlamydia trachomatis* infection in young women: results of a multicenter cohort study. Sex Transm Dis 2001;28:117–23. 10.1097/00007435-200102000-00011 11234786

[823] Kapil R, Press CG, Hwang ML, Brown L, Geisler WM. Investigating the epidemiology of repeat *Chlamydia trachomatis* detection after treatment by using *C. trachomatis* OmpA genotyping. J Clin Microbiol 2015;53:546–9. 10.1128/JCM.02483-14 25472488 PMC4298497

[824] Jacobson GF, Autry AM, Kirby RS, Liverman EM, Motley RU. A randomized controlled trial comparing amoxicillin and azithromycin for the treatment of *Chlamydia trachomatis* in pregnancy. Am J Obstet Gynecol 2001;184:1352–6. 10.1067/mob.2001.115050 11408852

[825] Kacmar J, Cheh E, Montagno A, Peipert JF. A randomized trial of azithromycin versus amoxicillin for the treatment of *Chlamydia trachomatis* in pregnancy. Infect Dis Obstet Gynecol 2001;9:197–202. 10.1155/S1064744901000321 11916175 PMC1784654

[826] Rahangdale L, Guerry S, Bauer HM, An observational cohort study of *Chlamydia trachomatis* treatment in pregnancy. Sex Transm Dis 2006;33:106–10. 10.1097/01.olq.0000187226.32145.ea 16432482

[827] Aggarwal A, Spitzer RF, Caccia N, Stephens D, Johnstone J, Allen L. Repeat screening for sexually transmitted infection in adolescent obstetric patients. J Obstet Gynaecol Can 2010;32:956–61. 10.1016/S1701-2163(16)34683-7 21176304

[828] Phillips Campbell R, Kintner J, Whittimore J, Schoborg RV. *Chlamydia muridarum* enters a viable but non-infectious state in amoxicillin-treated BALB/c mice. Microbes Infect 2012;14:1177–85. 10.1016/j.micinf.2012.07.017 22943883 PMC3654801

[829] Wyrick PB. *Chlamydia trachomatis* persistence in vitro: an overview. J Infect Dis 2010;201(Suppl 2):S88–95. 10.1086/652394 20470046 PMC2878585

[830] Fan H, Li L, Wijlaars L, Gilbert RE. Associations between use of macrolide antibiotics during pregnancy and adverse child outcomes: a systematic review and meta-analysis. PLoS One 2019;14:e0212212. 10.1371/journal.pone.0212212 30779772 PMC6380581

[831] Fan H, Gilbert R, O’Callaghan F, Li L. Associations between macrolide antibiotics prescribing during pregnancy and adverse child outcomes in the UK: population based cohort study. BMJ 2020;368:m331. Erratum in: BMJ 2020;368:m766. 10.1136/bmj.m331 32075790 PMC7190043

[832] Mallah N, Tohidinik HR, Etminan M, Figueiras A, Takkouche B. Prenatal exposure to macrolides and risk of congenital malformations: a meta-analysis. Drug Saf 2020;43:211–21. 10.1007/s40264-019-00884-5 31721138

[833] Hammerschlag MR, Cummings C, Roblin PM, Williams TH, Delke I. Efficacy of neonatal ocular prophylaxis for the prevention of chlamydial and gonococcal conjunctivitis. N Engl J Med 1989;320:769–72. 10.1056/NEJM198903233201204 2922026

[834] Zikic A, Schünemann H, Wi T, Lincetto O, Broutet N, Santesso N. Treatment of neonatal chlamydial conjunctivitis: a systematic review and meta-analysis. J Pediatric Infect Dis Soc 2018;7:e107–15. 10.1093/jpids/piy060 30007329 PMC6097578

[835] Hammerschlag MR, Chandler JW, Alexander ER, English M, Koutsky L. Longitudinal studies on chlamydial infections in the first year of life. Pediatr Infect Dis 1982;1:395–401. 10.1097/00006454-198211000-00007 7163029

[836] Beem MO, Saxon E, Tipple MA. Treatment of chlamydial pneumonia of infancy. Pediatrics 1979;63:198–203. 440807

[837] Brownell AD, Shapiro RA, Hammerschlag MR. Caution is required when using non-Food and Drug Administration-cleared assays to diagnose sexually transmitted infections in children. J Pediatr 2019;206:280–2. 10.1016/j.jpeds.2018.10.038 30466791

[838] Kreisel KM, Spicknall IH, Gargano JW, Sexually transmitted infections among US women and men: prevalence and incidence estimates, 2018. Sex Transm Dis 2021;48:208–14. 10.1097/OLQ.0000000000001355 33492089 PMC10245608

[839] Lunny C, Taylor D, Hoang L, Self-collected versus clinician-collected sampling for chlamydia and gonorrhea screening: a systemic review and meta-analysis. PLoS One 2015;10:e0132776. 10.1371/journal.pone.0132776 26168051 PMC4500554

[840] Schick V, Van Der Pol B, Dodge B, Baldwin A, Fortenberry JD. A mixed methods approach to assess the likelihood of testing for STI using self-collected samples among behaviourally bisexual women. Sex Transm Infect 2015;91:329–33. 10.1136/sextrans-2014-051842 25637328

[841] Mustanski B, Feinstein BA, Madkins K, Sullivan P, Swann G. Prevalence and risk factors for rectal and urethral sexually transmitted infections from self-collected samples among young men who have sex with men participating in the Keep It Up! 2.0 randomized controlled trial. Sex Transm Dis 2017;44:483–8. 10.1097/OLQ.0000000000000636 28703727 PMC5821498

[842] Salow KR, Cohen AC, Bristow CC, McGrath MR, Klausner JD. Comparing mail-in self-collected specimens sent via United States Postal Service versus clinic-collected specimens for the detection of *Chlamydia trachomatis* and *Neisseria gonorrhoeae* in extra-genital sites. PLoS One 2017;12:e0189515. 10.1371/journal.pone.0189515 29240781 PMC5730150

[843] Drake C, Barenfanger J, Lawhorn J, Verhulst S. Comparison of Easy-Flow Copan Liquid Stuart’s and Starplex Swab transport systems for recovery of fastidious aerobic bacteria. J Clin Microbiol 2005;43:1301–3. 10.1128/JCM.43.3.1301-1303.2005 15750099 PMC1081218

[844] Wade JJ, Graver MA. Survival of six auxotypes of *Neisseria gonorrhoeae* in transport media. J Clin Microbiol 2003;41:1720–1. 10.1128/JCM.41.4.1720-1721.2003 12682168 PMC153907

[845] Arbique JC, Forward KR, LeBlanc J. Evaluation of four commercial transport media for the survival of *Neisseria gonorrhoeae.* Diagn Microbiol Infect Dis 2000;36:163–8. 10.1016/S0732-8893(99)00134-0 10729658

[846] Hook EW 3rd, Kirkcaldy RD. A brief history of evolving diagnostics and therapy for gonorrhea: lessons learned. Clin Infect Dis 2018;67:1294–9. 10.1093/cid/ciy271 29659749 PMC6452490

[847] Unemo M, Shafer WM. Future treatment of gonorrhea—novel emerging drugs are essential and in progress? Expert Opin Emerg Drugs 2015;20:357–60. 10.1517/14728214.2015.1039981 25907334 PMC4550495

[848] Sánchez-Busó L, Golparian D, Corander J, The impact of antimicrobials on gonococcal evolution. Nat Microbiol 2019;4:1941–50. 10.1038/s41564-019-0501-y 31358980 PMC6817357

[849] Schwarcz SK, Zenilman JM, Schnell D, National surveillance of antimicrobial resistance in *Neisseria gonorrhoeae.* The Gonococcal Isolate Surveillance Project. JAMA 1990;264:1413–7. 10.1001/jama.1990.03450110059027 2144026

[850] CDC. Update to CDC’s sexually transmitted diseases treatment guidelines, 2006: fluoroquinolones no longer recommended for treatment of gonococcal infections. MMWR Morb Mortal Wkly Rep 2007;56:332–6. 17431378

[851] CDC. Sexually transmitted disease surveillance 2013. Atlanta, GA: US Department of Health and Human Services, CDC; 2014. https://www.cdc.gov/std/stats/archive/Surv2013-Print.pdf

[852] Muratani T, Akasaka S, Kobayashi T, Outbreak of cefozopran (penicillin, oral cephems, and aztreonam)-resistant *Neisseria gonorrhoeae* in Japan. Antimicrob Agents Chemother 2001;45:3603–6. 10.1128/AAC.45.12.3603-3606.2001 11709349 PMC90878

[853] Yokoi S, Deguchi T, Ozawa T, Threat to cefixime treatment for gonorrhea. Emerg Infect Dis 2007;13:1275–7. 17953118 10.3201/eid1308.060948 PMC2828067

[854] Lo JY, Ho KM, Leung AO, Ceftibuten resistance and treatment failure of *Neisseria gonorrhoeae* infection. Antimicrob Agents Chemother 2008;52:3564–7. 10.1128/AAC.00198-08 18663018 PMC2565891

[855] Deguchi T, Yasuda M, Yokoi S, Treatment of uncomplicated gonococcal urethritis by double-dosing of 200 mg cefixime at a 6-h interval. J Infect Chemother 2003;9:35–9. 10.1007/s10156-002-0204-8 12673405

[856] Unemo M, Golparian D, Hestner A. Ceftriaxone treatment failure of pharyngeal gonorrhoea verified by international recommendations, Sweden, July 2010. Euro Surveill 2011;16:19792. 21329645

[857] Unemo M, Golparian D, Syversen G, Vestrheim DF, Moi H. Two cases of verified clinical failures using internationally recommended first-line cefixime for gonorrhoea treatment, Norway, 2010. Euro Surveill 2010;15:19721. 10.2807/ese.15.47.19721-en 21144442

[858] Unemo M, Golparian D, Potočnik M, Jeverica S. Treatment failure of pharyngeal gonorrhoea with internationally recommended first-line ceftriaxone verified in Slovenia, September 2011. Euro Surveill 2012;17:20200. 22748003

[859] Ison CA, Hussey J, Sankar KN, Evans J, Alexander S. Gonorrhoea treatment failures to cefixime and azithromycin in England, 2010. Euro Surveill 2011;16:19833. 21492528

[860] Forsyth S, Penney P, Rooney G. Cefixime-resistant *Neisseria gonorrhoeae* in the UK: a time to reflect on practice and recommendations. Int J STD AIDS 2011;22:296–7. 10.1258/ijsa.2009.009191 21571983

[861] Lewis DA, Sriruttan C, Müller EE, Phenotypic and genetic characterization of the first two cases of extended-spectrum-cephalosporin-resistant *Neisseria gonorrhoeae* infection in South Africa and association with cefixime treatment failure. J Antimicrob Chemother 2013;68:1267–70. 10.1093/jac/dkt034 23416957

[862] Ota KV, Fisman DN, Tamari IE, Incidence and treatment outcomes of pharyngeal *Neisseria gonorrhoeae* and *Chlamydia trachomatis* infections in men who have sex with men: a 13-year retrospective cohort study. Clin Infect Dis 2009;48:1237–43. 10.1086/597586 19323630

[863] Allen VG, Mitterni L, Seah C, *Neisseria gonorrhoeae* treatment failure and susceptibility to cefixime in Toronto, Canada. JAMA 2013;309:163–70. 10.1001/jama.2012.176575 23299608

[864] Chen MY, Stevens K, Tideman R, Failure of 500 mg of ceftriaxone to eradicate pharyngeal gonorrhoea, Australia. J Antimicrob Chemother 2013;68:1445–7. 10.1093/jac/dkt017 23390207

[865] Tapsall J, Read P, Carmody C, Two cases of failed ceftriaxone treatment in pharyngeal gonorrhoea verified by molecular microbiological methods. J Med Microbiol 2009;58:683–7. 10.1099/jmm.0.007641-0 19369534

[866] Ohnishi M, Saika T, Hoshina S, Ceftriaxone-resistant *Neisseria gonorrhoeae*, Japan. Emerg Infect Dis 2011;17:148–9. 10.3201/eid1701.100397 21192886 PMC3204624

[867] Unemo M, Golparian D, Nicholas R, Ohnishi M, Gallay A, Sednaoui P. High-level cefixime- and ceftriaxone-resistant *Neisseria gonorrhoeae* in France: novel penA mosaic allele in a successful international clone causes treatment failure. Antimicrob Agents Chemother 2012;56:1273–80. 10.1128/AAC.05760-11 22155830 PMC3294892

[868] CDC. Update to CDC’s sexually transmitted diseases treatment guidelines, 2010: oral cephalosporins no longer a recommended treatment for gonococcal infections. MMWR Morb Mortal Wkly Rep 2012;61:590–4. 22874837

[869] Wind CM, de Vries E, Schim van der Loeff MF, Decreased azithromycin susceptibility of *Neisseria gonorrhoeae* isolates in patients recently treated with azithromycin. Clin Infect Dis 2017;65:37–45. 10.1093/cid/cix249 28510723

[870] Kong FYS, Horner P, Unemo M, Hocking JS. Pharmacokinetic considerations regarding the treatment of bacterial sexually transmitted infections with azithromycin: a review. J Antimicrob Chemother 2019;74:1157–66. 10.1093/jac/dky548 30649333

[871] CDC. Antibiotic resistance threats in the United States, 2019. Atlanta, GA: US Department of Health and Human Services, CDC; 2019. https://www.cdc.gov/drugresistance/pdf/threats-report/2019-ar-threats-report-508.pdf

[872] St Cyr S, Barbee L, Workowski KA, Update to CDC’s treatment guidelines for gonococcal infection, 2020. MMWR Morb Mortal Wkly Rep 2020;69:1911–6. 10.15585/mmwr.mm6950a6 33332296 PMC7745960

[873] Clinical and Laboratory Standards Institute. Performance standards for antimicrobial susceptibility testing: twentieth informational supplement. Clinical and Laboratory Standards Institute document M100-S20. Wayne, PA: Clinical and Laboratory Standards Institute; 2010.

[874] CDC. Cephalosporin-resistant *Neisseria gonorrhoeae* public health response plan. Atlanta, GA: US Department of Health and Human Services, CDC; 2012. https://www.cdc.gov/std/treatment/Ceph-R-ResponsePlanJuly30-2012.pdf

[875] Poncin T, Merimeche M, Braille A, Two cases of multidrug-resistant *Neisseria gonorrhoeae* related to travel in south-eastern Asia, France, June 2019. Euro Surveill 2019;24:1900528. 10.2807/1560-7917.ES.2019.24.36.1900528 31507264 PMC6737829

[876] Carnicer-Pont D, Smithson A, Fina-Homar E, Bastida MT; Gonococcus Antimicrobial Resistance Surveillance Working Group. First cases of *Neisseria gonorrhoeae* resistant to ceftriaxone in Catalonia, Spain, May 2011. Enferm Infecc Microbiol Clin 2012;30:218–9. 10.1016/j.eimc.2011.11.010 22244992

[877] Cámara J, Serra J, Ayats J, Molecular characterization of two high-level ceftriaxone-resistant *Neisseria gonorrhoeae* isolates detected in Catalonia, Spain. J Antimicrob Chemother 2012;67:1858–60. 10.1093/jac/dks162 22566592

[878] Eyre DW, Sanderson ND, Lord E, Gonorrhoea treatment failure caused by a *Neisseria gonorrhoeae* strain with combined ceftriaxone and high-level azithromycin resistance, England, February 2018. Euro Surveill 2018;23:1800323. 10.2807/1560-7917.ES.2018.23.27.1800323 29991383 PMC6152157

[879] Fifer H, Hughes G, Whiley D, Lahra MM. Lessons learnt from ceftriaxone-resistant gonorrhoea in the UK and Australia. Lancet Infect Dis 2020;20:276–8. 10.1016/S1473-3099(20)30055-4 32112753

[880] Chisholm SA, Mouton JW, Lewis DA, Nichols T, Ison CA, Livermore DM. Cephalosporin MIC creep among gonococci: time for a pharmacodynamic rethink? J Antimicrob Chemother 2010;65:2141–8. 10.1093/jac/dkq289 20693173

[881] Connolly KL, Eakin AE, Gomez C, Osborn BL, Unemo M, Jerse AE. Pharmacokinetic data are predictive of in vivo efficacy for cefixime and ceftriaxone against susceptible and resistant *Neisseria gonorrhoeae* strains in the gonorrhea mouse model. Antimicrob Agents Chemother 2019;63:e01644-18. 10.1128/AAC.01644-18 30642924 PMC6395893

[882] Blondeau JM, Hansen G, Metzler K, Hedlin P. The role of PK/PD parameters to avoid selection and increase of resistance: mutant prevention concentration. J Chemother 2004;16(Suppl 3):1–19. 10.1080/1120009X.2004.11782371 15334827

[883] Moran JS, Levine WC. Drugs of choice for the treatment of uncomplicated gonococcal infections. Clin Infect Dis 1995;20(Suppl 1):S47–65. 10.1093/clinids/20.Supplement_1.S47 7795109

[884] Unemo M, Golparian D, Eyre DW. Antimicrobial resistance in *Neisseria gonorrhoeae* and treatment of gonorrhea. Methods Mol Biol 2019;1997:37–58. 10.1007/978-1-4939-9496-0_3 31119616

[885] Kirkcaldy RD, Weinstock HS, Moore PC, The efficacy and safety of gentamicin plus azithromycin and gemifloxacin plus azithromycin as treatment of uncomplicated gonorrhea. Clin Infect Dis 2014;59:1083–91. 10.1093/cid/ciu521 25031289 PMC4271098

[886] Ross JDC, Brittain C, Cole M, ; G-ToG trial team. Gentamicin compared with ceftriaxone for the treatment of gonorrhoea (G-ToG): a randomised non-inferiority trial. Lancet 2019;393:2511–20. 10.1016/S0140-6736(18)32817-4 31056291 PMC6620599

[887] Singh V, Bala M, Bhargava A, Kakran M, Bhatnagar R. In vitro efficacy of 21 dual antimicrobial combinations comprising novel and currently recommended combinations for treatment of drug resistant gonorrhoea in future era. PLoS One 2018;13:e0193678. 10.1371/journal.pone.0193678 29509792 PMC5839552

[888] Mayer KH, Klausner JD, Handsfield HH. Intersecting epidemics and educable moments: sexually transmitted disease risk assessment and screening in men who have sex with men. Sex Transm Dis 2001;28:464–7. 10.1097/00007435-200108000-00008 11473219

[889] Linhart Y, Shohat T, Amitai Z, Sexually transmitted infections among brothel-based sex workers in Tel-Aviv area, Israel: high prevalence of pharyngeal gonorrhoea. Int J STD AIDS 2008;19:656–9. 10.1258/ijsa.2008.008127 18824615

[890] Johnson Jones ML, Chapin-Bardales J, Bizune D, ; National HIV Behavioral Surveillance Sexually Transmitted Infection Study Group. Extragenital chlamydia and gonorrhea among community venue-attending men who have sex with men—five cities, United States, 2017. MMWR Morb Mortal Wkly Rep 2019;68:321–5. 10.15585/mmwr.mm6814a1 30973847 PMC6459584

[891] Chow EP, Williamson DA, Fortune R, Prevalence of genital and oropharyngeal chlamydia and gonorrhoea among female sex workers in Melbourne, Australia, 2015–2017: need for oropharyngeal testing. Sex Transm Infect 2019;95:398–401. 10.1136/sextrans-2018-053957 31113904

[892] Cornelisse VJ, Williamson D, Zhang L, Evidence for a new paradigm of gonorrhoea transmission: cross-sectional analysis of *Neisseria gonorrhoeae* infections by anatomical site in both partners in 60 male couples. Sex Transm Infect 2019;95:437–42. 10.1136/sextrans-2018-053803 30996106

[893] Kissinger PJ, Reilly K, Taylor SN, Leichliter JS, Rosenthal S, Martin DH. Early repeat *Chlamydia trachomatis* and *Neisseria gonorrhoeae* infections among heterosexual men. Sex Transm Dis 2009;36:498–500. 10.1097/OLQ.0b013e3181a4d147 19617870 PMC3778679

[894] Berenger BM, Demczuk W, Gratrix J, Pabbaraju K, Smyczek P, Martin I. Genetic characterization and enhanced surveillance of ceftriaxone-resistant *Neisseria gonorrhoeae* strain, Alberta, Canada, 2018. Emerg Infect Dis 2019;25:1660–7. 10.3201/eid2509.190407 31407661 PMC6711210

[895] Rob F, Klubalová B, Nyčová E, Hercogová J, Unemo M. Gentamicin 240 mg plus azithromycin 2 g vs. ceftriaxone 500 mg plus azithromycin 2 g for treatment of rectal and pharyngeal gonorrhoea: a randomized controlled trial. Clin Microbiol Infect 2020;26:207–12. 10.1016/j.cmi.2019.08.004 31419483

[896] Romano A, Gaeta F, Valluzzi RL, Caruso C, Rumi G, Bousquet PJ. IgE-mediated hypersensitivity to cephalosporins: cross-reactivity and tolerability of penicillins, monobactams, and carbapenems. J Allergy Clin Immunol 2010;126:994–9. 10.1016/j.jaci.2010.06.052 20888035

[897] American College of Obstetricians and Gynecologists’ Committee on Obstetric Practice. Committee opinion No. 717: sulfonamides, nitrofurantoin, and risk of birth defects. Obstet Gynecol 2017;130:e150–2. 10.1097/AOG.0000000000002300 28832488

[898] Haimovici R, Roussel TJ. Treatment of gonococcal conjunctivitis with single-dose intramuscular ceftriaxone. Am J Ophthalmol 1989;107:511–4. 10.1016/0002-9394(89)90495-9 2496606

[899] Bleich AT, Sheffield JS, Wendel GD Jr, Sigman A, Cunningham FG. Disseminated gonococcal infection in women. Obstet Gynecol 2012;119:597–602. 10.1097/AOG.0b013e318244eda9 22353959

[900] Belkacem A, Caumes E, Ouanich J, ; Working Group FRA-DGI. Changing patterns of disseminated gonococcal infection in France: cross-sectional data 2009–2011. Sex Transm Infect 2013;89:613–5. 10.1136/sextrans-2013-051119 23920397

[901] Birrell JM, Gunathilake M, Singleton S, Williams S, Krause V. Characteristics and impact of disseminated gonococcal infection in the “Top End” of Australia. Am J Trop Med Hyg 2019;101:753–60. 10.4269/ajtmh.19-0288 31392956 PMC6779203

[902] Crew PE, Abara WE, McCulley L, Disseminated gonococcal infections in patients receiving eculizumab: a case series. Clin Infect Dis 2019;69:596–600. 10.1093/cid/ciy958 30418536 PMC6744347

[903] Curry SJ, Krist AH, Owens DK, ; US Preventive Services Task Force. Ocular prophylaxis for gonococcal ophthalmia neonatorum: US Preventive Services Task Force reaffirmation recommendation statement. JAMA 2019;321:394–8. 10.1001/jama.2018.21367 30694327

[904] Kreisel K, Weston E, Braxton J, Llata E, Torrone E. Keeping an eye on chlamydia and gonorrhea conjunctivitis in infants in the United States, 2010–2015. Sex Transm Dis 2017;44:356–8. 10.1097/OLQ.0000000000000613 28499285 PMC5527667

[905] Scott WJ, Eck CD. Povidone-iodine and ophthalmia neonatorum. Ophthalmology 2012;119:653–4. 10.1016/j.ophtha.2011.11.037 22385492

[906] David M, Rumelt S, Weintraub Z. Efficacy comparison between povidone iodine 2.5% and tetracycline 1% in prevention of ophthalmia neonatorum. Ophthalmology 2011;118:1454–8. 10.1016/j.ophtha.2010.12.003 21439642

[907] Binenbaum G, Bruno CJ, Forbes BJ, Periocular ulcerative dermatitis associated with gentamicin ointment prophylaxis in newborns. J Pediatr 2010;156:320–1. 10.1016/j.jpeds.2009.11.060 20105641 PMC2828447

[908] Nathawad R, Mendez H, Ahmad A, Severe ocular reactions after neonatal ocular prophylaxis with gentamicin ophthalmic ointment. Pediatr Infect Dis J 2011;30:175–6. 10.1097/INF.0b013e3181f6c2e5 20885334

[909] Taylor-Robinson D, Jensen JS. *Mycoplasma genitalium*: from chrysalis to multicolored butterfly. Clin Microbiol Rev 2011;24:498–514. 10.1128/CMR.00006-11 21734246 PMC3131060

[910] Seña AC, Lensing S, Rompalo A, *Chlamydia trachomatis, Mycoplasma genitalium*, and *Trichomonas vaginalis* infections in men with nongonococcal urethritis: predictors and persistence after therapy. J Infect Dis 2012;206:357–65. 10.1093/infdis/jis356 22615318 PMC3490700

[911] Huppert JS, Mortensen JE, Reed JL, Kahn JA, Rich KD, Hobbs MM. *Mycoplasma genitalium* detected by transcription-mediated amplification is associated with *Chlamydia trachomatis* in adolescent women. Sex Transm Dis 2008;35:250–4. 10.1097/OLQ.0b013e31815abac6 18490867 PMC3807598

[912] Mena L, Wang X, Mroczkowski TF, Martin DH. *Mycoplasma genitalium* infections in asymptomatic men and men with urethritis attending a sexually transmitted diseases clinic in New Orleans. Clin Infect Dis 2002;35:1167–73. 10.1086/343829 12410476

[913] Falk L. The overall agreement of proposed definitions of mucopurulent cervicitis in women at high risk of Chlamydia infection. Acta Derm Venereol 2010;90:506–11. 10.2340/00015555-0924 20814628

[914] Anagrius C, Loré B, Jensen JS. *Mycoplasma genitalium*: prevalence, clinical significance, and transmission. Sex Transm Infect 2005;81:458–62. 10.1136/sti.2004.012062 16326846 PMC1745067

[915] Manhart LE, Critchlow CW, Holmes KK, Mucopurulent cervicitis and *Mycoplasma genitalium.* J Infect Dis 2003;187:650–7. 10.1086/367992 12599082

[916] Lusk MJ, Konecny P, Naing ZW, Garden FL, Cumming RG, Rawlinson WD. *Mycoplasma genitalium* is associated with cervicitis and HIV infection in an urban Australian STI clinic population. Sex Transm Infect 2011;87:107–9. 10.1136/sti.2010.045138 21071566

[917] Dehon PM, McGowin CL. The immunopathogenesis of *Mycoplasma genitalium* infections in women: a narrative review. Sex Transm Dis 2017;44:428–32. 10.1097/OLQ.0000000000000621 28608793 PMC5470585

[918] Bjartling C, Osser S, Persson K. The association between *Mycoplasma genitalium* and pelvic inflammatory disease after termination of pregnancy. BJOG 2010;117:361–4. 10.1111/j.1471-0528.2009.02455.x 20015303

[919] Bjartling C, Osser S, Persson K. *Mycoplasma genitalium* in cervicitis and pelvic inflammatory disease among women at a gynecologic outpatient service. Am J Obstet Gynecol 2012;206:476.e1–8. 10.1016/j.ajog.2012.02.036 22483084

[920] Taylor BD, Zheng X, O’Connell CM, Wiesenfeld HC, Hillier SL, Darville T. Risk factors for *Mycoplasma genitalium* endometritis and incident infection: a secondary data analysis of the T cell Response Against Chlamydia (TRAC) Study. Sex Transm Infect 2018;94:414–20. 10.1136/sextrans-2017-053376 29563165 PMC6295147

[921] Cohen CR, Mugo NR, Astete SG, Detection of *Mycoplasma genitalium* in women with laparoscopically diagnosed acute salpingitis. Sex Transm Infect 2005;81:463–6. 10.1136/sti.2005.015701 16326847 PMC1745055

[922] Haggerty CL, Totten PA, Astete SG, Ness RB. *Mycoplasma genitalium* among women with nongonococcal, nonchlamydial pelvic inflammatory disease. Infect Dis Obstet Gynecol 2006;2006:30184. 10.1155/IDOG/2006/30184 17485798 PMC1581464

[923] Short VL, Totten PA, Ness RB, Astete SG, Kelsey SF, Haggerty CL. Clinical presentation of *Mycoplasma genitalium* infection versus *Neisseria gonorrhoeae* infection among women with pelvic inflammatory disease. Clin Infect Dis 2009;48:41–7. 10.1086/594123 19025498 PMC2652068

[924] Simms I, Eastick K, Mallinson H, Associations between *Mycoplasma genitalium, Chlamydia trachomatis*, and pelvic inflammatory disease. Sex Transm Infect 2003;79:154–6. 10.1136/sti.79.2.154 12690141 PMC1744630

[925] Oakeshott P, Aghaizu A, Hay P, Is *Mycoplasma genitalium* in women the “New Chlamydia?” A community-based prospective cohort study. Clin Infect Dis 2010;51:1160–6. 10.1086/656739 20942656

[926] Wiesenfeld HC, Hillier SL, Meyn L, *Mycoplasma genitalium*—is it a pathogen in acute pelvic inflammatory disease (PID)? Sex Transm Infect 2013;89(Suppl 1):A34. 10.1136/sextrans-2013-051184.0106

[927] Møller BR, Taylor-Robinson D, Furr PM, Freundt EA. Acute upper genital-tract disease in female monkeys provoked experimentally by *Mycoplasma genitalium.* Br J Exp Pathol 1985;66:417–26. 4027175 PMC2041089

[928] Wiesenfeld HC, Manhart LE. *Mycoplasma genitalium* in women: current knowledge and research priorities for this recently emerged pathogen. J Infect Dis 2017;216(suppl_2):S389–95. 28838078 10.1093/infdis/jix198 PMC5853983

[929] Clausen HF, Fedder J, Drasbek M, Serological investigation of *Mycoplasma genitalium* in infertile women. Hum Reprod 2001;16:1866–74. 10.1093/humrep/16.9.1866 11527890

[930] Svenstrup HF, Fedder J, Kristoffersen SE, Trolle B, Birkelund S, Christiansen G. *Mycoplasma genitalium, Chlamydia trachomatis*, and tubal factor infertility—a prospective study. Fertil Steril 2008;90:513–20. 10.1016/j.fertnstert.2006.12.056 17548070

[931] Idahl A, Jurstrand M, Olofsson JI, Fredlund H. *Mycoplasma genitalium* serum antibodies in infertile couples and fertile women. Sex Transm Infect 2015;91:589–91. 10.1136/sextrans-2015-052011 25921018

[932] Edwards RK, Ferguson RJ, Reyes L, Brown M, Theriaque DW, Duff P. Assessing the relationship between preterm delivery and various microorganisms recovered from the lower genital tract. J Matern Fetal Neonatal Med 2006;19:357–63. 10.1080/00207170600712071 16801313

[933] Vandepitte J, Bukenya J, Hughes P, Clinical characteristics associated with *Mycoplasma genitalium* infection among women at high risk of HIV and other STI in Uganda. Sex Transm Dis 2012;39:487–91. 10.1097/OLQ.0b013e31824b1cf3 22592838

[934] Rowlands S, Danielewski JA, Tabrizi SN, Walker SP, Garland SM. Microbial invasion of the amniotic cavity in midtrimester pregnancies using molecular microbiology. Am J Obstet Gynecol 2017;217:71.e1–5. 10.1016/j.ajog.2017.02.051 28268197

[935] Jurstrand M, Jensen JS, Magnuson A, Kamwendo F, Fredlund H. A serological study of the role of *Mycoplasma genitalium* in pelvic inflammatory disease and ectopic pregnancy. Sex Transm Infect 2007;83:319–23. 10.1136/sti.2007.024752 17475688 PMC2598688

[936] Ashshi AM, Batwa SA, Kutbi SY, Malibary FA, Batwa M, Refaat B. Prevalence of 7 sexually transmitted organisms by multiplex real-time PCR in Fallopian tube specimens collected from Saudi women with and without ectopic pregnancy. BMC Infect Dis 2015;15:569. 10.1186/s12879-015-1313-1 26666587 PMC4678466

[937] Bissessor M, Tabrizi SN, Bradshaw CS, The contribution of *Mycoplasma genitalium* to the aetiology of sexually acquired infectious proctitis in men who have sex with men. Clin Microbiol Infect 2016;22:260–5. 10.1016/j.cmi.2015.11.016 26686807

[938] Ong JJ, Aung E, Read TRH, Clinical characteristics of anorectal *Mycoplasma genitalium* infection and microbial cure in men who have sex with men. Sex Transm Dis 2018;45:522–6. 10.1097/OLQ.0000000000000793 29465653

[939] Read TRH, Murray GL, Danielewski JA, symptoms, sites, and significance of *Mycoplasma genitalium* in men who have sex with men. Emerg Infect Dis 2019;25:719–27. 10.3201/eid2504.181258 30882306 PMC6433010

[940] Cina M, Baumann L, Egli-Gany D, *Mycoplasma genitalium* incidence, persistence, concordance between partners and progression: systematic review and meta-analysis. Sex Transm Infect 2019;95:328–35. 10.1136/sextrans-2018-053823 31055469 PMC6678058

[941] Baumann L, Cina M, Egli-Gany D, Prevalence of *Mycoplasma genitalium* in different population groups: systematic review andmeta-analysis. Sex Transm Infect 2018;94:255–62. 10.1136/sextrans-2017-053384 29440466 PMC5969327

[942] Vandepitte J, Weiss HA, Bukenya J, Association between *Mycoplasma genitalium* infection and HIV acquisition among female sex workers in Uganda: evidence from a nested case-control study. Sex Transm Infect 2014;90:545–9. 10.1136/sextrans-2013-051467 24687129 PMC4215342

[943] Ferré VM, Ekouevi DK, Gbeasor-Komlanvi FA, Prevalence of human papillomavirus, human immunodeficiency virus and other sexually transmitted infections among female sex workers in Togo: a national cross-sectional survey. Clin Microbiol Infect 2019;25:1560.e1–7. 10.1016/j.cmi.2019.04.015 31051265

[944] Mavedzenge SN, Van Der Pol B, Weiss HA, The association between *Mycoplasma genitalium* and HIV-1 acquisition in African women. AIDS 2012;26:617–24. 10.1097/QAD.0b013e32834ff690 22210630

[945] Salado-Rasmussen K, Jensen JS. *Mycoplasma genitalium* testing pattern and macrolide resistance: a Danish nationwide retrospective survey. Clin Infect Dis 2014;59:24–30. 10.1093/cid/ciu217 24729494 PMC4305131

[946] Wold C, Sorthe J, Hartgill U, Olsen AO, Moghaddam A, Reinton N. Identification of macrolide-resistant *Mycoplasma genitalium* using real-time PCR. J Eur Acad Dermatol Venereol 2015;29:1616–20. 10.1111/jdv.12963 25622510

[947] Gesink D, Racey CS, Seah C, *Mycoplasma genitalium* in Toronto, Ont: estimates of prevalence and macrolide resistance. Can Fam Physician 2016;62:e96–101. 27331225 PMC4755653

[948] Kristiansen GQ, Lisby JG, Schønning K. 5’ nuclease genotyping assay for identification of macrolide-resistant *Mycoplasma genitalium* in clinical specimens. J Clin Microbiol 2016;54:1593–7. 10.1128/JCM.00012-16 27053672 PMC4879279

[949] Braam JF, Slotboom B, Van Marm S, High prevalence of the A2058T macrolide resistance-associated mutation in *Mycoplasma genitalium* strains from the Netherlands. J Antimicrob Chemother 2017;72:1529–30. 10.1093/jac/dkw584 28158595

[950] Murray GL, Bradshaw CS, Bissessor M, Increasing macrolide and fluoroquinolone resistance in *Mycoplasma genitalium.* Emerg Infect Dis 2017;23:809–12. 10.3201/eid2305.161745 28418319 PMC5403035

[951] Chernesky MA, Jang D, Martin I, ; Canadian MG Study Group. *Mycoplasma genitalium* antibiotic resistance-mediating mutations in Canadian women with or without *Chlamydia trachomatis* infection. Sex Transm Dis 2017;44:433–5. 10.1097/OLQ.0000000000000617 28608794

[952] Barberá MJ, Fernández-Huerta M, Jensen JS, Caballero E, Andreu A. *Mycoplasma genitalium* macrolide and fluoroquinolone resistance: prevalence and risk factors among a 2013–2014 cohort of patients in Barcelona, Spain. Sex Transm Dis 2017;44:457–62. 10.1097/OLQ.0000000000000631 28703723

[953] Sweeney EL, Trembizki E, Bletchly C, Levels of *Mycoplasma genitalium* antimicrobial resistance differ by both region and gender in the state of Queensland, Australia: implications for treatment guidelines. J Clin Microbiol 2019;57:e01555-18. 10.1128/JCM.01555-18 30602443 PMC6425175

[954] Bissessor M, Tabrizi SN, Twin J, Macrolide resistance and azithromycin failure in a *Mycoplasma genitalium*-infected cohort and response of azithromycin failures to alternative antibiotic regimens. Clin Infect Dis 2015;60:1228–36. 10.1093/cid/ciu1162 25537875

[955] Piñeiro L, Idigoras P, de la Caba I, López-Olaizola M, Cilla G. Guided antibiotic therapy for *Mycoplasma genitalium* infections: analysis of mutations associated with resistance to macrolides and fluoroquinolones [Spanish]. Enferm Infecc Microbiol Clin 2019;37:394–7. 30396750 10.1016/j.eimc.2018.10.003

[956] Dionne-Odom J, Geisler WM, Aaron KJ, High prevalence of multidrug-resistant *Mycoplasma genitalium* in human immunodeficiency virus-infected men who have sex with men in Alabama. Clin Infect Dis 2018;66:796–8. 10.1093/cid/cix853 29028993 PMC5850425

[957] Pitt R, Fifer H, Woodford N, Alexander S. Detection of markers predictive of macrolide and fluoroquinolone resistance in *Mycoplasma genitalium* from patients attending sexual health services in England. Sex Transm Infect 2018;94:9–13. 10.1136/sextrans-2017-053164 28717051

[958] Unemo M, Salado-Rasmussen K, Hansen M, Clinical and analytical evaluation of the new Aptima *Mycoplasma genitalium* assay, with data on *M. genitalium* prevalence and antimicrobial resistance in *M. genitalium* in Denmark, Norway and Sweden in 2016. Clin Microbiol Infect 2018;24:533–9. 10.1016/j.cmi.2017.09.006 28923377

[959] Anderson T, Coughlan E, Werno A. *Mycoplasma genitalium* macrolide and fluoroquinolone resistance detection and clinical implications in a selected cohort in New Zealand. J Clin Microbiol 2017;55:3242–8. 10.1128/JCM.01087-17 28878004 PMC5654908

[960] Shimada Y, Deguchi T, Nakane K, Emergence of clinical strains of *Mycoplasma genitalium* harbouring alterations in ParC associated with fluoroquinolone resistance. Int J Antimicrob Agents 2010;36:255–8. 10.1016/j.ijantimicag.2010.05.011 20580532

[961] Muller EE, Mahlangu MP, Lewis DA, Kularatne RS. Macrolide and fluoroquinolone resistance-associated mutations in *Mycoplasma genitalium* in Johannesburg, South Africa, 2007–2014. BMC Infect Dis 2019;19:148. 10.1186/s12879-019-3797-6 30760230 PMC6373000

[962] Chambers LC, Jensen JS, Morgan JL, Lack of association between the S83I ParC mutation in *Mycoplasma genitalium* and treatment outcomes among men who have sex with men with nongonococcal urethritis. Sex Transm Dis 2019;46:805–9. 10.1097/OLQ.0000000000001035 31259853 PMC7011110

[963] Durukan D, Read TRH, Murray G, Resistance-guided antimicrobial therapy using doxycycline-moxifloxacin and doxycycline-2.5g azithromycin for the treatment of *Mycoplasma genitalium* infection: efficacy and tolerability. Clin Infect Dis 2020;71:1461–8. 10.1093/cid/ciz1031 31629365

[964] Li Y, Le WJ, Li S, Cao YP, Su XH. Meta-analysis of the efficacy of moxifloxacin in treating *Mycoplasma genitalium* infection. Int J STD AIDS 2017;28:1106–14. 10.1177/0956462416688562 28118803

[965] Mondeja BA, Couri J, Rodríguez NM, Blanco O, Fernández C, Jensen JS. Macrolide-resistant *Mycoplasma genitalium* infections in Cuban patients: an underestimated health problem. BMC Infect Dis 2018;18:601. 10.1186/s12879-018-3523-9 30486786 PMC6264040

[966] Glaser AM, Geisler WM, Ratliff AE, Xiao L, Waites KB, Gaisa M. Two cases of multidrug-resistant genitourinary *Mycoplasma genitalium* infection successfully eradicated with minocycline. Int J STD AIDS 2019;30:512–4. 10.1177/0956462418816757 30999836

[967] Xiao L, Waites KB, Van Der Pol B, Aaron KJ, Hook EW 3rd, Geisler WM. *Mycoplasma genitalium* infections with macrolide and fluoroquinolone resistance-associated mutations in heterosexual African American couples in Alabama. Sex Transm Dis 2019;46:18–24. 10.1097/OLQ.0000000000000891 29979336 PMC6289787

[968] Slifirski JB, Vodstrcil LA, Fairley CK, *Mycoplasma genitalium* infection in adults reporting sexual contact with infected partners, Australia, 2008–2016. Emerg Infect Dis 2017;23:1826–33. 10.3201/eid2311.170998 29047422 PMC5652440

[969] Anderson MR, Klink K, Cohrssen A. Evaluation of vaginal complaints. JAMA 2004;291:1368–79. 10.1001/jama.291.11.1368 15026404

[970] Swidsinski A, Mendling W, Loening-Baucke V, Adherent biofilms in bacterial vaginosis. Obstet Gynecol 2005;106:1013–23. 10.1097/01.AOG.0000183594.45524.d2 16260520

[971] Brotman RM, Klebanoff MA, Nansel TR, Bacterial vaginosis assessed by Gram stain and diminished colonization resistance to incident gonococcal, chlamydial, and trichomonal genital infection. J Infect Dis 2010;202:1907–15. 10.1086/657320 21067371 PMC3053135

[972] Peebles K, Velloza J, Balkus JE, McClelland RS, Barnabas RV. High global burden and costs of bacterial vaginosis: a systematic review and meta-analysis. Sex Transm Dis 2019;46:304–11. 10.1097/OLQ.0000000000000972 30624309

[973] Kenyon CR, Buyze J, Klebanoff M, Brotman RM. Association between bacterial vaginosis and partner concurrency: a longitudinal study. Sex Transm Infect 2018;94:75–7. 10.1136/sextrans-2016-052652 27645157 PMC5429208

[974] Sanchez S, Garcia PJ, Thomas KK, Catlin M, Holmes KK. Intravaginal metronidazole gel versus metronidazole plus nystatin ovules for bacterial vaginosis: a randomized controlled trial. Am J Obstet Gynecol 2004;191:1898–906. 10.1016/j.ajog.2004.06.089 15592270

[975] Ness RB, Soper DE, Holley RL, ; PID Evaluation and Clinical Health (PEACH) Study Investigators. Douching and endometritis: results from the PID evaluation and clinical health (PEACH) study. Sex Transm Dis 2001;28:240–5. 10.1097/00007435-200104000-00010 11318257

[976] Gondwe T, Ness R, Totten PA, Novel bacterial vaginosis-associated organisms mediate the relationship between vaginal douching and pelvic inflammatory disease. Sex Transm Infect 2020;96:439–44. 10.1136/sextrans-2019-054191 31810995 PMC7476288

[977] Abbai NS, Reddy T, Ramjee G. Prevalent bacterial vaginosis infection—a risk factor for incident sexually transmitted infections in women in Durban, South Africa. Int J STD AIDS 2016;27:1283–8. 10.1177/0956462415616038 26538552

[978] Morris BJ, Hankins CA, Banerjee J, Does male circumcision reduce women’s risk of sexually transmitted infections, cervical cancer, and associated conditions? Front Public Health 2019;7:4. 10.3389/fpubh.2019.00004 30766863 PMC6365441

[979] Srinivasan S, Liu C, Mitchell CM, Temporal variability of human vaginal bacteria and relationship with bacterial vaginosis. PLoS One 2010;5:e10197. 10.1371/journal.pone.0010197 20419168 PMC2855365

[980] Gajer P, Brotman RM, Bai G, Temporal dynamics of the human vaginal microbiota. Sci Transl Med 2012;4:132ra52. 10.1126/scitranslmed.3003605 22553250 PMC3722878

[981] Fethers KA, Fairley CK, Morton A, Early sexual experiences and risk factors for bacterial vaginosis. J Infect Dis 2009;200:1662–70. 10.1086/648092 19863439

[982] Achilles SL, Austin MN, Meyn LA, Mhlanga F, Chirenje ZM, Hillier SL. Impact of contraceptive initiation on vaginal microbiota. Am J Obstet Gynecol 2018;218:622.e1–10. 10.1016/j.ajog.2018.02.017 29505773 PMC5990849

[983] Vodstrcil LA, Plummer ME, Fairley CK, Combined oral contraceptive pill-exposure alone does not reduce the risk of bacterial vaginosis recurrence in a pilot randomised controlled trial. Sci Rep 2019;9:3555. 10.1038/s41598-019-39879-8 30837554 PMC6401172

[984] Brooks JP, Edwards DJ, Blithe DL, Effects of combined oral contraceptives, depot medroxyprogesterone acetate and the levonorgestrel-releasing intrauterine system on the vaginal microbiome. Contraception 2017;95:405–13. 10.1016/j.contraception.2016.11.006 27913230 PMC5376524

[985] Moore KR, Harmon QE, Baird DD. Serum 25-hydroxyvitamin D and risk of self-reported bacterial vaginosis in a prospective cohort study of young African American women. J Womens Health (Larchmt) 2018;27:1278–84. 10.1089/jwh.2017.6804 29897832 PMC6205036

[986] Lokken EM, Balkus JE, Kiarie J, Association of recent bacterial vaginosis with acquisition of *Mycoplasma genitalium.* Am J Epidemiol 2017;186:194–201. 10.1093/aje/kwx043 28472225 PMC5860020

[987] Brusselaers N, Shrestha S, van de Wijgert J, Verstraelen H. Vaginal dysbiosis and the risk of human papillomavirus and cervical cancer: systematic review and meta-analysis. Am J Obstet Gynecol 2019;221:9–18.e8. 10.1016/j.ajog.2018.12.011 30550767

[988] Abbai NS, Nyirenda M, Naidoo S, Ramjee G. Prevalent herpes simplex virus-2 increases the risk of incident bacterial vaginosis in women from South Africa. AIDS Behav 2018;22:2172–80. 10.1007/s10461-017-1924-1 28956191 PMC5871553

[989] Laxmi U, Agrawal S, Raghunandan C, Randhawa VS, Saili A. Association of bacterial vaginosis with adverse fetomaternal outcome in women with spontaneous preterm labor: a prospective cohort study. J Matern Fetal Neonatal Med 2012;25:64–7. 10.3109/14767058.2011.565390 21557693

[990] Cherpes TL, Wiesenfeld HC, Melan MA, The associations between pelvic inflammatory disease, *Trichomonas vaginalis* infection, and positive herpes simplex virus type 2 serology. Sex Transm Dis 2006;33:747–52. 10.1097/01.olq.0000218869.52753.c7 16691155

[991] Nelson DB, Hanlon A, Hassan S, Preterm labor and bacterial vaginosis-associated bacteria among urban women. J Perinat Med 2009;37:130–4. 10.1515/JPM.2009.026 18999913 PMC3979329

[992] Atashili J, Poole C, Ndumbe PM, Adimora AA, Smith JS. Bacterial vaginosis and HIV acquisition: a meta-analysis of published studies. AIDS 2008;22:1493–501. 10.1097/QAD.0b013e3283021a37 18614873 PMC2788489

[993] Gosmann C, Anahtar MN, Handley SA, Lactobacillus-deficient cervicovaginal bacterial communities are associated with increased HIV acquisition in young South African women. Immunity 2017;46:29–37. 10.1016/j.immuni.2016.12.013 28087240 PMC5270628

[994] McClelland RS, Lingappa JR, Srinivasan S, Evaluation of the association between the concentrations of key vaginal bacteria and the increased risk of HIV acquisition in African women from five cohorts: a nested case-control study. Lancet Infect Dis 2018;18:554–64. 10.1016/S1473-3099(18)30058-6 29396006 PMC6445552

[995] Johnston C, Magaret A, Srinivasan S, P239 Genital HSV-2 suppression is not associated with alterations in the vaginal microbiome: a one-way, cross-over study. Sex Transm Infect 2019;95(Suppl 1):A148.

[996] Zozaya M, Ferris MJ, Siren JD, Bacterial communities in penile skin, male urethra, and vaginas of heterosexual couples with and without bacterial vaginosis. Microbiome 2016;4:16. 10.1186/s40168-016-0161-6 27090518 PMC4835890

[997] Liu CM, Hungate BA, Tobian AA, Penile microbiota and female partner bacterial vaginosis in Rakai, Uganda. MBio 2015;6:e00589. 10.1128/mBio.00589-15 26081632 PMC4471566

[998] Mehta SD. Systematic review of randomized trials of treatment of male sexual partners for improved bacteria vaginosis outcomes in women. Sex Transm Dis 2012;39:822–30. 10.1097/OLQ.0b013e3182631d89 23007709

[999] Amsel R, Totten PA, Spiegel CA, Chen KC, Eschenbach D, Holmes KK. Nonspecific vaginitis. Diagnostic criteria and microbial and epidemiologic associations. Am J Med 1983;74:14–22. 10.1016/0002-9343(83)91112-9 6600371

[1000] Nugent RP, Krohn MA, Hillier SL. Reliability of diagnosing bacterial vaginosis is improved by a standardized method of Gram stain interpretation. J Clin Microbiol 1991;29:297–301. 10.1128/JCM.29.2.297-301.1991 1706728 PMC269757

[1001] Schwebke JR, Hillier SL, Sobel JD, McGregor JA, Sweet RL. Validity of the vaginal Gram stain for the diagnosis of bacterial vaginosis. Obstet Gynecol 1996;88:573–6. 10.1016/0029-7844(96)00233-5 8841221

[1002] Coleman JS, Gaydos CA. Molecular diagnosis of bacterial vaginosis: an update. J Clin Microbiol 2018;56:e00342-18. 10.1128/JCM.00342-18 29769280 PMC6113459

[1003] Myziuk L, Romanowski B, Johnson SC. BVBlue test for diagnosis of bacterial vaginosis. J Clin Microbiol 2003;41:1925–8. 10.1128/JCM.41.5.1925-1928.2003 12734228 PMC154737

[1004] Bradshaw CS, Morton AN, Garland SM, Horvath LB, Kuzevska I, Fairley CK. Evaluation of a point-of-care test, BVBlue, and clinical and laboratory criteria for diagnosis of bacterial vaginosis. J Clin Microbiol 2005;43:1304–8. 10.1128/JCM.43.3.1304-1308.2005 15750100 PMC1081297

[1005] West B, Morison L, Schim van der Loeff M, Evaluation of a new rapid diagnostic kit (FemExam) for bacterial vaginosis in patients with vaginal discharge syndrome in The Gambia. Sex Transm Dis 2003;30:483–9. 10.1097/00007435-200306000-00003 12782948

[1006] Fredricks DN, Fiedler TL, Thomas KK, Oakley BB, Marrazzo JM. Targeted PCR for detection of vaginal bacteria associated with bacterial vaginosis. J Clin Microbiol 2007;45:3270–6. 10.1128/JCM.01272-07 17687006 PMC2045326

[1007] Gaydos CA, Beqaj S, Schwebke JR, Clinical validation of a test for the diagnosis of vaginitis. Obstet Gynecol 2017;130:181–9. 10.1097/AOG.0000000000002090 28594779 PMC5635603

[1008] Cartwright CP, Lembke BD, Ramachandran K, Development and validation of a semiquantitative, multitarget PCR assay for diagnosis of bacterial vaginosis. J Clin Microbiol 2012;50:2321–9. 10.1128/JCM.00506-12 22535982 PMC3405607

[1009] Hilbert DW, Smith WL, Chadwick SG, Development and validation of a highly accurate quantitative real-time PCR assay for diagnosis of bacterial vaginosis. J Clin Microbiol 2016;54:1017–24. Erratum in: J Clin Microbiol 2016;54:1930. 10.1128/JCM.03104-15 26818677 PMC4809904

[1010] Schwebke JR, Desmond R. A randomized trial of metronidazole in asymptomatic bacterial vaginosis to prevent the acquisition of sexually transmitted diseases. Am J Obstet Gynecol 2007;196:517.e1–6. 10.1016/j.ajog.2007.02.048 17547876 PMC1993882

[1011] Fjeld H, Raknes G. Is combining metronidazole and alcohol really hazardous? [Norwegian]. Tidsskr Nor Laegeforen 2014;134:1661–3. 10.4045/tidsskr.14.0081 25223673

[1012] Hillier SL, Nyirjesy P, Waldbaum AS, Secnidazole treatment of bacterial vaginosis: a randomized controlled trial. Obstet Gynecol 2017;130:379–86. 10.1097/AOG.0000000000002135 28697102

[1013] Schwebke JR, Morgan FG Jr, Koltun W, Nyirjesy P. A phase-3, double-blind, placebo-controlled study of the effectiveness and safety of single oral doses of secnidazole 2 g for the treatment of women with bacterial vaginosis. Am J Obstet Gynecol 2017;217:678.e1–9. Erratum in: Am J Obstet Gynecol 2018;219;110. 10.1016/j.ajog.2017.08.017 28867602

[1014] Chavoustie SE, Gersten JK, Samuel MJ, Schwebke JR. A phase 3, multicenter, prospective, open-label study to evaluate the safety of a single dose of secnidazole 2 g for the treatment of women and postmenarchal adolescent girls with bacterial vaginosis. J Womens Health (Larchmt) 2018;27:492–7. 10.1089/jwh.2017.6500 29323627

[1015] Livengood CH 3rd, Ferris DG, Wiesenfeld HC, Effectiveness of two tinidazole regimens in treatment of bacterial vaginosis: a randomized controlled trial. Obstet Gynecol 2007;110:302–9. 10.1097/01.AOG.0000275282.60506.3d 17666604

[1016] Sobel JD, Nyirjesy P, Brown W. Tinidazole therapy for metronidazole-resistant vaginal trichomoniasis. Clin Infect Dis 2001;33:1341–6. 10.1086/323034 11565074

[1017] Chavoustie SE, Jacobs M, Reisman HA, Metronidazole vaginal gel 1.3% in the treatment of bacterial vaginosis: a dose-ranging study. J Low Genit Tract Dis 2015;19:129–34. 10.1097/LGT.0000000000000062 24983350 PMC4376277

[1018] Schwebke JR, Marrazzo J, Beelen AP, Sobel JD. A phase 3, multicenter, randomized, double-blind, vehicle-controlled study evaluating the safety and efficacy of metronidazole vaginal gel 1.3% in the treatment of bacterial vaginosis. Sex Transm Dis 2015;42:376–81. 10.1097/OLQ.0000000000000300 26222750 PMC4463027

[1019] Faro S, Skokos CK; Clindesse Investigators Group. The efficacy and safety of a single dose of Clindesse vaginal cream versus a seven-dose regimen of Cleocin vaginal cream in patients with bacterial vaginosis. Infect Dis Obstet Gynecol 2005;13:155–60. 10.1080/10647440500148321 16240515 PMC1784567

[1020] Marrazzo JM, Dombrowski JC, Wierzbicki MR, Safety and efficacy of a novel vaginal anti-infective, TOL-463, in the treatment of bacterial vaginosis and vulvovaginal candidiasis: a randomized, single-blind, phase 2, controlled trial. Clin Infect Dis 2019;68:803–9. 10.1093/cid/ciy554 30184181 PMC6376090

[1021] Antonio MA, Meyn LA, Murray PJ, Busse B, Hillier SL. Vaginal colonization by probiotic *Lactobacillus crispatus* CTV-05 is decreased by sexual activity and endogenous *Lactobacilli.* J Infect Dis 2009;199:1506–13. 10.1086/598686 19331578

[1022] Senok AC, Verstraelen H, Temmerman M, Botta GA. Probiotics for the treatment of bacterial vaginosis. Cochrane Database Syst Rev 2009;(4):CD006289. 10.1002/14651858.CD006289.pub2 19821358

[1023] Abad CL, Safdar N. The role of *Lactobacillus* probiotics in the treatment or prevention of urogenital infections—a systematic review. J Chemother 2009;21:243–52. 10.1179/joc.2009.21.3.243 19567343

[1024] Mastromarino P, Macchia S, Meggiorini L, Effectiveness of *Lactobacillus*-containing vaginal tablets in the treatment of symptomatic bacterial vaginosis. Clin Microbiol Infect 2009;15:67–74. 10.1111/j.1469-0691.2008.02112.x 19046169

[1025] Hemmerling A, Harrison W, Schroeder A, Phase 2a study assessing colonization efficiency, safety, and acceptability of *Lactobacillus crispatus* CTV-05 in women with bacterial vaginosis. Sex Transm Dis 2010;37:745–50. 10.1097/OLQ.0b013e3181e50026 20644497

[1026] Bunge KE, Beigi RH, Meyn LA, Hillier SL. The efficacy of retreatment with the same medication for early treatment failure of bacterial vaginosis. Sex Transm Dis 2009;36:711–3. 10.1097/OLQ.0b013e3181af6cfd 19652628

[1027] Aguin T, Akins RA, Sobel JD. High-dose vaginal maintenance metronidazole for recurrent bacterial vaginosis: a pilot study. Sex Transm Dis 2014;41:290–1. 10.1097/OLQ.0000000000000123 24722380

[1028] Sobel JD, Ferris D, Schwebke J, Suppressive antibacterial therapy with 0.75% metronidazole vaginal gel to prevent recurrent bacterial vaginosis. Am J Obstet Gynecol 2006;194:1283–9. 10.1016/j.ajog.2005.11.041 16647911

[1029] Reichman O, Akins R, Sobel JD. Boric acid addition to suppressive antimicrobial therapy for recurrent bacterial vaginosis. Sex Transm Dis 2009;36:732–4. 10.1097/OLQ.0b013e3181b08456 19704395

[1030] McClelland RS, Richardson BA, Hassan WM, Improvement of vaginal health for Kenyan women at risk for acquisition of human immunodeficiency virus type 1: results of a randomized trial. J Infect Dis 2008;197:1361–8. 10.1086/587490 18444793 PMC4122228

[1031] Schwebke J, Carter B, Waldbaum A, Results of a phase 3, randomized, double-blind, placebo-controlled study to evaluate the efficacy and safety of astodrimer gel for prevention of recurrent bacterial vaginosis. Am J Obstet Gynecol 2019;221:672–3. 10.1016/j.ajog.2019.10.087

[1032] Cohen CR, Wierzbicki MR, French AL, Randomized trial of Lactin-V to prevent recurrence of bacterial vaginosis. N Engl J Med 2020;382:1906–15. 10.1056/NEJMoa1915254 32402161 PMC7362958

[1033] Turner AN, Carr Reese P, Fields KS, A blinded, randomized controlled trial of high-dose vitamin D supplementation to reduce recurrence of bacterial vaginosis. Am J Obstet Gynecol 2014;211:479.e1–13. 10.1016/j.ajog.2014.06.023 24949544 PMC4254061

[1034] Plummer EL, Vodstrcil LA, Danielewski JA, Combined oral and topical antimicrobial therapy for male partners of women with bacterial vaginosis: acceptability, tolerability and impact on the genital microbiota of couples—a pilot study. PLoS One 2018;13:e0190199. 10.1371/journal.pone.0190199 29293559 PMC5749747

[1035] Schwebke JR, Lensing SY, Lee J, Treatment of male sexual partners of women with bacterial vaginosis (BV): a randomized, double-blind, placebo-controlled trial. Clin Infect Dis 2020; Epub December 31, 2020. 33383580 10.1093/cid/ciaa1903 PMC8326574

[1036] Koumans EH, Kendrick JS; CDC Bacterial Vaginosis Working Group. Preventing adverse sequelae of bacterial vaginosis: a public health program and research agenda. Sex Transm Dis 2001;28:292–7. 10.1097/00007435-200105000-00011 11354269

[1037] Hauth JC, Goldenberg RL, Andrews WW, DuBard MB, Copper RL. Reduced incidence of preterm delivery with metronidazole and erythromycin in women with bacterial vaginosis. N Engl J Med 1995;333:1732–6. 10.1056/NEJM199512283332603 7491136

[1038] Morales WJ, Schorr S, Albritton J. Effect of metronidazole in patients with preterm birth in preceding pregnancy and bacterial vaginosis: a placebo-controlled, double-blind study. Am J Obstet Gynecol 1994;171:345–9. 10.1016/S0002-9378(94)70033-8 8059811

[1039] Yudin MH, Landers DV, Meyn L, Hillier SL. Clinical and cervical cytokine response to treatment with oral or vaginal metronidazole for bacterial vaginosis during pregnancy: a randomized trial. Obstet Gynecol 2003;102:527–34. 12962937 10.1016/s0029-7844(03)00566-0

[1040] Ugwumadu A, Reid F, Hay P, Manyonda I. Natural history of bacterial vaginosis and intermediate flora in pregnancy and effect of oral clindamycin. Obstet Gynecol 2004;104:114–9. 10.1097/01.AOG.0000130068.21566.4e 15229009

[1041] Burtin P, Taddio A, Ariburnu O, Einarson TR, Koren G. Safety of metronidazole in pregnancy: a meta-analysis. Am J Obstet Gynecol 1995;172:525–9. 10.1016/0002-9378(95)90567-7 7856680

[1042] Piper JM, Mitchel EF, Ray WA. Prenatal use of metronidazole and birth defects: no association. Obstet Gynecol 1993;82:348–52. 8355932

[1043] Sheehy O, Santos F, Ferreira E, Berard A. The use of metronidazole during pregnancy: a review of evidence. Curr Drug Saf 2015;10:170–9. 10.2174/157488631002150515124548 25986038

[1044] Lamont RF, Nhan-Chang CL, Sobel JD, Workowski K, Conde-Agudelo A, Romero R. Treatment of abnormal vaginal flora in early pregnancy with clindamycin for the prevention of spontaneous preterm birth: a systematic review and metaanalysis. Am J Obstet Gynecol 2011;205:177–90. 10.1016/j.ajog.2011.03.047 22071048 PMC3217181

[1045] Odendaal HJ, Popov I, Schoeman J, Smith M, Grové D. Preterm labour—is bacterial vaginosis involved? S Afr Med J 2002;92:231–4. 12040953

[1046] Carey JC, Klebanoff MA, Hauth JC, ; National Institute of Child Health and Human Development Network of Maternal-Fetal Medicine Units. Metronidazole to prevent preterm delivery in pregnant women with asymptomatic bacterial vaginosis. N Engl J Med 2000;342:534–40. 10.1056/NEJM200002243420802 10684911

[1047] Vermeulen GM, Bruinse HW. Prophylactic administration of clindamycin 2% vaginal cream to reduce the incidence of spontaneous preterm birth in women with an increased recurrence risk: a randomised placebo-controlled double-blind trial. Br J Obstet Gynaecol 1999;106:652–7. 10.1111/j.1471-0528.1999.tb08363.x 10428520

[1048] McDonald HM, O’Loughlin JA, Vigneswaran R, Impact of metronidazole therapy on preterm birth in women with bacterial vaginosis flora (*Gardnerella vaginalis*): a randomised, placebo controlled trial. Br J Obstet Gynaecol 1997;104:1391–7. 10.1111/j.1471-0528.1997.tb11009.x 9422018

[1049] Ugwumadu A, Manyonda I, Reid F, Hay P. Effect of early oral clindamycin on late miscarriage and preterm delivery in asymptomatic women with abnormal vaginal flora and bacterial vaginosis: a randomised controlled trial. Lancet 2003;361:983–8. 10.1016/S0140-6736(03)12823-1 12660054

[1050] Subtil D, Brabant G, Tilloy E, Early clindamycin for bacterial vaginosis in pregnancy (PREMEVA): a multicentre, double-blind, randomised controlled trial. Lancet 2018;392:2171–9. 10.1016/S0140-6736(18)31617-9 30322724

[1051] Erickson SH, Oppenheim GL, Smith GH. Metronidazole in breast milk. Obstet Gynecol 1981;57:48–50. 7454176

[1052] Passmore CM, McElnay JC, Rainey EA, D’Arcy PF. Metronidazole excretion in human milk and its effect on the suckling neonate. Br J Clin Pharmacol 1988;26:45–51. 10.1111/j.1365-2125.1988.tb03362.x 3203060 PMC1386498

[1053] United Kingdom National Health Service. Medicines Q&A: metronidazole—is it safe to use with breastfeeding? [Internet]. London, England: United Kingdom National Health Service, UK Medicines Information; 2012. https://studylib.net/doc/7341270/metronidazole-in-breastfeeding-mothers

[1054] Jamieson DJ, Duerr A, Klein RS, Longitudinal analysis of bacterial vaginosis: findings from the HIV epidemiology research study. Obstet Gynecol 2001;98:656–63. 10.1097/00006250-200110000-00023 11576584

[1055] Rowley J, Vander Hoorn S, Korenromp E, Chlamydia, gonorrhoea, trichomoniasis and syphilis: global prevalence and incidence estimates, 2016. Bull World Health Organ 2019;97:548–562P. 10.2471/BLT.18.228486 31384073 PMC6653813

[1056] Hoots BE, Peterman TA, Torrone EA, Weinstock H, Meites E, Bolan GA. A Trich-y question: should *Trichomonas vaginalis* infection be reportable? Sex Transm Dis 2013;40:113–6. 10.1097/OLQ.0b013e31827c08c3 23321992 PMC5024551

[1057] Flagg EW, Meites E, Phillips C, Papp J, Torrone EA. Prevalence of *Trichomonas vaginalis* among civilian, noninstitutionalized male and female population aged 14 to 59 years: United States, 2013 to 2016. Sex Transm Dis 2019;46:e93–6. 10.1097/OLQ.0000000000001013 31517807 PMC6924265

[1058] Daugherty M, Glynn K, Byler T. Prevalence of *Trichomonas vaginalis* infection among US males, 2013–2016. Clin Infect Dis 2019;68:460–5. 10.1093/cid/ciy499 29893808

[1059] Alcaide ML, Feaster DJ, Duan R, The incidence of *Trichomonas vaginalis* infection in women attending nine sexually transmitted diseases clinics in the USA. Sex Transm Infect 2016;92:58–62. 10.1136/sextrans-2015-052010 26071390 PMC4874593

[1060] Muzny CA, Blackburn RJ, Sinsky RJ, Austin EL, Schwebke JR. Added benefit of nucleic acid amplification testing for the diagnosis of *Trichomonas vaginalis* among men and women attending a sexually transmitted diseases clinic. Clin Infect Dis 2014;59:834–41. 10.1093/cid/ciu446 24928292

[1061] Meites E, Llata E, Braxton J, *Trichomonas vaginalis* in selected U.S. sexually transmitted disease clinics: testing, screening, and prevalence. Sex Transm Dis 2013;40:865–9. 10.1097/OLQ.0000000000000038 24113409 PMC4677780

[1062] Ginocchio CC, Chapin K, Smith JS, Prevalence of *Trichomonas vaginalis* and coinfection with *Chlamydia trachomatis* and *Neisseria gonorrhoeae* in the United States as determined by the Aptima *Trichomonas vaginalis* nucleic acid amplification assay. J Clin Microbiol 2012;50:2601–8. 10.1128/JCM.00748-12 22622447 PMC3421522

[1063] Shuter J, Bell D, Graham D, Holbrook KA, Bellin EY. Rates of and risk factors for trichomoniasis among pregnant inmates in New York City. Sex Transm Dis 1998;25:303–7. 10.1097/00007435-199807000-00006 9662764

[1064] Sosman JM, MacGowan RJ, Margolis AD, ; Project START Study Group. Screening for sexually transmitted diseases and hepatitis in 18–29-year-old men recently released from prison: feasibility and acceptability. Int J STD AIDS 2005;16:117–22. 10.1258/0956462053057594 15825246

[1065] Rogers SM, Turner CF, Hobbs M, Epidemiology of undiagnosed trichomoniasis in a probability sample of urban young adults. PLoS One 2014;9:e90548. 10.1371/journal.pone.0090548 24626058 PMC3953116

[1066] Mayer KH, Bush T, Henry K, ; SUN Investigators. Ongoing sexually transmitted disease acquisition and risk-taking behavior among US HIV-infected patients in primary care: implications for prevention interventions. Sex Transm Dis 2012;39:1–7. 10.1097/OLQ.0b013e31823b1922 22183836 PMC3740591

[1067] Seña AC, Miller WC, Hobbs MM, *Trichomonas vaginalis* infection in male sexual partners: implications for diagnosis, treatment, and prevention. Clin Infect Dis 2007;44:13–22. 10.1086/511144 17143809

[1068] Kelley CF, Rosenberg ES, OʼHara BM, Sanchez T, del Rio C, Sullivan PS. Prevalence of urethral *Trichomonas vaginalis* in black and white men who have sex with men. Sex Transm Dis 2012;39:739. 10.1097/OLQ.0b013e318264248b 22902674 PMC3665349

[1069] Sutton M, Sternberg M, Koumans EH, McQuillan G, Berman S, Markowitz L. The prevalence of *Trichomonas vaginalis* infection among reproductive-age women in the United States, 2001–2004. Clin Infect Dis 2007;45:1319–26. 10.1086/522532 17968828

[1070] Peterman TA, Tian LH, Metcalf CA, Malotte CK, Paul SM, Douglas JM Jr; RESPECT-2 Study Group. Persistent, undetected *Trichomonas vaginalis* infections? Clin Infect Dis 2009;48:259–60. 10.1086/595706 19113985

[1071] Wølner-Hanssen P, Krieger JN, Stevens CE, Clinical manifestations of vaginal trichomoniasis. JAMA 1989;261:571–6. 10.1001/jama.1989.03420040109029 2783346

[1072] Gray RH, Kigozi G, Serwadda D, The effects of male circumcision on female partners’ genital tract symptoms and vaginal infections in a randomized trial in Rakai, Uganda. Am J Obstet Gynecol 2009;200:42.e1–7. 10.1016/j.ajog.2008.07.069 18976733 PMC2727852

[1073] Sobngwi-Tambekou J, Taljaard D, Nieuwoudt M, Lissouba P, Puren A, Auvert B. Male circumcision and *Neisseria gonorrhoeae*, *Chlamydia trachomatis* and *Trichomonas vaginalis*: observations after a randomised controlled trial for HIV prevention. Sex Transm Infect 2009;85:116–20. 10.1136/sti.2008.032334 19074928 PMC2652030

[1074] Tsai CS, Shepherd BE, Vermund SH. Does douching increase risk for sexually transmitted infections? A prospective study in high-risk adolescents. Am J Obstet Gynecol 2009;200:38.e1–8. 10.1016/j.ajog.2008.06.026 18667177 PMC3199592

[1075] Silver BJ, Guy RJ, Kaldor JM, Jamil MS, Rumbold AR. *Trichomonas vaginalis* as a cause of perinatal morbidity: a systematic review and meta-analysis. Sex Transm Dis 2014;41:369–76. 10.1097/OLQ.0000000000000134 24825333

[1076] Yang S, Zhao W, Wang H, Wang Y, Li J, Wu X. *Trichomonas vaginalis* infection-associated risk of cervical cancer: a meta-analysis. Eur J Obstet Gynecol Reprod Biol 2018;228:166–73. 10.1016/j.ejogrb.2018.06.031 29980111

[1077] Najafi A, Chaechi Nosrati MR, Ghasemi E, Is there association between *Trichomonas vaginalis* infection and prostate cancer risk?: A systematic review and meta-analysis. Microb Pathog 2019;137:103752. 10.1016/j.micpath.2019.103752 31539586

[1078] Wang CC, McClelland RS, Reilly M, The effect of treatment of vaginal infections on shedding of human immunodeficiency virus type 1. J Infect Dis 2001;183:1017–22. 10.1086/319287 11237825

[1079] Kissinger P, Amedee A, Clark RA, *Trichomonas vaginalis* treatment reduces vaginal HIV-1 shedding. Sex Transm Dis 2009;36:11–6. 10.1097/OLQ.0b013e318186decf 19008776 PMC3779369

[1080] Minkoff H, Grunebaum AN, Schwarz RH, Risk factors for prematurity and premature rupture of membranes: a prospective study of the vaginal flora in pregnancy. Am J Obstet Gynecol 1984;150:965–72. 10.1016/0002-9378(84)90392-2 6391179

[1081] Cotch MF, Pastorek JG 2nd, Nugent RP, ; The Vaginal Infections and Prematurity Study Group. *Trichomonas vaginalis* associated with low birth weight and preterm delivery. Sex Transm Dis 1997;24:353–60. 10.1097/00007435-199707000-00008 9243743

[1082] Moodley P, Wilkinson D, Connolly C, Moodley J, Sturm AW. *Trichomonas vaginalis* is associated with pelvic inflammatory disease in women infected with human immunodeficiency virus. Clin Infect Dis 2002;34:519–22. 10.1086/338399 11797180

[1083] Francis SC, Kent CK, Klausner JD, Prevalence of rectal *Trichomonas vaginalis* and *Mycoplasma genitalium* in male patients at the San Francisco STD clinic, 2005–2006. Sex Transm Dis 2008;35:797–800. 10.1097/OLQ.0b013e318177ec39 18607317 PMC3776945

[1084] Hollman D, Coupey SM, Fox AS, Herold BC. Screening for *Trichomonas vaginalis* in high-risk adolescent females with a new transcription-mediated nucleic acid amplification test (NAAT): associations with ethnicity, symptoms, and prior and current STIs. J Pediatr Adolesc Gynecol 2010;23:312–6. 10.1016/j.jpag.2010.03.004 20493735

[1085] Roth AM, Williams JA, Ly R, Changing sexually transmitted infection screening protocol will result in improved case finding for *Trichomonas vaginalis* among high-risk female populations. Sex Transm Dis 2011;38:398–400. 10.1097/OLQ.0b013e318203e3ce 21217417

[1086] Hobbs MM, Seña AC. Modern diagnosis of *Trichomonas vaginalis* infection. Sex Transm Infect 2013;89:434–8. 10.1136/sextrans-2013-051057 23633669 PMC3787709

[1087] Kingston MA, Bansal D, Carlin EM. ‘Shelf life’ of *Trichomonas vaginalis.* Int J STD AIDS 2003;14:28–9. 10.1258/095646203321043228 12590789

[1088] Schwebke JR, Hobbs MM, Taylor SN, Molecular testing for *Trichomonas vaginalis* in women: results from a prospective U.S. clinical trial. J Clin Microbiol 2011;49:4106–11. 10.1128/JCM.01291-11 21940475 PMC3232944

[1089] Huppert JS, Mortensen JE, Reed JL, Rapid antigen testing compares favorably with transcription-mediated amplification assay for the detection of *Trichomonas vaginalis* in young women. Clin Infect Dis 2007;45:194–8. 10.1086/518851 17578778

[1090] Van Der Pol B, Williams JA, Taylor SN, Detection of *Trichomonas vaginalis* DNA by use of self-obtained vaginal swabs with the BD ProbeTec Qx assay on the BD Viper system. J Clin Microbiol 2014;52:885–9. 10.1128/JCM.02966-13 24391200 PMC3957762

[1091] Van Der Pol B, Williams JA, Fuller D, Taylor SN, Hook EW 3rd. Combined testing for chlamydia, gonorrhea, and trichomonas by use of the BD Max CT/GC/TV assay with genitourinary specimen types. J Clin Microbiol 2016;55:155–64. 10.1128/JCM.01766-16 27795343 PMC5228226

[1092] Schwebke JR, Gaydos CA, Davis T, Clinical evaluation of the Cepheid Xpert TV Assay for detection of *Trichomonas vaginalis* with prospectively collected specimens from men and women. J Clin Microbiol 2018;56:e01091-17. 10.1128/JCM.01091-17 29167292 PMC5786720

[1093] Campbell L, Woods V, Lloyd T, Elsayed S, Church DL. Evaluation of the OSOM Trichomonas rapid test versus wet preparation examination for detection of *Trichomonas vaginalis* vaginitis in specimens from women with a low prevalence of infection. J Clin Microbiol 2008;46:3467–9. 10.1128/JCM.00671-08 18685008 PMC2566104

[1094] Huppert JS, Hesse E, Kim G, Adolescent women can perform a point-of-care test for trichomoniasis as accurately as clinicians. Sex Transm Infect 2010;86:514–9. 10.1136/sti.2009.042168 20595142 PMC3221308

[1095] Sheele JM, Crandall CJ, Arko BL, The OSOM® Trichomonas Test is unable to accurately diagnose *Trichomonas vaginalis* from urine in men. Am J Emerg Med 2019;37:1002–3. 10.1016/j.ajem.2018.10.022 30361151

[1096] Gaydos CA, Schwebke J, Dombrowski J, Clinical performance of the Solana® Point-of-Care Trichomonas Assay from clinician-collected vaginal swabs and urine specimens from symptomatic and asymptomatic women. Expert Rev Mol Diagn 2017;17:303–6. 10.1080/14737159.2017.1282823 28092466 PMC5615814

[1097] Gaydos CA, Hobbs M, Marrazzo J, Rapid diagnosis of *Trichomonas vaginalis* by testing vaginal swabs in an isothermal helicase-dependent AmpliVue Assay. Sex Transm Dis 2016;43:369–73. 10.1097/OLQ.0000000000000447 27196258 PMC4874652

[1098] Patil MJ, Nagamoti JM, Metgud SC. Diagnosis of *Trichomonas vaginalis* from vaginal specimens by wet mount microscopy, In Pouch TV culture system, and PCR. J Glob Infect Dis 2012;4:22–5. 10.4103/0974-777X.93756 22529623 PMC3326953

[1099] Lawing LF, Hedges SR, Schwebke JR. Detection of trichomonosis in vaginal and urine specimens from women by culture and PCR. J Clin Microbiol 2000;38:3585–8. 10.1128/JCM.38.10.3585-3588.2000 11015368 PMC87441

[1100] Mohamed OA, Cohen CR, Kungu D, Urine proves a poor specimen for culture of *Trichomonas vaginalis* in women. Sex Transm Infect 2001;77:78–9. 10.1136/sti.77.1.78 11158705 PMC1758328

[1101] Rivers CA, Muzny CA, Schwebke JR. Diagnostic rates differ on the basis of the number of read days with the use of the InPouch culture system for *Trichomonas vaginalis* screening. J Clin Microbiol 2013;51:3875–6. 10.1128/JCM.02006-13 24006006 PMC3889787

[1103] Audisio T, Pigini T, de Riutort SV, Validity of the Papanicolaou smear in the diagnosis of *Candida* spp., *Trichomonas vaginalis*, and bacterial vaginosis. J Low Genit Tract Dis 2001;5:223–5. 17050980

[1104] Loo SK, Tang WY, Lo KK. Clinical significance of *Trichomonas vaginalis* detected in Papanicolaou smear: a survey in female Social Hygiene Clinic. Hong Kong Med J 2009;15:90–3. 19342733

[1105] Howe K, Kissinger PJ. Single-dose compared with multidose metronidazole for the treatment of trichomoniasis in women: a meta-analysis. Sex Transm Dis 2017;44:29–34. 10.1097/OLQ.0000000000000537 27898571 PMC5145758

[1105] Kissinger P, Mena L, Levison J, A randomized treatment trial: single versus 7-day dose of metronidazole for the treatment of *Trichomonas vaginalis* among HIV-infected women. J Acquir Immune Defic Syndr 2010;55:565–71. 10.1097/QAI.0b013e3181eda955 21423852 PMC3058179

[1106] Wood BA, Monro AM. Pharmacokinetics of tinidazole and metronidazole in women after single large oral doses. Br J Vener Dis 1975;51:51–3. 10.1136/sti.51.1.51 1092424 PMC1045111

[1107] Viitanen J, Haataja H, Männistö PT. Concentrations of metronidazole and tinidazole in male genital tissues. Antimicrob Agents Chemother 1985;28:812–4. 10.1128/AAC.28.6.812 4083864 PMC180334

[1108] Gabriel G, Robertson E, Thin RN. Single dose treatment of trichomoniasis. J Int Med Res 1982;10:129–30. 10.1177/030006058201000212 7067925

[1109] Mati JK, Wallace RJ. The treatment of trichomonal vaginitis using a single dose of tinidazole by mouth. East Afr Med J 1974;51:883–8. 4616829

[1110] Anjaeyulu R, Gupte SA, Desai DB. Single-dose treatment of trichomonal vaginitis: a comparison of tinidazole and metronidazole. J Int Med Res 1977;5:438–41. 590601 10.1177/030006057300100210

[1111] Apte VV, Packard RS. Tinidazole in the treatment of trichomoniasis, giardiasis and amoebiasis. Report of a multicentre study. Drugs 1978;15(Suppl 1):43–8. 10.2165/00003495-197800151-00009 657995

[1112] O-Prasertsawat P, Jetsawangsri T. Split-dose metronidazole or single-dose tinidazole for the treatment of vaginal trichomoniasis. Sex Transm Dis 1992;19:295–7. 10.1097/00007435-199209000-00011 1411848

[1113] Kawamura N. Metronidazole and tinidazole in a single large dose for treating urogenital infections with *Trichomonas vaginalis* in men. Br J Vener Dis 1978;54:81–3. 10.1136/sti.54.2.81 305809 PMC1046366

[1114] Forna F, Gülmezoglu AM. Interventions for treating trichomoniasis in women. Cochrane Database Syst Rev 2003;(2):CD000218. 10796512 10.1002/14651858.CD000218 PMC6532670

[1115] Cu-Uvin S, Ko H, Jamieson DJ, ; HIV Epidemiology Research Study (HERS) Group. Prevalence, incidence, and persistence or recurrence of trichomoniasis among human immunodeficiency virus (HIV)-positive women and among HIV-negative women at high risk for HIV infection. Clin Infect Dis 2002;34:1406–11. 10.1086/340264 11981738

[1116] Schwebke JR, Barrientes FJ. Prevalence of *Trichomonas vaginalis* isolates with resistance to metronidazole and tinidazole. Antimicrob Agents Chemother 2006;50:4209–10. 10.1128/AAC.00814-06 17000740 PMC1693974

[1117] Van Der Pol B, Williams JA, Orr DP, Batteiger BE, Fortenberry JD. Prevalence, incidence, natural history, and response to treatment of *Trichomonas vaginalis* infection among adolescent women. J Infect Dis 2005;192:2039–44. 10.1086/498217 16288365

[1118] Kirkcaldy RD, Augostini P, Asbel LE, *Trichomonas vaginalis* antimicrobial drug resistance in 6 US cities, STD Surveillance Network, 2009–2010. Emerg Infect Dis 2012;18:939–43. 10.3201/eid1806.111590 22608054 PMC3358158

[1119] Crowell AL, Sanders-Lewis KA, Secor WE. In vitro metronidazole and tinidazole activities against metronidazole-resistant strains of *Trichomonas vaginalis.* Antimicrob Agents Chemother 2003;47:1407–9. 10.1128/AAC.47.4.1407-1409.2003 12654679 PMC152533

[1120] Muzny CA, Mena L, Lillis RA, A comparison of 2 g single-dose versus 7-day 500 mg twice daily metronidazole for the treatment trichomoniasis in women by selected clinical factors. Am J Obstet Gynecol 2019;221:669. 10.1016/j.ajog.2019.10.079

[1121] Sobel JD, Nyirjesy P, Brown W. Tinidazole therapy for metronidazole-resistant vaginal trichomoniasis. Clin Infect Dis 2001;33:1341–6. 10.1086/323034 11565074

[1122] Nyirjesy P, Gilbert J, Mulcahy LJ. Resistant trichomoniasis: successful treatment with combination therapy. Sex Transm Dis 2011;38:962–3. 10.1097/OLQ.0b013e31822037e4 21934573

[1123] Muzny C, Barnes A, Mena L. Symptomatic *Trichomonas vaginalis* infection in the setting of severe nitroimidazole allergy: successful treatment with boric acid. Sex Health 2012;9:389–91. 10.1071/SH11114 22877600

[1124] Aggarwal A, Shier RM. Recalcitrant *Trichomonas vaginalis* infections successfully treated with vaginal acidification. J Obstet Gynaecol Can 2008;30:55–8. 10.1016/S1701-2163(16)32714-1 18198069

[1125] Dan M, Sobel JD. Failure of nitazoxanide to cure trichomoniasis in three women. Sex Transm Dis 2007;34:813–4. 10.1097/NMD.0b013e31802f5d9a 17551415

[1126] Seña AC, Bachmann LH, Hobbs MM. Persistent and recurrent *Trichomonas vaginalis* infections: epidemiology, treatment and management considerations. Expert Rev Anti Infect Ther 2014;12:673–85. 10.1586/14787210.2014.887440 24555561

[1127] Helms DJ, Mosure DJ, Secor WE, Workowski KA. Management of *Trichomonas vaginalis* in women with suspected metronidazole hypersensitivity. Am J Obstet Gynecol 2008;198:370.e1–7. 10.1016/j.ajog.2007.10.795 18221927

[1128] Gendelman SR, Pien LC, Gutta RC, Abouhassan SR. Modified oral metronidazole desensitization protocol. Allergy Rhinol (Providence) 2014;5:66–9. 10.2500/ar.2014.5.0080 24612959 PMC4124580

[1129] Nyirjesy P, Sobel JD, Weitz MV, Leaman DJ, Gelone SP. Difficult-to-treat trichomoniasis: results with paromomycin cream. Clin Infect Dis 1998;26:986–8. 10.1086/513951 9564487

[1130] Klebanoff MA, Carey JC, Hauth JC, ; National Institute of Child Health and Human Development Network of Maternal-Fetal Medicine Units. Failure of metronidazole to prevent preterm delivery among pregnant women with asymptomatic *Trichomonas vaginalis* infection. N Engl J Med 2001;345:487–93. 10.1056/NEJMoa003329 11519502

[1131] Stringer E, Read JS, Hoffman I, Valentine M, Aboud S, Goldenberg RL. Treatment of trichomoniasis in pregnancy in sub-Saharan Africa does not appear to be associated with low birth weight or preterm birth. S Afr Med J 2010;100:58–64. 20429491 PMC3090676

[1132] Caro-Patón T, Carvajal A, Martin de Diego I, Martin-Arias LH, Alvarez Requejo A, Rodríguez Pinilla E. Is metronidazole teratogenic? A meta-analysis. Br J Clin Pharmacol 1997;44:179–82. 10.1046/j.1365-2125.1997.00660.x 9278206 PMC2042833

[1133] Gülmezoglu AM. Interventions for trichomoniasis in pregnancy. Cochrane Database Syst Rev 2002;(3):CD000220. 12137609 10.1002/14651858.CD000220

[1134] Goldenberg RL, Mwatha A, Read JS, ; Hptn024 Team. The HPTN 024 Study: the efficacy of antibiotics to prevent chorioamnionitis and preterm birth. Am J Obstet Gynecol 2006;194:650–61. 10.1016/j.ajog.2006.01.004 16522393

[1135] Mann JR, McDermott S, Zhou L, Barnes TL, Hardin J. Treatment of trichomoniasis in pregnancy and preterm birth: an observational study. J Womens Health (Larchmt) 2009;18:493–7. 10.1089/jwh.2008.0964 19361316

[1136] Carter JE, Whithaus KC. Neonatal respiratory tract involvement by *Trichomonas vaginalis*: a case report and review of the literature. Am J Trop Med Hyg 2008;78:17–9. 10.4269/ajtmh.2008.78.17 18187779

[1137] Trintis J, Epie N, Boss R, Riedel S. Neonatal *Trichomonas vaginalis* infection: a case report and review of literature. Int J STD AIDS 2010;21:606–7. 10.1258/ijsa.2010.010174 20975098

[1138] Miller M, Liao Y, Wagner M, Korves C. HIV, the clustering of sexually transmitted infections, and sex risk among African American women who use drugs. Sex Transm Dis 2008;35:696–702. 10.1097/OLQ.0b013e31816b1fb8 18418289

[1139] Anderson BL, Firnhaber C, Liu T, Effect of trichomoniasis therapy on genital HIV viral burden among African women. Sex Transm Dis 2012;39:638–42. 10.1097/OLQ.0b013e31825725ad 22797689 PMC3398383

[1140] Masese LN, Graham SM, Gitau R, A prospective study of vaginal trichomoniasis and HIV-1 shedding in women on antiretroviral therapy. BMC Infect Dis 2011;11:307. 10.1186/1471-2334-11-307 22047086 PMC3231993

[1141] Balkus JE, Richardson BA, Mochache V, A prospective cohort study comparing the effect of single-dose 2 g metronidazole on *Trichomonas vaginalis* infection in HIV-seropositive versus HIV-seronegative women. Sex Transm Dis 2013;40:499–505. 10.1097/OLQ.0b013e31828fce34 23677023 PMC3676301

[1142] Gumbo FZ, Duri K, Kandawasvika GQ, Risk factors of HIV vertical transmission in a cohort of women under a PMTCT program at three peri-urban clinics in a resource-poor setting. J Perinatol 2010;30:717–23. 10.1038/jp.2010.31 20336078 PMC2994594

[1143] Brüggemann RJ, Alffenaar JW, Blijlevens NM, Clinical relevance of the pharmacokinetic interactions of azole antifungal drugs with other coadministered agents. Clin Infect Dis 2009;48:1441–58. 10.1086/598327 19361301

[1144] Shahid Z, Sobel JD. Reduced fluconazole susceptibility of *Candida albicans* isolates in women with recurrent vulvovaginal candidiasis: effects of long-term fluconazole therapy. Diagn Microbiol Infect Dis 2009;64:354–6. 10.1016/j.diagmicrobio.2009.03.021 19501794

[1145] Marchaim D, Lemanek L, Bheemreddy S, Kaye KS, Sobel JD. Fluconazole-resistant *Candida albicans* vulvovaginitis. Obstet Gynecol 2012;120:1407–14. 10.1097/AOG.0b013e31827307b2 23168767

[1146] Denning DW, Kneale M, Sobel JD, Rautemaa-Richardson R. Global burden of recurrent vulvovaginal candidiasis: a systematic review. Lancet Infect Dis 2018;18:e339–47. 10.1016/S1473-3099(18)30103-8 30078662

[1147] Crouss T, Sobel JD, Smith K, Nyirjesy P. Long-term outcomes of women with recurrent vulvovaginal candidiasis after a course of maintenance antifungal therapy. J Low Genit Tract Dis 2018;22:382–6. 10.1097/LGT.0000000000000413 29975334

[1148] Kennedy MA, Sobel JD. Vulvovaginal candidiasis caused by non-*albicans* *Candida* species: new insights. Curr Infect Dis Rep 2010;12:465–70. 10.1007/s11908-010-0137-9 21308556

[1149] Sobel JD, Chaim W, Nagappan V, Leaman D. Treatment of vaginitis caused by *Candida glabrata*: use of topical boric acid and flucytosine. Am J Obstet Gynecol 2003;189:1297–300. 10.1067/S0002-9378(03)00726-9 14634557

[1150] Mølgaard-Nielsen D, Svanström H, Melbye M, Hviid A, Pasternak B. Association between use of oral fluconazole during pregnancy and risk of spontaneous abortion and stillbirth. JAMA 2016;315:58–67. 10.1001/jama.2015.17844 26746458

[1151] Bérard A, Sheehy O, Zhao JP, Associations between low- and high-dose oral fluconazole and pregnancy outcomes: 3 nested case-control studies. CMAJ 2019;191:E179–87. 10.1503/cmaj.180963 30782643 PMC6379167

[1152] Ohmit SE, Sobel JD, Schuman P, ; HIV Epidemiology Research Study (HERS) Group. Longitudinal study of mucosal *Candida* species colonization and candidiasis among human immunodeficiency virus (HIV)-seropositive and at-risk HIV-seronegative women. J Infect Dis 2003;188:118–27. 10.1086/375746 12825180

[1153] Duerr A, Heilig CM, Meikle SF, ; HER Study Group. Incident and persistent vulvovaginal candidiasis among human immunodeficiency virus-infected women: risk factors and severity. Obstet Gynecol 2003;101:548–56. 10.1097/00006250-200303000-00022 12636961

[1154] Vazquez JA, Peng G, Sobel JD, Evolution of antifungal susceptibility among *Candida* species isolates recovered from human immunodeficiency virus-infected women receiving fluconazole prophylaxis. Clin Infect Dis 2001;33:1069–75. 10.1086/322641 11528582

[1155] Darville T; Pelvic Inflammatory Disease Workshop Proceedings Committee. Pelvic inflammatory disease: identifying research gaps—proceedings of a workshop sponsored by Department of Health and Human Services/National Institutes of Health/National Institute of Allergy and Infectious Diseases, November 3–4, 2011. Sex Transm Dis 2013;40:761–7. 10.1097/OLQ.0000000000000028 24275724 PMC5911392

[1156] Wiesenfeld HC, Sweet RL, Ness RB, Krohn MA, Amortegui AJ, Hillier SL. Comparison of acute and subclinical pelvic inflammatory disease. Sex Transm Dis 2005;32:400–5. 10.1097/01.olq.0000154508.26532.6a 15976596

[1157] Wiesenfeld HC, Hillier SL, Meyn LA, Amortegui AJ, Sweet RL. Subclinical pelvic inflammatory disease and infertility. Obstet Gynecol 2012;120:37–43. 10.1097/AOG.0b013e31825a6bc9 22678036

[1158] Ness RB, Soper DE, Holley RL, Effectiveness of inpatient and outpatient treatment strategies for women with pelvic inflammatory disease: results from the Pelvic Inflammatory Disease Evaluation and Clinical Health (PEACH) Randomized Trial. Am J Obstet Gynecol 2002;186:929–37. 10.1067/mob.2002.121625 12015517

[1159] Burnett AM, Anderson CP, Zwank MD. Laboratory-confirmed gonorrhea and/or chlamydia rates in clinically diagnosed pelvic inflammatory disease and cervicitis. Am J Emerg Med 2012;30:1114–7. 10.1016/j.ajem.2011.07.014 22030186

[1160] Wiesenfeld HC, Meyn LA, Darville T, Macio IS, Hillier SL. A randomized controlled trial of ceftriaxone and doxycycline, with or without metronidazole, for the treatment of acute pelvic inflammatory disease. Clin Infect Dis 2021;72:1181–9. 10.1093/cid/ciaa101 32052831 PMC8028096

[1161] Ness RB, Kip KE, Hillier SL, A cluster analysis of bacterial vaginosis-associated microflora and pelvic inflammatory disease. Am J Epidemiol 2005;162:585–90. 10.1093/aje/kwi243 16093289

[1162] Scholes D, Stergachis A, Heidrich FE, Andrilla H, Holmes KK, Stamm WE. Prevention of pelvic inflammatory disease by screening for cervical chlamydial infection. N Engl J Med 1996;334:1362–6. 10.1056/NEJM199605233342103 8614421

[1163] Oakeshott P, Kerry S, Aghaizu A, Randomised controlled trial of screening for *Chlamydia trachomatis* to prevent pelvic inflammatory disease: the POPI (Prevention of Pelvic Infection) trial. BMJ 2010;340:c1642. 10.1136/bmj.c1642 20378636 PMC2851939

[1164] Peipert JF, Ness RB, Blume J, ; Pelvic Inflammatory Disease Evaluation and Clinical Health Study Investigators. Clinical predictors of endometritis in women with symptoms and signs of pelvic inflammatory disease. Am J Obstet Gynecol 2001;184:856–64. 10.1067/mob.2001.113847 11303192

[1165] Gaitán H, Angel E, Diaz R, Parada A, Sanchez L, Vargas C. Accuracy of five different diagnostic techniques in mild-to-moderate pelvic inflammatory disease. Infect Dis Obstet Gynecol 2002;10:171–80. 10.1155/S1064744902000194 12648310 PMC1784624

[1166] Vicetti Miguel RD, Chivukula M, Krishnamurti U, Limitations of the criteria used to diagnose histologic endometritis in epidemiologic pelvic inflammatory disease research. Pathol Res Pract 2011;207:680–5. 10.1016/j.prp.2011.08.007 21996319 PMC3215901

[1167] Jacobson L, Weström L. Objectivized diagnosis of acute pelvic inflammatory disease. Diagnostic and prognostic value of routine laparoscopy. Am J Obstet Gynecol 1969;105:1088–98. 10.1016/0002-9378(69)90132-X 4242830

[1168] Sellors J, Mahony J, Goldsmith C, The accuracy of clinical findings and laparoscopy in pelvic inflammatory disease. Am J Obstet Gynecol 1991;164:113–20. 10.1016/0002-9378(91)90639-9 1824740

[1169] Bevan CD, Johal BJ, Mumtaz G, Ridgway GL, Siddle NC. Clinical, laparoscopic and microbiological findings in acute salpingitis: report on a United Kingdom cohort. Br J Obstet Gynaecol 1995;102:407–14. 10.1111/j.1471-0528.1995.tb11294.x 7612536

[1170] Jaiyeoba O, Soper DE. A practical approach to the diagnosis of pelvic inflammatory disease. Infect Dis Obstet Gynecol 2011;2011:753037. 10.1155/2011/753037 21822367 PMC3148590

[1171] Sweet RL. Treatment of acute pelvic inflammatory disease. Infect Dis Obstet Gynecol 2011;2011:561909. 10.1155/2011/561909 22228985 PMC3249632

[1172] Smith KJ, Ness RB, Wiesenfeld HC, Roberts MS. Cost-effectiveness of alternative outpatient pelvic inflammatory disease treatment strategies. Sex Transm Dis 2007;34:960–6. 10.1097/01.olq.0000225321.61049.13 18077847

[1173] Petrina MAB, Cosentino LA, Wiesenfeld HC, Darville T, Hillier SL. Susceptibility of endometrial isolates recovered from women with clinical pelvic inflammatory disease or histological endometritis to antimicrobial agents. Anaerobe 2019;56:61–5. 10.1016/j.anaerobe.2019.02.005 30753898 PMC6559736

[1174] Haggerty CL, Ness RB, Amortegui A, Endometritis does not predict reproductive morbidity after pelvic inflammatory disease. Am J Obstet Gynecol 2003;188:141–8. 10.1067/mob.2003.87 12548208

[1175] Haggerty CL, Totten PA, Tang G, Identification of novel microbes associated with pelvic inflammatory disease and infertility. Sex Transm Infect 2016;92:441–6. 10.1136/sextrans-2015-052285 26825087 PMC5013099

[1176] Ness RB, Randall H, Richter HE, ; Pelvic Inflammatory Disease Evaluation and Clinical Health Study Investigators. Condom use and the risk of recurrent pelvic inflammatory disease, chronic pelvic pain, or infertility following an episode of pelvic inflammatory disease. Am J Public Health 2004;94:1327–9. 10.2105/AJPH.94.8.1327 15284036 PMC1448448

[1177] McGregor JA, Crombleholme WR, Newton E, Sweet RL, Tuomala R, Gibbs RS. Randomized comparison of ampicillin-sulbactam to cefoxitin and doxycycline or clindamycin and gentamicin in the treatment of pelvic inflammatory disease or endometritis. Obstet Gynecol 1994;83:998–1004. 10.1097/00006250-199406000-00020 8190448

[1178] Bevan CD, Ridgway GL, Rothermel CD. Efficacy and safety of azithromycin as monotherapy or combined with metronidazole compared with two standard multidrug regimens for the treatment of acute pelvic inflammatory disease. J Int Med Res 2003;31:45–54. 10.1177/147323000303100108 12635534

[1179] Heystek M, Ross JD; PID Study Group. A randomized double-blind comparison of moxifloxacin and doxycycline/metronidazole/ciprofloxacin in the treatment of acute, uncomplicated pelvic inflammatory disease. Int J STD AIDS 2009;20:690–5. 10.1258/ijsa.2008.008495 19815913

[1180] Boothby M, Page J, Pryor R, Ross JD. A comparison of treatment outcomes for moxifloxacin versus ofloxacin/metronidazole for first-line treatment of uncomplicated non-gonococcal pelvic inflammatory disease. Int J STD AIDS 2010;21:195–7. 10.1258/ijsa.2009.009374 20215625

[1181] Judlin P, Liao Q, Liu Z, Reimnitz P, Hampel B, Arvis P. Efficacy and safety of moxifloxacin in uncomplicated pelvic inflammatory disease: the MONALISA study. BJOG 2010;117:1475–84. 10.1111/j.1471-0528.2010.02687.x 20716255

[1182] Korn AP. Pelvic inflammatory disease in women infected with HIV. AIDS Patient Care STDS 1998;12:431–4. 10.1089/apc.1998.12.431 11361990

[1183] Irwin KL, Moorman AC, O’Sullivan MJ, Influence of human immunodeficiency virus infection on pelvic inflammatory disease. Obstet Gynecol 2000;95:525–34. 10725484 10.1016/s0029-7844(99)00621-3

[1184] Bukusi EA, Cohen CR, Stevens CE, Effects of human immunodeficiency virus 1 infection on microbial origins of pelvic inflammatory disease and on efficacy of ambulatory oral therapy. Am J Obstet Gynecol 1999;181:1374–81. 10.1016/S0002-9378(99)70378-9 10601915

[1185] Mugo NR, Kiehlbauch JA, Nguti R, Effect of human immunodeficiency virus-1 infection on treatment outcome of acute salpingitis. Obstet Gynecol 2006;107:807–12. 10.1097/01.AOG.0000207597.70524.e8 16582116

[1186] Grimes DA. Intrauterine device and upper-genital-tract infection. Lancet 2000;356:1013–9. 10.1016/S0140-6736(00)02699-4 11041414

[1187] Viberga I, Odlind V, Lazdane G, Kroica J, Berglund L, Olofsson S. Microbiology profile in women with pelvic inflammatory disease in relation to IUD use. Infect Dis Obstet Gynecol 2005;13:183–90. 10.1155/2005/376830 16338777 PMC1784576

[1188] Jatlaoui TC, Riley HEM, Curtis KM. The safety of intrauterine devices among young women: a systematic review. Contraception 2017;95:17–39. 10.1016/j.contraception.2016.10.006 27771475 PMC6511984

[1189] Chen MJ, Kim CR, Whitehouse KC, Berry-Bibee E, Gaffield ME. Development, updates, and future directions of the World Health Organization Selected Practice Recommendations for Contraceptive Use. Int J Gynaecol Obstet 2017;136:113–9. 10.1002/ijgo.12064 28099730 PMC6546088

[1190] Tepper NK, Steenland MW, Gaffield ME, Marchbanks PA, Curtis KM. Retention of intrauterine devices in women who acquire pelvic inflammatory disease: a systematic review. Contraception 2013;87:655–60. 10.1016/j.contraception.2012.08.011 23040135

[1191] Louette A, Krahn J, Caine V, Ha S, Lau TTY, Singh AE. Treatment of acute epididymitis: a systematic review and discussion of the implications for treatment based on etiology. Sex Transm Dis 2018;45:e104–8. 10.1097/OLQ.0000000000000901 30044339

[1192] Pilatz A, Hossain H, Kaiser R, Acute epididymitis revisited: impact of molecular diagnostics on etiology and contemporary guideline recommendations. Eur Urol 2015;68:428–35. 10.1016/j.eururo.2014.12.005 25542628

[1193] Hongo H, Kikuchi E, Matsumoto K, Novel algorithm for management of acute epididymitis. Int J Urol 2017;24:82–7. 10.1111/iju.13236 27714879

[1194] de Villiers EM, Fauquet C, Broker TR, Bernard HU, zur Hausen H. Classification of papillomaviruses. Virology 2004;324:17–27. 10.1016/j.virol.2004.03.033 15183049

[1195] Myers ER, McCrory DC, Nanda K, Bastian L, Matchar DB. Mathematical model for the natural history of human papillomavirus infection and cervical carcinogenesis. Am J Epidemiol 2000;151:1158–71. 10.1093/oxfordjournals.aje.a010166 10905528

[1196] Chesson HW, Dunne EF, Hariri S, Markowitz LE. The estimated lifetime probability of acquiring human papillomavirus in the United States. Sex Transm Dis 2014;41:660–4. 10.1097/OLQ.0000000000000193 25299412 PMC6745688

[1197] Cogliano V, Baan R, Straif K, Grosse Y, Secretan B, El Ghissassi F; WHO International Agency for Research on Cancer. Carcinogenicity of human papillomaviruses. Lancet Oncol 2005;6:204. 10.1016/S1470-2045(05)70086-3 15830458

[1198] Senkomago V, Henley SJ, Thomas CC, Mix JM, Markowitz LE, Saraiya M. Human papillomavirus-attributable cancers—United States, 2012–2016. MMWR Morb Mortal Wkly Rep 2019;68:724–8. 10.15585/mmwr.mm6833a3 31437140 PMC6705893

[1199] Chesson HW, Ekwueme DU, Saraiya M, Watson M, Lowy DR, Markowitz LE. Estimates of the annual direct medical costs of the prevention and treatment of disease associated with human papillomavirus in the United States. Vaccine 2012;30:6016–9. 10.1016/j.vaccine.2012.07.056 22867718 PMC6629018

[1200] Markowitz LE, Hariri S, Lin C, Reduction in human papillomavirus (HPV) prevalence among young women following HPV vaccine introduction in the United States, National Health and Nutrition Examination Surveys, 2003–2010. J Infect Dis 2013;208:385–93. 10.1093/infdis/jit192 23785124

[1201] Flagg EW, Schwartz R, Weinstock H. Prevalence of anogenital warts among participants in private health plans in the United States, 2003–2010: potential impact of human papillomavirus vaccination. Am J Public Health 2013;103:1428–35. 10.2105/AJPH.2012.301182 23763409 PMC4007878

[1202] McClung NM, Lewis RM, Gargano JW, Querec T, Unger ER, Markowitz LE. Declines in vaccine-type human papillomavirus prevalence in females across racial/ethnic groups: data from a national survey. J Adolesc Health 2019;65:715–22. 10.1016/j.jadohealth.2019.07.003 31515134

[1203] Drolet M, Bénard É, Pérez N, ; HPV Vaccination Impact Study Group. Population-level impact and herd effects following the introduction of human papillomavirus vaccination programmes: updated systematic review and meta-analysis. Lancet 2019;394:497–509. 10.1016/S0140-6736(19)30298-3 31255301 PMC7316527

[1204] Mayhew A, Mullins TL, Ding L, Risk perceptions and subsequent sexual behaviors after HPV vaccination in adolescents. Pediatrics 2014;133:404–11. 10.1542/peds.2013-2822 24488747 PMC3934341

[1205] Brouwer AF, Delinger RL, Eisenberg MC, HPV vaccination has not increased sexual activity or accelerated sexual debut in a college-aged cohort of men and women. BMC Public Health 2019;19:821. 10.1186/s12889-019-7134-1 31238911 PMC6593582

[1206] Garland SM, Steben M, Sings HL, Natural history of genital warts: analysis of the placebo arm of 2 randomized phase III trials of a quadrivalent human papillomavirus (types 6, 11, 16, and 18) vaccine. J Infect Dis 2009;199:805–14. 10.1086/597071 19199546

[1207] Flagg EW, Torrone EA. Declines in anogenital warts among age groups most likely to be impacted by human papillomavirus vaccination, United States, 2006–2014. Am J Public Health 2018;108:112–9. 10.2105/AJPH.2017.304119 29161070 PMC5719685

[1208] Hariri S, Schuler MS, Naleway AL, Human papillomavirus vaccine effectiveness against incident genital warts among female health-plan enrollees, United States. Am J Epidemiol 2018;187:298–305. 10.1093/aje/kwx253 28641366

[1209] Wangu Z, Hsu KK. Impact of HPV vaccination on anogenital warts and respiratory papillomatosis. Hum Vaccin Immunother 2016;12:1357–62. 10.1080/21645515.2016.1172754 27217191 PMC5036961

[1210] Swedish KA, Goldstone SE. Prevention of anal condyloma with quadrivalent human papillomavirus vaccination of older men who have sex with men. PLoS One 2014;9:e93393. 10.1371/journal.pone.0093393 24714693 PMC3979673

[1211] Sandø N, Kofoed K, Zachariae C, Fouchard J. A reduced national incidence of anogenital warts in young Danish men and women after introduction of a national quadrivalent human papillomavirus vaccination programme for young women—an ecological study. Acta Derm Venereol 2014;94:288–92. 10.2340/00015555-1721 24150529

[1212] Herweijer E, Ploner A, Sparén P. Substantially reduced incidence of genital warts in women and men six years after HPV vaccine availability in Sweden. Vaccine 2018;36:1917–20. 10.1016/j.vaccine.2018.02.097 29523448

[1213] Harrison C, Britt H, Garland S, Decreased management of genital warts in young women in Australian general practice post introduction of national HPV vaccination program: results from a nationally representative cross-sectional general practice study. PLoS One 2014;9:e105967. 10.1371/journal.pone.0105967 25180698 PMC4152193

[1214] Canvin M, Sinka K, Hughes G, Mesher D. Decline in genital warts diagnoses among young women and young men since the introduction of the bivalent HPV (16/18) vaccination programme in England: an ecological analysis. Sex Transm Infect 2017;93:125–8. 10.1136/sextrans-2016-052626 27365492

[1215] Chow EP, Read TR, Wigan R, Ongoing decline in genital warts among young heterosexuals 7 years after the Australian human papillomavirus (HPV) vaccination programme. Sex Transm Infect 2015;91:214–9. 10.1136/sextrans-2014-051813 25305210

[1216] Petrosky EY, Liu G, Hariri S, Markowitz LE. Human papillomavirus vaccination and age at first sexual activity, National Health and Nutrition Examination Survey. Clin Pediatr (Phila) 2017;56:363–70. 10.1177/0009922816660541 27609513 PMC5342939

[1217] Gotovtseva EP, Kapadia AS, Smolensky MH, Lairson DR. Optimal frequency of imiquimod (aldara) 5% cream for the treatment of external genital warts in immunocompetent adults: a meta-analysis. Sex Transm Dis 2008;35:346–51. 10.1097/OLQ.0b013e31815ea8d1 18360317

[1218] Baker DA, Ferris DG, Martens MG, Imiquimod 3.75% cream applied daily to treat anogenital warts: combined results from women in two randomized, placebo-controlled studies. Infect Dis Obstet Gynecol 2011;2011:806105. 10.1155/2011/806105 21876641 PMC3162968

[1219] Mashiah J, Brenner S. Possible mechanisms in the induction of vitiligo-like hypopigmentation by topical imiquimod. Clin Exp Dermatol 2008;33:74–6. 17979992 10.1111/j.1365-2230.2007.02520.x

[1220] Domingues E, Chaney KC, Scharf MJ, Wiss K. Imiquimod reactivation of lichen planus. Cutis 2012;89:276–7, 283. 22838091

[1221] Patel U, Mark NM, Machler BC, Levine VJ. Imiquimod 5% cream induced psoriasis: a case report, summary of the literature and mechanism. Br J Dermatol 2011;164:670–2. 10.1111/j.1365-2133.2010.10124.x 21062268

[1222] Kumar B, Narang T. Local and systemic adverse effects to topical imiquimod due to systemic immune stimulation. Sex Transm Infect 2011;87:432. 10.1136/sextrans-2011-050025 21606474

[1223] Stockfleth E, Beti H, Orasan R, Topical Polyphenon E in the treatment of external genital and perianal warts: a randomized controlled trial. Br J Dermatol 2008;158:1329–38. 10.1111/j.1365-2133.2008.08520.x 18363746

[1224] Gross G, Meyer KG, Pres H, Thielert C, Tawfik H, Mescheder A. A randomized, double-blind, four-arm parallel-group, placebo-controlled Phase II/III study to investigate the clinical efficacy of two galenic formulations of Polyphenon E in the treatment of external genital warts. J Eur Acad Dermatol Venereol 2007;21:1404–12. 10.1111/j.1468-3083.2007.02441.x 17958849

[1225] Tatti S, Swinehart JM, Thielert C, Tawfik H, Mescheder A, Beutner KR. Sinecatechins, a defined green tea extract, in the treatment of external anogenital warts: a randomized controlled trial. Obstet Gynecol 2008;111:1371–9. 10.1097/AOG.0b013e3181719b60 18515521

[1226] National Institute for Occupational Safety and Health. Control of smoke from laser/electric surgical procedures. Washington, DC: US Department of Health and Human Services, CDC, National Institute for Occupational Safety and Health; 1996. https://www.cdc.gov/niosh/docs/hazardcontrol/pdfs/hc11.pdf?id=10.26616/NIOSHPUB96128 10.1080/104732299303205 10457631

[1227] Filley CM, Graff-Richard NR, Lacy JR, Heitner MA, Earnest MP. Neurologic manifestations of podophyllin toxicity. Neurology 1982;32:308–11. 10.1212/WNL.32.3.308 7199647

[1228] Conard PF, Hanna N, Rosenblum M, Gross JB. Delayed recognition of podophyllum toxicity in a patient receiving epidural morphine. Anesth Analg 1990;71:191–3. 10.1213/00000539-199008000-00013 2375521

[1229] Karol MD, Conner CS, Watanabe AS, Murphrey KJ. Podophyllum: suspected teratogenicity from topical application. Clin Toxicol 1980;16:283–6. 10.3109/15563658008989950 7398215

[1230] Silverberg MJ, Thorsen P, Lindeberg H, Grant LA, Shah KV. Condyloma in pregnancy is strongly predictive of juvenile-onset recurrent respiratory papillomatosis. Obstet Gynecol 2003;101:645–52. 12681865 10.1016/s0029-7844(02)03081-8

[1231] Dolev JC, Maurer T, Springer G, Incidence and risk factors for verrucae in women. AIDS 2008;22:1213–9. 10.1097/QAD.0b013e3283021aa3 18525267 PMC2615554

[1232] Silverberg MJ, Ahdieh L, Munoz A, The impact of HIV infection and immunodeficiency on human papillomavirus type 6 or 11 infection and on genital warts. Sex Transm Dis 2002;29:427–35. 10.1097/00007435-200208000-00001 12172526

[1233] De Panfilis G, Melzani G, Mori G, Ghidini A, Graifemberghi S. Relapses after treatment of external genital warts are more frequent in HIV-positive patients than in HIV-negative controls. Sex Transm Dis 2002;29:121–5. 10.1097/00007435-200203000-00001 11875372

[1234] Conley LJ, Ellerbrock TV, Bush TJ, Chiasson MA, Sawo D, Wright TC. HIV-1 infection and risk of vulvovaginal and perianal condylomata acuminata and intraepithelial neoplasia: a prospective cohort study. Lancet 2002;359:108–13. 10.1016/S0140-6736(02)07368-3 11809252

[1235] Schlecht HP, Fugelso DK, Murphy RK, Frequency of occult high-grade squamous intraepithelial neoplasia and invasive cancer within anal condylomata in men who have sex with men. Clin Infect Dis 2010;51:107–10. 10.1086/653426 20482370 PMC4460603

[1236] Maniar KP, Ronnett BM, Vang R, Yemelyanova A. Coexisting high-grade vulvar intraepithelial neoplasia (VIN) and condyloma acuminatum: independent lesions due to different HPV types occurring in immunocompromised patients. Am J Surg Pathol 2013;37:53–60. 10.1097/PAS.0b013e318263cda6 23026935 PMC3524383

[1237] Massad LS, Xie X, Darragh T, ; Women’s Interagency HIV Study Collaborative Study Group. Genital warts and vulvar intraepithelial neoplasia: natural history and effects of treatment and human immunodeficiency virus infection. Obstet Gynecol 2011;118:831–9. 10.1097/AOG.0b013e31821a0f4d 21934446 PMC3178036

[1238] Forman D, de Martel C, Lacey CJ, Global burden of human papillomavirus and related diseases. Vaccine 2012;30(Suppl 5):F12–23. 10.1016/j.vaccine.2012.07.055 23199955

[1239] Darragh TM, Colgan TJ, Cox JT, ; Members of LAST Project Work Groups. The lower anogenital squamous terminology standardization project for HPV-associated lesions: background and consensus recommendations from the College of American Pathologists and the American Society for Colposcopy and Cervical Pathology. Arch Pathol Lab Med 2012;136:1266–97. 10.5858/arpa.LGT200570 22742517

[1240] Committee on Practice Bulletins—Gynecology. Practice Bulletin No. 168 Summary: Cervical cancer screening and prevention. Obstet Gynecol 2016;128:923–5. 10.1097/AOG.0000000000001699 27661643

[1241] Perkins RB, Guido RL, Saraiya M, Summary of current guidelines for cervical cancer screening and management of abnormal test results: 2016–2020. J Womens Health (Larchmt) 2021;30:5–13. 10.1089/jwh.2020.8918 33464997 PMC8020523

[1242] Kim JJ, Burger EA, Regan C, Sy S. Screening for cervical cancer in primary care: a decision analysis for the US Preventive Services Task Force. JAMA 2018;320:706–14. 10.1001/jama.2017.19872 30140882 PMC8653579

[1243] Sawaya GF, Sanstead E, Alarid-Escudero F, Estimated quality of life and economic outcomes associated with 12 cervical cancer screening strategies: a cost-effectiveness analysis. JAMA Intern Med 2019;179:867–78. 10.1001/jamainternmed.2019.0299 31081851 PMC6515585

[1244] Saslow D, Solomon D, Lawson HW, ; ACS-ASCCP-ASCP Cervical Cancer Guideline Committee. American Cancer Society, American Society for Colposcopy and Cervical Pathology, and American Society for Clinical Pathology screening guidelines for the prevention and early detection of cervical cancer. CA Cancer J Clin 2012;62:147–72. 10.3322/caac.21139 22422631 PMC3801360

[1245] Committee on Practice Bulletins—Gynecology. ACOG Practice Bulletin No. 131: Screening for cervical cancer. Obstet Gynecol 2012;120:1222–38. 10.1097/AOG.0b013e318277c92a 23090560

[1246] Meyerson BE, Sayegh MA, Davis A, Cervical cancer screening in a sexually transmitted disease clinic: screening adoption experiences from a midwestern clinic. Am J Public Health 2015;105(Suppl 2):e8–14. 10.2105/AJPH.2014.302272 25689199 PMC4355685

[1247] Perkins RB, Guido RS, Castle PE, ; 2019 ASCCP Risk-Based Management Consensus Guidelines Committee. 2019 ASCCP risk-based management consensus guidelines for abnormal cervical cancer screening tests and cancer precursors. J Low Genit Tract Dis 2020;24:102–31. 10.1097/LGT.0000000000000525 32243307 PMC7147428

[1248] Saraiya M, Lee NC, Blackman D, Smith MJ, Morrow B, McKenna MA. Self-reported Papanicolaou smears and hysterectomies among women in the United States. Obstet Gynecol 2001;98:269–78. 11506844 10.1016/s0029-7844(01)01447-8

[1249] Sirovich BE, Welch HG. Cervical cancer screening among women without a cervix. JAMA 2004;291:2990–3. 10.1001/jama.291.24.2990 15213211

[1250] Stokes-Lampard H, Wilson S, Waddell C, Ryan A, Holder R, Kehoe S. Vaginal vault smears after hysterectomy for reasons other than malignancy: a systematic review of the literature. BJOG 2006;113:1354–65. 10.1111/j.1471-0528.2006.01099.x 17081187

[1251] Arbyn M, Herbert A, Schenck U, European guidelines for quality assurance in cervical cancer screening: recommendations for collecting samples for conventional and liquid-based cytology. Cytopathology 2007;18:133–9. 10.1111/j.1365-2303.2007.00464.x 17573762

[1252] Daley E, Perrin K, Vamos C, Confusion about Pap smears: lack of knowledge among high-risk women. J Womens Health (Larchmt) 2013;22:67–74. 10.1089/jwh.2012.3667 23215902

[1253] Drolet M, Bénard É, Boily MC, Population-level impact and herd effects following human papillomavirus vaccination programmes: a systematic review and meta-analysis. Lancet Infect Dis 2015;15:565–80. 10.1016/S1473-3099(14)71073-4 25744474 PMC5144106

[1254] Ogbechie OA, Hacker MR, Dodge LE, Patil MM, Ricciotti HA. Confusion regarding cervical cancer screening and chlamydia screening among sexually active young women. Sex Transm Infect 2012;88:35–7. 10.1136/sextrans-2011-050289 22123163 PMC3724364

[1255] Dunne EF, Friedman A, Datta SD, Markowitz LE, Workowski KA. Updates on human papillomavirus and genital warts and counseling messages from the 2010 Sexually Transmitted Diseases Treatment Guidelines. Clin Infect Dis 2011;53(Suppl 3):S143–52. 10.1093/cid/cir703 22080267

[1256] Fry AM, Ferries-Rowe EA, Learman LA, Haas DM. Pap smear versus speculum examination: can we teach providers to educate patients? J Womens Health (Larchmt) 2010;19:1715–9. 10.1089/jwh.2009.1862 20662627

[1257] Adab P, Marshall T, Rouse A, Randhawa B, Sangha H, Bhangoo N. Randomised controlled trial of the effect of evidence based information on women’s willingness to participate in cervical cancer screening. J Epidemiol Community Health 2003;57:589–93. 10.1136/jech.57.8.589 12883063 PMC1732533

[1258] Drolet M, Brisson M, Maunsell E, The psychosocial impact of an abnormal cervical smear result. Psychooncology 2012;21:1071–81. 10.1002/pon.2003 21695747

[1259] McCaffery KJ, Irwig L, Turner R, Psychosocial outcomes of three triage methods for the management of borderline abnormal cervical smears: an open randomised trial. BMJ 2010;340(feb23 1):b4491. 10.1136/bmj.b4491 20179125 PMC2827716

[1260] Daley EM, Perrin KM, McDermott RJ, The psychosocial burden of HPV: a mixed-method study of knowledge, attitudes and behaviors among HPV+ women. J Health Psychol 2010;15:279–90. 10.1177/1359105309351249 20207671

[1261] Pirotta M, Ung L, Stein A, The psychosocial burden of human papillomavirus related disease and screening interventions. Sex Transm Infect 2009;85:508–13. 10.1136/sti.2009.037028 19703844

[1262] Lin L, Benard VB, Greek A, Roland KB, Hawkins NA, Saraiya M. Communication practices about HPV testing among providers in Federally Qualified Health Centers. Prev Med Rep 2015;2:436–9. 10.1016/j.pmedr.2015.05.006 26213683 PMC4511727

[1263] Kapeu AS, Luostarinen T, Jellum E, Is smoking an independent risk factor for invasive cervical cancer? A nested case-control study within Nordic biobanks. Am J Epidemiol 2009;169:480–8. 10.1093/aje/kwn354 19074773

[1264] Plummer M, Herrero R, Franceschi S, ; IARC Multi-centre Cervical Cancer Study Group. Smoking and cervical cancer: pooled analysis of the IARC multi-centric case-control study. Cancer Causes Control 2003;14:805–14. 10.1023/B:CACO.0000003811.98261.3e 14682438

[1265] de Sanjosé S, Brotons M, Pavón MA. The natural history of human papillomavirus infection. Best Pract Res Clin Obstet Gynaecol 2018;47:2–13. 10.1016/j.bpobgyn.2017.08.015 28964706

[1266] Louie KS, Castellsague X, de Sanjose S, ; International Agency for Research on Cancer Multicenter Cervical Cancer Study Group. Smoking and passive smoking in cervical cancer risk: pooled analysis of couples from the IARC multicentric case-control studies. Cancer Epidemiol Biomarkers Prev 2011;20:1379–90. 10.1158/1055-9965.EPI-11-0284 21610224

[1267] Nelson HD, Cantor A, Wagner J, Effectiveness of patient navigation to increase cancer screening in populations adversely affected by health disparities: a meta-analysis. J Gen Intern Med 2020;35:3026–35. 10.1007/s11606-020-06020-9 32700218 PMC7573022

[1268] Stillson T, Knight AL, Elswick RK Jr. The effectiveness and safety of two cervical cytologic techniques during pregnancy. J Fam Pract 1997;45:159–63. 9267375

[1269] Foster JC, Smith HL. Use of the Cytobrush for Papanicolaou smear screens in pregnant women. J Nurse Midwifery 1996;41:211–7. 10.1016/0091-2182(96)00013-4 8708804

[1270] Paraiso MF, Brady K, Helmchen R, Roat TW. Evaluation of the endocervical Cytobrush and Cervex-Brush in pregnant women. Obstet Gynecol 1994;84:539–43. 8090390

[1271] Silverberg MJ, Leyden WA, Chi A, Human immunodeficiency virus (HIV)- and non-HIV-associated immunosuppression and risk of cervical neoplasia. Obstet Gynecol 2018;131:47–55. 10.1097/AOG.0000000000002371 29215531 PMC5740002

[1272] Videla S, Tarrats A, Ornelas A, Incidence of cervical high-grade squamous intraepithelial lesions in HIV-1-infected women with no history of cervical pathology: up to 17 years of follow-up. Int J STD AIDS 2019;30:56–63. 10.1177/0956462418792653 30170532

[1273] Liu G, Sharma M, Tan N, Barnabas RV. HIV-positive women have higher risk of human papilloma virus infection, precancerous lesions, and cervical cancer. AIDS 2018;32:795–808. 10.1097/QAD.0000000000001765 29369827 PMC5854529

[1274] Sawaya GF, Lamar R, Perkins RB. Managing minimally abnormal cervical cancer screening test results. JAMA 2020; 324:1557. 10.1001/jama.2020.12488 32975557

[1275] Silverberg MJ, Lau B, Justice AC, ; North American AIDS Cohort Collaboration on Research and Design (NA-ACCORD) of IeDEA. Risk of anal cancer in HIV-infected and HIV-uninfected individuals in North America. Clin Infect Dis 2012;54:1026–34. 10.1093/cid/cir1012 22291097 PMC3297645

[1276] Tomassi MJ, Abbas MA, Klaristenfeld DD. Expectant management surveillance for patients at risk for invasive squamous cell carcinoma of the anus: a large US healthcare system experience. Int J Colorectal Dis 2019;34:47–54. 10.1007/s00384-018-3167-7 30244347

[1277] Colón-López V, Shiels MS, Machin M, Anal cancer risk among people with HIV infection in the United States. J Clin Oncol 2018;36:68–75. 10.1200/JCO.2017.74.9291 29140774 PMC5791846

[1278] Machalek DA, Grulich AE, Jin F, Templeton DJ, Poynten IM. The epidemiology and natural history of anal human papillomavirus infection in men who have sex with men. Sex Health 2012;9:527–37. 10.1071/SH12043 23380235

[1279] Deshmukh AA, Suk R, Shiels MS, Recent trends in squamous cell carcinoma of the anus incidence and mortality in the United States, 2001–2015. J Natl Cancer Inst 2020;112:829–38. 10.1093/jnci/djz219 31742639 PMC7825484

[1280] Edgren G, Sparén P. Risk of anogenital cancer after diagnosis of cervical intraepithelial neoplasia: a prospective population-based study. Lancet Oncol 2007;8:311–6. 10.1016/S1470-2045(07)70043-8 17395104

[1281] Chaturvedi AK, Engels EA, Gilbert ES, Second cancers among 104,760 survivors of cervical cancer: evaluation of long-term risk. J Natl Cancer Inst 2007;99:1634–43. 10.1093/jnci/djm201 17971527

[1282] Suk R, Mahale P, Sonawane K, Trends in risks for second primary cancers associated with index human papillomavirus-associated cancers. JAMA Netw Open 2018;1:e181999. 10.1001/jamanetworkopen.2018.1999 30646145 PMC6324459

[1283] Hillman RJ, Berry-Lawhorn JM, Ong JJ, ; International Anal Neoplasia Society. International Anal Neoplasia Society guidelines for the practice of digital anal rectal examination. J Low Genit Tract Dis 2019;23:138–46. 10.1097/LGT.0000000000000458 30907777

[1284] Ong JJ, Grulich A, Walker S, Baseline findings from the Anal Cancer Examination (ACE) study: screening using digital ano-rectal examination in HIV-positive men who have sex with men. J Med Screen 2016;23:70–6. 10.1177/0969141315604658 26462726

[1285] Read TR, Vodstrcil L, Grulich AE, Acceptability of digital anal cancer screening examinations in HIV-positive homosexual men. HIV Med 2013;14:491–6. 10.1111/hiv.12035 23590621

[1286] Jin F, Grulich AE, Poynten IM, ; SPANC Study Team. The performance of anal cytology as a screening test for anal HSILs in homosexual men. Cancer Cytopathol 2016;124:415–24. 10.1002/cncy.21702 26915346

[1287] Silva M, Peixoto A, Sarmento JA, Coelho R, Macedo G. Anal cytology, histopathology and anoscopy in an anal dysplasia screening program: is anal cytology enough? Rev Esp Enferm Dig 2018;110:109–14. 10.17235/reed.2018.5678/2018 29168646

[1288] Iribarren Díaz M, Ocampo Hermida A, González-Carreró Fojón J, Preliminary results of a screening program for anal cancer and its precursors for HIV-infected men who have sex with men in Vigo-Spain [Spanish]. Rev Esp Enferm Dig 2017;109:242–9. 10.17235/reed.2017.4274/2016 28229612

[1289] Burgos J, Hernández-Losa J, Landolfi S, The role of oncogenic human papillomavirus determination for diagnosis of high-grade anal intraepithelial neoplasia in HIV-infected MSM. AIDS 2017;31:2227–33. 10.1097/QAD.0000000000001605 28723712

[1290] Cheng SH, Wang CC, Chang SL, Chu FY, Hsueh YM. Oncogenic human papillomavirus is not helpful for cytology screening of the precursor lesions of anal cancers in Taiwanese men who are infected with human immunodeficiency virus. Int J Clin Oncol 2015;20:943–51. 10.1007/s10147-015-0804-9 25712159

[1291] Hidalgo-Tenorio C, Rivero-Rodriguez M, Gil-Anguita C, The role of polymerase chain reaction of high-risk human papilloma virus in the screening of high-grade squamous intraepithelial lesions in the anal mucosa of human immunodeficiency virus-positive males having sex with males. PLoS One 2015;10:e0123590. 10.1371/journal.pone.0123590 25849412 PMC4388587

[1292] Richel O, de Vries HJ, van Noesel CJ, Dijkgraaf MG, Prins JM. Comparison of imiquimod, topical fluorouracil, and electrocautery for the treatment of anal intraepithelial neoplasia in HIV-positive men who have sex with men: an open-label, randomised controlled trial. Lancet Oncol 2013;14:346–53. 10.1016/S1470-2045(13)70067-6 23499546

[1293] Goldstone SE, Johnstone AA, Moshier EL. Long-term outcome of ablation of anal high-grade squamous intraepithelial lesions: recurrence and incidence of cancer. Dis Colon Rectum 2014;57:316–23. 10.1097/DCR.0000000000000058 24509453

[1294] Tong WW, Shepherd K, Garland S, ; Study of the Prevention of Anal Cancer (SPANC) team. Human papillomavirus 16-specific T-cell responses and spontaneous regression of anal high-grade squamous intraepithelial lesions. J Infect Dis 2015;211:405–15. 10.1093/infdis/jiu461 25139018

[1295] Tong WW, Jin F, McHugh LC, Progression to and spontaneous regression of high-grade anal squamous intraepithelial lesions in HIV-infected and uninfected men. AIDS 2013;27:2233–43. 10.1097/QAD.0b013e3283633111 24157904

[1296] Shin EC, Jeong SH. Natural history, clinical manifestations, and pathogenesis of hepatitis A. Cold Spring Harb Perspect Med 2018;8:a031708. 10.1101/cshperspect.a031708 29440324 PMC6120688

[1297] Nelson NP, Weng MK, Hofmeister MG, Prevention of hepatitis A virus infection in the United States: recommendations of the Advisory Committee on Immunization Practices, 2020. MMWR Recomm Rep 2020;69(No. RR-5). 10.15585/mmwr.rr6905a1 32614811 PMC8631741

[1298] Foster MA, Hofmeister MG, Kupronis BA, Increase in hepatitis A virus infections—United States, 2013–2018. MMWR Morb Mortal Wkly Rep 2019;68:413–5. 10.15585/mmwr.mm6818a2 31071072 PMC6542191

[1299] Bower WA, Nainan OV, Han X, Margolis HS. Duration of viremia in hepatitis A virus infection. J Infect Dis 2000;182:12–7. 10.1086/315701 10882576

[1300] Clemens R, Safary A, Hepburn A, Roche C, Stanbury WJ, André FE. Clinical experience with an inactivated hepatitis A vaccine. J Infect Dis 1995;171(Suppl 1):S44–9. 10.1093/infdis/171.Supplement_1.S44 7876648

[1301] Sharapov UM, Bulkow LR, Negus SE, Persistence of hepatitis A vaccine induced seropositivity in infants and young children by maternal antibody status: 10-year follow-up. Hepatology 2012;56:516–22. 10.1002/hep.25687 22371069

[1302] Mosites E, Gounder P, Snowball M, Hepatitis A vaccine immune response 22 years after vaccination. J Med Virol 2018;90:1418–22. 10.1002/jmv.25197 29663458

[1303] Theeten H, Van Herck K, Van Der Meeren O, Crasta P, Van Damme P, Hens N. Long-term antibody persistence after vaccination with a 2-dose Havrix (inactivated hepatitis A vaccine): 20 years of observed data, and long-term model-based predictions. Vaccine 2015;33:5723–7. 10.1016/j.vaccine.2015.07.008 26190091

[1304] Hens N, Habteab Ghebretinsae A, Hardt K, Van Damme P, Van Herck K. Model based estimates of long-term persistence of inactivated hepatitis A vaccine-induced antibodies in adults. Vaccine 2014;32:1507–13. 10.1016/j.vaccine.2013.10.088 24508042

[1305] Mosites E, Seeman S, Negus S, Immunogenicity of the hepatitis A vaccine 20 years after infant immunization. Vaccine 2020;38:4940–3. 10.1016/j.vaccine.2020.05.069 32535018

[1306] Yin S, Barker L, Ly KN, Susceptibility to hepatitis A virus infection in the United States, 2007–2016. Clin Infect Dis 2020;71:e571–9. 10.1093/cid/ciaa298 32193542 PMC11009793

[1307] Moro PL, Arana J, Marquez PL, Is there any harm in administering extra-doses of vaccine to a person? Excess doses of vaccine reported to the Vaccine Adverse Event Reporting System (VAERS), 2007–2017. Vaccine 2019;37:3730–4. 10.1016/j.vaccine.2019.04.088 31155414 PMC6925972

[1308] Winokur PL, Stapleton JT. Immunoglobulin prophylaxis for hepatitis A. Clin Infect Dis 1992;14:580–6. 10.1093/clinids/14.2.580 1554845

[1309] Alter HJ, Purcell RH, Gerin JL, Transmission of hepatitis B to chimpanzees by hepatitis B surface antigen-positive saliva and semen. Infect Immun 1977;16:928–33. 10.1128/IAI.16.3.928-933.1977 892901 PMC421053

[1310] Villarejos VM, Visoná KA, Gutiérrez A, Rodríguez A. Role of saliva, urine and feces in the transmission of type B hepatitis. N Engl J Med 1974;291:1375–8. 10.1056/NEJM197412262912602 4427641

[1311] Busch K, Thimme R. Natural history of chronic hepatitis B virus infection. Med Microbiol Immunol (Berl) 2015;204:5–10. 10.1007/s00430-014-0369-7 25540037

[1312] Hyams KC. Risks of chronicity following acute hepatitis B virus infection: a review. Clin Infect Dis 1995;20:992–1000. 10.1093/clinids/20.4.992 7795104

[1313] Goldstein ST, Zhou F, Hadler SC, Bell BP, Mast EE, Margolis HS. A mathematical model to estimate global hepatitis B disease burden and vaccination impact. Int J Epidemiol 2005;34:1329–39. 10.1093/ije/dyi206 16249217

[1314] Thompson ND, Perz JF, Moorman AC, Holmberg SD. Nonhospital health care-associated hepatitis B and C virus transmission: United States, 1998–2008. Ann Intern Med 2009;150:33–9. 10.7326/0003-4819-150-1-200901060-00007 19124818

[1315] Davis LG, Weber DJ, Lemon SM. Horizontal transmission of hepatitis B virus. Lancet 1989;1:889–93. 10.1016/S0140-6736(89)92876-6 2564960

[1316] Martinson FE, Weigle KA, Royce RA, Weber DJ, Suchindran CM, Lemon SM. Risk factors for horizontal transmission of hepatitis B virus in a rural district in Ghana. Am J Epidemiol 1998;147:478–87. 10.1093/oxfordjournals.aje.a009474 9525535

[1317] CDC. Healthcare-associated hepatitis B and C outbreaks (≥2 cases) reported to the CDC 2008–2019. Atlanta, GA: US Department of Health and Human Services, CDC; 2019. https://www.cdc.gov/hepatitis/outbreaks/pdfs/HealthcareInvestigationTable.pdf

[1318] Schillie S, Harris A, Link-Gelles R, Romero J, Ward J, Nelson N. Recommendations of the Advisory Committee on Immunization Practices for use of a hepatitis B vaccine with a novel adjuvant. MMWR Morb Mortal Wkly Rep 2018;67:455–8. 10.15585/mmwr.mm6715a5 29672472 PMC6191098

[1319] Lu PJ, Byrd KK, Murphy TV, Weinbaum C. Hepatitis B vaccination coverage among high-risk adults 18–49 years, U.S., 2009. Vaccine 2011;29:7049–57. 10.1016/j.vaccine.2011.07.030 21782873

[1320] Williams WW, Lu PJ, O’Halloran A, Surveillance of vaccination coverage among adult populations—United States, 2015. MMWR Surveill Summ 2017;66(No. SS-11). 10.15585/mmwr.ss6611a1 28472027 PMC5829683

[1321] CDC. Hepatitis B vaccination coverage among adults—United States, 2004. MMWR Morb Mortal Wkly Rep 2006;55:509–11. 16691181

[1322] MacKellar DA, Valleroy LA, Secura GM, ; Young Men’s Survey Study Group. Two decades after vaccine license: hepatitis B immunization and infection among young men who have sex with men. Am J Public Health 2001;91:965–71. 10.2105/AJPH.91.6.965 11392942 PMC1446476

[1323] Terrault NA, Lok ASF, McMahon BJ, Update on prevention, diagnosis, and treatment of chronic hepatitis B: AASLD 2018 hepatitis B guidance. Hepatology 2018;67:1560–99. 10.1002/hep.29800 29405329 PMC5975958

[1324] Ezeanolue E, Harriman K, Hunter P, Kroger A, Pellegrini C. General best practice guidelines for immunization: best practices guidance of the Advisory Committee on Immunization Practices (ACIP) [Internet]. Atlanta, GA: US Department of Health and Human Services, CDC, Advisory Committee on Immunization Practices; 2020. https://www.cdc.gov/vaccines/hcp/acip-recs/general-recs/downloads/general-recs.pdf

[1325] Bruce MG, Bruden D, Hurlburt D, Antibody levels and protection after hepatitis B vaccine: results of a 30-year follow-up study and response to a booster dose. J Infect Dis 2016;214:16–22. 10.1093/infdis/jiv748 26802139

[1326] McMahon BJ, Bulkow LR, Singleton RJ, Elimination of hepatocellular carcinoma and acute hepatitis B in children 25 years after a hepatitis B newborn and catch-up immunization program. Hepatology 2011;54:801–7. 10.1002/hep.24442 21618565

[1327] Simons BC, Spradling PR, Bruden DJ, A longitudinal hepatitis B vaccine cohort demonstrates long-lasting hepatitis B virus (HBV) cellular immunity despite loss of antibody against HBV surface antigen. J Infect Dis 2016;214:273–80. 10.1093/infdis/jiw142 27056956 PMC4918827

[1328] Bohlke K, Davis RL, Marcy SM, ; Vaccine Safety Datalink Team. Risk of anaphylaxis after vaccination of children and adolescents. Pediatrics 2003;112:815–20. 10.1542/peds.112.4.815 14523172

[1329] André FE. Summary of safety and efficacy data on a yeast-derived hepatitis B vaccine. Am J Med 1989;87(3A):14S–20. 10.1016/0002-9343(89)90525-1 2528292

[1330] Abara WE, Qaseem A, Schillie S, McMahon BJ, Harris AM; High Value Care Task Force of the American College of Physicians and the Centers for Disease Control and Prevention. Hepatitis B vaccination, screening, and linkage to care: best practice advice from the American College of Physicians and the Centers for Disease Control and Prevention. Ann Intern Med 2017;167:794–804. 10.7326/M17-1106 29159414

[1331] Minuk GY, Bohme CE, Bowen TJ, Efficacy of commercial condoms in the prevention of hepatitis B virus infection. Gastroenterology 1987;93:710–4. 10.1016/0016-5085(87)90431-8 3040512

[1332] Hofmeister MG, Rosenthal EM, Barker LK, Estimating prevalence of hepatitis C virus infection in the United States, 2013–2016. Hepatology 2019;69:1020–31. 10.1002/hep.30297 30398671 PMC6719781

[1333] Lockart I, Matthews GV, Danta M. Sexually transmitted hepatitis C infection: the evolving epidemic in HIV-positive and HIV-negative MSM. Curr Opin Infect Dis 2019;32:31–7. 10.1097/QCO.0000000000000515 30531370

[1334] Terrault NA, Dodge JL, Murphy EL, Sexual transmission of hepatitis C virus among monogamous heterosexual couples: the HCV partners study. Hepatology 2013;57:881–9. 10.1002/hep.26164 23175457 PMC4384338

[1335] Price JC, McKinney JE, Crouch PC, Sexually acquired hepatitis C infection in HIV-uninfected men who have sex with men using preexposure prophylaxis against HIV. J Infect Dis 2019;219:1373–6. 10.1093/infdis/jiy670 30462305

[1336] Tohme RA, Holmberg SD. Transmission of hepatitis C virus infection through tattooing and piercing: a critical review. Clin Infect Dis 2012;54:1167–78. 10.1093/cid/cir991 22291098 PMC4613802

[1337] Brettler DB, Mannucci PM, Gringeri A, The low risk of hepatitis C virus transmission among sexual partners of hepatitis C-infected hemophilic males: an international, multicenter study. Blood 1992;80:540–3. 10.1182/blood.V80.2.540.540 1627805

[1338] Kao JH, Hwang YT, Chen PJ, Transmission of hepatitis C virus between spouses: the important role of exposure duration. Am J Gastroenterol 1996;91:2087–90. 8855726

[1339] Fierer DS, Mullen MP, Dieterich DT, Isabel Fiel M, Branch AD. Early-onset liver fibrosis due to primary hepatitis C virus infection is higher over time in HIV-infected men. Clin Infect Dis 2012;55:887–8, author reply 888–9. 10.1093/cid/cis538 22677713 PMC3697433

[1340] van de Laar TJ, van der Bij AK, Prins M, Increase in HCV incidence among men who have sex with men in Amsterdam most likely caused by sexual transmission. J Infect Dis 2007;196:230–8. 10.1086/518796 17570110

[1341] Nijmeijer BM, Koopsen J, Schinkel J, Prins M, Geijtenbeek TB. Sexually transmitted hepatitis C virus infections: current trends, and recent advances in understanding the spread in men who have sex with men. J Int AIDS Soc 2019;22(Suppl 6):e25348. 10.1002/jia2.25348 31468692 PMC6715947

[1342] Todesco E, Day N, Amiel C, High clustering of acute HCV infections and high rate of associated STIs among Parisian HIV-positive male patients. Int J Antimicrob Agents 2019;53:678–81. 10.1016/j.ijantimicag.2019.02.002 30742957

[1343] Jin F, Matthews GV, Grulich AE. Sexual transmission of hepatitis C virus among gay and bisexual men: a systematic review. Sex Health 2017;14:28–41. 10.1071/SH16141 27712618

[1344] Hegazi A, Lee MJ, Whittaker W, Chemsex and the city: sexualised substance use in gay bisexual and other men who have sex with men attending sexual health clinics. Int J STD AIDS 2017;28:362–6. Erratum in: Int J STD AIDS 2017;28:423. 10.1177/0956462416651229 27178067

[1345] Page EE, Nelson M. Hepatitis C and sex. Clin Med (Lond) 2016;16:189–92. 10.7861/clinmedicine.16-2-189 27037392 PMC4952976

[1346] Apers L, Vanden Berghe W, De Wit S, Risk factors for HCV acquisition among HIV-positive MSM in Belgium. J Acquir Immune Defic Syndr 2015;68:585–93. 10.1097/QAI.0000000000000528 25763786

[1347] Daskalopoulou M, Rodger AJ, Phillips AN, ; ASTRA Study Group. Condomless sex in HIV-diagnosed men who have sex with men in the UK: prevalence, correlates, and implications for HIV transmission. Sex Transm Infect 2017;93:590–8. 10.1136/sextrans-2016-053029 28679630 PMC5739863

[1348] Vanhommerig JW, Lambers FA, Schinkel J, ; MOSAIC (MSM Observational Study of Acute Infection With Hepatitis C) Study Group. Risk factors for sexual transmission of hepatitis C virus among human immunodeficiency virus-infected men who have sex with men: a case-control study. Open Forum Infect Dis 2015;2:ofv115. 10.1093/ofid/ofv115 26634219 PMC4665384

[1349] Turner SS, Gianella S, Yip MJ, Shedding of hepatitis C virus in semen of human immunodeficiency virus-infected men. Open Forum Infect Dis 2016;3:ofw057. 27186582 10.1093/ofid/ofw057 PMC4866572

[1350] Foster AL, Gaisa MM, Hijdra RM, Shedding of hepatitis C virus into the rectum of HIV-infected men who have sex with men. Clin Infect Dis 2017;64:284–8. 10.1093/cid/ciw740 28013267

[1351] Hammer GP, Kellogg TA, McFarland WC, Low incidence and prevalence of hepatitis C virus infection among sexually active non-intravenous drug-using adults, San Francisco, 1997–2000. Sex Transm Dis 2003;30:919–24. 10.1097/01.OLQ.0000091152.31366.E6 14646642

[1352] Roy KM, Goldberg DJ, Hutchinson S, Cameron SO, Wilson K, MacDonald L. Hepatitis C virus among self declared non-injecting sexual partners of injecting drug users. J Med Virol 2004;74:62–6. 10.1002/jmv.20146 15258969

[1353] Mele A, Stroffolini T, Tosti ME, Heterosexual transmission of hepatitis C in Italy. J Med Virol 1999;57:111–3. 10.1002/(SICI)1096-9071(199902)57:2<111::AID-JMV4>3.0.CO;2-C 9892393

[1354] Rauch A, Rickenbach M, Weber R, ; Swiss HIV Cohort Study. Unsafe sex and increased incidence of hepatitis C virus infection among HIV-infected men who have sex with men: the Swiss HIV Cohort Study. Clin Infect Dis 2005;41:395–402. 10.1086/431486 16007539

[1355] Browne R, Asboe D, Gilleece Y, Increased numbers of acute hepatitis C infections in HIV positive homosexual men; is sexual transmission feeding the increase? Sex Transm Infect 2004;80:326–7. 10.1136/sti.2003.008532 15295139 PMC1744861

[1356] Danta M, Brown D, Bhagani S, ; HIV and Acute HCV (HAAC) group. Recent epidemic of acute hepatitis C virus in HIV-positive men who have sex with men linked to high-risk sexual behaviours. AIDS 2007;21:983–91. 10.1097/QAD.0b013e3281053a0c 17457092

[1357] Ghosn J, Pierre-François S, Thibault V, Acute hepatitis C in HIV-infected men who have sex with men. HIV Med 2004;5:303–6. 10.1111/j.1468-1293.2004.00225.x 15236621

[1358] van de Laar T, Pybus O, Bruisten S, Evidence of a large, international network of HCV transmission in HIV-positive men who have sex with men. Gastroenterology 2009;136:1609–17. 10.1053/j.gastro.2009.02.006 19422083 PMC4260925

[1359] Hoornenborg E, Achterbergh RCA, Schim van der Loeff MF, ; Amsterdam PrEP Project team in the HIV Transmission Elimination AMsterdam Initiative, MOSAIC study group. MSM starting preexposure prophylaxis are at risk of hepatitis C virus infection. AIDS 2017;31:1603–10. 10.1097/QAD.0000000000001522 28657964

[1360] Gras J, Mahjoub N, Charreau I, ; IPERGAY Study Group. Early diagnosis and risk factors of acute hepatitis C in high-risk MSM on preexposure prophylaxis. AIDS 2020;34:47–52. 10.1097/QAD.0000000000002364 31789889

[1361] Hoofnagle JH. Hepatitis C: the clinical spectrum of disease. Hepatology 1997;26(Suppl 1):15S–20S. 10.1002/hep.510260703 9305658

[1362] Orland JR, Wright TL, Cooper S. Acute hepatitis C. Hepatology 2001;33:321–7. 10.1053/jhep.2001.22112 11172332

[1363] Alter MJ, Margolis HS, Krawczynski K, The natural history of community-acquired hepatitis C in the United States. The Sentinel Counties Chronic non-A, non-B Hepatitis Study Team. N Engl J Med 1992;327:1899–905. 10.1056/NEJM199212313272702 1280771

[1364] Farci P, Alter HJ, Wong D, A long-term study of hepatitis C virus replication in non-A, non-B hepatitis. N Engl J Med 1991;325:98–104. 10.1056/NEJM199107113250205 1646962

[1365] Liang TJ, Rehermann B, Seeff LB, Hoofnagle JH. Pathogenesis, natural history, treatment, and prevention of hepatitis C. Ann Intern Med 2000;132:296–305. 10.7326/0003-4819-132-4-200002150-00008 10681285

[1366] Thomas DL, Seeff LB. Natural history of hepatitis C. Clin Liver Dis 2005;9:383–98, vi. 10.1016/j.cld.2005.05.003 16023972

[1367] Westbrook RH, Dusheiko G. Natural history of hepatitis C. J Hepatol 2014;61(Suppl):S58–68. 10.1016/j.jhep.2014.07.012 25443346

[1368] Zou S, Stramer SL, Dodd RY. Donor testing and risk: current prevalence, incidence, and residual risk of transfusion-transmissible agents in US allogeneic donations. Transfus Med Rev 2012;26:119–28. 10.1016/j.tmrv.2011.07.007 21871776

[1369] Bixler D, Annambholta P, Abara WE, Hepatitis B and C virus infections transmitted through organ transplantation investigated by CDC, United States, 2014–2017. Am J Transplant 2019;19:2570–82. 10.1111/ajt.15352 30861300 PMC9112229

[1370] CDC. Testing for HCV infection: an update of guidance for clinicians and laboratorians. MMWR Morb Mortal Wkly Rep 2013;62:362–5. 23657112 PMC4605020

[1371] Marincovich B, Castilla J, del Romero J, Absence of hepatitis C virus transmission in a prospective cohort of heterosexual serodiscordant couples. Sex Transm Infect 2003;79:160–2. 10.1136/sti.79.2.160 12690143 PMC1744643

[1372] Tahan V, Karaca C, Yildirim B, Sexual transmission of HCV between spouses. Am J Gastroenterol 2005;100:821–4. 10.1111/j.1572-0241.2005.40879.x 15784025

[1373] Vandelli C, Renzo F, Romanò L, Lack of evidence of sexual transmission of hepatitis C among monogamous couples: results of a 10-year prospective follow-up study. Am J Gastroenterol 2004;99:855–9. 10.1111/j.1572-0241.2004.04150.x 15128350

[1374] Fierer DS, Uriel AJ, Carriero DC, Liver fibrosis during an outbreak of acute hepatitis C virus infection in HIV-infected men: a prospective cohort study. J Infect Dis 2008;198:683–6. 10.1086/590430 18627270 PMC4520699

[1375] Cottrell EB, Chou R, Wasson N, Rahman B, Guise JM. Reducing risk for mother-to-infant transmission of hepatitis C virus: a systematic review for the U.S. Preventive Services Task Force. Ann Intern Med 2013;158:109–13. 10.7326/0003-4819-158-2-201301150-00575 23437438

[1376] Mast EE, Hwang LY, Seto DS, Risk factors for perinatal transmission of hepatitis C virus (HCV) and the natural history of HCV infection acquired in infancy. J Infect Dis 2005;192:1880–9. 10.1086/497701 16267758

[1377] Barbosa C, Fraser H, Hoerger TJ, Cost-effectiveness of scaling-up HCV prevention and treatment in the United States for people who inject drugs. Addiction 2019;114:2267–78. 10.1111/add.14731 31307116 PMC7751348

[1378] Lambers FA, Prins M, Thomas X, ; MOSAIC (MSM Observational Study of Acute Infection with hepatitis C) study group. Alarming incidence of hepatitis C virus re-infection after treatment of sexually acquired acute hepatitis C virus infection in HIV-infected MSM. AIDS 2011;25:F21–7. 10.1097/QAD.0b013e32834bac44 21857492

[1379] Martin TC, Singh GJ, McClure M, Nelson M. HCV reinfection among HIV-positive men who have sex with men: a pragmatic approach. Hepatology 2015;61:1437. 10.1002/hep.27391 25147047

[1380] Ingiliz P, Martin TC, Rodger A, ; NEAT study group. HCV reinfection incidence and spontaneous clearance rates in HIV-positive men who have sex with men in Western Europe. J Hepatol 2017;66:282–7. 10.1016/j.jhep.2016.09.004 27650285

[1381] Briat A, Dulioust E, Galimand J, Hepatitis C virus in the semen of men coinfected with HIV-1: prevalence and origin. AIDS 2005;19:1827–35. 10.1097/01.aids.0000189847.98569.2d 16227790

[1382] Bissessor M, Fairley CK, Read T, Denham I, Bradshaw C, Chen M. The etiology of infectious proctitis in men who have sex with men differs according to HIV status. Sex Transm Dis 2013;40:768–70. 10.1097/OLQ.0000000000000022 24275725

[1383] Gutierrez-Fernandez J, Medina V, Hidalgo-Tenorio C, Abad R. Two cases of *Neisseria meningitidis* proctitis in HIV-positive men who have sex with men. Emerg Infect Dis 2017;23:542–3. 10.3201/eid2303.161039 28221124 PMC5382739

[1384] Levy I, Gefen-Halevi S, Nissan I, Delayed diagnosis of colorectal sexually transmitted diseases due to their resemblance to inflammatory bowel diseases. Int J Infect Dis 2018;75:34–8. 10.1016/j.ijid.2018.08.004 30125691

[1385] Lebari D. Syphilis presenting as colorectal cancer [Abstract 34216]. Sex Transm Dis 2014;41(Suppl 1):S4.

[1386] Hines JZ, Pinsent T, Rees K, Notes from the field: shigellosis outbreak among men who have sex with men and homeless persons—Oregon, 2015–2016. MMWR Morb Mortal Wkly Rep 2016;65:812–3. 10.15585/mmwr.mm6531a5 27513523

[1387] Marchand-Senécal X, Bekal S, Pilon PA, Sylvestre JL, Gaudreau C. Campylobacter fetus cluster among men who have sex with men, Montreal, Quebec, Canada, 2014–2016. Clin Infect Dis 2017;65:1751–3. 10.1093/cid/cix610 29020280

[1388] Klausner JD, Kohn R, Kent C. Etiology of clinical proctitis among men who have sex with men. Clin Infect Dis 2004;38:300–2. 10.1086/380838 14699467

[1389] Stoner BP, Cohen SE. Lymphogranuloma venereum 2015: clinical presentation, diagnosis, and treatment. Clin Infect Dis 2015;61(Suppl 8):S865–73. 10.1093/cid/civ756 26602624

[1390] Mohrmann G, Noah C, Sabranski M, Sahly H, Stellbrink HJ. Ongoing epidemic of lymphogranuloma venereum in HIV-positive men who have sex with men: how symptoms should guide treatment. J Int AIDS Soc 2014;17(Suppl 3):19657. 10.7448/IAS.17.4.19657 25394161 PMC4225278

[1391] Hoffmann C, Sahly H, Jessen A, High rates of quinolone-resistant strains of *Shigella sonnei* in HIV-infected MSM. Infection 2013;41:999–1003. 10.1007/s15010-013-0501-4 23852945

[1392] Heiman KE, Karlsson M, Grass J, ; CDC. Notes from the field: *Shigella* with decreased susceptibility to azithromycin among men who have sex with men—United States, 2002–2013. MMWR Morb Mortal Wkly Rep 2014;63:132–3. 24522098 PMC4584870

[1393] Galiczynski EM Jr, Elston DM. What’s eating you? Pubic lice (*Pthirus pubis*). Cutis 2008;81:109–14. 18441761

[1394] Meinking TL, Serrano L, Hard B, Comparative in vitro pediculicidal efficacy of treatments in a resistant head lice population in the United States. Arch Dermatol 2002;138:220–4. 10.1001/archderm.138.2.220 11843643

[1395] Yoon KS, Gao JR, Lee SH, Clark JM, Brown L, Taplin D. Permethrin-resistant human head lice, *Pediculus capitis*, and their treatment. Arch Dermatol 2003;139:994–1000. 10.1001/archderm.139.8.994 12925385

[1396] Burkhart CG, Burkhart CN. Oral ivermectin for *Phthirus pubis.* J Am Acad Dermatol 2004;51:1037–8. 10.1016/j.jaad.2004.04.041 15583618

[1397] Scott GR, Chosidow O; IUSTI/WHO. European guideline for the management of pediculosis pubis, 2010. Int J STD AIDS 2011;22:304–5. 10.1258/ijsa.2011.011114 21680662

[1398] Goldust M, Rezaee E, Raghifar R, Hemayat S. Comparing the efficacy of oral ivermectin vs malathion 0.5% lotion for the treatment of scabies. Skinmed 2014;12:284–7. 25632646

[1399] Veraldi S, Schianchi R, Ramoni S, Nazzaro G. Pubic hair removal and *Phthirus pubis* infestation. Int J STD AIDS 2018;29:103–4. 10.1177/0956462417740292 29130406

[1400] Leung AKC, Lam JM, Leong KF. Scabies: a neglected global disease. Curr Pediatr Rev 2020;16:33–42. 10.2174/1573396315666190717114131 31544694

[1401] Engelman D, Cantey PT, Marks M, The public health control of scabies: priorities for research and action. Lancet 2019;394:81–92. 10.1016/S0140-6736(19)31136-5 31178154 PMC11257500

[1402] Shimose L, Munoz-Price LS. Diagnosis, prevention, and treatment of scabies. Curr Infect Dis Rep 2013;15:426–31. 10.1007/s11908-013-0354-0 23904181

[1403] Walter B, Heukelbach J, Fengler G, Worth C, Hengge U, Feldmeier H. Comparison of dermoscopy, skin scraping, and the adhesive tape test for the diagnosis of scabies in a resource-poor setting. Arch Dermatol 2011;147:468–73. 10.1001/archdermatol.2011.51 21482897

[1404] Micali G, Lacarrubba F, Verzì AE, Chosidow O, Schwartz RA. Scabies: advances in noninvasive diagnosis. PLoS Negl Trop Dis 2016;10:e0004691. 10.1371/journal.pntd.0004691 27311065 PMC4911127

[1405] Strong M, Johnstone P. Interventions for treating scabies. Cochrane Database Syst Rev 2007;(3):CD000320. 17636630 10.1002/14651858.CD000320.pub2 PMC6532717

[1406] Abdel-Raheem TA, Méabed EM, Nasef GA, Abdel Wahed WY, Rohaim RM. Efficacy, acceptability and cost effectiveness of four therapeutic agents for treatment of scabies. J Dermatolog Treat 2016;27:473–9. 10.3109/09546634.2016.1151855 27027929

[1407] Alipour H, Goldust M. The efficacy of oral ivermectin vs. sulfur 10% ointment for the treatment of scabies. Ann Parasitol 2015;61:79–84. 26342502

[1408] Al Jaff DAA, Amin MHM. Comparison of the effectiveness of sulphur ointment, permethrin and oral ivermectin in treatment of scabies. Res J Pharm Biol Chem Sci 2018;9:670–6.

[1409] Goldust M, Rezaee E, Raghifar R, Hemayat S. Treatment of scabies: the topical ivermectin vs. permethrin 2.5% cream. Ann Parasitol 2013;59:79–84. 24171301

[1410] Ahmad HM, Abdel-Azim ES, Abdel-Aziz RT. Clinical efficacy and safety of topical versus oral ivermectin in treatment of uncomplicated scabies. Dermatol Ther (Heidelb) 2016;29:58–63. 10.1111/dth.12310 26555785

[1411] Currie BJ, McCarthy JS. Permethrin and ivermectin for scabies. N Engl J Med 2010;362:717–25. 10.1056/NEJMct0910329 20181973

[1412] Chiu S, Argaez C. Ivermectin for parasitic skin infections of scabies: a review of comparative clinical effectiveness, cost-effectiveness, and guidelines. Ottawa, ON: Canadian Agency for Drugs and Technologies in Health; 2019. https://www.ncbi.nlm.nih.gov/books/NBK545083/31424718

[1413] Nolan K, Kamrath J, Levitt J. Lindane toxicity: a comprehensive review of the medical literature. Pediatr Dermatol 2012;29:141–6. 10.1111/j.1525-1470.2011.01519.x 21995612

[1414] Mounsey KE, Holt DC, McCarthy J, Currie BJ, Walton SF. Scabies: molecular perspectives and therapeutic implications in the face of emerging drug resistance. Future Microbiol 2008;3:57–66. 10.2217/17460913.3.1.57 18230034

[1415] Mounsey KE, Holt DC, McCarthy JS, Currie BJ, Walton SF. Longitudinal evidence of increasing in vitro tolerance of scabies mites to ivermectin in scabies-endemic communities. Arch Dermatol 2009;145:840–1. 10.1001/archdermatol.2009.125 19620572

[1416] Mounsey KE, McCarthy JS, Walton SF. Scratching the itch: new tools to advance understanding of scabies. Trends Parasitol 2013;29:35–42. 10.1016/j.pt.2012.09.006 23088958

[1417] van der Linden N, van Gool K, Gardner K, A systematic review of scabies transmission models and data to evaluate the cost-effectiveness of scabies interventions. PLoS Negl Trop Dis 2019;13:e0007182. 10.1371/journal.pntd.0007182 30849124 PMC6426261

[1418] Roberts LJ, Huffam SE, Walton SF, Currie BJ. Crusted scabies: clinical and immunological findings in seventy-eight patients and a review of the literature. J Infect 2005;50:375–81. 10.1016/j.jinf.2004.08.033 15907543

[1419] Ortega-Loayza AG, McCall CO, Nunley JR. Crusted scabies and multiple dosages of ivermectin. J Drugs Dermatol 2013;12:584–5. 23652958

[1420] Bouvresse S, Chosidow O. Scabies in healthcare settings. Curr Opin Infect Dis 2010;23:111–8. 10.1097/QCO.0b013e328336821b 20075729

[1421] Marotta M, Toni F, Dallolio L, Toni G, Leoni E. Management of a family outbreak of scabies with high risk of spread to other community and hospital facilities. Am J Infect Control 2018;46:808–13. 10.1016/j.ajic.2017.12.004 29397231

[1422] Romani L, Whitfeld MJ, Koroivueta J, Mass drug administration for scabies control in a population with endemic disease. N Engl J Med 2015;373:2305–13. 10.1056/NEJMoa1500987 26650152

[1423] Ackerman DR, Sugar NF, Fine DN, Eckert LO. Sexual assault victims: factors associated with follow-up care. Am J Obstet Gynecol 2006;194:1653–9. 10.1016/j.ajog.2006.03.014 16635464

[1424] Parekh V, Beaumont Brown C. Follow up of patients who have been recently sexually assaulted. Sex Transm Infect 2003;79:349. 10.1136/sti.79.4.349-a 12902602 PMC1744715

[1425] Vrees RA. Evaluation and management of female victims of sexual assault. Obstet Gynecol Surv 2017;72:39–53. 10.1097/OGX.0000000000000390 28134394

[1426] Unger ER, Fajman NN, Maloney EM, Anogenital human papillomavirus in sexually abused and nonabused children: a multicenter study. Pediatrics 2011;128:e658–65. 10.1542/peds.2010-2247 21844060

[1427] Kreimer AR, Rodriguez AC, Hildesheim A, ; CVT Vaccine Group. Proof-of-principle evaluation of the efficacy of fewer than three doses of a bivalent HPV16/18 vaccine. J Natl Cancer Inst 2011;103:1444–51. 10.1093/jnci/djr319 21908768 PMC3186781

[1428] Claydon E, Murphy S, Osborne EM, Kitchen V, Smith JR, Harris JR. Rape and HIV. Int J STD AIDS 1991;2:200–1. 10.1177/095646249100200310 1863649

[1429] Murphy S, Kitchen V, Harris JR, Forster SM. Rape and subsequent seroconversion to HIV. BMJ 1989;299:718. 10.1136/bmj.299.6701.718 2508885 PMC1837511

[1430] Cardo DM, Culver DH, Ciesielski CA, ; CDC Needlestick Surveillance Group. A case-control study of HIV seroconversion in health care workers after percutaneous exposure. N Engl J Med 1997;337:1485–90. 10.1056/NEJM199711203372101 9366579

[1431] Kuhar DT, Henderson DK, Struble KA, ; US Public Health Service Working Group. Updated US Public Health Service guidelines for the management of occupational exposures to human immunodeficiency virus and recommendations for postexposure prophylaxis. Infect Control Hosp Epidemiol 2013;34:875–92. 10.1086/672271 23917901

[1432] Du Mont J, Myhr TL, Husson H, Macdonald S, Rachlis A, Loutfy MR. HIV postexposure prophylaxis use among Ontario female adolescent sexual assault victims: a prospective analysis. Sex Transm Dis 2008;35:973–8. 18836390 10.1097/OLQ.0b013e3181824f3c

[1433] Neu N, Heffernan-Vacca S, Millery M, Stimell M, Brown J. Postexposure prophylaxis for HIV in children and adolescents after sexual assault: a prospective observational study in an urban medical center. Sex Transm Dis 2007;34:65–8. 10.1097/01.olq.0000225329.07765.d8 16794560

[1434] Loutfy MR, Macdonald S, Myhr T, Prospective cohort study of HIV post-exposure prophylaxis for sexual assault survivors. Antivir Ther 2008;13:87–95. 18389902 10.1177/135965350801300109

[1435] Inciarte A, Leal L, Masfarre L, ; Sexual Assault Victims Study Group. Post-exposure prophylaxis for HIV infection in sexual assault victims. HIV Med 2020;21:43–52. 10.1111/hiv.12797 31603619 PMC6916272

[1436] Announcement: Updated guidelines for antiretroviral postexposure prophylaxis after sexual, injection-drug use, or other nonoccupational exposure to HIV—United States, 2016. MMWR Morb Mortal Wkly Rep 2016;65:458. 10.15585/mmwr.mm6517a5 27149423

[1437] Ford N, Venter F, Irvine C, Beanland RL, Shubber Z. Starter packs versus full prescription of antiretroviral drugs for postexposure prophylaxis: a systematic review. Clin Infect Dis 2015;60(Suppl 3):S182–6. 10.1093/cid/civ093 25972501

[1438] Jenny C, Crawford-Jakubiak JE; Committee on Child Abuse and Neglect; American Academy of Pediatrics. The evaluation of children in the primary care setting when sexual abuse is suspected. Pediatrics 2013;132:e558–67. 10.1542/peds.2013-1741 23897912

[1439] Girardet RG, Lahoti S, Howard LA, Epidemiology of sexually transmitted infections in suspected child victims of sexual assault. Pediatrics 2009;124:79–86. 10.1542/peds.2008-2947 19564286

[1440] Black CM, Driebe EM, Howard LA, Multicenter study of nucleic acid amplification tests for detection of *Chlamydia trachomatis* and *Neisseria gonorrhoeae* in children being evaluated for sexual abuse. Pediatr Infect Dis J 2009;28:608–13. 10.1097/INF.0b013e31819b592e 19451856

[1441] Trotman GE, Young-Anderson C, Deye KP. Acute sexual assault in the pediatric and adolescent population. J Pediatr Adolesc Gynecol 2016;29:518–26. 10.1016/j.jpag.2015.05.001 26702774

[1442] Schwandt A, Williams C, Beigi RH. Perinatal transmission of *Trichomonas vaginalis*: a case report. J Reprod Med 2008;53:59–61. 18251366

[1443] Bell TA, Stamm WE, Wang SP, Kuo CC, Holmes KK, Grayston JT. Chronic *Chlamydia trachomatis* infections in infants. JAMA 1992;267:400–2. 10.1001/jama.1992.03480030078041 1727964

[1444] Adachi K, Nielsen-Saines K, Klausner JD. *Chlamydia trachomatis* infection in pregnancy: the global challenge of preventing adverse pregnancy and infant outcomes in sub-Saharan Africa and Asia. BioMed Res Int 2016;2016:9315757. 10.1155/2016/9315757 27144177 PMC4837252

[1445] Schachter J, Grossman M, Sweet RL, Holt J, Jordan C, Bishop E. Prospective study of perinatal transmission of *Chlamydia trachomatis.* JAMA 1986;255:3374–7. 10.1001/jama.1986.03370240044034 3712696

[1446] Smith EM, Swarnavel S, Ritchie JM, Wang D, Haugen TH, Turek LP. Prevalence of human papillomavirus in the oral cavity/oropharynx in a large population of children and adolescents. Pediatr Infect Dis J 2007;26:836–40. 10.1097/INF.0b013e318124a4ae 17721381

[1447] Sabeena S, Bhat P, Kamath V, Arunkumar G. Possible non-sexual modes of transmission of human papilloma virus. J Obstet Gynaecol Res 2017;43:429–35. 10.1111/jog.13248 28165175

[1448] Adams JA, Farst KJ, Kellogg ND. Interpretation of medical findings in suspected child sexual abuse: an update for 2018. J Pediatr Adolesc Gynecol 2018;31:225–31. 10.1016/j.jpag.2017.12.011 29294380

[1449] Kellogg ND, Melville JD, Lukefahr JL, Nienow SM, Russell EL. Genital and extragenital gonorrhea and chlamydia in children and adolescents evaluated for sexual abuse. Pediatr Emerg Care 2018;34:761–6. 10.1097/PEC.0000000000001014 28072668

[1450] Gavril AR, Kellogg ND, Nair P. Value of follow-up examinations of children and adolescents evaluated for sexual abuse and assault. Pediatrics 2012;129:282–9. 10.1542/peds.2011-0804 22291113

[1451] Bandea CI, Joseph K, Secor EW, Development of PCR assays for detection of *Trichomonas vaginalis* in urine specimens. J Clin Microbiol 2013;51:1298–300. 10.1128/JCM.03101-12 23390274 PMC3666790

[1452] Gallion HR, Dupree LJ, Scott TA, Arnold DH. Diagnosis of *Trichomonas vaginalis* in female children and adolescents evaluated for possible sexual abuse: a comparison of the InPouch TV culture method and wet mount microscopy. J Pediatr Adolesc Gynecol 2009;22:300–5. 10.1016/j.jpag.2008.12.006 19576816

[1453] Lalor K, McElvaney R. Child sexual abuse, links to later sexual exploitation/high-risk sexual behavior, and prevention/treatment programs. Trauma Violence Abuse 2010;11:159–77. 10.1177/1524838010378299 20679329

[1454] Girardet RG, Lemme S, Biason TA, Bolton K, Lahoti S. HIV post-exposure prophylaxis in children and adolescents presenting for reported sexual assault. Child Abuse Negl 2009;33:173–8. 10.1016/j.chiabu.2008.05.010 19324415

[1455] Panel on Antiretroviral Therapy and Medical Management of HIV-Infected Children. Guidelines for the use of antiretroviral agents in pediatric HIV infection. Washington, DC: US Department of Health and Human Services, National Institutes of Health, AIDSinfo; 2020. https://aidsinfo.nih.gov/contentfiles/lvguidelines/pediatricguidelines.pdf

