# Erratum: Vol. 70, No. RR-4

**DOI:** 10.15585/mmwr.mm7204a5

**Published:** 2023-01-27

In the Recommendation and Report “Sexually Transmitted Infections Treatment Guidelines, 2021,” multiple errors occurred.

On page 5 under External Condoms, in the first paragraph, the callout for reference 23 should have been for reference 489: “**Magaret AS, Mujugira A, Hughes JP, et al.; Partners in Prevention HSV/HIV Transmission Study Team. Effect of condom use on per-act HSV-2 transmission risk in HIV-1, HSV-2-discordant couples. Clin Infect Dis 2016;62:456–61.**”

On page 23 under Viral Hepatitis, the paragraph should have read, “All persons housed in juvenile and adult correctional facilities should be screened at entry for **hepatitis B and hepatitis C. All persons who are susceptible to HBV infection should be offered hepatitis B vaccine, per ACIP recommendations (**https://www.cdc.gov/vaccines/hcp/acip-recs/vacc-specific/hepb.html**). During outbreaks in the facility or the surrounding community, all unvaccinated persons should be offered the hepatitis A vaccine; regardless of outbreak conditions, all persons who are at risk for HAV infection or severe disease should be offered hepatitis A vaccine, per ACIP recommendations (**https://www.cdc.gov/vaccines/hcp/acip-recs/vacc-specific/hepa.html).”

On page 35 under Antiviral-Resistant HSV Infection, the fifth sentence should have read, “Foscarnet (**80–120 mg/kg/day IV in 2–3 divided doses; for example,** 40 mg/kg body weight IV every 8 hours until clinical resolution is attained) is the treatment of choice for acyclovir-resistant genital herpes (*508*,*509*).”

On page 43 under Penicillin Allergy, the next to last sentence in the first paragraph should have read “in **multiple geographic** areas” and the citations should have read (***23,****606–608*).” 

 On page 55 in the paragraph under the Recommended Regimen for Congenital Syphilis Among Infants and Children box, the number of weekly doses in the first sentence should have read “**up to** 3.”

On page 65 under Pregnancy, the sentence should have read, “Diagnosis and treatment of cervicitis for pregnant women **should follow treatment recommendations for chlamydia and gonorrhea (see Chlamydial Infections, Special Considerations, Pregnancy; Gonococcal Infections, Special Considerations, Pregnancy)**.” 

On page 68 under Pregnancy, the third sentence in the second paragraph should have read “during the third **trimester**.”

On page 73 under Uncomplicated Gonococcal Infection of the Cervix, Urethra, or Rectum, the Alternative Regimens box should have read, “Alternative Regimen if Ceftriaxone Is Not Available or Not Feasible; Cefixime* 800 mg orally in a single dose; *If chlamydial infection has not been excluded, providers should treat for chlamydia with doxycycline 100 mg orally 2 times/day for 7 days. Alterative Regimen if Cephalosporin Allergy; Gentamicin 240 mg IM in a single dose *plus* Azithromycin 2 g orally in a single dose.”

On page 77 under Disseminated Gonococcal Infection, the fifth sentence should have read “NAATS **and** culture.” Under Treatment of Arthritis and Arthritis-Dermatitis Syndrome in the sentence under the Alternative Regimens box, the total treatment course should have read “**at least** 7 days.” Under Treatment of Gonococcal Meningitis and Endocarditis, the Recommended Regimen of ceftriaxone should have read “every **12–**24 hours.”

On page 79 under Treatment in the Absence of Signs of Gonococcal Infection, the Recommended Regimen of ceftriaxone should have read “**25**–50 mg/kg.”

On page 87 under Trichomoniasis in the first sentence, the number of persons affected in the United States should have read “approximately **2.6** million.” The first reference cited in the last sentence of the second paragraph should have read “***910***.”

On page 88 under Diagnostic Considerations in the second sentence of the second paragraph, the manufacturer of the Aptima *T. vaginalis* assay should have read “**Hologic**.”

On page 89 in the first sentence under Follow-Up, recommended retesting should have read “**approximately** 3 months.”

On page 90 under Pregnancy, the last sentence in the first paragraph should have read “sub-**Saharan Africa**.”

On page 96 under Alternative Parenteral Regimens, the first sentence of the last paragraph should have read “after 24–**48** hours.”

On page 97 under Alternative Intramuscular or Oral Regimens, the third sentence should have read, “However, if the patient has cephalosporin allergy, the community prevalence and individual risk for gonorrhea are low, and follow-up is likely, alternative therapy can be considered **with one of the following alternative regimens: 1) levofloxacin 500 mg orally once daily in combination with metronidazole 500 mg orally 2 times/day for 14 days, 2) moxifloxacin 400 mg orally once daily for 14 days, or 3) azithromycin 500 mg IV daily for 1–2 doses, followed by 250 mg orally daily for a total azithromycin duration of 7 days or in combination with metronidazole 500 mg 3 times/day for 12–14 days (*1178–1181*)**.”

On page 103 in the second sentence of the paragraph after the Recommended Regimens box, the duration of treatment should have read “**up to** 16 weeks.” In the third sentence, the duration of treatment should have read “**up to** 8 weeks.”

On page 127 under Treatment, the regimen “***or* Ivermectin 1% lotion applied to all areas of the body from the neck down and washed off after 8–14 hours; repeat treatment in 1 week if symptoms persist**” in the Recommended Regimens box should have been removed because that formulation is not available in the United States.

On page 172, reference 1103 should have read “**1102**,” reference 1104 should have read “**1103**,” and reference 1105 should have read “**1104**.”

